# Antidepressant Mechanism of Traditional Chinese Medicine Formula Xiaoyaosan in CUMS-Induced Depressed Mouse Model via RIPK1-RIPK3-MLKL Mediated Necroptosis Based on Network Pharmacology Analysis

**DOI:** 10.3389/fphar.2021.773562

**Published:** 2021-11-19

**Authors:** Zhi-Yi Yan, Hai-Yan Jiao, Jian-Bei Chen, Kai-Wen Zhang, Xi-Hong Wang, You-Ming Jiang, Yue-Yun Liu, Zhe Xue, Qing-Yu Ma, Xiao-Juan Li, Jia-Xu Chen

**Affiliations:** ^1^ School of Traditional Chinese Medicine, Beijing University of Chinese Medicine, Beijing, China; ^2^ Dongfang Hospital Affiliated to Beijing University of Chinese Medicine, Beijing, China; ^3^ Formula-pattern Research Center, School of Traditional Chinese Medicine, Jinan University, Guangzhou, China

**Keywords:** xiaoyaosan, chronic unpredictable mild stress, depression, necroptosis, neuroinflammation, microglial activation

## Abstract

**Background:** Depression is a stress-related disorder that seriously threatens people’s physical and mental health. Xiaoyaosan is a classical traditional Chinese medicine formula, which has been used to treat mental depression since ancient times. More and more notice has been given to the relationship between the occurrence of necroptosis and the pathogenesis of mental disorders.

**Objective:** The purpose of present study is to explore the potential mechanism of Xiaoyaosan for the treatment of depression using network pharmacology and experimental research, and identify the potential targets of necroptosis underlying the antidepressant mechanism of Xiaoyaosan.

**Methods:** The mice model of depression was induced by chronic unpredictable mild stress (CUMS) for 6 weeks. Adult C57BL/6 mice were randomly divided into five groups, including control group, chronic unpredictable mild stress group, Xiaoyaosan treatment group, necrostatin-1 (Nec-1) group and solvent group. Drug intervention performed from 4^th^ to 6^th^ week of modeling. The mice in Xiaoyaosan treatment group received Xiaoyaosan by intragastric administration (0.254 g/kg/d), and mice in CUMS group received 0.5 ml physiological saline. Meanwhile, the mice in Nec-1 group were injected intraperitoneally (i.p.) with Nec-1 (10 mg/kg/d), and the equivalent volume of DMSO/PBS (8.3%) was injected into solvent group mice. The behavior tests such as sucrose preference test, forced swimming test and novelty-suppressed feeding test were measured to evaluate depressive-like behaviors of model mice. Then, the active ingredients in Xiaoyaosan and the related targets of depression and necroptosis were compiled through appropriate databases, while the “botanical drugs-active ingredients-target genes” network was constructed by network pharmacology analysis. The expressions of RIPK1, RIPK3, MLKL, *p*-MLKL were detected as critical target genes of necroptosis and the potential therapeutic target compounds of Xiaoyaosan. Furthermore, the levels of neuroinflammation and microglial activation of hippocampus were measured by detecting the expressions of IL-1β, Lipocalin-2 and IBA1, and the hematoxylin and eosin (H&E) stained was used to observe the morphology in hippocampus sections.

**Results:** After 6-weeks of modeling, the behavioral data showed that mice in CUMS group and solvent group had obvious depressive-like behaviors, and the medication of Xiaoyaosan or Nec-1 could improve these behavioral changes. A total of 96 active ingredients in Xiaoyaosan which could regulate the 23 key target genes were selected from databases. Xiaoyaosan could alleviate the core target genes in necroptosis and improve the hippocampal function and neuroinflammation in depressed mice.

**Conclusion:** The activation of necroptosis existed in the hippocampus of CUMS-induced mice, which was closely related to the pathogenesis of depression. The antidepressant mechanism of Xiaoyaosan included the regulation of multiple targets in necroptosis. It also suggested that necroptosis could be a new potential target for the treatment of depression.

## Introduction

Depression is a common clinical mental disorder characterized by constant down in spirits and cognitive impairment, and the prevalence of which is increasing year by year with the lifetime prevalence as high as 10–15% (Moussavi et al., 2007; Chiriţă et al., 2015). According to the latest statistics of the World Health Organization (WHO) in 2019, more than 350 million people worldwide suffer from depression, which has covered all age groups and increased by more than 18% from 2005 to 2015. It is also a major factor contributing to the increase in global burden of disease and medical expenses.

The classic Chinese formula Xiaoyaosan, originated from the book *Prescriptions of the Bureau of Taiping People’s Welfare Pharmacy* in Song Dynasty of China, is one of the effective traditional Chinese compound recipes in treating psycho-emotional related symptoms and diseases (Zhang et al., 2019). According to the recent clinical and basic studies, Xiaoyaosan has a multi-target regulating effect which is reflected in the treatment of depression-related diseases (Li et al., 2015; Jiao et al., 2019; Xiong et al., 2019; Liu et al., 2020; Zhou et al., 2021). In the pathogenesis study of depression, it is found that psychological and physical stressors can activate the immune system, lead to inflammation, and then participate in the development of depression (Iwata et al., 2013). Overproduction of pro-inflammatory cytokines is a key factor in the interaction between immune system and central nervous system (CNS) (Dowlati et al., 2010). Microglia is innate immune-related glial cell in CNS, which is the most widely-investigated in stress-related neuroinflammation (Sawada et al., 2014). Meanwhile, many studies have confirmed that inhibition of necroptosis can provide neuroprotective effect, so necroptosis is also a potential target for the treatment of CNS-related diseases (Liu et al., 2015). Xiaoyaosan has also been shown to play a therapeutic role by improving inflammation and regulating the function of hippocampal microglia (Li et al., 2017; Jiao et al., 2021).

Necroptosis is an active cell death process characterized by cell necrosis and inflammation. Studies have confirmed that necroptosis has a unique signal transduction pathway, which is closely related to the function of receptor interacting protein kinase 1 (RIPK1) and RIPK3(Galluzzi et al., 2009). Necroptosis is often accompanied by significant inflammatory response, characterized by a large number of inflammatory cell infiltration and activation of inflammatory pathways (Newton and Manning, 2016). The formation of a RIPK1–RIPK3–mixed lineage kinase domain-like (MLKL) complex leads to the initiation of necroptosis, while the phosphorylation of MLKL protein by RIPK3 eventually leads to necroptosis by destroying plasma membrane and cytolysis (Cho et al., 2009; Shan et al., 2018). Moreover, inflammatory response is triggered by the release of intracellular contents from necrotic cells, and inflammatory factors further promote cell death, which forms a circulatory circuit (Pasparakis et al., 2015). Here, Necrostatin-1 (Nec-1) as an effective, selective and permeable necroptosis inhibitor, can effectively block the active sites of RIPK1 and RIPK3, thus inhibit the necroptosis pathway by controlling the phosphorylation (Degterev et al., 2008). Therefore, the detection of RIPK1, RIPK3 and MLKL can verify the occurrence of necroptosis, and the effective measurement to regulate necroptosis may alleviate the inflammatory response of the lesion regions, thereby slowing down the pathological changes of the corresponding diseases (Schwabe and Luedde, 2018).

**FIGURE 1:**
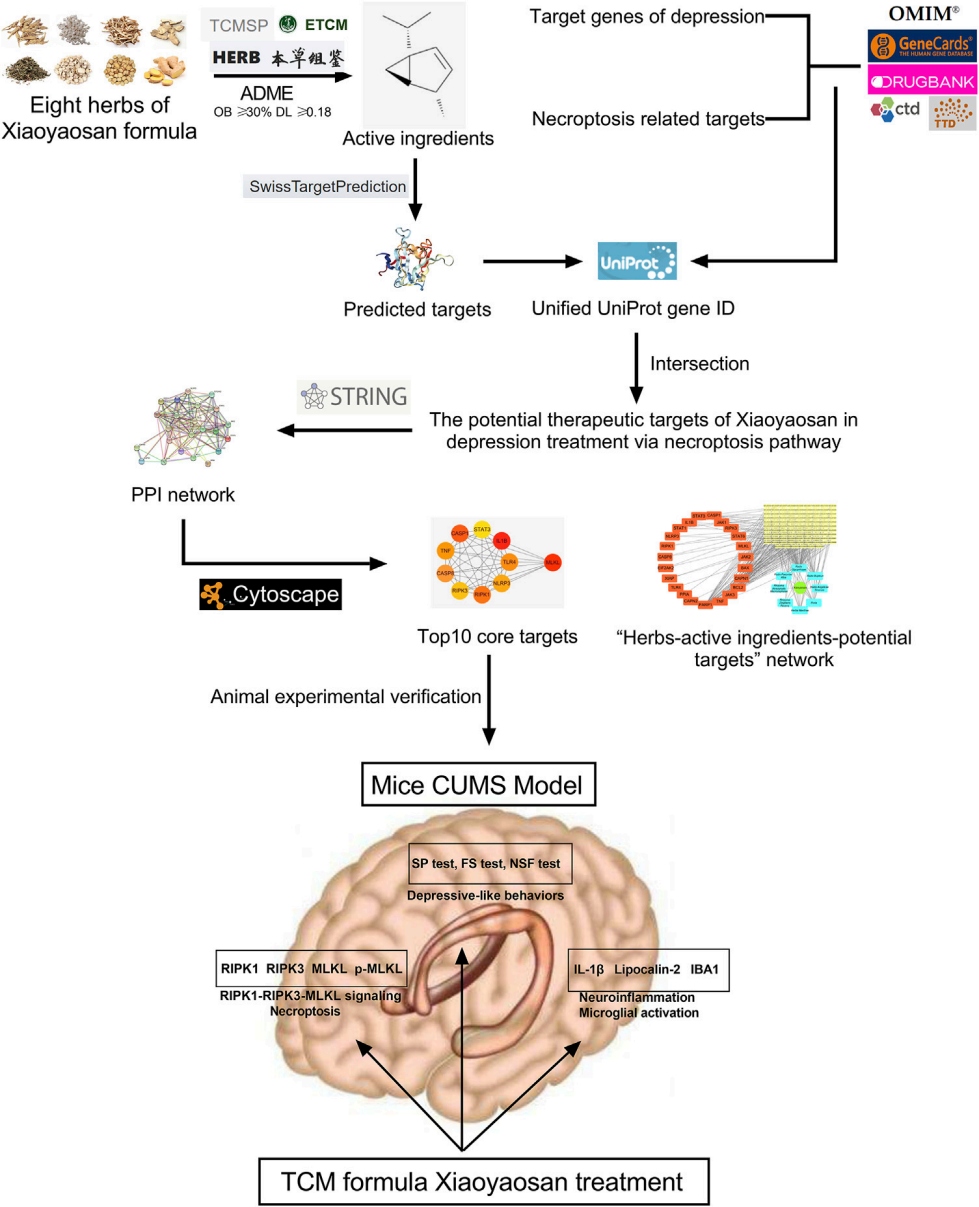
Flow chart of network pharmacological analysis and experimental study of Xiaoyaosan for depression treatment through necroptosis pathway.

Under the guidance of the overall concept of traditional Chinese medicine (TCM) and the thought of syndrome differentiation and treatment, TCM formula is a treatment method with specific therapeutic efficacy based on the principles of drug properties, drug properties and compatibility, which contains profound and complex scientific connotation (Wang et al., 2008). However, due to the lack of quantifiable and objective data support, it is very difficult to clarify the mechanism of TCM formula, which has become an important reason why TCM formula is difficult to be accepted by the international community (Luo et al., 2020). Network pharmacology, a new discipline to reveal the mechanism of diseases and the pharmacodynamic mechanism of drugs from the perspective of biological network, is well meet the concept of holism in TCM, while the core element of network pharmacology –“network target, multi-components” theory was also proposed by TCM researchers (Li and Zhang, 2013). Nowadays, network pharmacology has become an important breakthrough in the cross-innovation of information science and medical science, and has been widely used in the research of traditional Chinese medicine (Li et al., 2014; Guo et al., 2021; Sheng et al., 2021). Thus far, studies have shown that necroptosis plays an important role in neuroinflammation and central nervous system related diseases (Chen et al., 2021; Thadathil et al., 2021), and the role of necroptosis remains largely unexamined in the depression-related CNS mechanism. Also, the antidepressant effect of Xiaoyaosan has still not yet been comprehensively studied. Therefore, this study utilized network pharmacology approach to explore the therapeutic effects of Xiaoyaosan on depression via necroptosis pathway, then the antidepressant mechanism was observed *in vivo* using CUMS mice. Figure 1 showed the flow chart of the study.

## Methods

### Preparation of Traditional Chinese Medicine

TCM formula Xiaoyaosan consists of the following eight Chinese botanical drugs: Bupleurum chinense DC (Description: Apiaceae), Angelica sinensis (Oliv.) Diels (Description: Apiaceae), Paeonia lactiflora Pall (Description: Paeoniaceae), Poria cocos (Schw.) Wolf (Description: Polyporaceae), Atractylodes macrocephala Koidz (Description: Compositae), Glycyrrhiza uralensis Fisch (Description: Leguminosea), Zingiber officinale Rosc (Description: Zingiberaceae), and Mentha haplocalyx Briq (Description: Lamiaceae) at a ratio of 5:5:5:5:5:4:5:1. The prepared drug in pieces of Xiaoyaosan were purchased from Beijing Tongrentang (Bozhou) Pieces Co. Ltd., Bozhou, China, while the ultra-performance chromatography-electrospray tandem mass spectrometry (UPLC-MS/MS) was used to identify the samples of these decoction pieces (Ding et al., 2017; Yuan et al., 2020). The Xiaoyaosan dry powder (J2447) used in experimental studies was prepared by Jiuzhitang Co. Ltd, Changsha, China, in accord with the process recorded in the Chinese Pharmacopoeia 2015 Edition (Pan et al., 2019; Zhu et al., 2019). 2.36 g of crude drug yielded One Gram the finished dry powder administered experimentally.

### Network Pharmacology Approach

The active ingredients and potential targets of TCM formula Xiaoyaosan for application to depression were investigated based on network pharmacology. The active ingredients in Xiaoyaosan were screened according to absorption, distribution, metabolism and enhancement (ADME) of drugs, while the bioavailability (OB) and drug likeness (DL) were used as screening parameters (Vugmeyster et al., 2012). According to ADME (OB ≥ 30% and DL ≥ 0.18), the active ingredients of Xiaoyaosan were collected from traditional Chinese medicine databases including the TCM Systems Pharmacology database and Analysis Platform (TCMSP, https://tcmsp-e.com/) (Ru et al., 2014), the Encyclopedia of Traditional Chinese Medicine (ETCM, http://www.tcmip.cn/ETCM/) (Zhang et al., 2019) and the High-throughput Experiment- and Reference-guided database of Traditional Chinese Medicine (HERB, http://herb.ac.cn/) (Fang et al., 2021), then SwissTargetPrediction database (http://www.swisstargetprediction.ch/) (Daina et al., 2019) was used to predict the targets of active ingredients in Xiaoyaosan, the reliability screening of predicted targets was also carried out (Probability ≥30%).

The related genes with depression and necroptosis pathway were collected from GeneCards database (https://www.genecards.org/) (Stelzer et al., 2016), Online Mendelian Inheritance in Man (OMIM, https://omim.org/) (Amberger et al., 2015), Comparative Toxicogenomic database (CTD, http://ctdbase.org/) (Davis et al., 2020), Therapeutic Target database (TTD, http://db.idrblab.net/ttd/) (Wang et al., 2020), and DrugBank database (http://www.drugbank.ca/) (Wishart et al., 2018), using “Depression”, “Depressive disorder” or “Necroptosis” as the search term. Then, the gene symbols of the ingredients in Xiaoyaosan, depression and necroptosis pathway were searched in the universal Protein Resource (UniProt, https://www.UniProt.org/) to get the unified UniProt gene ID.

The potential therapeutic targets of Xiaoyaosan in depression treatment via necroptosis pathway were obtained by overlapping the predicted targets of the ingredients in Xiaoyaosan and the target genes of depression and necroptosis pathway. Furthermore, the STRING database (https://string-db.org/) (Szklarczyk et al., 2019) was used to generate the protein–protein interaction (PPI) network of potential target genes of Xiaoyaosan with the species limited to human (Homo sapiens) and the interaction score ≥0.4. Then, the Cytoscape software (version 3.7.2, http://www.cytoscape.org/, Boston, MA, United States) was used to construct the “botanical drugs-active ingredients-potential targets” network, and identify the core target genes from the results of PPI network with the cytoHubba plugin (Guo et al., 2021).

### Animals

The study was approved by the Institutional Animal Care and Use Committee of Beijing University of Chinese Medicine (BUCM-4-2018120401-4053). Experimental protocols applied in our study were performed in agreement with the existing current animal welfare guidelines. Specific-pathogen free (SPF) male C57BL/6 mice aged 8-week-old (SYXK (Jing) 2016-0006, Beijing Vital River Laboratory Animal Technology Limited Company, Beijing, China) were housed separately in a standard animal feeding room (room temperature: 22 ± 2°C; relative humidity: 30–40%; light condition: a 12 h/12 h dark/light cycle) and fed standard rodent diet. Animals were adapted to their new environment for 7 days before CUMS procedure.

### CUMS Model and Medication Process

**FIGURE 2:**
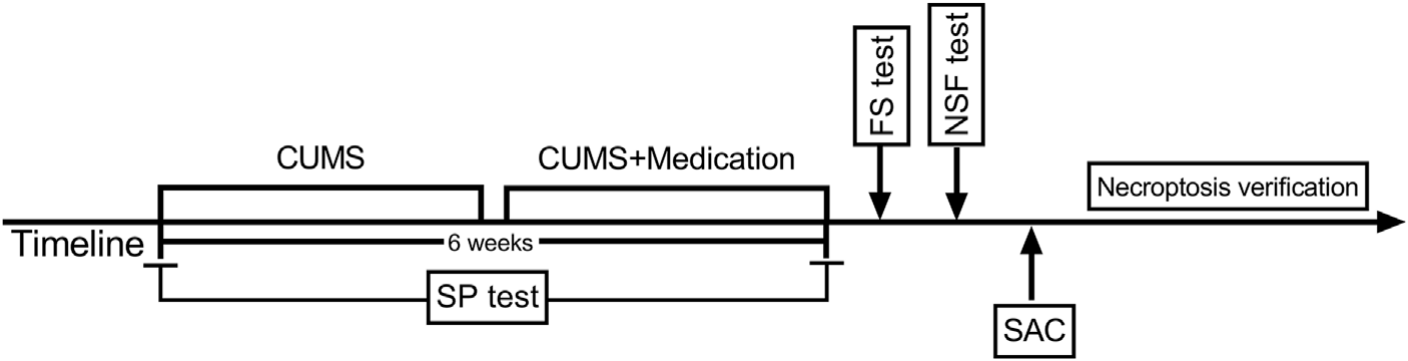
Schedule of the experimental design.

A total of 75 mice were randomly assigned to five groups (n = 15), including control group (no stress + physiological saline), CUMS group (CUMS + physiological saline), solvent group [CUMS + dimethyl sulphoxide (DMSO)/phosphate buffered saline (PBS)], Nec-1 group (CUMS + Nec-1), and Xiaoyaosan treatment group (CUMS + Xiaoyaosan). The CUMS modeling was performed as previously reported (Yan et al., 2019). Briefly, animals except the control group mice were subjected to the following stressors for six consecutive weeks: food deprivation for 24 h; water deprivation for 24 h; empty cages for 11 h; crowded cages for 24 h; restraint stress for 3 h; exposed to wet and soiled cages for 24 h; and 4°C cold water swimming for 5 min. The schedule of the study was showed in Figure 2.

Drug intervention lasted for 3 weeks, starting from the 4th week of modeling. The dosage of Xiaoyaosan and the duration of administration were confirmed according to the previous publications (Ding et al., 2017; Liu Y. et al., 2019). The Xiaoyaosan dry powder was dissolved in distilled water, then the mice in Xiaoyaosan treatment group received Xiaoyaosan by intragastric administration (0.254 g/kg/d), meanwhile mice in the CUMS group received 0.5 ml physiological saline (Yan et al., 2018). The concentration and volume of the gavage administration were adjusted once a week according to the changes in the body weight of the mice. Nec-1 (product number: RH82010-10mg, CAS number: 4311-88-0, BIORuler, Danbury, CT, United States) was dissolved in DMSO/PBS (1 mg in 125 μL of DMSO diluted with 1,375 μL of 0.1 M PBS). The Nec-1 group were injected intraperitoneally (i.p.) with Nec-1 (10 mg/kg) daily for 3 weeks, and the equivalent volume of DMSO/PBS (8.3%) was injected into solvent group mice (Dara et al., 2016).

The SP test was performed every week during the modeling process. The scheme lasted for successive 6 weeks, and the 3-weeks’s daily medication of Xiaoyaosan or Nec-1 was conducted. After the modeling, the FS test, and NSF test were carried out, then the mice were sacrificed for further detection.

### Behavioral Assessment

To evaluate the depressive behaviors of model animals, the sucrose preference (SP) test, forced swimming (FS) test and novelty-suppressed feeding (NSF) test were conducted as previous described with minor modifications (Barfield et al., 2013; Liao et al., 2018).

The SP test was performed every week during the modeling process. In brief, each mouse in this study was given two bottles filled with deionized water and 1% sucrose solution after 24-h fasting and water deprivation, then the consumption of sucrose solution and deionized water in 1 h was recorded, respectively. The sucrose preference rate was presented as the percentage of the 1% sucrose solution consumed out of the total amount of liquid consumed, which represented the parameter of hedonic behavior.

The FS test and NSF test were conducted after the 6-weeks modeling to further assess the depressed behaviors in mice. In the FS test, each mouse was placed in a clear glass aquarium (24 cm height and 19 cm diameter) containing approximately 6 cm of water (24 ± 1°C) for 5 min, and the whole time was recorded using a high definition camera. The mouse was accepted and considered to be immobile when it stopped swimming and kept afloat on the water which reflected the despair of animals after they failed to cope with the attempt, the immobility time was recorded in our study. The NSF test was performed in a white plastic box (25 × 25 × 20 cm) with a single food pellet of regular chow placed in the center. After 24 h of food deprivation, the mouse was placed in a corner of the box, the latency to chew the food pellet was recorded, followed by the measurement of the food consumption in the subsequent 5 min.

### Preparation of Tissue Samples

After 6 weeks of modeling, mice sacrificed by decapitation after behavioral tests. The hippocampal tissues of five mice in each group were collected for western blot analysis, the RNA preservation solution (Biotech, #2714) was used to preserve the hippocampi of five mice in each group for quantitative RT-PCR assay. The whole brains of the remaining mice in each group were fixed in 4% paraformaldehyde (PFA) solution (4% PFA, 2.5% glutaraldehyde, and 0.1 M PBS) and prepared for tissue slicing.

### Quantitative RT-PCR Analysis

Total RNA of hippocampus was extracted using Trizol^®^ reagent (Applied Biosystems, Waltham, MA, United States), then the quality and concentration of total RNA were determined by 1% agarose gel electrophoresis and Q3000 micro-volume spectrophotometer (Quawell Technology, San Jose, CA, United States). The RevertAid First Strand cDNA Synthesis Kit (Termo Fisher Scientifc, Waltham, MA, United States) was used to transfer total RNA into cDNA. Table 1 showed the sequences for primers used in our study which were designed based on published mRNA sequences in NCBI and synthesized by Sangon Biotech Co., Ltd, Shanghai, China. HPRT1 (BBI LifeScience, Amherst, MA, United States) was selected as the housekeeping gene in the experiment through reference genes selection method by geNorm, NormFinder, and BestKeeper software (Dongliang et al., 2011). The quantitative RT-PCR (qRT-PCR) reaction system was prepared by SYBR ^®^ Green PCR Master Mix (Applied Biosystems) in a final volume of 25 μL, then performed on Multicolor Real-time PCR Detection System (Bio-Rad, Hercules, CA, United States) with the following thermal cycling conditions: preincubation at 94 °C for 3 min, followed by 40 cycles of denaturation at 94°C for 30 s, annealing at 55°C for 30 s, and extending at 72°C for 30 s. The signals were normalized to HPRT1 and the relative expression of mRNA in each sample was calculated by 2^−∆∆Ct^ method.

**TABLE 1:** Primer sequences used in the quantitative RT-PCR analysis.

Gene	—	Sequences
IBA1	Forward	5′-TGG​CTC​CGA​GGA​GAC​GTT​CAG-3′
	Reverse	5′-GGA​CCA​GTT​GGC​CTC​TTG​TGT​TC-3′
IL-1β	Forward	5′-TCG​CAG​CAG​CAC​ATC​AAC​AAG​AG-3′
	Reverse	5′-TGC​TCA​TGT​CCT​CAT​CCT​GGA​AGG-3′
Lipocalin-2	Forward	5′-CGC​TAC​TGG​ATC​AGA​ACA​TTT​G-3′
	Reverse	5′-CTT​GCA​CAT​TGT​AGC​TCT​GTA​C-3′

### Western Blot Analysis

Protein levels of IBA1, IL-1β, Lipocalin-2, RIPK1, RIPK3, MLKL, and *p*-MLKL were measured by western blot analysis. The total protein of hippocampal tissues was extracted using RIPA Lysis buffer (Biomiga, Santiago, CA, United States). The proteins were loaded onto 12% SDS-PAGE gels at 30 µg per lane, and then transferred onto polyvinylidene fluoride (PVDF) membranes. The 5% non-fat dry milk in 0.05% tris buffered saline tween (TBST) was used to block the membranes, and the membranes were incubated with primary antibodies at 4 °C overnight (IBA1, 1:1,000 dilution, ab178847, Abcam, San Francisco, CA, United States; IL-1β, ab9722, 1:1,000 dilution, Abcam; Lipocalin-2, ab216462, 1:100 dilution, Abcam; RIPK1, 1:1,000 dilution, ab72139, Abcam; RIPK3, 1:1,000 dilution, ab56164, Abcam; phosphorylated MLKL (*p*-MLKL), 1:1,000 dilution, ab196436, Abcam; MLKL, 1:2000 dilution, orb32399, Biorbyt, Cambridge, UK; β-actin, 1:5,000 dilution, #5125, Cell Signal, Danvers, MA, United States). Then, the membranes were incubated with horseradish peroxidase- (HRP-) conjugated secondary antibody [Goat Anti-Rabbit IgG H&L (HRP), 1:10,000 dilution, ab205718, Abcam; Goat Anti-Mouse IgG H&L (HRP), 1:10,000 dilution, ab205719, Abcam] for 1 h at room temperature. The enhanced chemiluminescence (ECL) detection reagent (Thermo Fisher Scientific) was used to develop the membranes for 1 min, and the Tanon-5200 system (Tanon, Shanghai, China) was used to visualize the protein bands. The optical density of protein bands was quantified by Tanon Gis software (Tanon).

### Histopathological Examination, Immunohistochemistry and Immunofluorescence

The brain samples fixed in 4% PFA solution were used to prepared paraffin-embedded tissue sections which included hippocampal CA1 and CA3 regions based on the stereotaxic atlases of the mouse brain (Konsman, 2003). The sections were stained with hematoxylin-eosin (H&E) to observe the cellular morphology and infiltration of inflammatory cells. A four-point severity scale (0, normal; 1, mild; 2, moderate; 3, severe) was used to score the H&E stained sections by an experienced pathologist in a blinded manner.

Immunohistochemical (IHC) staining was used to observe the levels of IBA1 and *p*-MLKL in hippocampus followed the previous described procedures (Yan et al., 2018). After dewaxing, dehydration and antigen retrieval, the brain sections were incubated with 3% H_2_O_2_ solution at room temperature for 10 min. Next, sections were incubated with primary antibodies (IBA1, 1:8,000 dilution, ab178847, Abcam; *p*-MLKL, 1:250 dilution, ab196436, Abcam) overnight at 4 °C. Then sections were incubated with secondary antibodies, and the diaminobezidin 3, 3 (DAB) solution (Invitrogen, Carlsbad, CA, United States) was used for coloration. For quantitative analysis, the original immunohistochemical images were collected with an image analyzer (MIAS99, Fubo-Tech Co., Beijing, China) with a color video camera (TK-C1381, JVC, Beijing, China) and BX50 microscope (Olympus Co., Tokyo, Japan), and the positive staining of hippocampal CA1 and CA3 regions at high power magnification (×400) was assessed by mean optical density (MOD) using Image-pro Plus software (version 6.0, Rockville, MD, United States).

Immunofluorescence (IF) staining was used to observe the expression of *p*-MLKL as previous described (Qu et al., 2017). The brain sections were incubated with 0.5% TritonX-100 solution at room temperature for 10 min. Next, sections were incubated with primary antibody (*p*-MLKL, 1:100 dilution, ab196436, Abcam) overnight at 4°C. Then sections were incubated with fluorescent-conjugated goat anti-rabbit IgG, follow by the Thioflavin T and DAPI staining. The fluorescence signal of sections was observed and photographed under an Olympus BX53 fluorescence microscope (Olympus Co., Tokyo, Japan), and the mean fluorescence intensity (MFI) was analyzed using Image-pro Plus software.

### Statistical Analysis

In this study, all data were analyzed using SPSS software (version 20.0, Chicago, IL, United States) and expressed as means ± SEM. The statistical analyses were performed by one-way analysis of variance (ANOVA) with least significant difference (LSD) post hoc multiple comparisons when equal variances were assumed, Dunnett’s T3 test was used when the data had a normal distribution but the variances are not homogeneous. A *p*-value of 0.05 was considered statistically significant.

## Results

### Active Ingredients of Xiaoyaosan Regulated Necroptosis in Depression

The network pharmacology method was used to initial investigate whether the active components of Xiaoyaosan could improve necroptosis pathway in depression.

**FIGURE 3:**
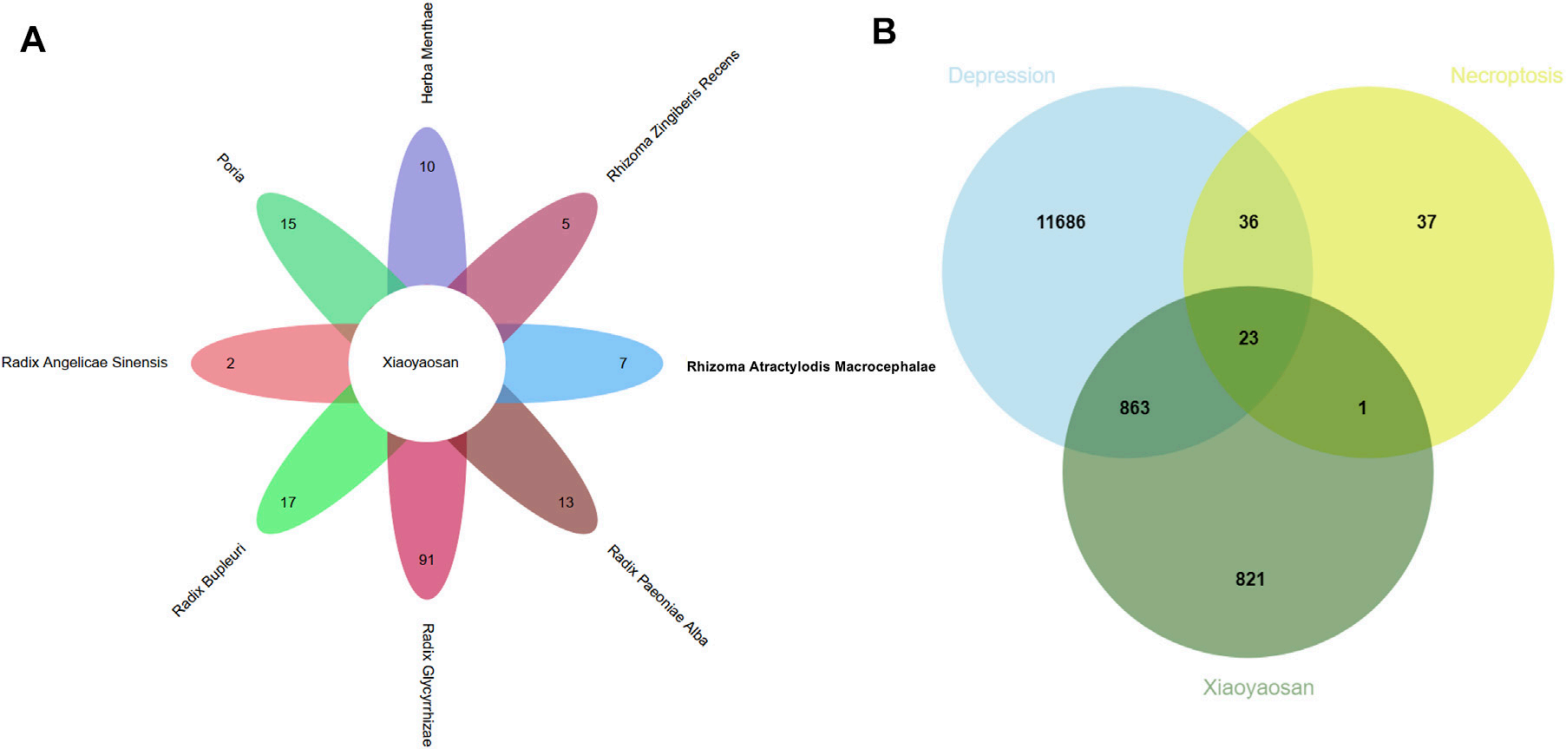
Wynn maps for the active ingredients of Xiaoyaosan and potential treatment targets **(A)** Based on the ADME parameters (OB ≥ 30% and DL ≥ 0.18), there were 17 ingredients in *Radix Bupleuri*, two ingredients in *Radix Angelicae Sinensis*, 13 ingredients in *Radix Paeoniae Alba*, 15 ingredients in *Poria*, seven ingredients in *Rhizoma Atractylodis Macrocephalae*, 91 ingredients in *Radix Glycyrrhizae*, five ingredients in *Rhizoma Zingiberis Recens* and 10 ingredients in *Herba Menthae* **(B)** A total of 1708 predicted targets of TCM formula Xiaoyaosan, 12,608 therapeutic targets for depression and 97 necroptosis related targets were intersected, and there were 23 potential treatment target genes.

As showed in Figure 3, a total of 148 active ingredients in the eight botanical drugs of Xiaoyaosan were collected based on the ADME parameters (OB ≥ 30% and DL ≥ 0.18), and we obtained 23 potential treatment targets (BAX, BCL2, CAPN1, CAPN2, CASP1, CASP8, EIF2AK2, IL1B, JAK1, JAK2, JAK3, MLKL, NLRP3, PARP1, PPIA, RIPK1, RIPK3, STAT1, STAT3, STAT6, TLR4, TNF, XIAP) that might be associated with the antidepressant mechanism of Xiaoyaosan through necroptosis pathway by taking the intersection of 1708 putative targets of the active ingredients in Xiaoyaosan, 12,608 therapeutic targets for depression and 97 necroptosis related targets.

**FIGURE 4:**
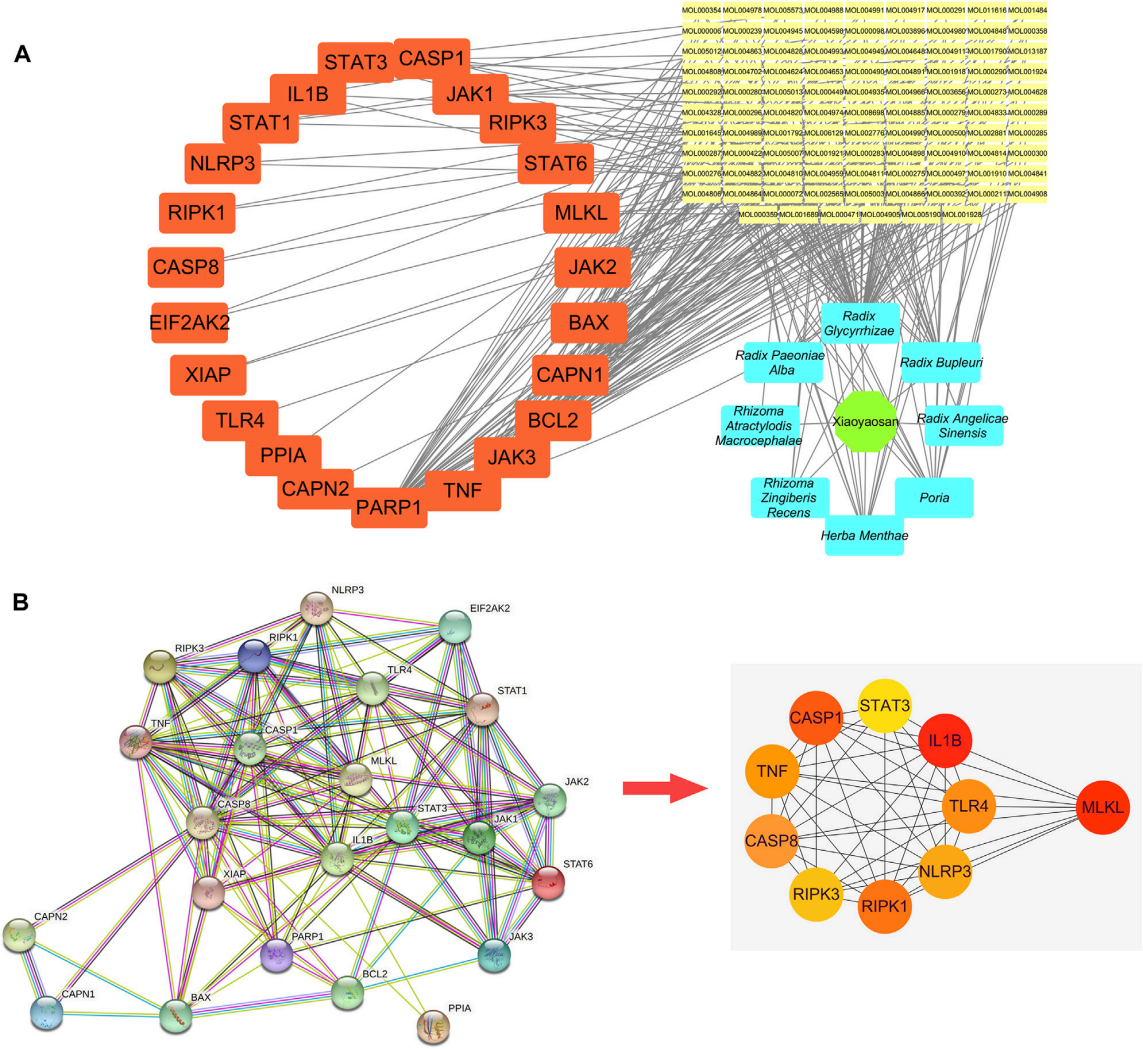
“Botanical drugs-active ingredients-potential targets” network and PPI network of potential target genes of Xiaoyaosan **(A)** The “Botanical drugs-active ingredients-potential targets” network: These eight botanical drugs of TCM formula Xiaoyaosan had 96 active ingredients which could regulate the 23 key target genes according to the network pharmacological analysis **(B)** PPI network of the 23 key target genes: The sub-network of the top 10 core targets was picked up from the PPI network of potential target genes of Xiaoyaosan using the cytoHubba plugin in Cytoscape software.

Figure 4A showed the “botanical drugs-active ingredients-potential targets” network, eight botanical drugs of TCM formula Xiaoyaosan had 96 active ingredients which could regulate the 23 key target genes. Therefore, based on the network pharmacological analysis we can speculate that traditional Chinese formula Xiaoyaosan could regulate the necroptosis pathway in depression. Figure 4B showed the PPI network of potential target genes of Xiaoyaosan and the sub-network of the top 10 core targets which was picked up using the cytoHubba plugin in Cytoscape software. As showed in Table 2, the hub nodes of sub-network were ranked by maximal clique centrality (MCC) method, and MLKL, IL1B, CASP8, CASP1, RIPK1, TLR4, NLRP3, RIPK3, TNF, STAT3 were the top 10 potential treatment targets of Xiaoyaosan.

**TABLE 2:** The Top 10 core target genes in PPI network ranked by MCC method.

Rank	Name	Score
1	MLKL	147,962
2	IL1B	142,802
3	CASP8	137,046
4	CASP1	131,880
5	RIPK1	131,040
6	TLR4	92,280
7	NLRP3	85,680
8	RIPK3	80,640
8	TNF	80,640
10	STAT3	67,446

### Depressive-like Behaviors of CUMS Mice

**FIGURE 5:**
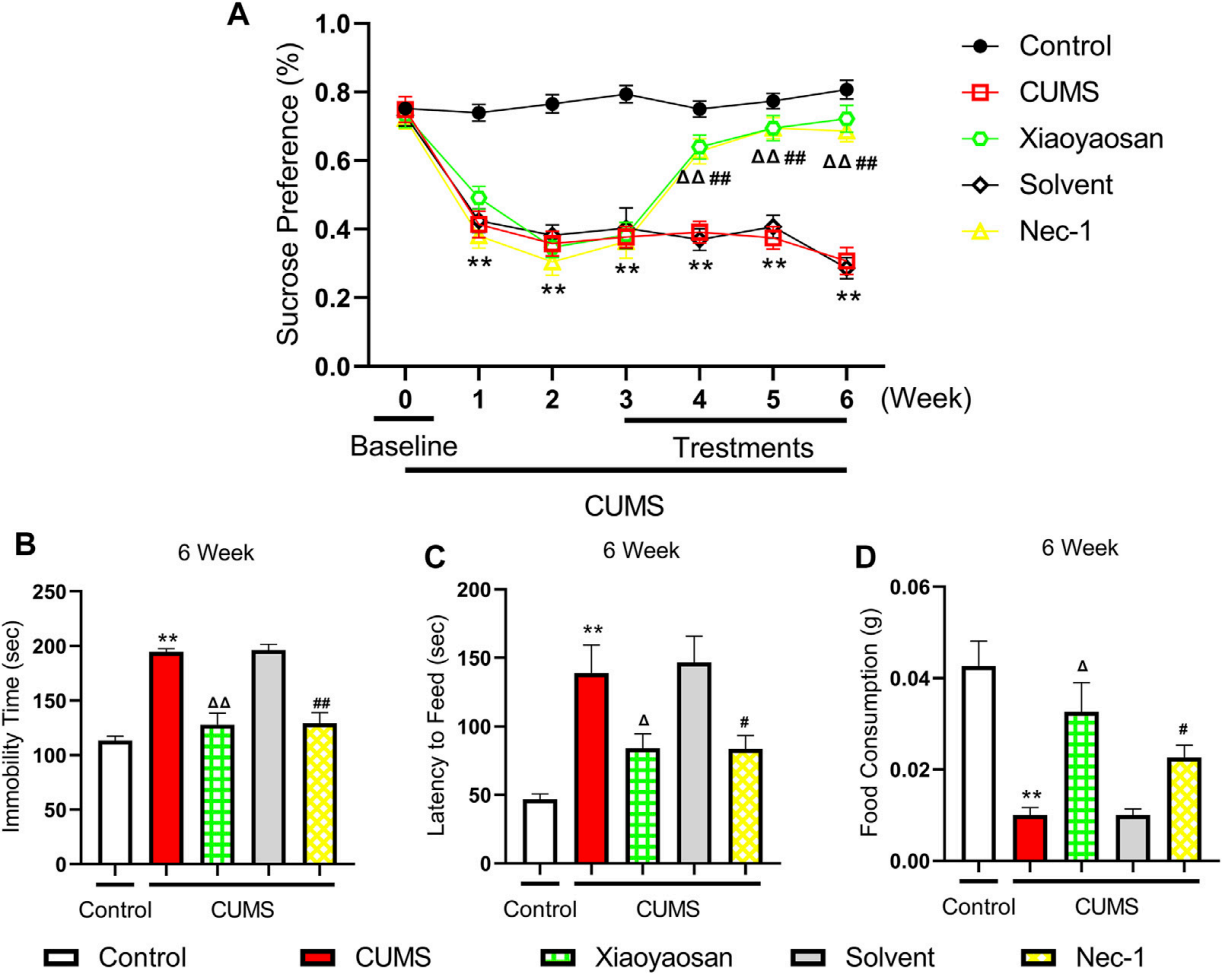
Changes in the Depressive-like behaviors of CUMS mice **(A)** Changes of sucrose preference rate during the CUMS modeling period (n = 15) **(B)** FS test results after the 6-weeks modeling (n = 15) **(C)** The results of latency to feed in NSF test after the 6-weeks modeling (n = 15) **(D)** The results of food consumption in NSF test after the 6-weeks modeling (n = 15). Data were expressed as means ± SEM, **p < 0.01 versus control group; Δ *p* < 0.05, ΔΔ *p* < 0.01 versus CUMS group; #*p* < 0.05, ##*p* < 0.01 versus solvent group.

We performed the sucrose preference (SP) test, forced swimming (FS) test and novelty-suppressed feeding (NSF) test to observe the effects of Xiaoyaosan on depressive-like behaviors. As shown in Figure 5A, the baseline sucrose preference rate of control group, CUMS group, solvent group, Nec-1 group and Xiaoyaosan group had no significant differences. However, after CUMS modeling, the mice in CUMS group had a significant drop in sucrose preference rate compared with the control group mice (*p* < 0.01), while the Nec-1 group and Xiaoyaosan treatment group had no differences with the stress groups. Then, the CUMS progress continued for another 3 weeks with medication procedure, the mice in CUMS group and solvent group still had a low sucrose preference rate (*p* < 0.01), while the sucrose preference rate of Nec-1 group and Xiaoyaosan treatment group mice was significant increased (both *p* < 0.01). The CUMS group and solvent group mice exhibited significantly longer immobility time in the FS test (*p* < 0.01), and Xiaoyaosan or Nec-1 ameliorated this depressive-like behavior in comparison with the CUMS group and solvent group (both *p* < 0.01) (Figure 5B). For the NSF test, it was shown that mice in CUMS group had a significant longer latency to chew the food pellet compared with the control mice (*p* < 0.01) (Figure 5C), and the food consumption significantly decreased (*p* < 0.01) (Figure 5D). There was no significant difference between the solvent group and the CUMS group. Xiaoyaosan or Nec-1 could reverse these changes in the NSF test (both *p* < 0.05).

### Necroptosis in the Hippocampus of CUMS Mice

**FIGURE 6:**
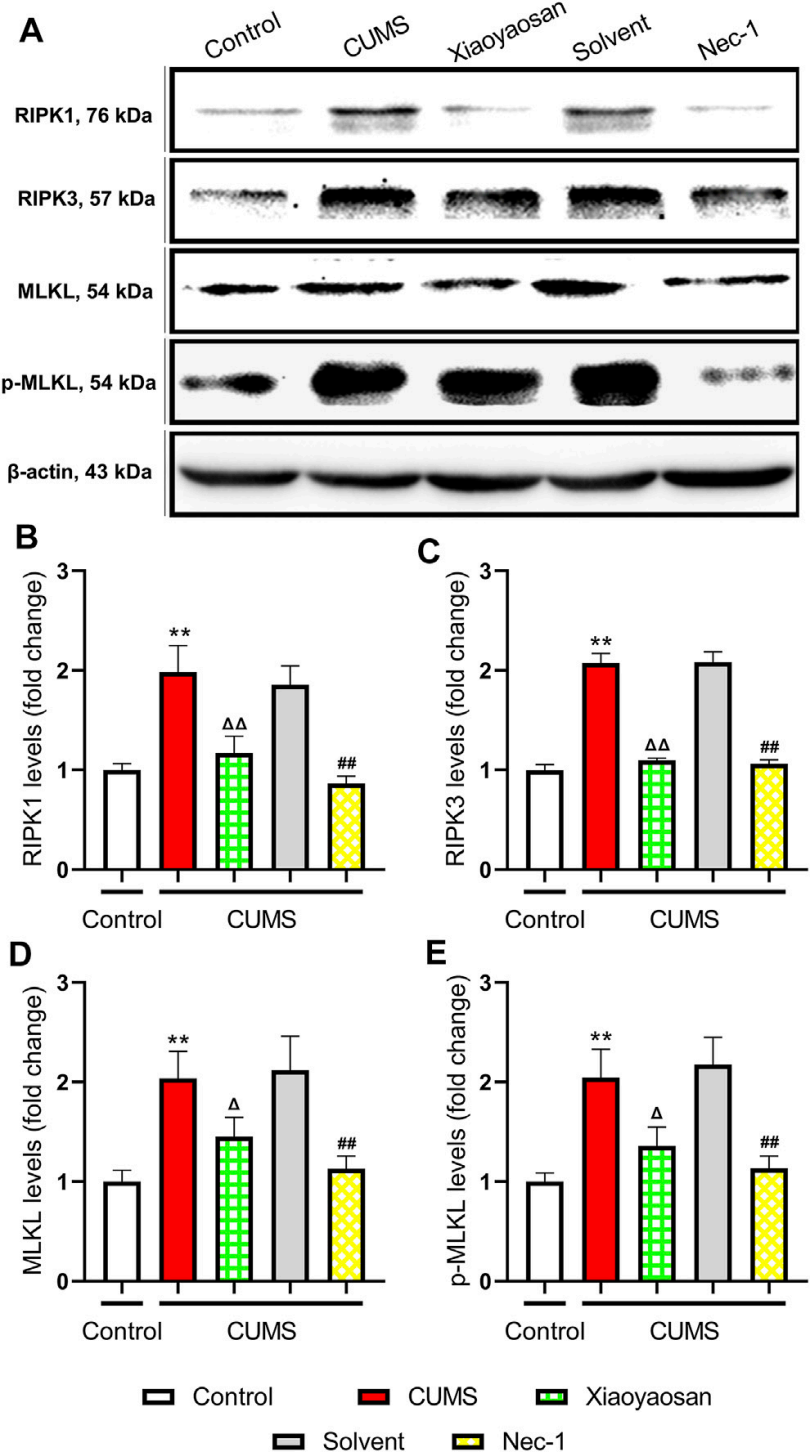
Changes of necroptosis biomarkers in the hippocampi of CUMS mice **(A)** Representative micrographs of WB for necroptosis biomarkers **(B)** The expression of RIPK1 in the hippocampus of experimental mice (n = 5) **(C)** The expression of RIPK3 in the hippocampus of experimental mice (n = 5) **(D)** The expression of MLKL in the hippocampus of experimental mice (n = 5) **(E)** The expression of *p*-MLKL in the hippocampus of experimental mice (n = 5). Data were expressed as means ± SEM, **p < 0.01 versus control group; Δ *p* < 0.05, ΔΔ *p* < 0.01 versus CUMS group; ##*p* < 0.01 versus solvent group.

In order to explore the possible mechanism of necroptosis in the mouse model of depression, the expressions of RIPK1, RIPK3, MLKL, *p*-MLKL were detected by western blot assay. As shown in Figure 6, the levels of RIPK1, RIPK3, MLKL and *p*-MLKL significant up-regulated in the hippocampi of CUMS-exposed mice compared to the control group (*p* < 0.01). Compared with the solvent group, these necroptosis biomarkers in Nec-1 group were significant down-regulated (both *p* < 0.01). Also, the effects of Xiaoyaosan were similar to Nec-1, which could mitigate the stress-induced elevation of RIPK1, RIPK3, MLKL and *p*-MLKL levels, as shown in the comparison between Xiaoyaosan treatment group and CUMS group (*p* < 0.05 or *p* < 0.01).

**FIGURE 7:**
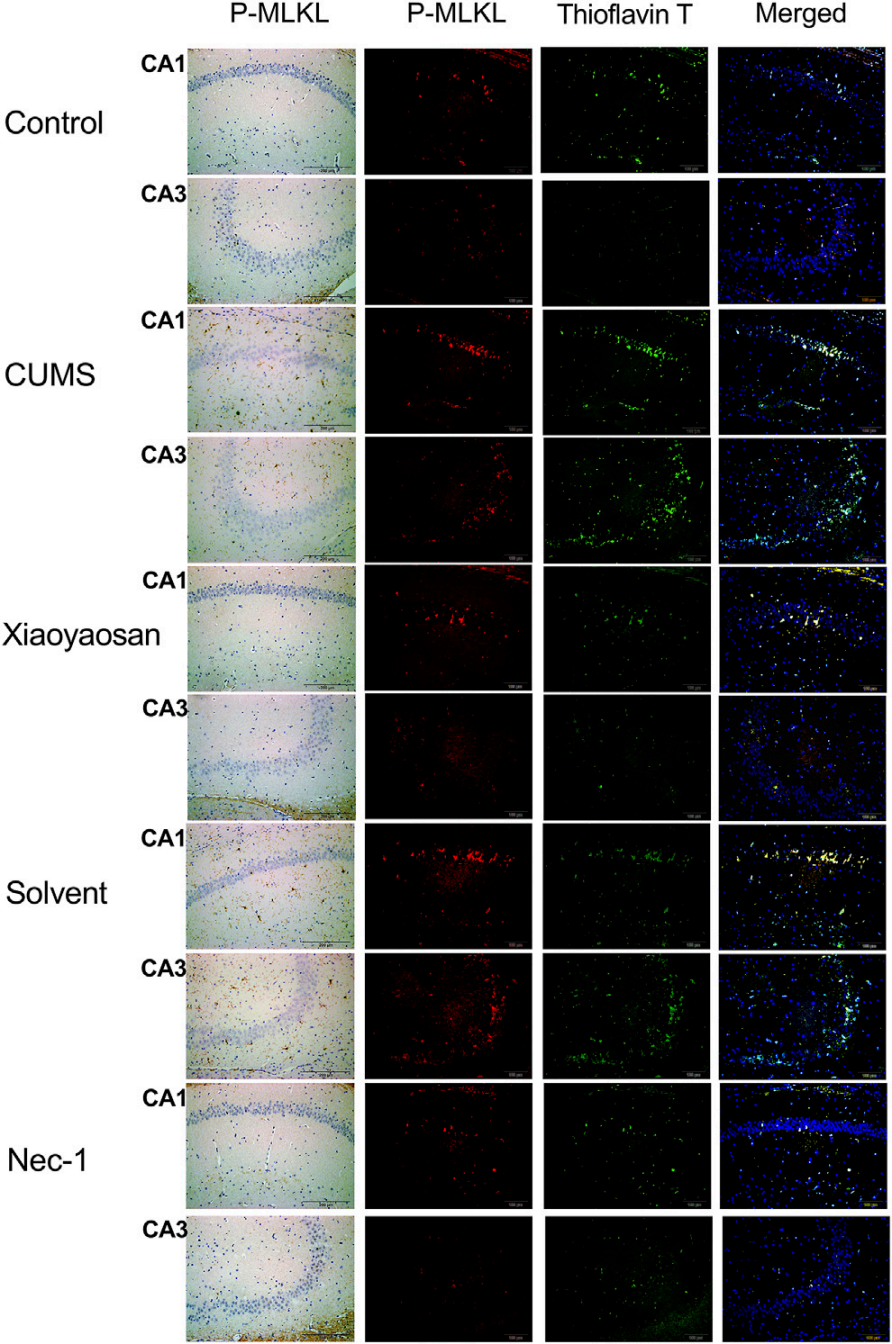
Representative micrographs of IHC and IF staining for *p*-MLKL (scale bar = 200 μm, ×400 magnification) in the CA1 and CA3 regions.

**FIGURE 8:**
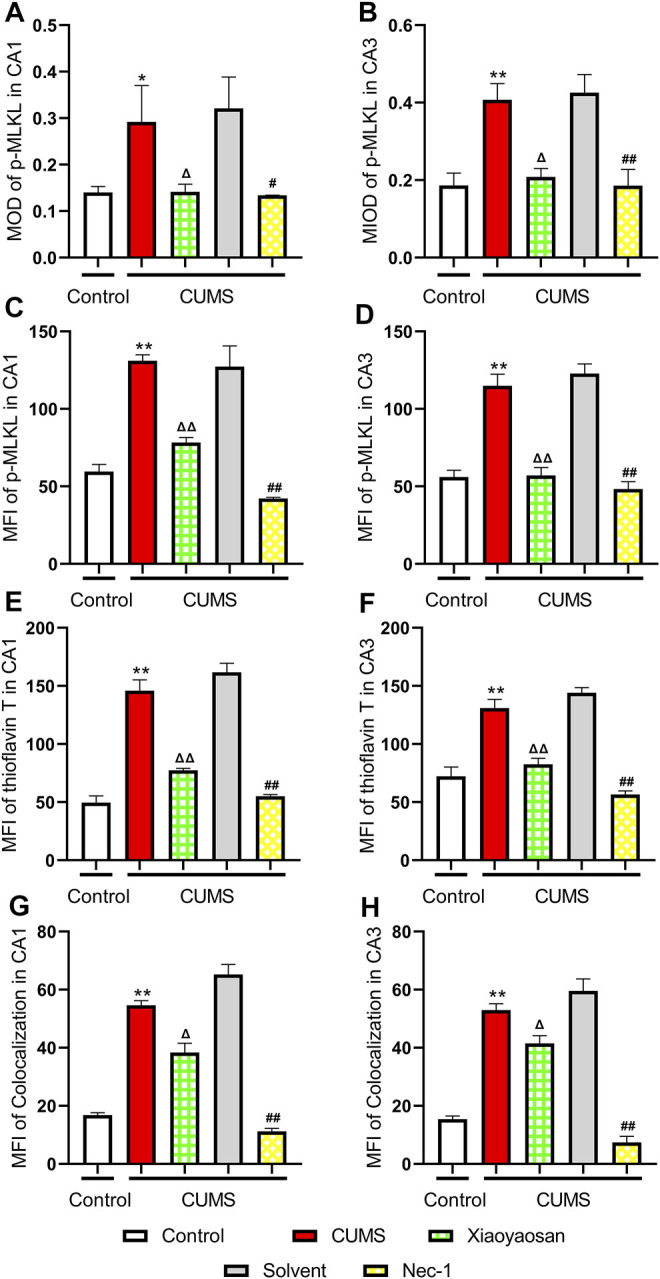
Expression of *p*-MLKL in CA1 and CA3 regions of the hippocampus **(A)** The MOD level of *p*-MLKL in the CA1 region (n = 5) **(B)** The MOD level of *p*-MLKL in the CA3 region (n = 5) **(C)** The MFI level of *p*-MLKL in the CA1 region (n = 5) **(D)** The MFI level of *p*-MLKL in the CA3 region (n = 5) **(E)** The MFI level of thioflavine T staining in the CA1 region (n = 5) **(F)** The MFI level of thioflavine T staining in the CA3 region (n = 5) **(G)** The MFI level of *p*-MLKL colocalized with thioflavin T staining in the CA1 region (n = 5) **(H)** The MFI level of *p*-MLKL colocalized with thioflavin T staining in the CA3 region (n = 5). Data were expressed as means ± SEM, *p < 0.05, **p < 0.01 versus control group; Δ *p* < 0.05, ΔΔ *p* < 0.01 versus CUMS group; #*p* < 0.05, ##*p* < 0.01 versus solvent group.

To further verified the process of necroptosis in CUMS-induced mice, the IHC and IF staining methods were used to observe the distribution of *p*-MLKL in hippocampus. Figure 7 exhibited the representative micrographs of IHC and IF staining for *p*-MLKL in the CA1 and CA3 regions of hippocampus, and the results were set out in Figure 8. The MOD of *p*-MLKL in both CA1 and CA3 regions of CUMS group mice was significantly increased when compared with the control group (*p* < 0.05, *p* < 0.01, respectively), and there was no significant difference between solvent group and CUMS group. Then the treatment of Nec-1 or Xiaoyaosan reversed the CUMS-induced *p*-MLKL changes in the CA1 and CA3 (*p* < 0.05 or *p* < 0.01, Figures 8A,B). Compared with the control group, the MFI of *p*-MLKL of CUMS group mice was significantly increased, and CUMS group had no significant difference with solvent group. Similarly to IHC staining results, the *p*-MLKL MFI was decreased in Nec-1 and Xiaoyaosan group mice (*p* < 0.05, *p* < 0.01, Figures 8C,D). The outcomes of thioflavine T staining showed that the MFI of thioflavine T labelled amyloid-like polymers was also increased in the CA1 and CA3 regions of CUMS group mice compared to the control group (both *p* < 0.01, Figures 8E,F). In the end, the IF staining results revealed that high levels of the *p*-MLKL colocalized with thioflavin T staining was detected in the CA1 and CA3 regions of CUMS group mice, in contrast with the low colocalization in control group (*p* < 0.01, Figures 8G,H). The low levels of colocalization were observed in the CA1 and CA3 regions of mice in Xiaoyaosan group and Nec-1 group as well (*p* < 0.05, *p* < 0.01).

### Hippocampal Neuroinflammation of CUMS Mice

**FIGURE 9:**
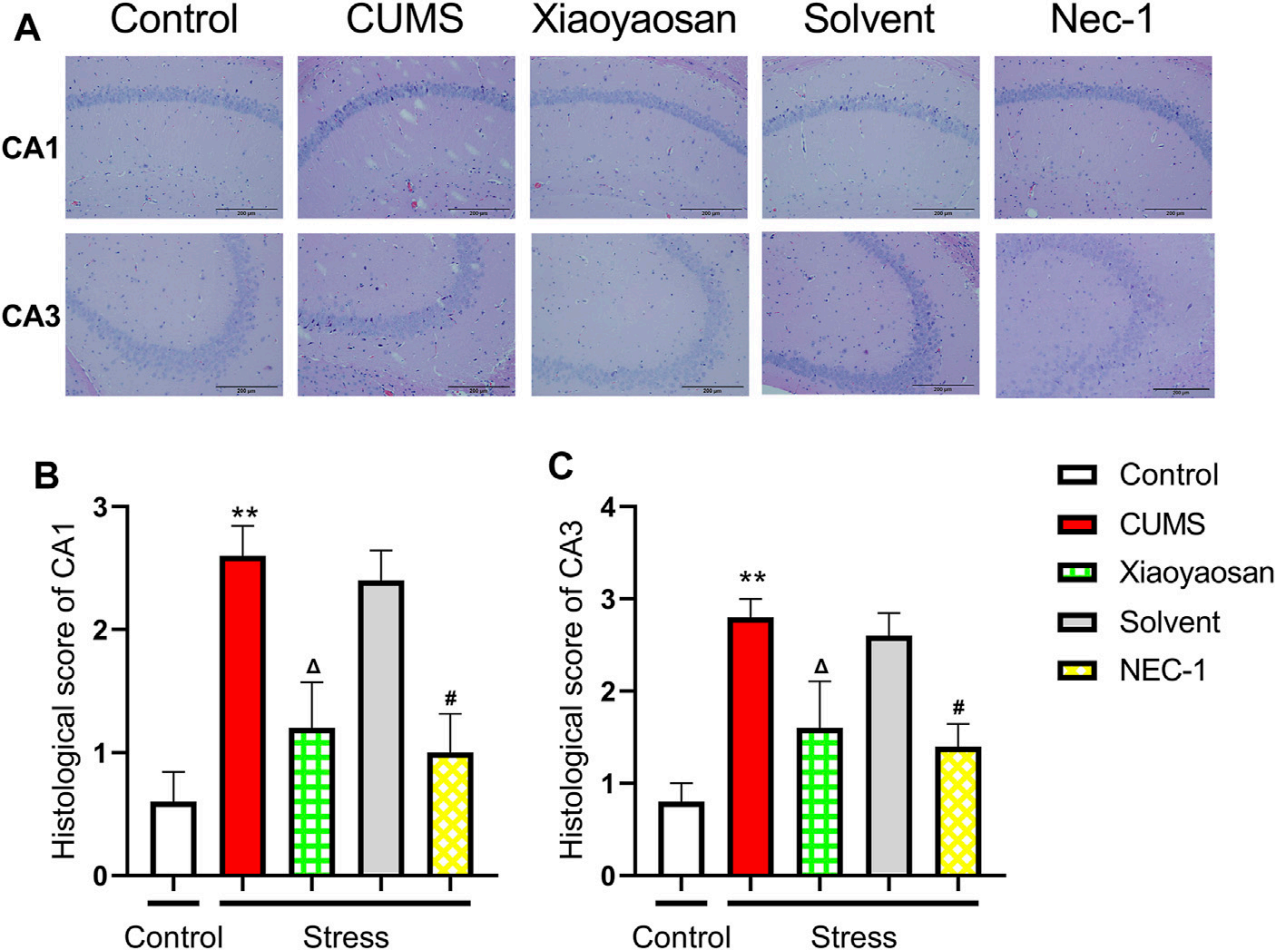
The histological changes in CA1 and CA3 regions of the hippocampi in CUMS mice **(A)** Representative micrographs of H&E staining (scale bar = 200 μm, ×400 magnification) in the CA1 and CA3 regions **(B)** The histological scores in the CA1 region (n = 5) **(C)** The histological scores in the CA1 region (n = 5). Data were expressed as means ± SEM, **p < 0.01 versus control group; Δ *p* < 0.05 versus CUMS group; #*p* < 0.05 versus Solvent group.

**FIGURE 10:**
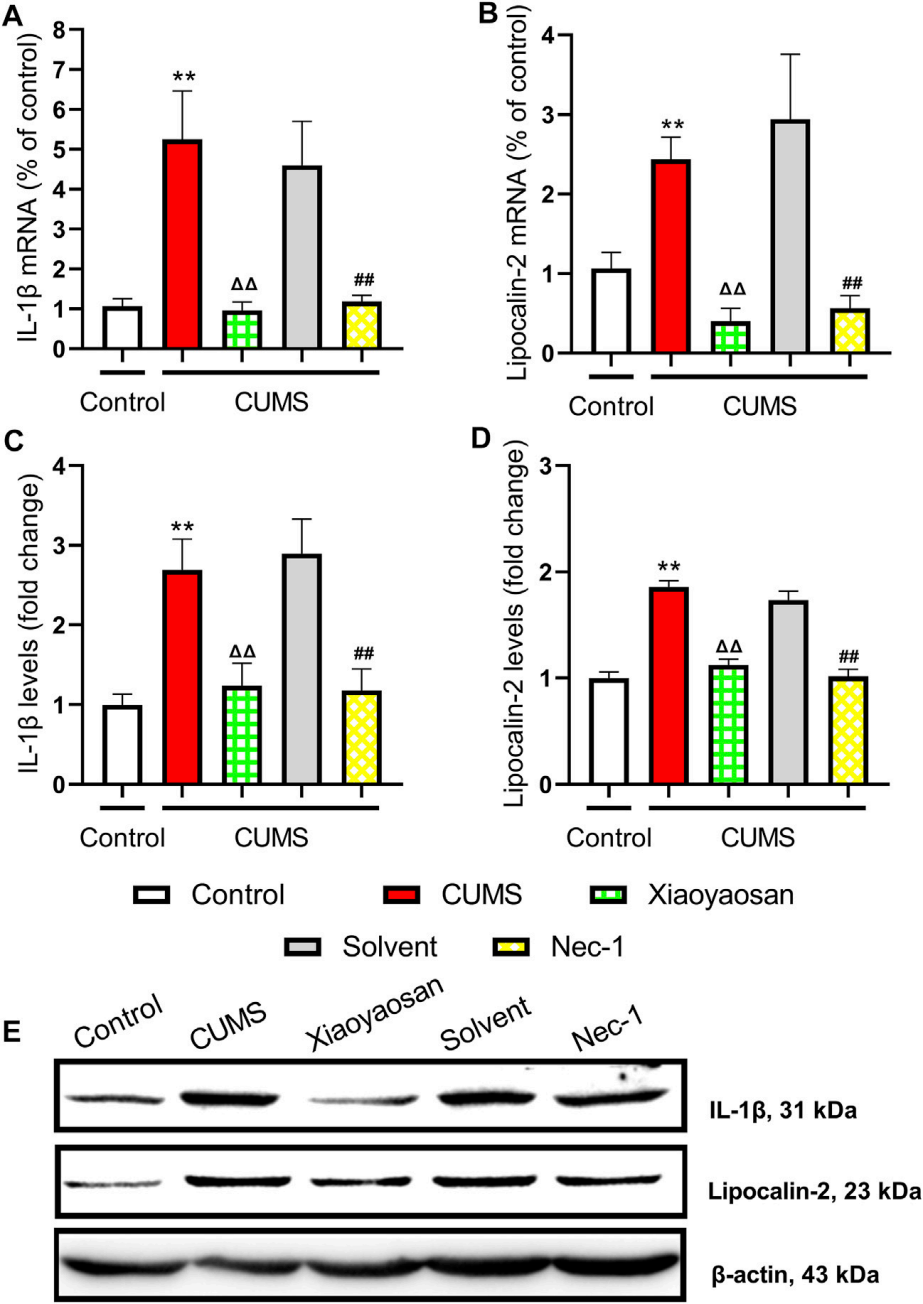
The levels of IL-1β and Lipocalin-2 in the hippocampus of CUMS mice **(A)** The mRNA results of IL-1β (n = 5) **(B)** The mRNA results of Lipocalin-2 (n = 5) **(C)** The protein results of IL-1β (n = 5) **(D)** The protein results of Lipocalin-2 (n = 5) **(E)** Representative micrographs of WB for IL-1β and Lipocalin-2. Data were expressed as means ± SEM, **p < 0.01 versus control group; ΔΔ *p* < 0.01 versus CUMS group; ##*p* < 0.01 versus Solvent group.

The H&E staining was performed in order to evaluate the inflammatory infiltrates in mice hippocampal CA1 and CA3 regions. As exhibited in Figure 9A, a large number of inflammatory cell infiltration was observed in the hippocampus of the mice in chronically stressed groups treated with vehicle (physiological saline and solvent group). The histological scores of the hippocampus in each group were judged, it was found that the scores of CUMS group and solvent group in CA1 and CA3 regions were significantly higher than that of control group (both *p* < 0.01), Xiaoyaosan or Nec-1 treatment notably mitigated CUMS-induced inflammatory infiltrates (both *p* < 0.05) (Figures 9B,C). The expression levels of IL-1β and Lipocalin-2 were measured by qRT-PCR and western blot. The results indicated that the 6-weeks CUMS significant up-regulated the mRNA and protein levels of IL-1β in the hippocampus of CUMS-exposed mice (both *p* < 0.01), and the mice in the Xiaoyaosan treatment group and Nec-1 group showed a significant increase in IL-1β expression compared with the CUMS group mice (both *p* < 0.01) (Figures 10A,C). The CUMS modeling also increased the Lipocalin-2 level (*p* < 0.01), whereas the administration of Xiaoyaosan and Nec-1 noticeably inhibited the expressions of Lipocalin-2 (both *p* < 0.01) (Figures 10B,D).

### Hippocampal Microglial Activation of CUMS Mice

**FIGURE 11:**
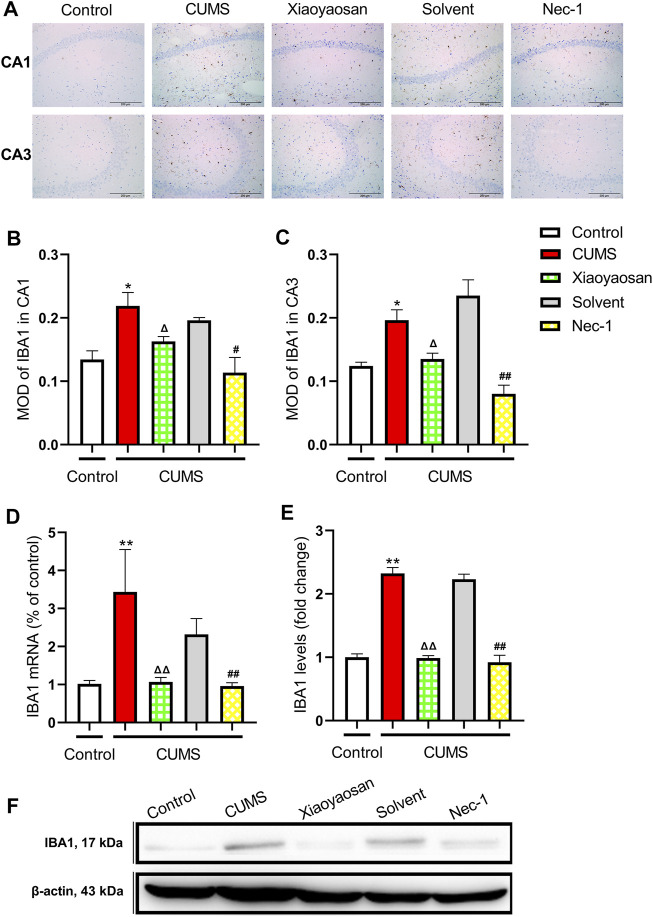
Expression of IBA1 in CA1 and CA3 regions of the hippocampi in CUMS mice **(A)** Representative micrographs of immunohistochemical staining for IBA1 (scale bar = 200 μm, ×400 magnification) in the CA1 and CA3 regions **(B)** The MOD level of IBA1 in the CA1 region (n = 5) **(C)** The MOD level of IBA1 in the CA3 region (n = 5) **(D)** The mRNA results of IBA1 (n = 5) **(E)** The protein results of IBA1 (n = 5). Data were expressed as means ± SEM, *p < 0.05, **p < 0.01 versus control group; Δ *p* < 0.05, ΔΔ *p* < 0.01 versus CUMS group; #*p* < 0.05, ##*p* < 0.01 versus Solvent group.

To initial test the level of microglial activation in the hippocampi of CUMS-exposed mice, the expression of IBA1 was detected. Figure 11A presented the representative micrographs of IHC staining for IBA1 in the CA1 and CA3 regions of hippocampus. As showed in Figures 11B,C, the MOD of IBA1 in CA1 and CA3 of CUMS mice was significantly increased when compared to the control group (both *p* < 0.05), whereas the expression of IBA1 in CA1 and CA3 was noticeably reduced by the interference of Nec-1 and Xiaoyaosan (*p* < 0.05, *p* < 0.01). As showed in Figures 11D,E, the qRT-PCR and western blot data also demonstrated the high level of IBA1 in the hippocampus of the chronically stressed mice (both *p* < 0.01), and the treatment with Nec-1 or Xiaoyaosan significantly reduced the expression of IBA1 as compared to stressed groups (both *p* < 0.01).

## Discussion

Our study proposed that necroptosis was involved in the antidepressant mechanism of Xiaoyaosan. A total of 23 potential treatment targets were screened out through network pharmacological analysis that might be related to the active ingredients of Xiaoyaosan intervened necroptosis pathway in depression. In the meantime, the animal experiment revealed that Xiaoyaosan displayed antidepressant-like effects via regulating the expression of RIPK1, RIPK3, MLKL, and the phosphorylation level of MLKL of depressed mice, and then improving the neuroinflammation and microglia function in the hippocampus.

Depression is a prevalent and persistent psychiatric illness affecting millions of people worldwide. Many hypotheses such as inflammatory cytokines, hypothalamic pituitary adrenal (HPA) axis, neurotransmitter system, brain-derived neurotrophic factor (BDNF) in the brain and endogenous metabolites are involved in depression pathogenesis, but it is still obscure (Peng et al., 2015). Today, there is still lack of precise and effective diagnostic or therapeutic methods for depression. Xiaoyaosan is a common TCM formula for treating syndrome of stagnation of liver qi and spleen deficiency with the characteristics of multiple ingredients, multiple targets and multiple pathways, and it also has a good therapeutic effect on depressive diseases (Chen et al., 2020). Although previous studies have revealed some antidepressant mechanisms of the active ingredients of Xiaoyaosan (Liu X. et al., 2021; Liu XJ. et al., 2021), while whether the components of Xiaoyaosan could improve necroptosis pathway in depression remains to be discussed. The present study preliminary disclosure that Xiaoyaosan could regulate the necroptosis pathway in depression via its 96 active ingredients that could regulate the 23 key target genes based on the network pharmacological analysis. Through PPI network analysis of these 23 target genes, we confirmed that MLKL, IL1B, CASP8, CASP1, RIPK1, TLR4, NLRP3, RIPK3, TNF, STAT3 as the potential target genes of Xiaoyaosan in the treatment of depression, among which the MCC score of MLKL was the highest. Mounting evidence suggested that necroptosis depended on RIPK1/RIPK3/MLKL activities, RIPK1 and RIPK3 upregulated upon activation of death receptors, triggered a signaling cascade involving phosphorylation of MLKL which contributes to the change of plasm membrane permeability, then resulting in cellular necrosis (Liu C. et al., 2021; Marunouchi et al., 2021; Yu et al., 2021). Therefore, the expressions of RIPK1, RIPK3, MLKL were evaluated as biomarkers of necroptosis in the study.

Stress was closely related to neurological disorders, and the sustained psychosocial stress is one of the main causes of depression. Chronic or repeated stress can cause neuronal disturbances, which was similar to changes observed in the brain during depression (de Kloet and Joëls, 2005). Animal model of depression was an important method to explore the dysregulatory mechanisms of depression and the regulatory mechanisms by which antidepressant treatments alleviate the various depressive symptoms (Frazer and Morilak, 2005). The depression model by CUMS method was widely recognized and applied for mechanism research and drug selection. Also, some studies showed that the depressive behaviors induced by CUMS was similar to the clinical manifestations of depression induced by long-term exposure to multiple stresses (D'Aquila et al., 1994; Papp et al., 1996). Here, we found that after 6 weeks of CUMS, mice were observed to exhibit significant depressive symptoms through behavioral evaluation, which was consistent with other studies (Cao et al., 2013). This study used classical evaluation methods to observe depressive behavior in CUMS-induced mice, including SP test, FS test and NSF test. The performance of anhedonia was manifested by lack of interest in reward stimulus, which was a manifestation of affective disorders including depression, while SP test was a method to evaluate the degree of pleasure loss in rodents (Weiss, 1997). FS test was a kind of negative stress experiment that animals could not escape from hostile environment and produce hopeless behaviors, the immobility time was used as an important index to evaluate depressive behaviors (Bächli et al., 2008). So, it was widely used in the exploration of the pathogenesis of stress and the pharmacological study of potential antidepressants. The NSF test observed the contradiction between the fear of new environment and the great need for things, the food consumption and latency of feed could represent the degree of depression and anxiety in animals (Shalom et al., 2006). The results showed that there were obvious behavioral changes in the CUMS model group, including decreased sucrose preference rate, prolonged immobility time, longer latency of feed and decreased appetite. Thus, it could be proved that the 6-weeks modeling successfully established depressive-like changes in mice. In the meantime, we found that the treatment of Xiaoyaosan could significantly improve the depressive-like behavioral changes in mice which was consistent with the results of previous studies, indicating that Xiaoyaosan exhibited good antidepressant performances (Wang et al., 2018; Liu X. et al., 2019).

The mechanism of cell death had always been one of the focuses of biomedical research, and it was common believed that cell death patterns included apoptosis, necrosis and autophagy at present. Apoptosis and autophagy required the synthesis of new proteins and energy supplies, which was an active process of cell self-regulation, also known as programmed death (M Stacey and Wei-Xing, 2006). Cell necrosis was previously considered to be a passive disordered process that occurred under overwhelming physical and chemical damage from the outside world, and therefore could not be regulated (Vandenabeele et al., 2008). With the in-depth study of the mechanism of cell death, it was found that cell necrosis was also a programmed death process with notable necrosis features, then it was redefined as necroptosis (Degterev et al., 2005). Necroptosis was mainly initiated by the members of tumor necrosis factor (TNF) receptor family and toll-like receptor (TLR) family, which phosphorylated MLKL by transmitting death signals through RIPK1 and RIPK3(Galluzzi and Kroemer, 2008). Thus, as the executor of cell death, phosphorylation of MLKL eventually led to cell necrosis. The necrotic cells released their contents as damage-associated molecular pattern molecules (DAMPs) to stimulate inflammation in peripheral cells, then activated immune responses of the body (Sonenshine and Macaluso, 2017). Therefore, the role of necroptosis in the neurological diseases involved a variety of biological mechanisms related to neuroinflammation (Liu et al., 2014; Bian et al., 2017; Shao et al., 2018). Nec-1 could inhibit necroptosis and reduce inflammation, which was beneficial to the improvement of pathological damage (Takahashi et al., 2012). The increased inflammation was associated with stress-related affective disorders (Stein et al., 2018), while the activations of immune system and inflammatory cytokines might be involved in the pathogenesis of some patients with depression (Haapakoski et al., 2016). The involvement of chronic low-grade inflammatory response, compensatory anti-inflammatory reflex system, and cellular mediated immunity might be the key factors leading to depression (Maes, 1995). Also, higher level of inflammation was associated with increased risk of depression (Pasco et al., 2010). The treatment of antidepressants, especially selective serotonin reuptake inhibitors (SSRIs), could significantly reduce the production of pro-inflammatory cytokines and inflammatory markers, such as IL-1β, IL-6 and TNF-α, and increase the production of anti-inflammatory cytokines, such as IL-10 (Berk et al., 2013). Accordingly, anti-inflammatory could be an effective strategy for the treatment of depression and had great research value (Müller and Schwarz, 2010; Hayley, 2011).

Our results explained that the necroptosis inhibitor Nec-1 could improve depressive-like behaviors in mice, which suggested that the necroptosis pathway might be associated with the pathogenesis of depression. Hippocampus, as the most learning and memory function-related brain area, was also the main target of stress injury (Christie, 2015). Studies have found that inflammation of hippocampus and overactivation of microglia were important central mechanisms of depression (Streit et al., 2004; Xu et al., 2015), and the regulation of necroptosis on CNS function was also closely related to the occurrence of neuroinflammation (Antunes et al., 2019). Therefore, in order to further investigate the results of network pharmacology, and further explore the relationship between the pathogenesis of depression and necroptosis, we detected the expressions of RIPK1, RIPK3, MLKL and *p*-MLKL in hippocampus of CUMS-induced mice to confirm the level of necroptosis. It was observed that RIPK1, RIPK3 and MLKL in hippocampus of model mice were significantly up-regulated, and the phosphorylation level of MLKL was also increased, meanwhile, Nec-1 played a significant role in regulating the expressions of the above indicators. Phosphorylated MLKL was the most precise protein, which could definitely indicate the necroptosis activated via RIPK-mediated MLKL signaling (Rodriguez et al., 2016). In other words, the phosphorylation of MLKL at ser345 was crucial for the recruitment of MLKL and the activation of necroptosis pathway (Rodriguez et al., 2016). We also found high level of the *p*-MLKL colocalized with thioflavin T staining in the hippocampus of CUMS group mice, indicating that MLKL was phosphorylated in the presence of amyloid-like polymers, characteristic of necrosome complexes (Li et al., 2012; Liu et al., 2017).

Finally, our results displayed a large number of inflammatory cells infiltrated into the hippocampus of CUMS-exposed mice and revealed that the expressions of IL-1β, Lipocalin-2 and IBA1 were significantly increased, which proved that the existence of neuroinflammation and excessive activation of microglia were associated with the necroptosis pathway. Necroptosis can be specifically blocked by small molecule compound Nec-1, but not by specific inhibitors of apoptosis and autophagy, such as z-vad. fmk (N-benzyloxycarbonyl Val ala ASP (o-me) fluoromethylketone) and 3-methyladenine (3 mA). Many studies on different perspectives have revealed that the inhibition of necroptosis mediated by RIPK1/RIPK3/MLKL provides a protective effect against inflammation (Moriwaki and Chan, 2016; Duan et al., 2020; Kumari et al., 2021). Therefore, it was found that Nec-1 could also suppress neuroinflammation and activation of microglia in hippocampus. It was reported that Lipocalin-2 was an inflammatory-related protein closely correlated to hippocampal structure and function, and it might be a key regulator of emotional behaviors and cognitive function (Ferreira et al., 2013). Lipocalin-2 secreted by microglia and astrocytes participated in the activation of microglia, then excessive activation of microglia caused neuroinflammation and injured the structure and function of neurons (Kim et al., 2017). Normally, there was low expression of Lipocalin-2 in hippocampus, but it might continuously increase under stress stimulation, which was related to its ability to regulate the formation and maturation of dendritic spines of neurons in hippocampal CA1-CA3 regions (Mucha et al., 2011). Moreover, the activation of MLKL during necroptosis triggered the release of IL-1β, IL-6 and TNF(Wu et al., 2013). The inflammatory cytokines such as IL-1β might be the key substance regulating the expression of Lipocalin-2 (Hu et al., 2015). At this point we could conclude that necroptosis might activate the Lipocalin-2 involved inflammatory pathway, induce the neuroinflammation and microglial over-activation, and then participate in the functional changes of hippocampus leading to depressive-like behaviors in depressed mice. Grounded on rigorous science, the TCM formula Xiaoyaosan was an antidepressant therapy conceptually based on a multiple component, multiple targeting principle. As showed in this study, Xiaoyaosan might mediate the RIPK1-RIPK3-MLKL signaling and its subsequent inflammatory pathway, regulate the inflammatory-related mediator Lipocalin-2, and then improve the function of hippocampal microglia to exert antidepressant and anti-inflammatory effect.

## Conclusion

According to the network pharmacological study, the active ingredients of Xiaoyaosan may exert the antidepressant effect via 23 necroptosis related potential targets. Then, the animal experiment found that the RIPK1-RIPK3-MLKL signaling was triggered in the stress-induced depression in mice. These findings were consistent with the cytokine hypothesis of depression that postulated stress may activate the necroptosis pathway in hippocampus, leading to neuroinflammation and microglial activation, then contribute to the development of depressive-like behaviors. Moreover, our findings further illustrated the possible mechanism of Xiaoyaosan with explicit experiment results that it could regulate the necroptosis mediated inflammatory signaling pathway to alleviated the depressive symptoms of mice, which provided an experimental basis for the clinical application of Xiaoyaosan in the treatment of depression. For future research, whether necroptosis can be used as a new potential pathway for the treatment of depression still needs to be confirmed with RIPK or MLKL knockout mice, and the functional impact of necroptosis in depression with the specific role of the active components of Xiaoyaosan on neuroinflammation could be further explored by more novel and accurate network pharmacological analysis.

## Data Availability

The original contributions presented in the study are included in the article, further inquiries can be directed to the corresponding authors.

## Abbreviations

ADME, absorption, distribution, metabolism and enhancement; ANOVA, one-way analysis of variance; BDNF, brain-derived neurotrophic factor; CTD, Comparative Toxicogenomic database; CUMS, chronic unpredictable mild stress; CNS, central nervous system; DL, drug likeness; DMSO, dimethyl sulphoxide; DAMPs, damage-associated molecular pattern molecules; ECL, enhanced chemiluminescence; ETCM, Encyclopedia of Traditional Chinese Medicine; FS, forced swimming; H&E, hematoxylin and eosin; HPA, hypothalamic pituitary adrenal; HRP, horseradish peroxidase; HERB, High-throughput Experiment- and Reference-guided database of Traditional Chinese Medicine; IF, immunofluorescence; IHC, immunohistochemical; LSD, least significant difference; MOD, mean optical density; MFI, mean fluorescence intensity; MLKL, mixed lineage kinase domain-like; Nec-1, necrostatin-1; NSF, novelty-suppressed feeding; OB, bioavailability; OMIM, Online Mendelian Inheritance in Man; PFA, paraformaldehyde; PPI, protein–protein interaction; PVDF, polyvinylidene fluoride; RIPK, receptor interacting protein kinas; SAC, sacrificed; SP, sucrose preference; SPF, specific-pathogen free; SEM, standard error of mean; SSRIs, selective serotonin reuptake inhibitors; TCM, traditional Chinese medicine; TCMSP, TCM Systems Pharmacology database and Analysis Platform; TBST, tris buffered saline tween; TLR, toll-like receptor; TNF, tumor necrosis factor; TTD, Therapeutic Target database; UPLC-MS/MS, ultra-performance chromatography-electrospray tandem mass spectrometr; UniProt, universal Protein Resource; WB, western blot; WHO, World Health Organization.
