# Supplementary figures and images for: Antidepressant Mechanism of Traditional Chinese Medicine Formula Xiaoyaosan in CUMS-Induced Depressed Mouse Model via RIPK1-RIPK3-MLKL Mediated Necroptosis Based on Network Pharmacology Analysis
Source: Front Pharmacol. 2021 Nov 19;12:773562. doi: 10.3389/fphar.2021.773562 (PMC8641697; doi:10.3389/fphar.2021.773562)

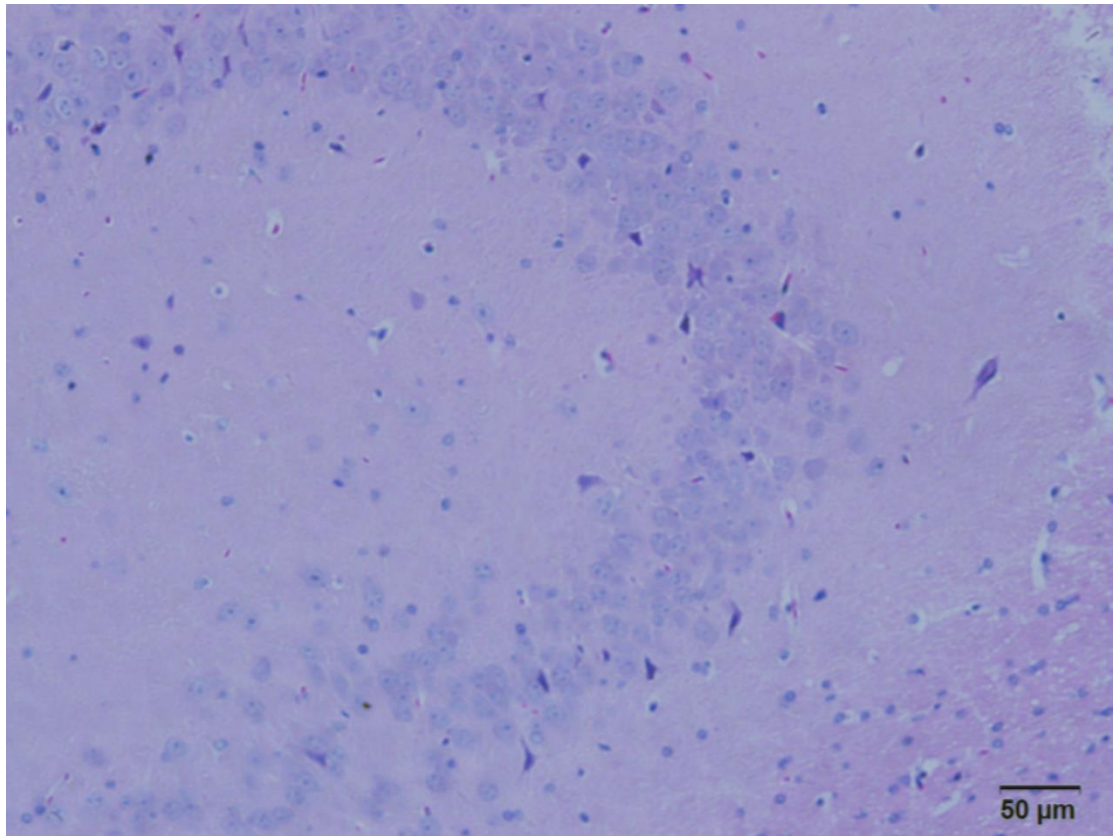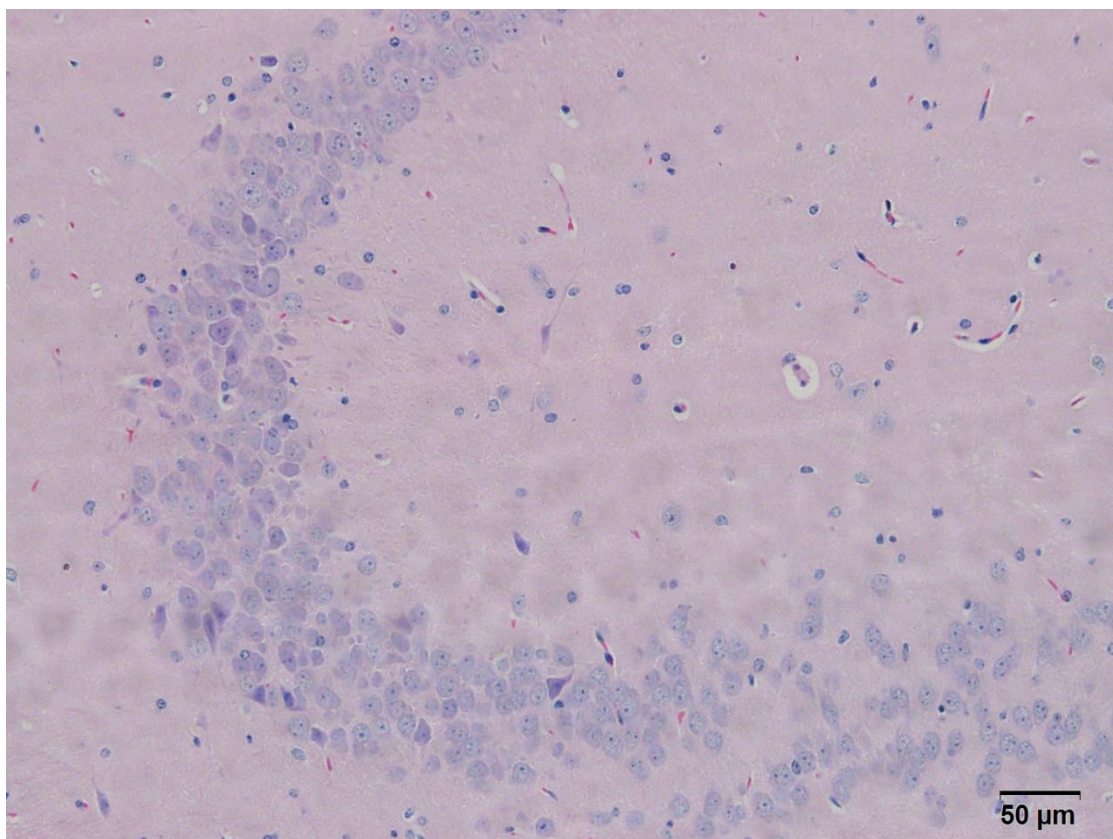

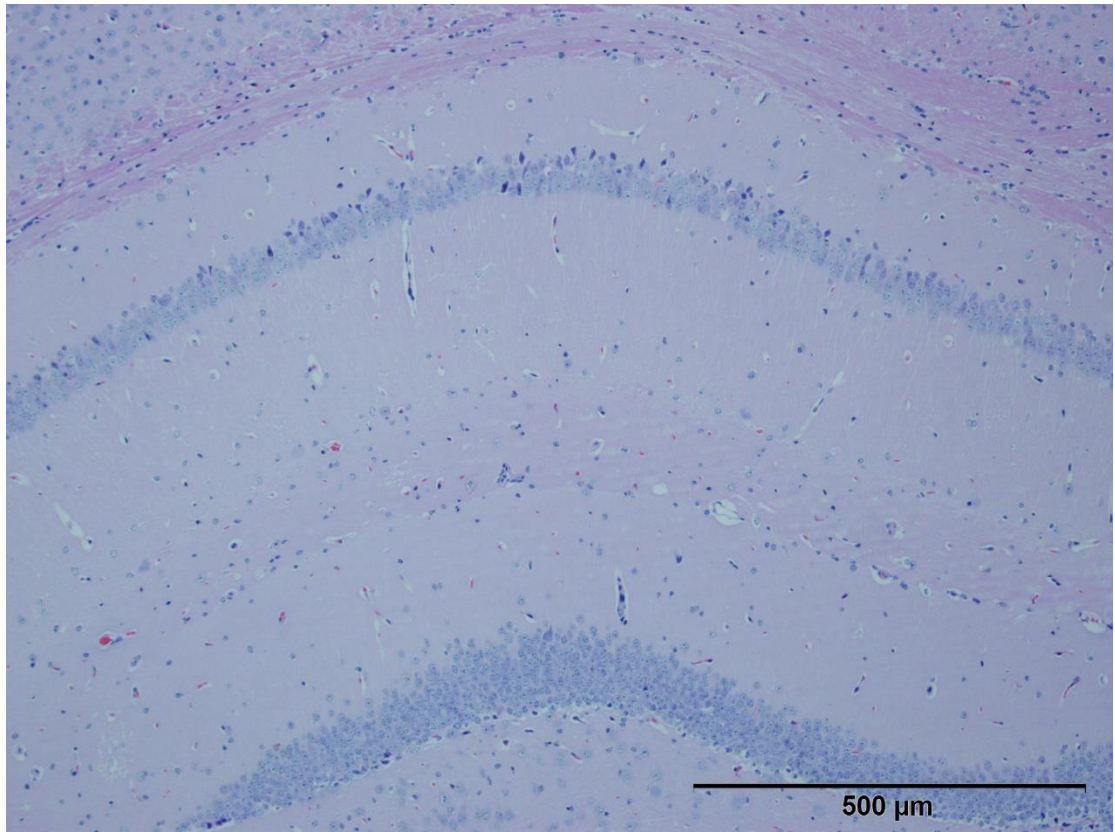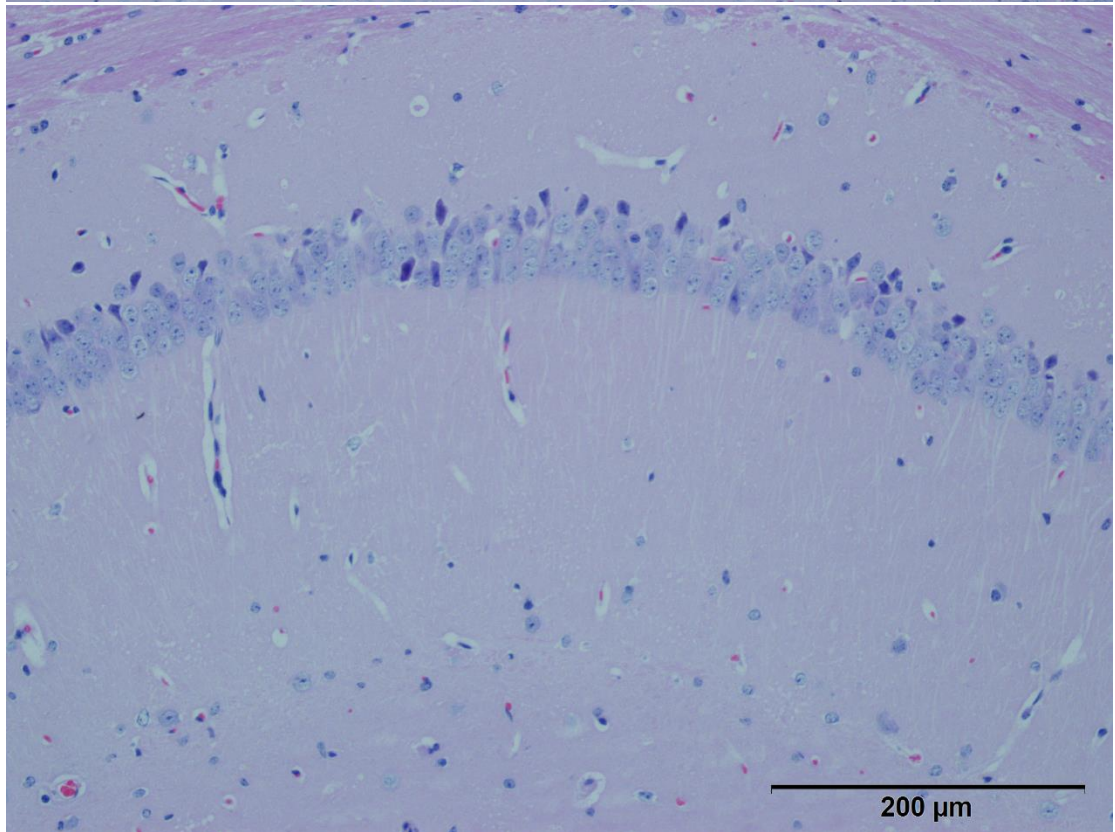

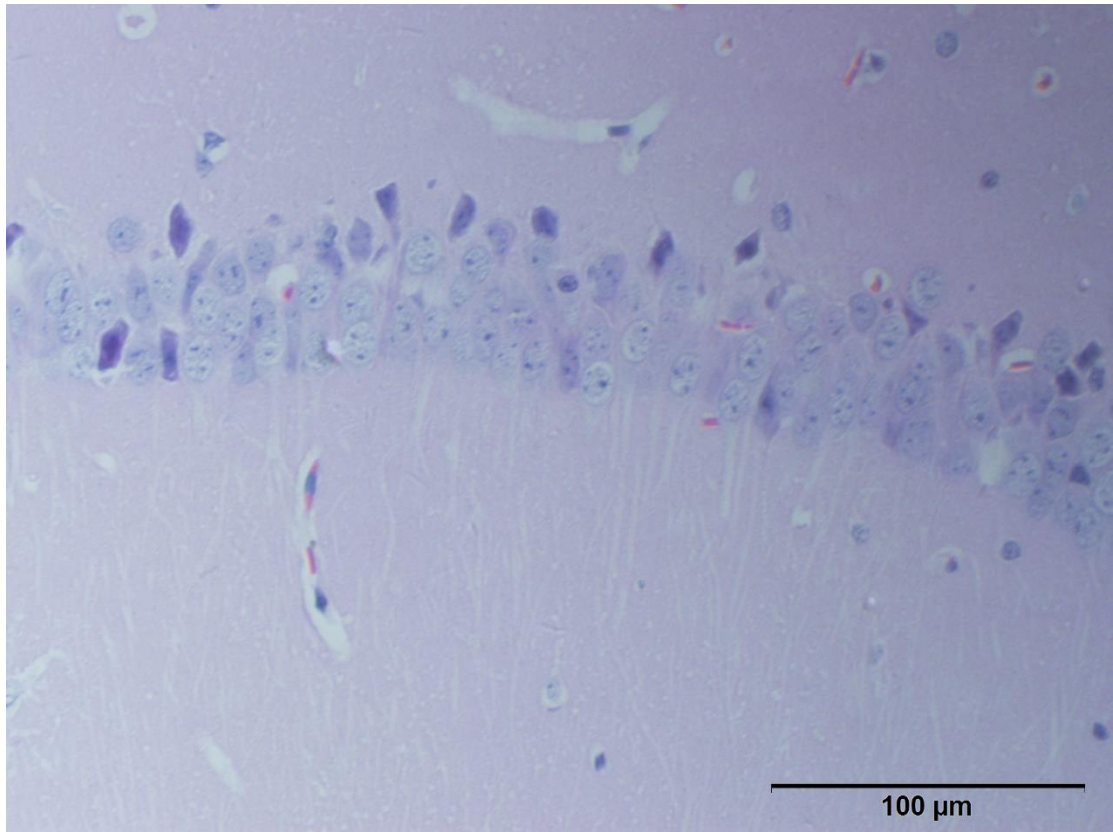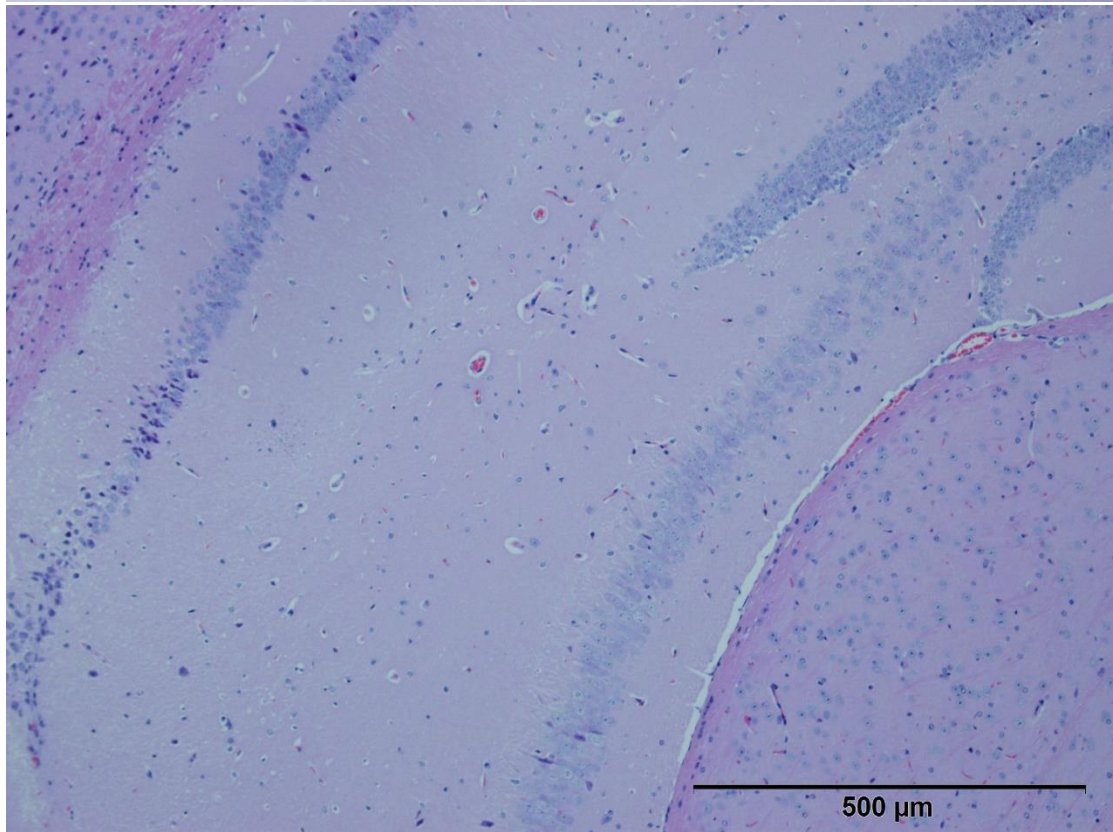

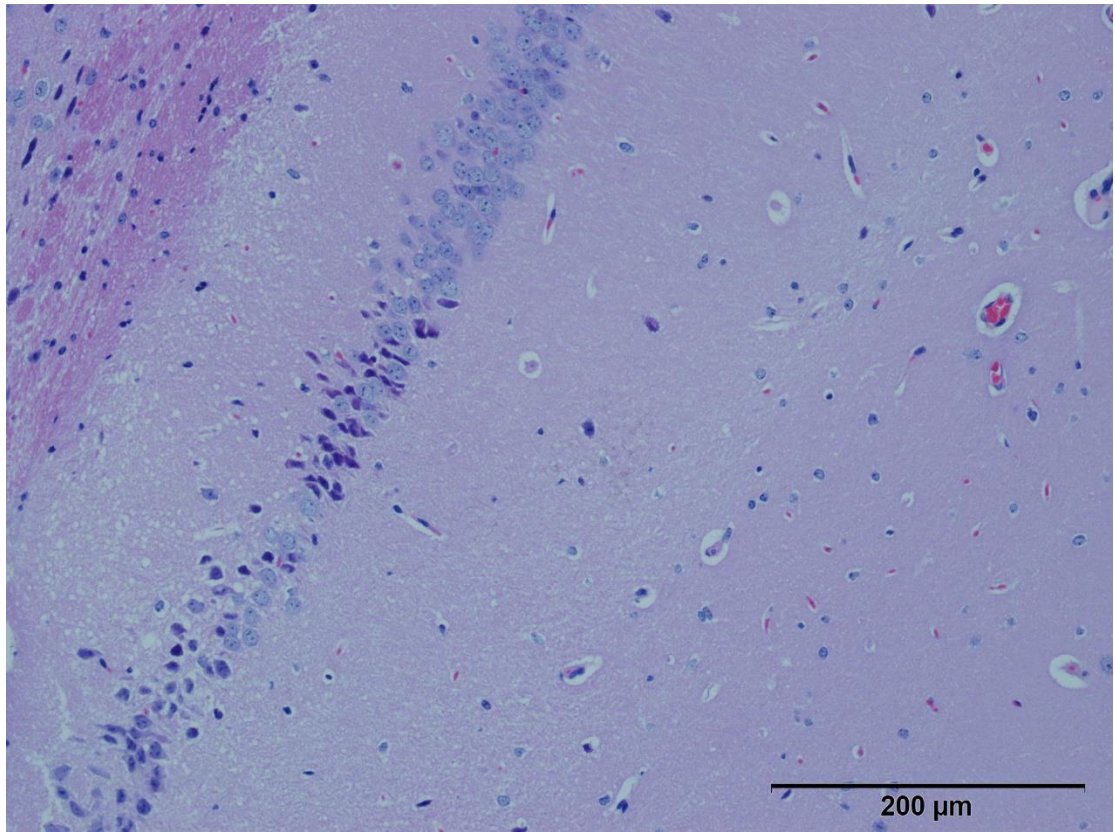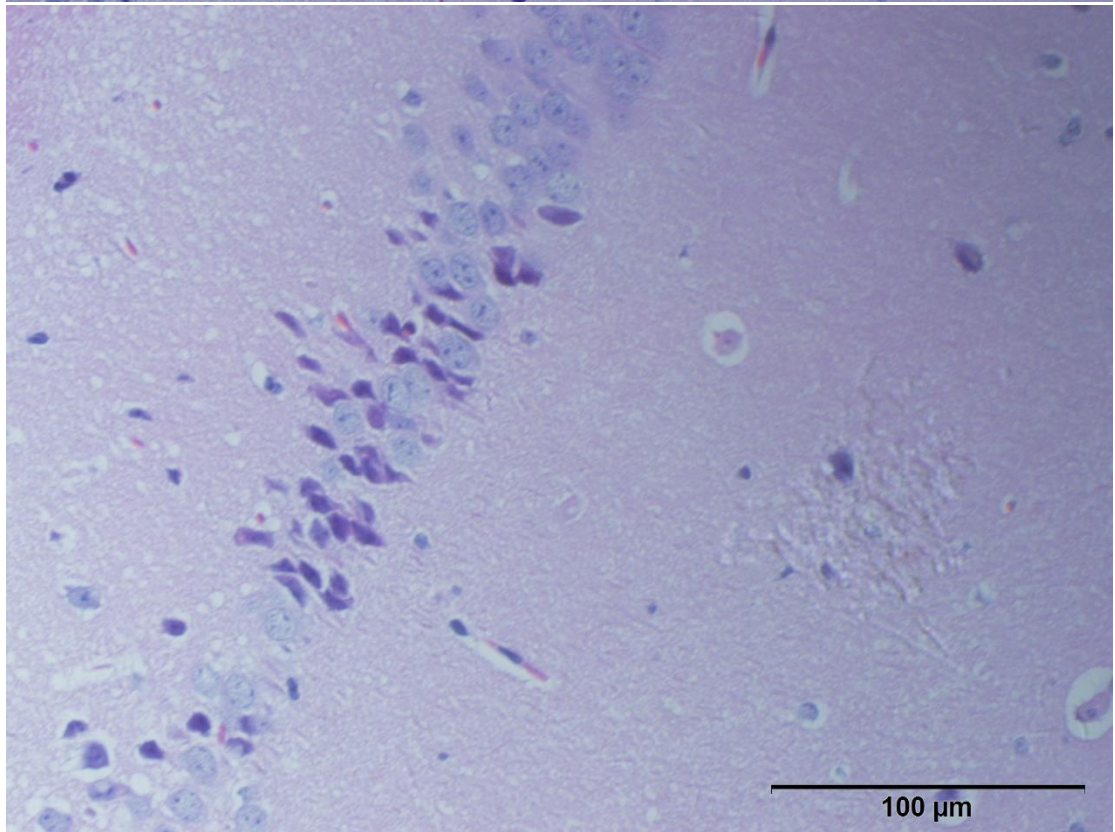

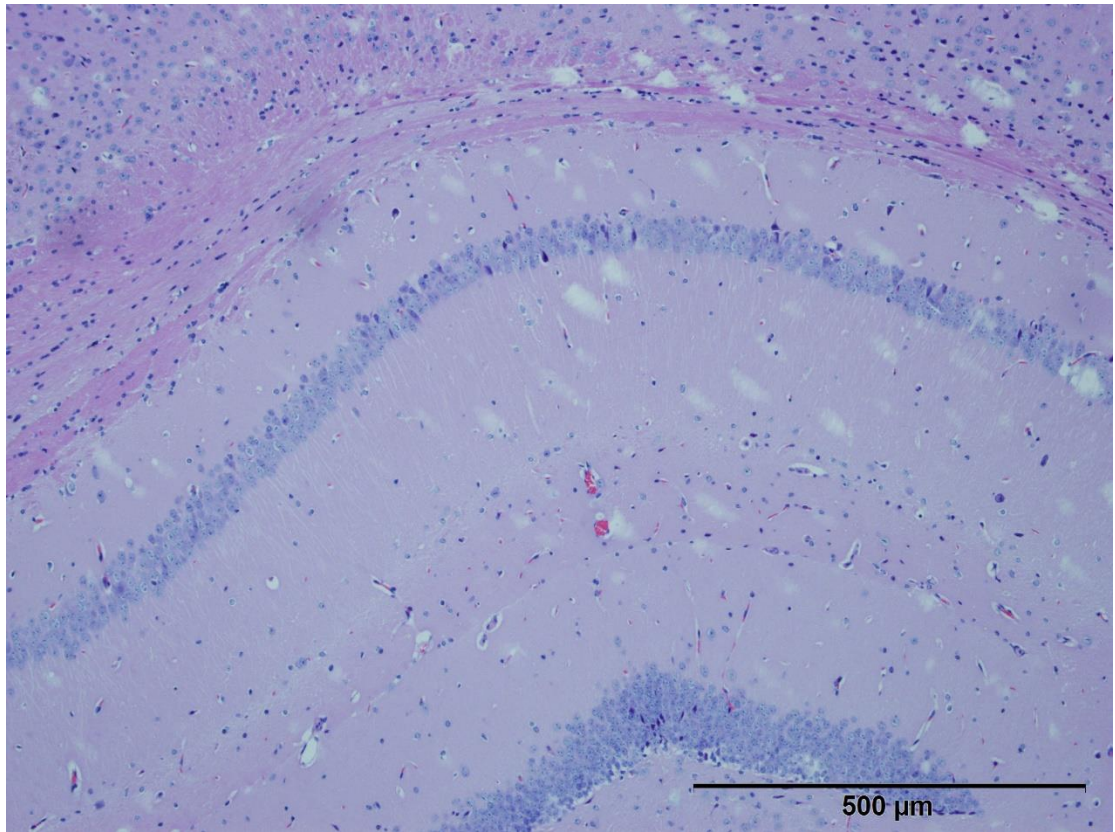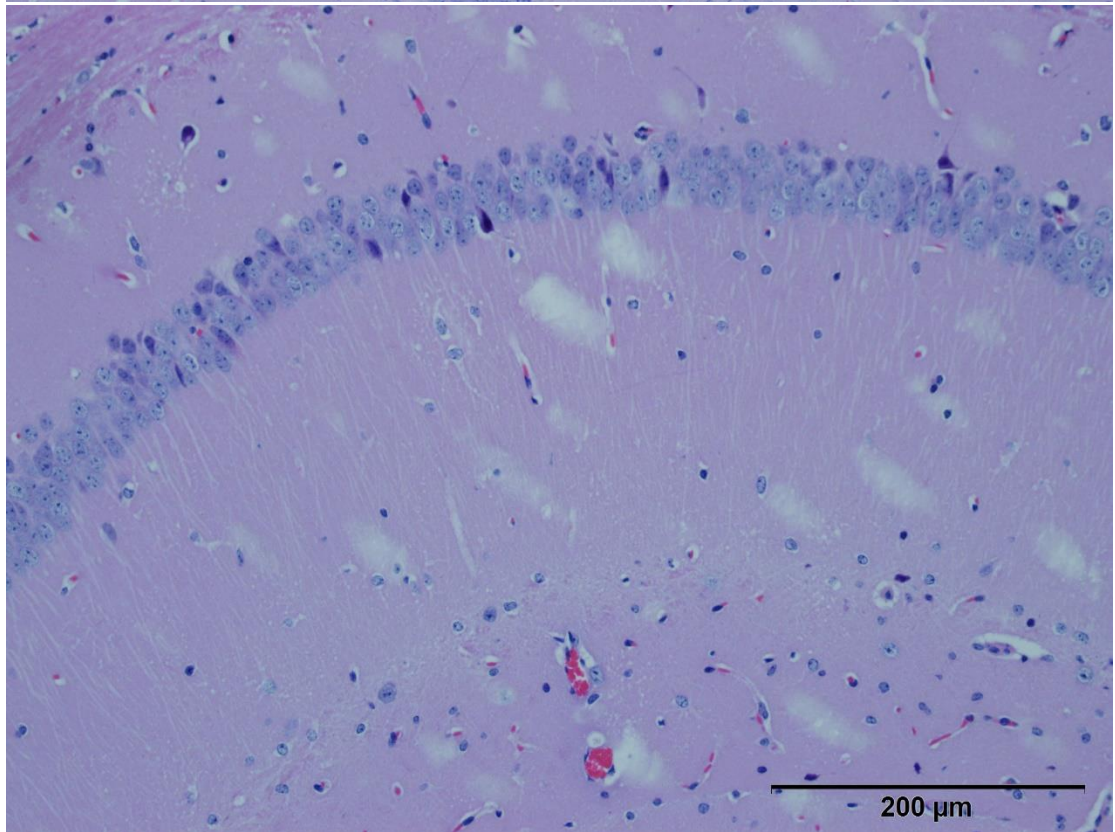

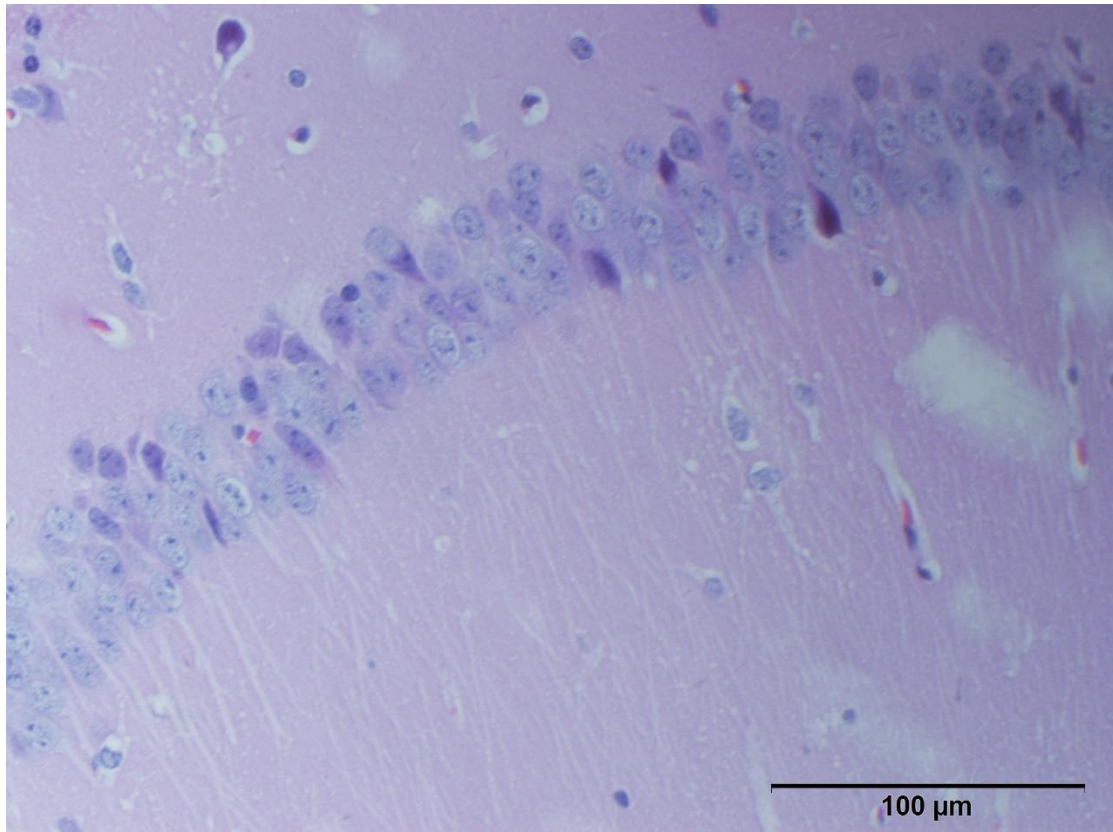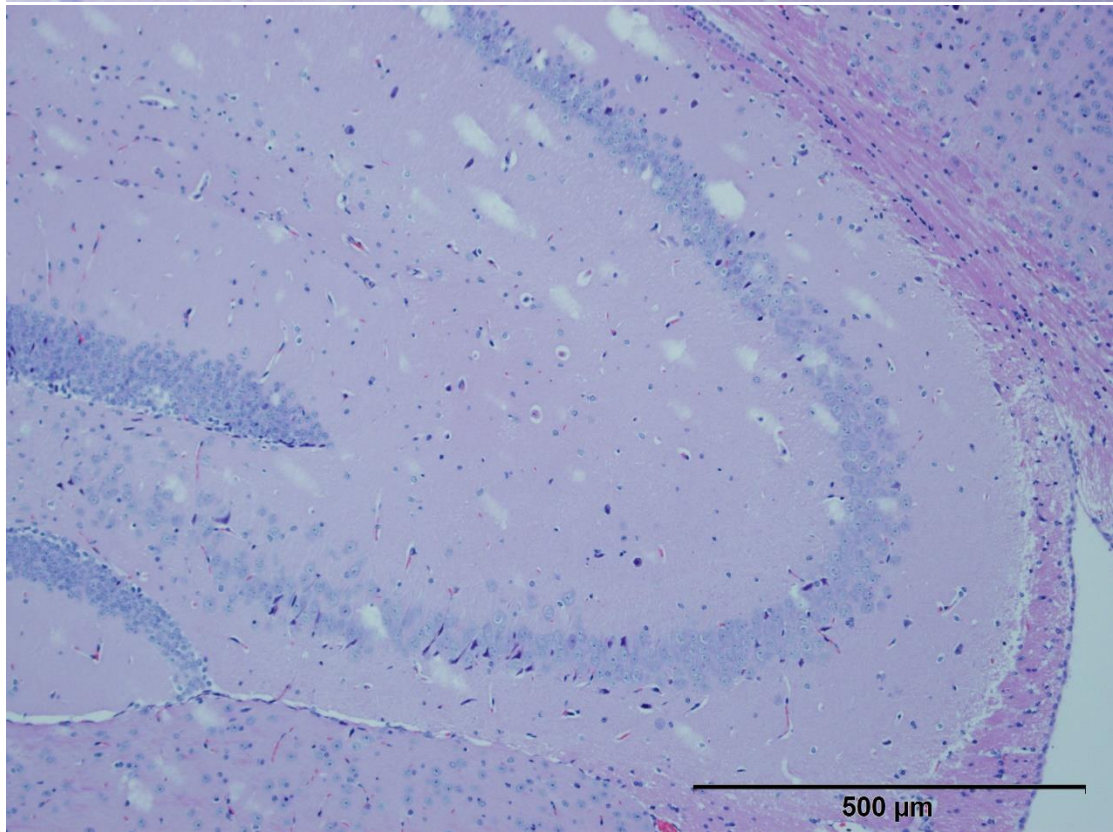

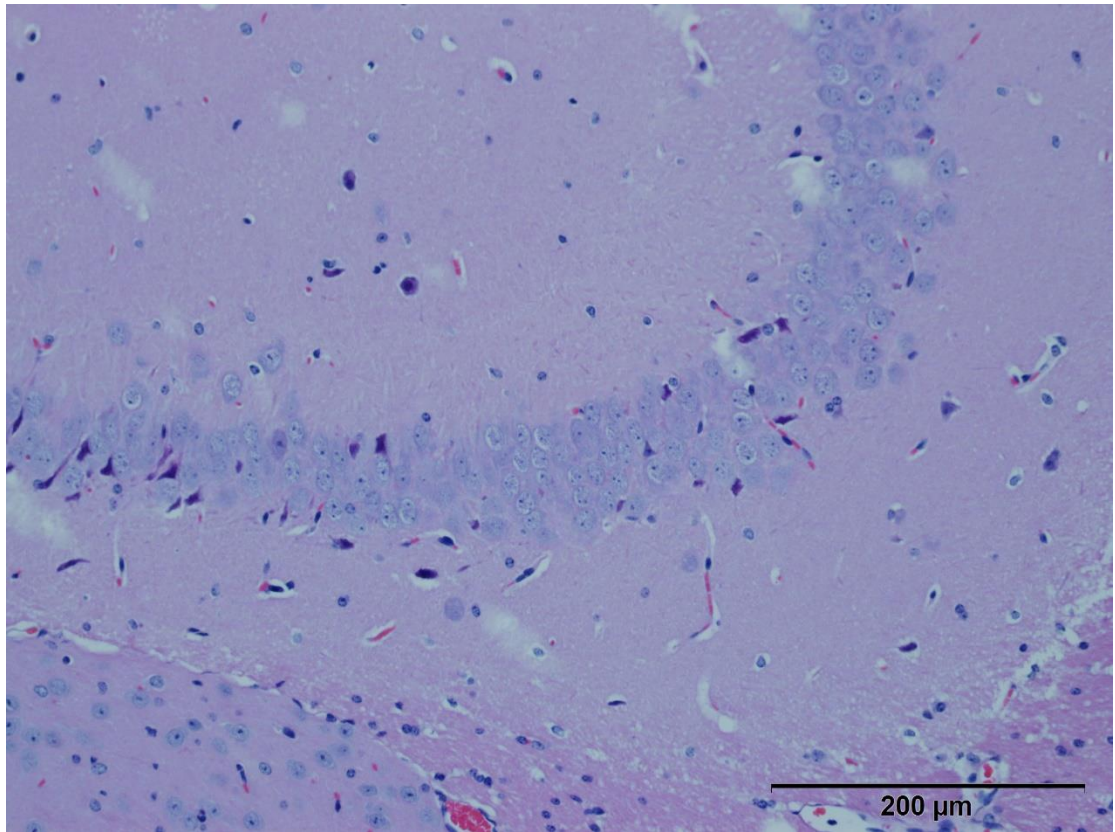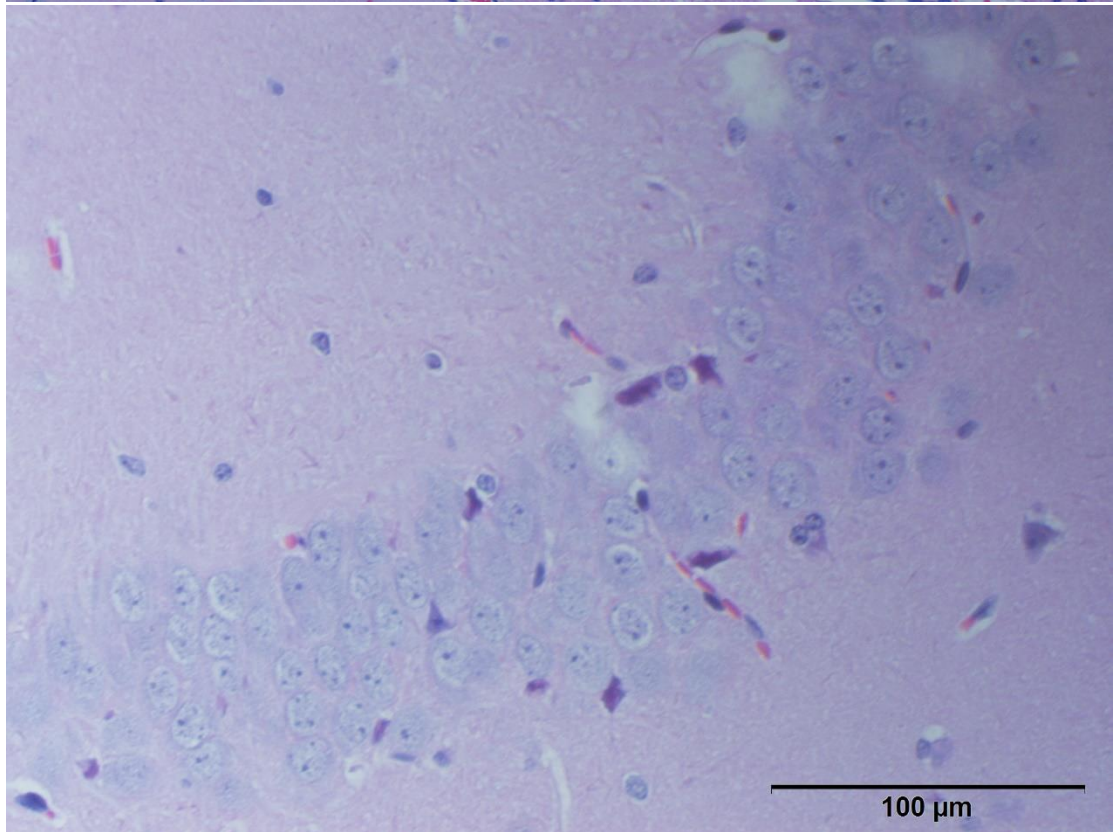

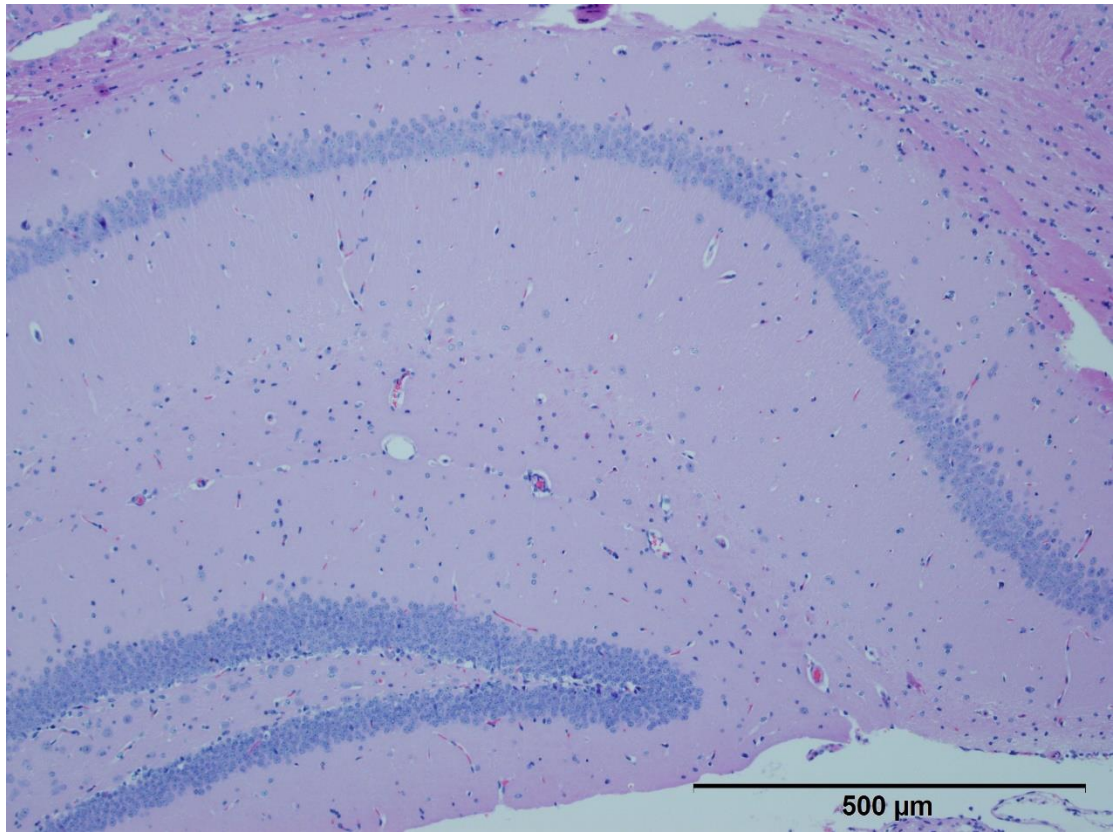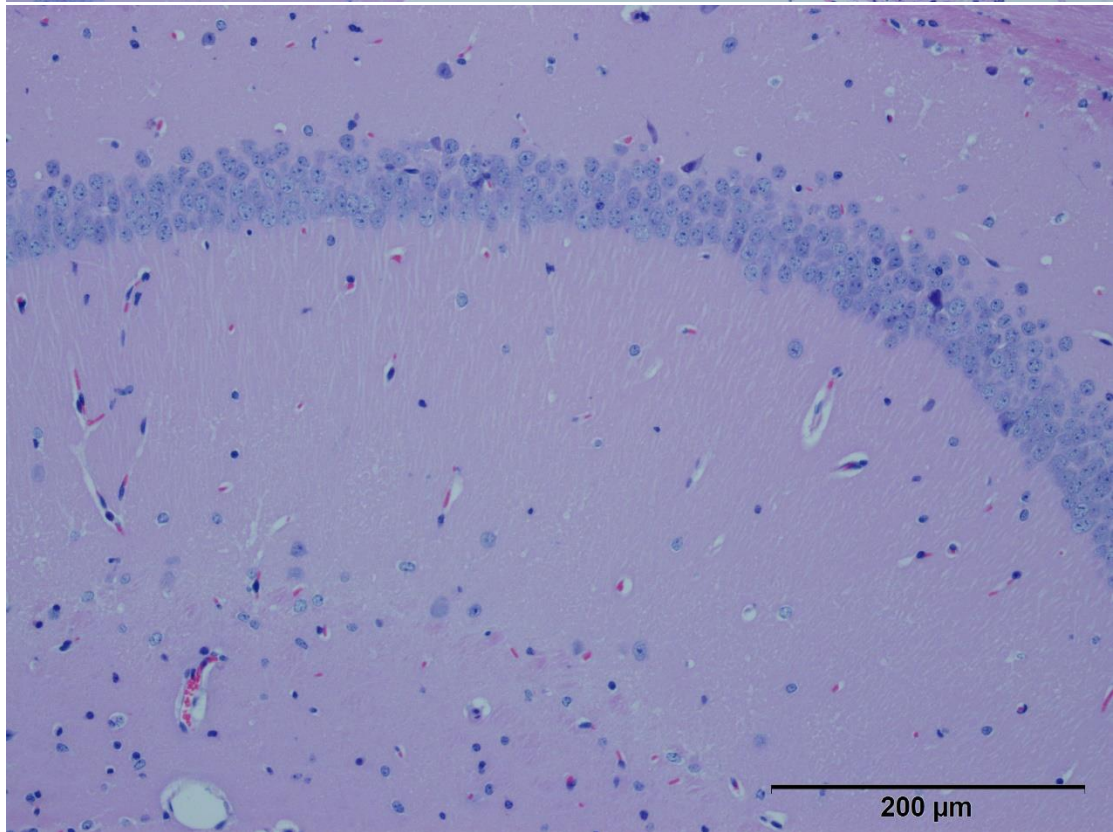

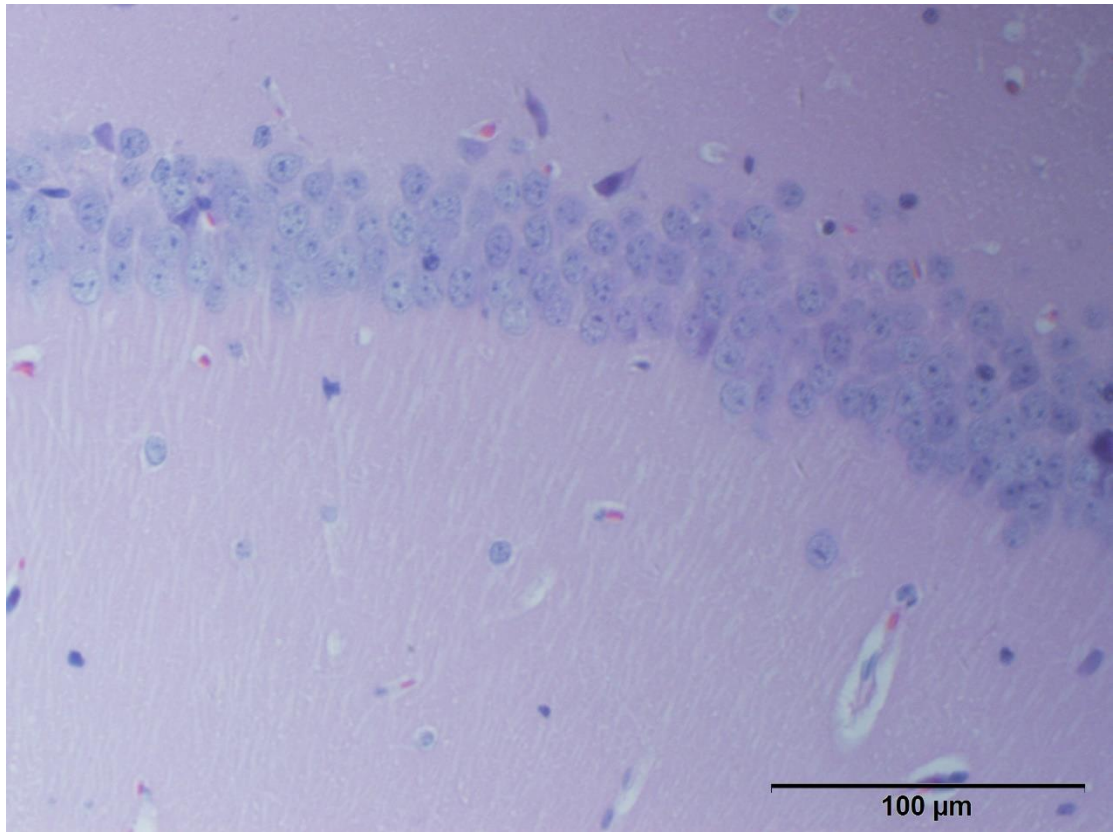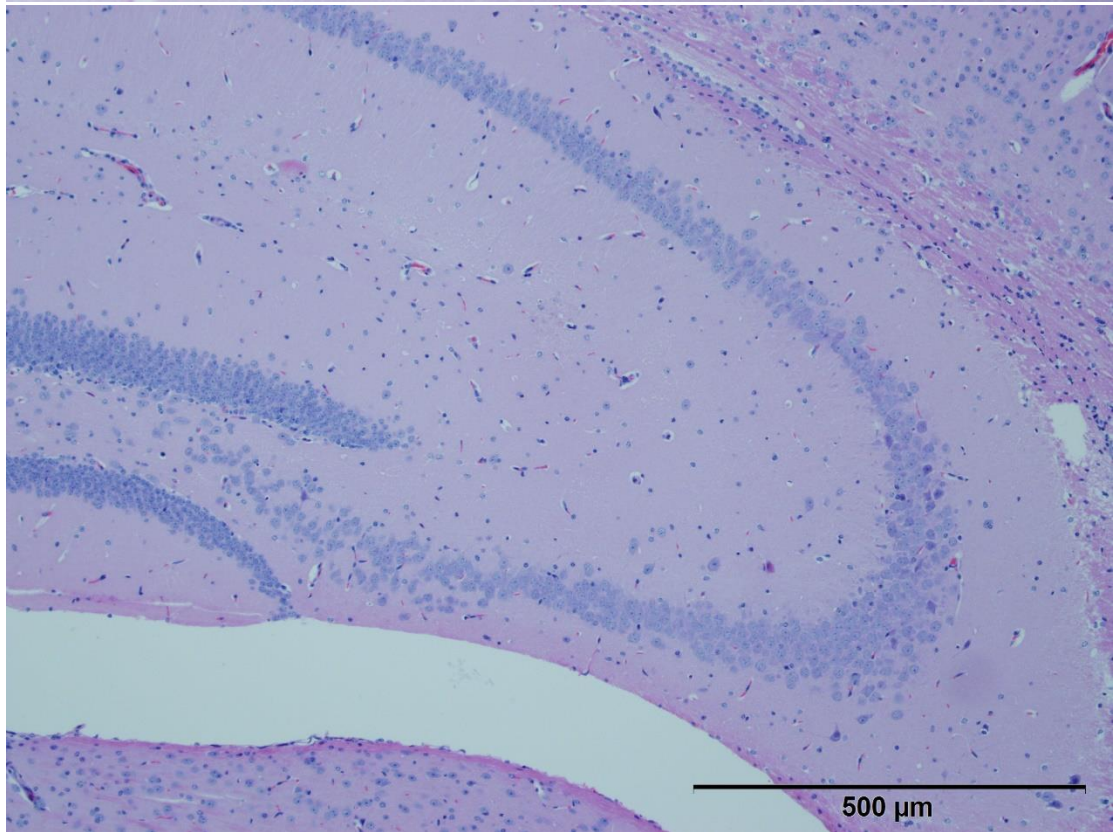

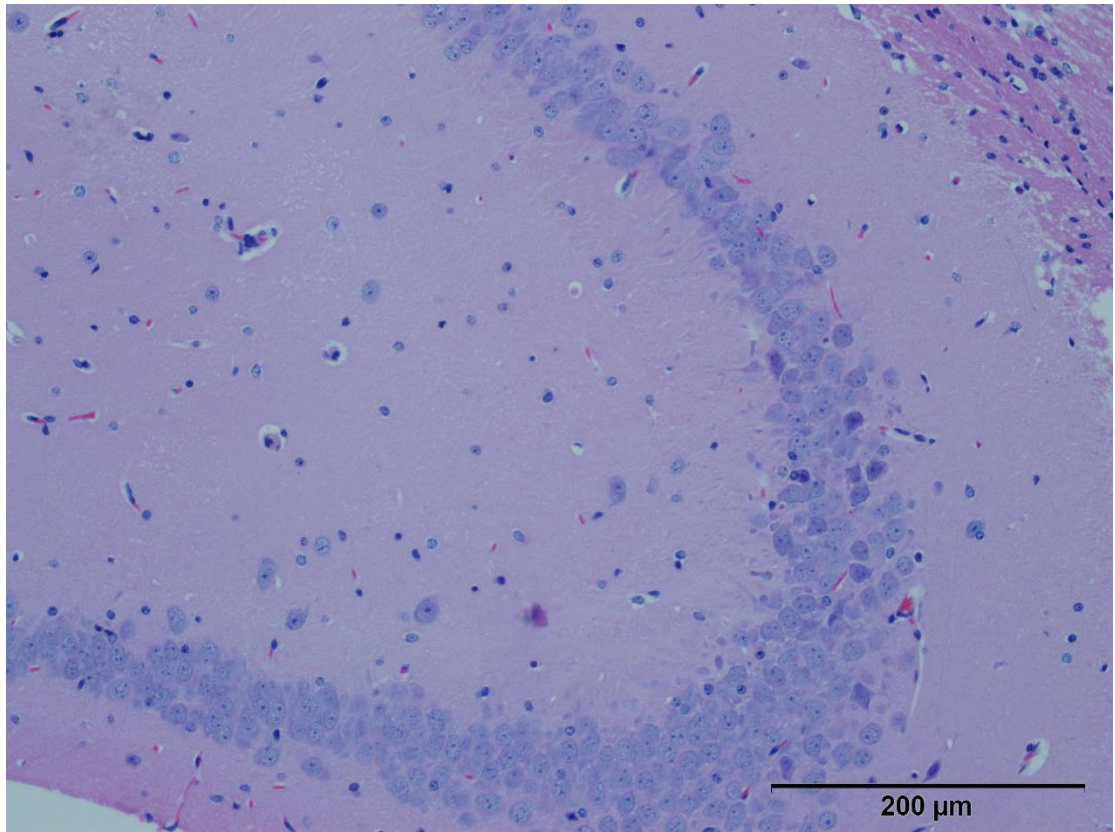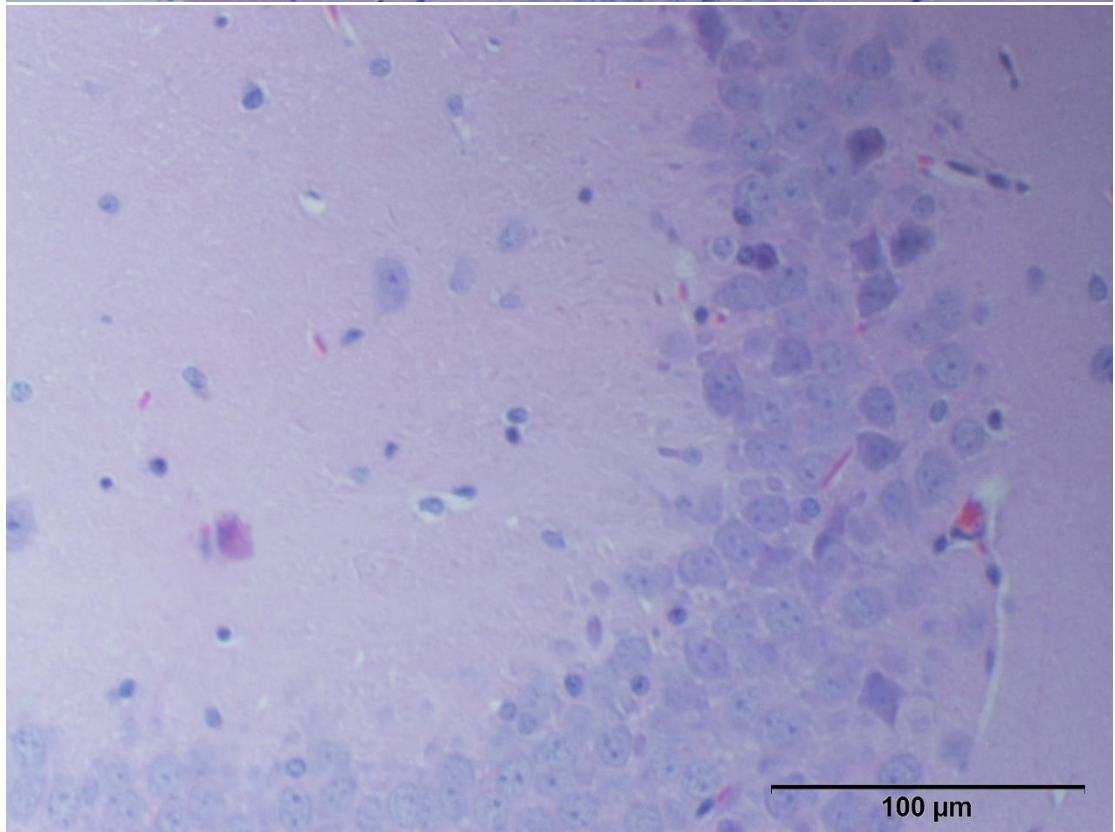

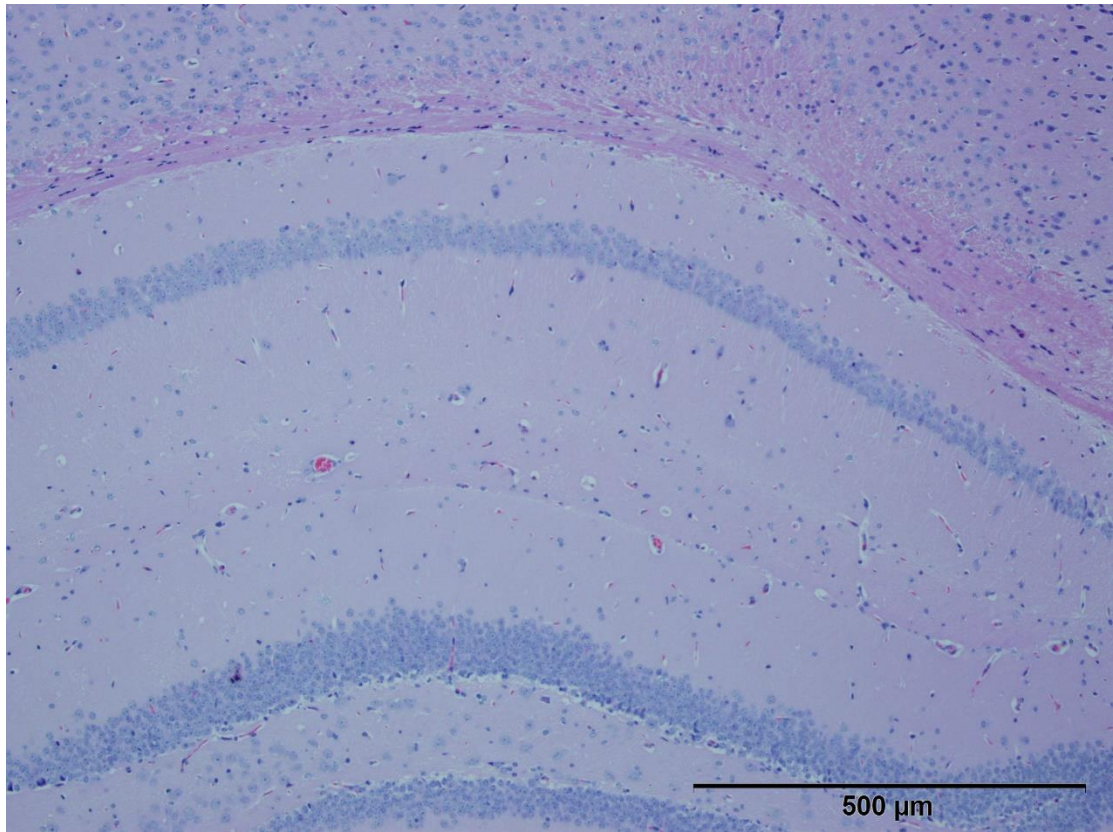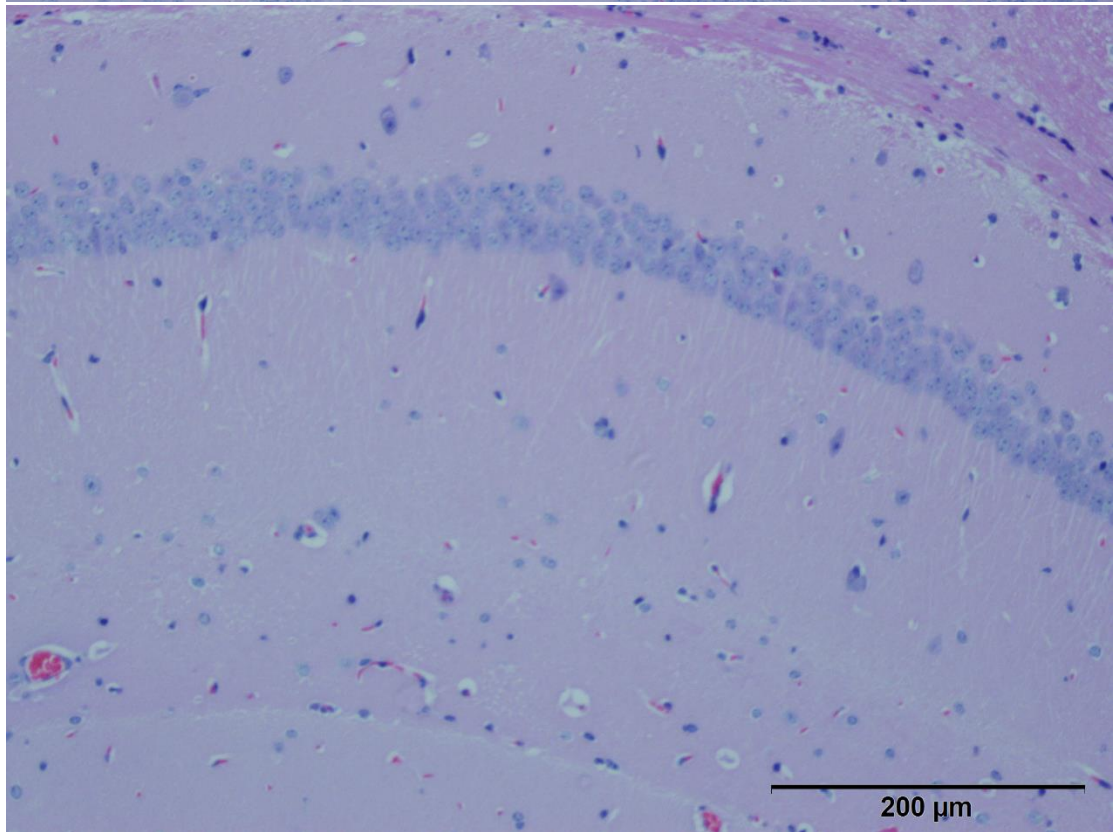

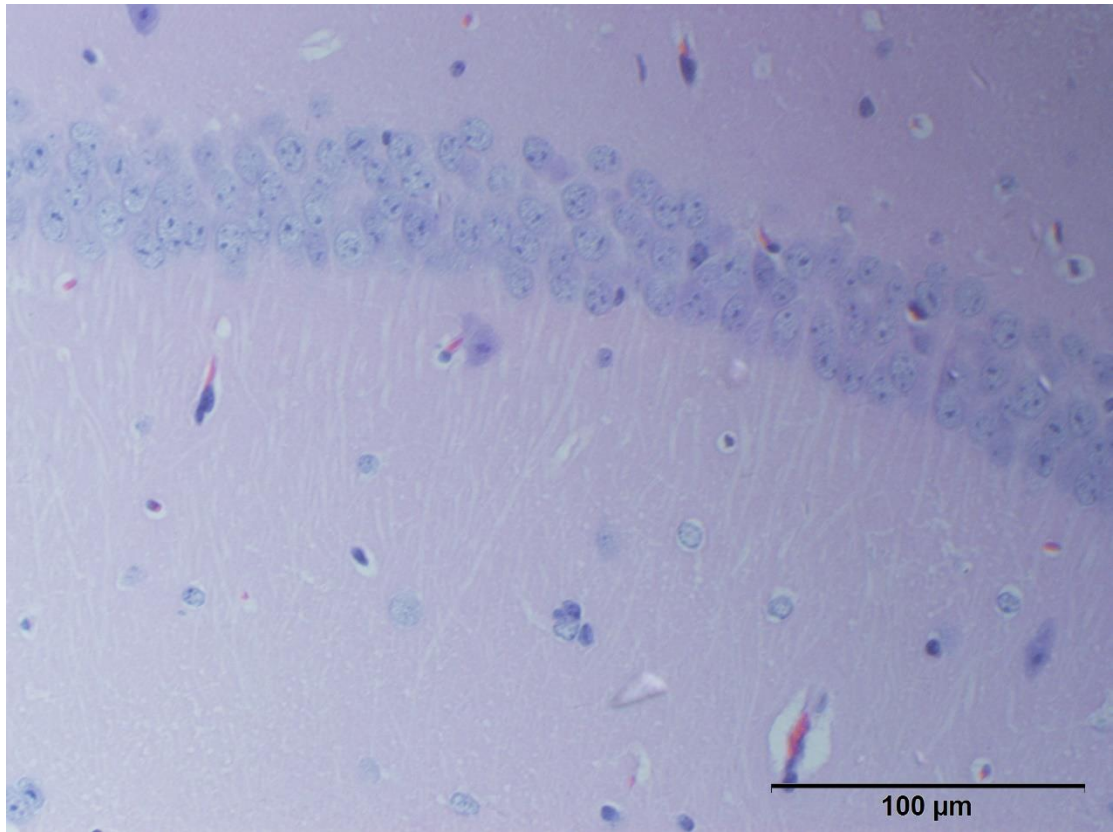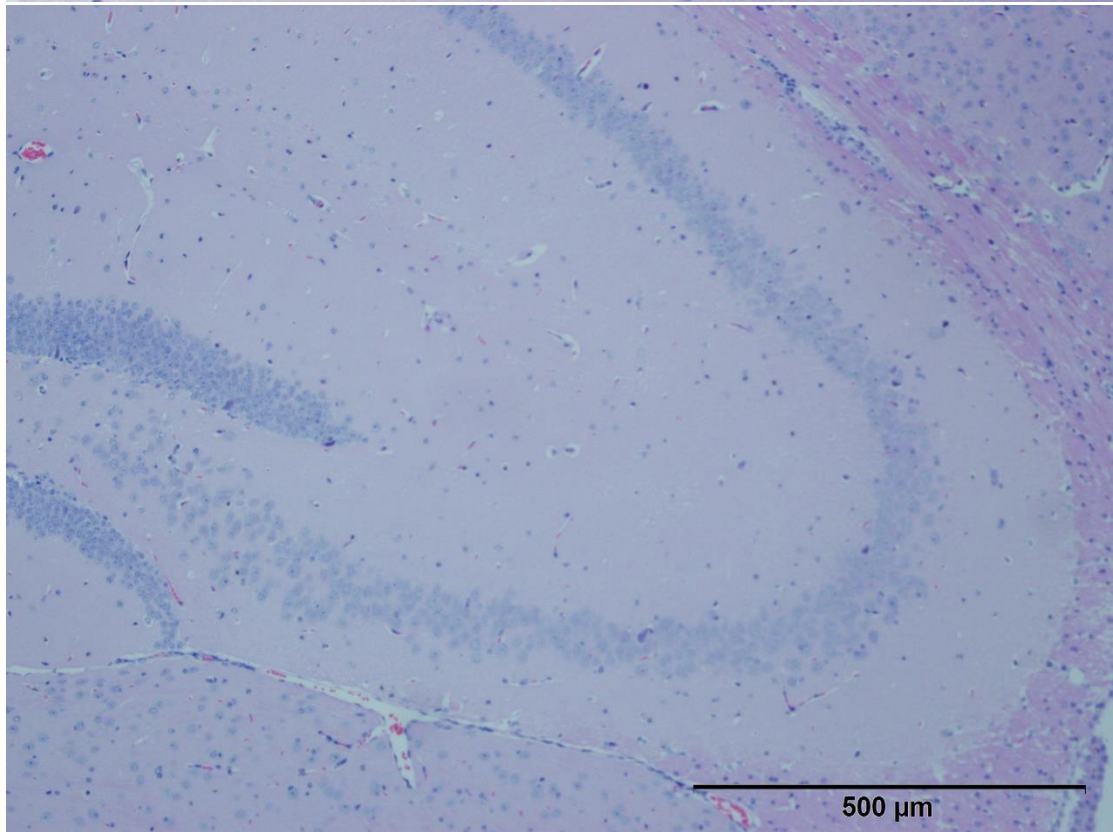

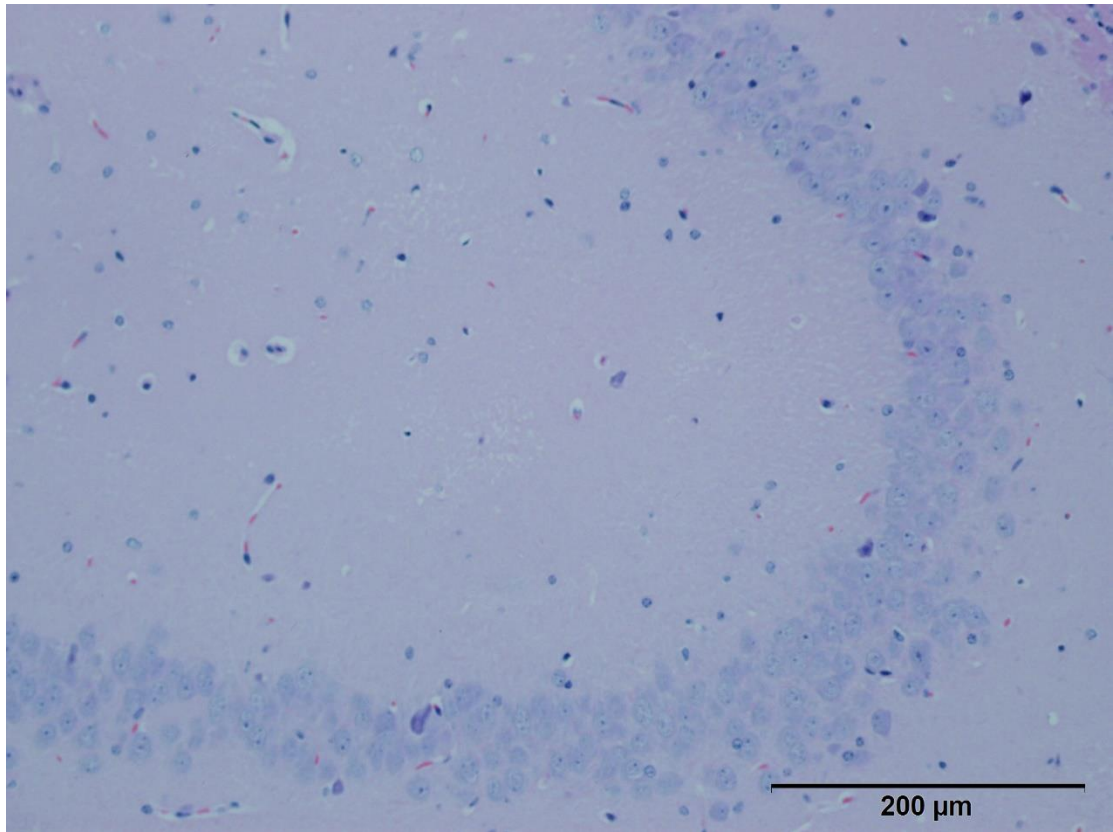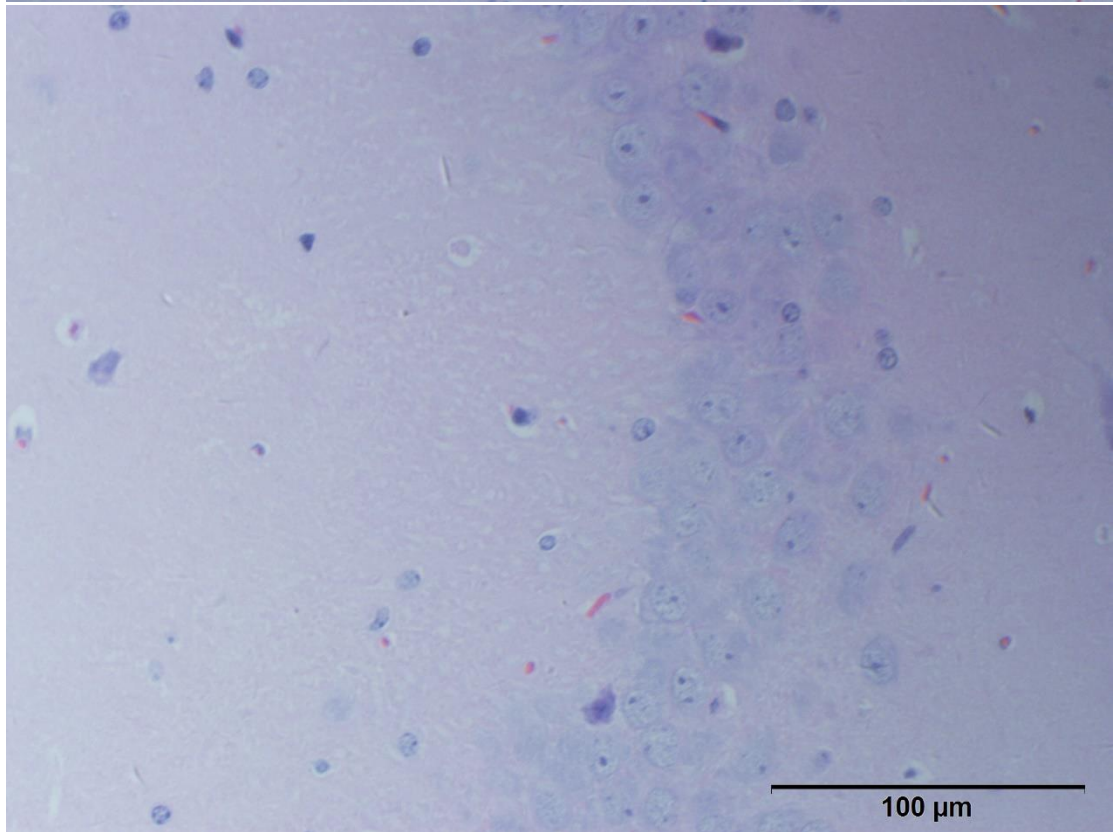

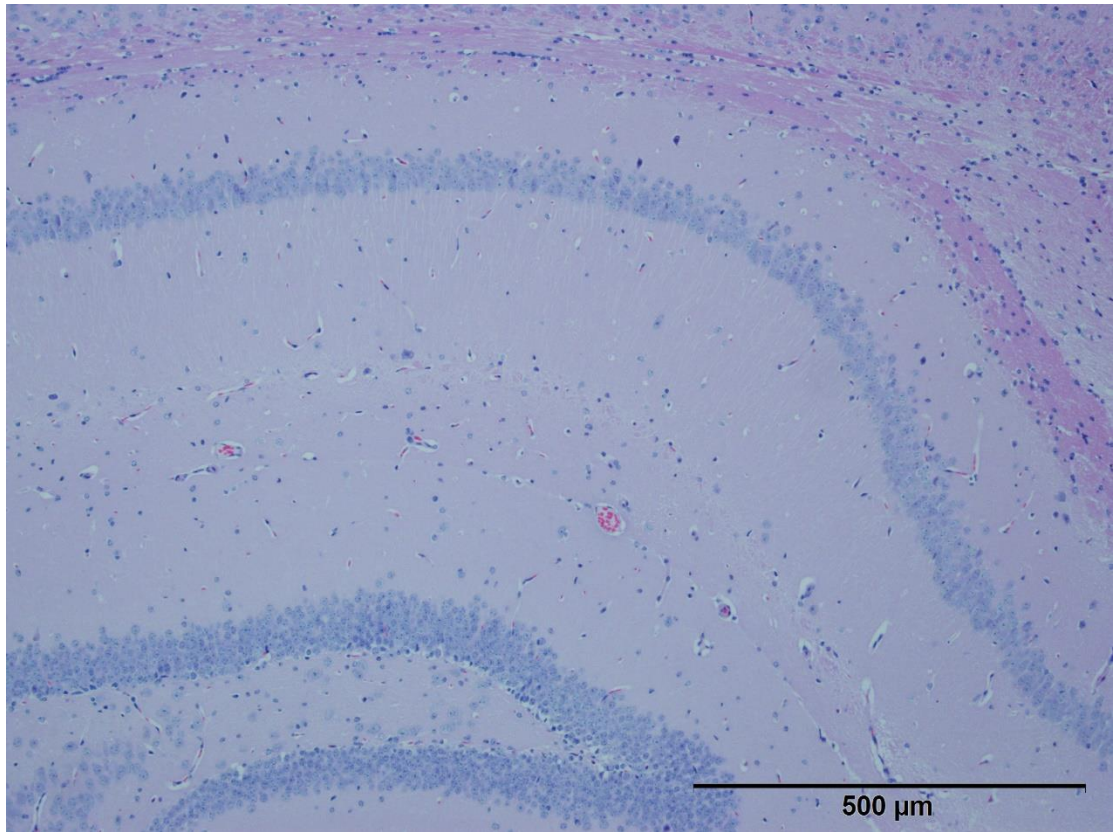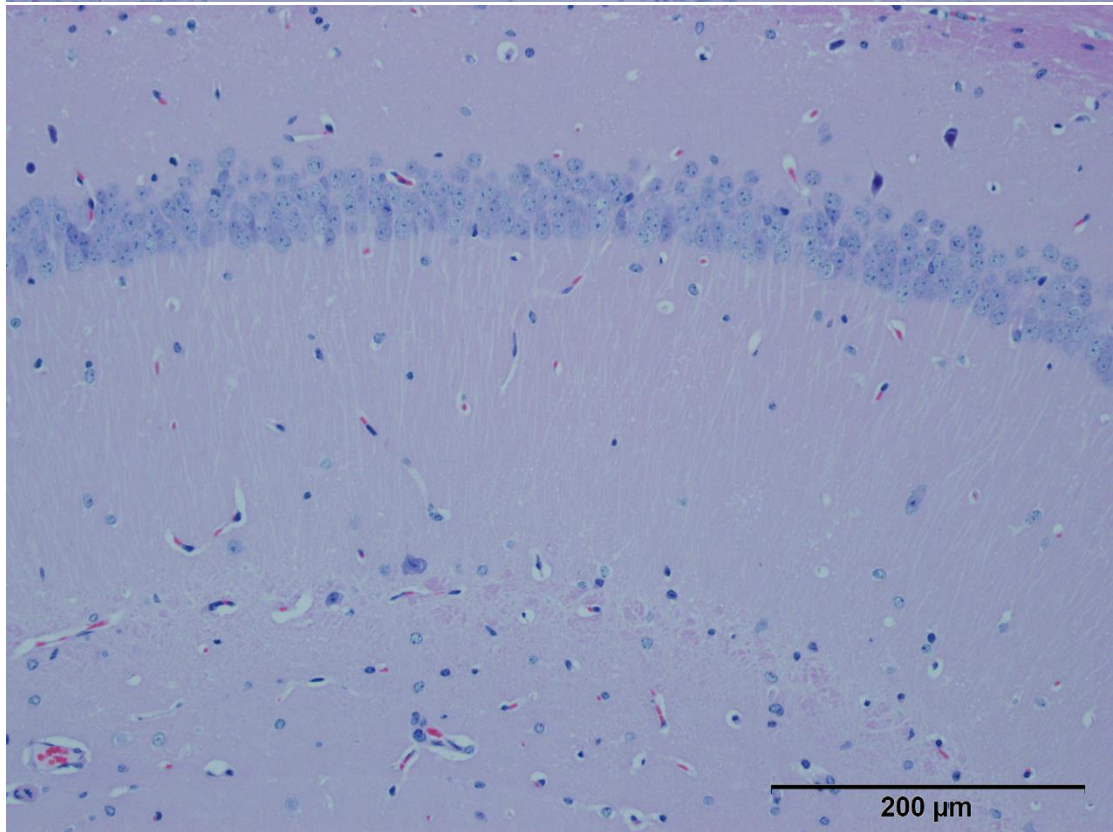

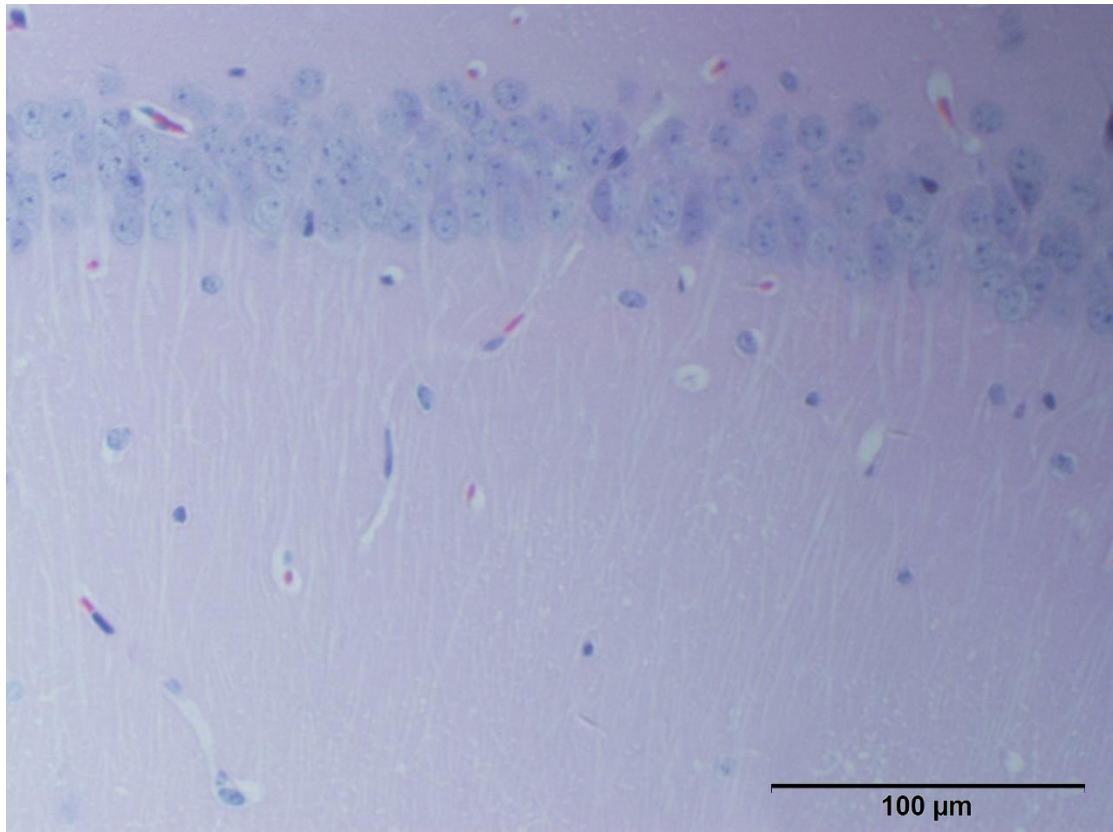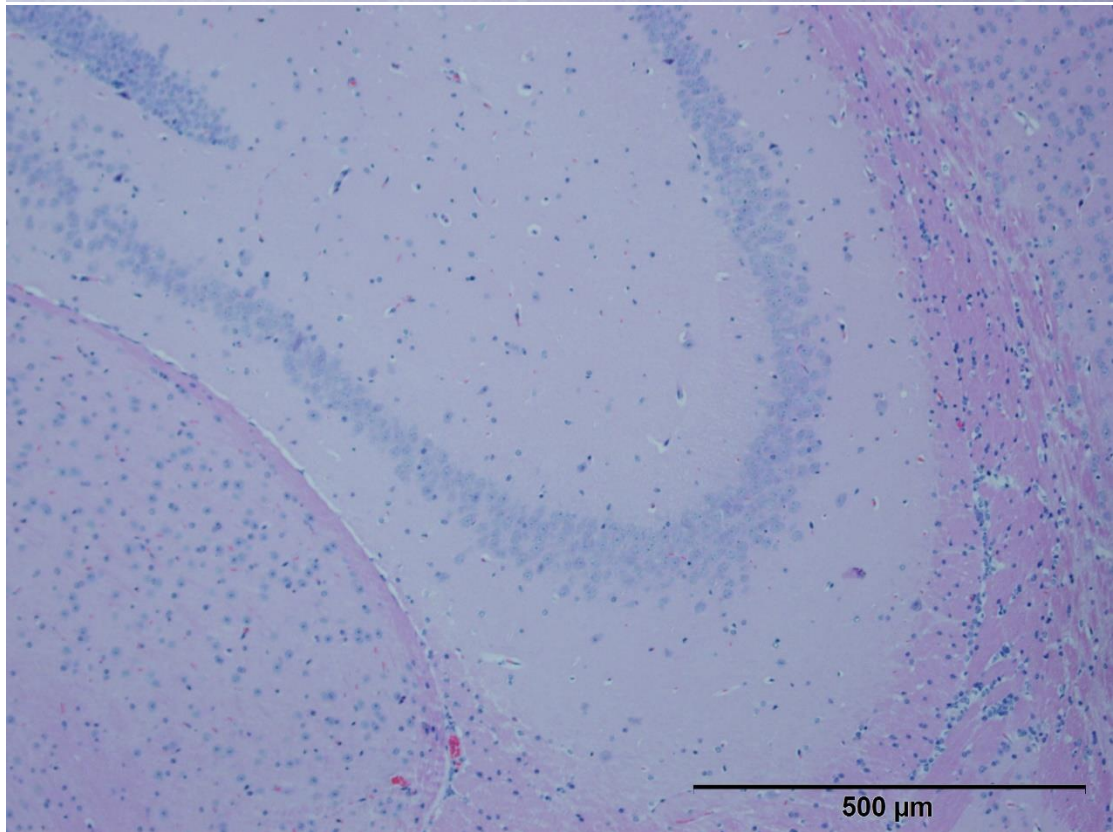

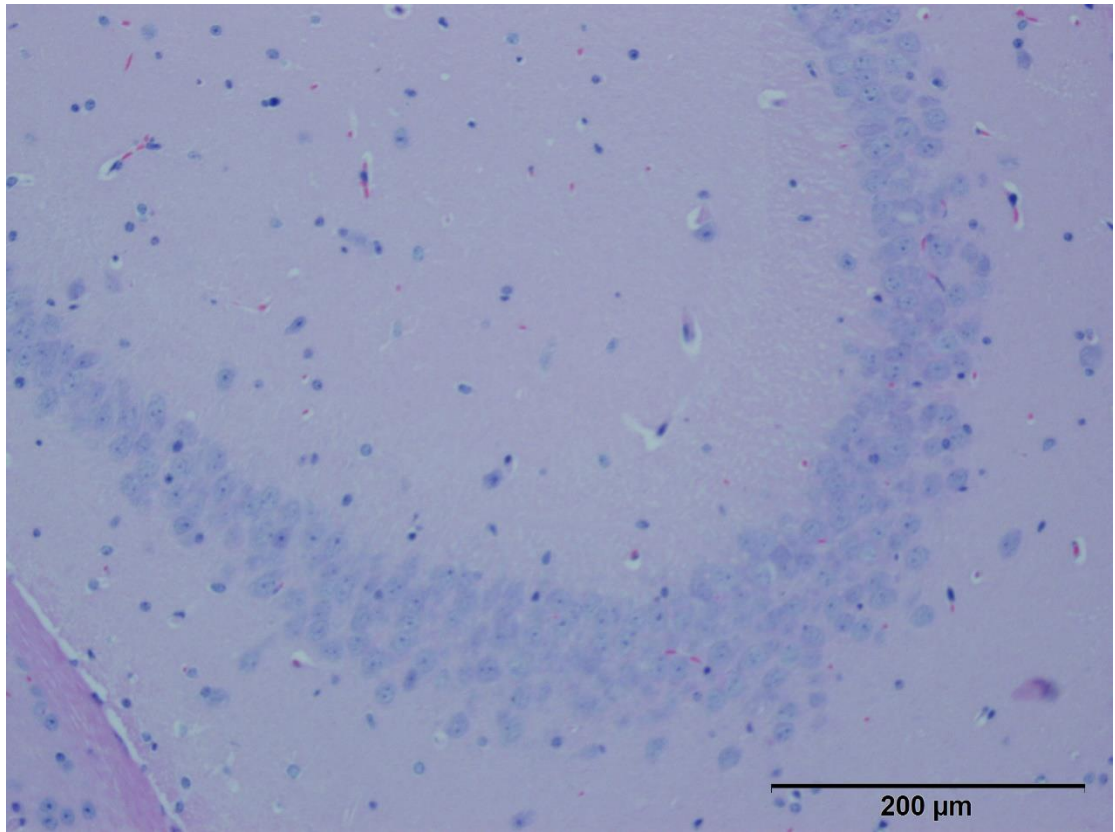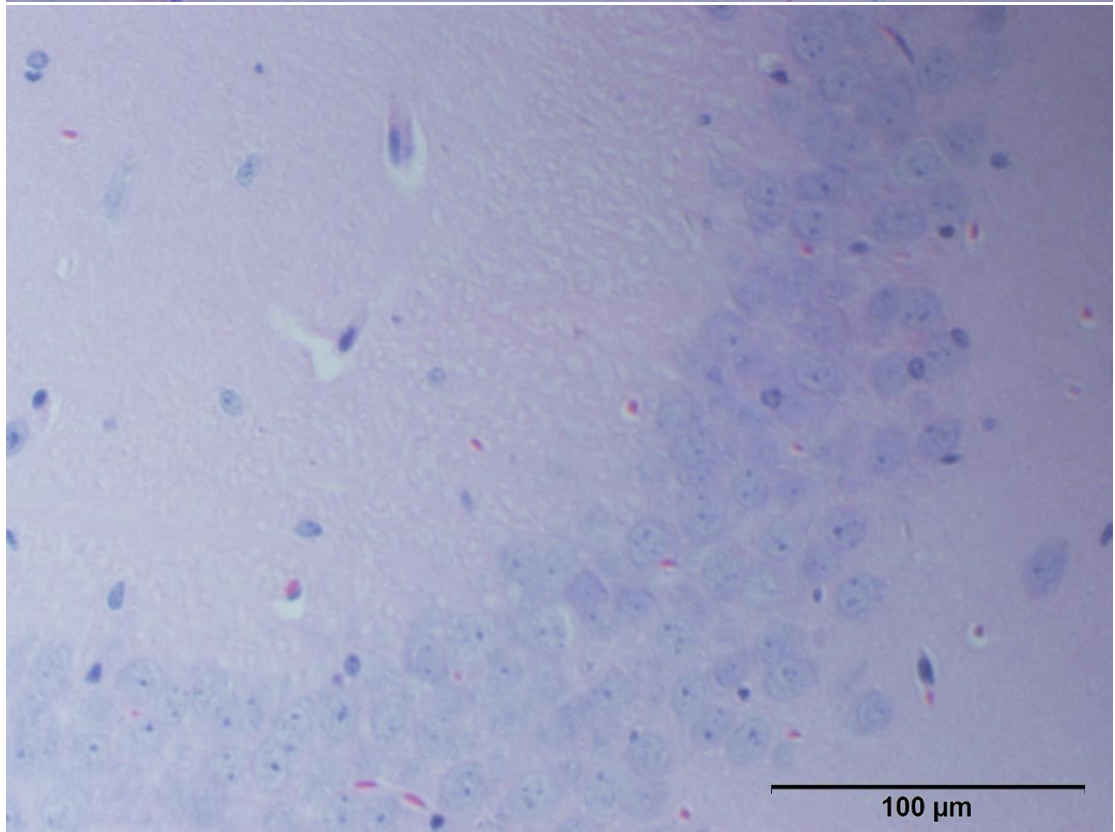

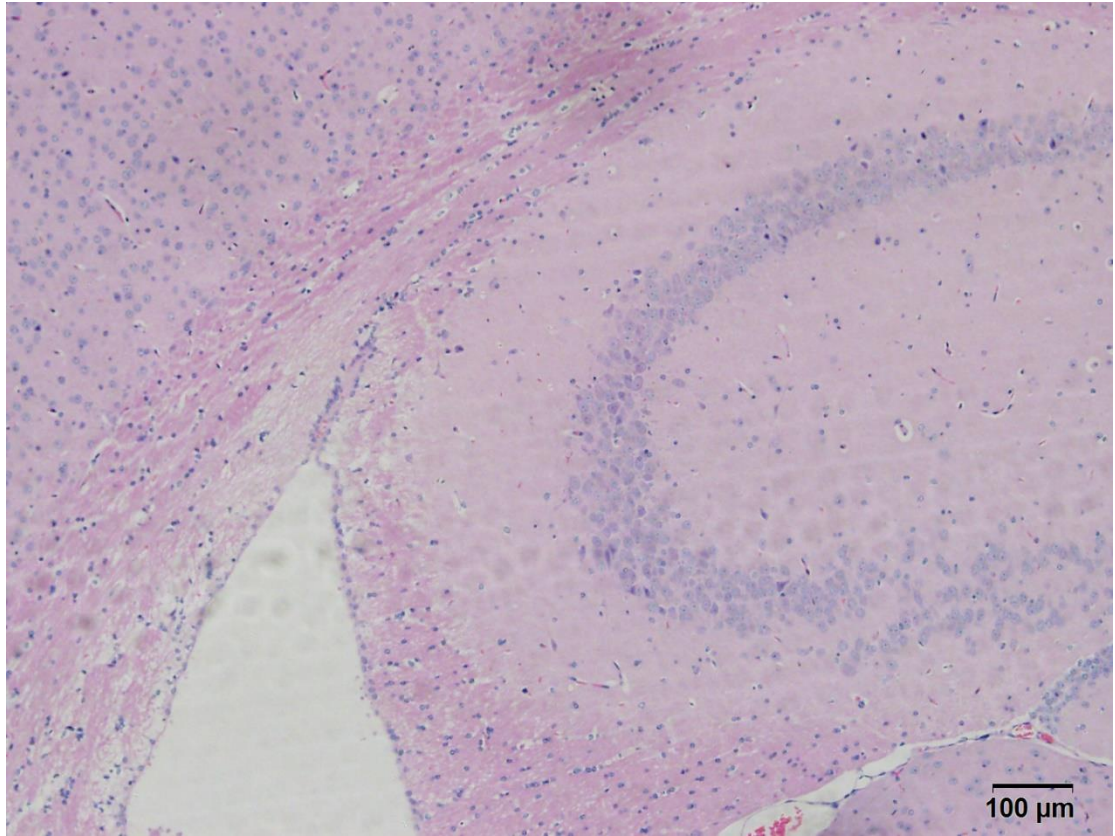

Supplement: Supplementary file 1 [file DataSheet2.PDF]

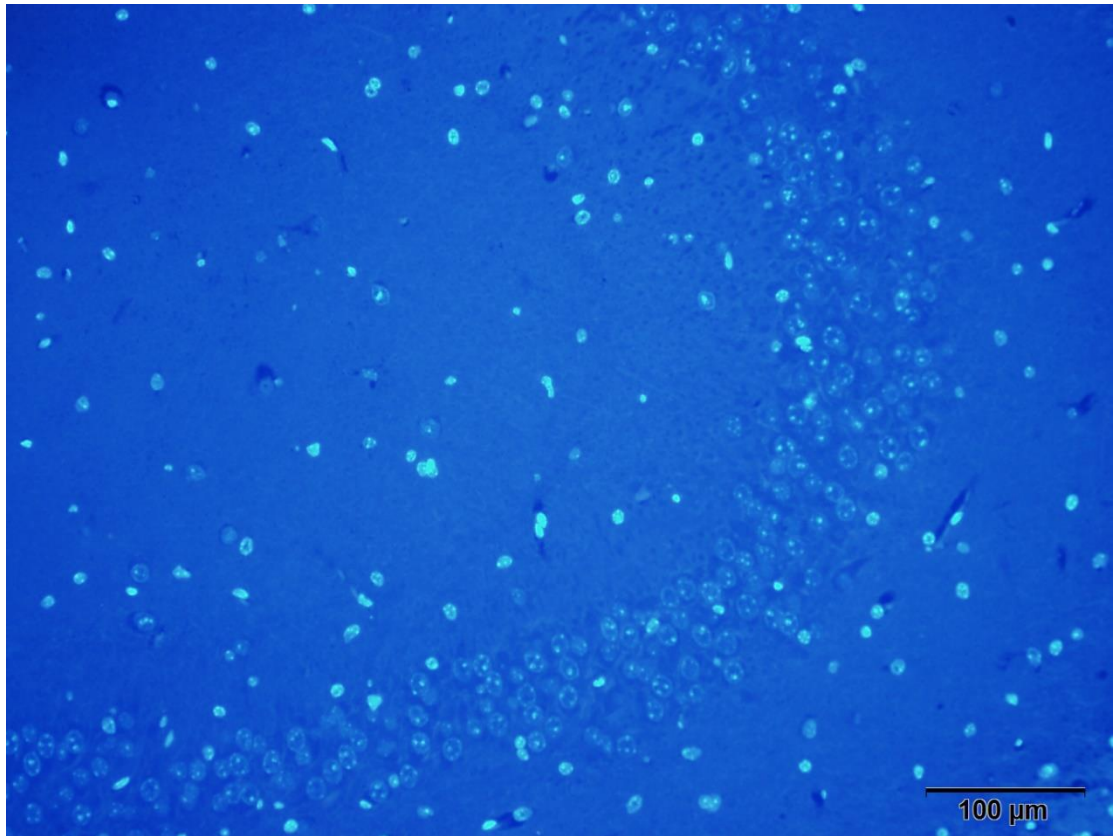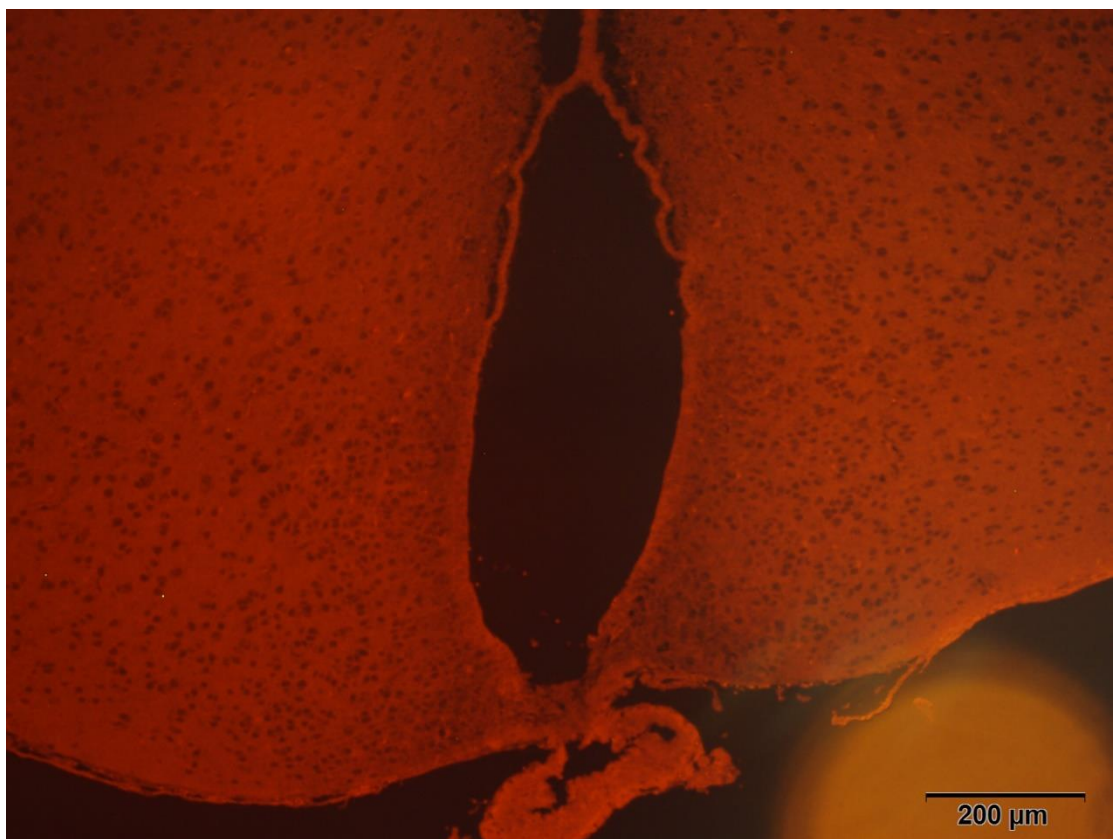

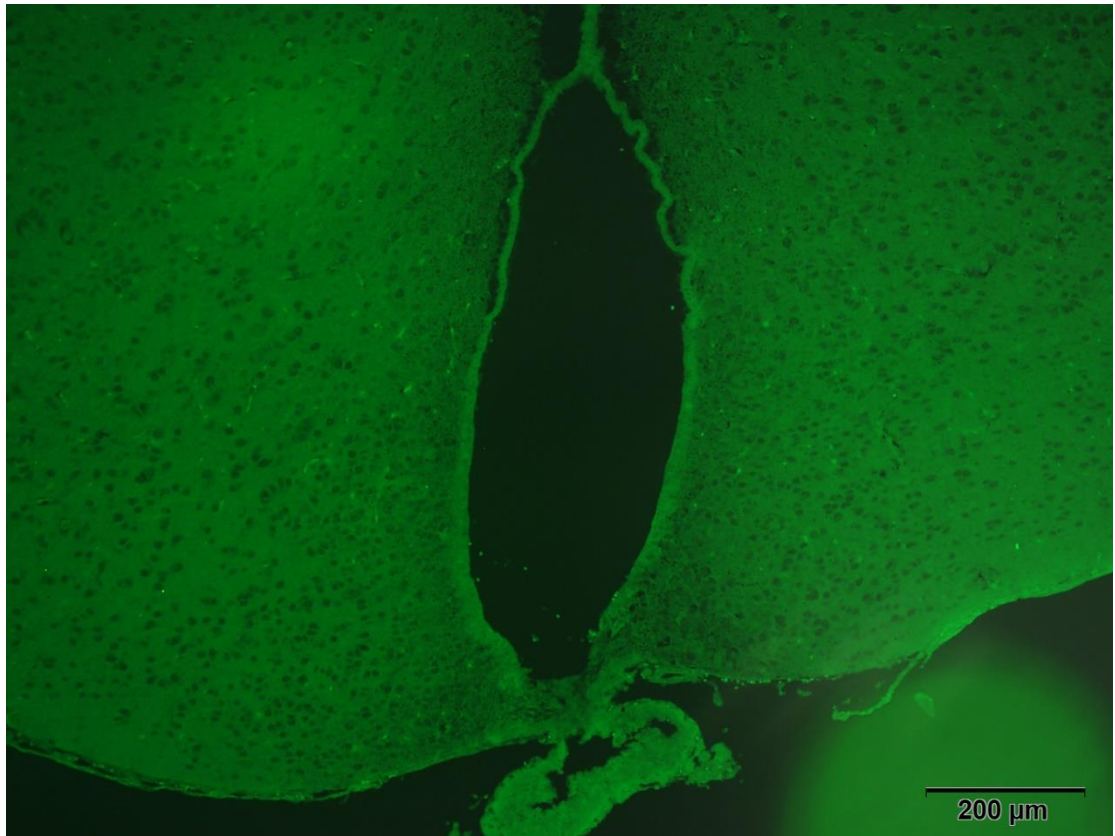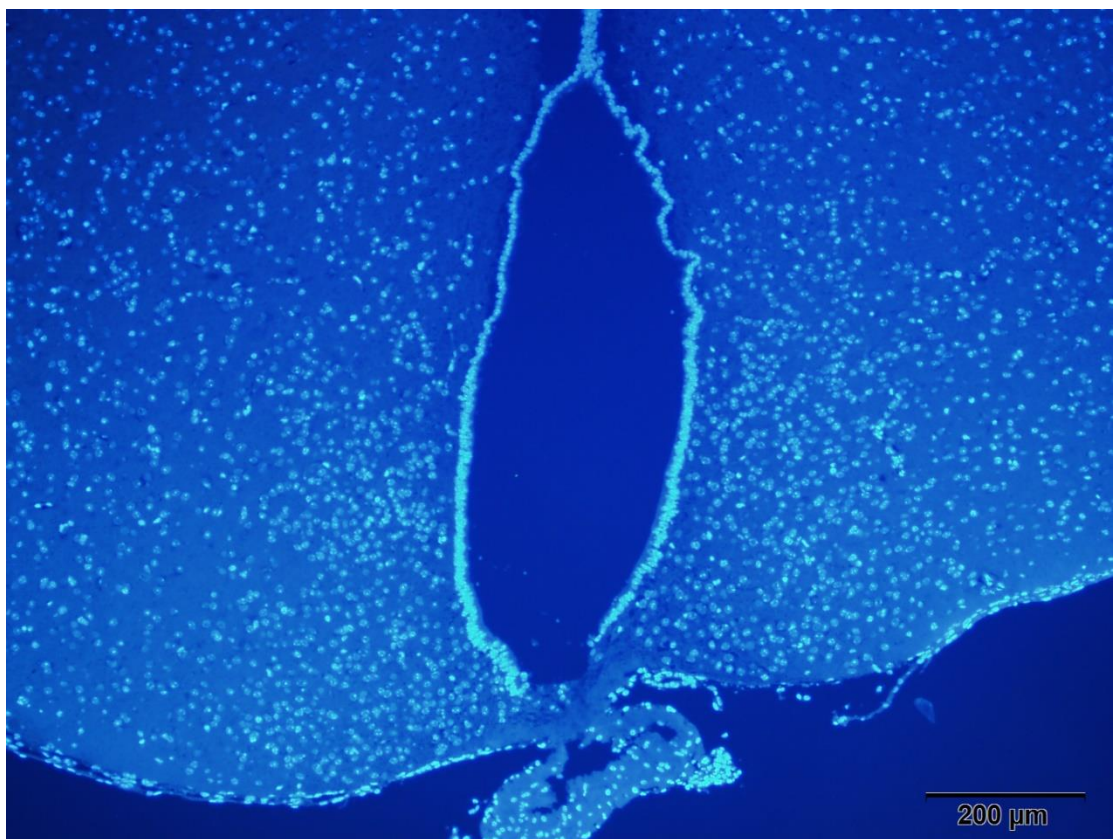

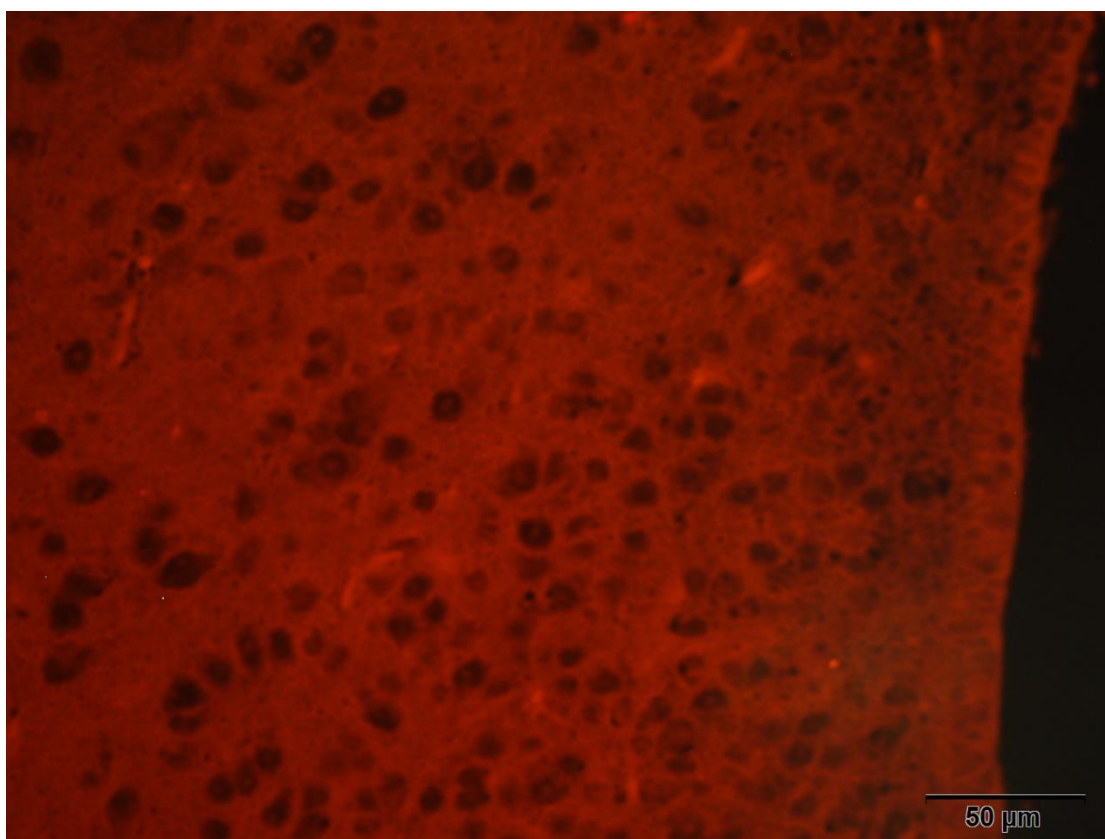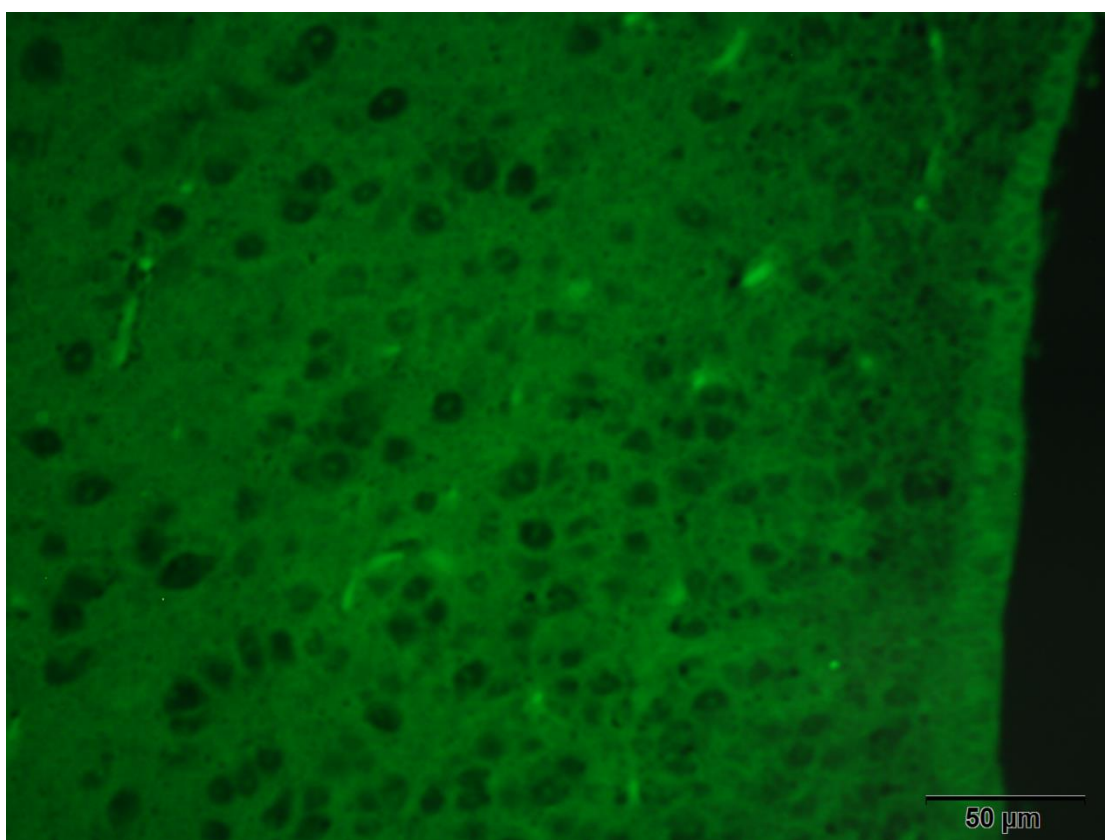

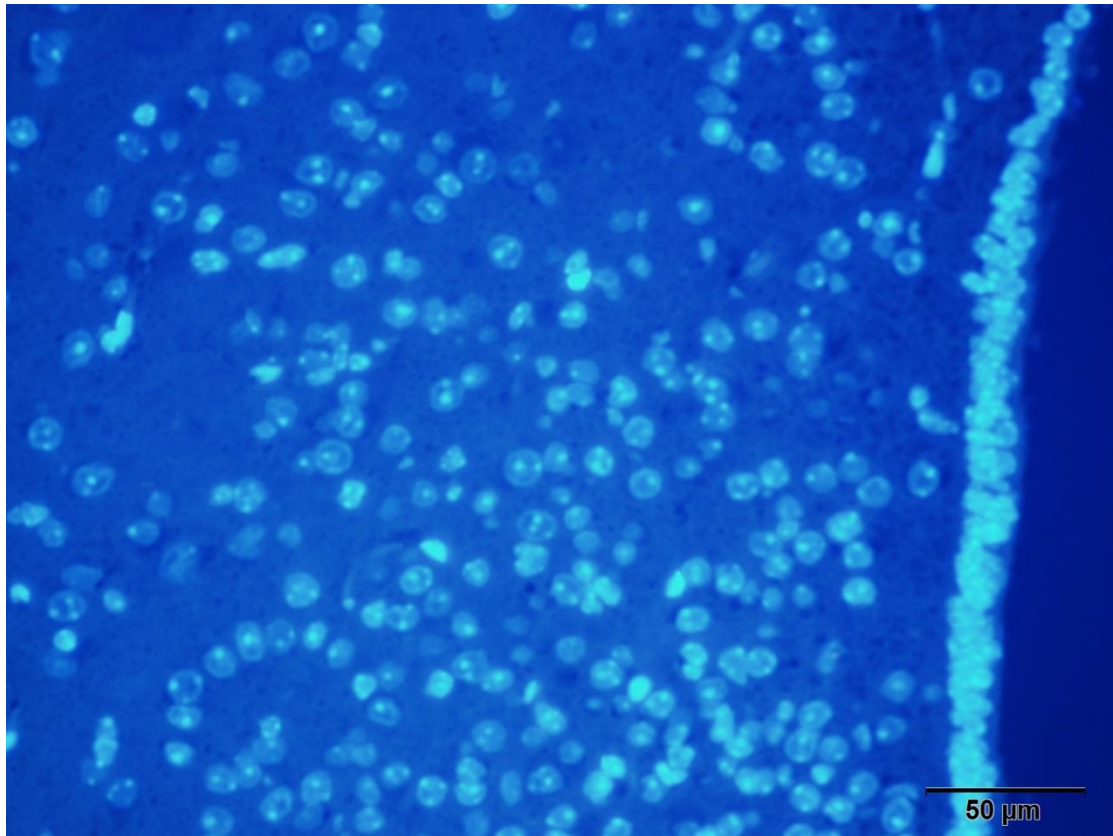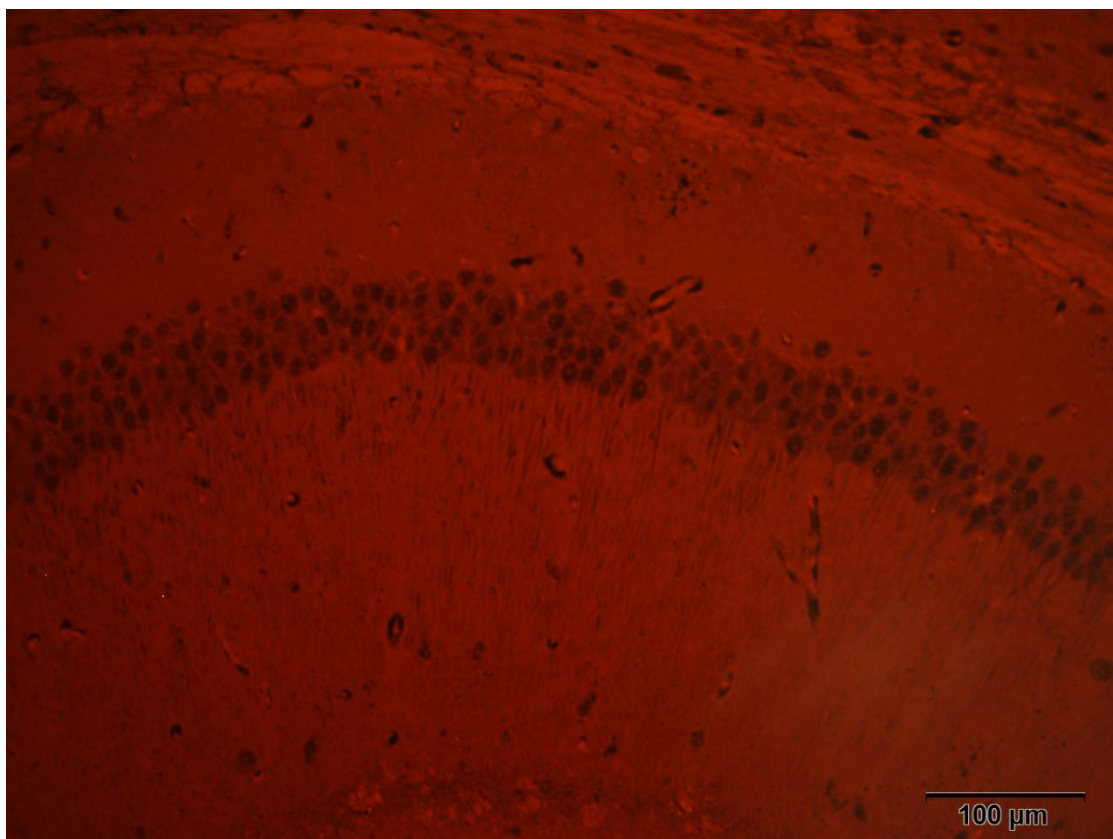

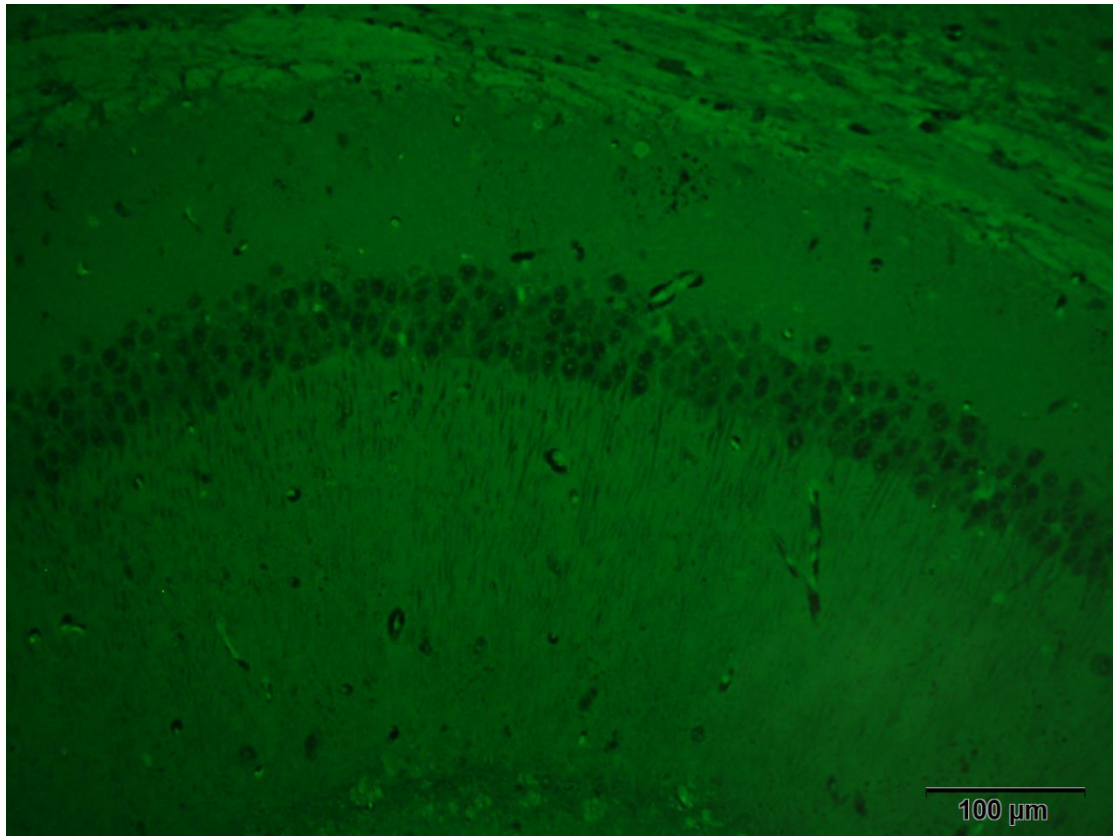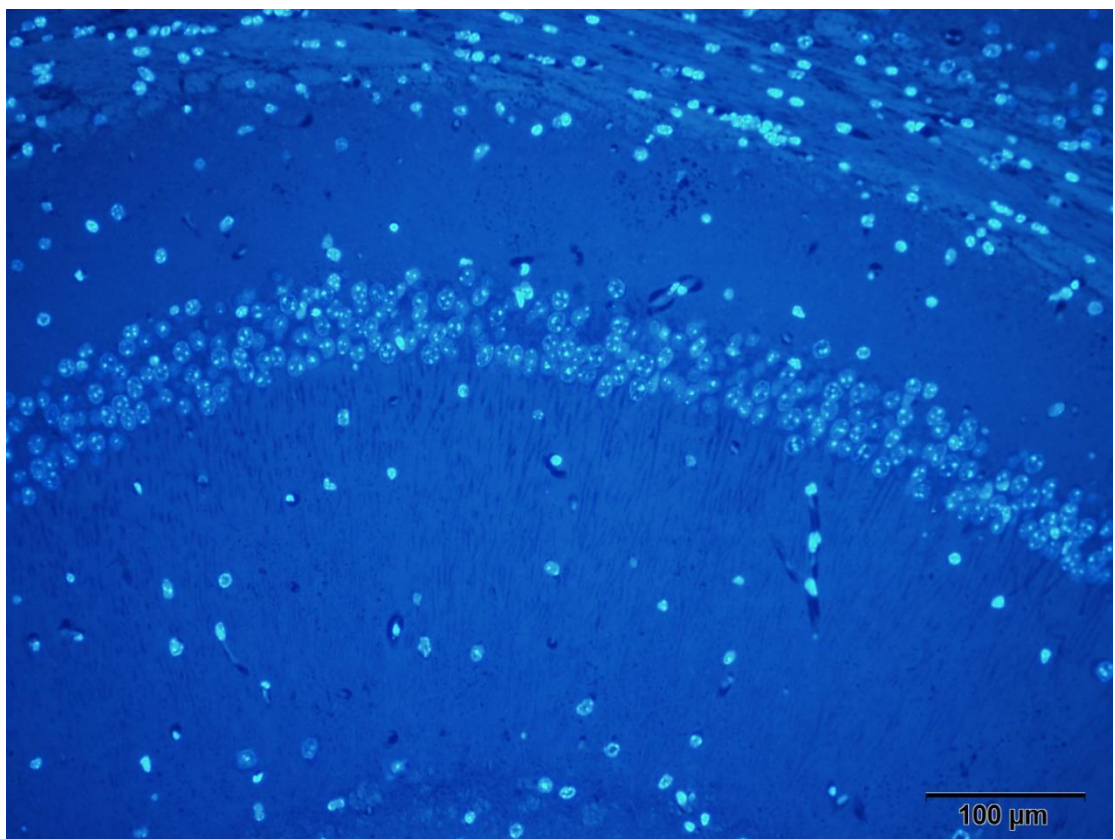

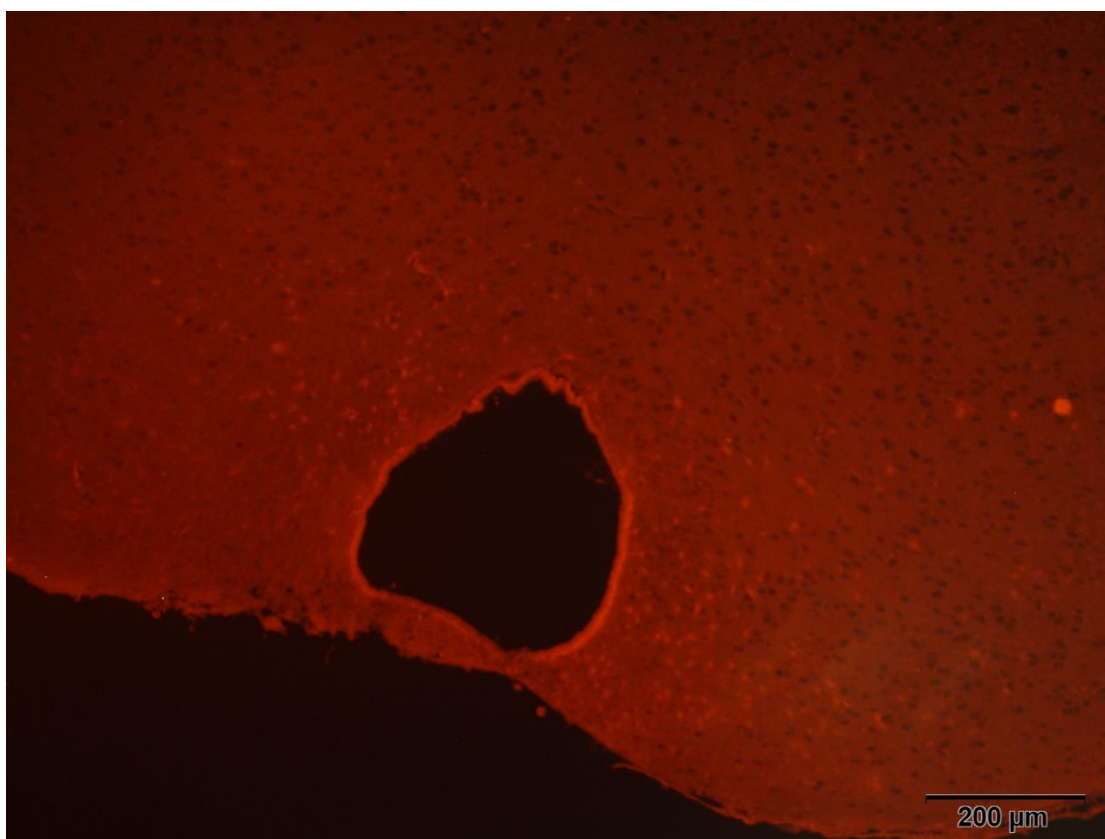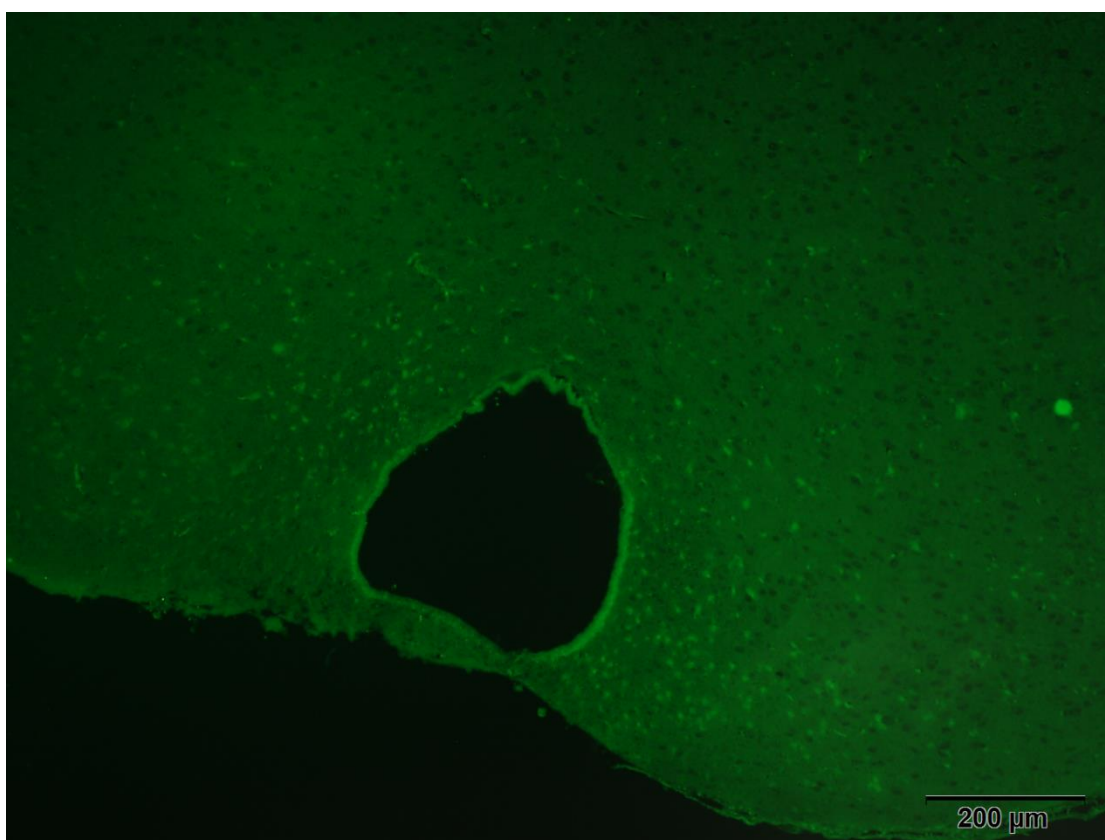

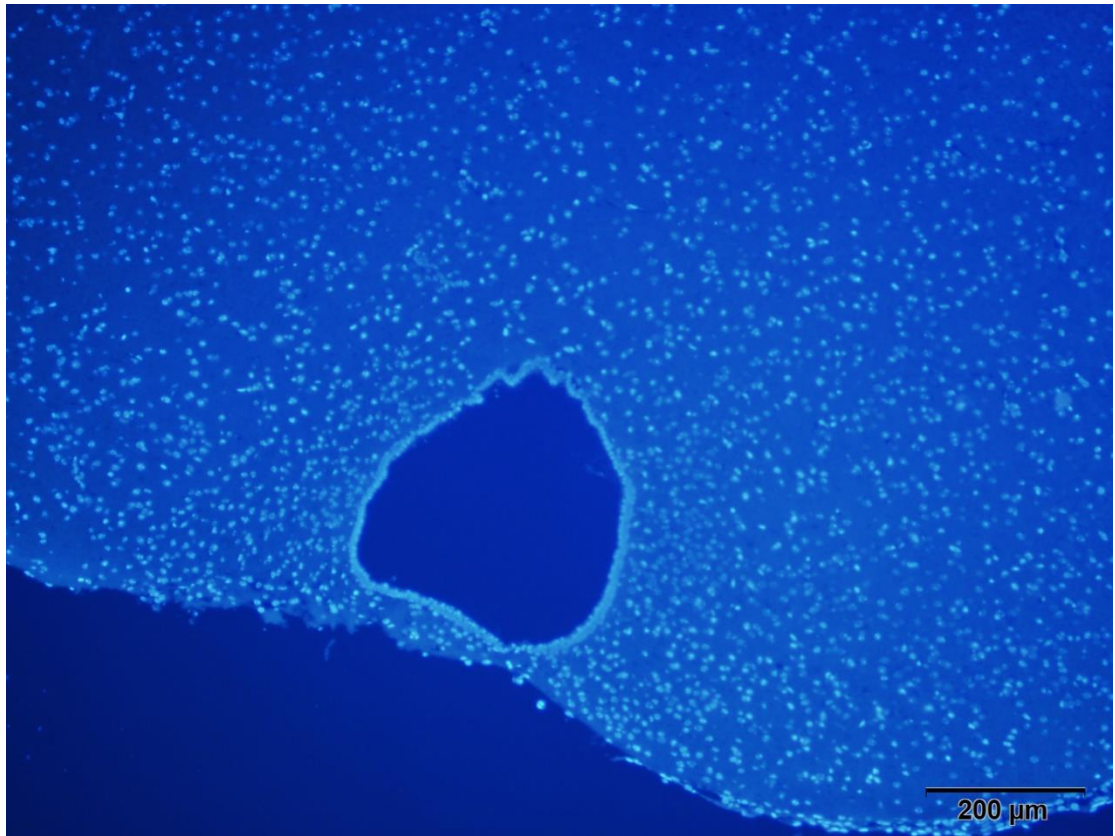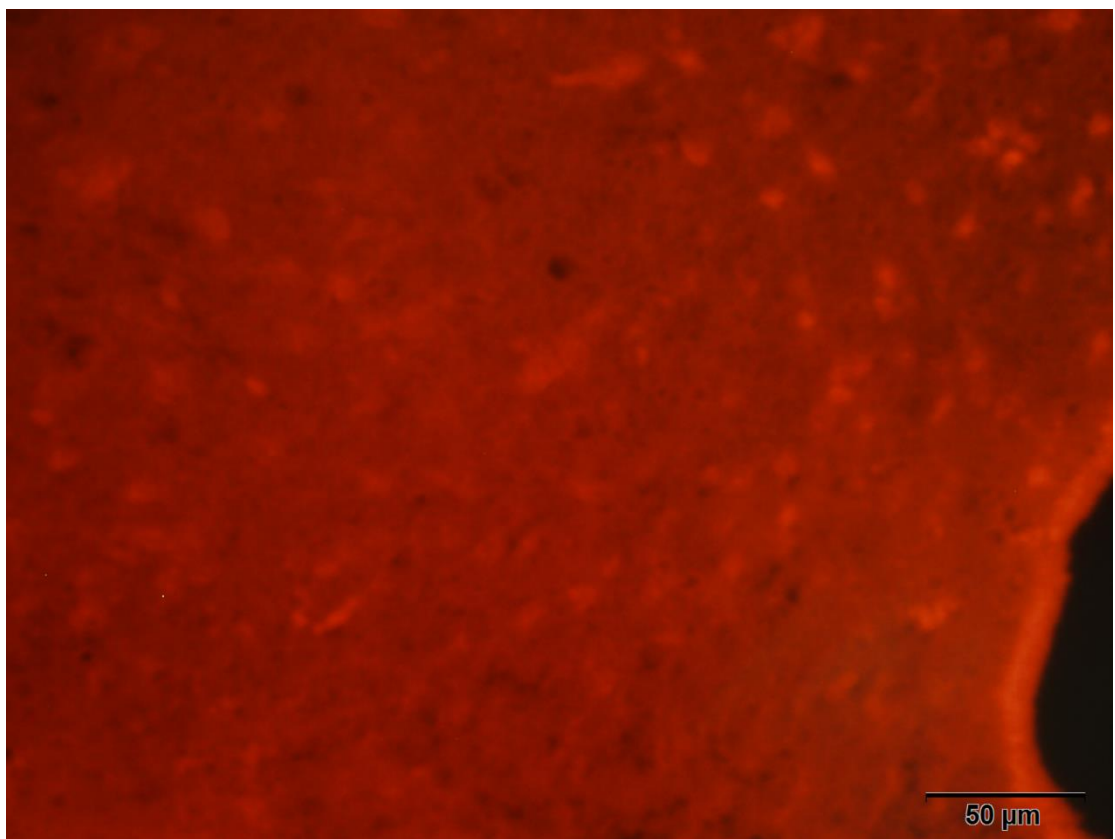

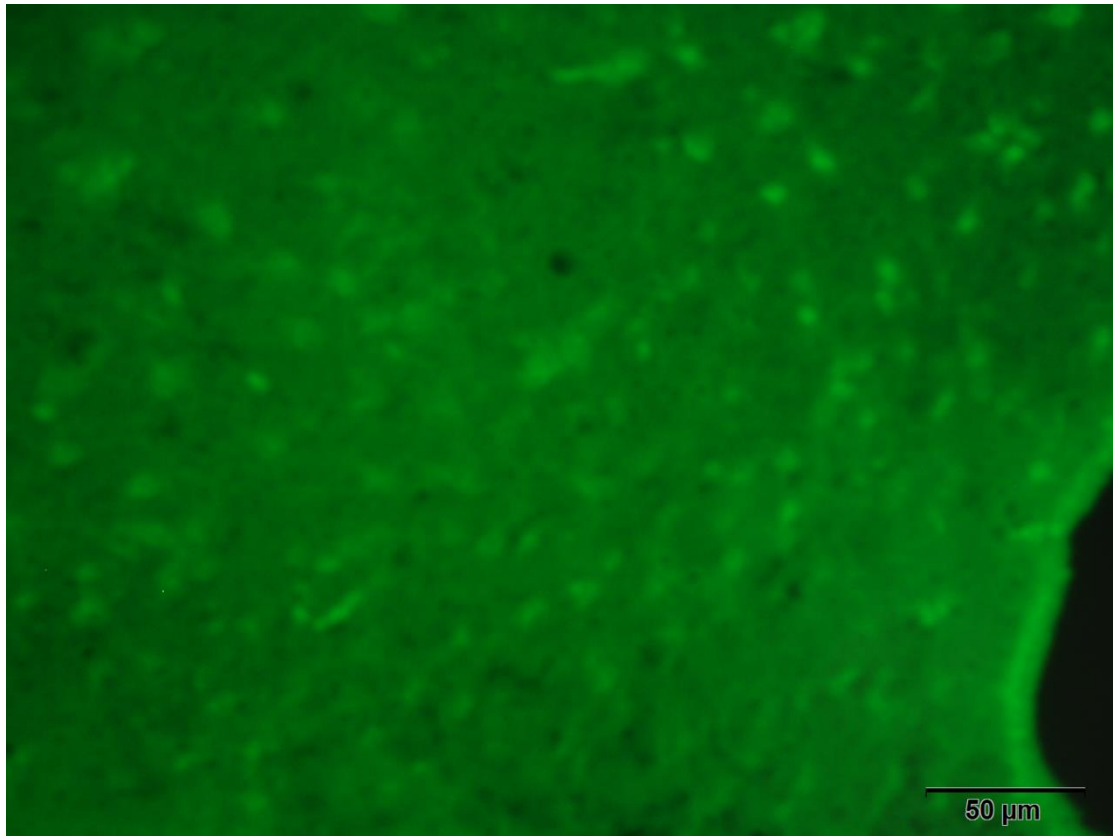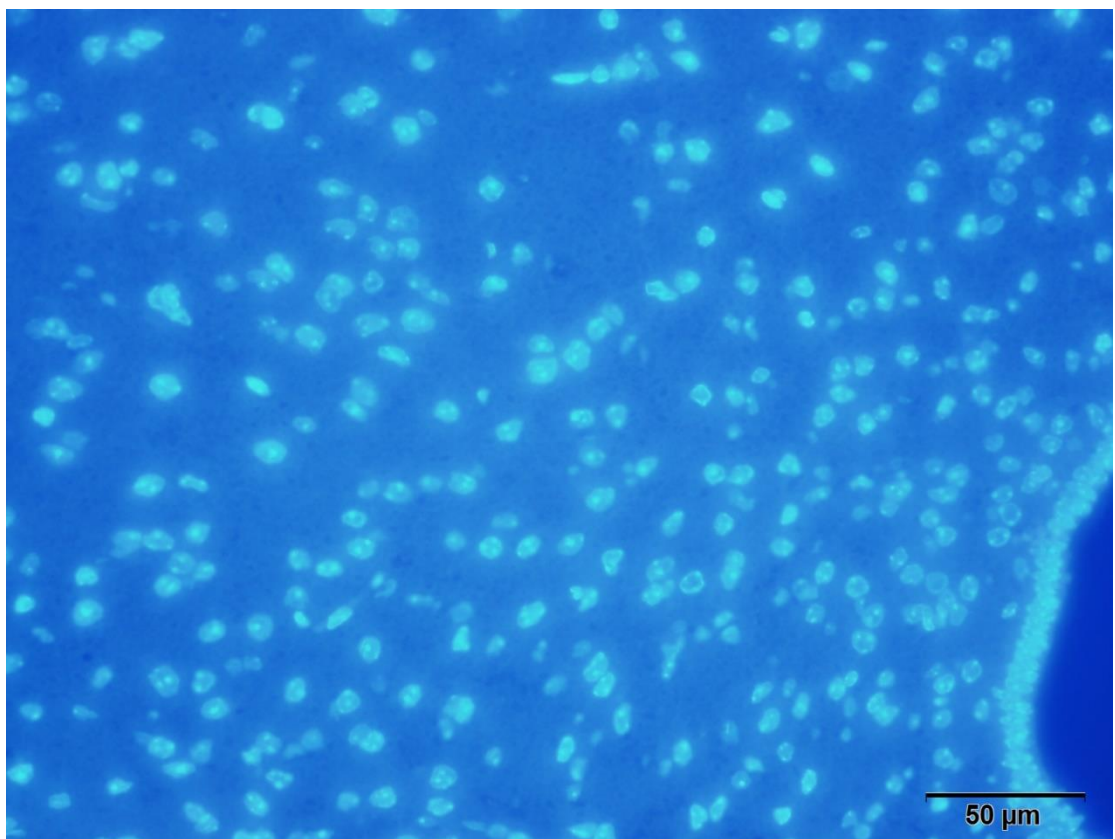

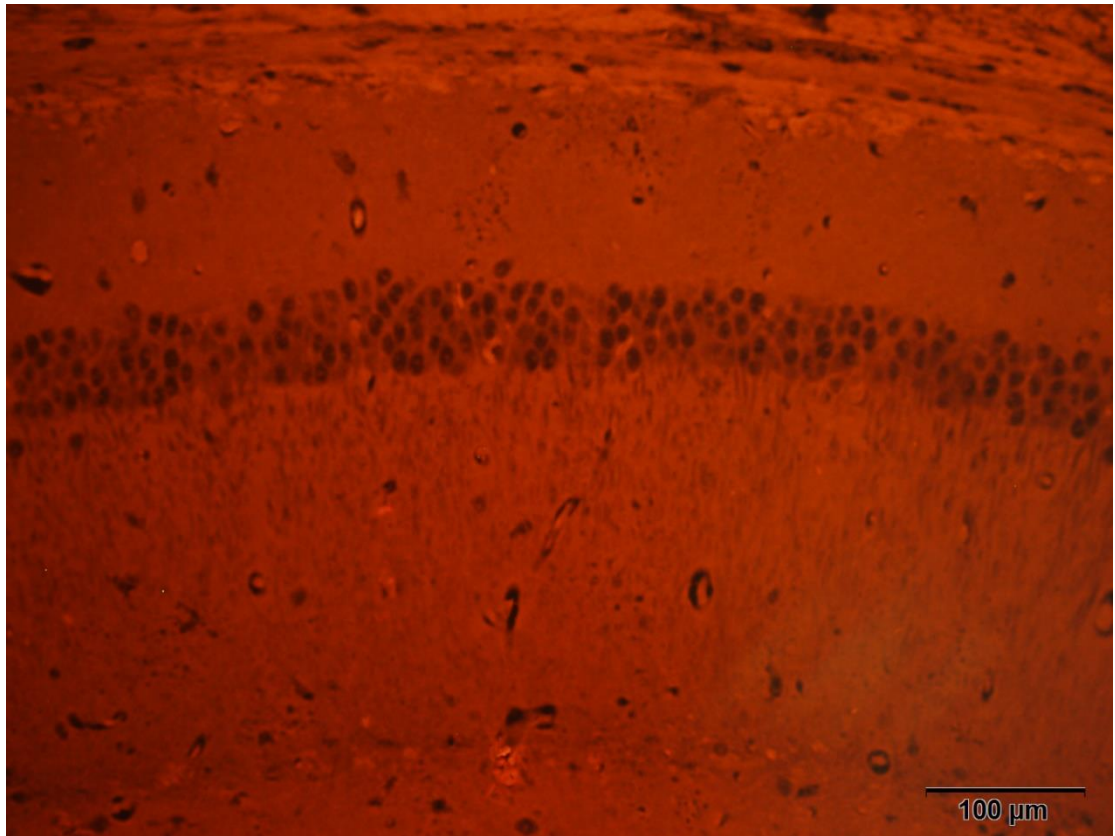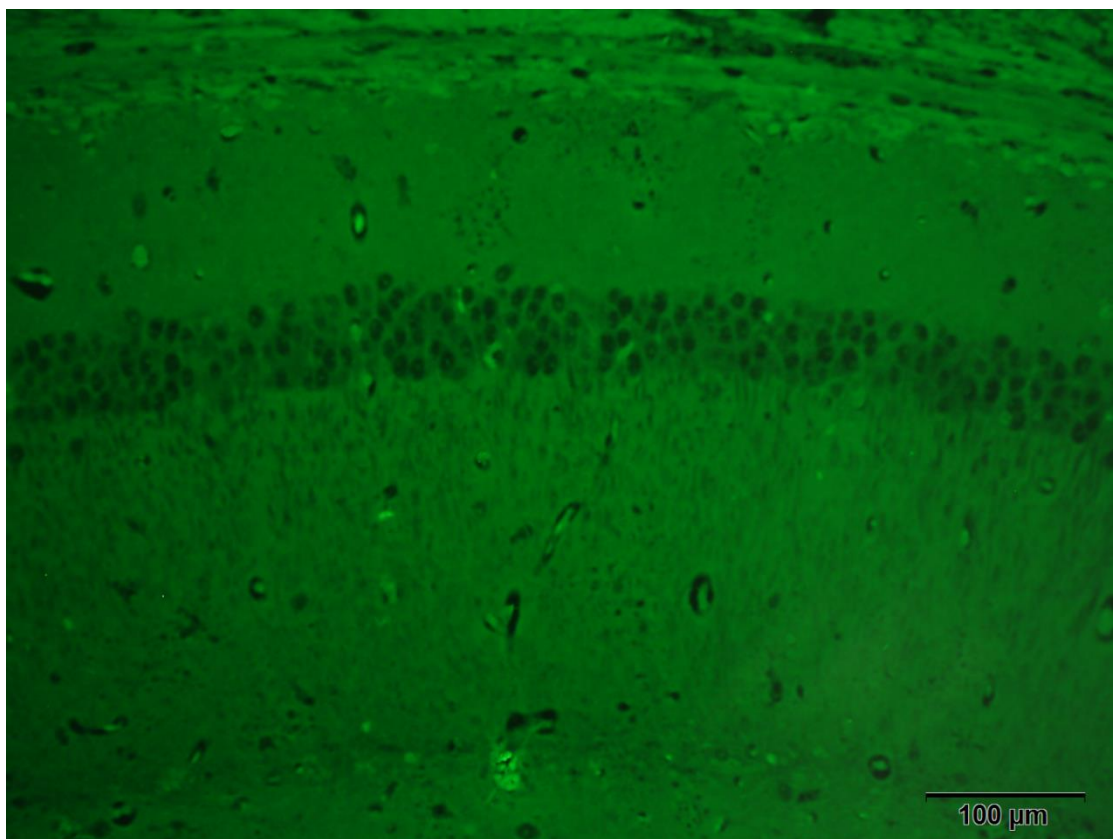

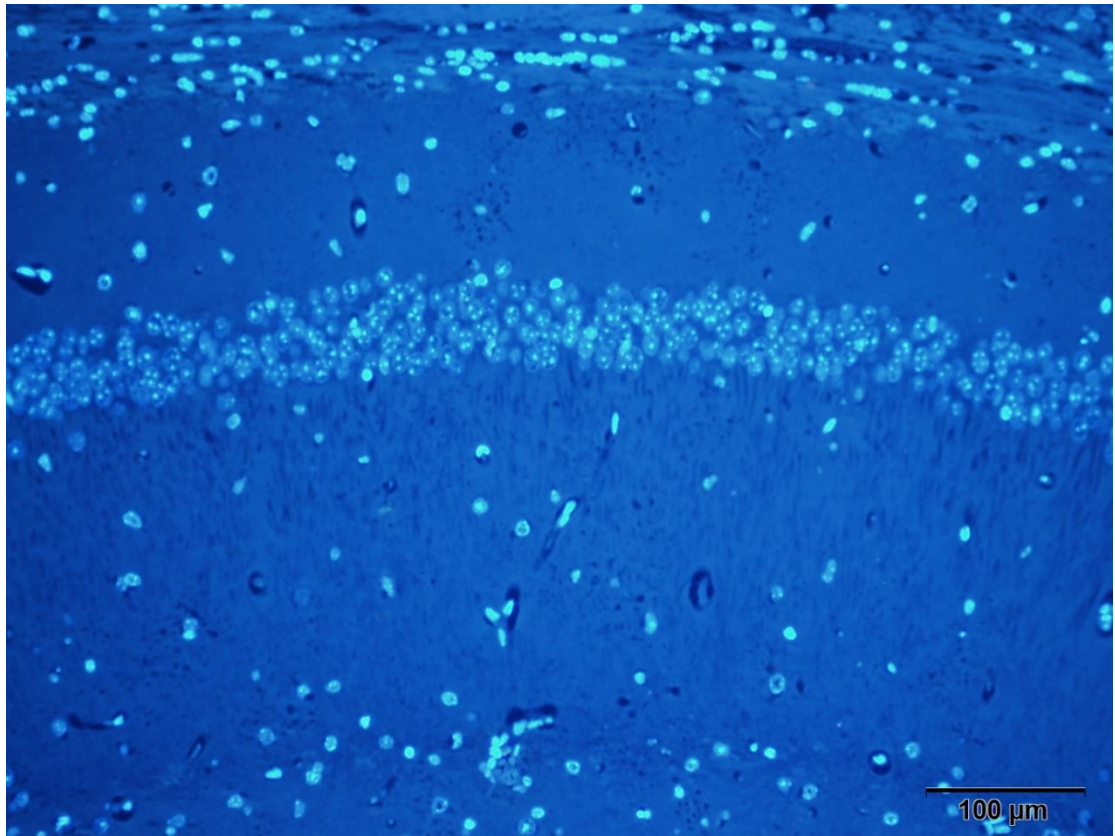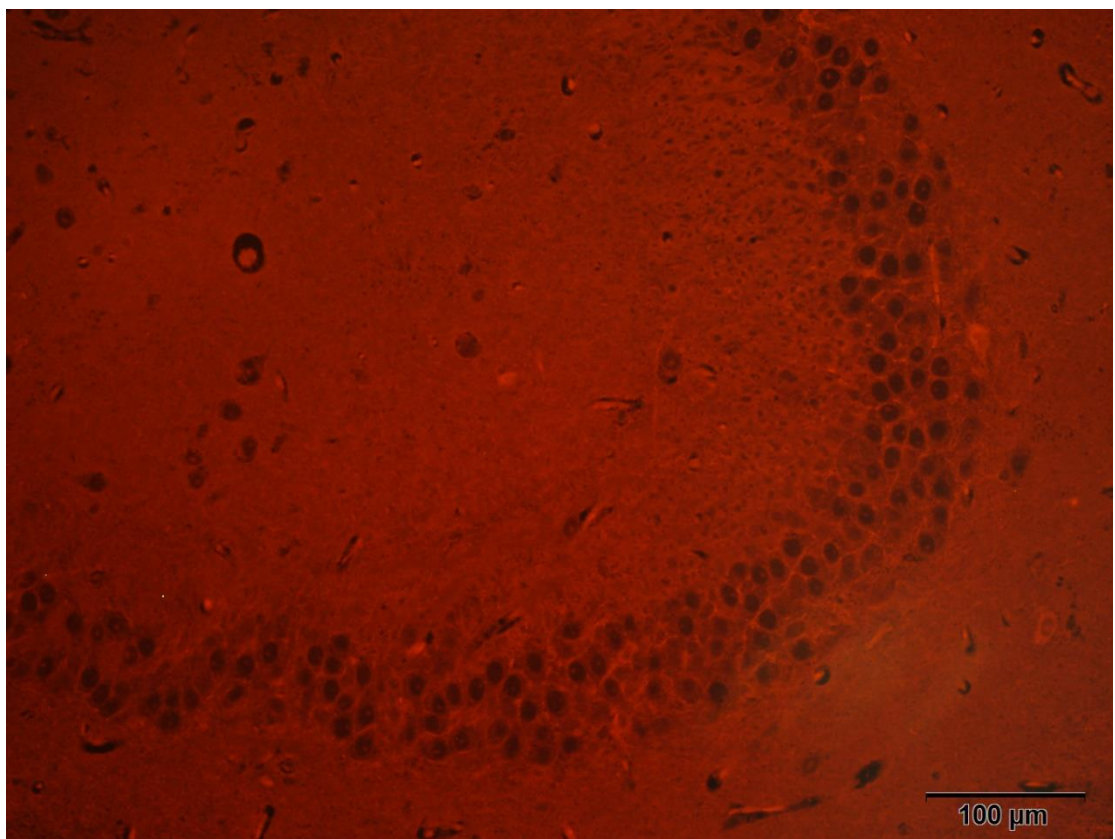

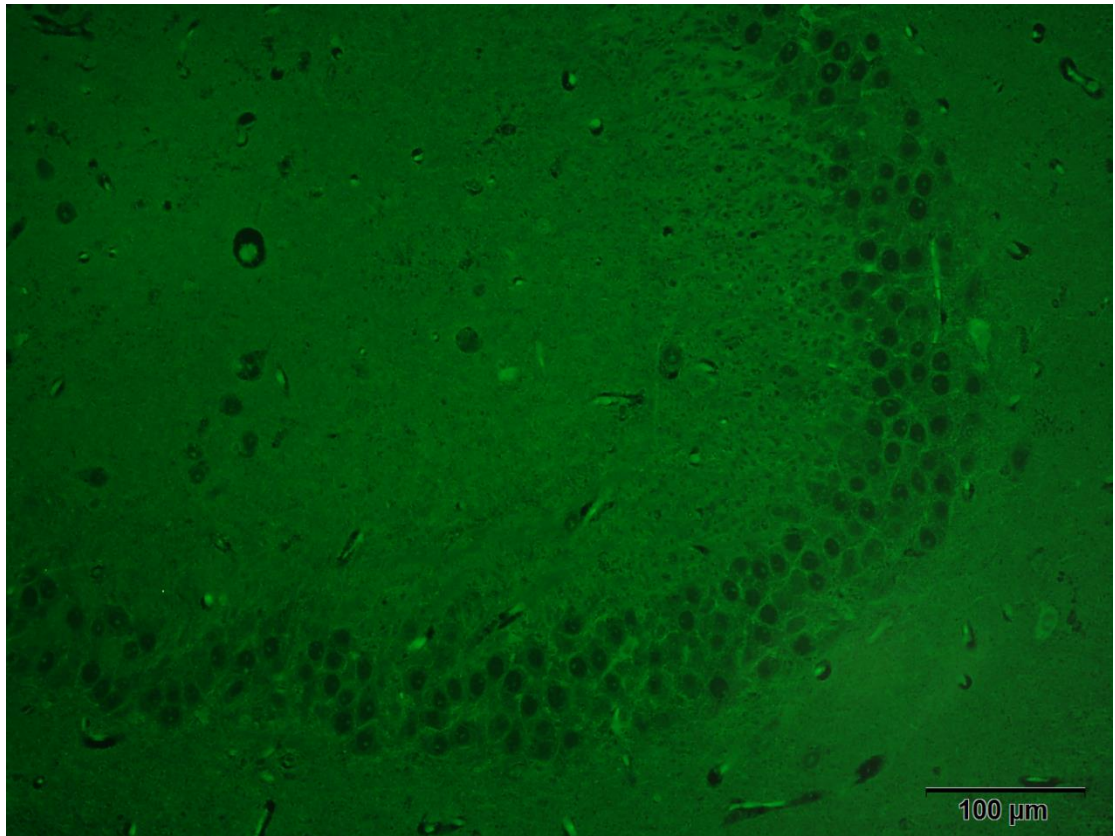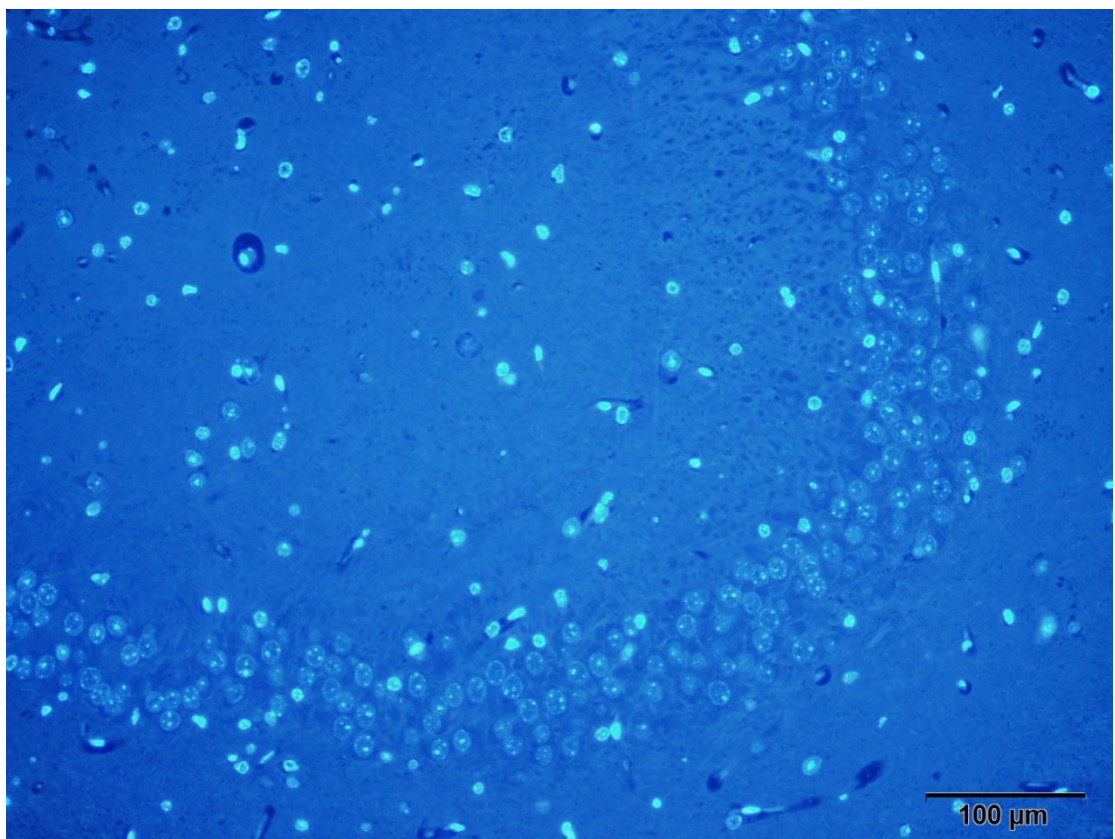

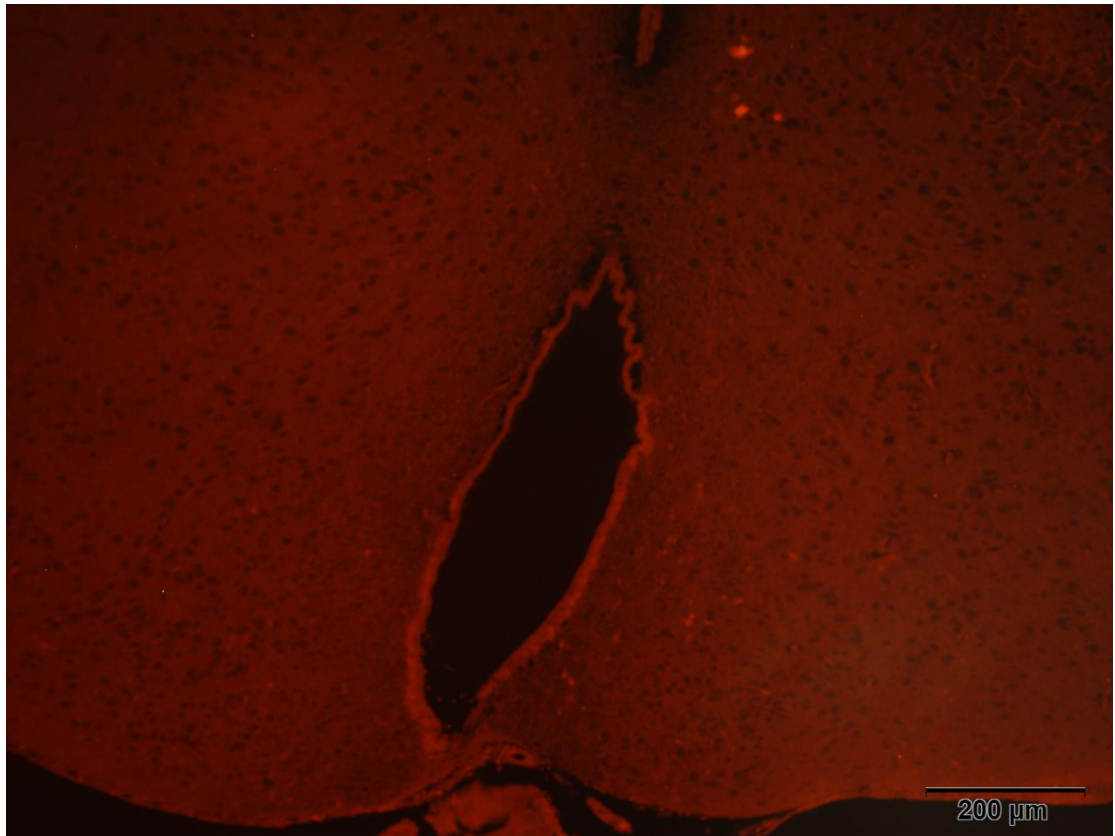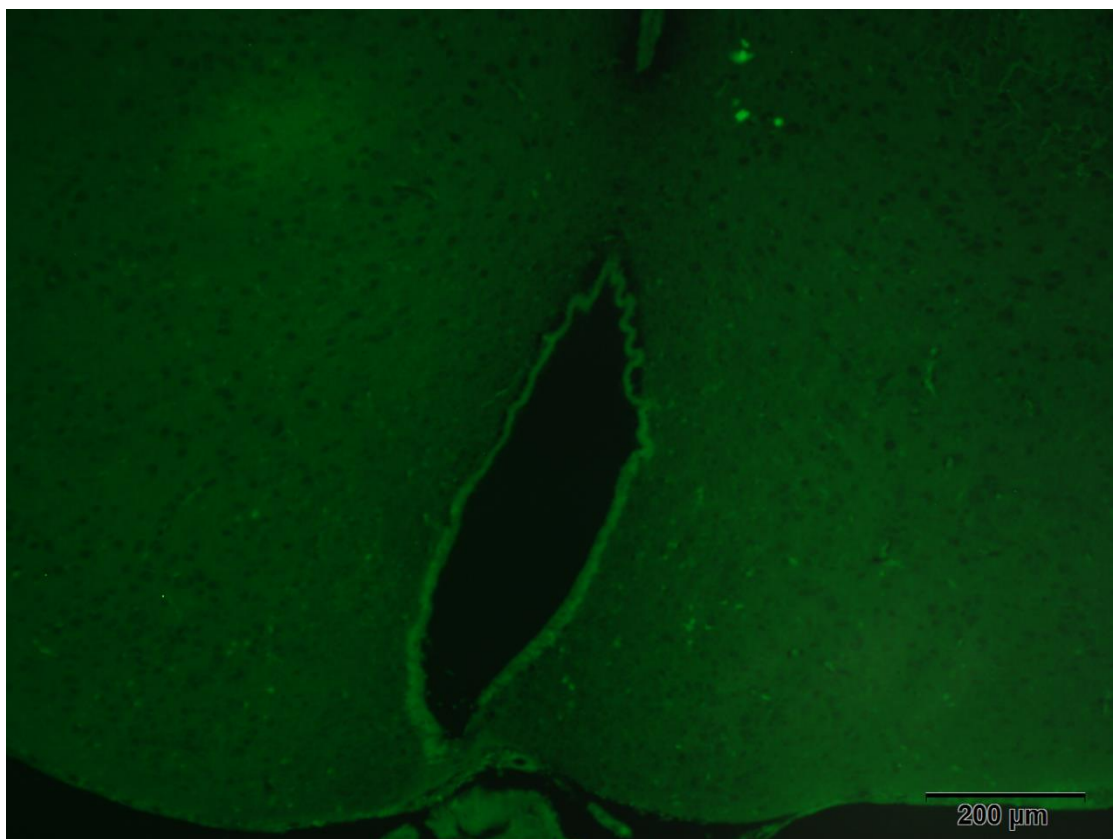

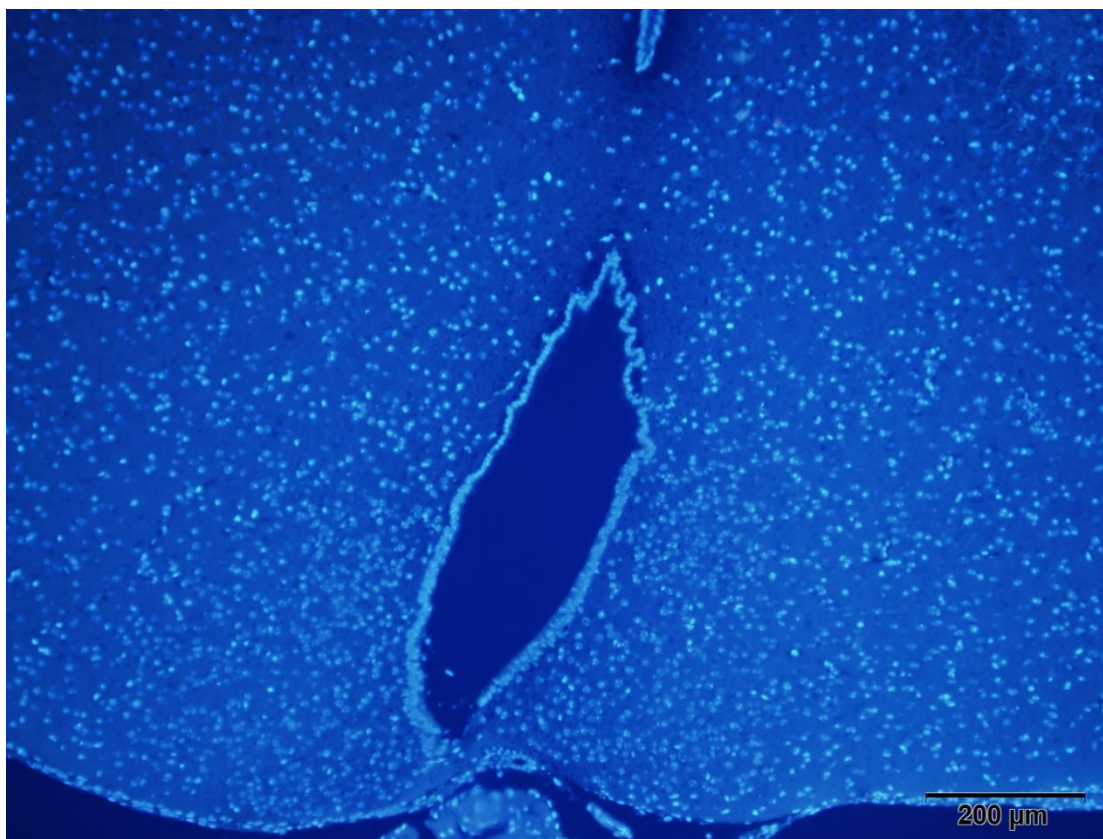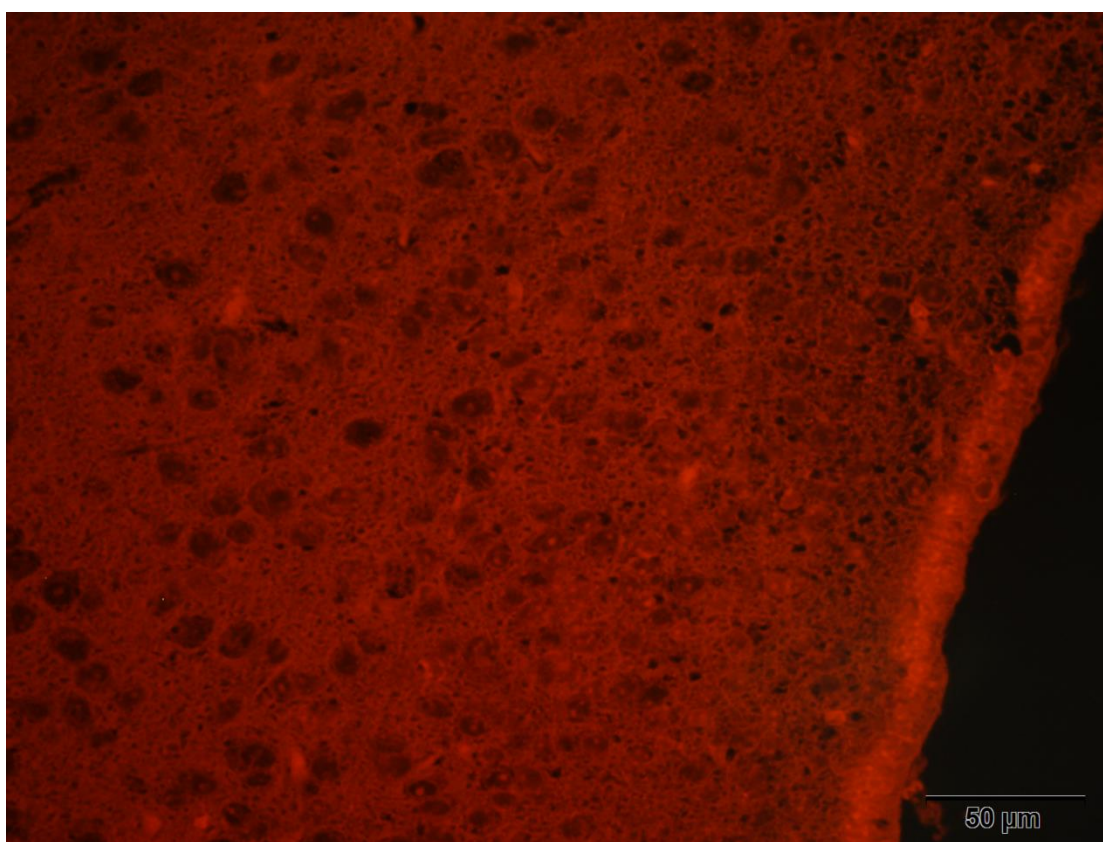

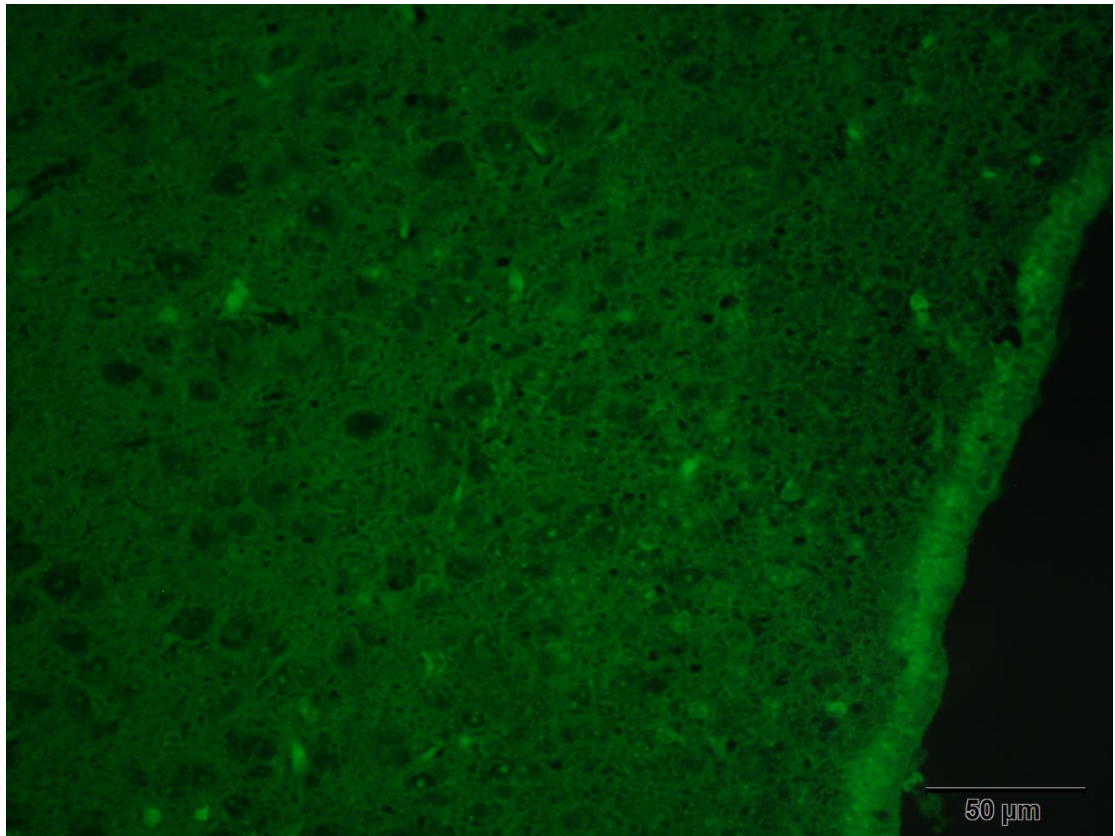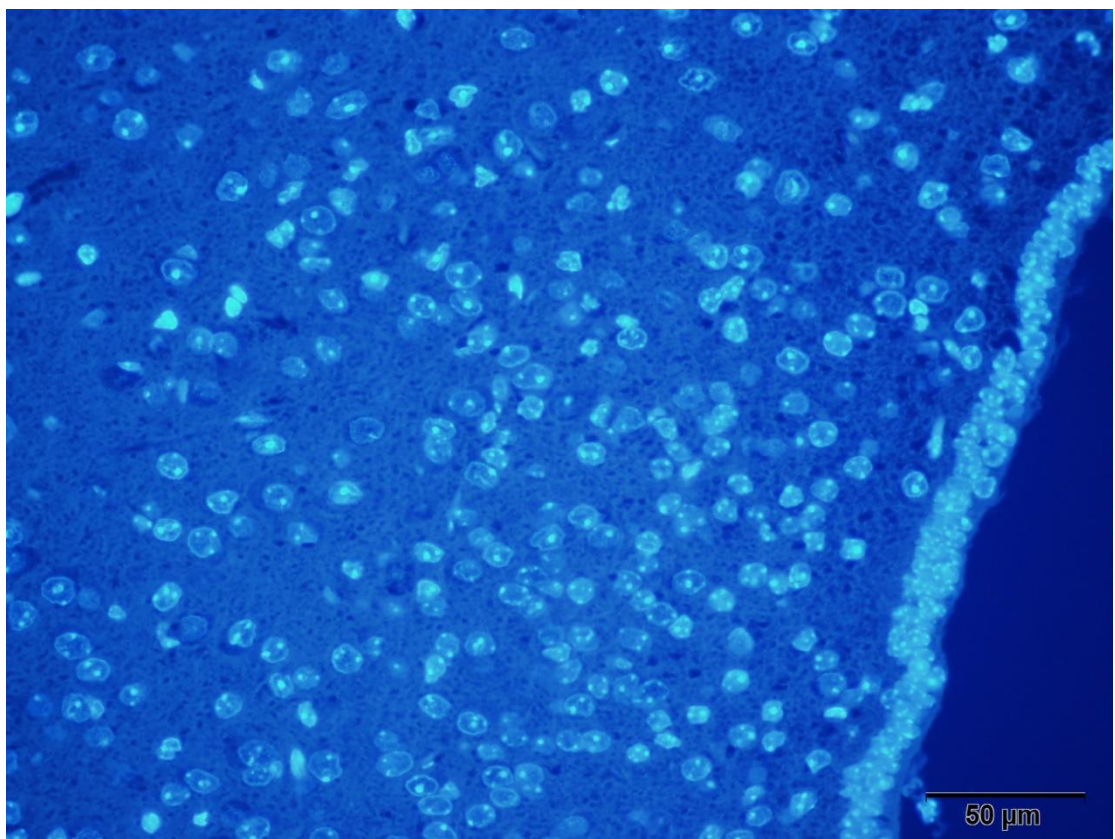

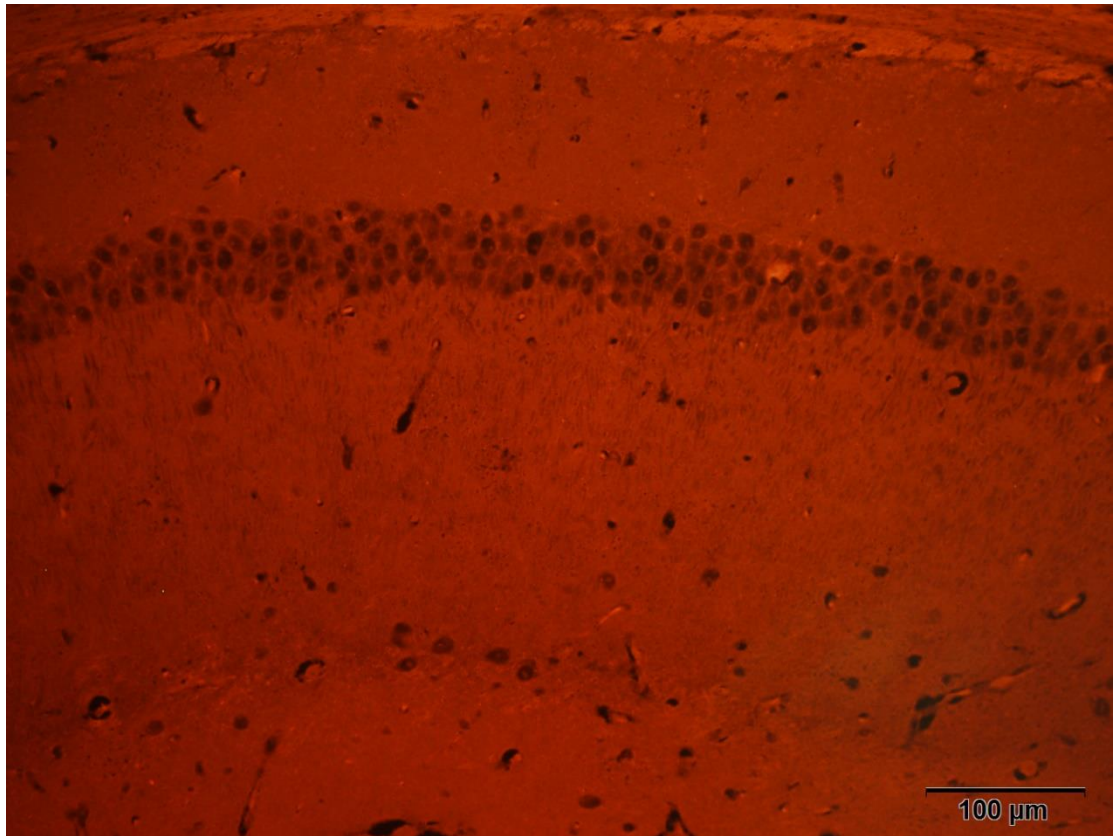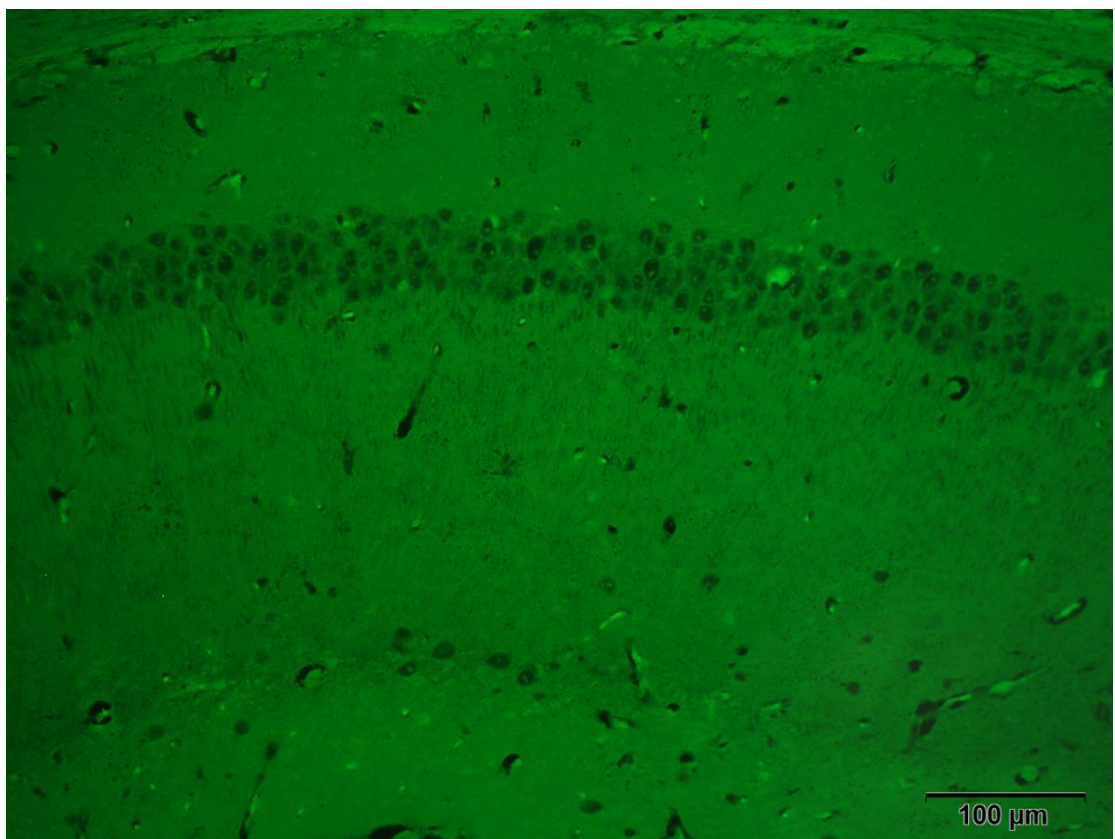

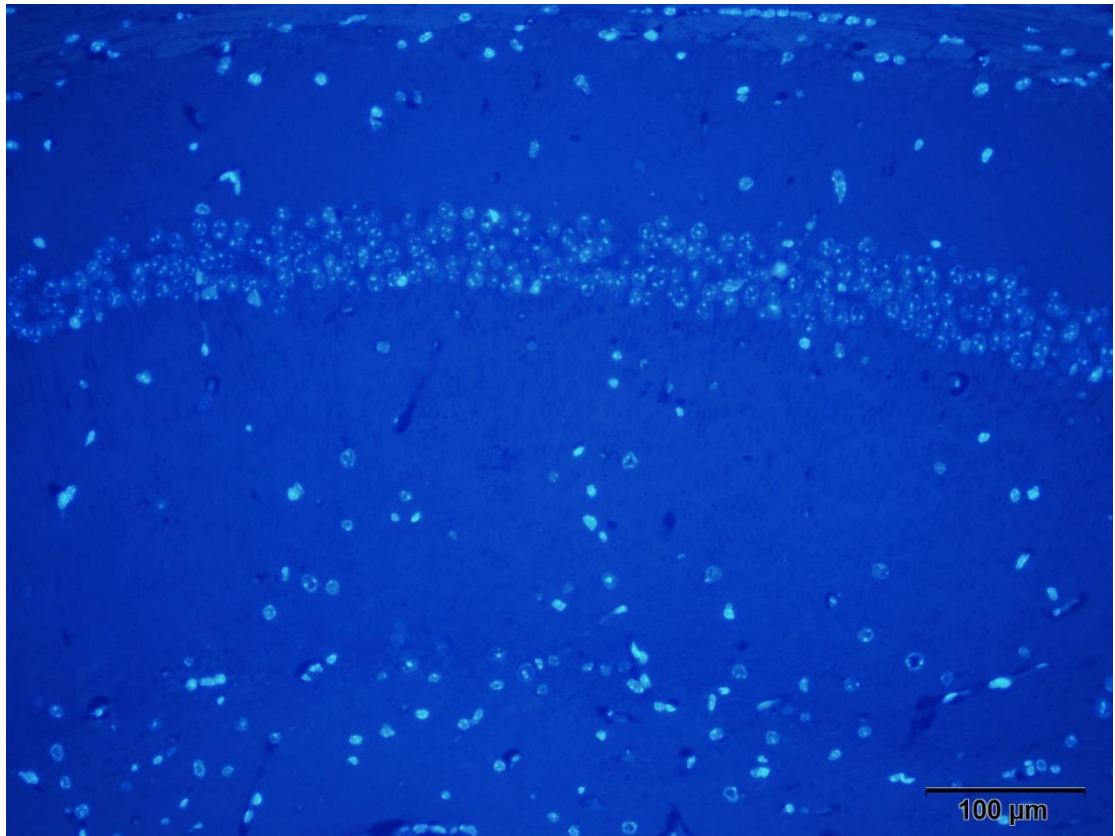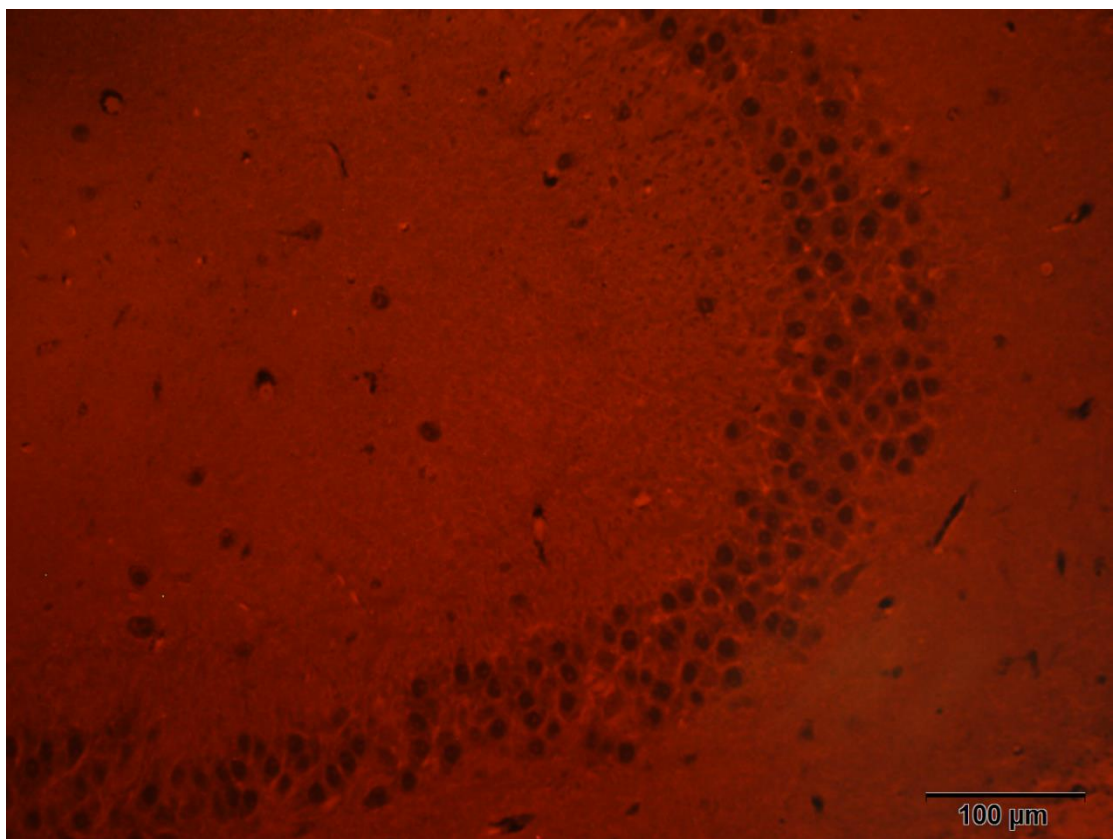

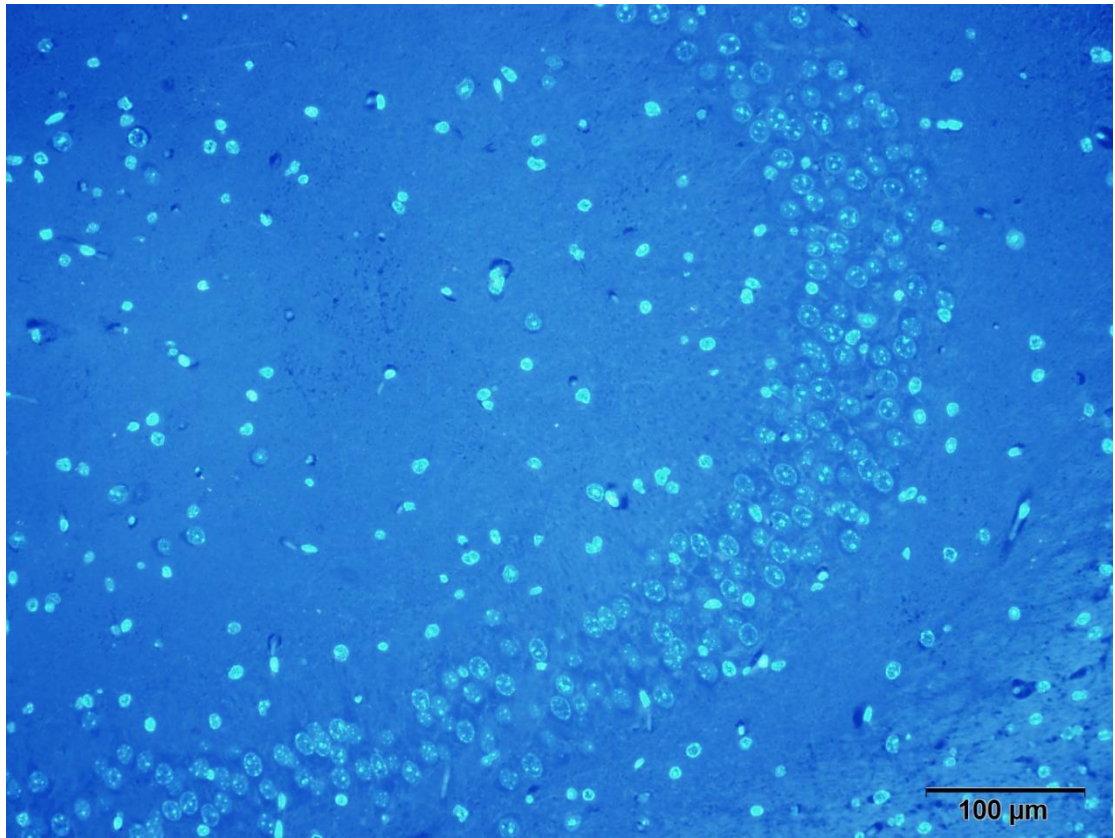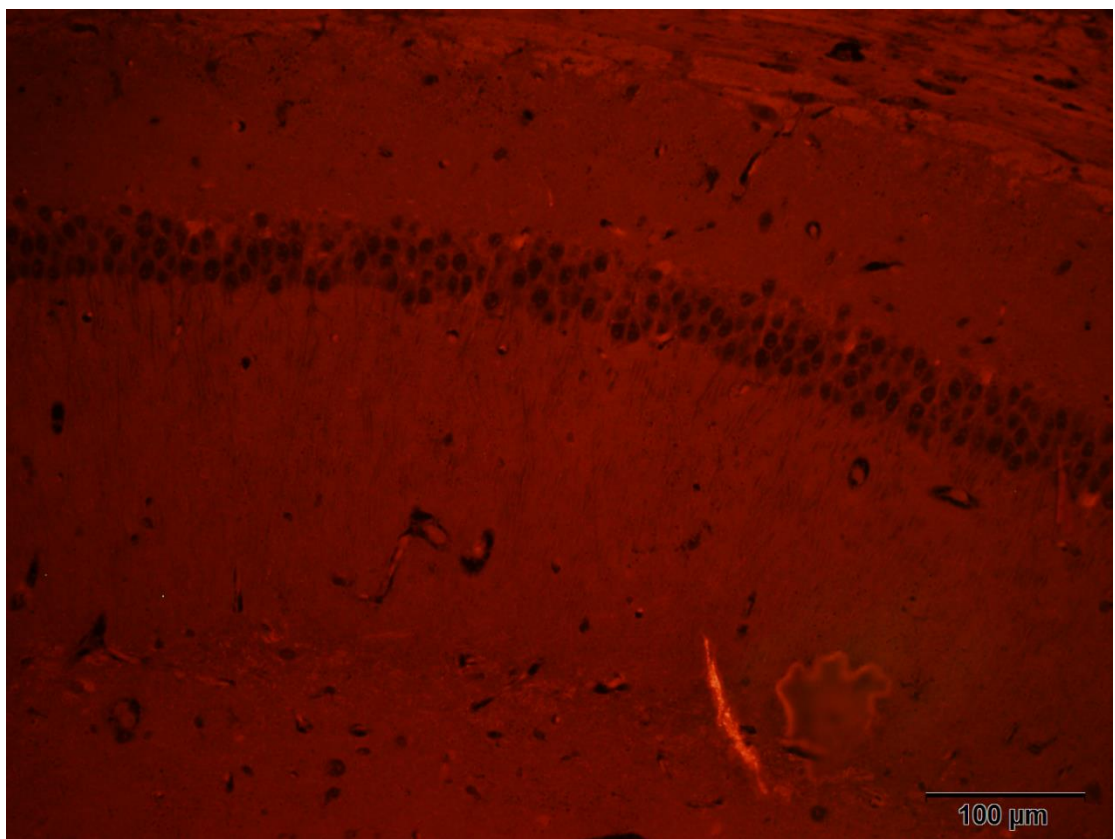

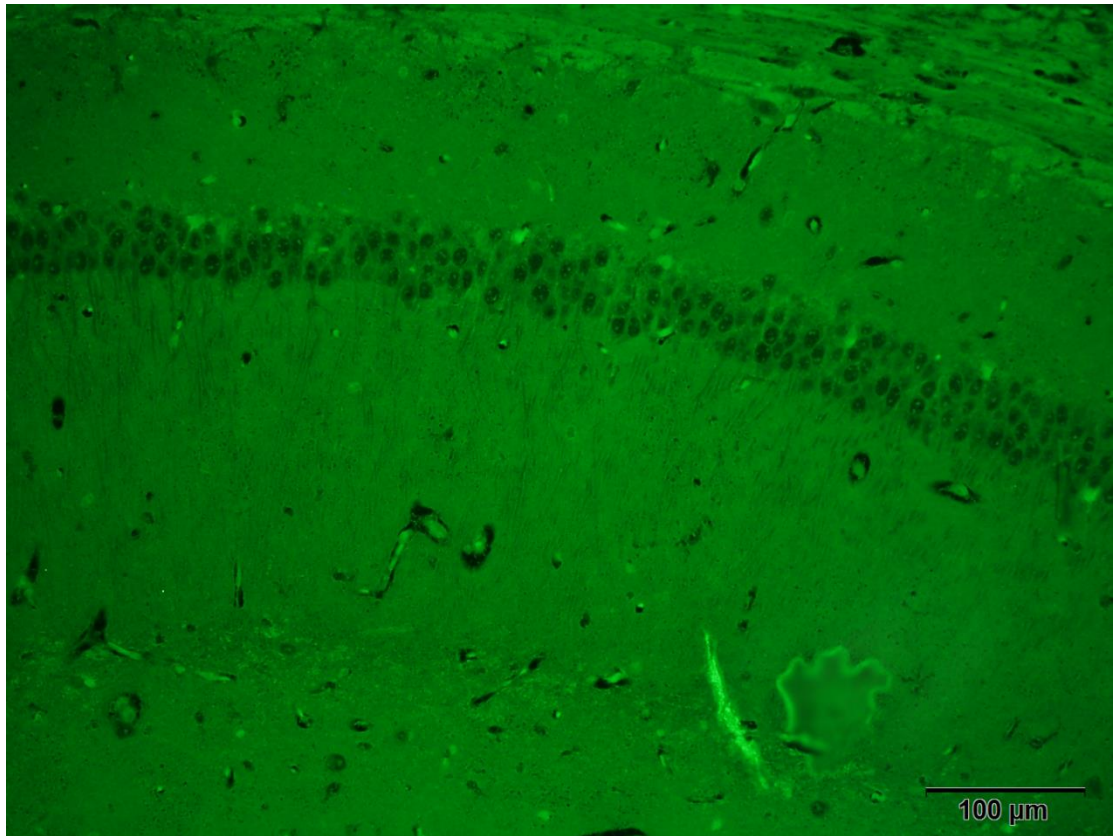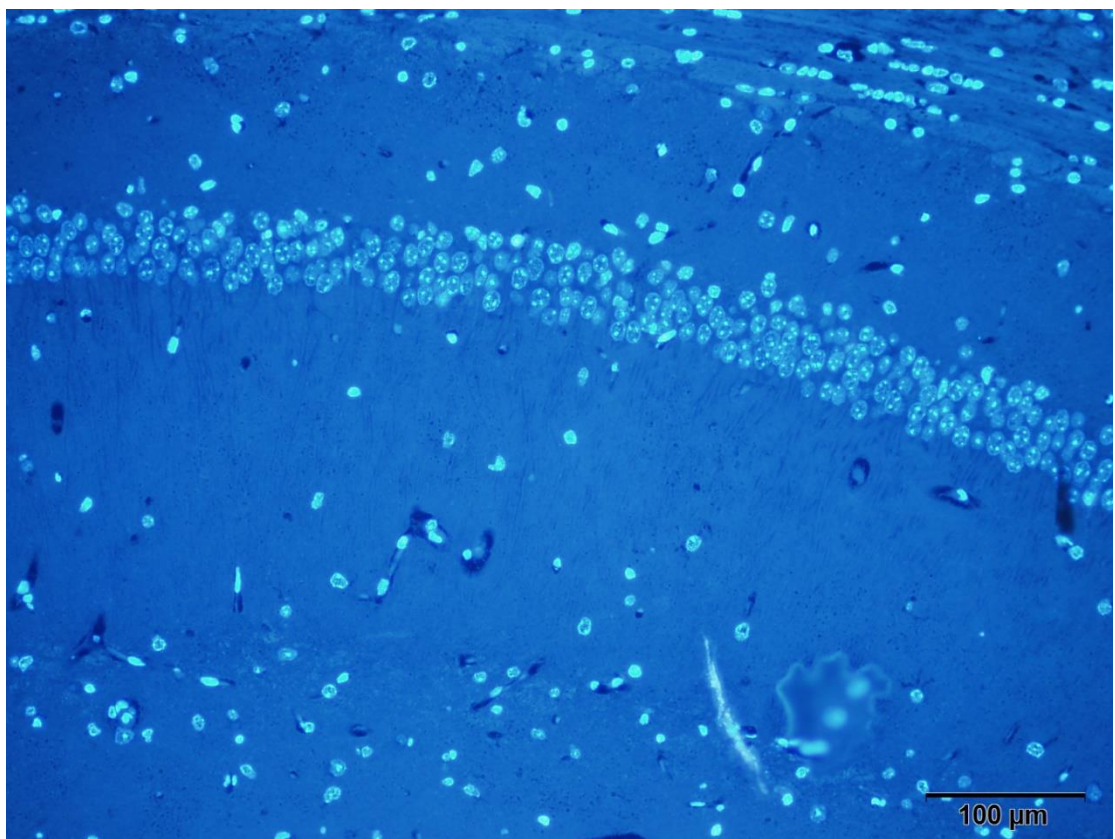

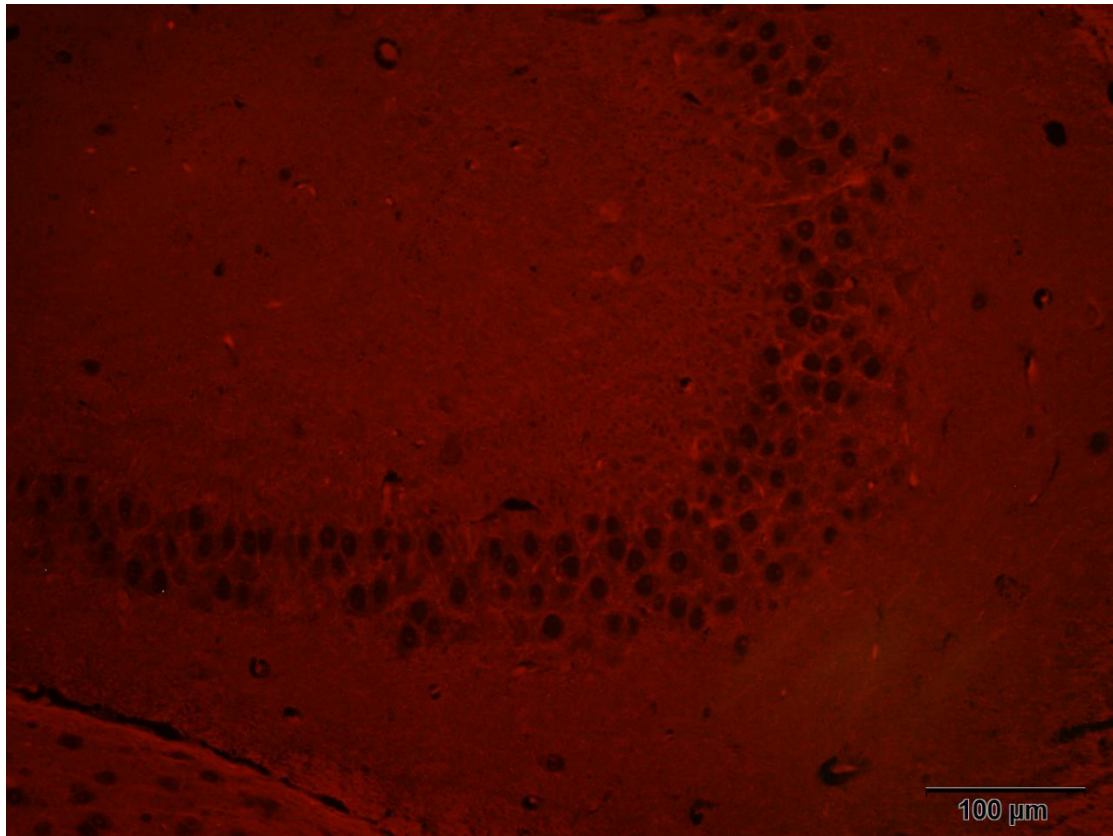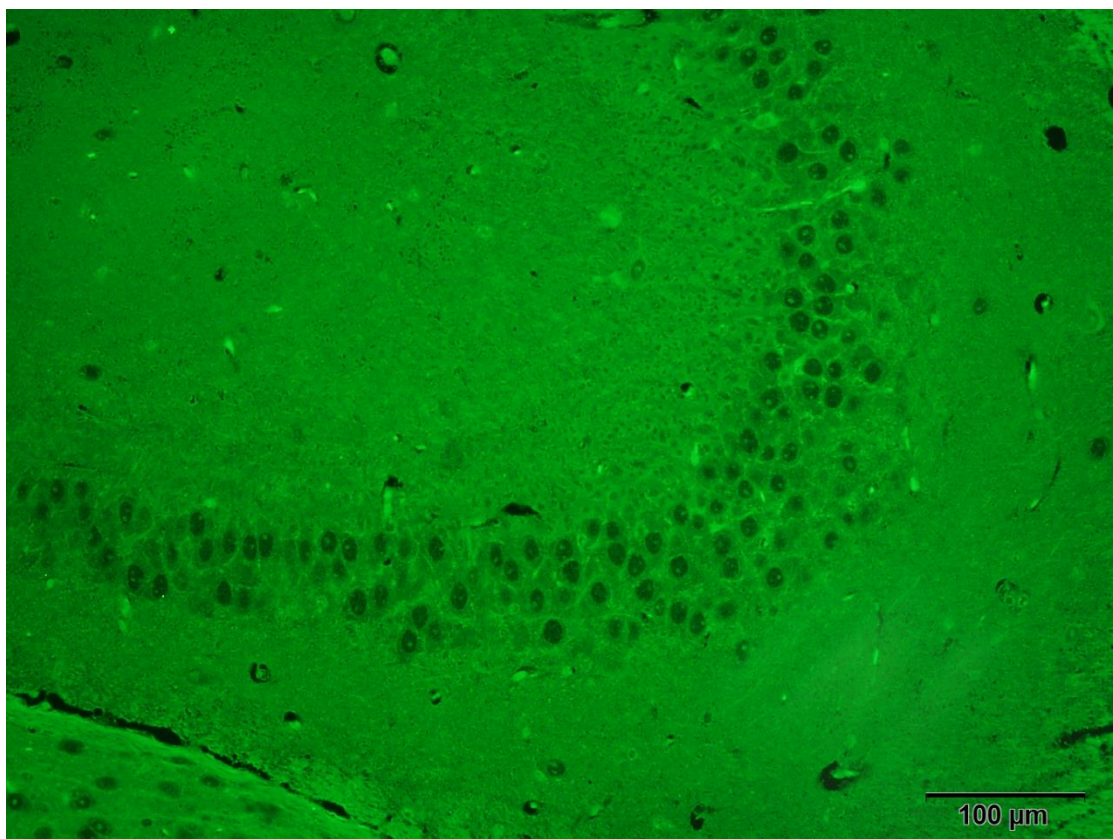

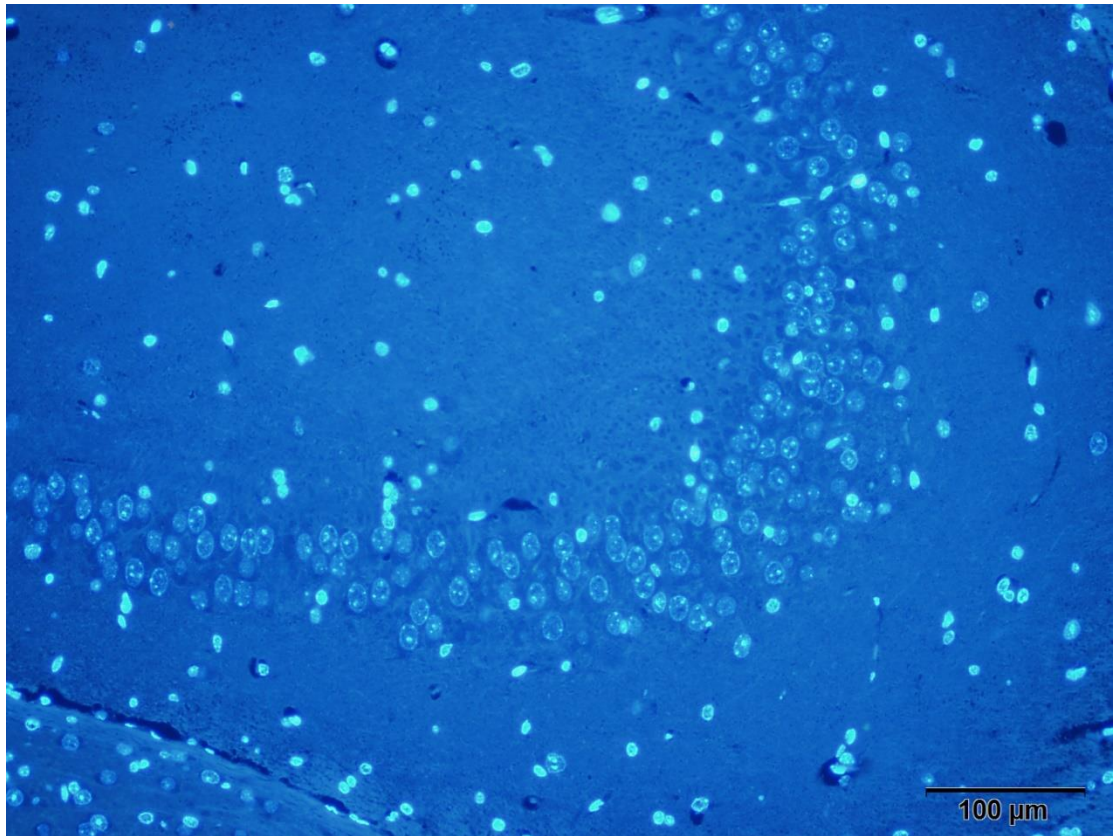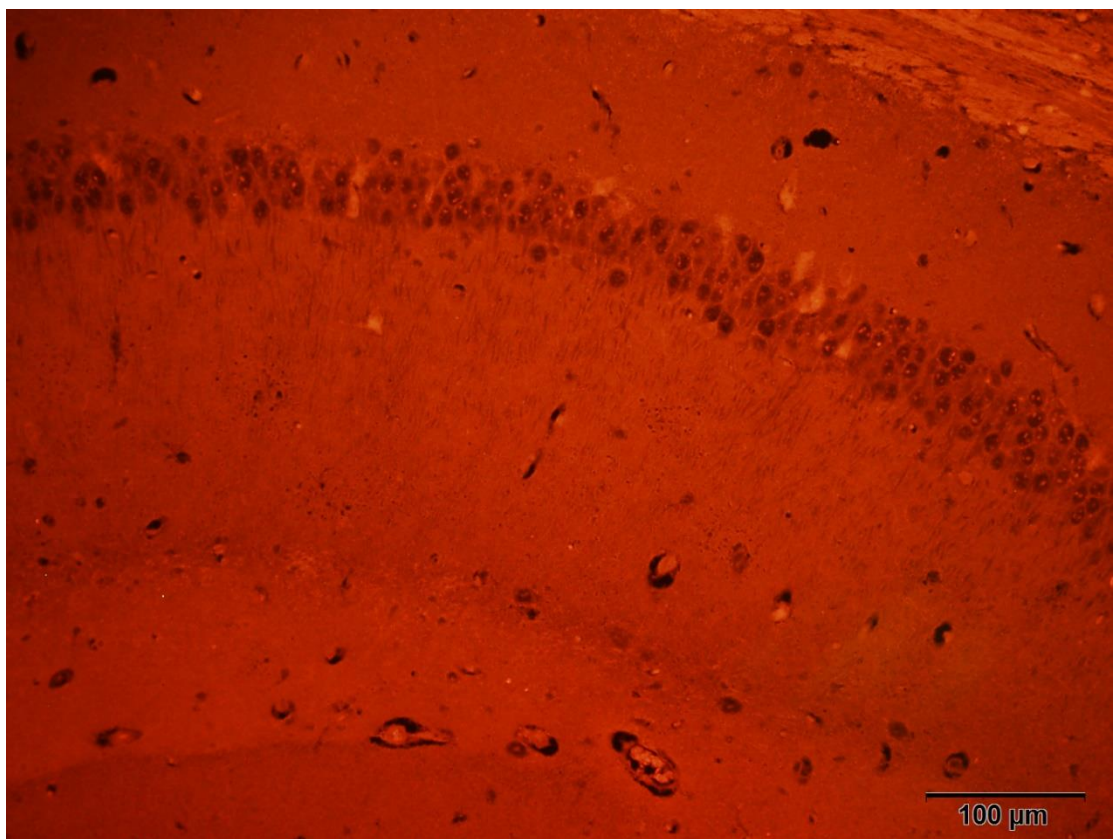

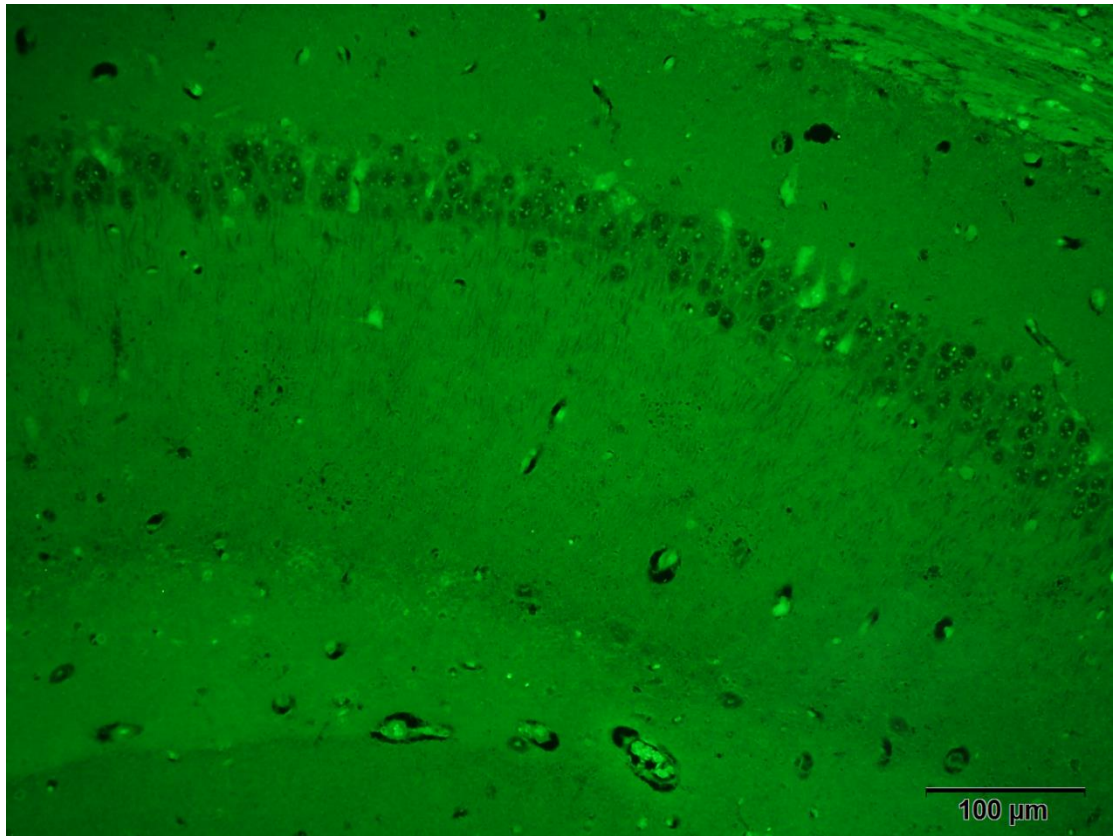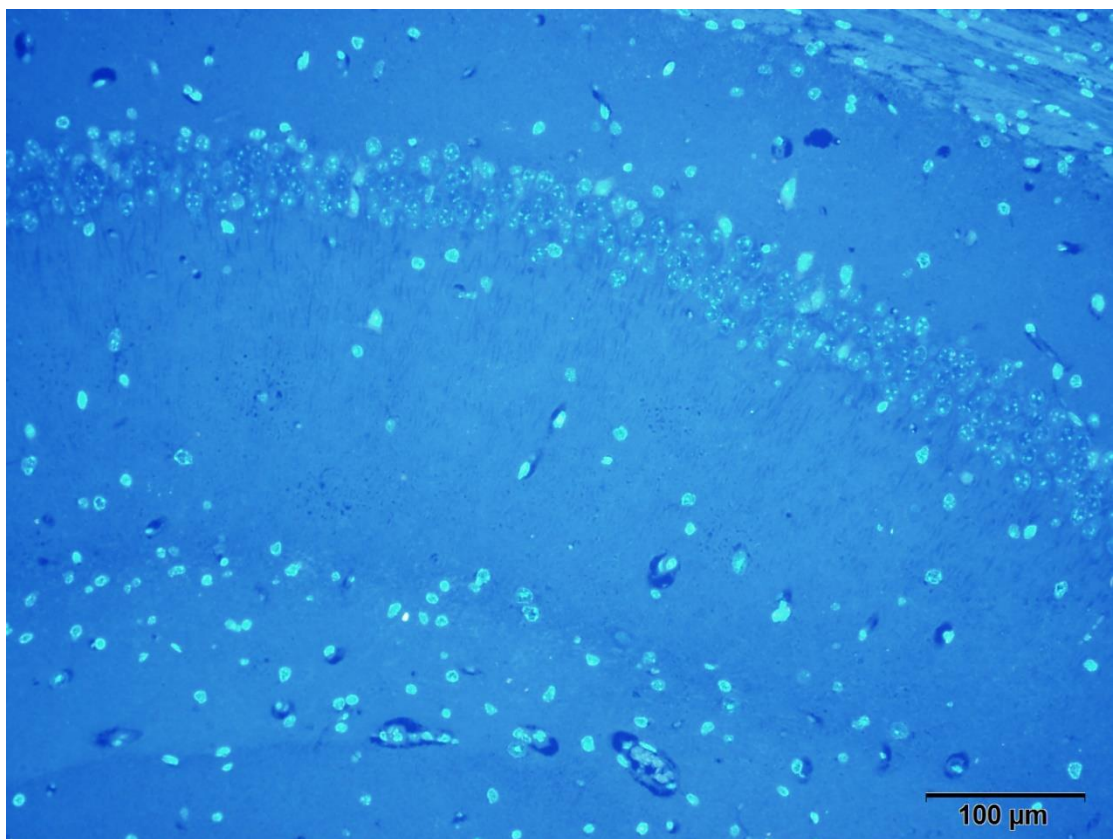

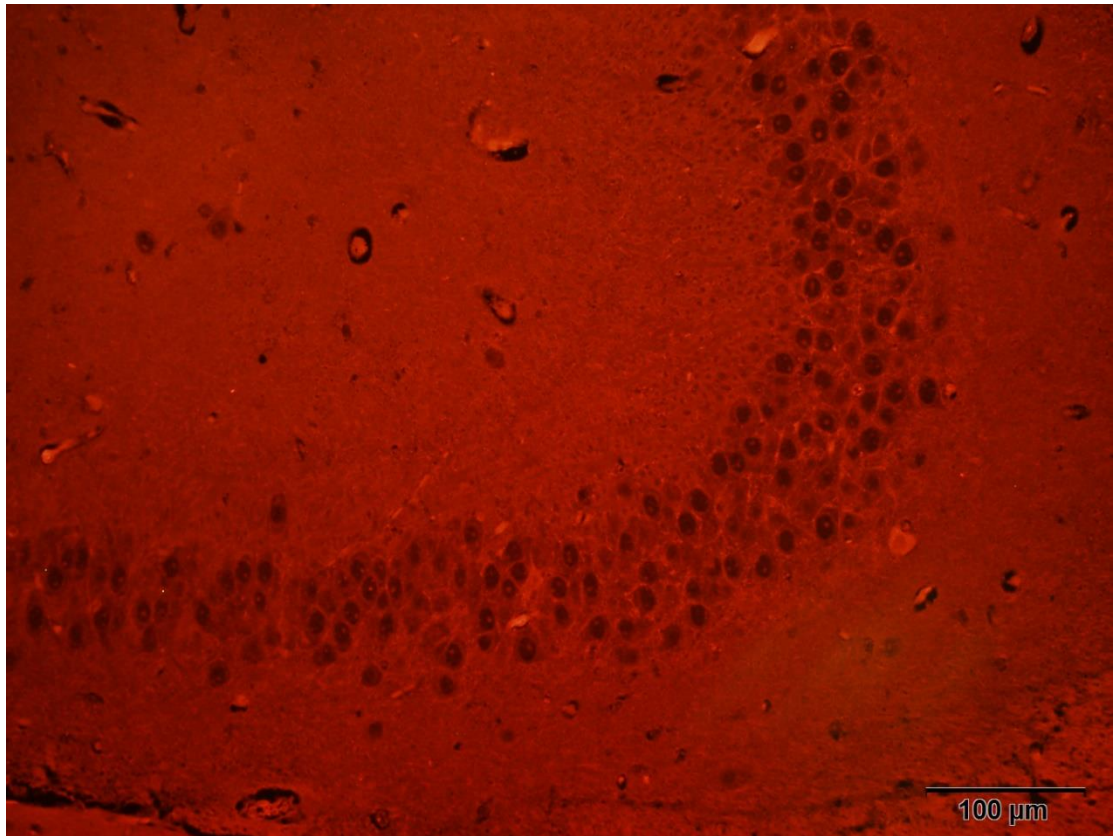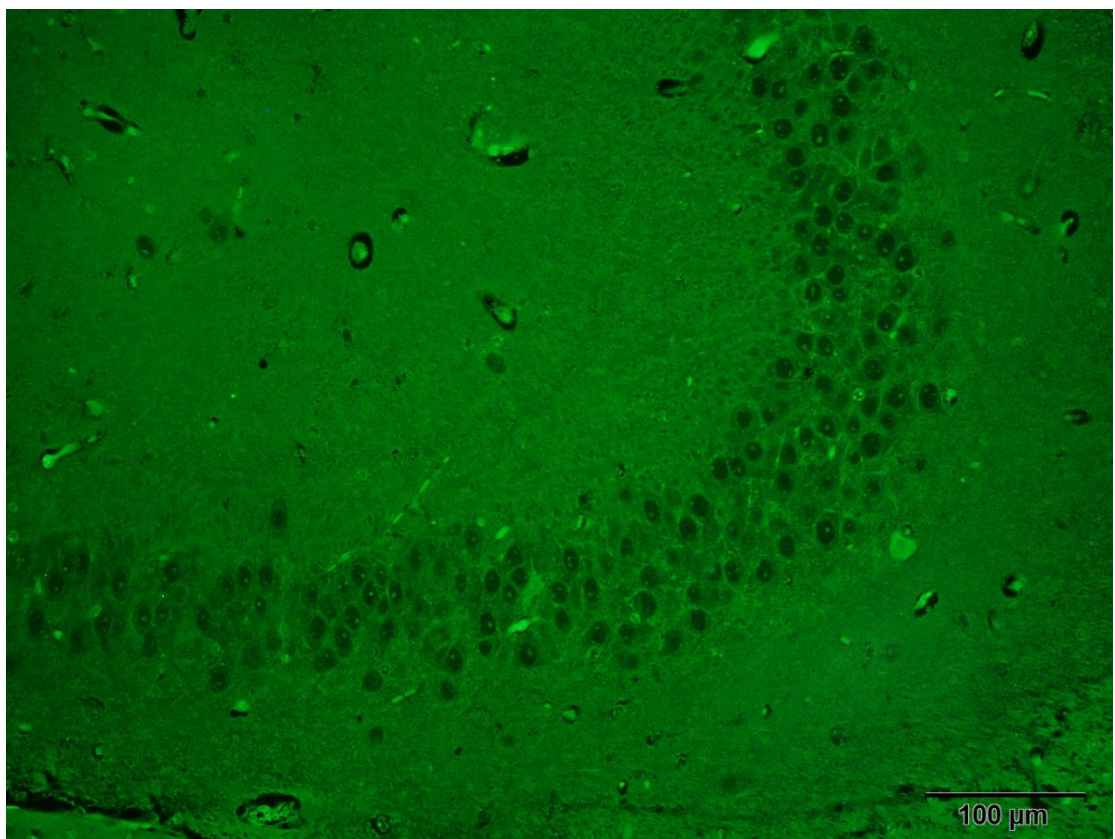

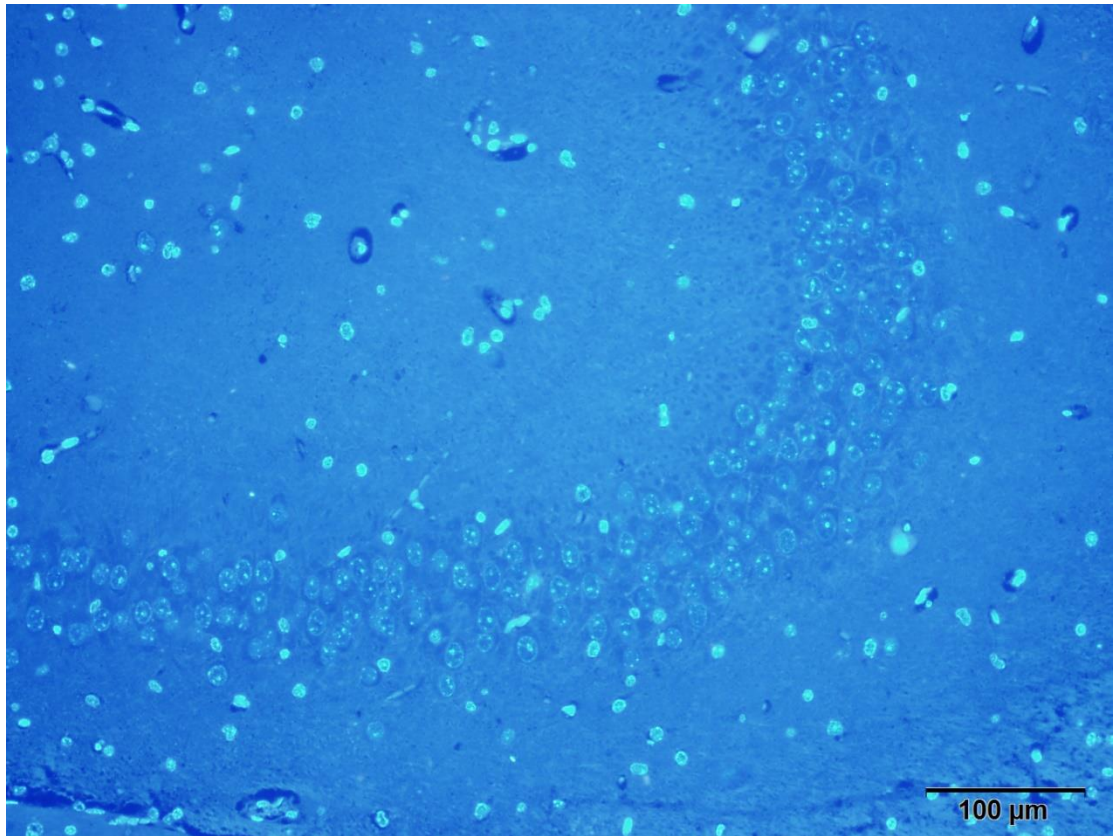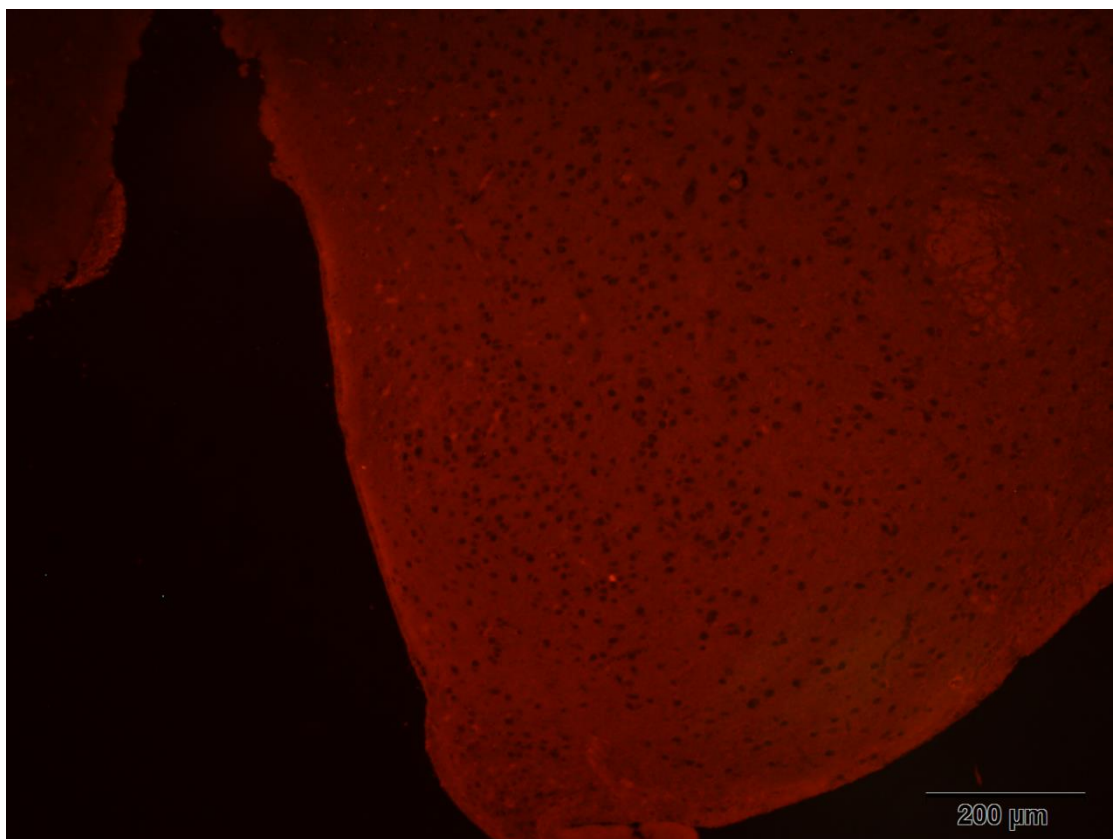

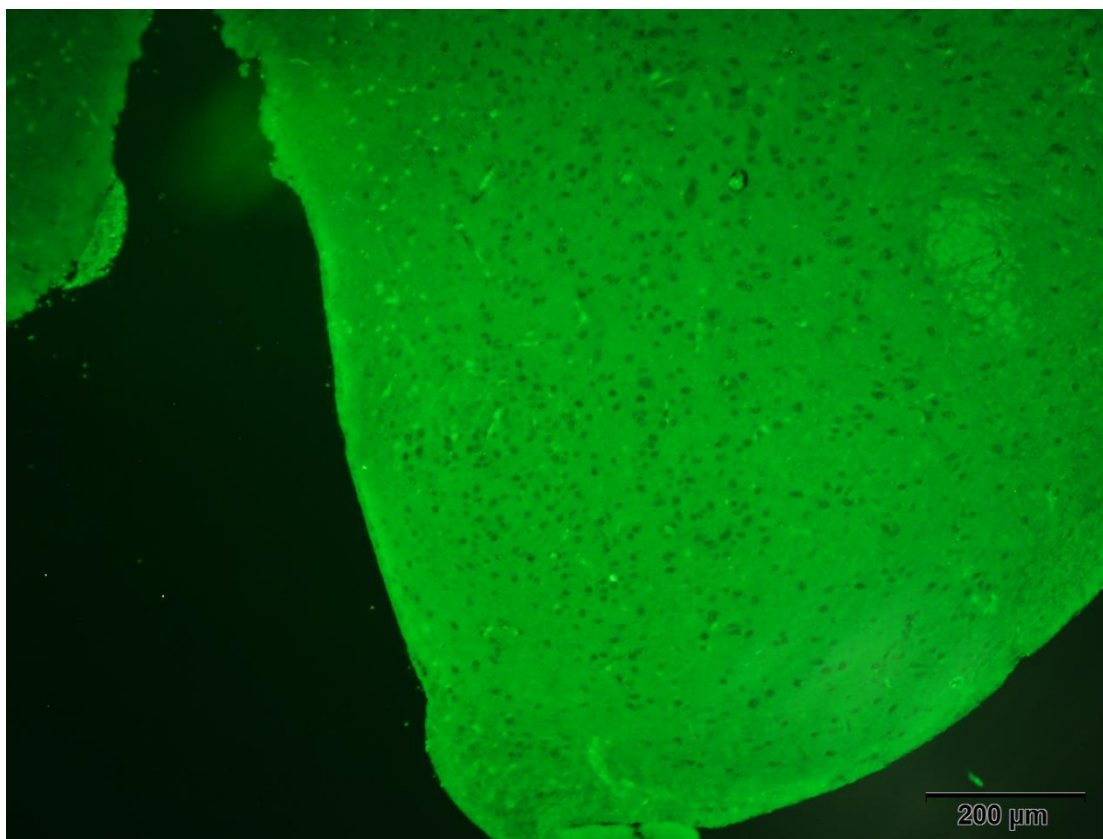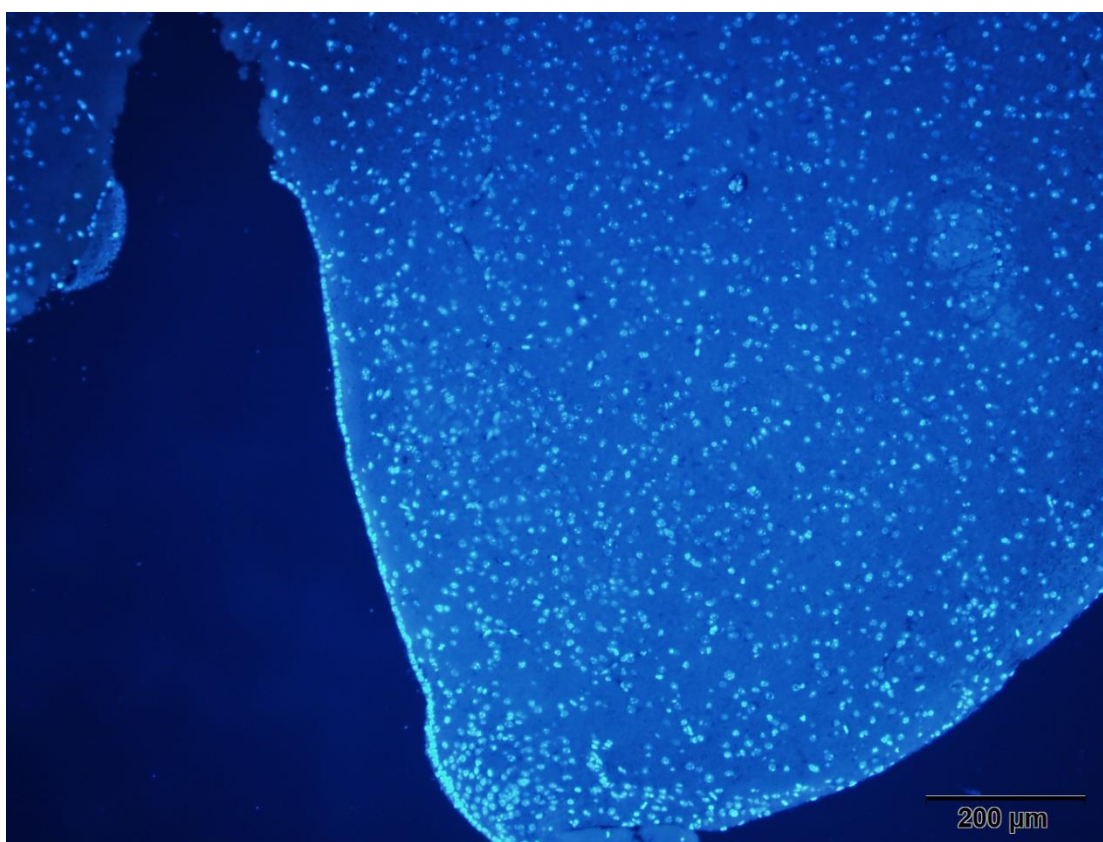

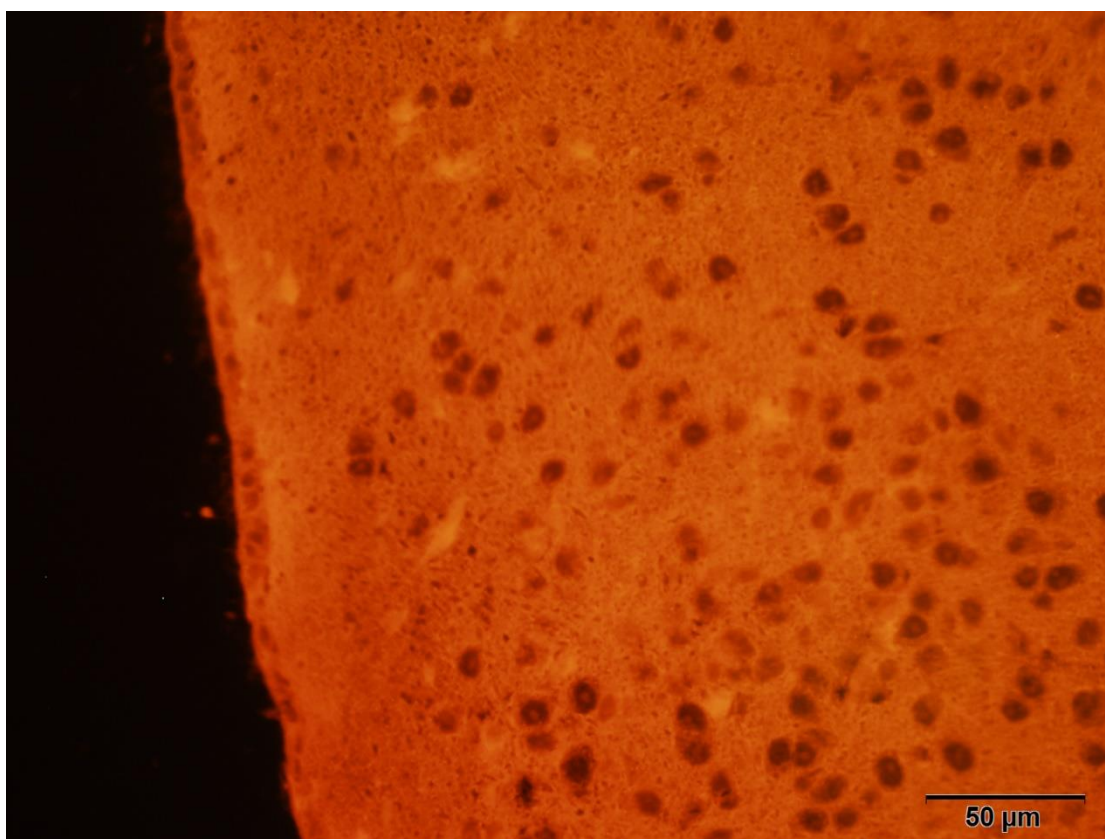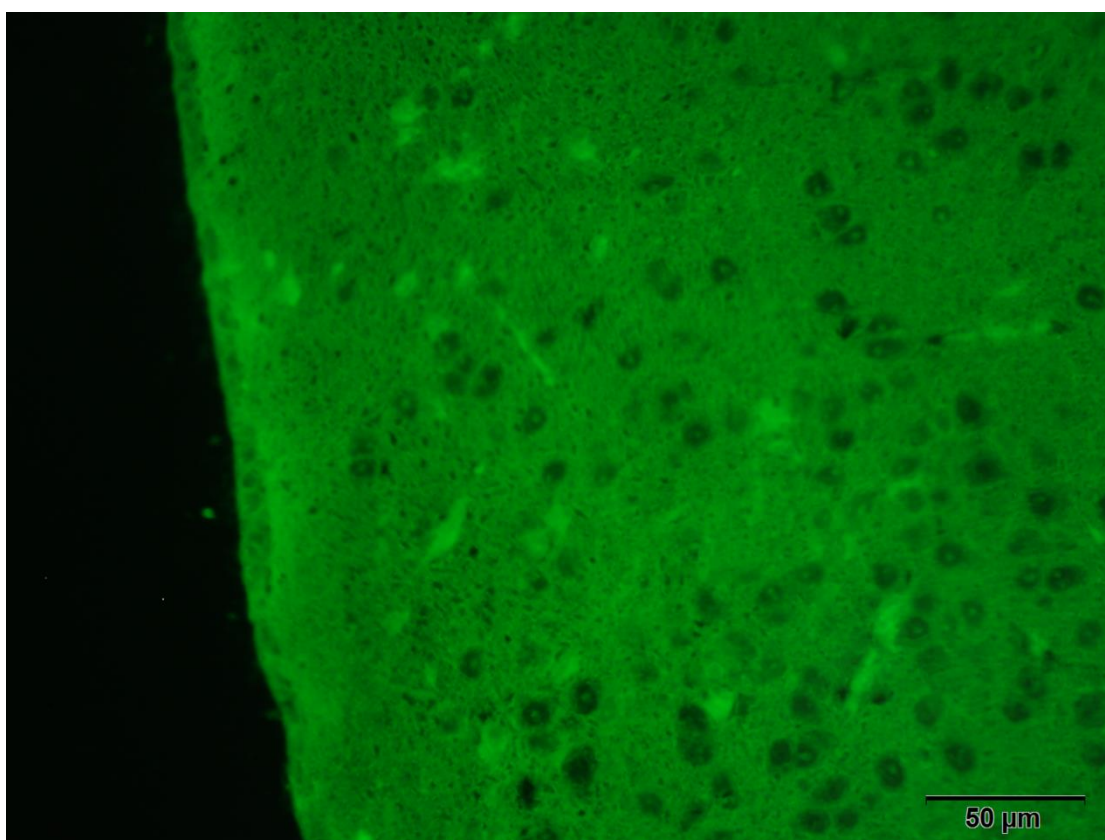

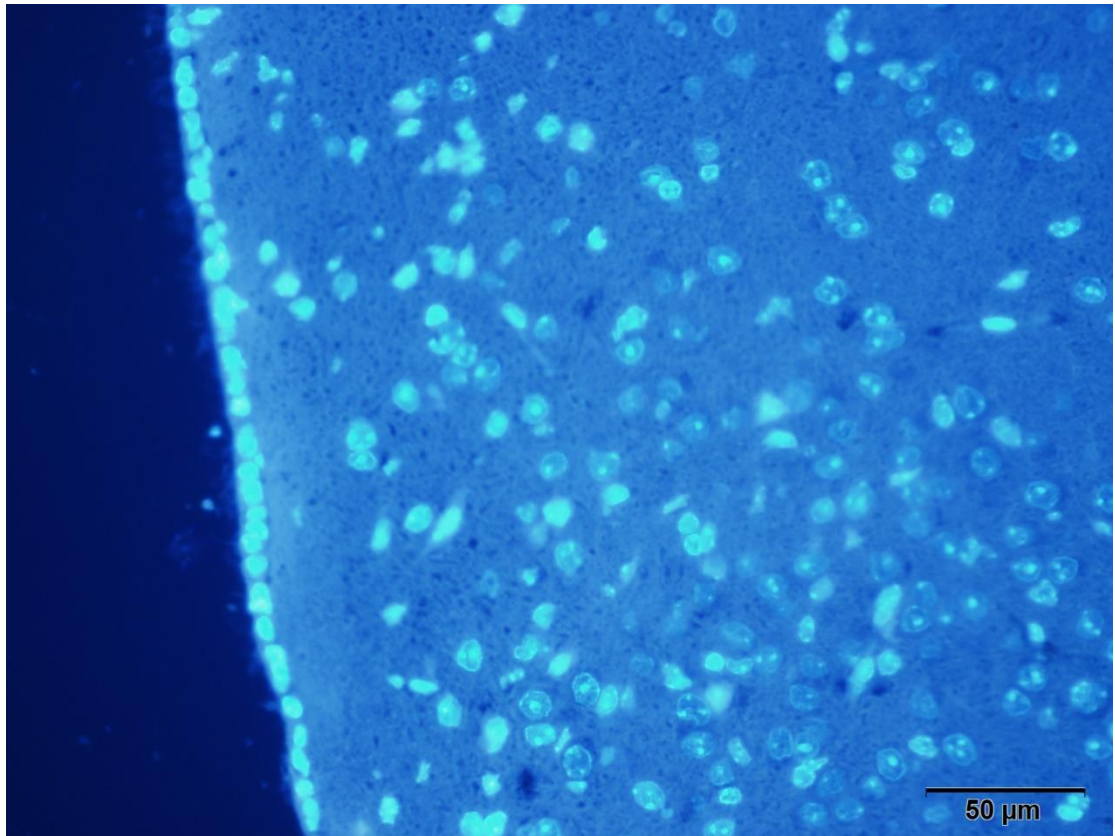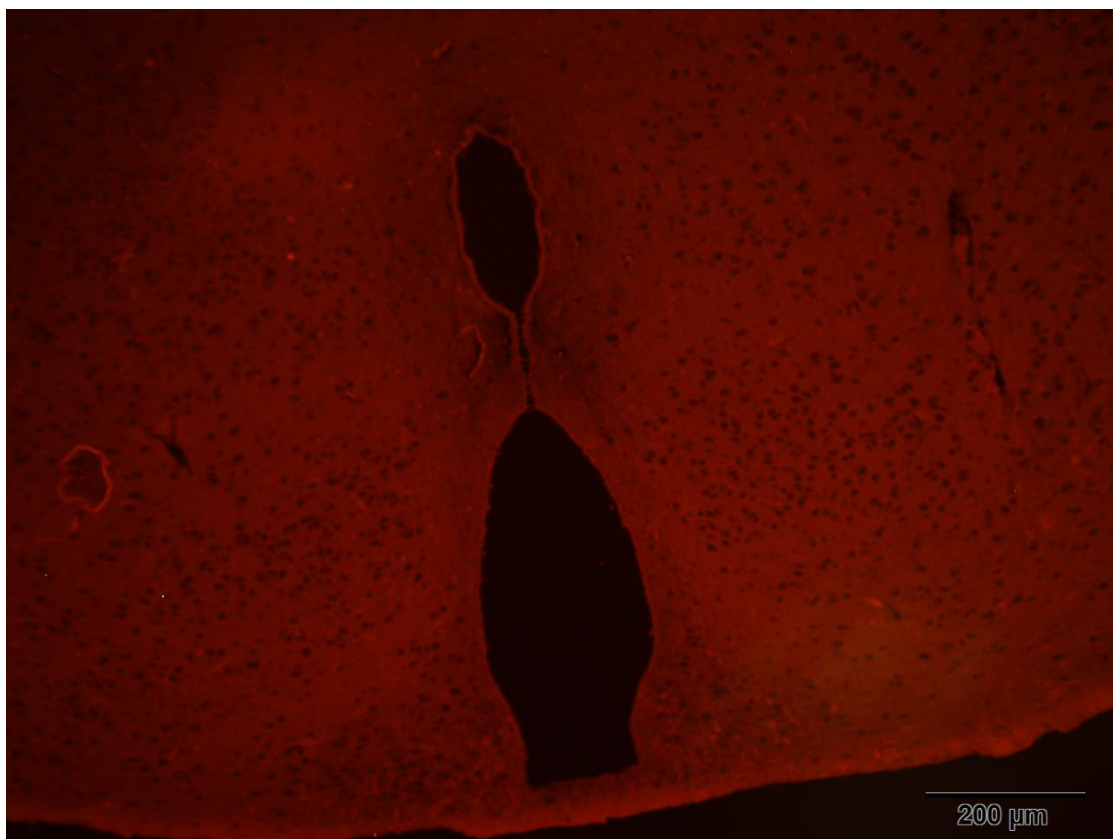

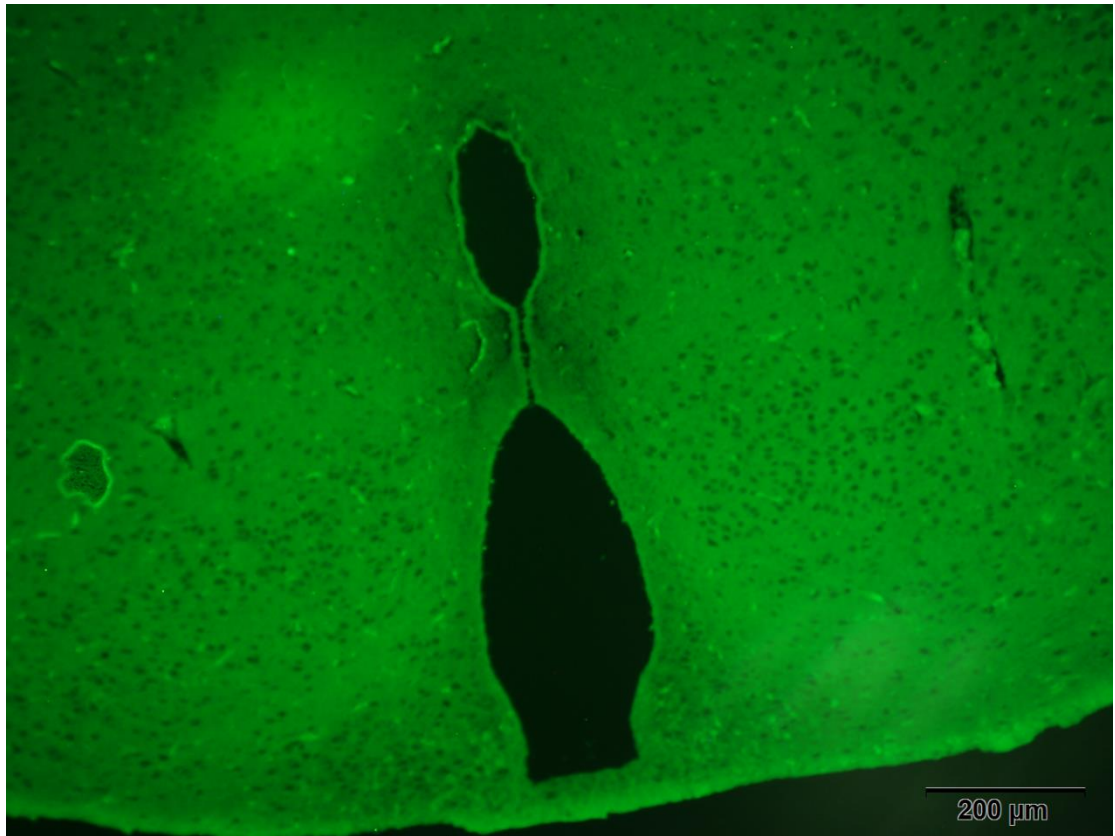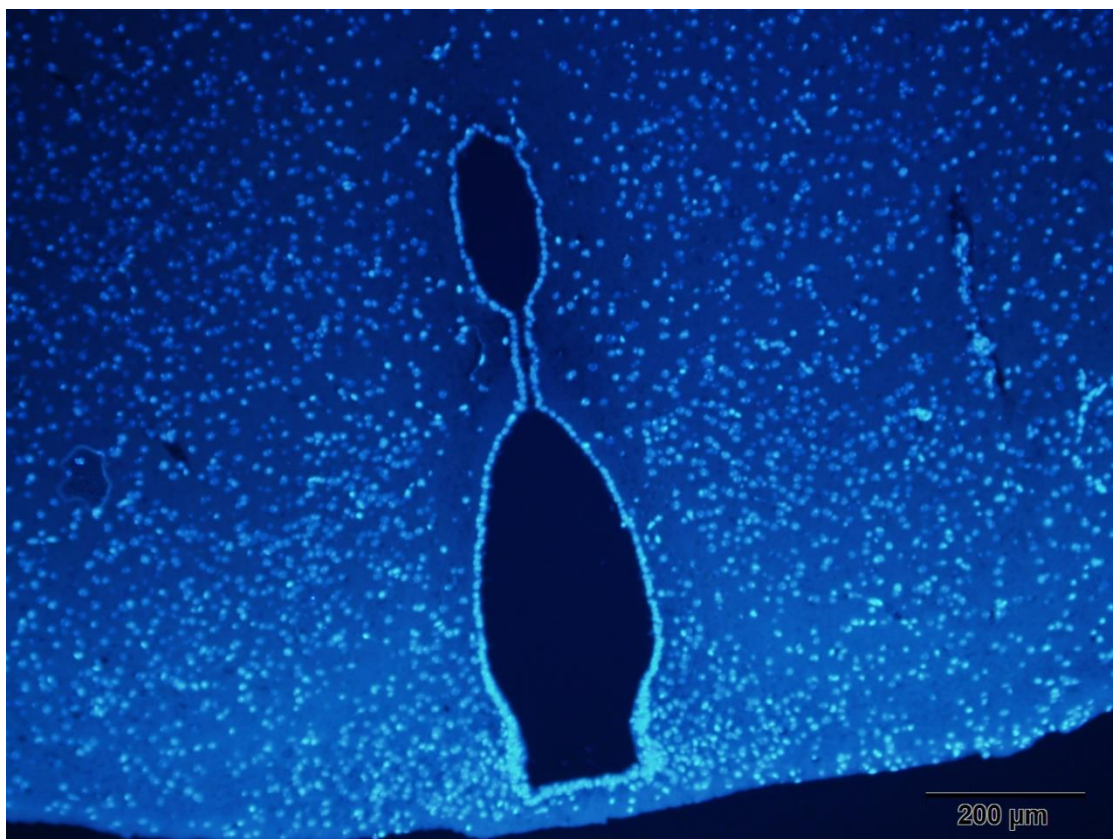

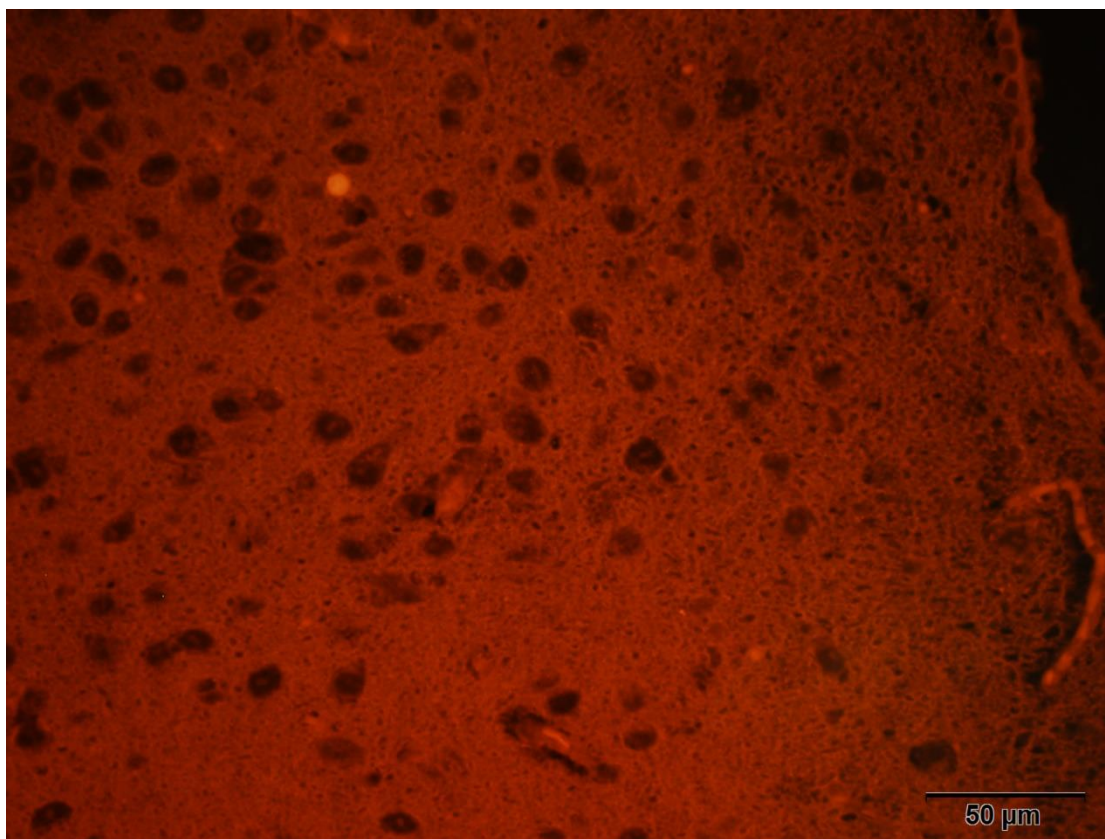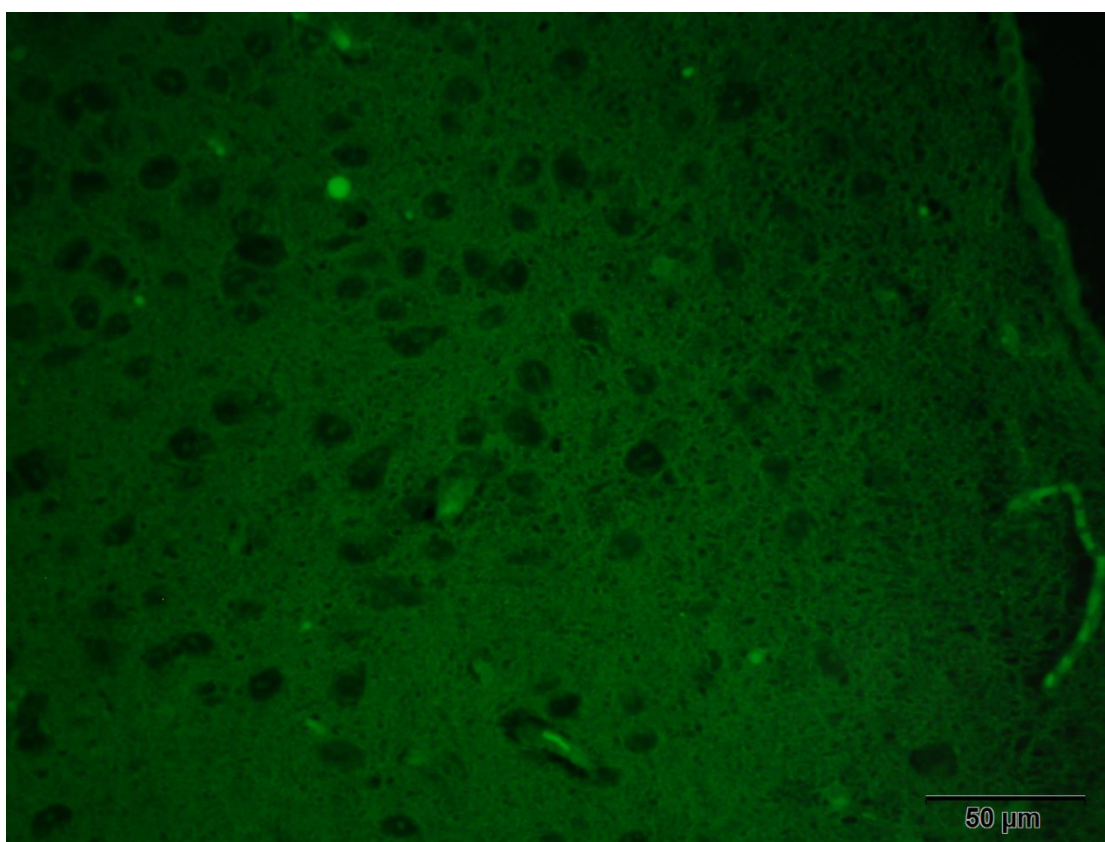

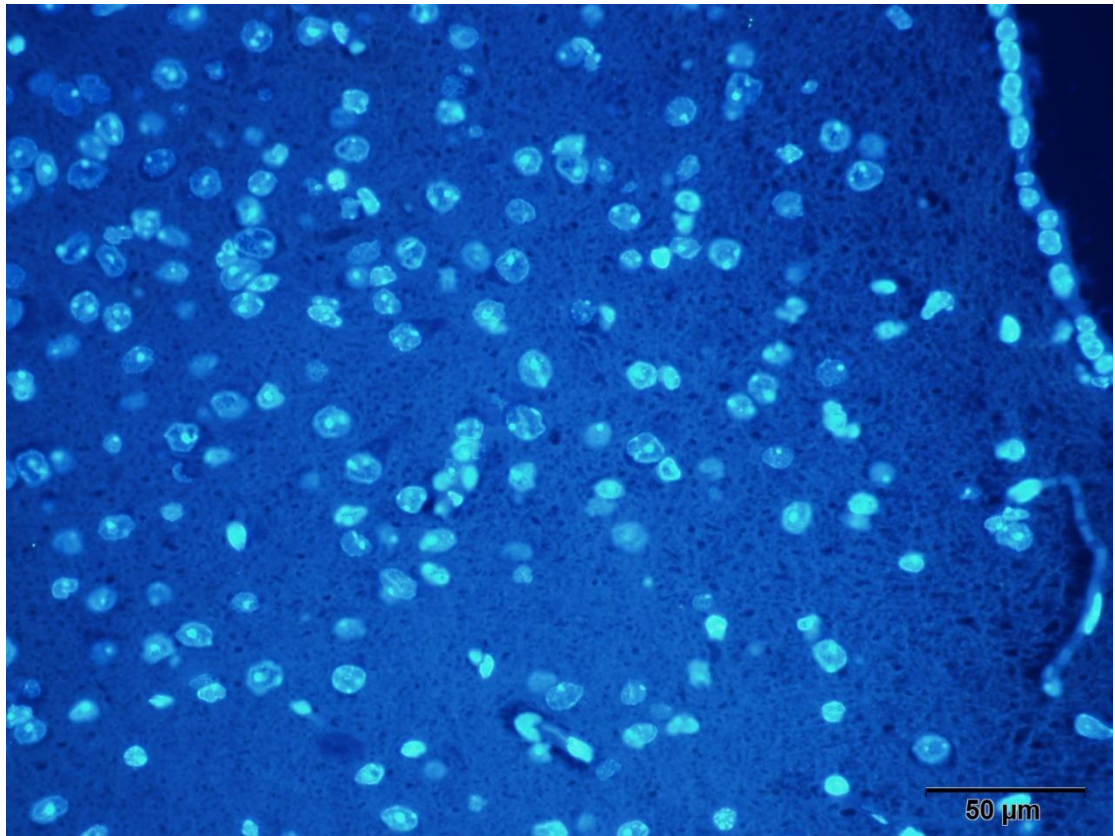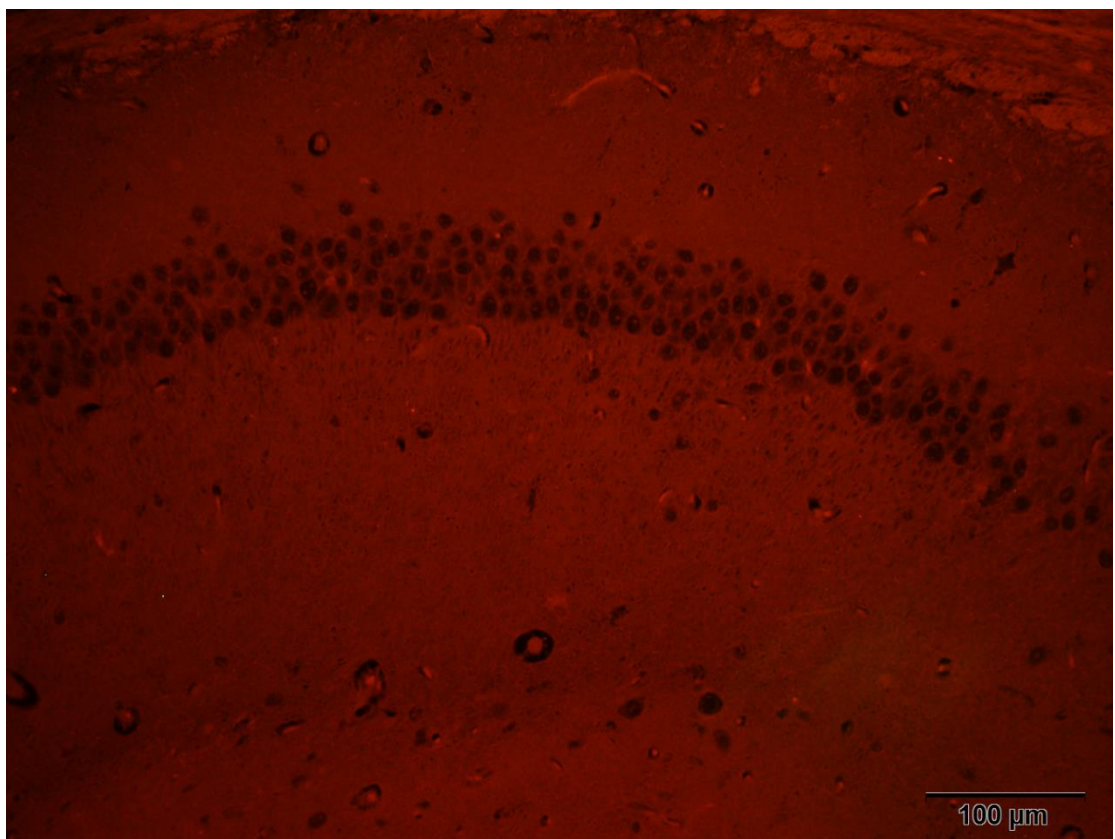

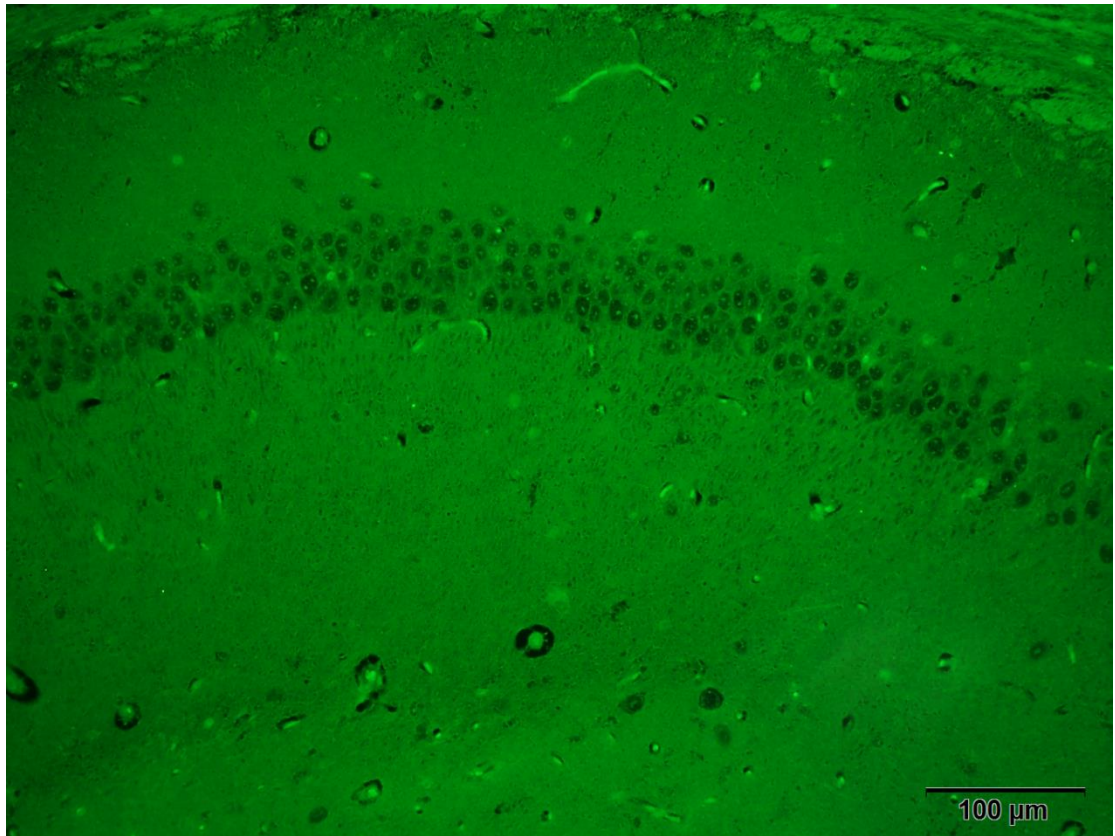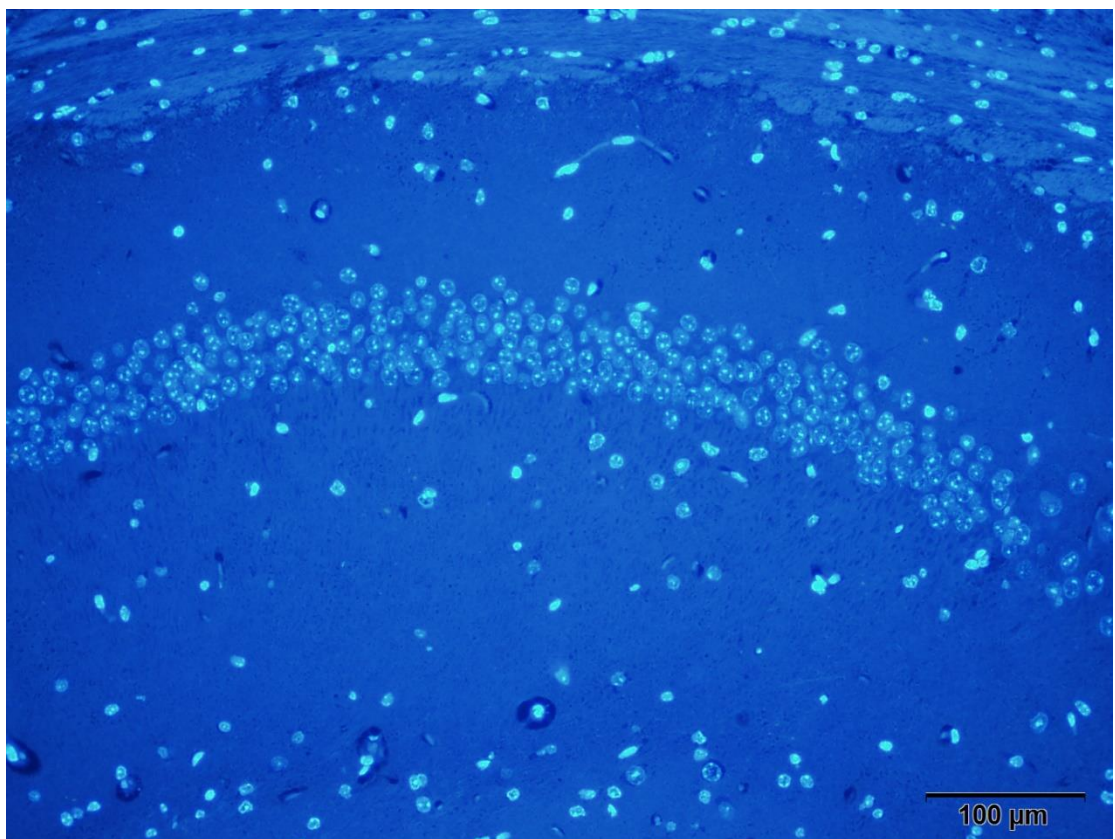

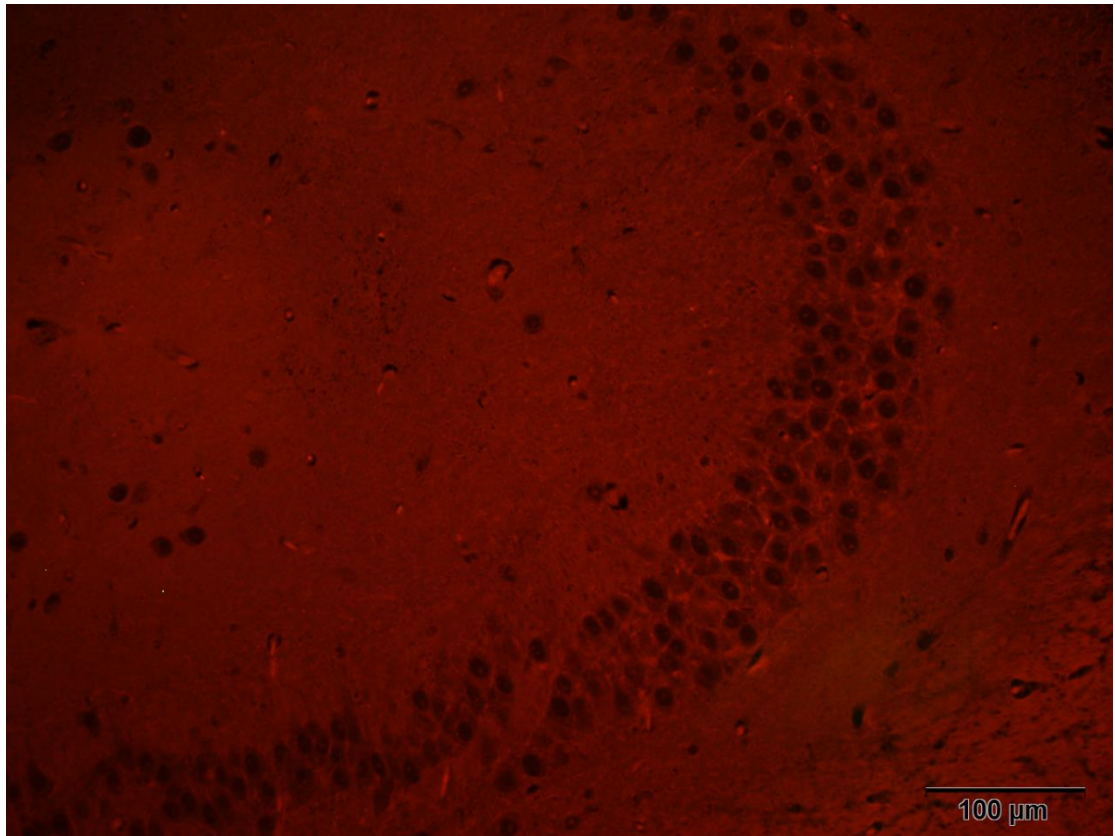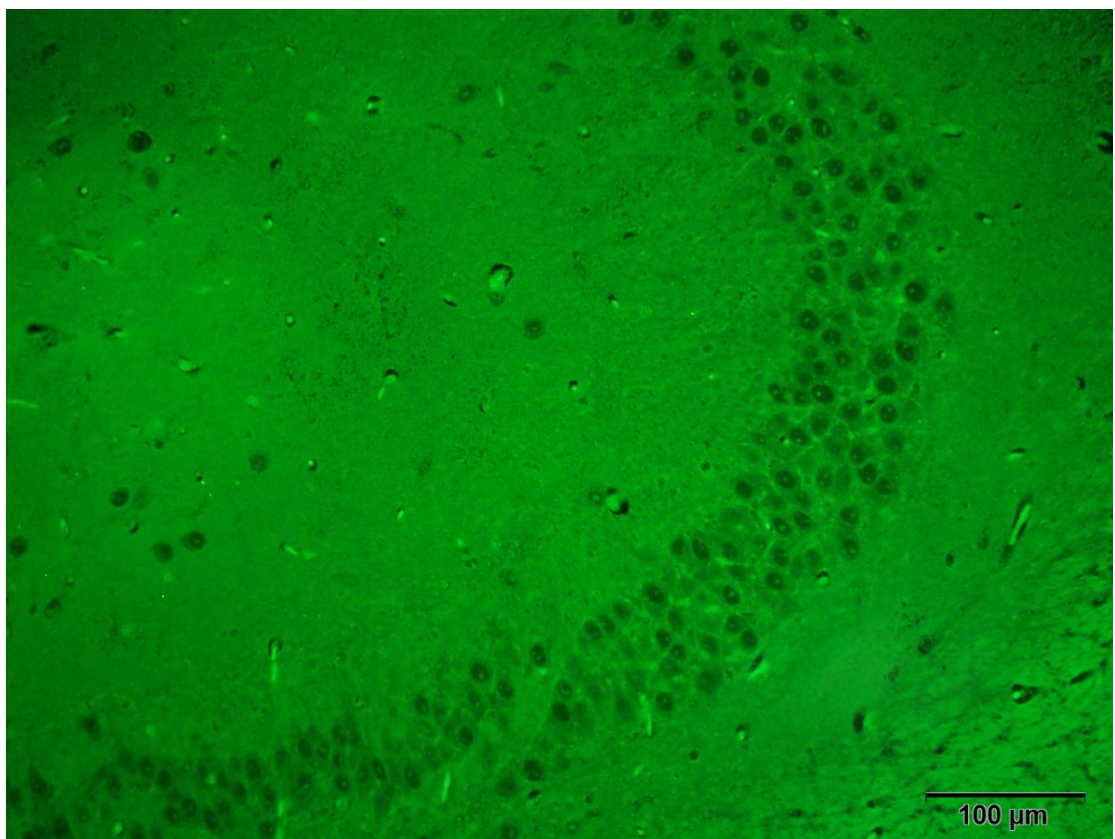

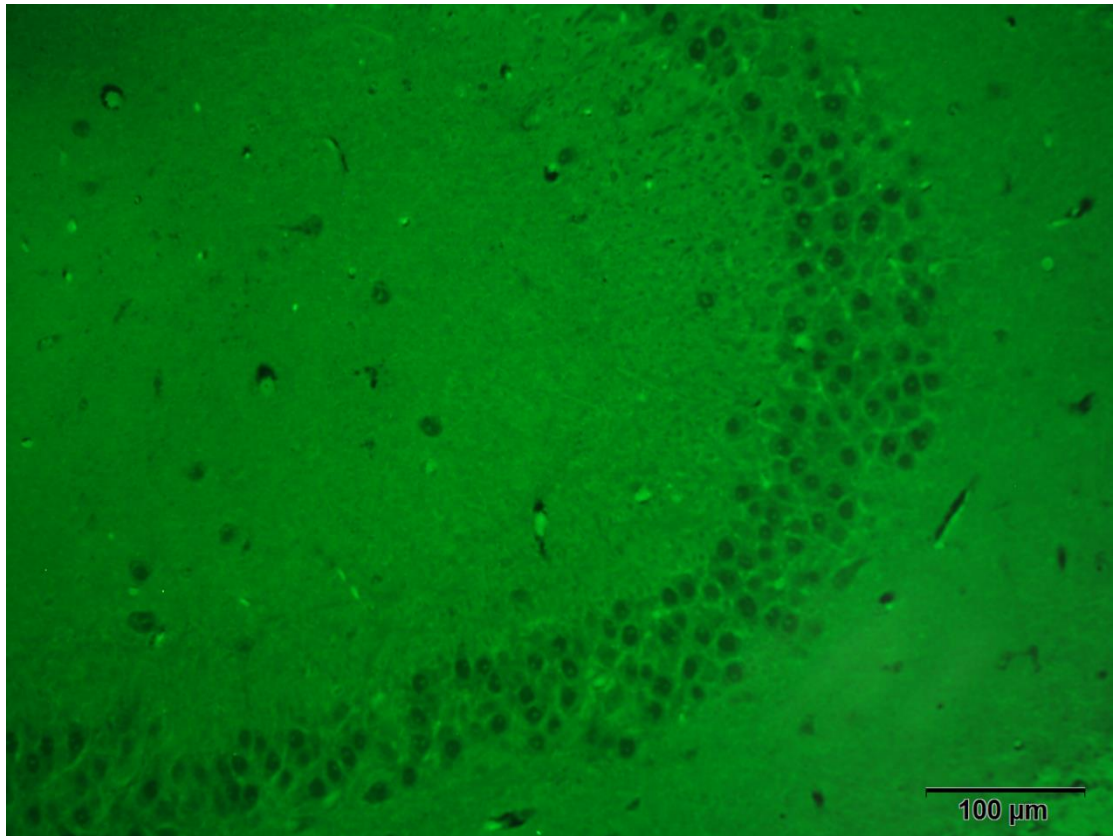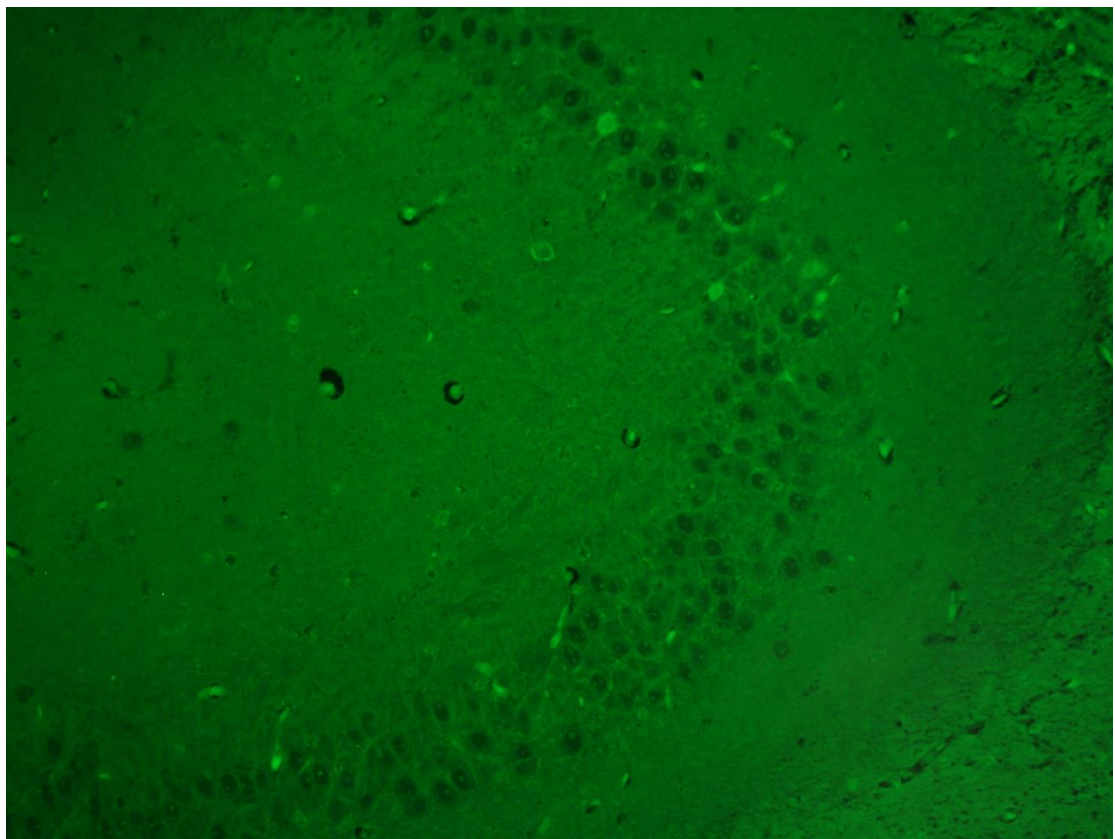

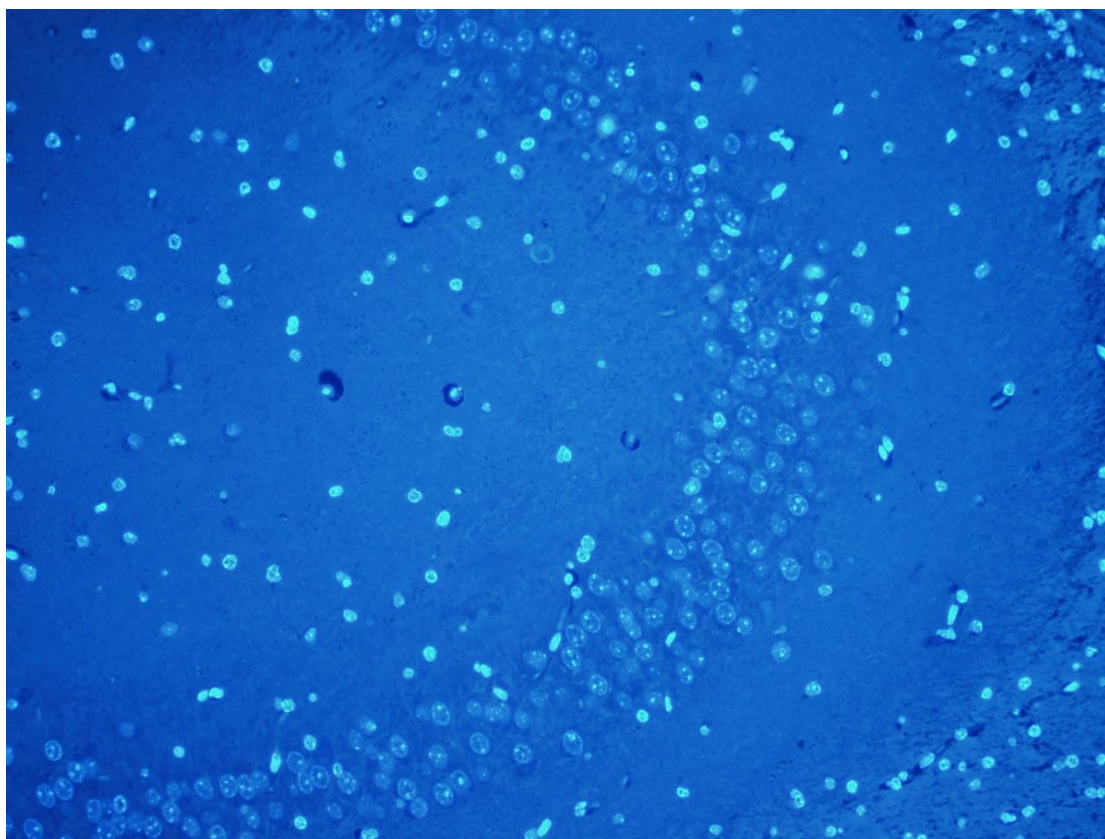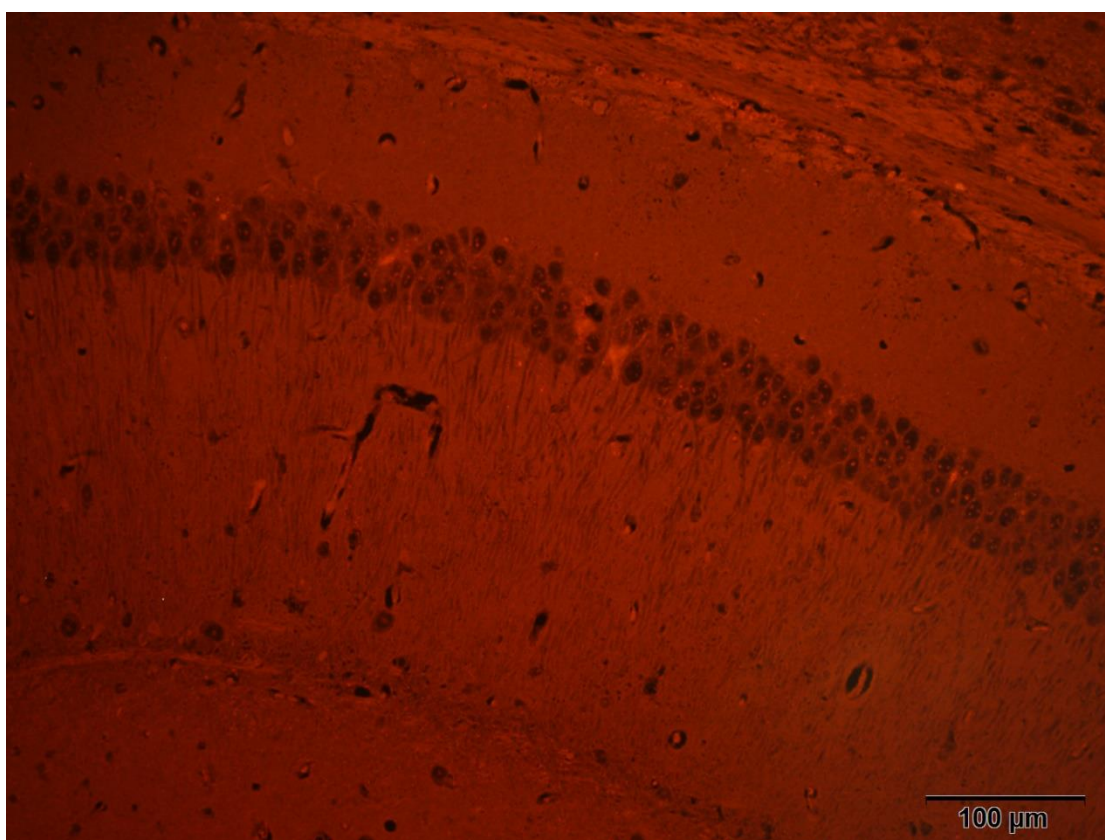

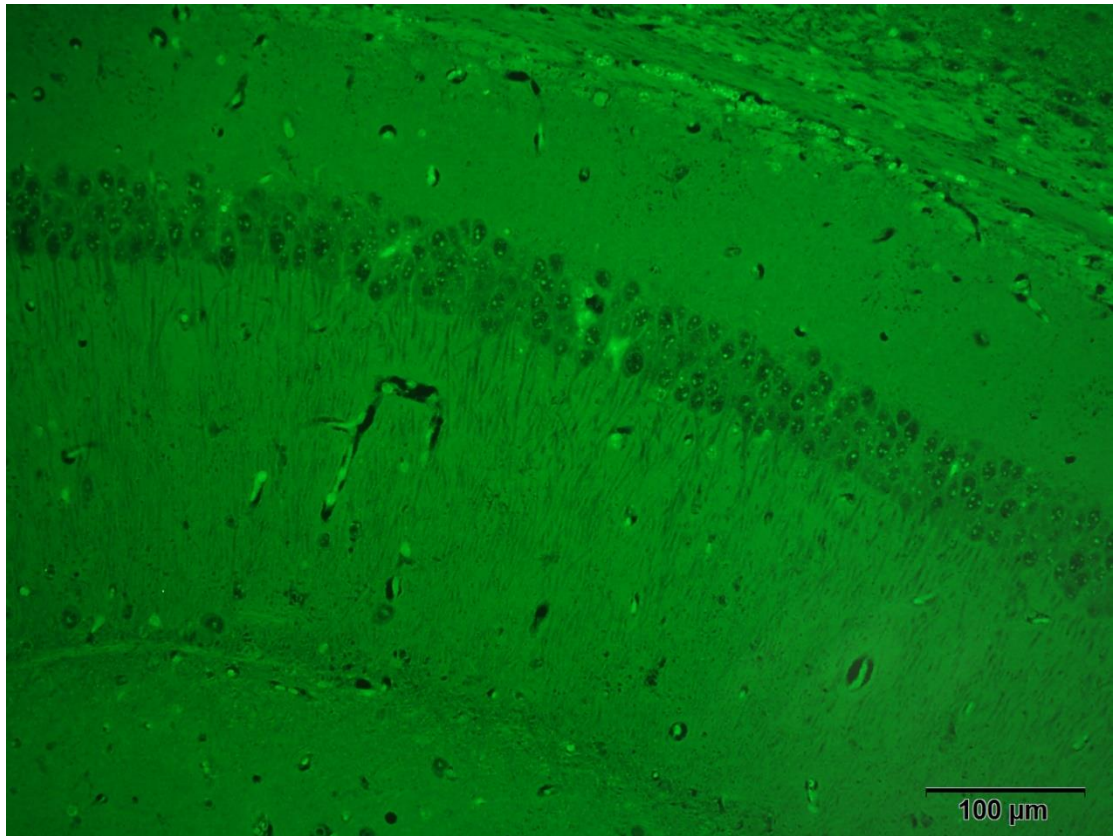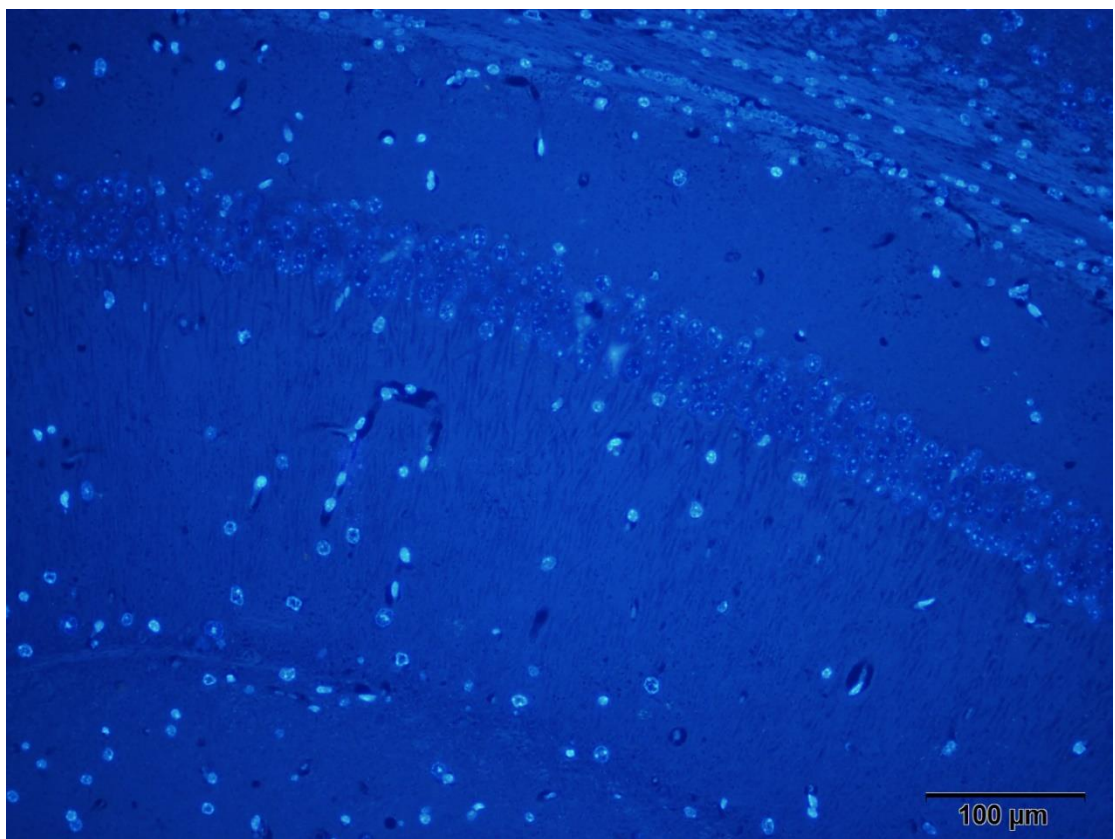

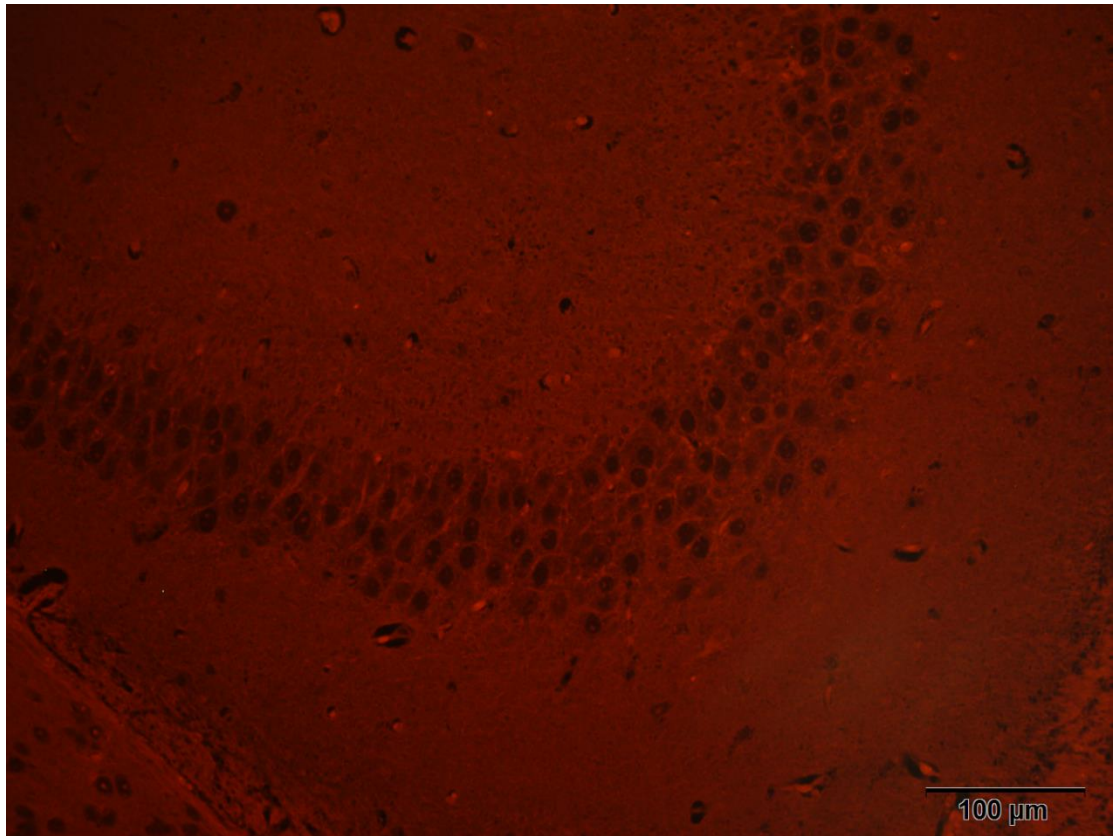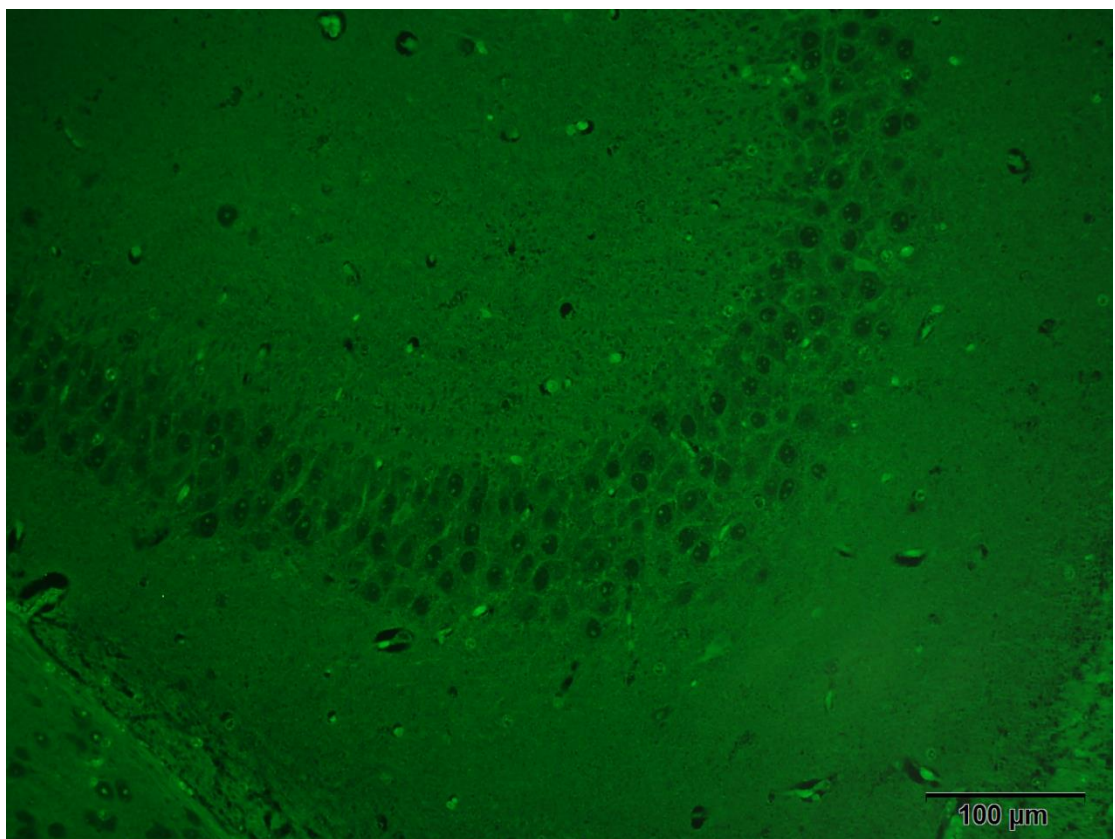

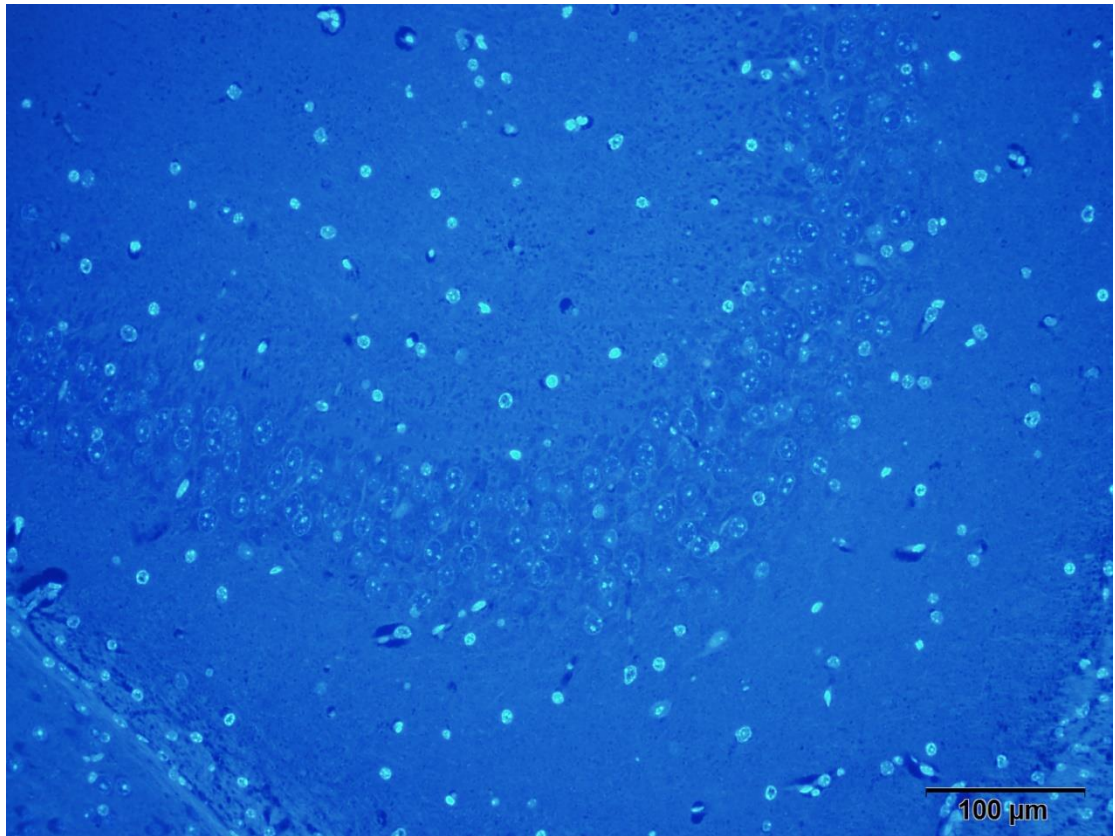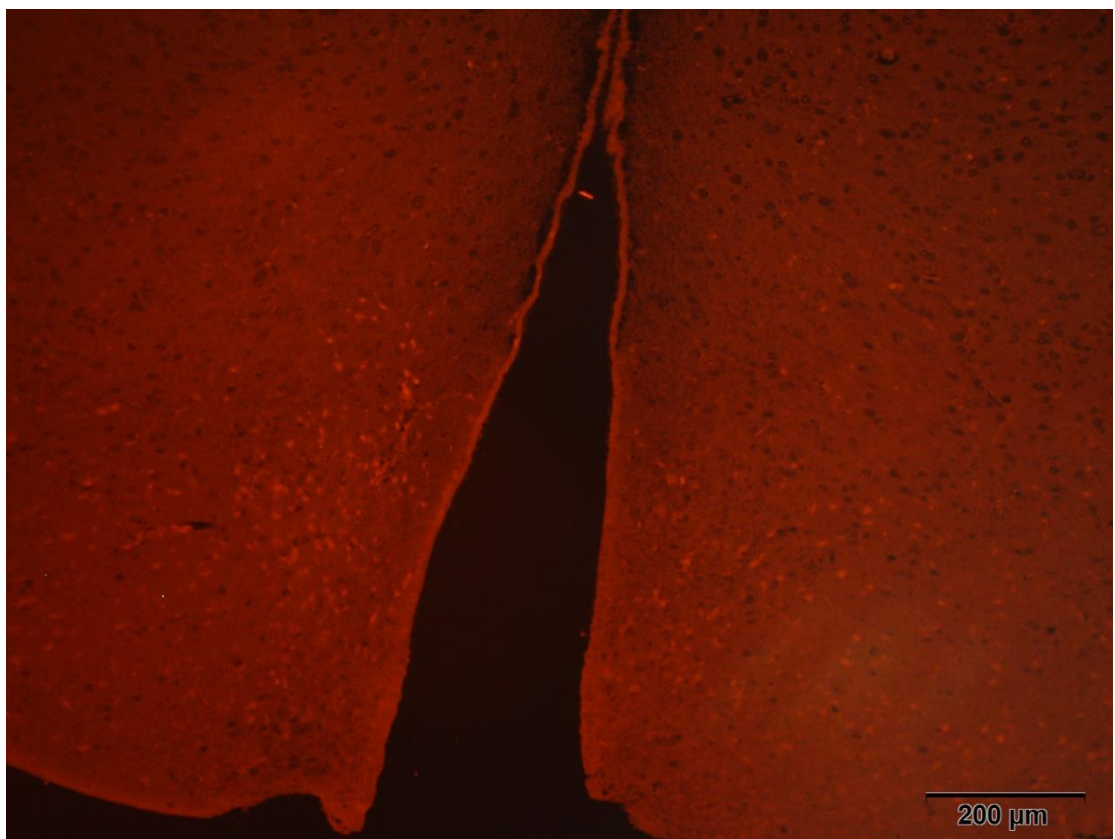

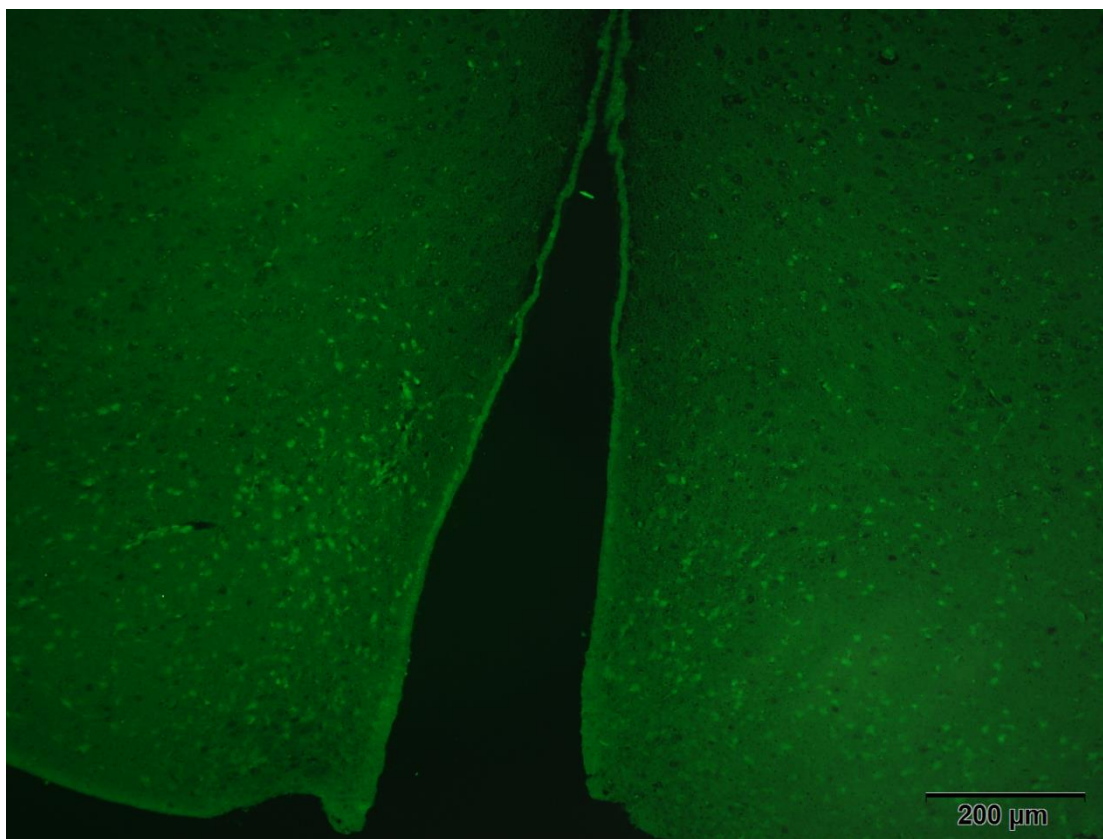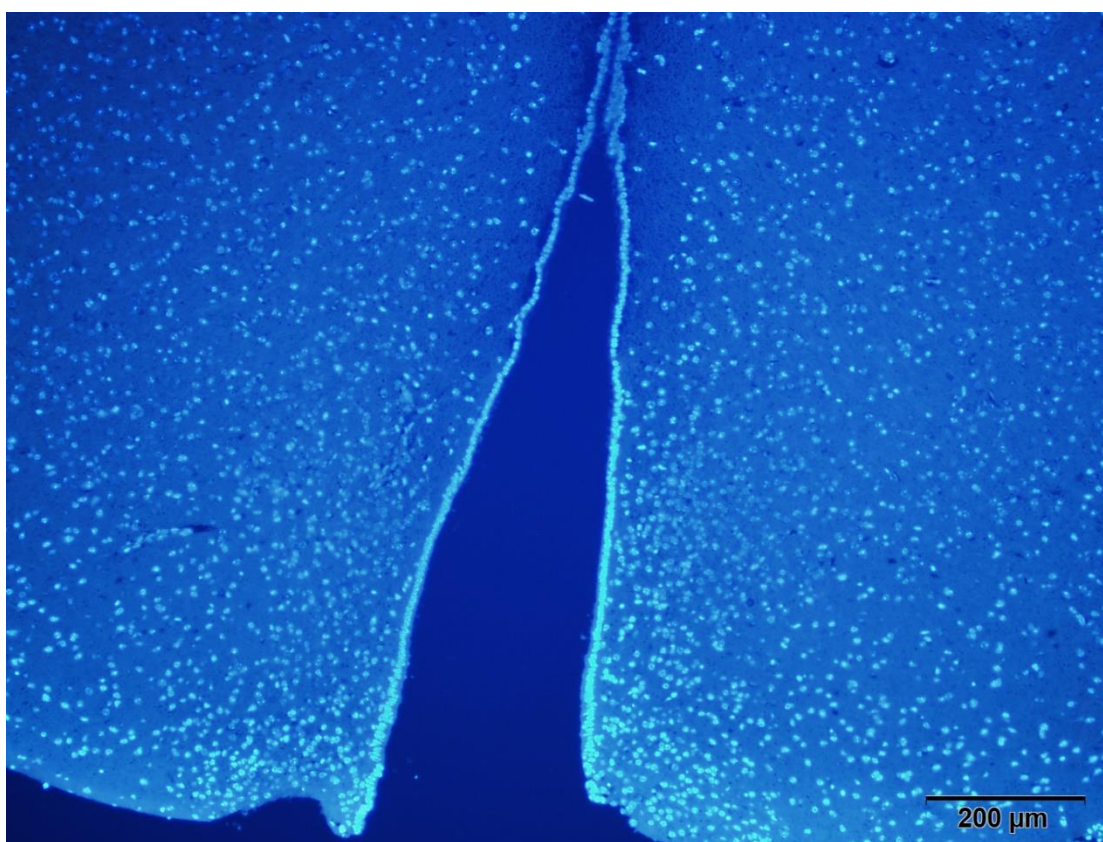

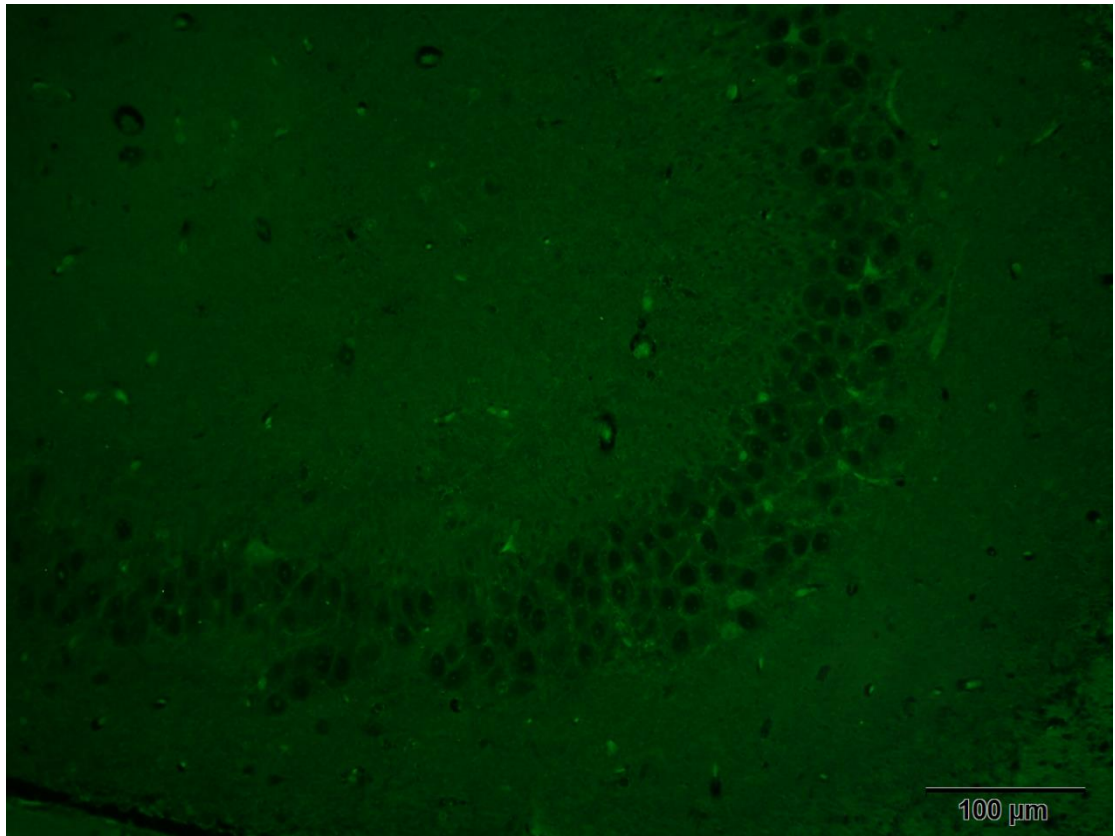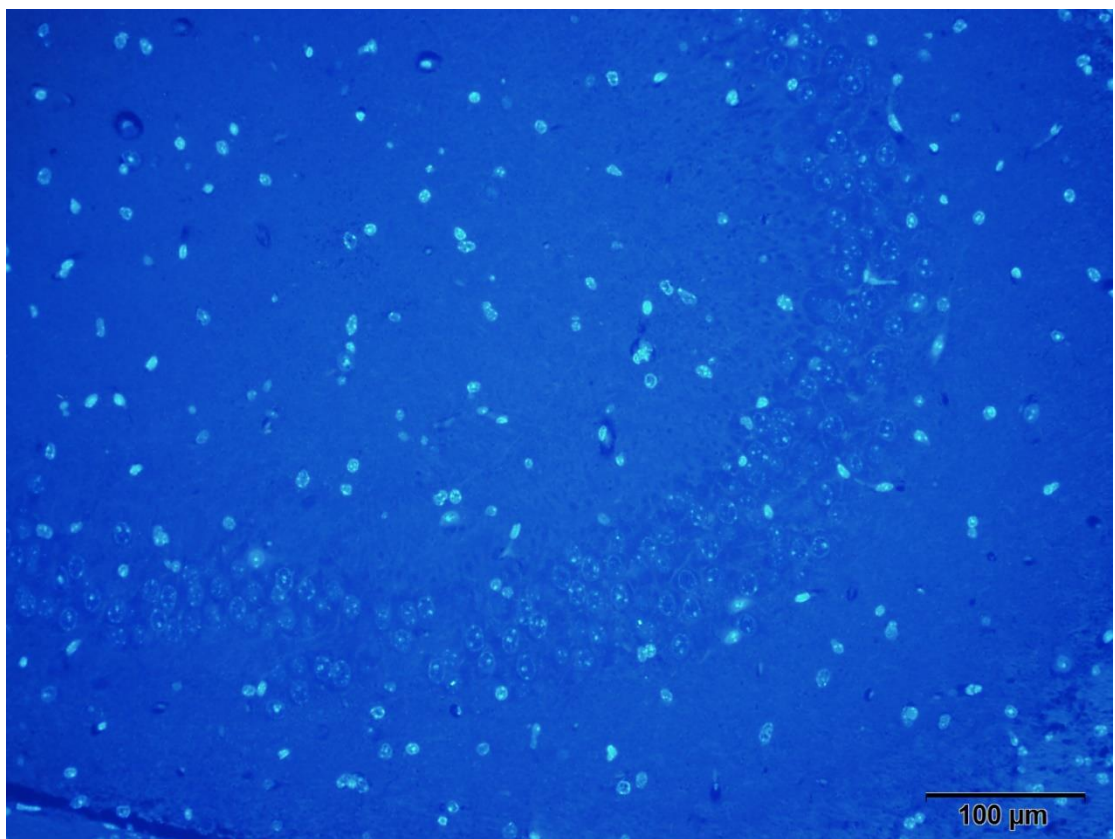

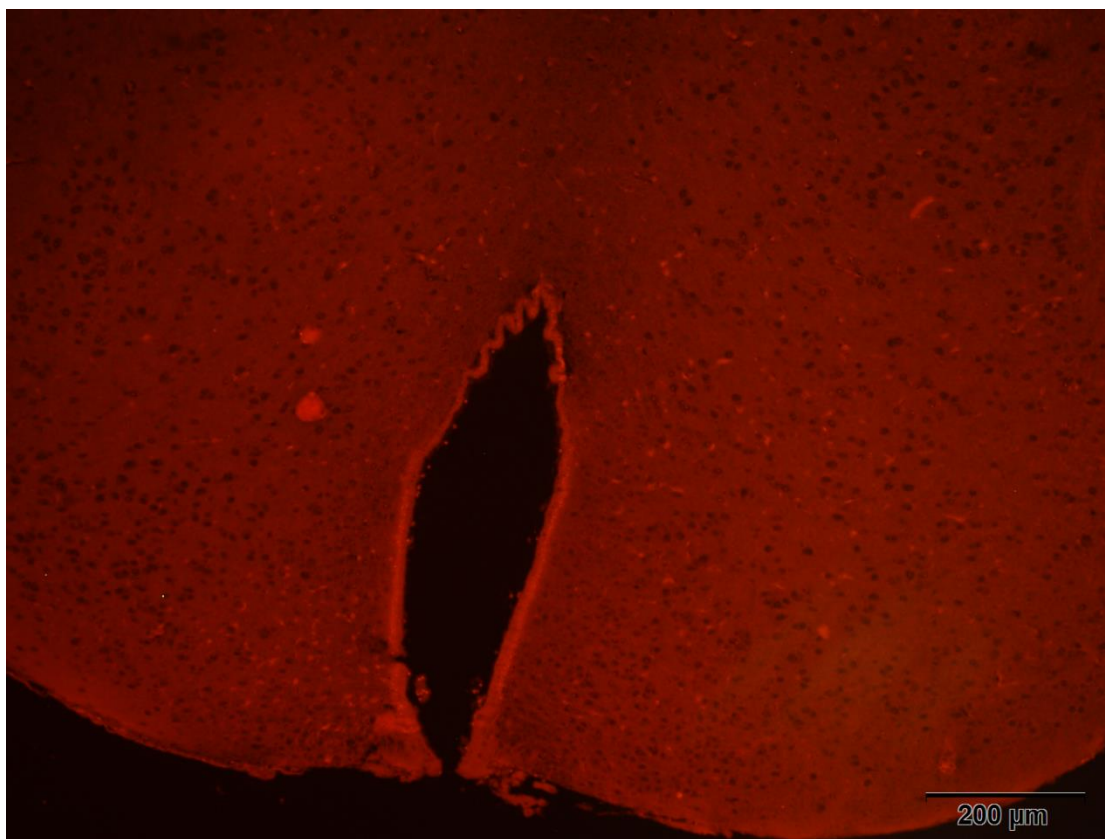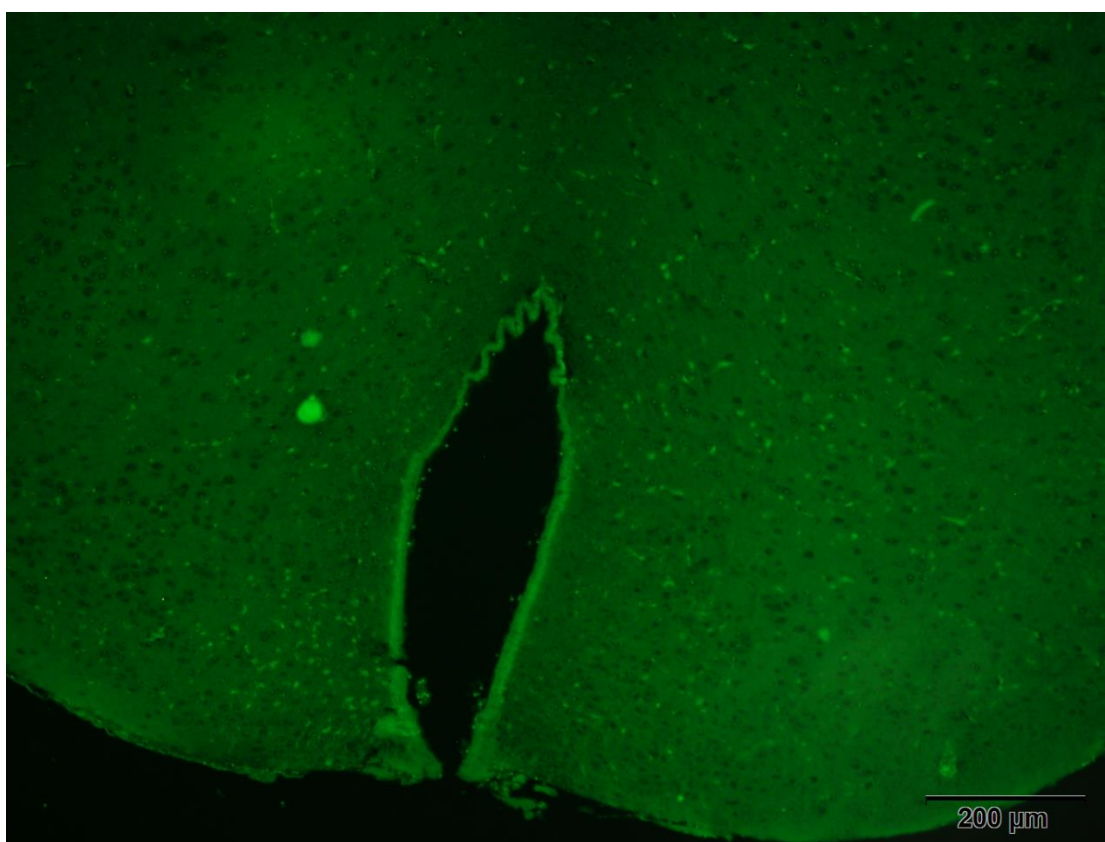

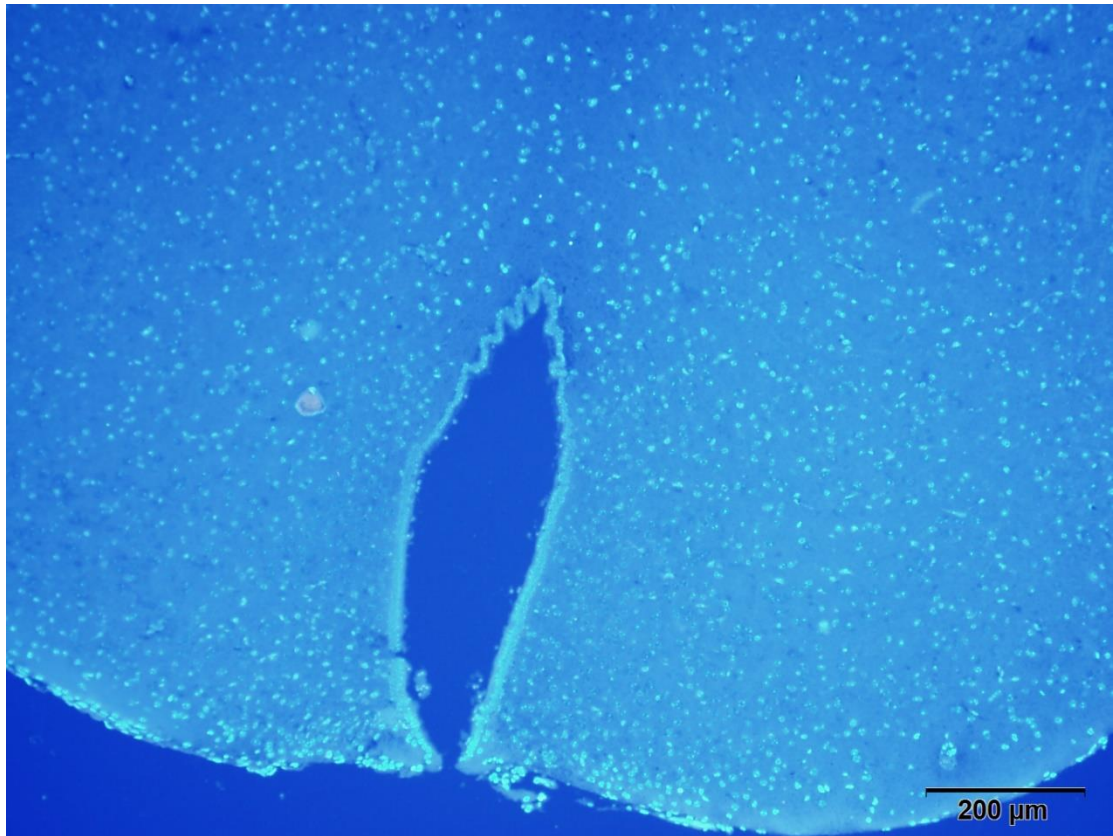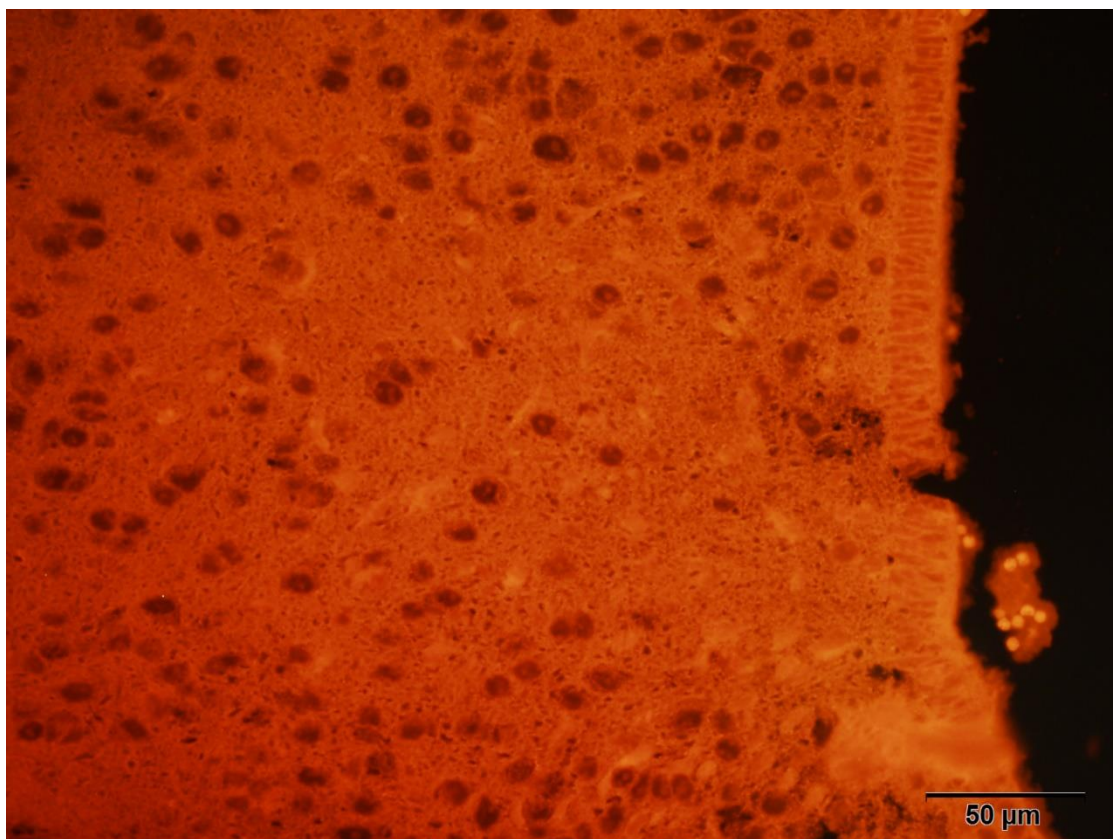

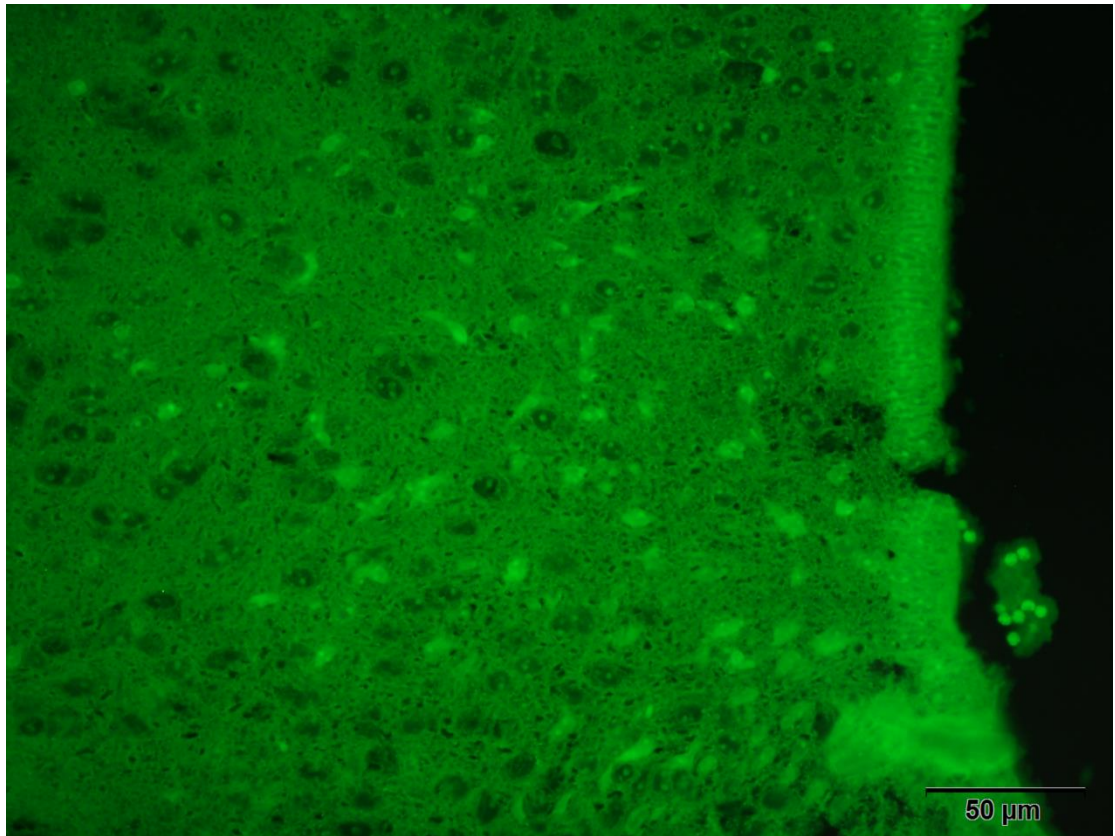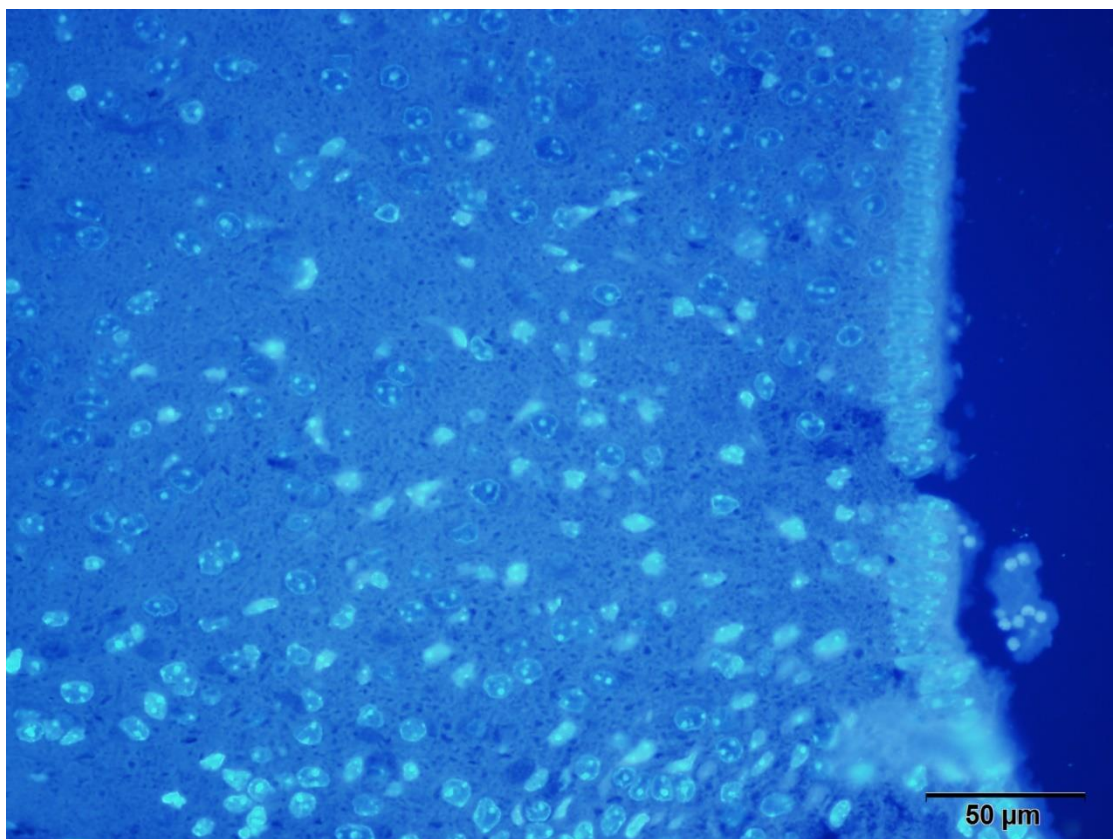

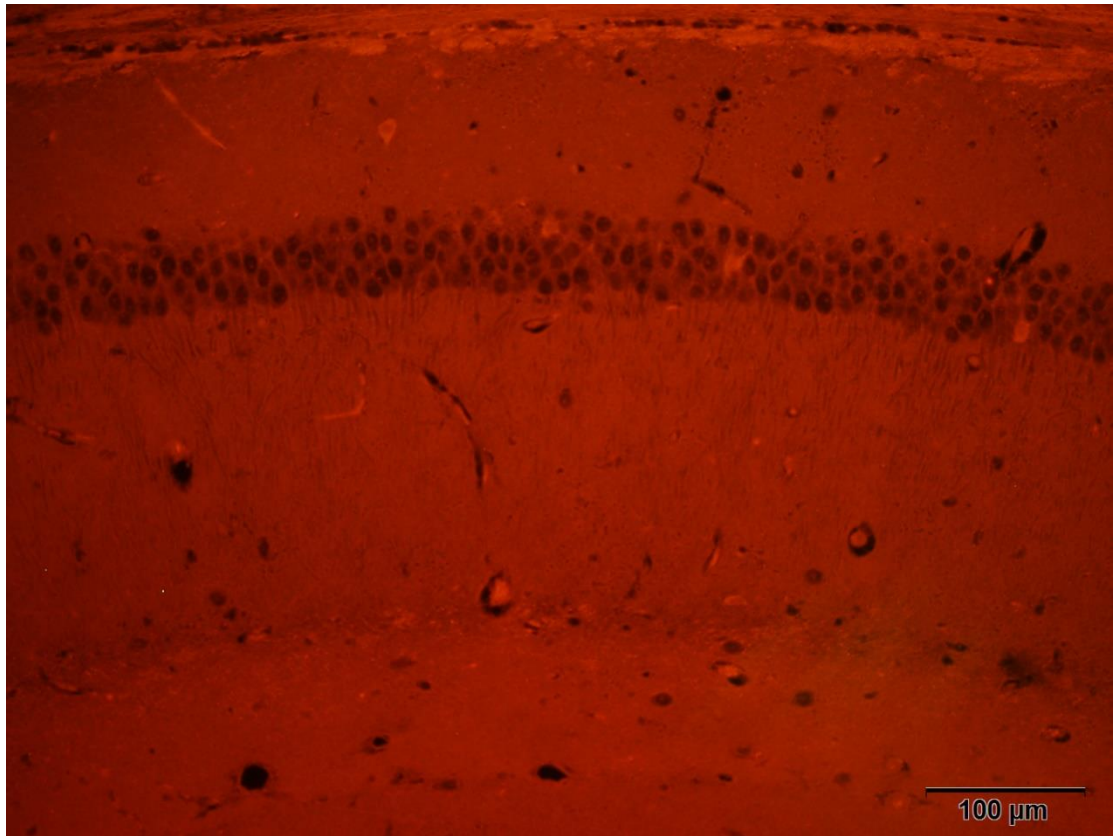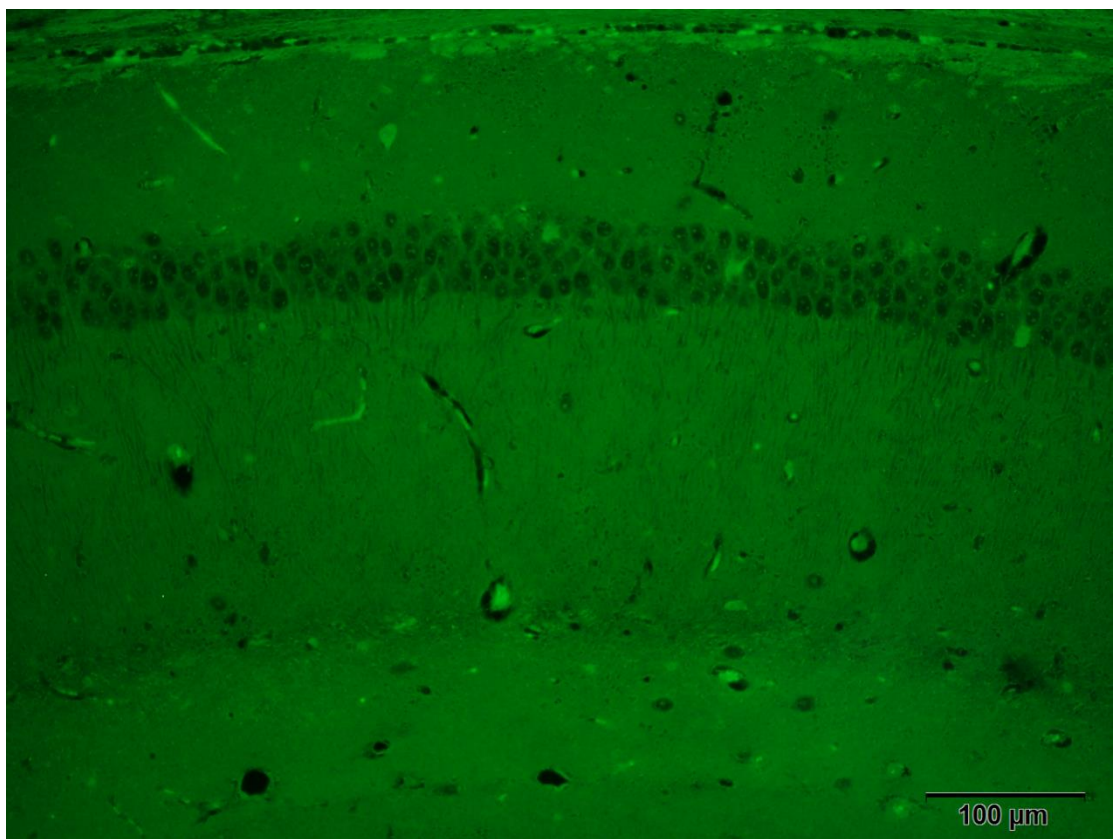

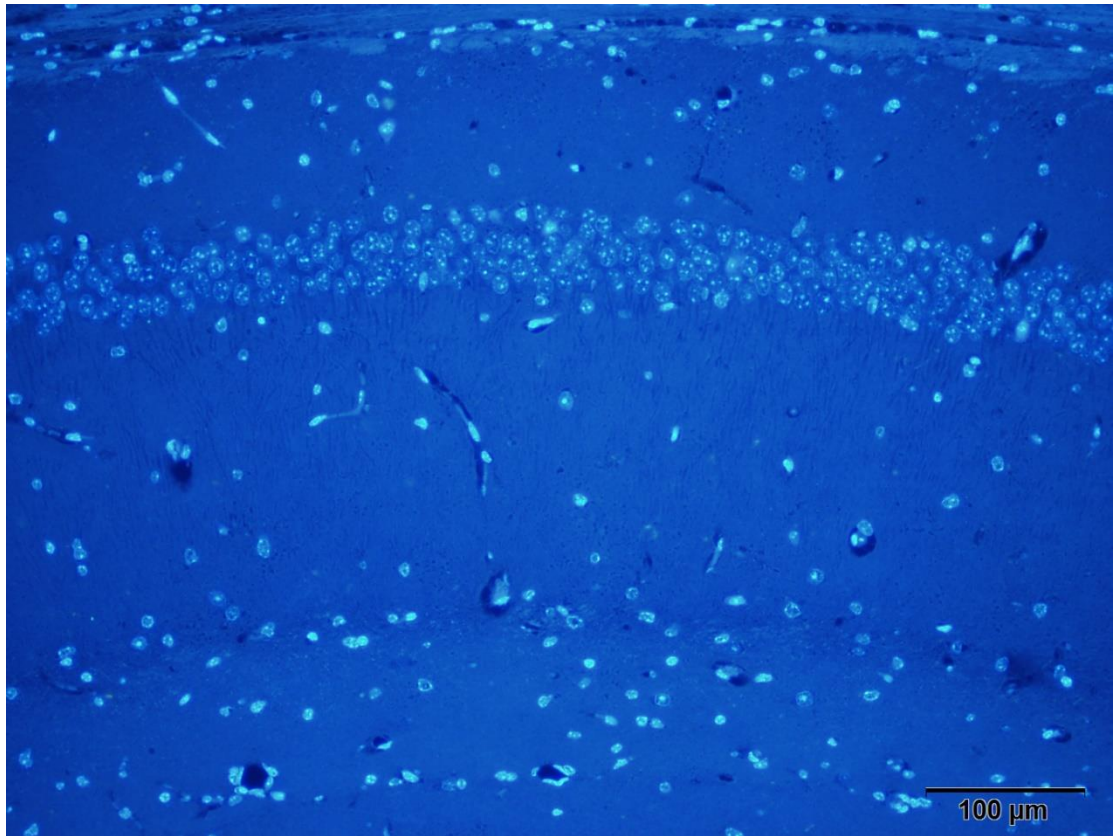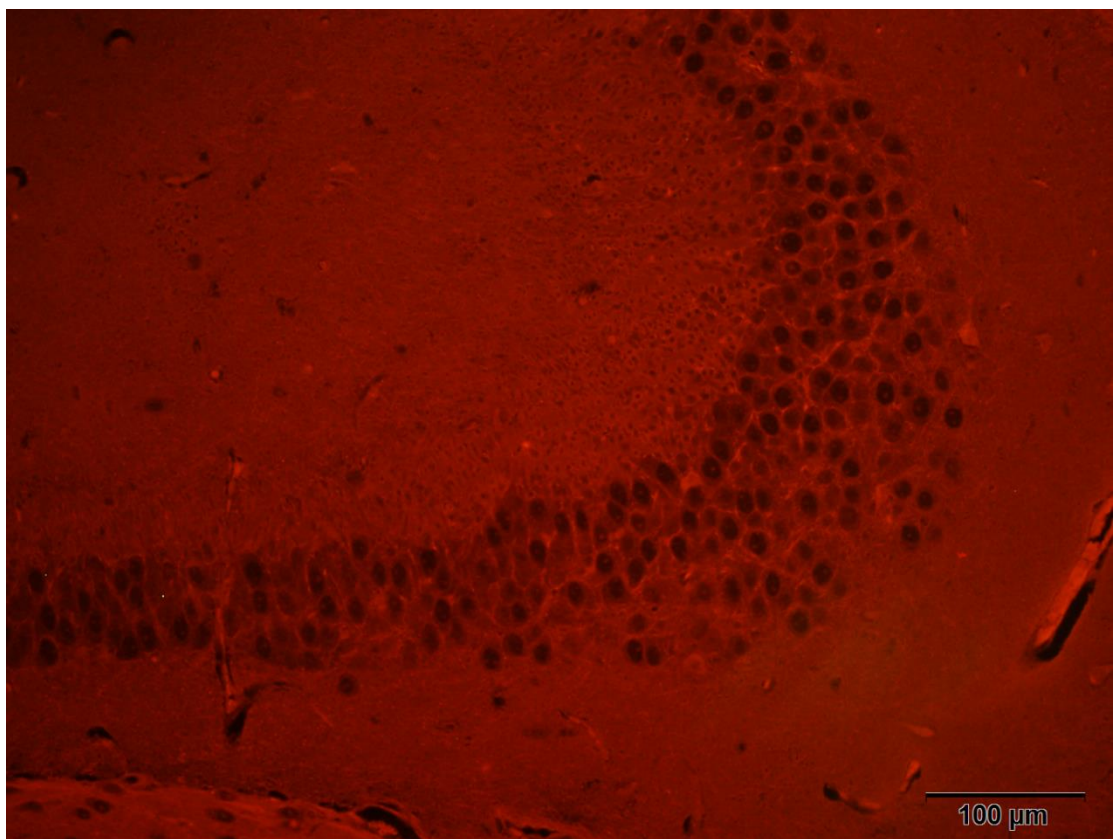

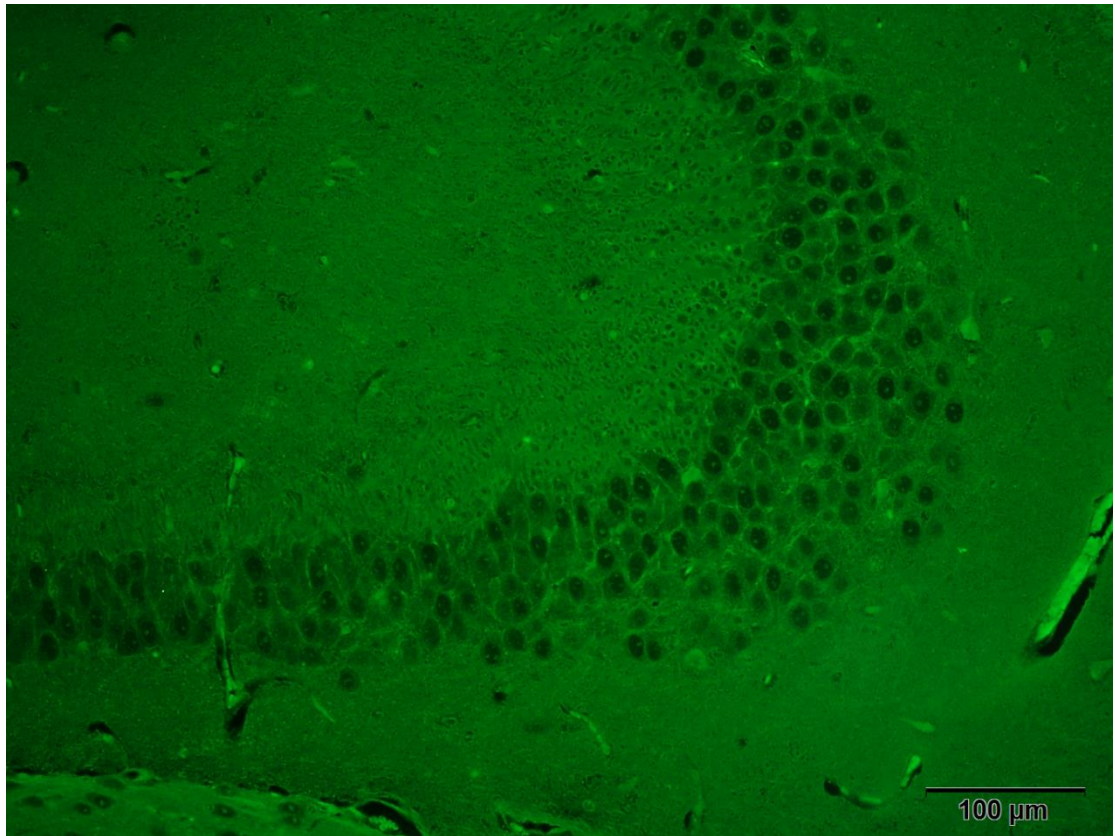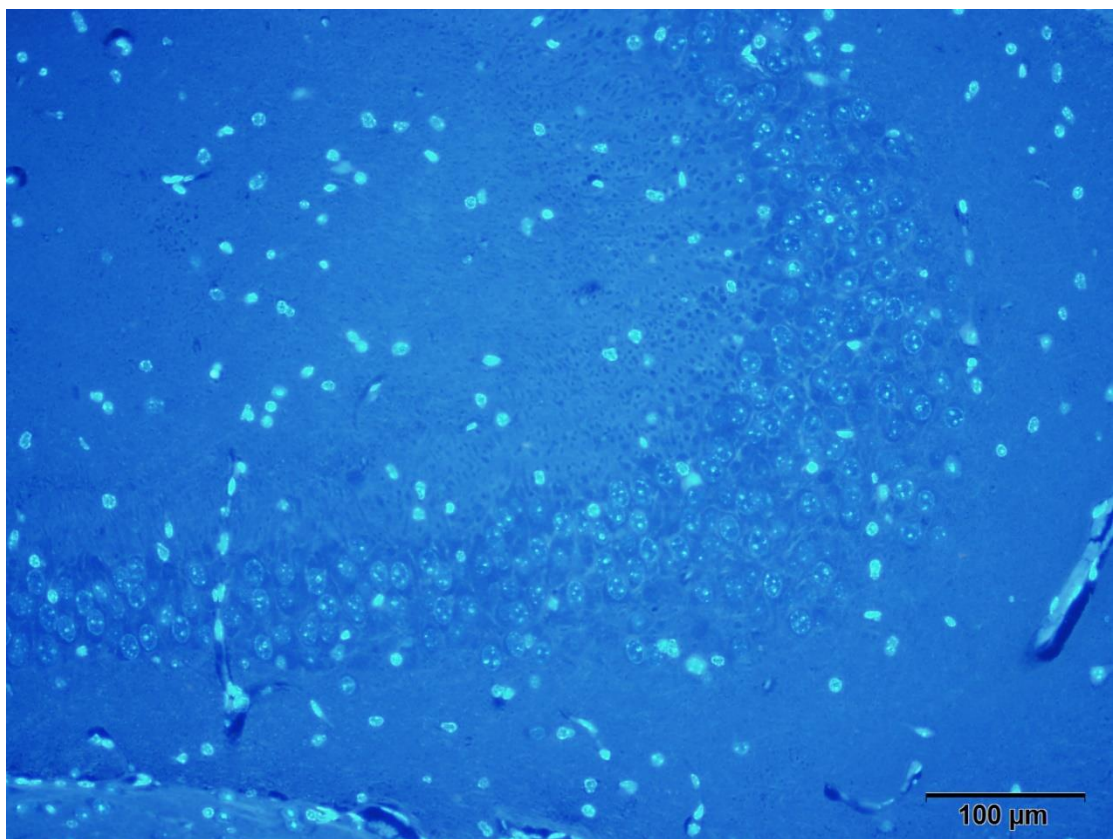

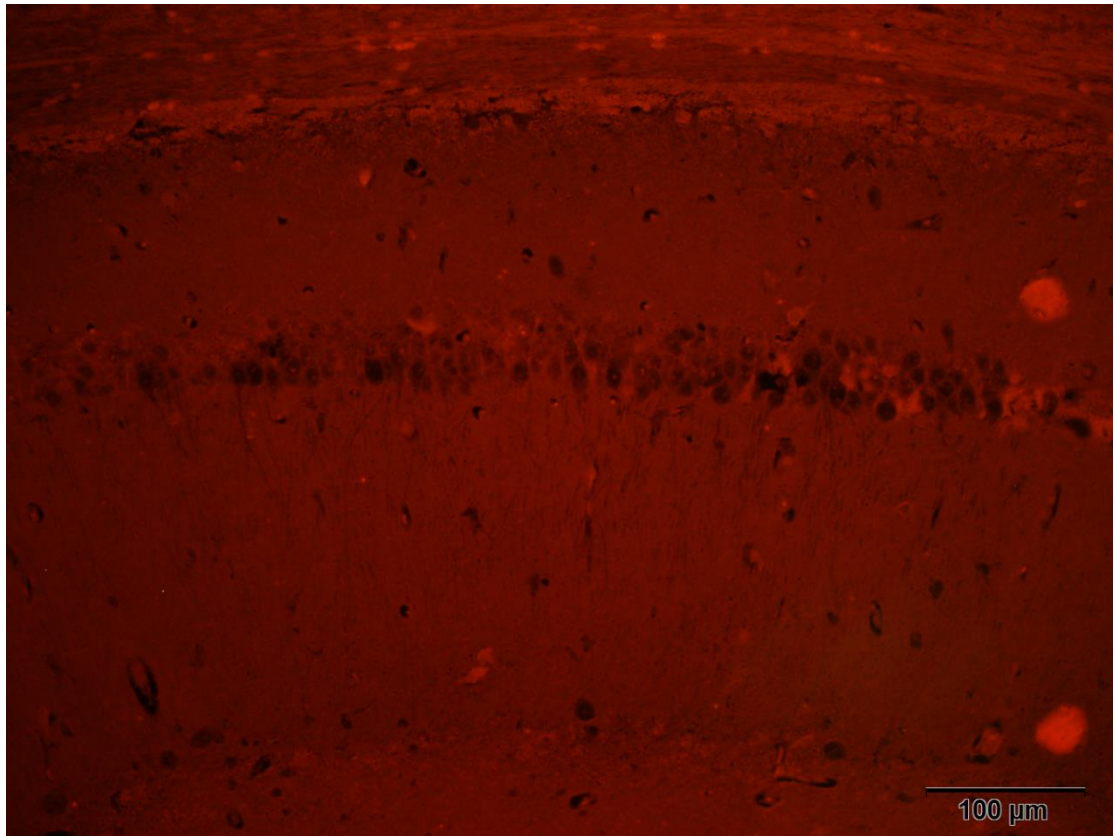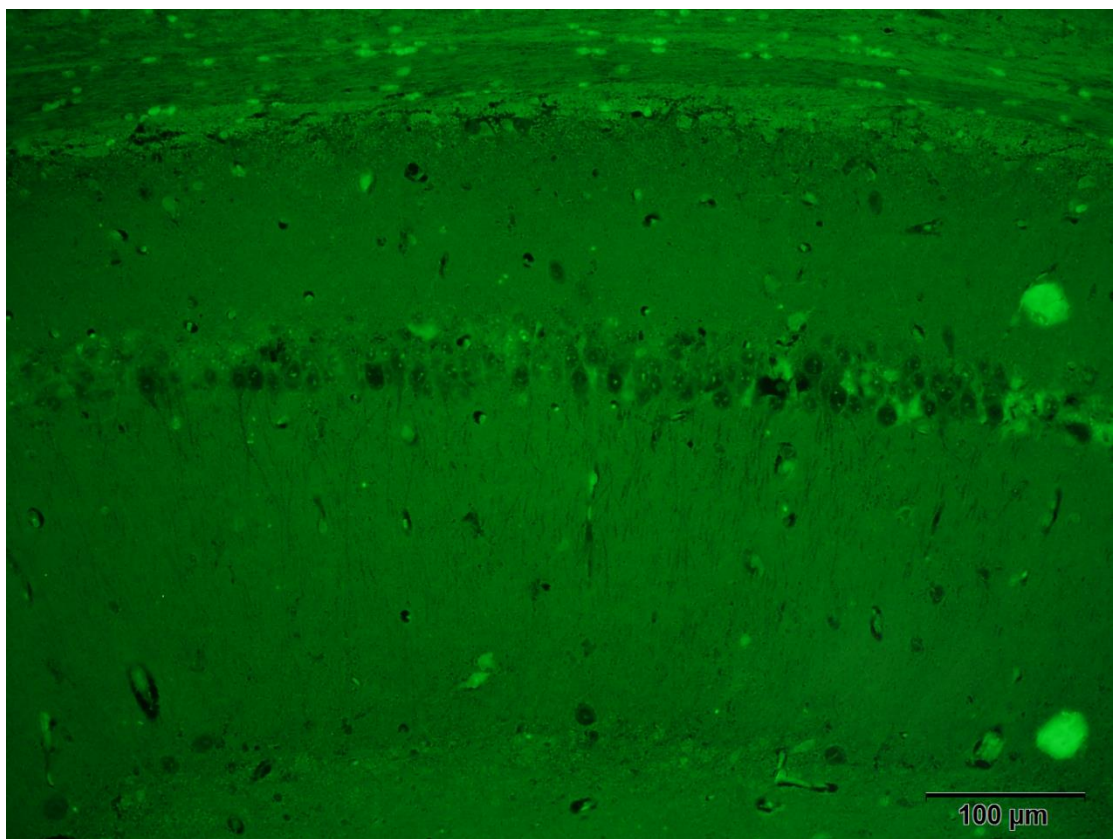

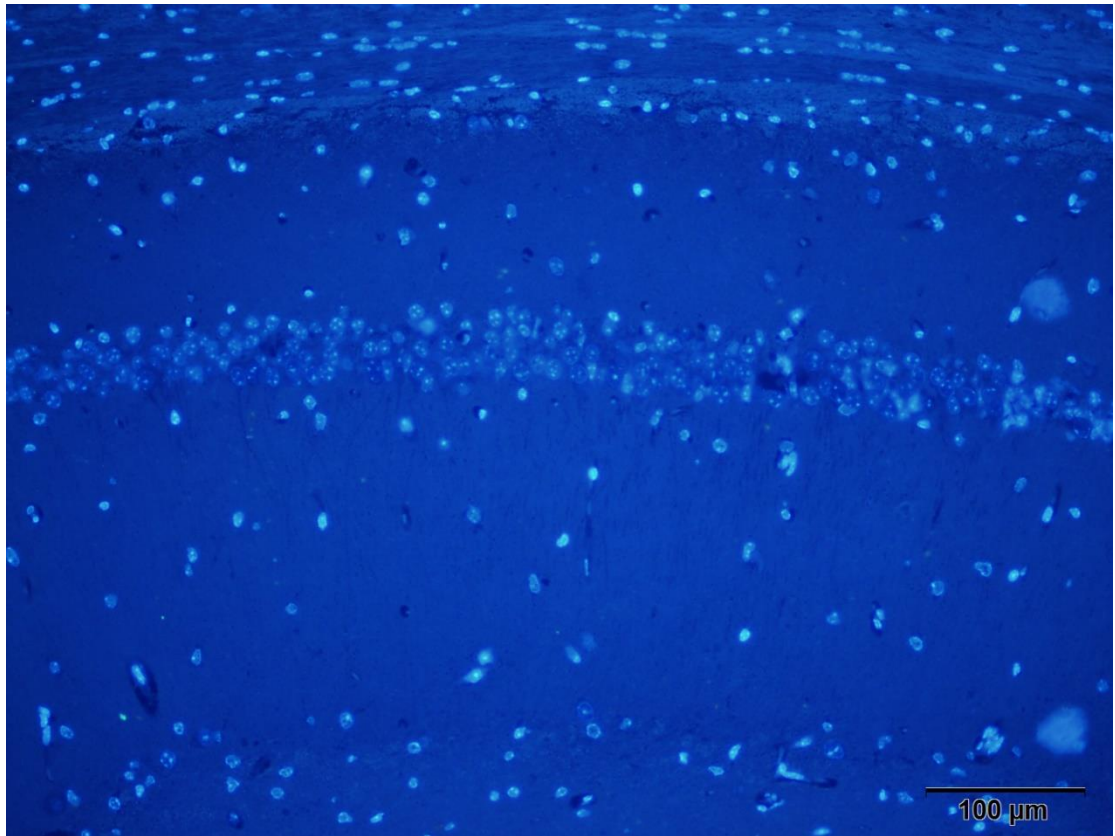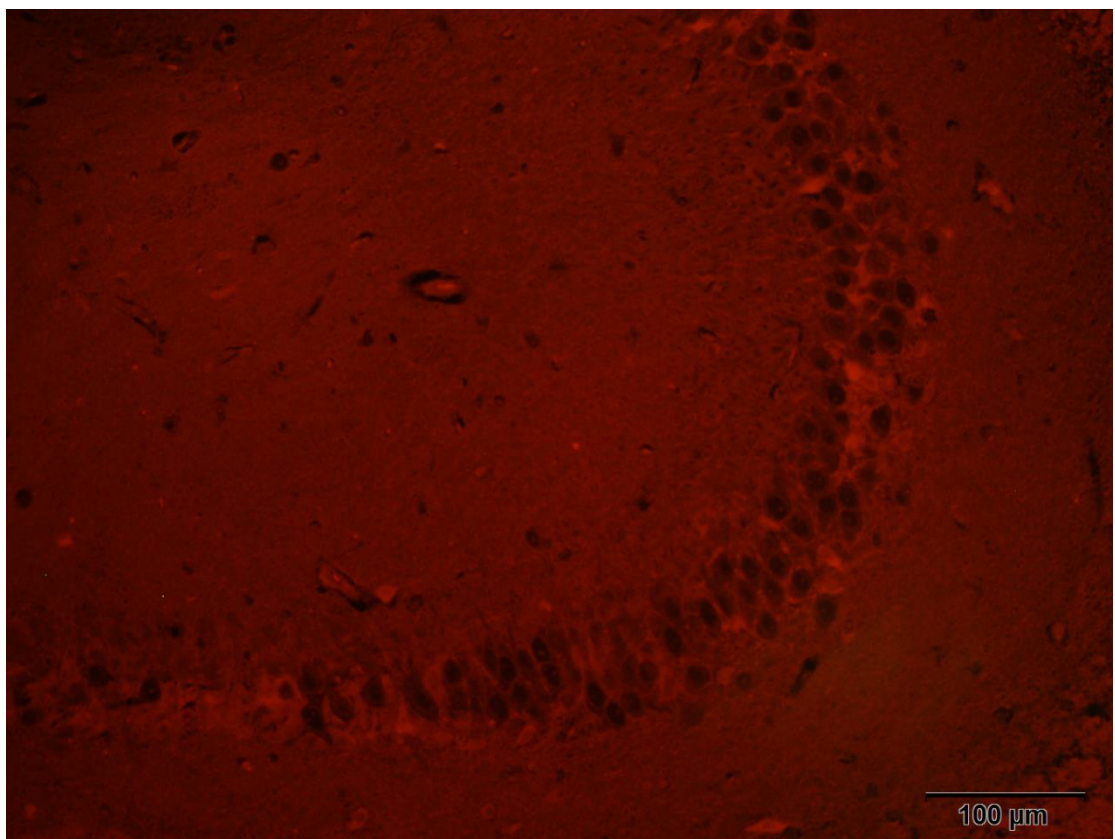

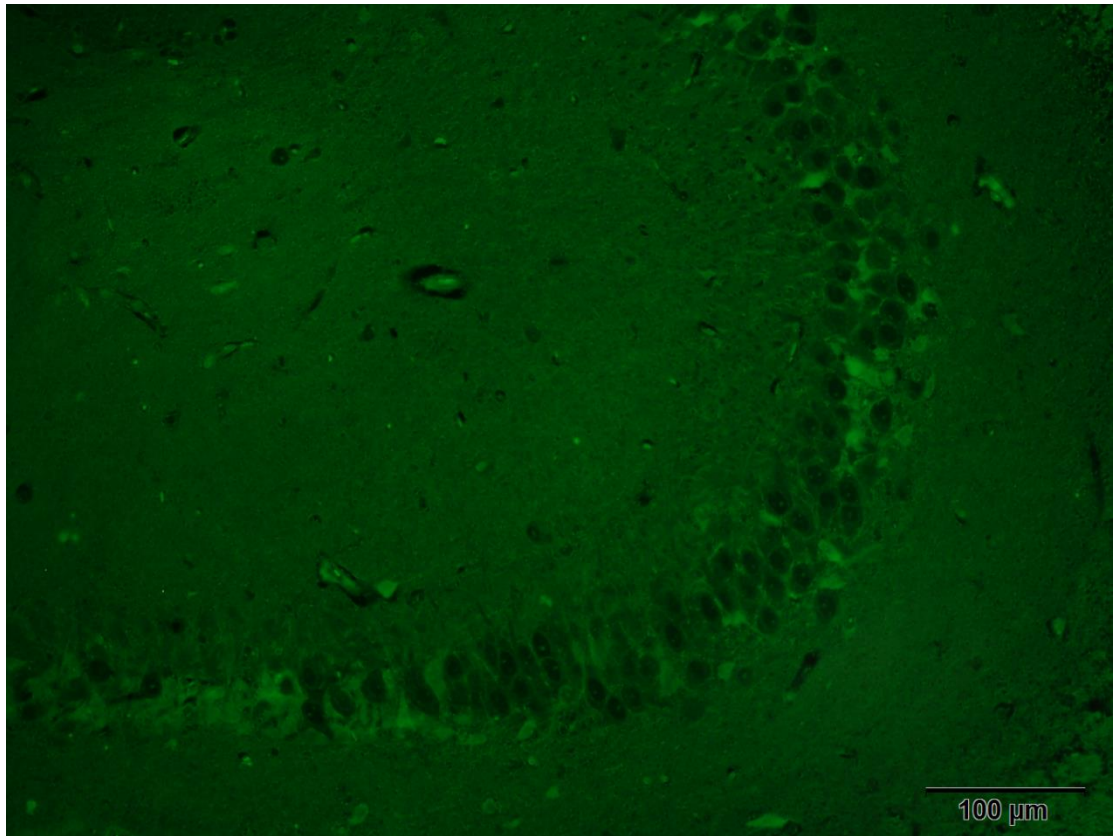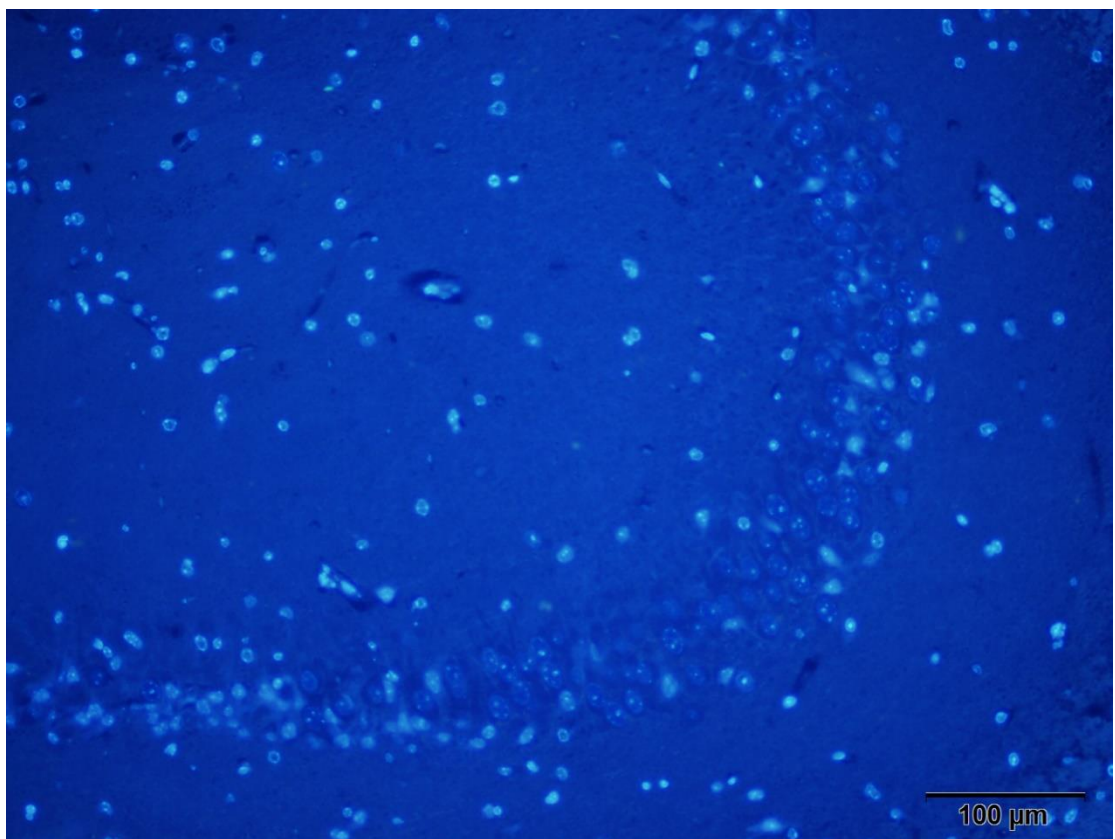

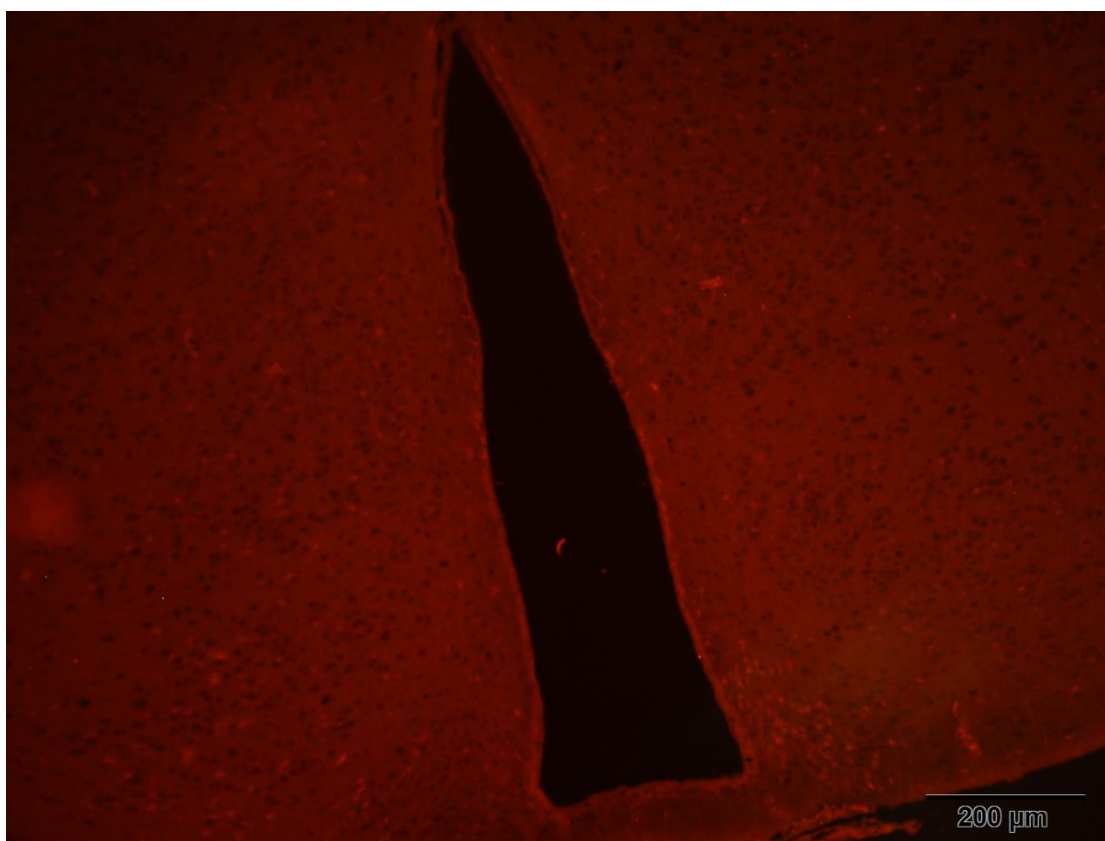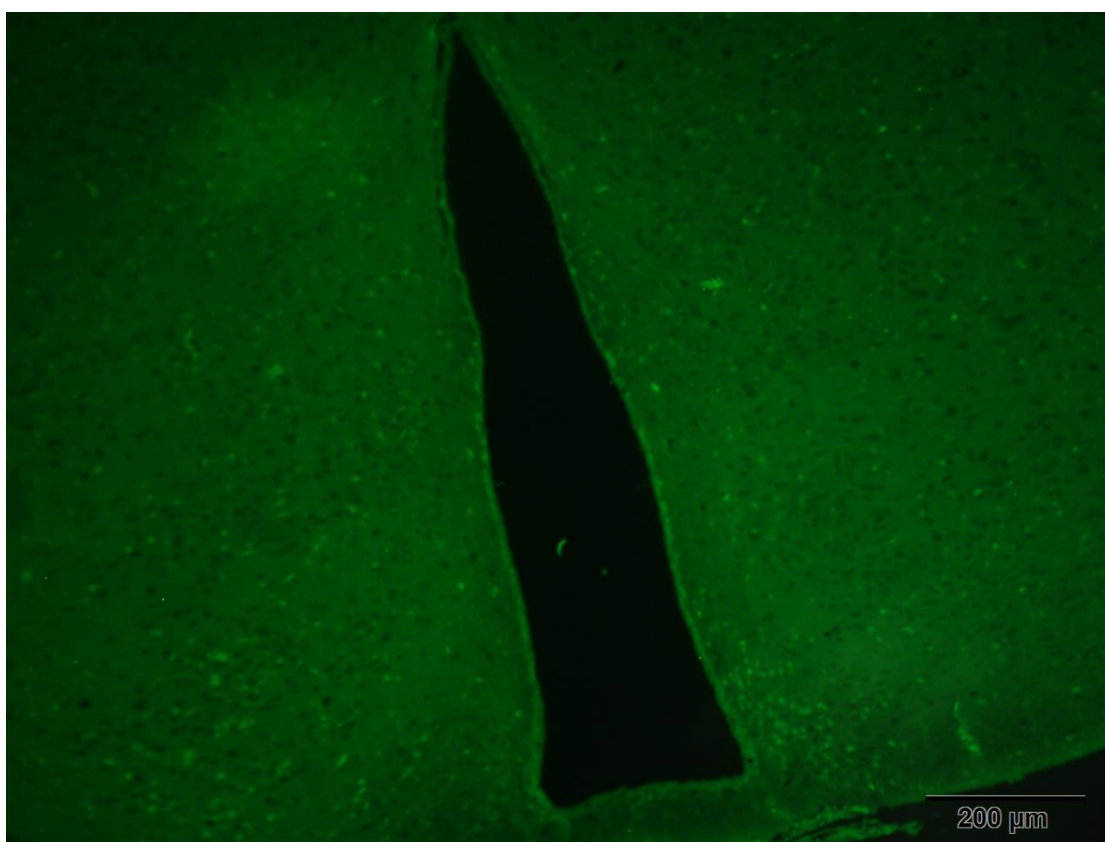

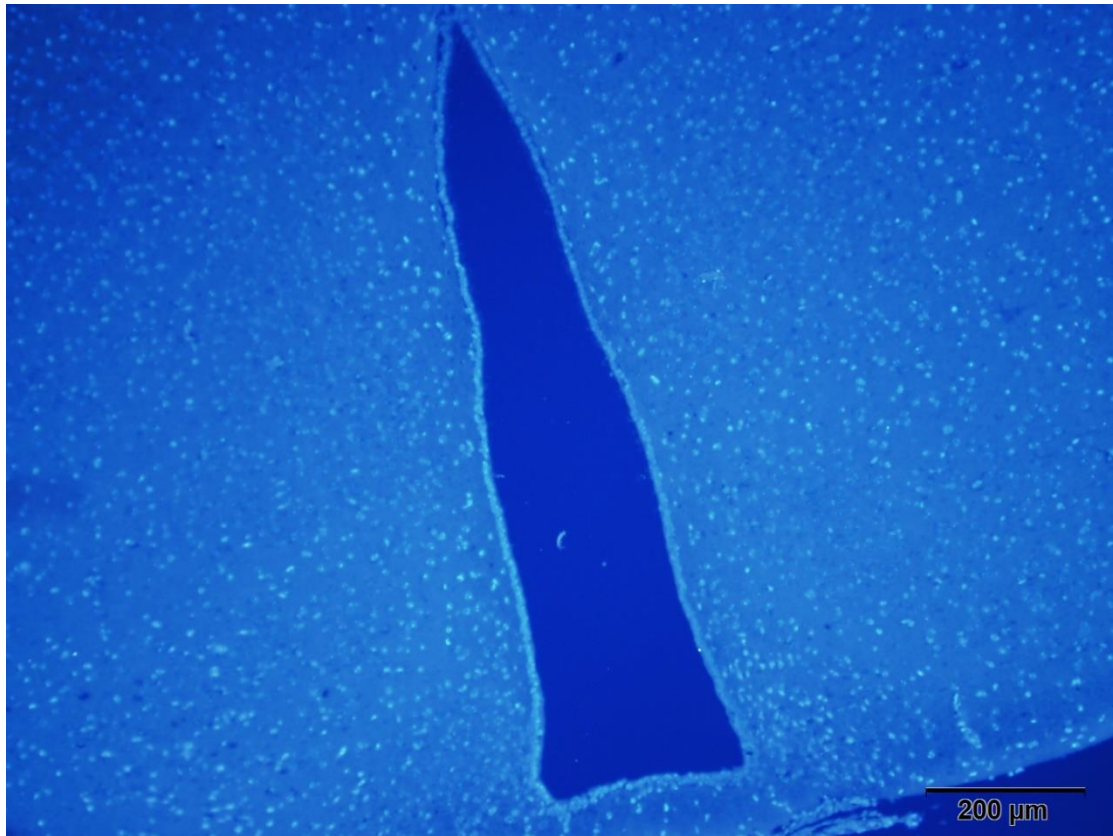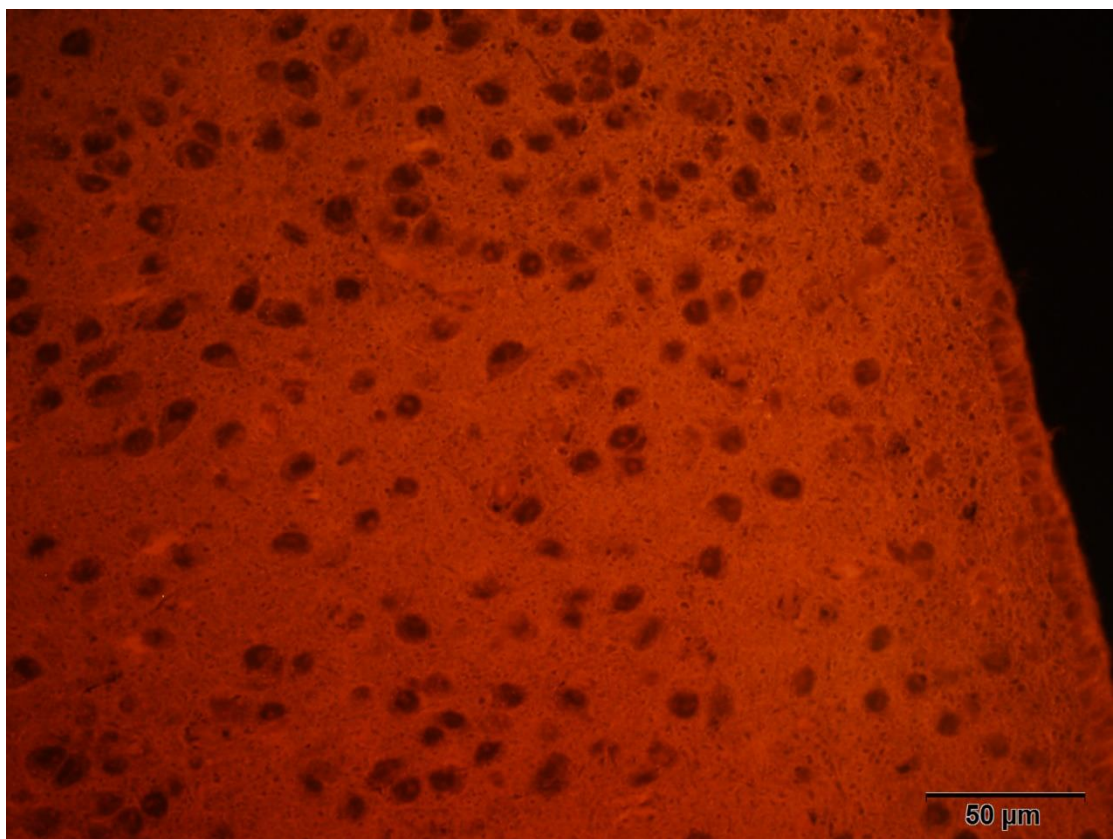

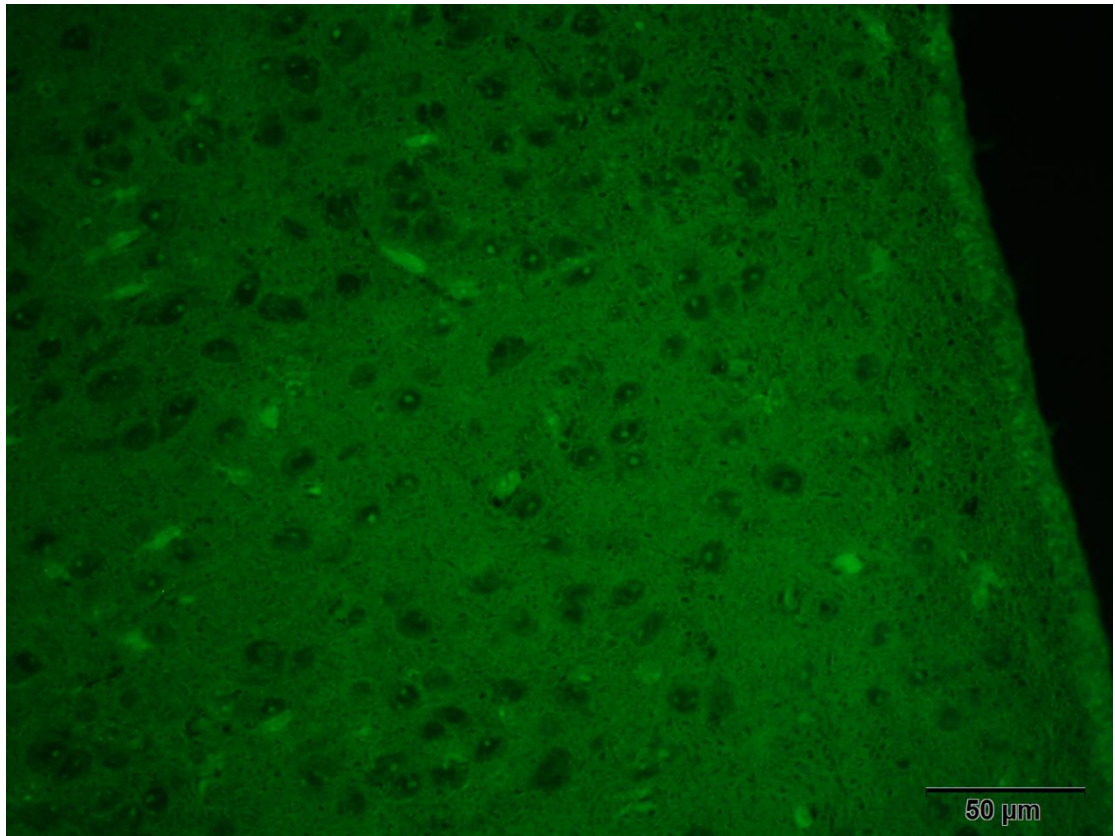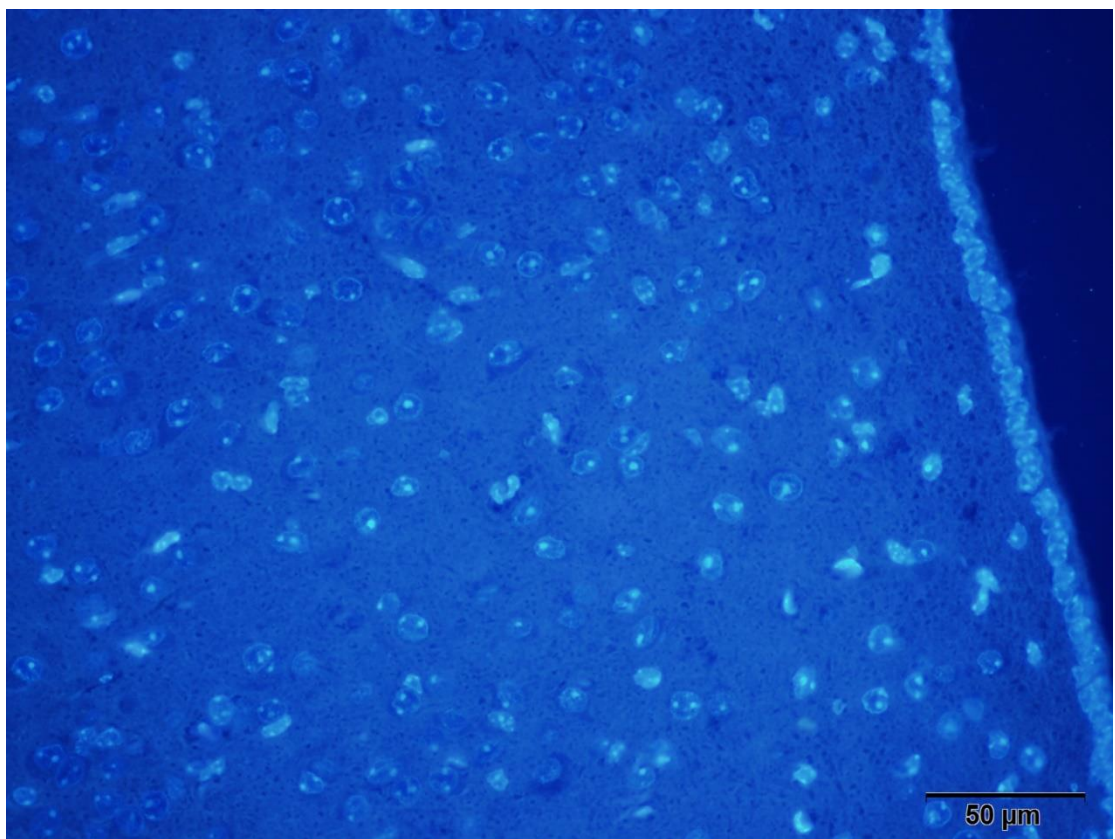

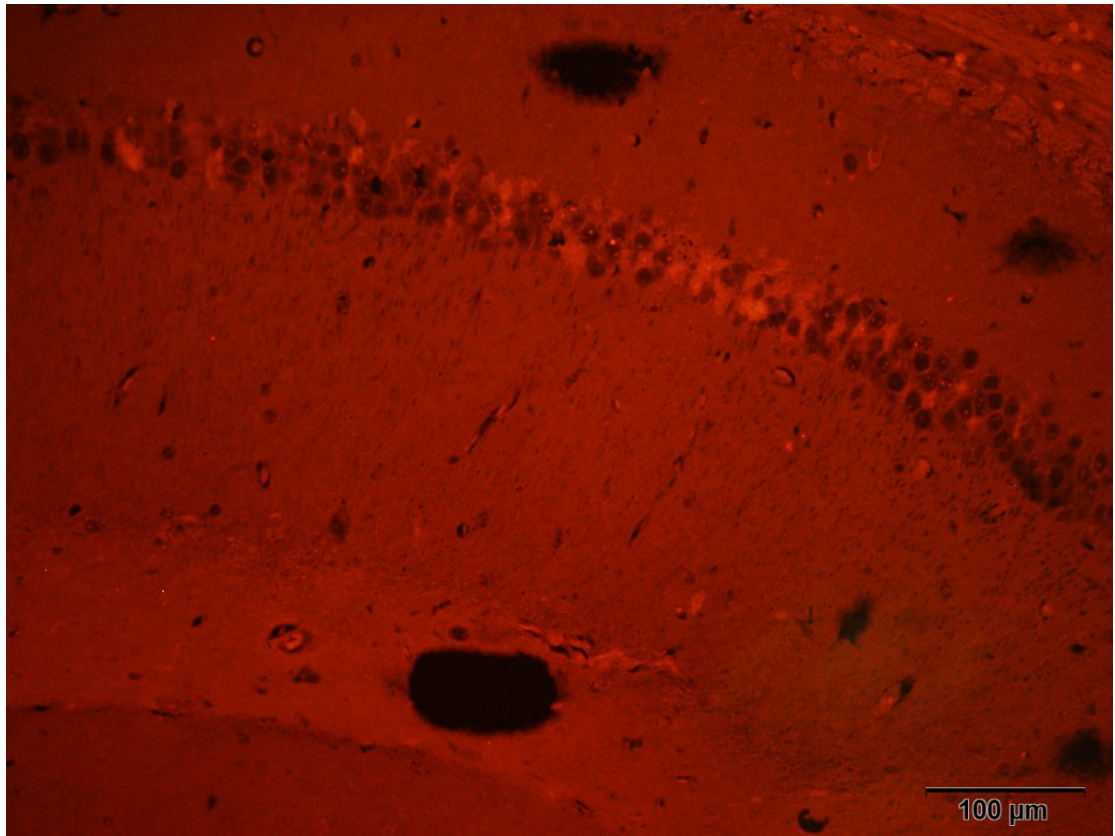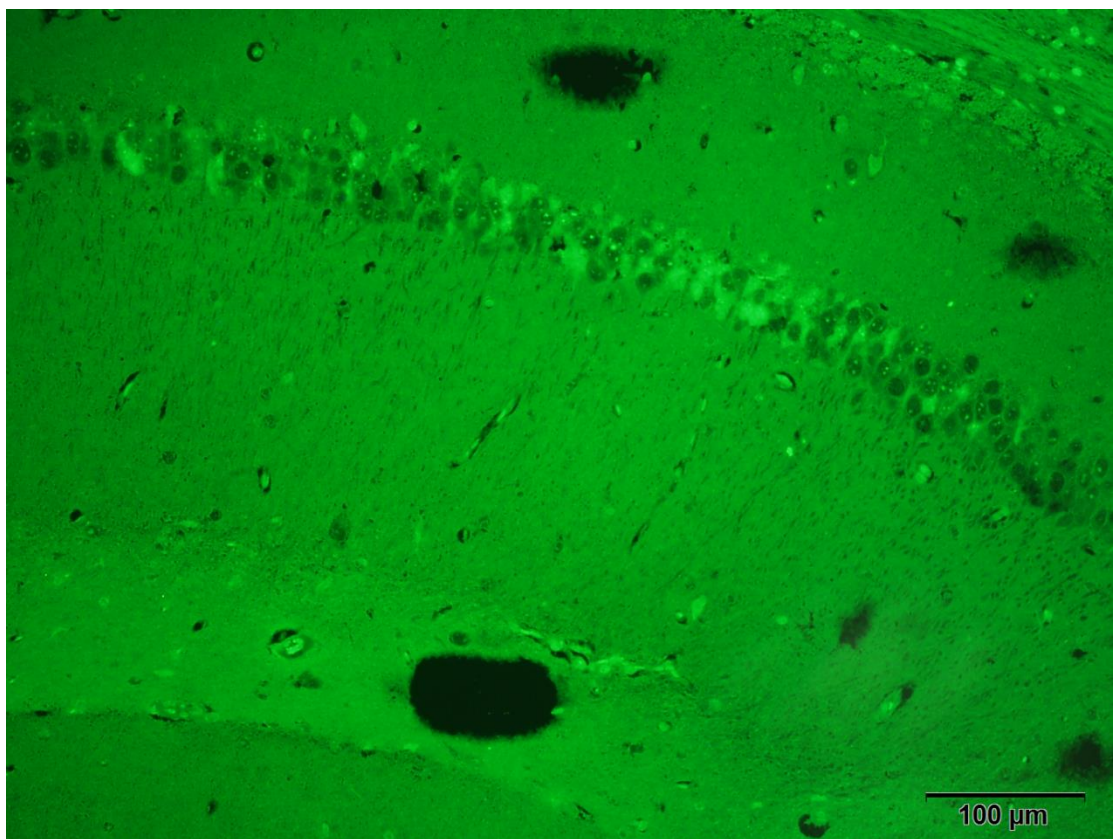

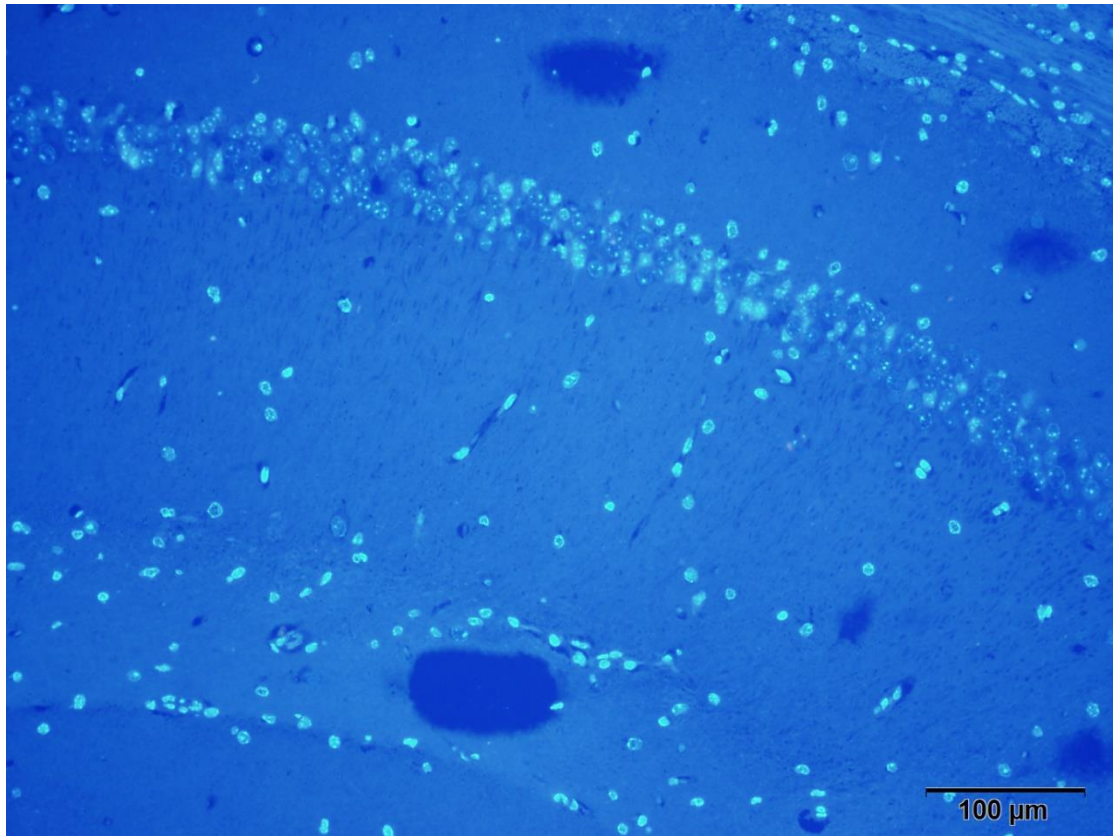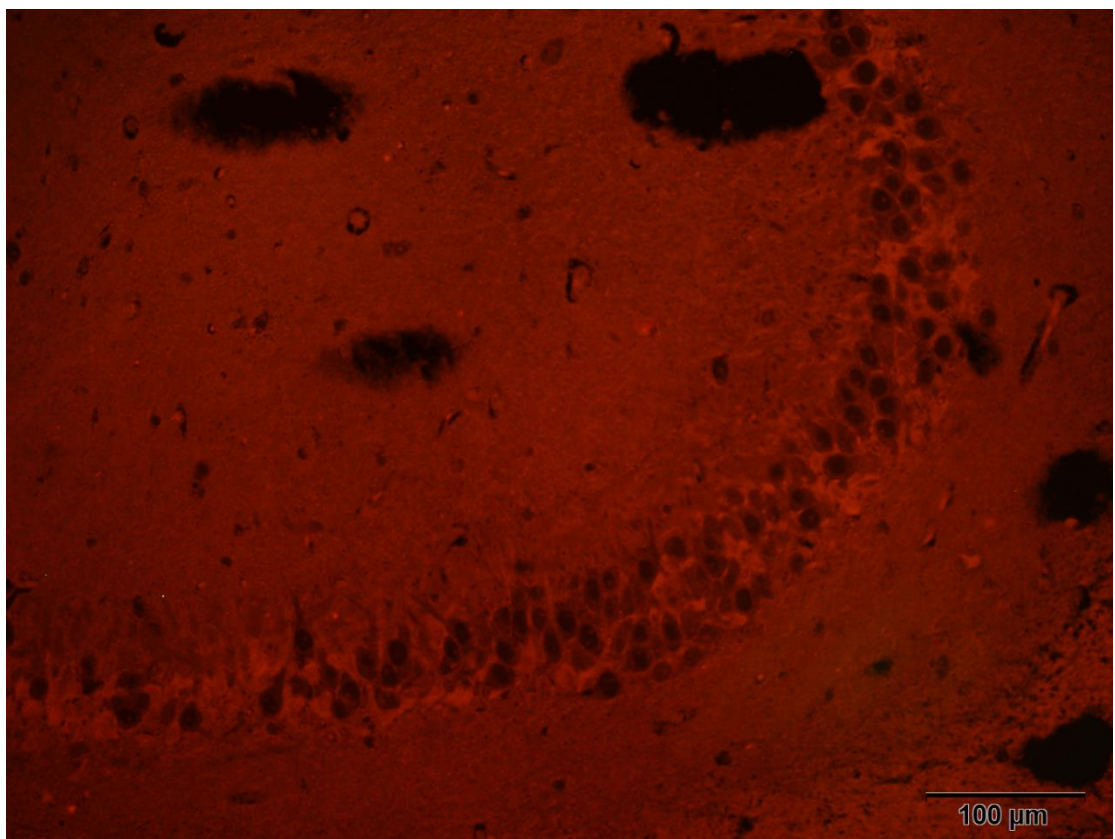

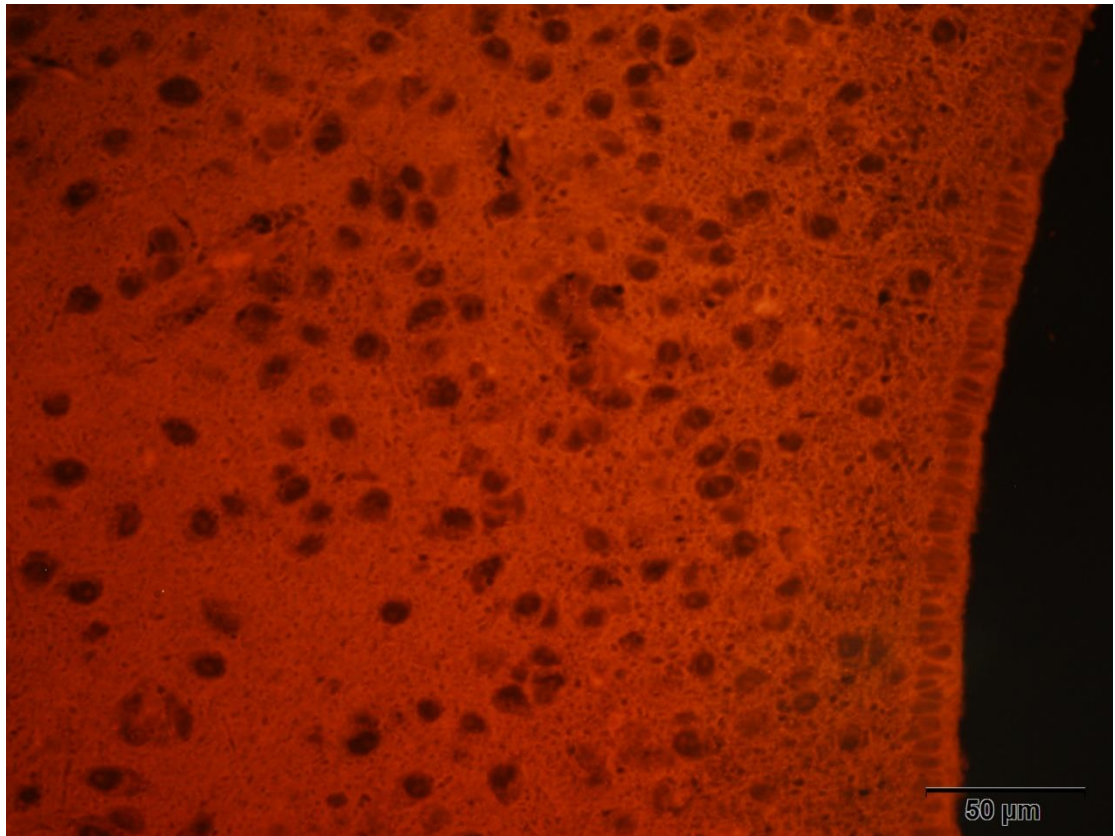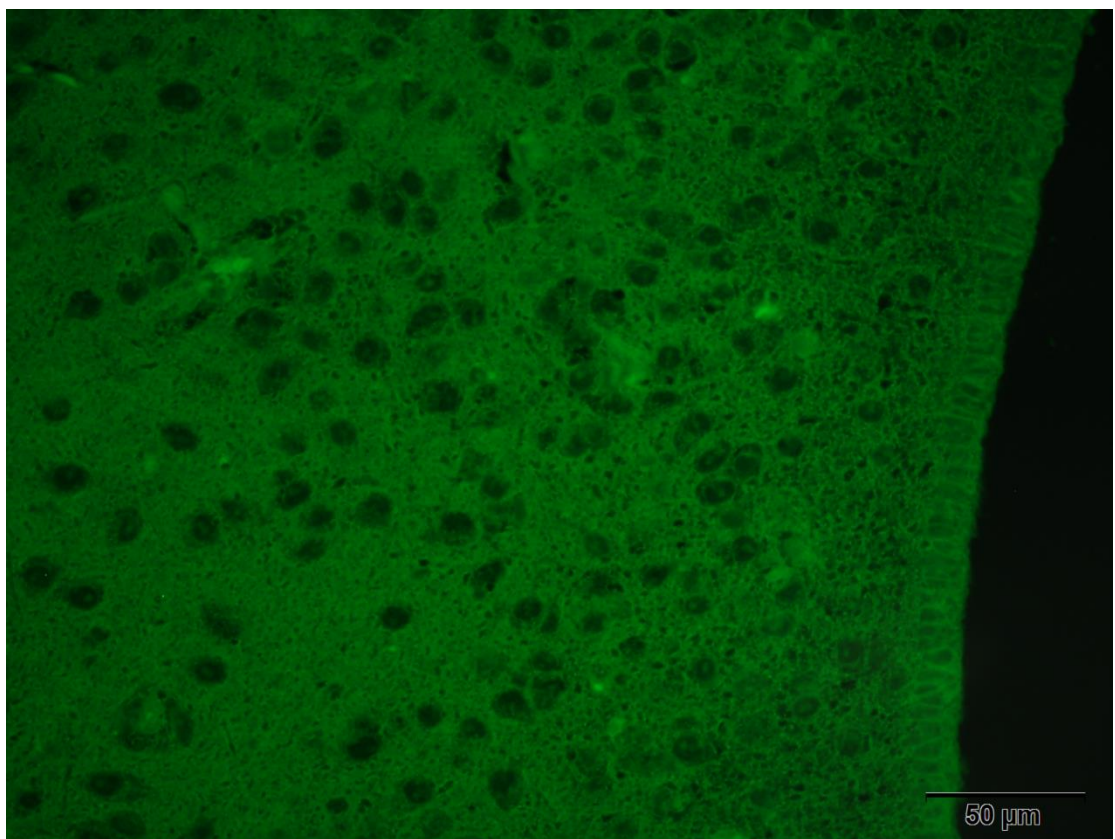

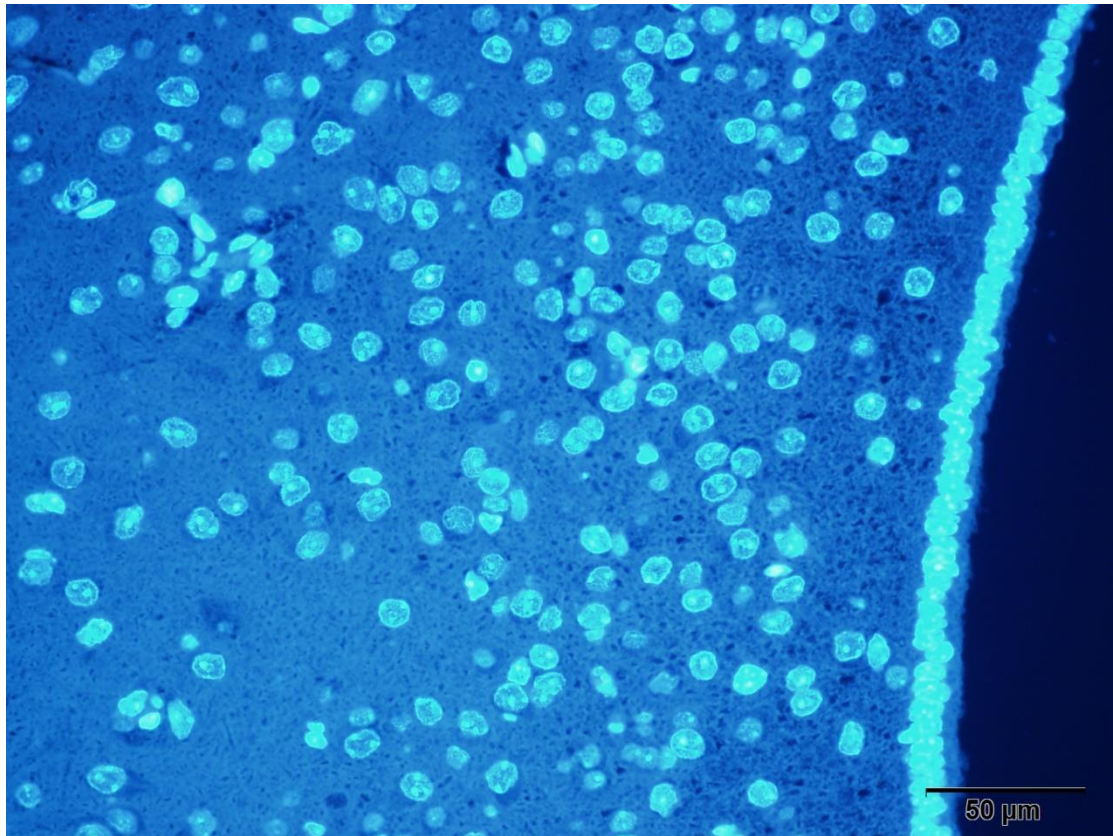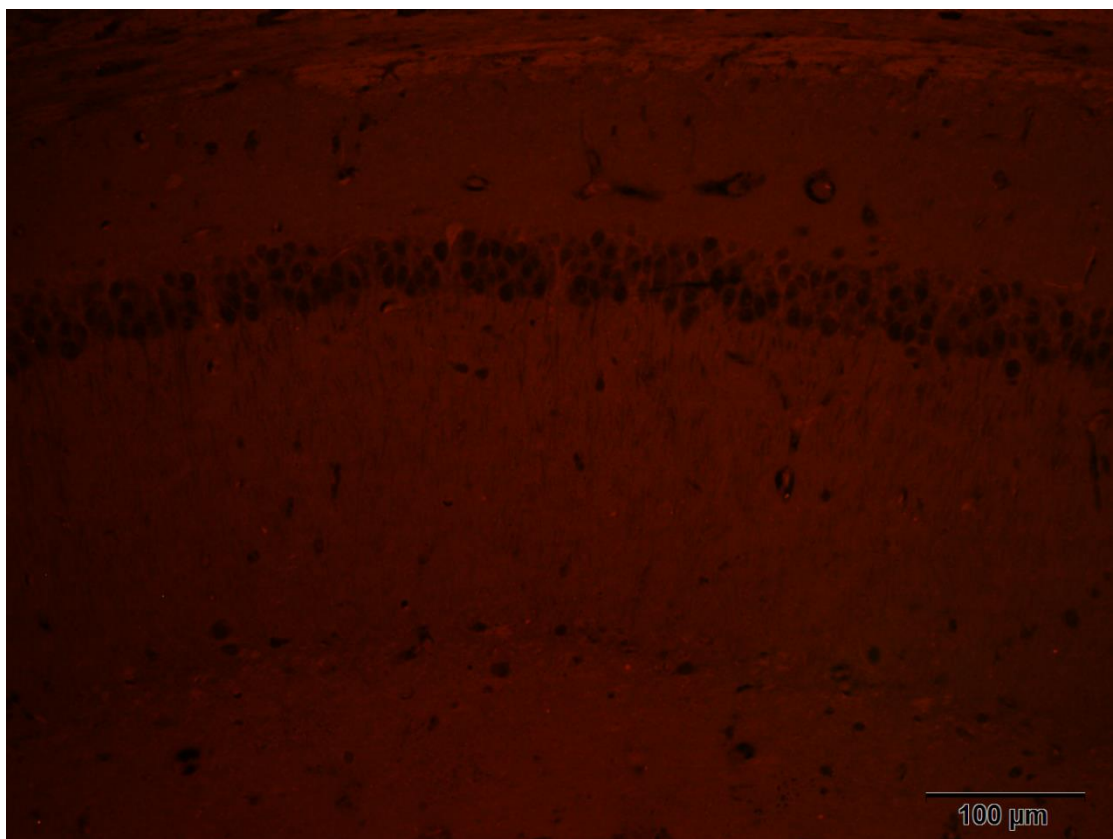

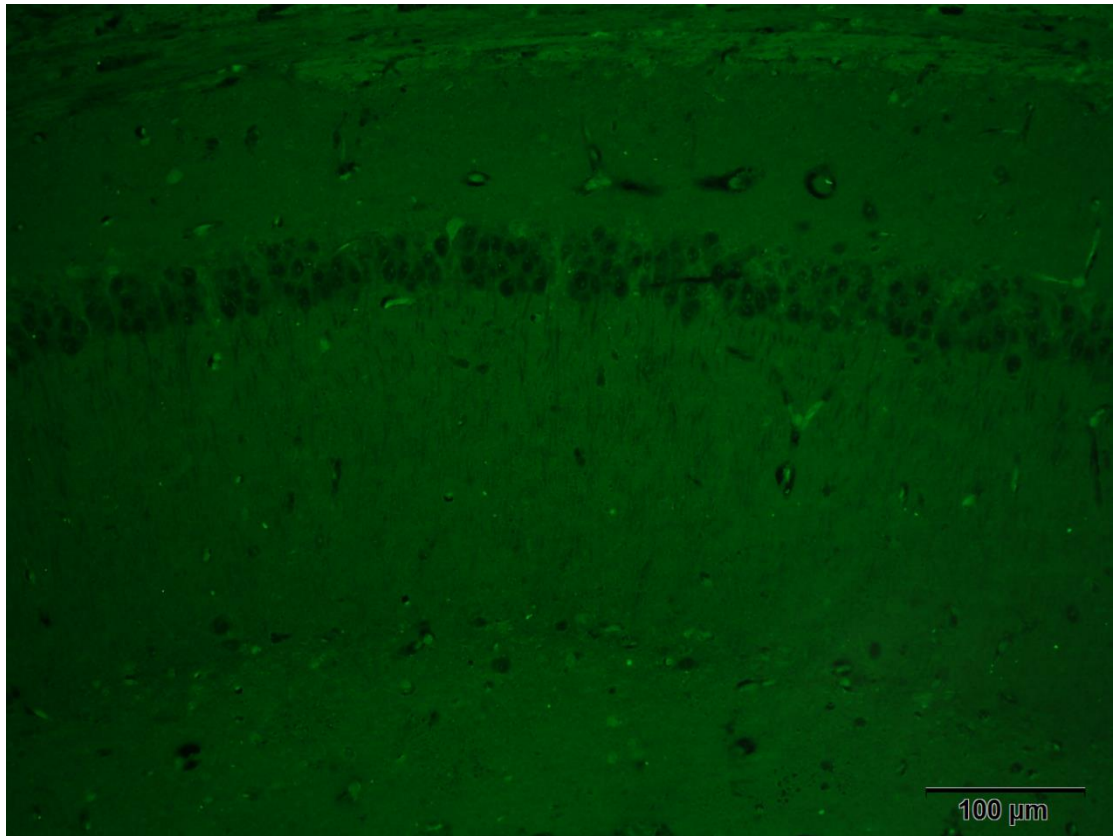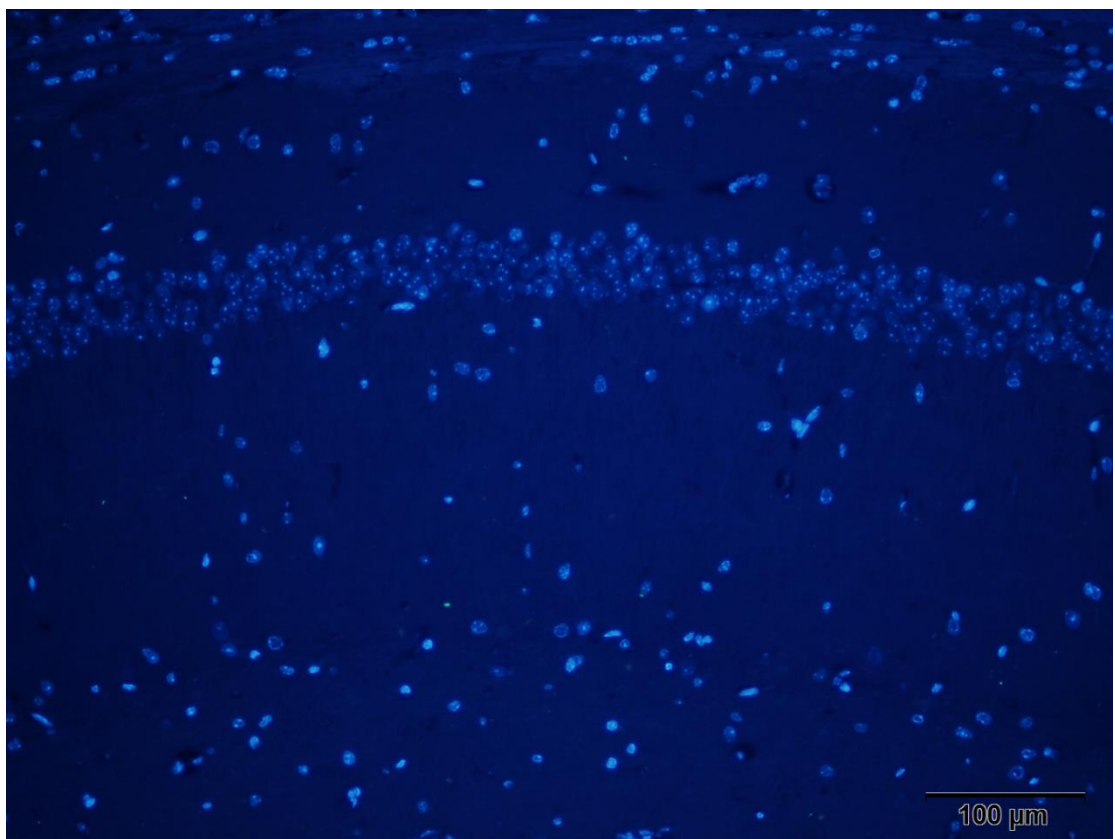

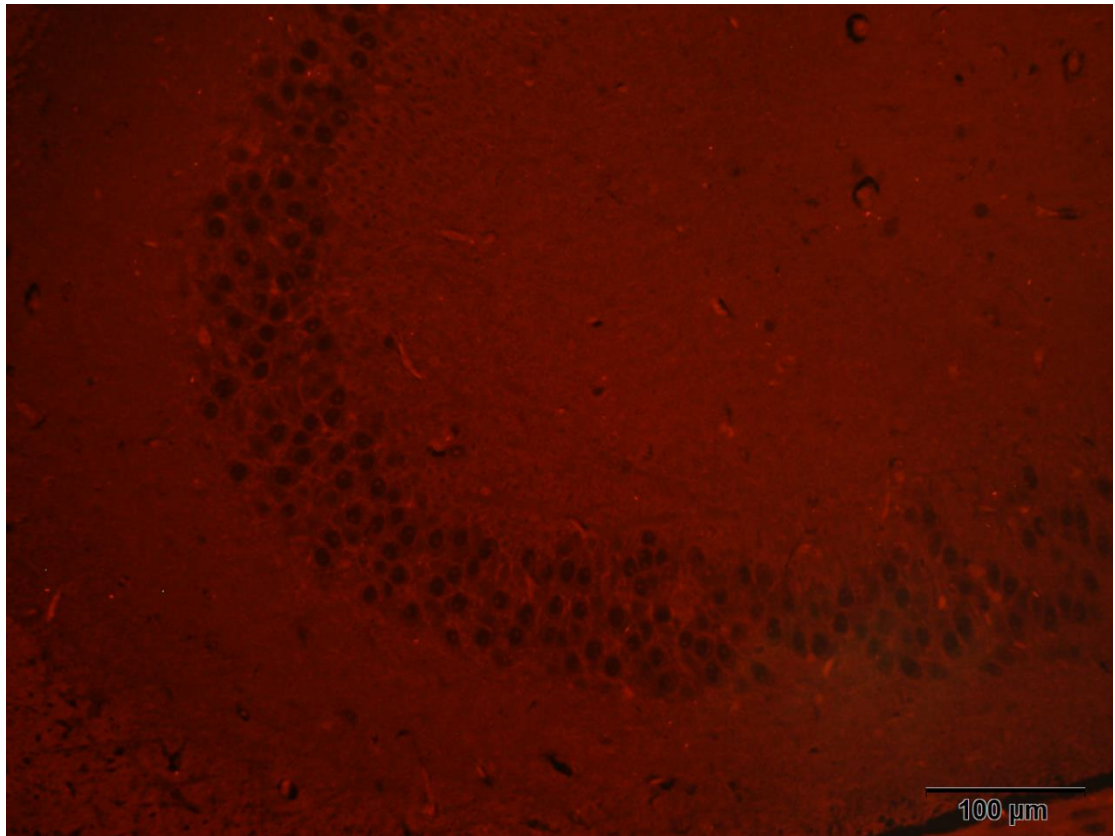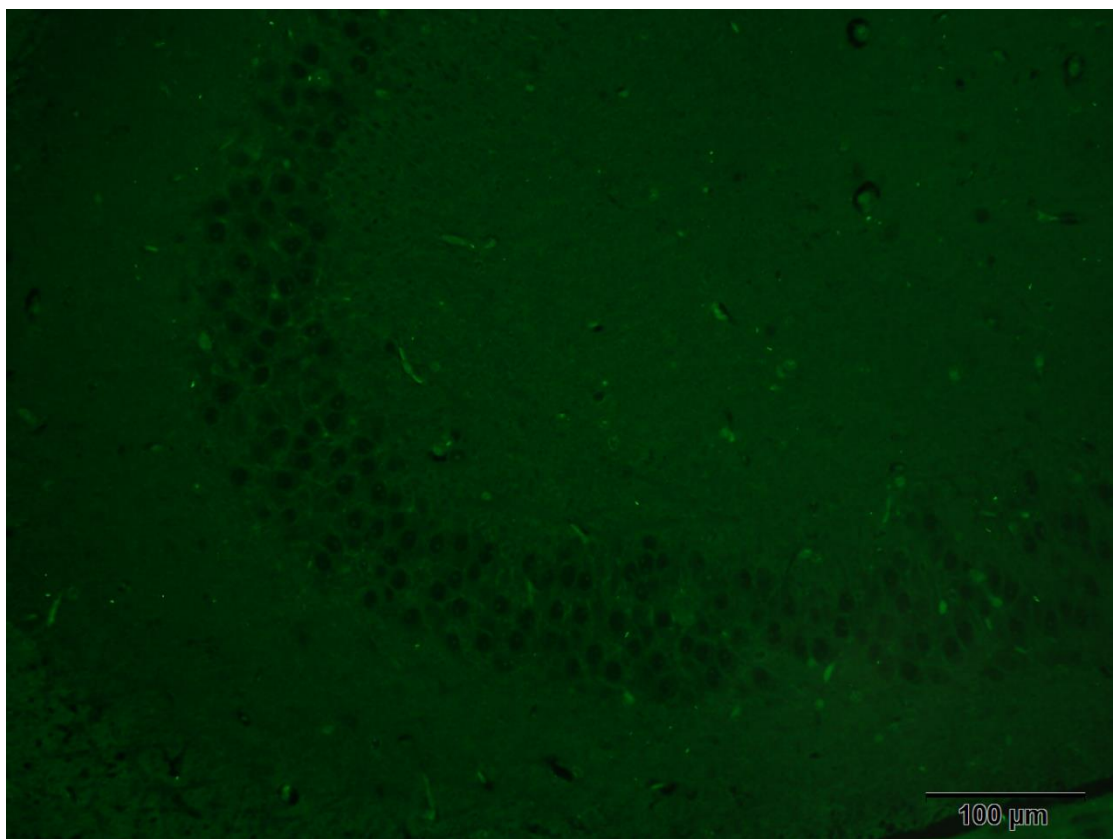

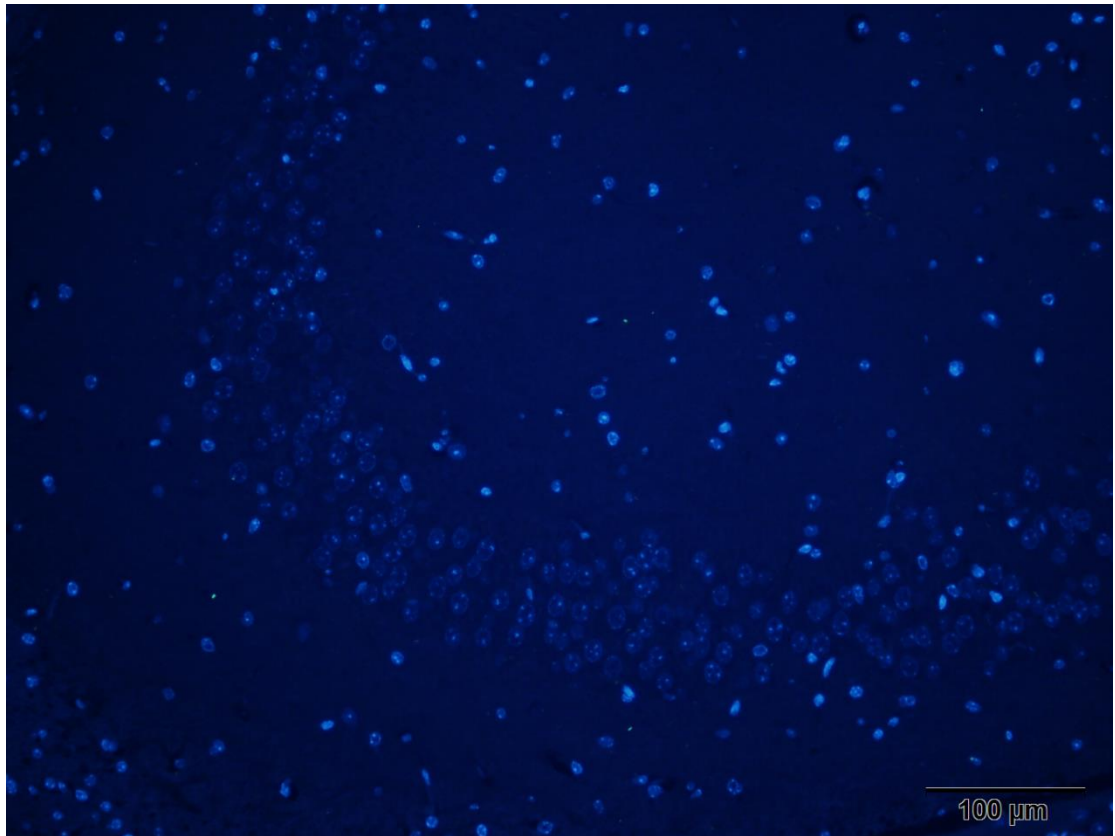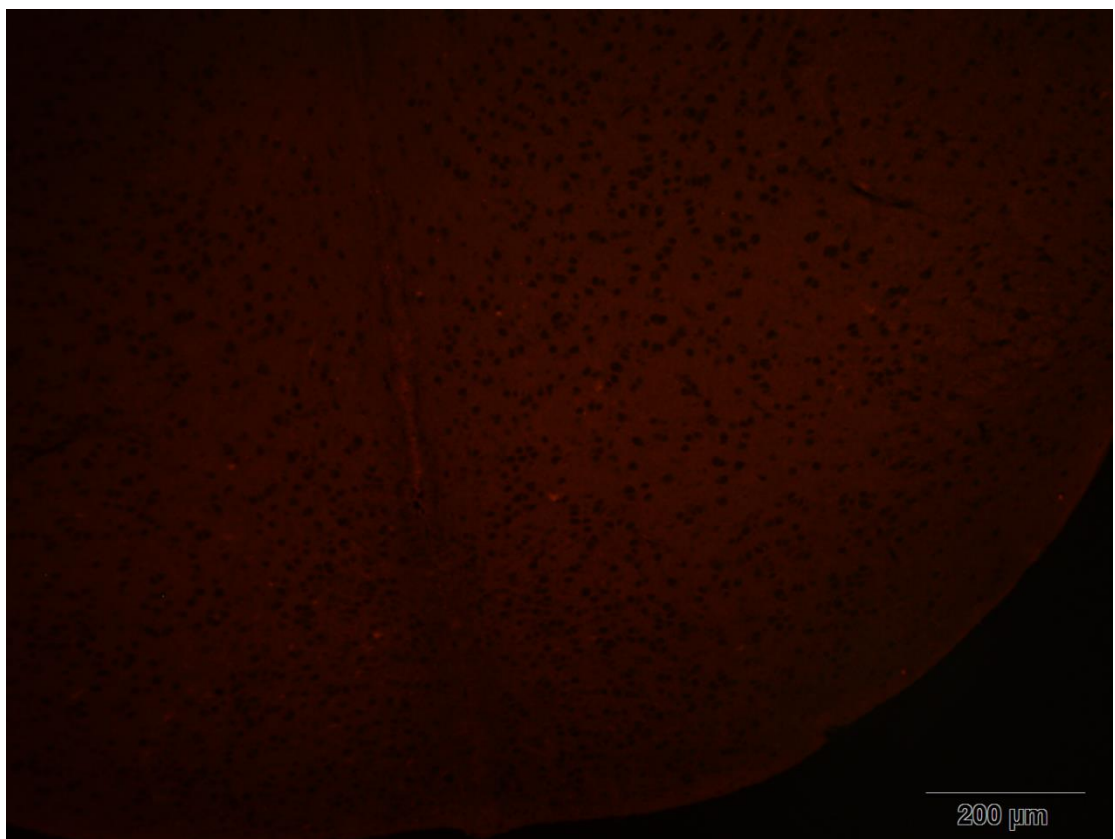

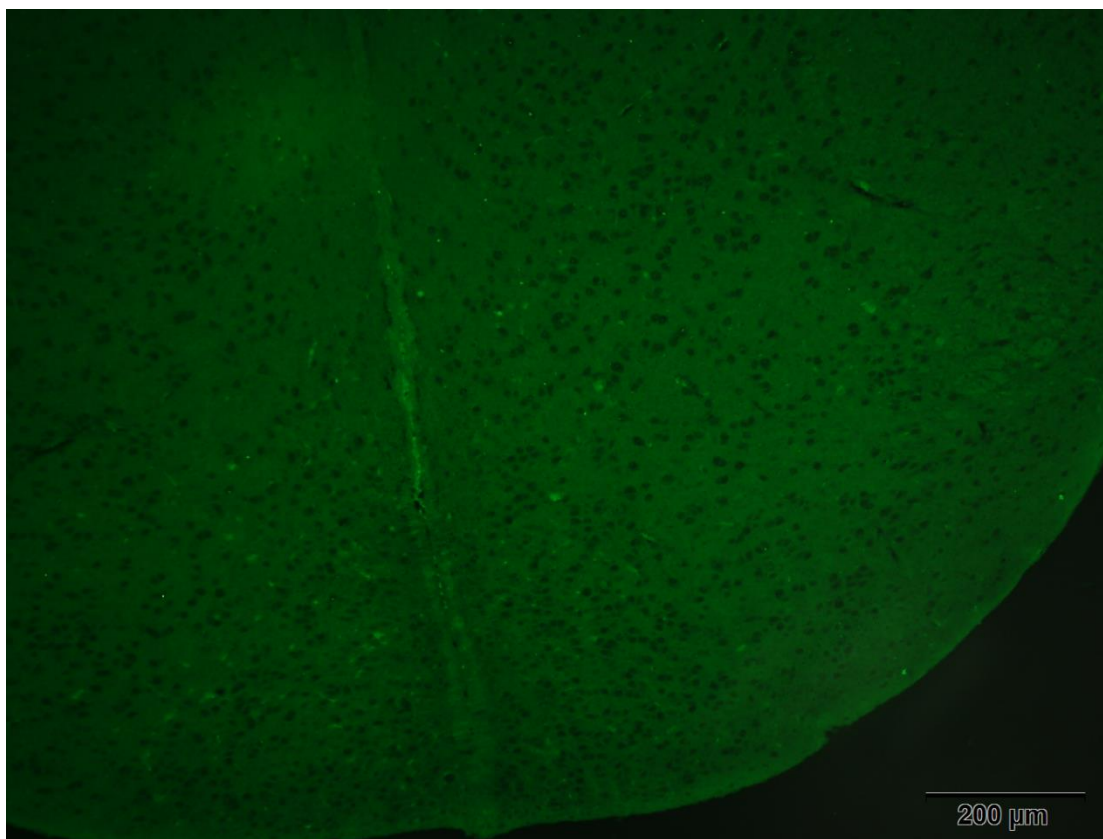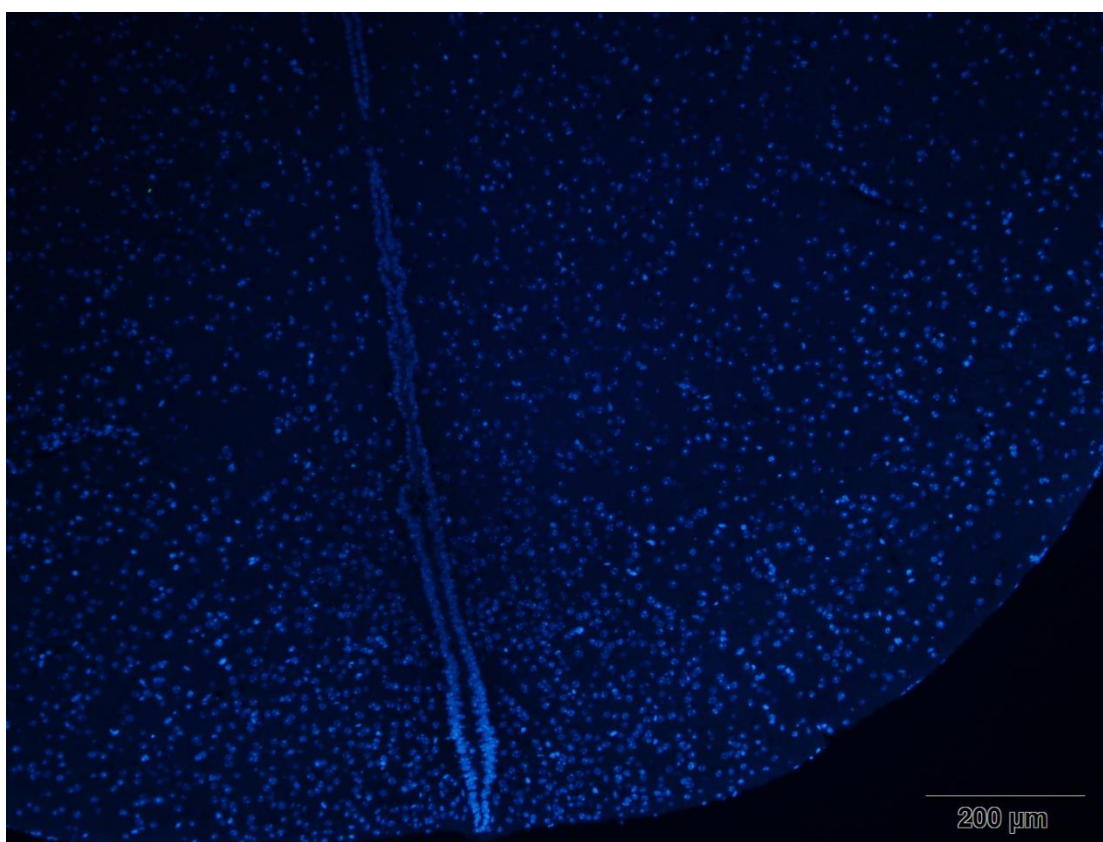

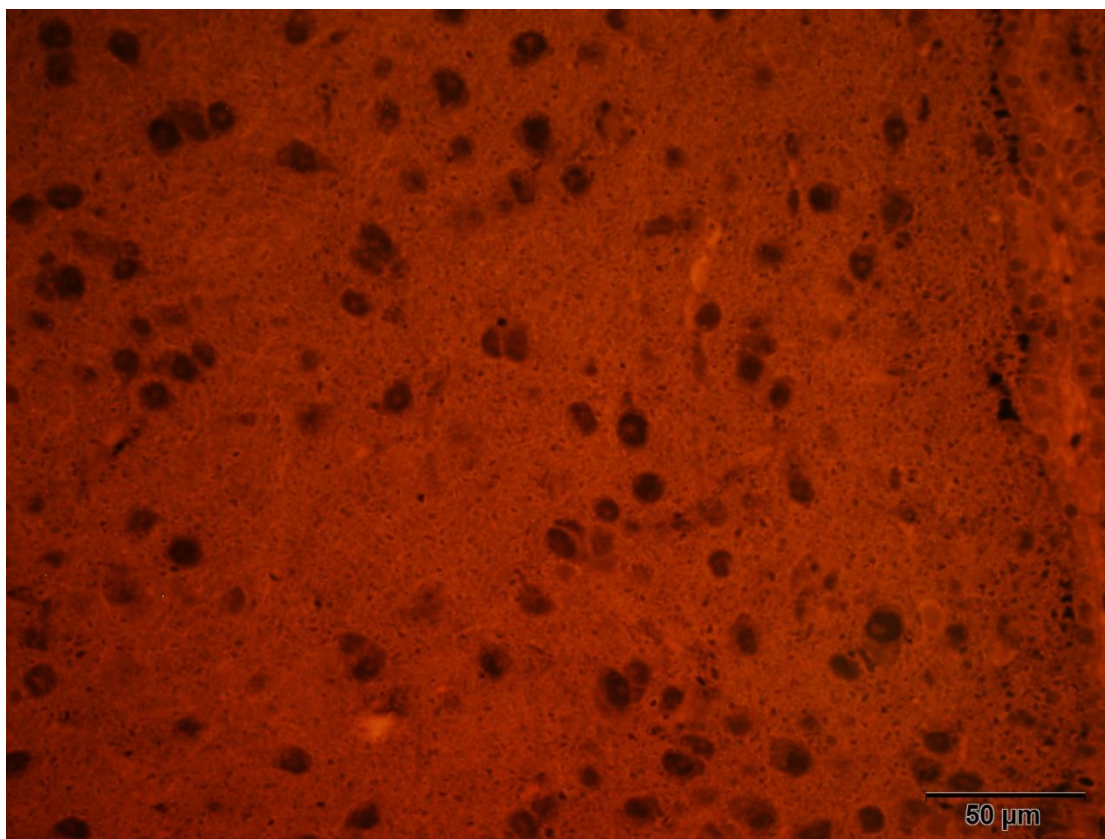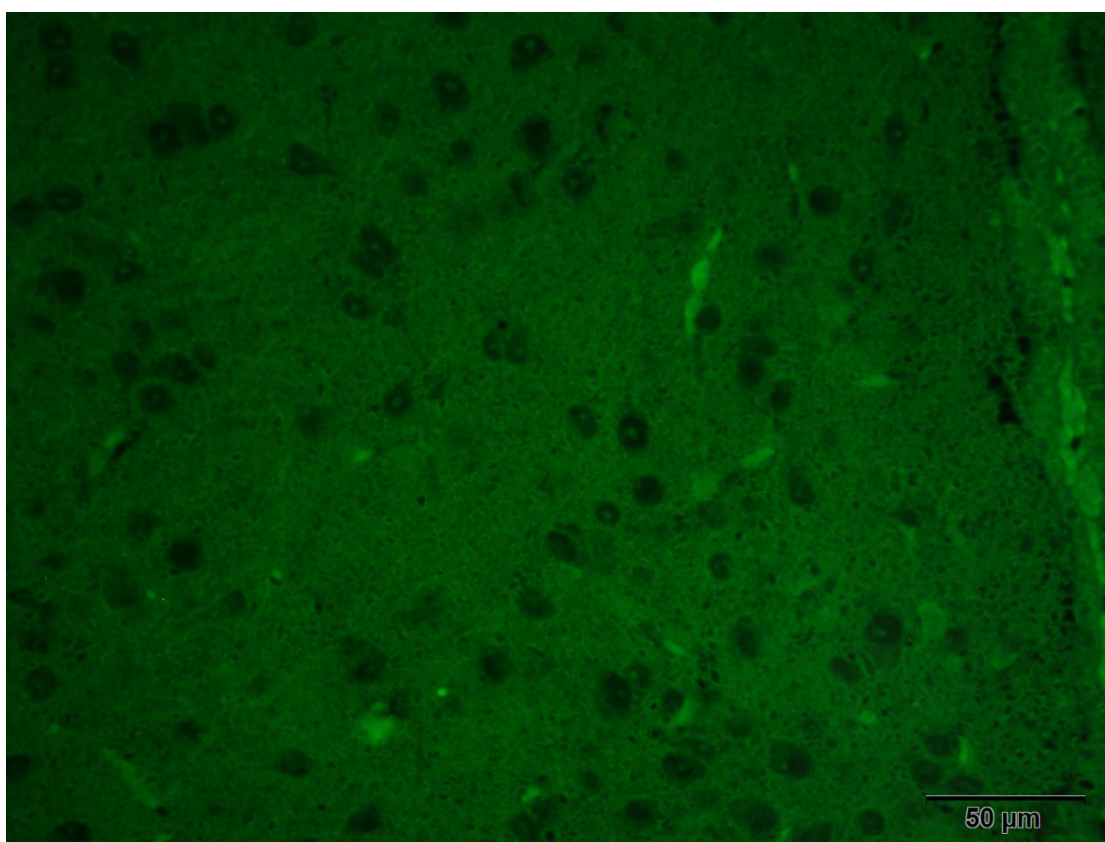

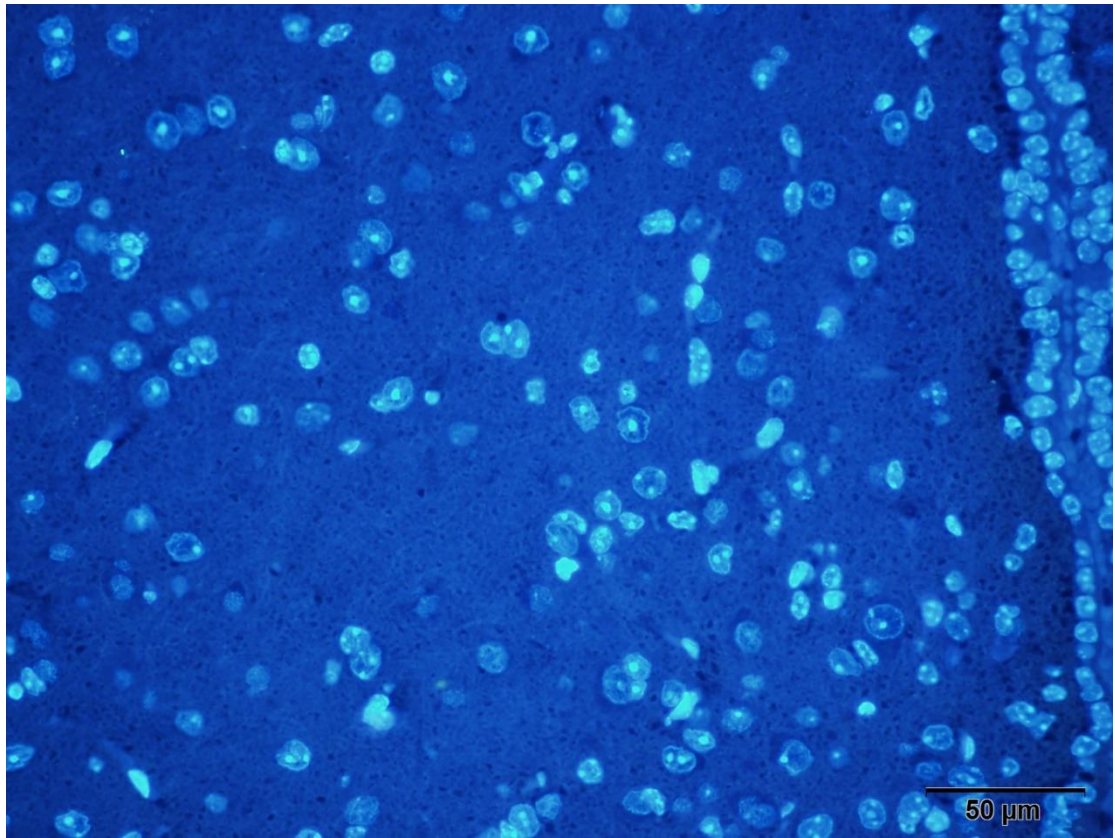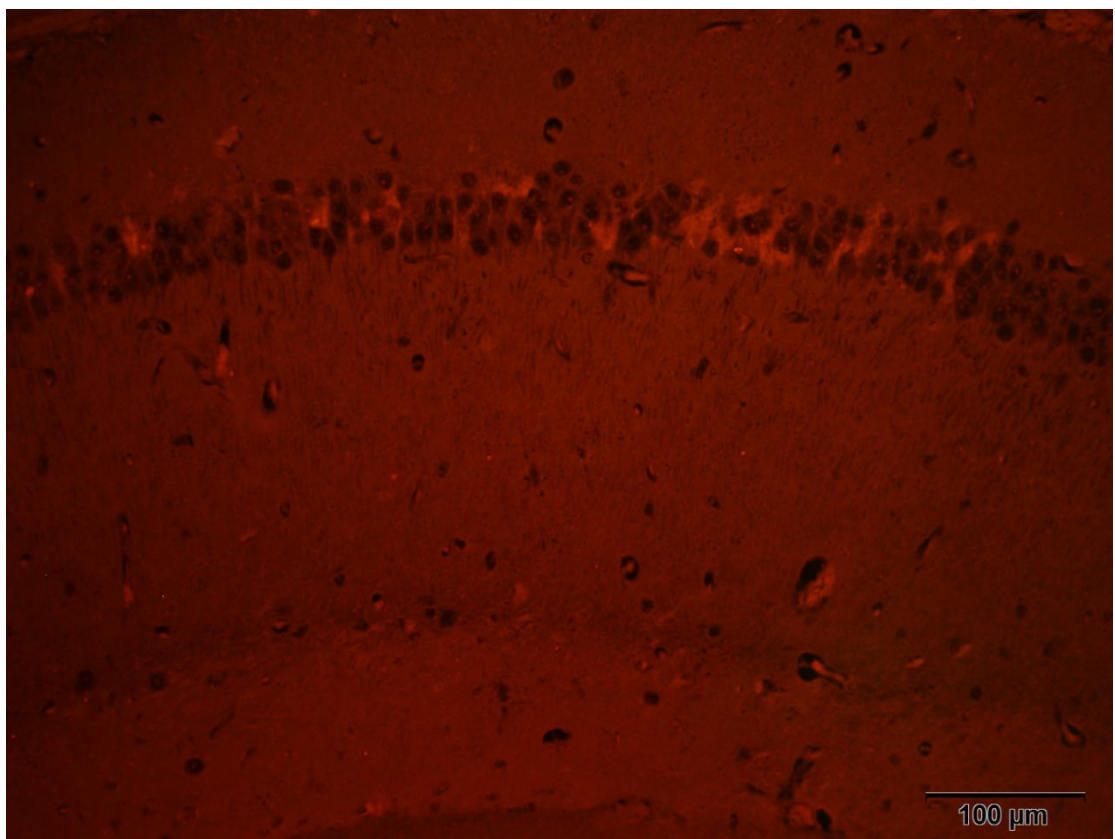

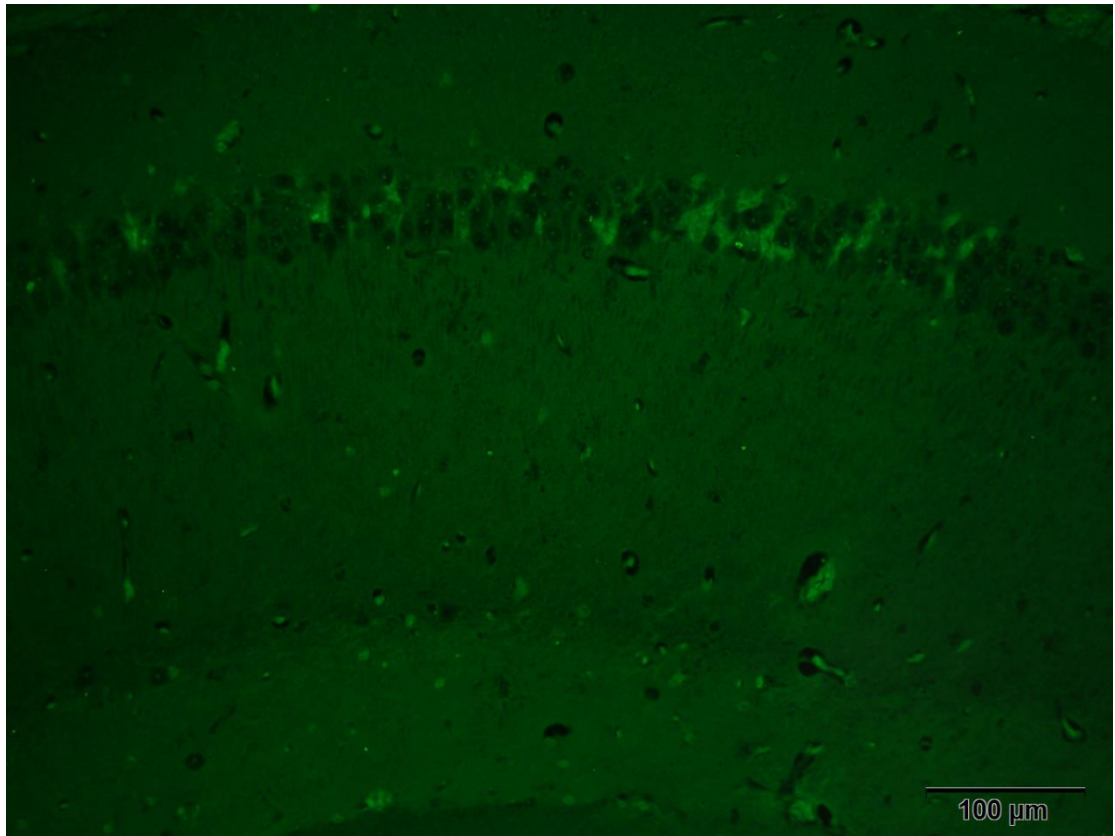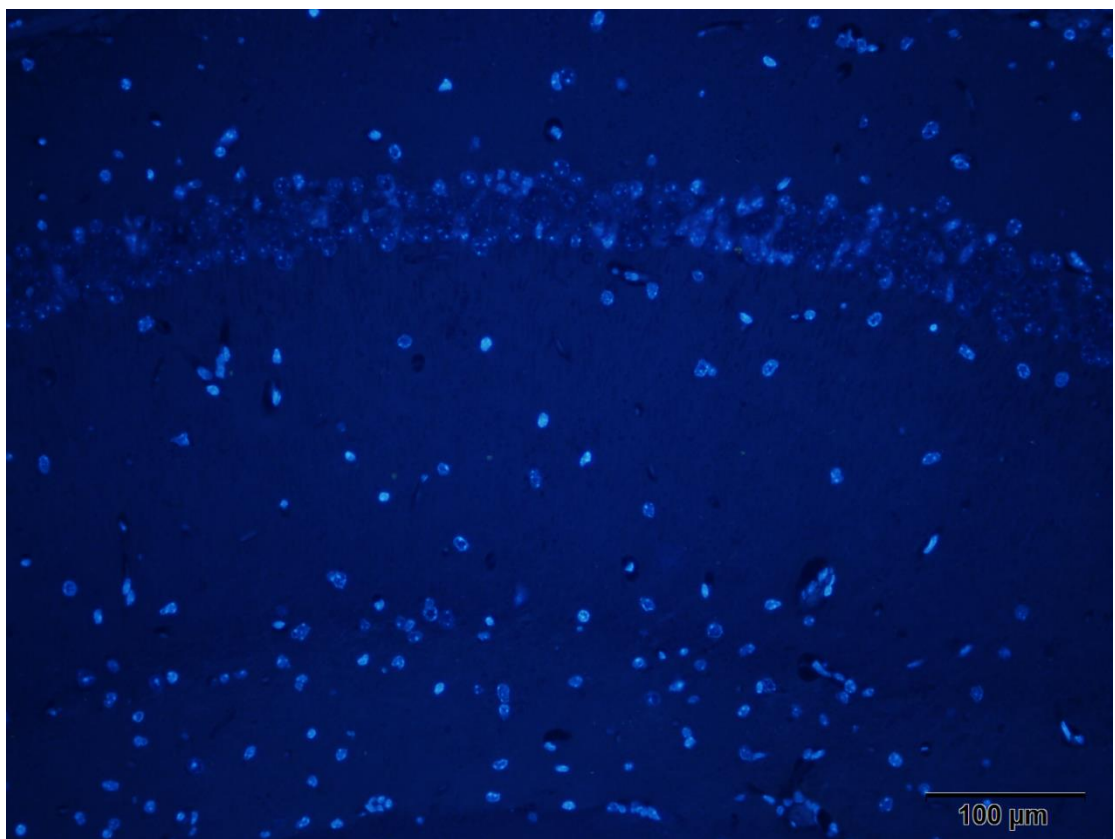

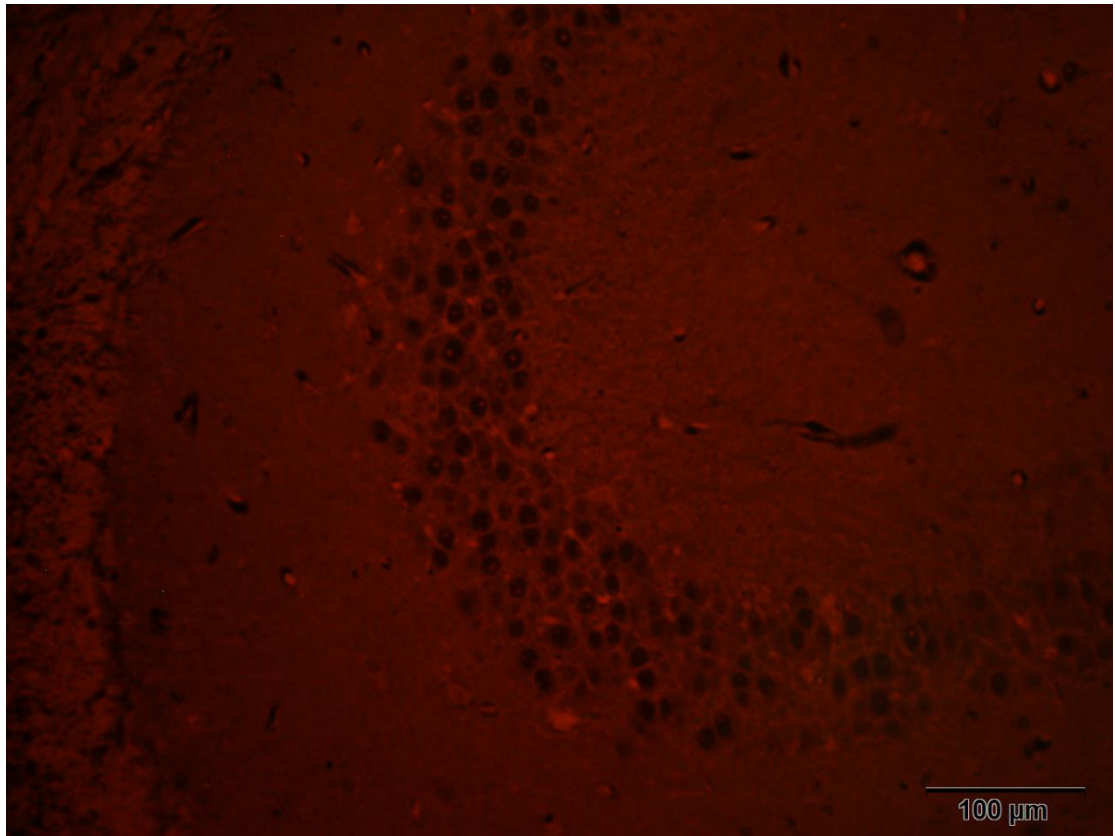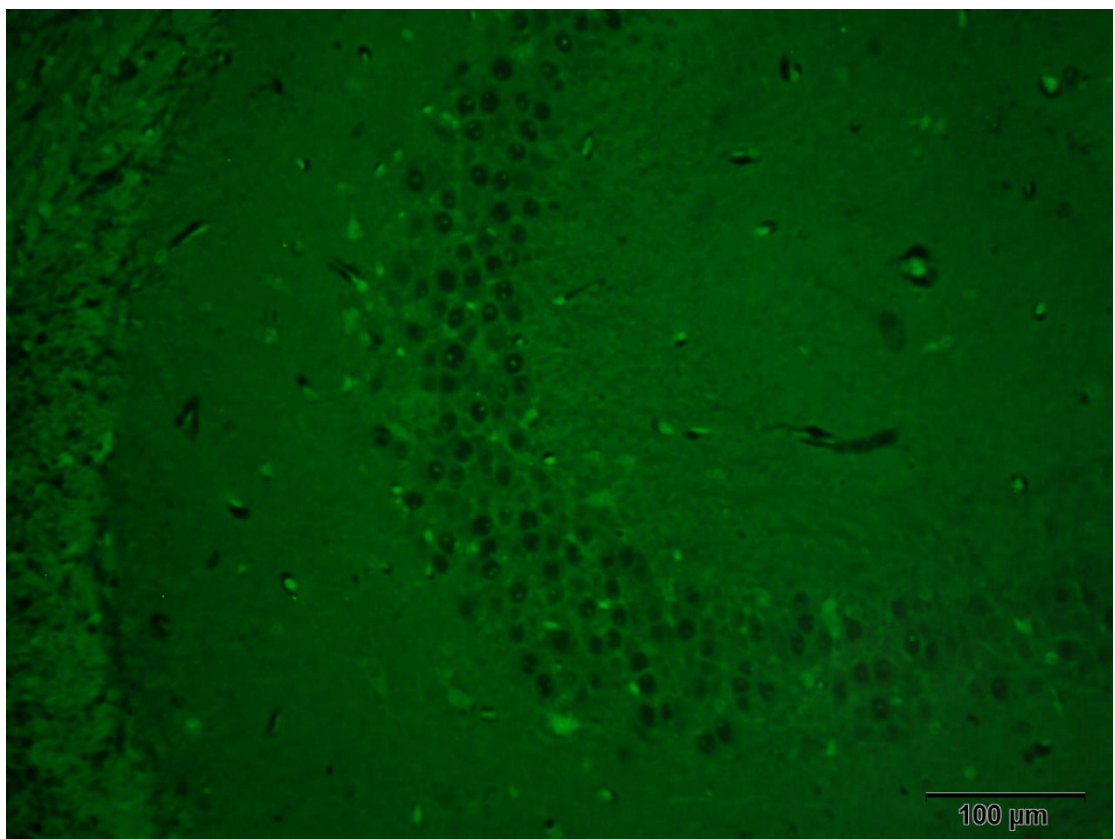

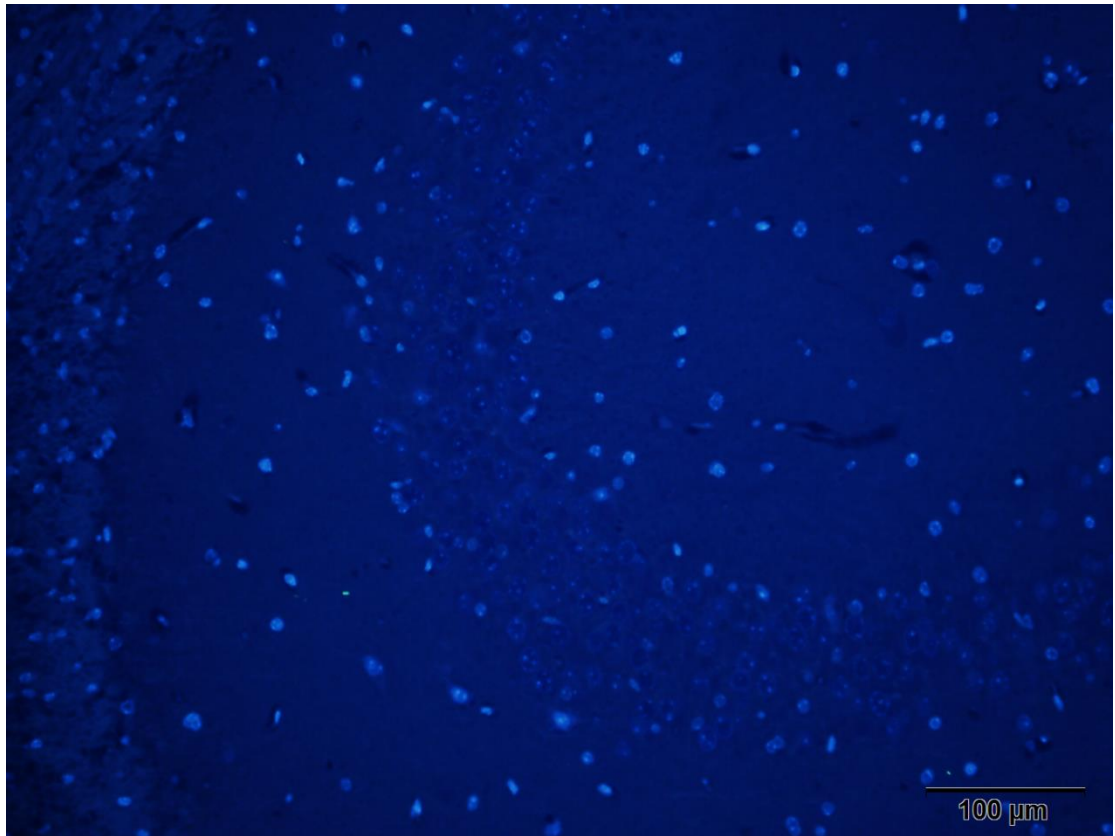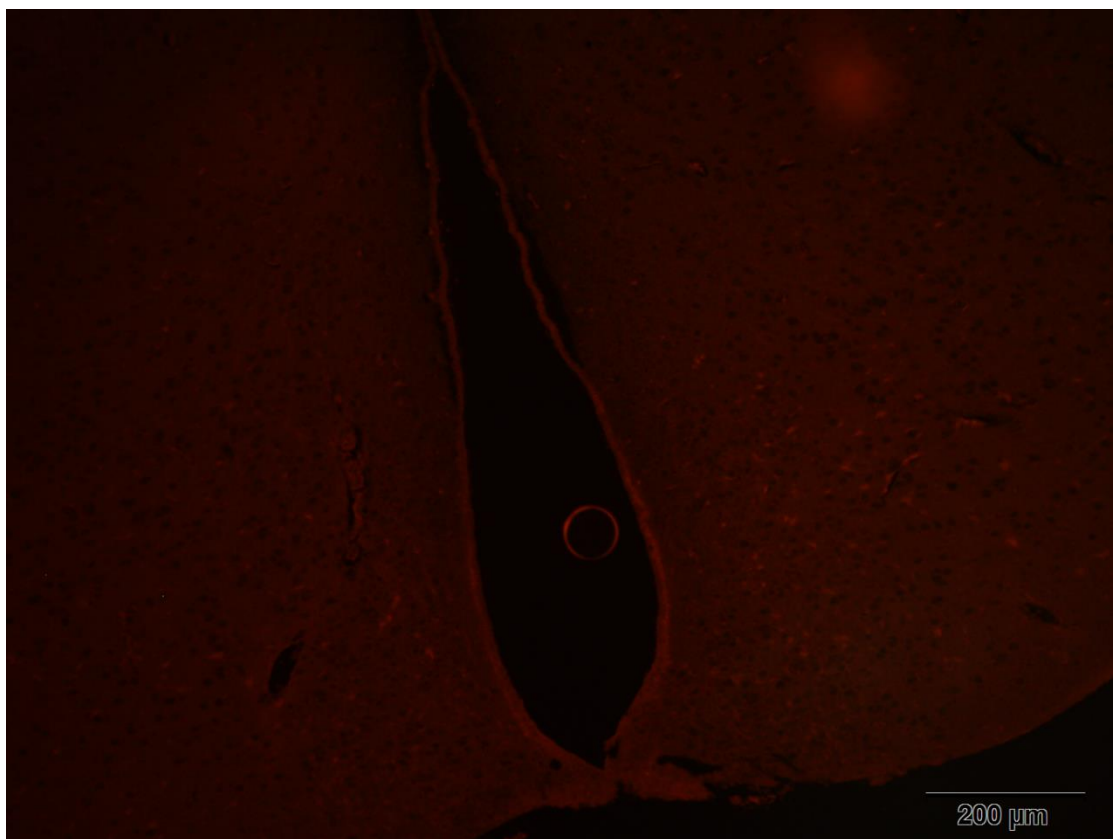

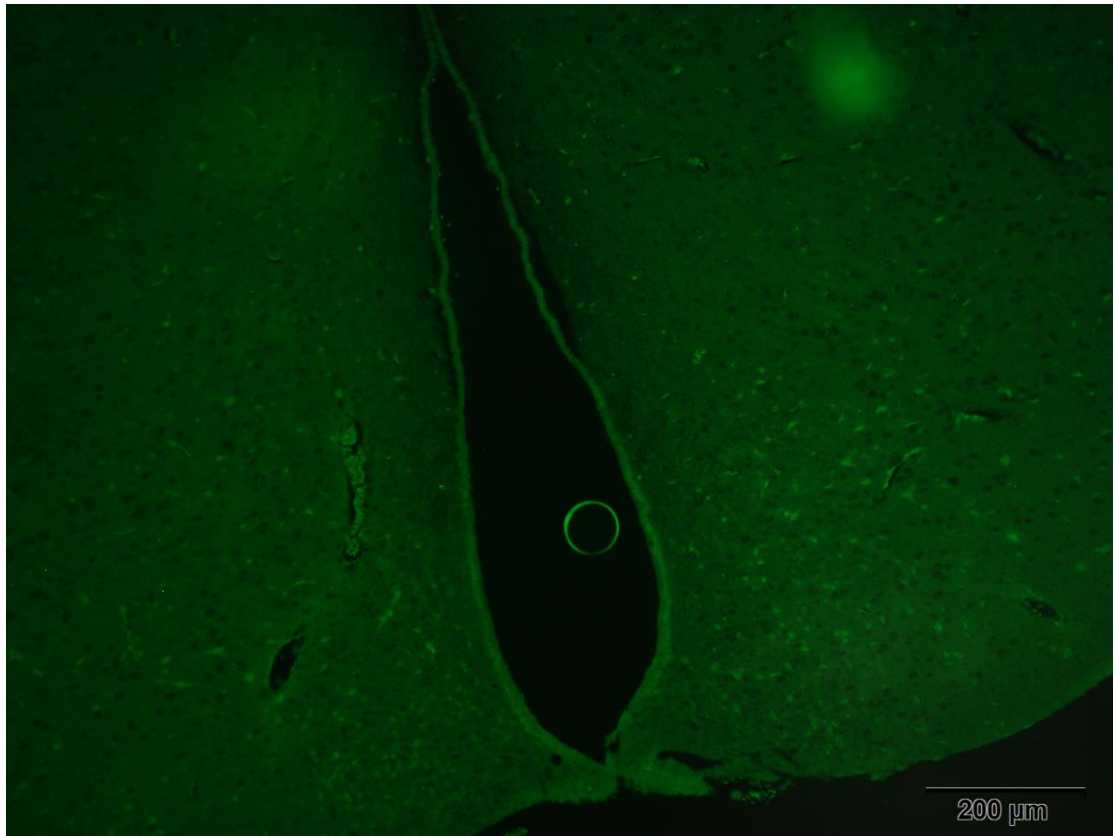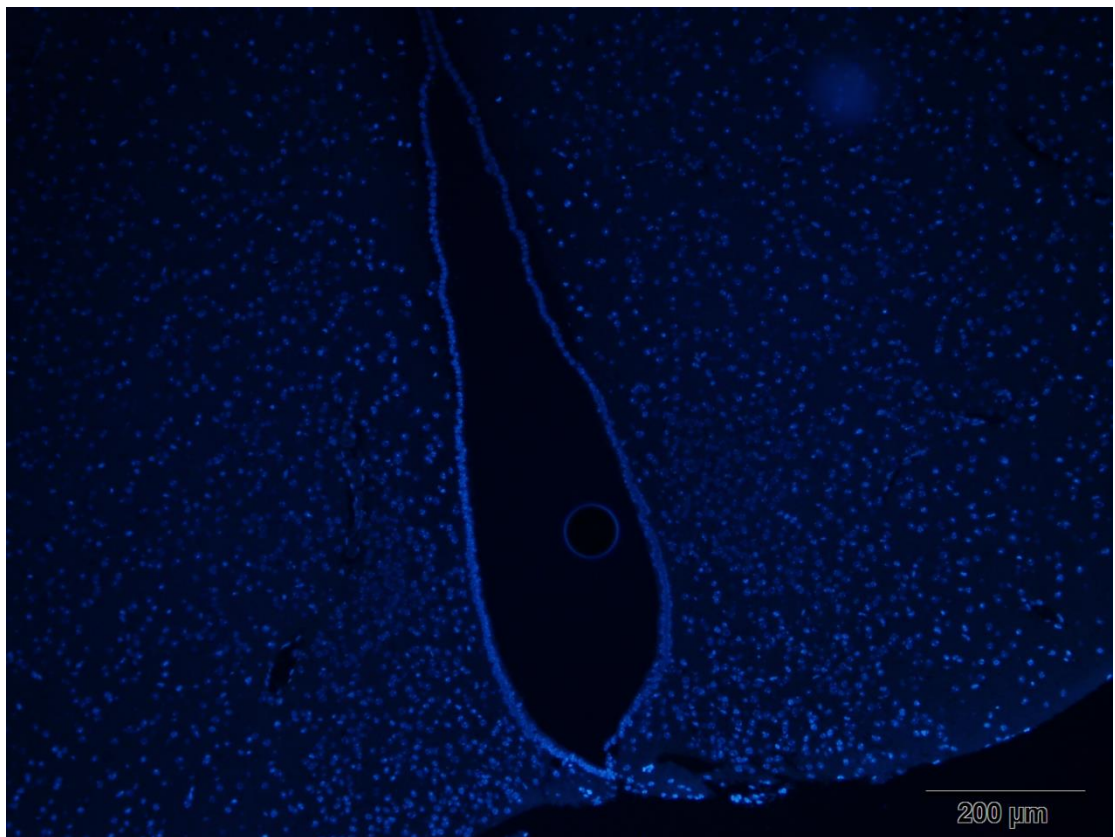

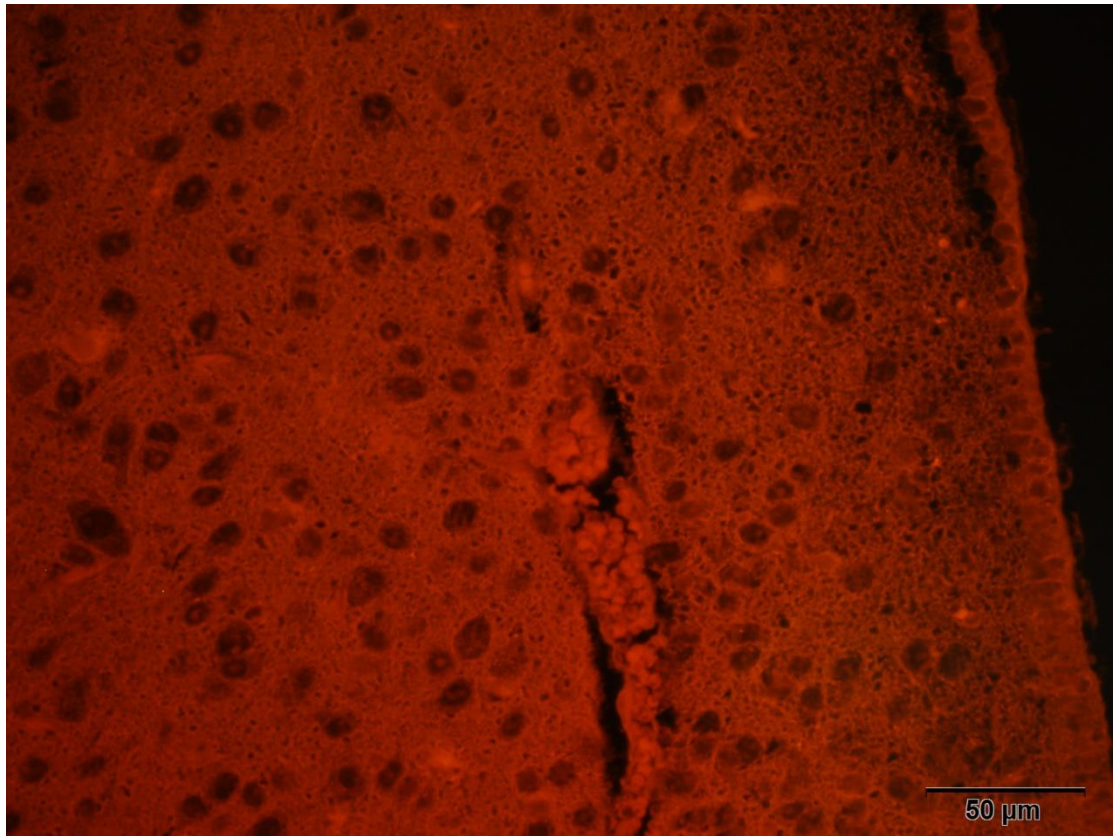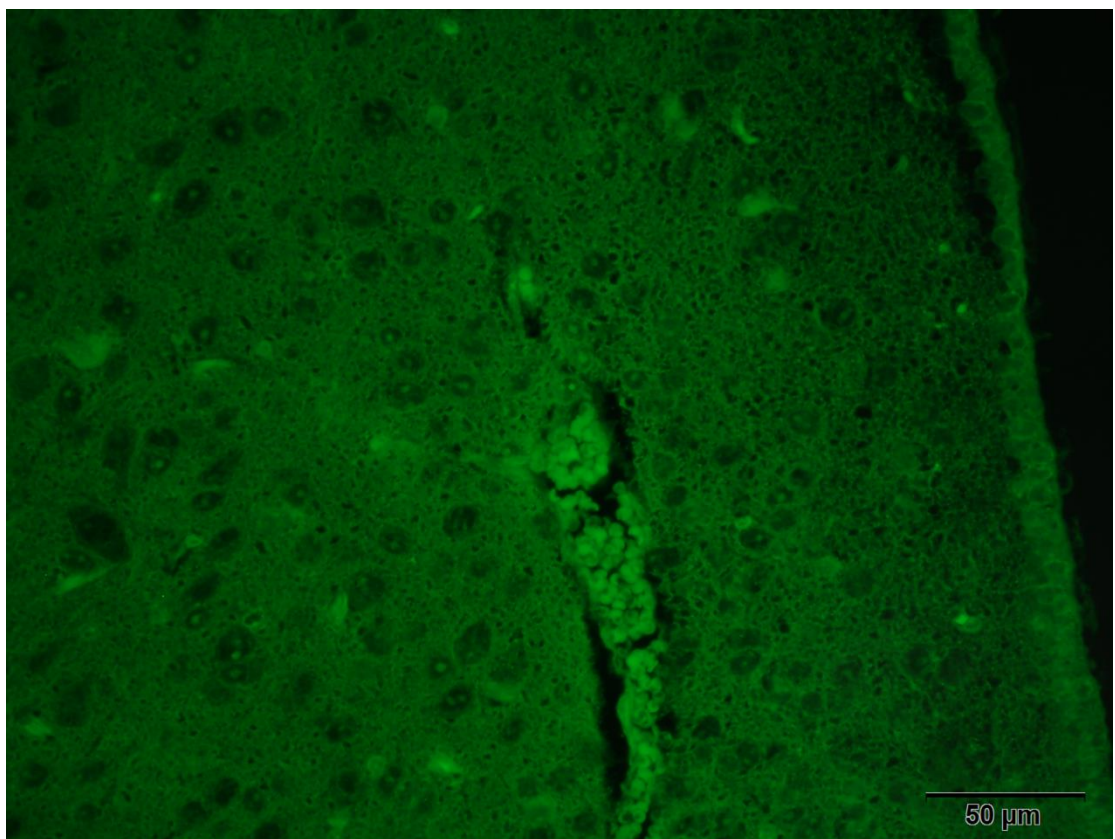

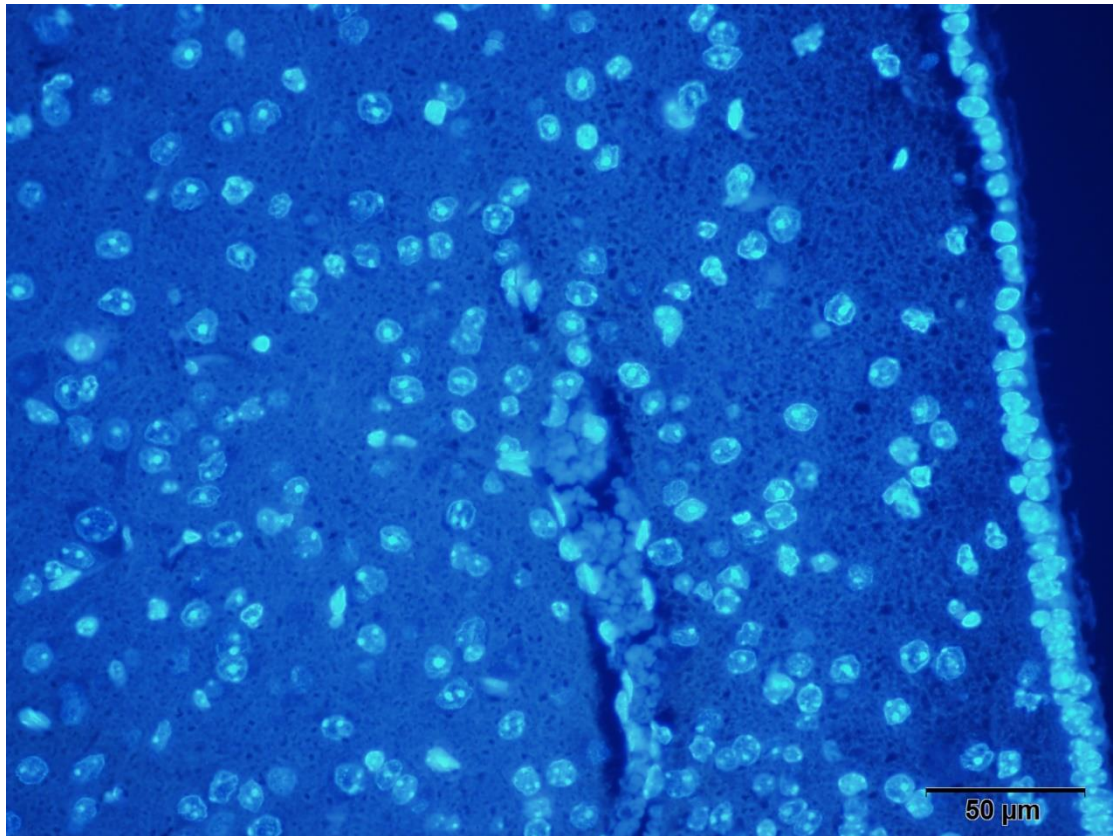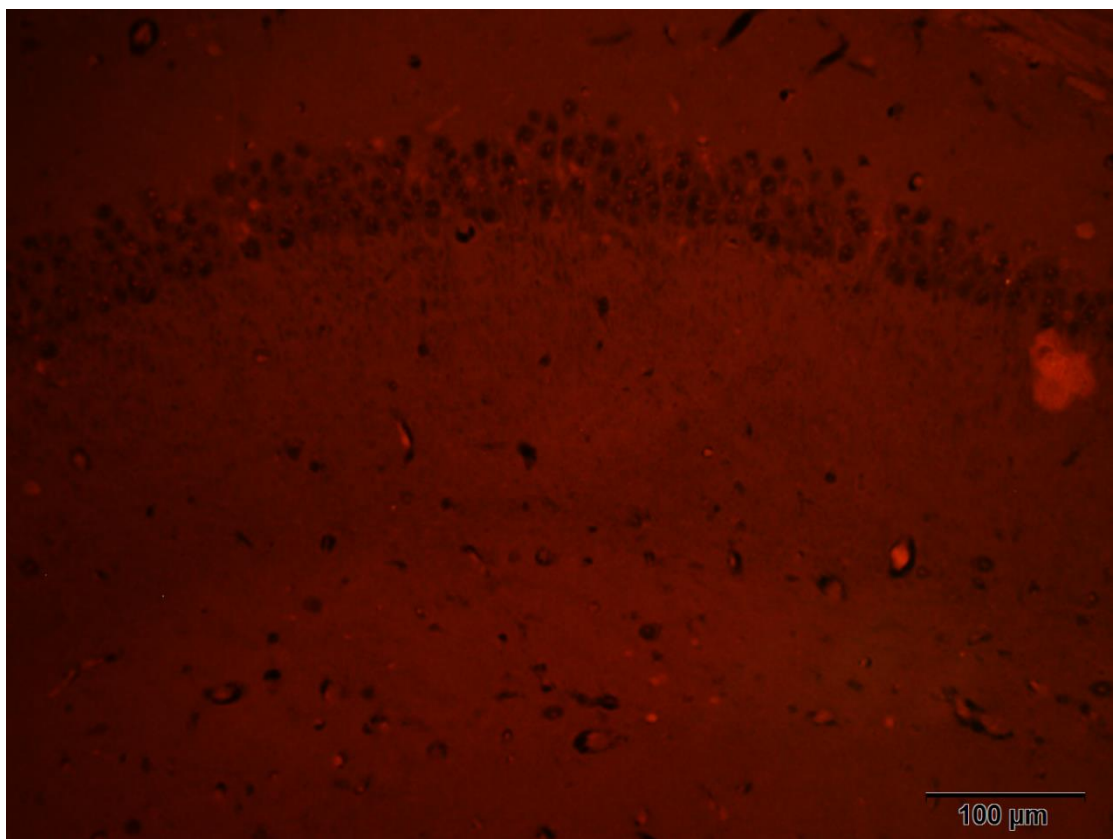

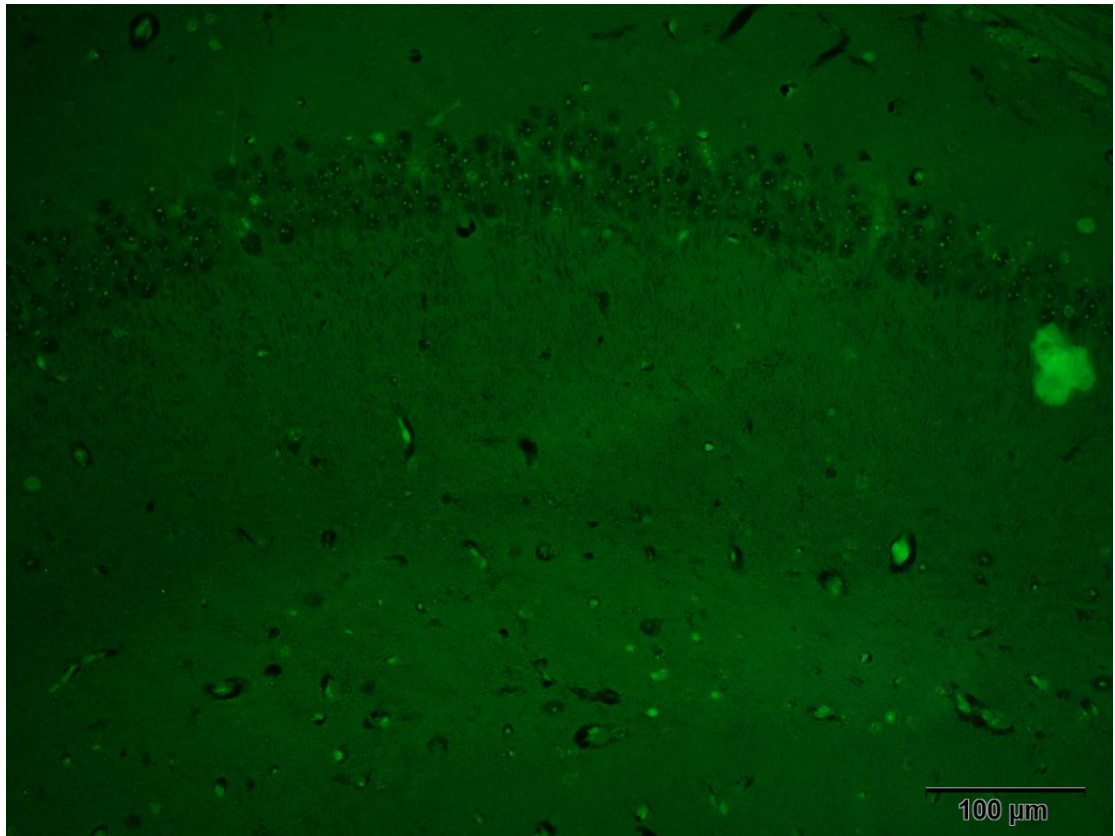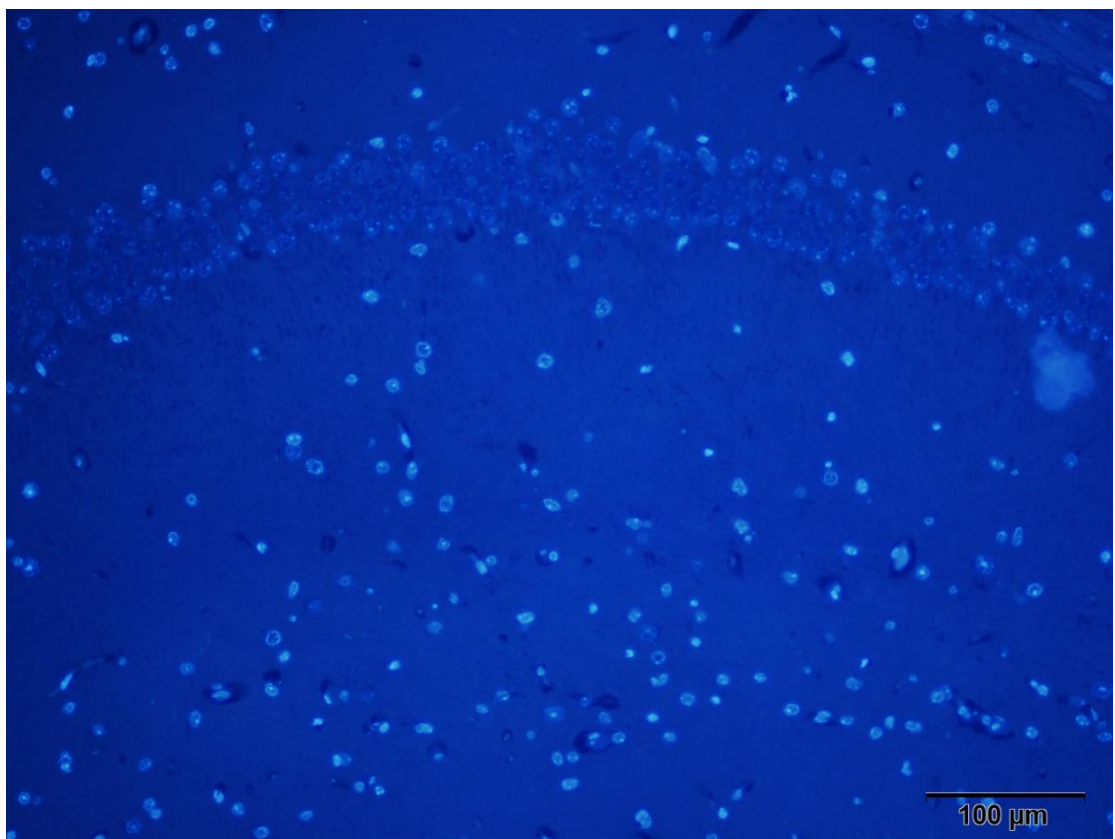

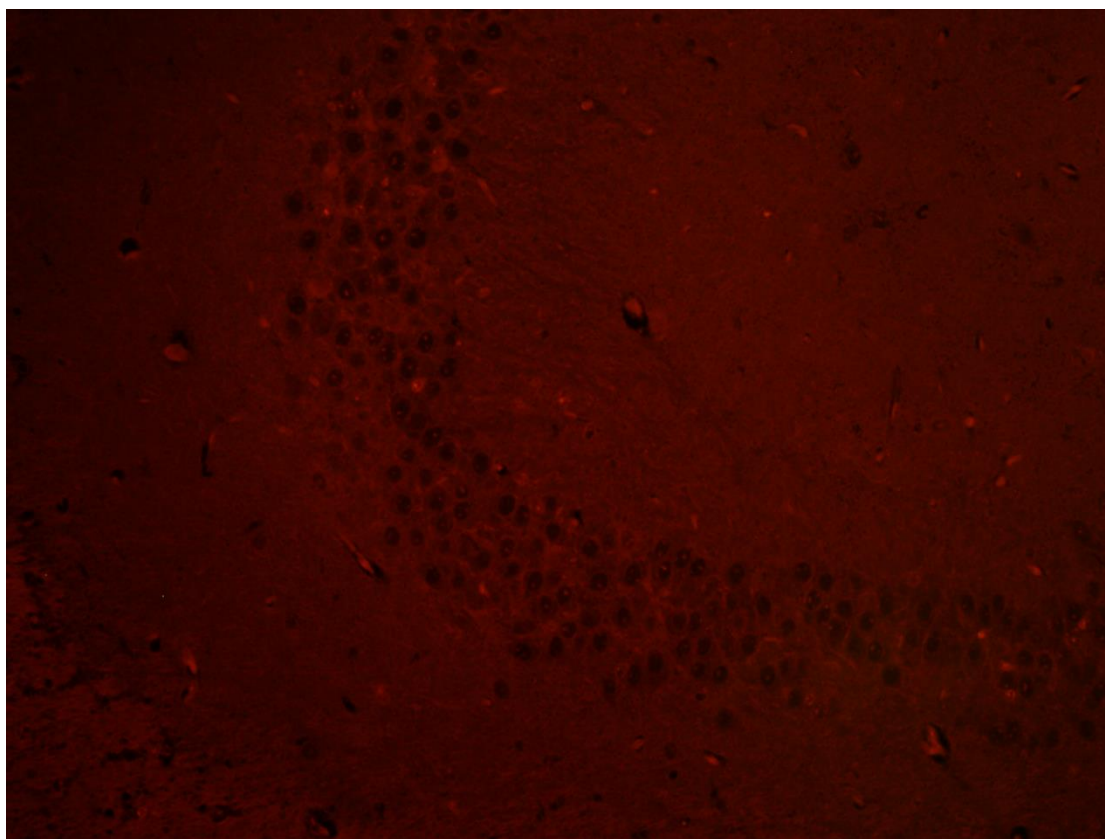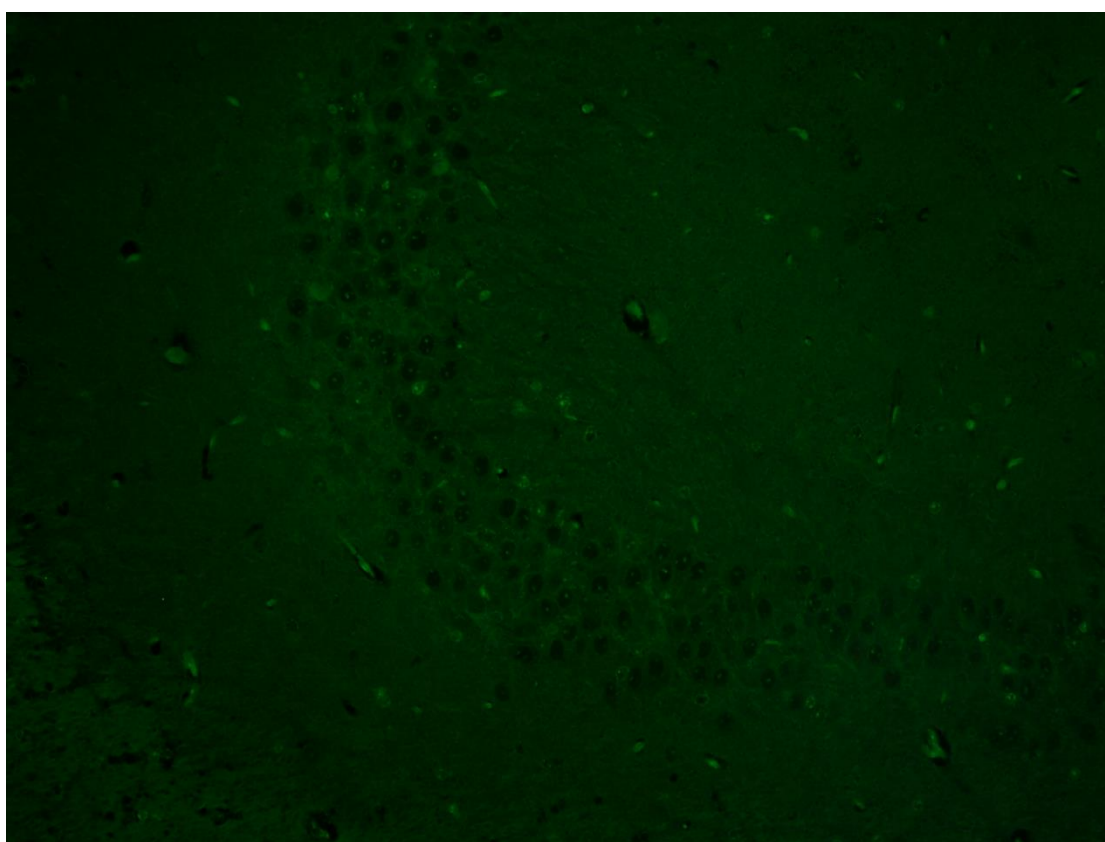

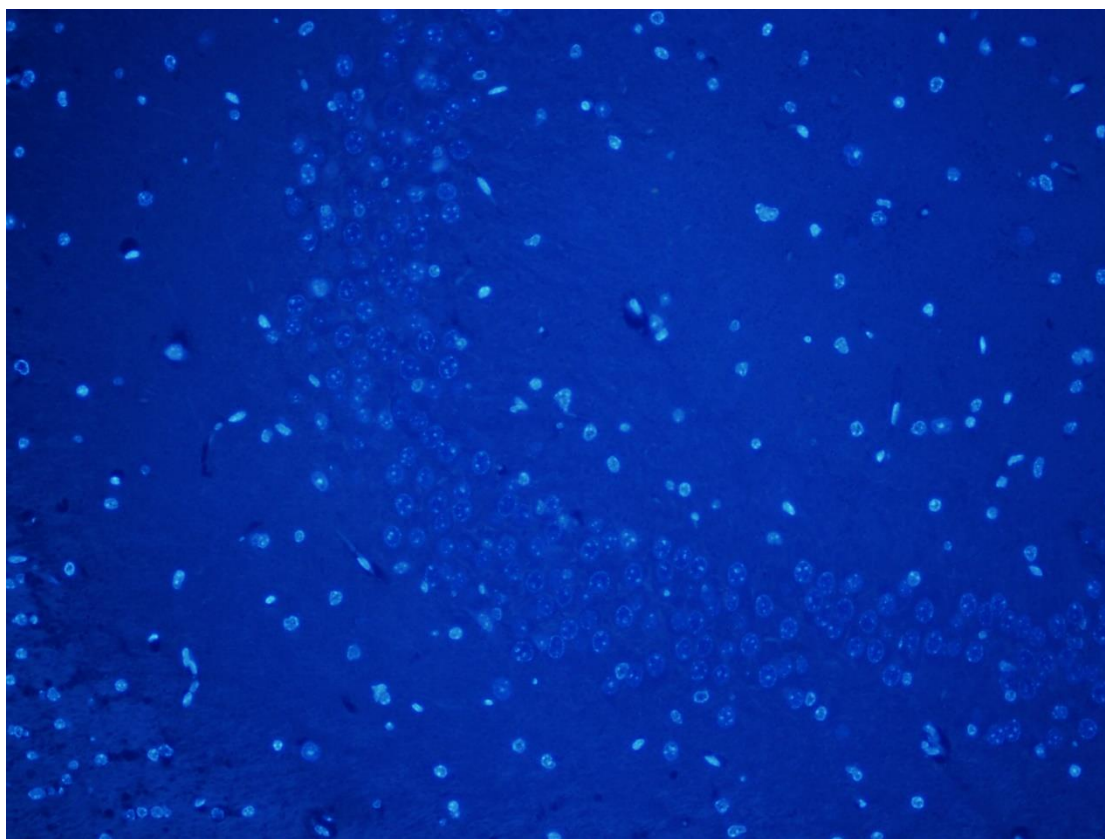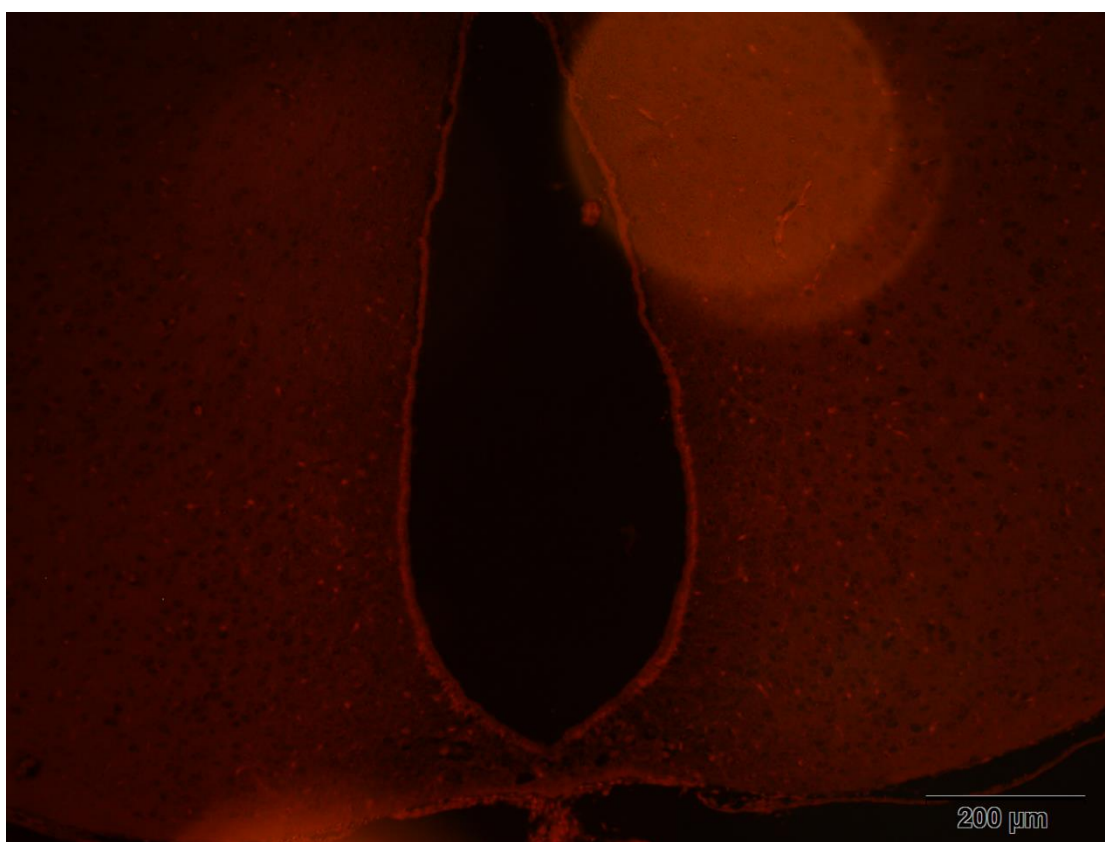

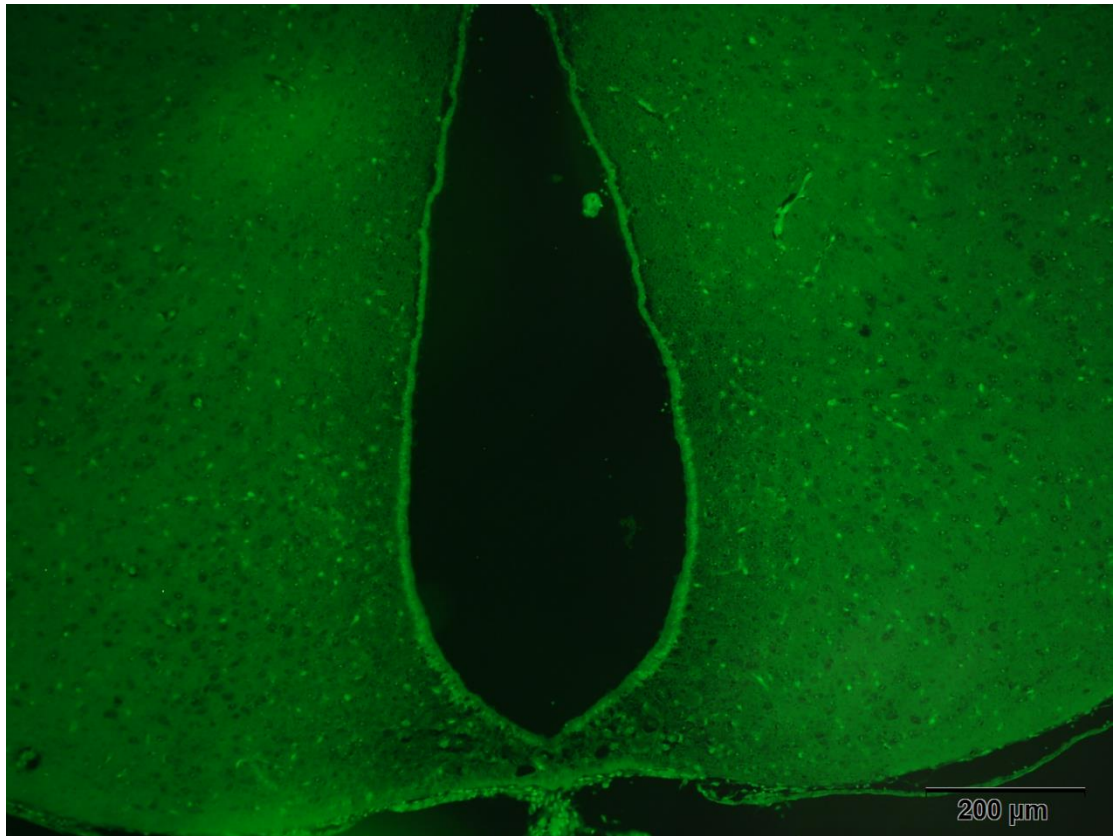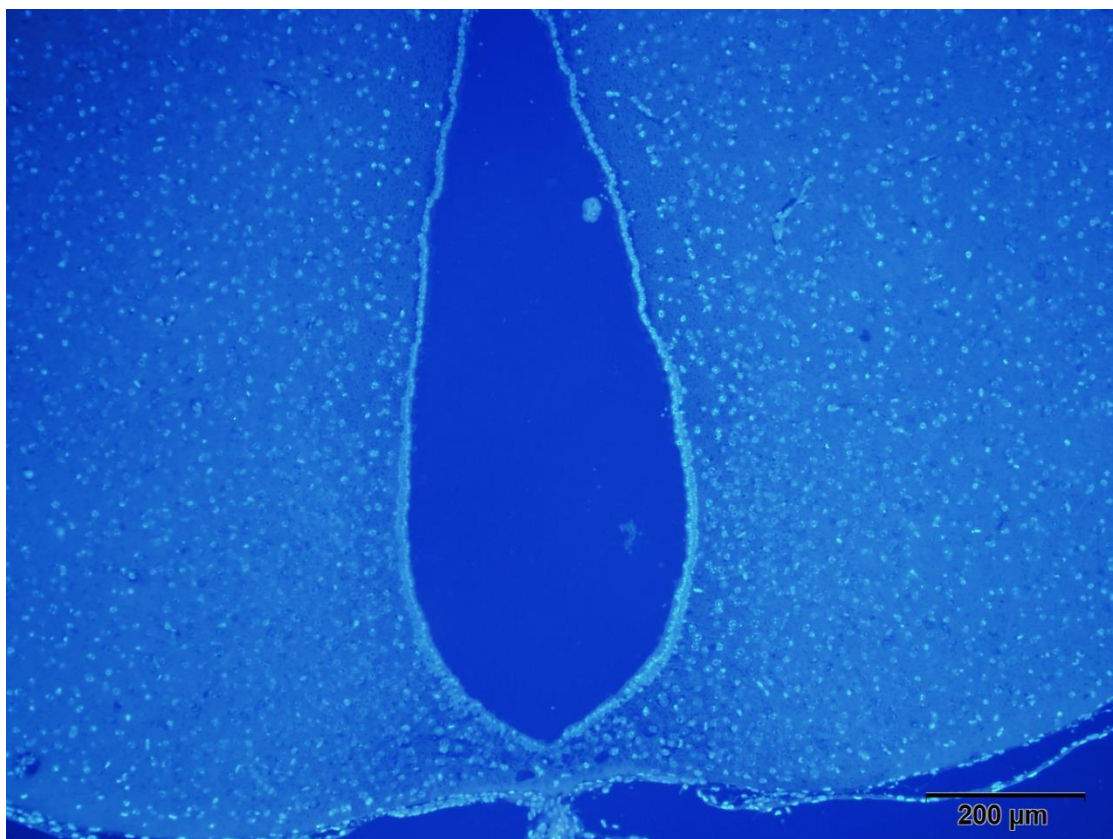

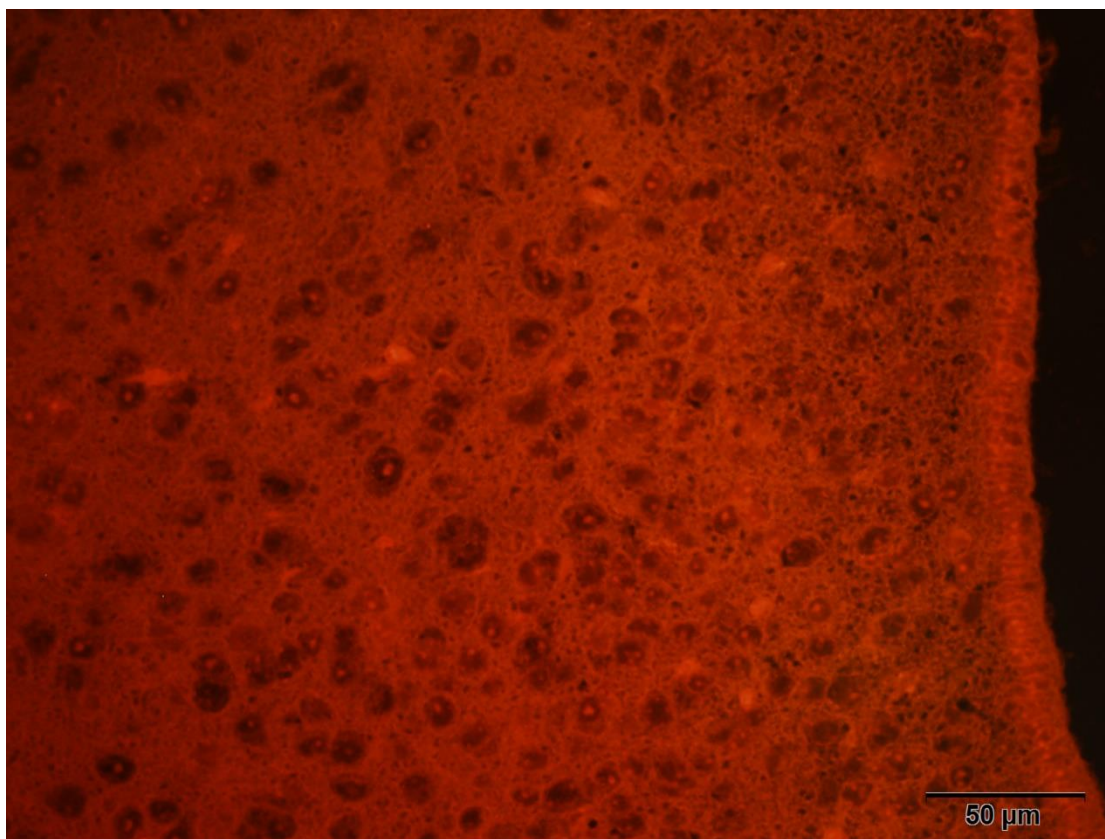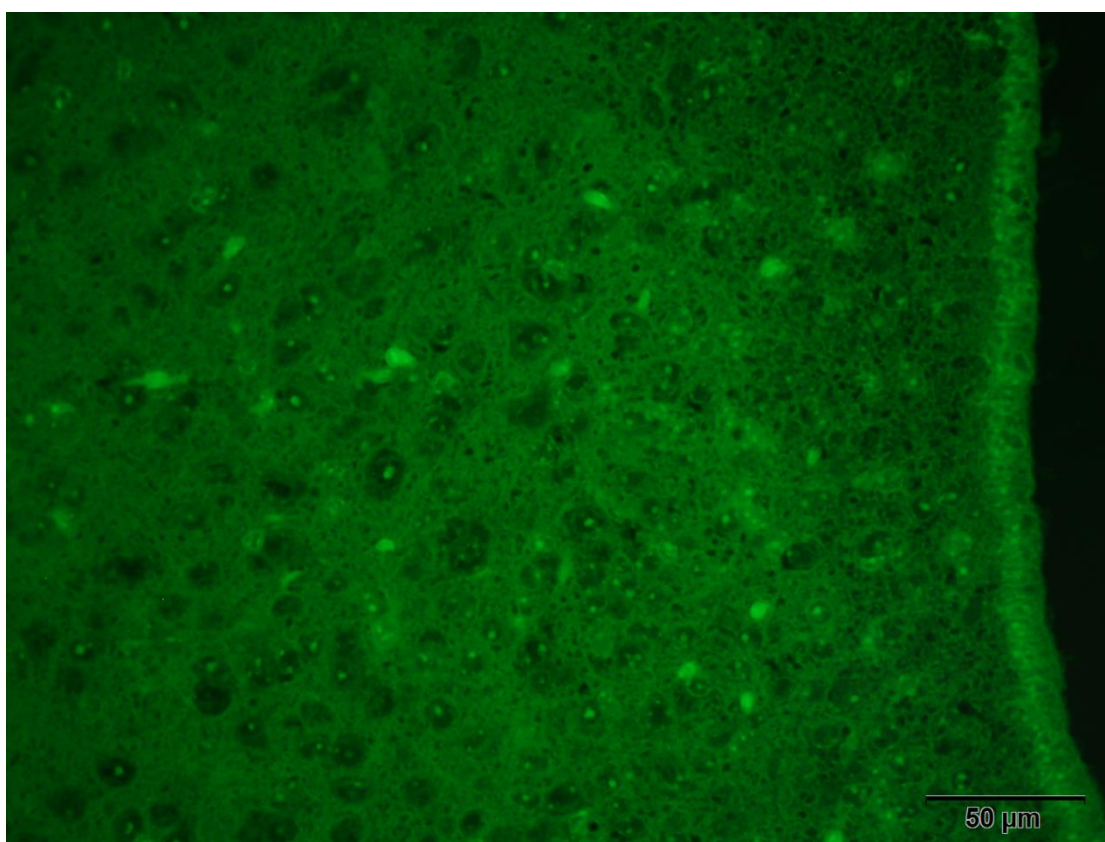

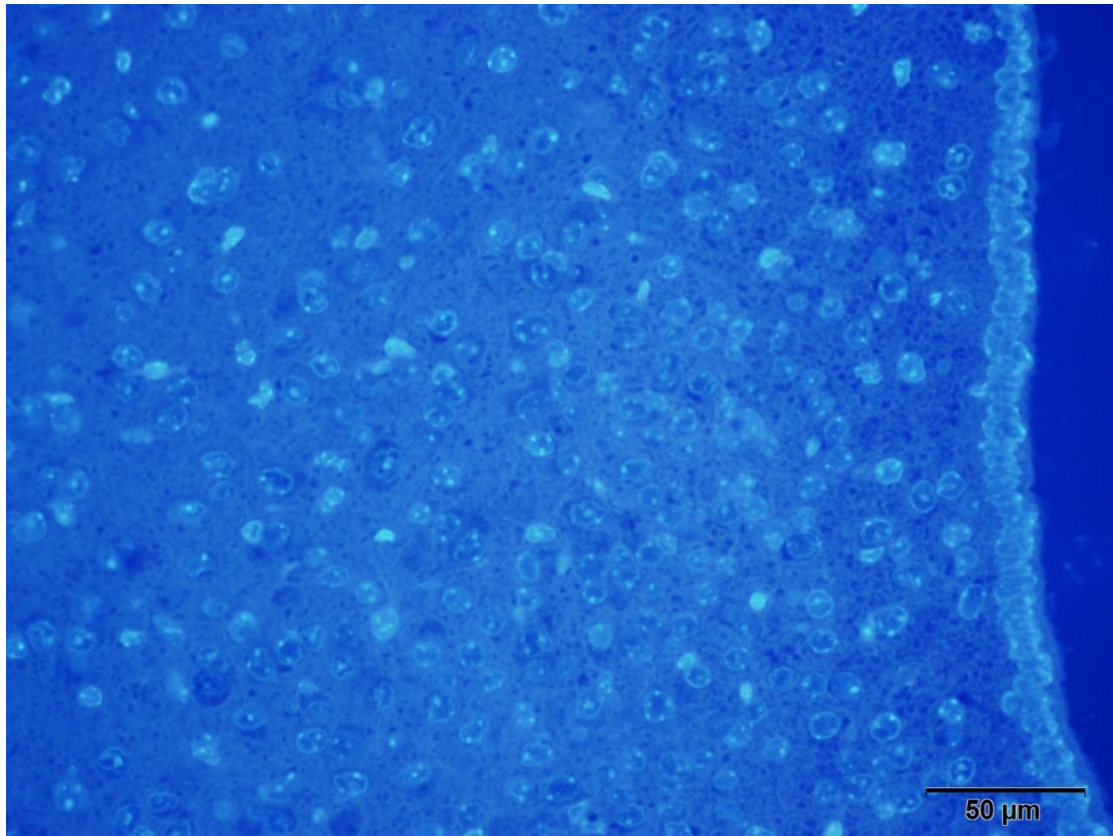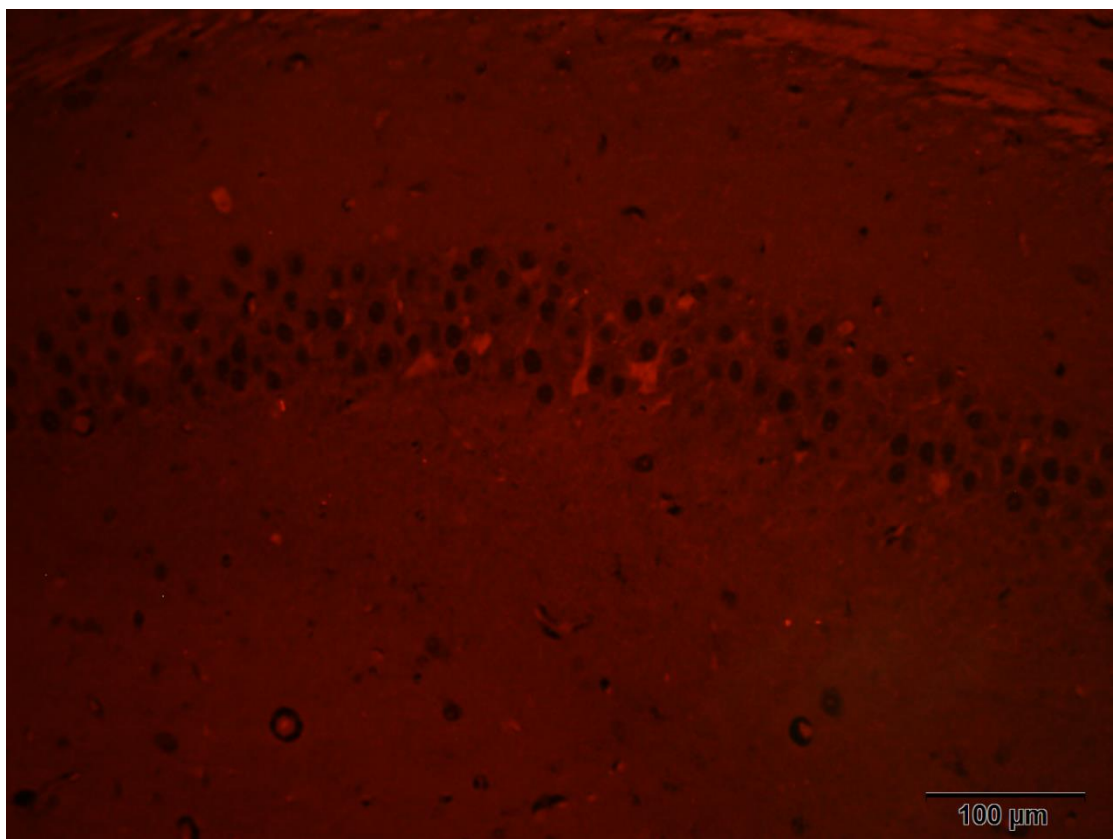

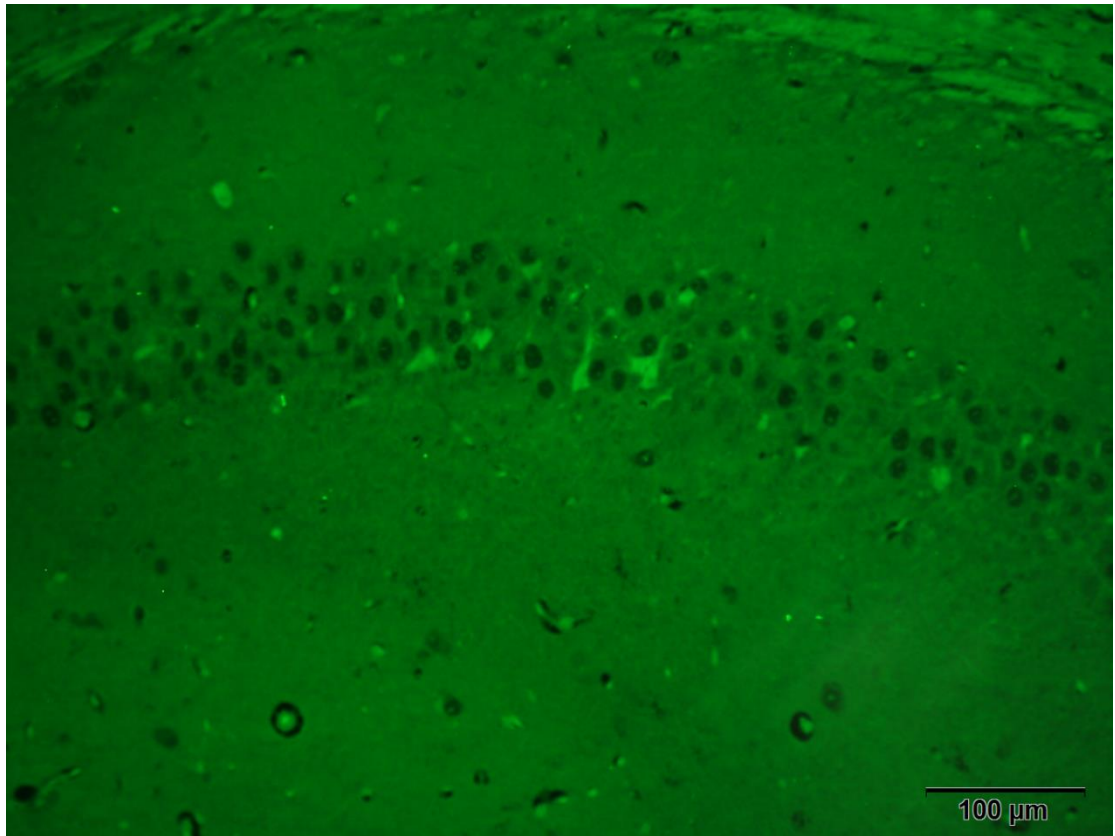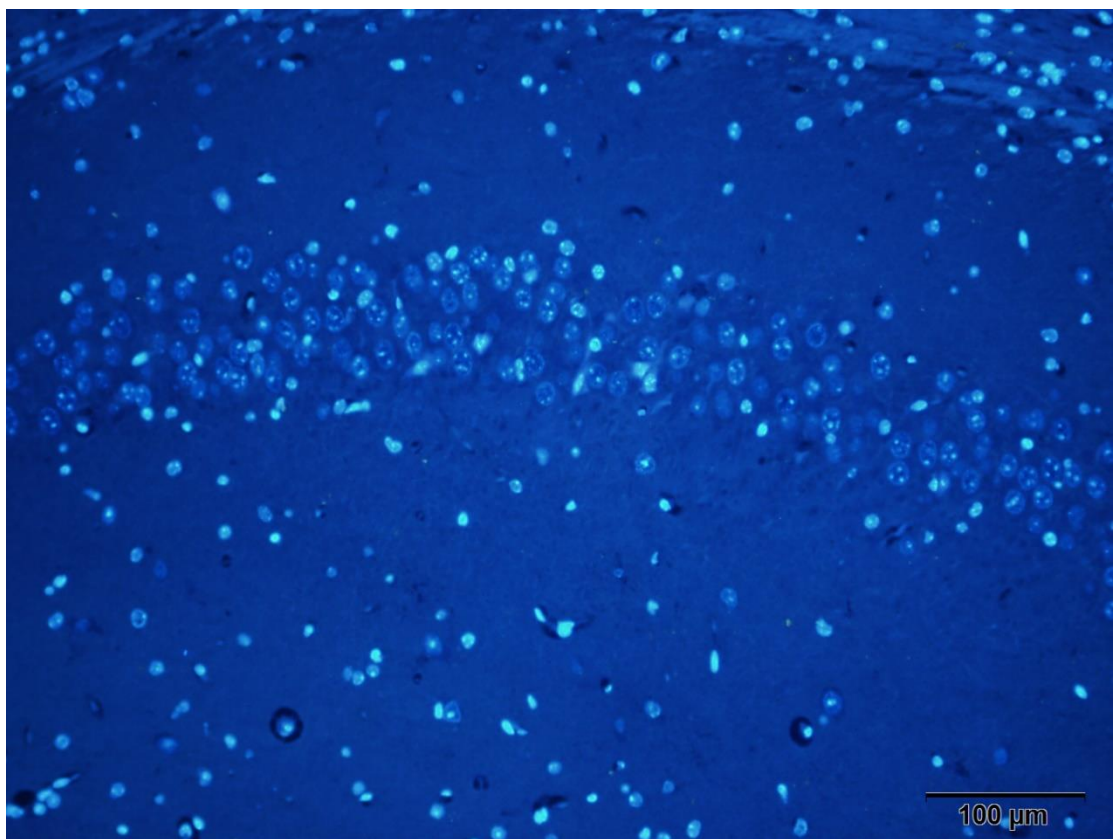

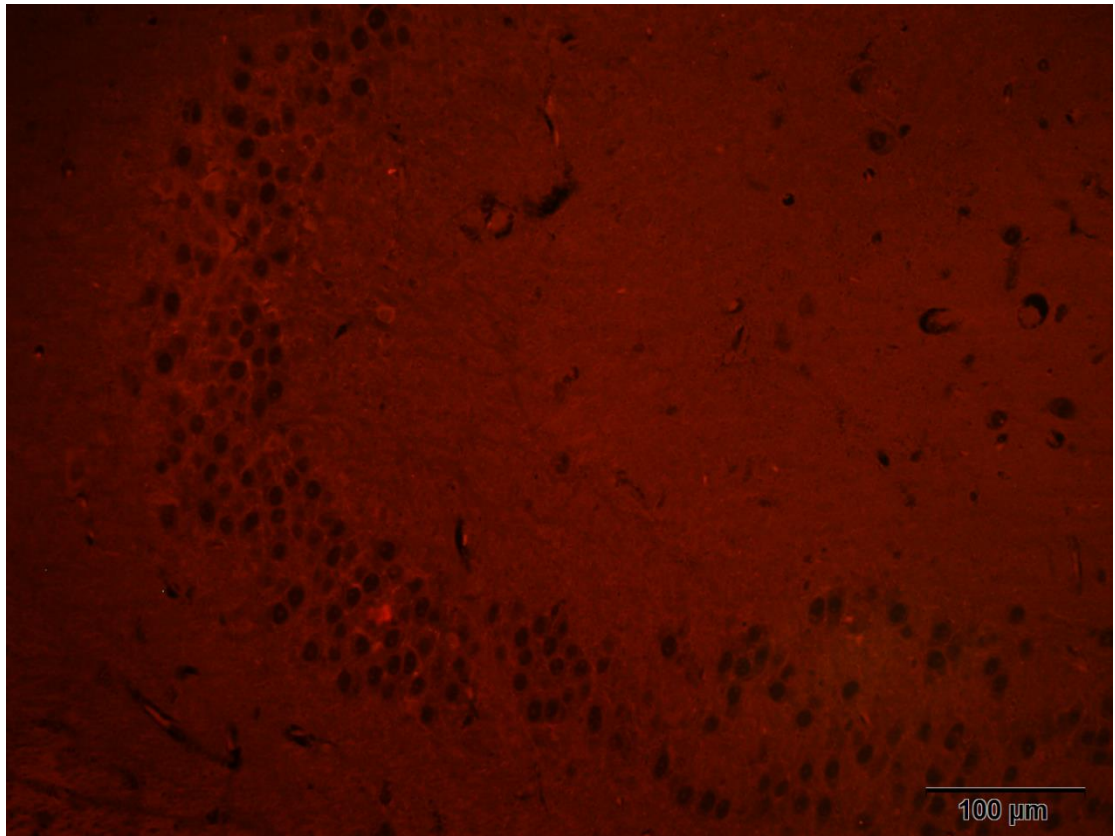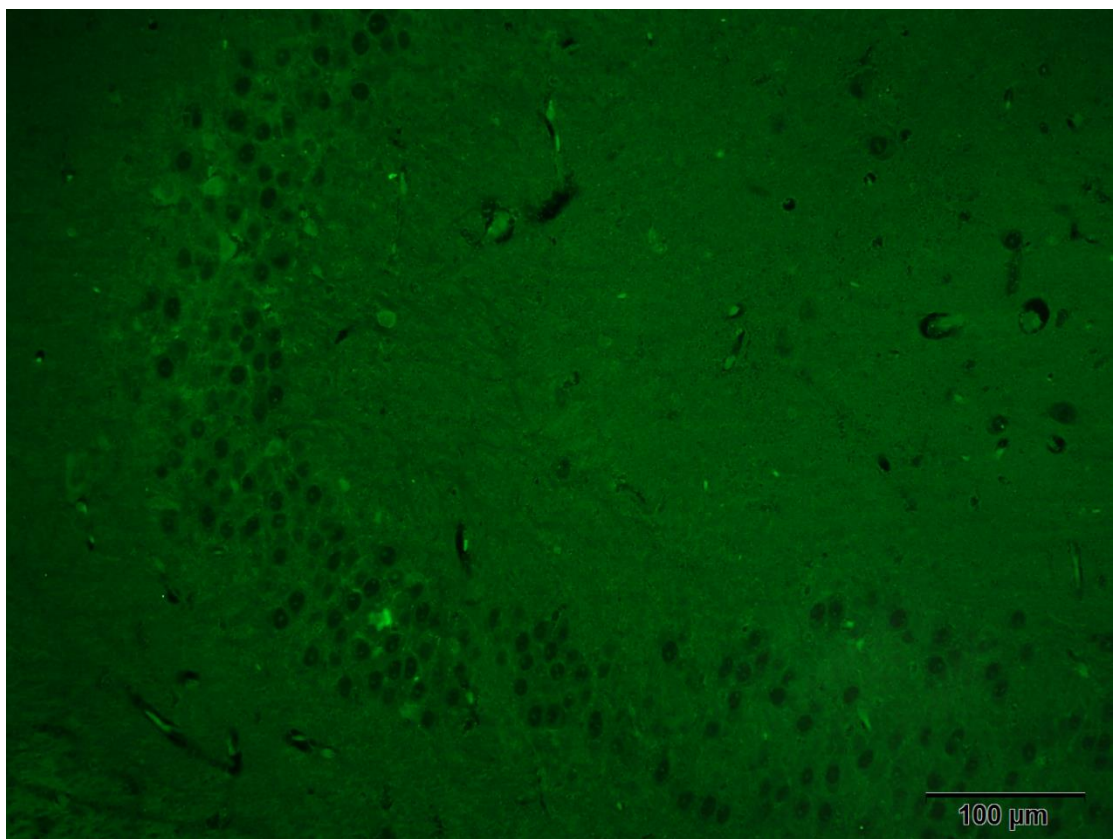

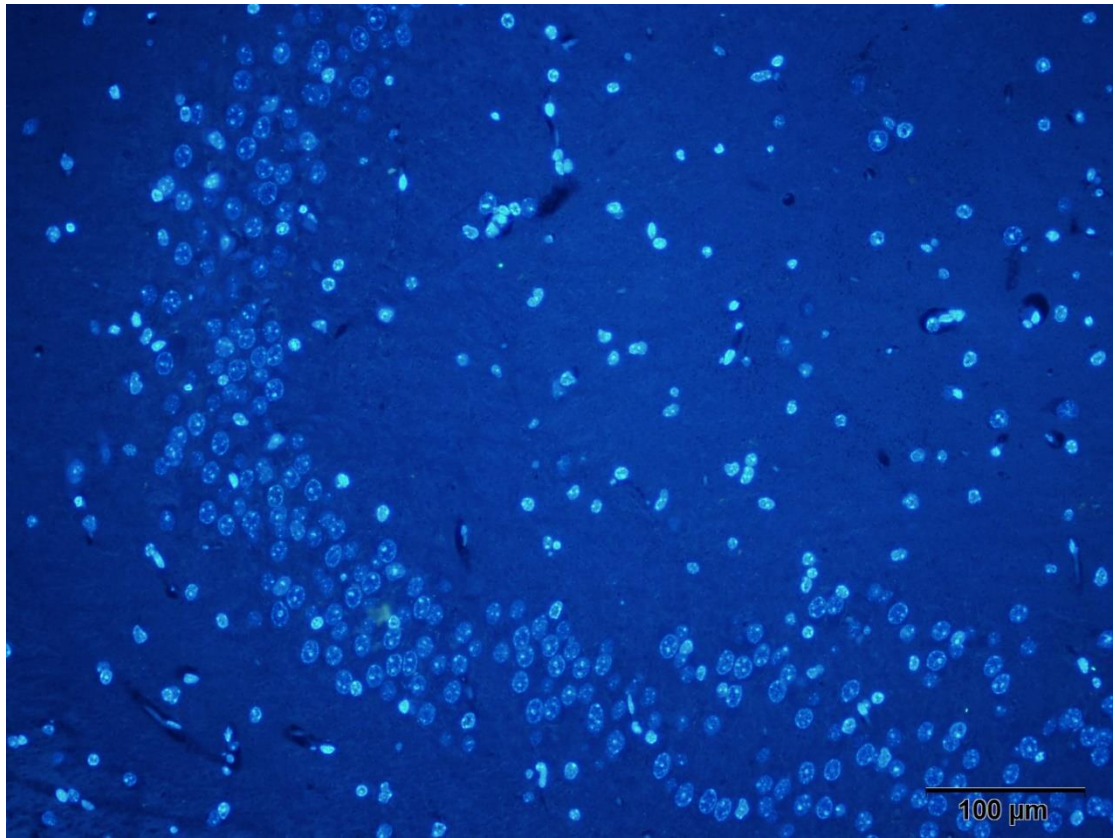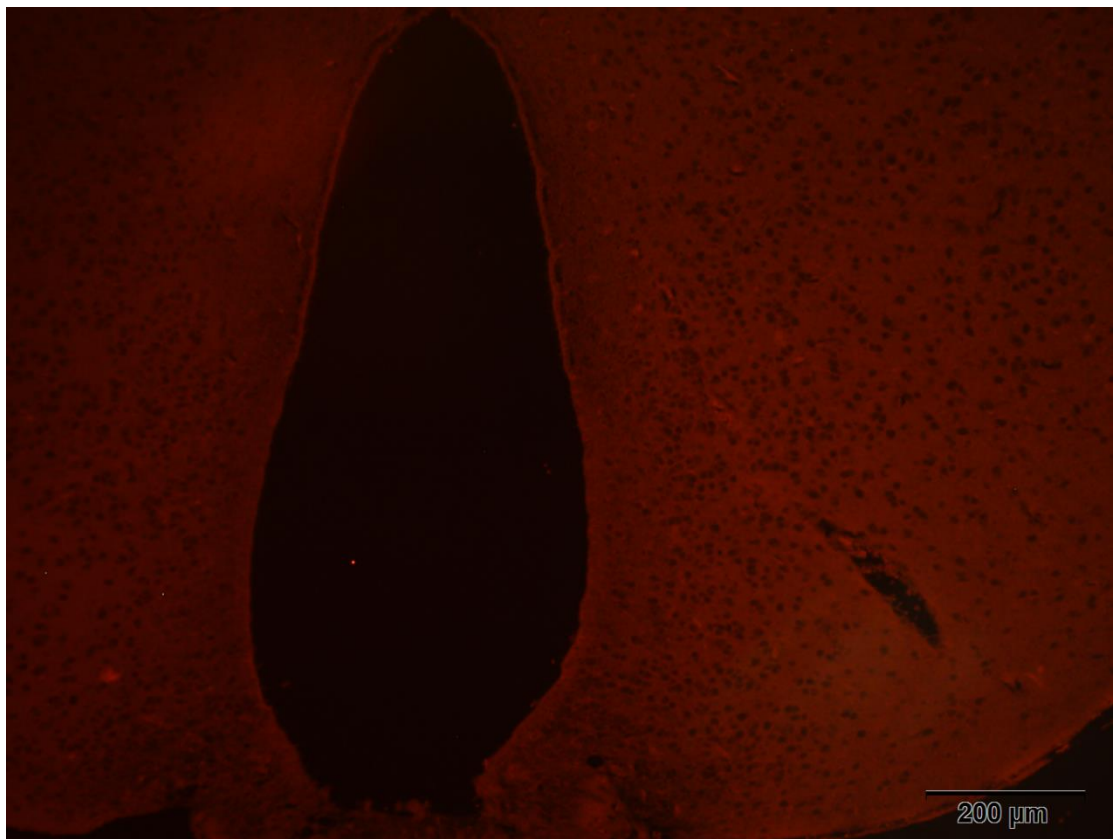

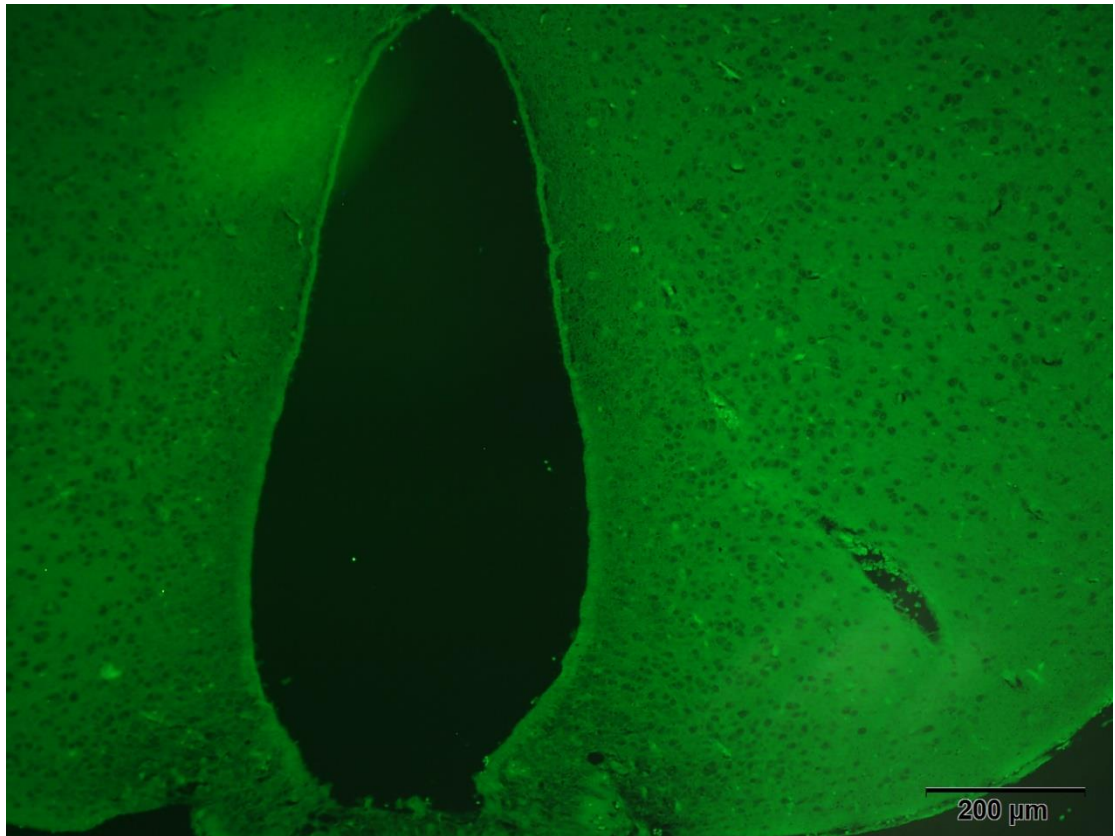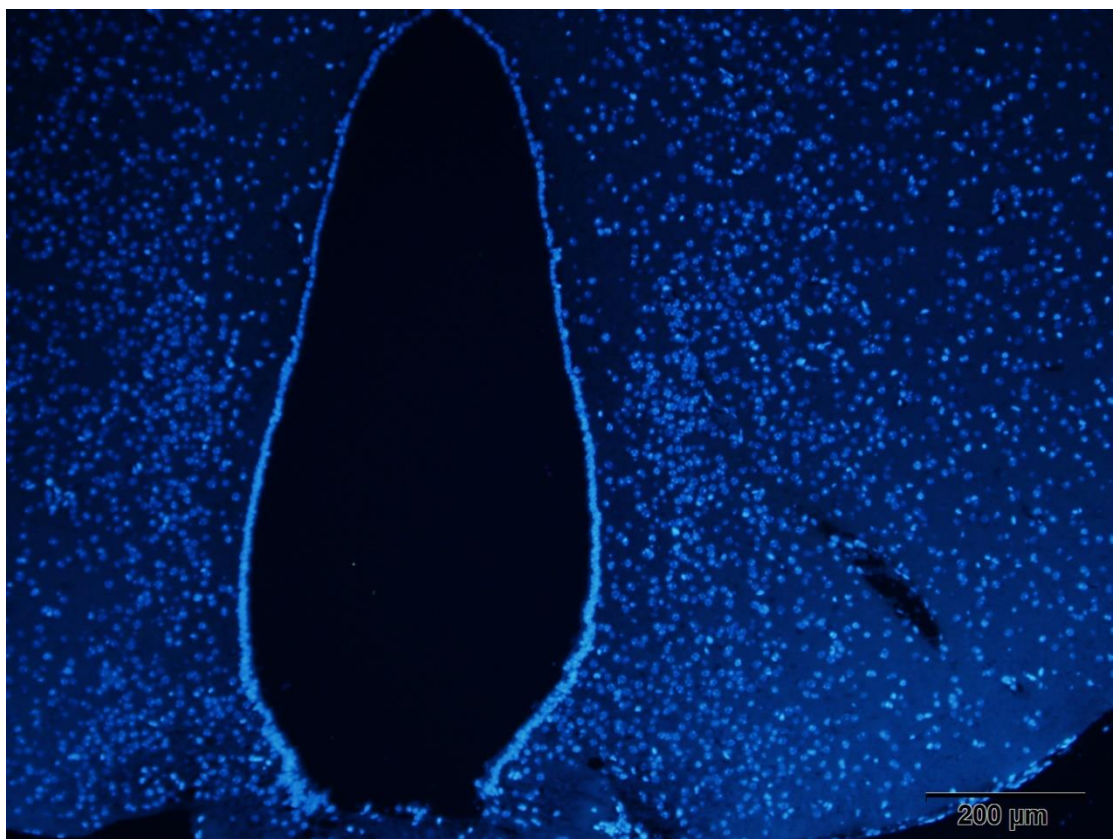

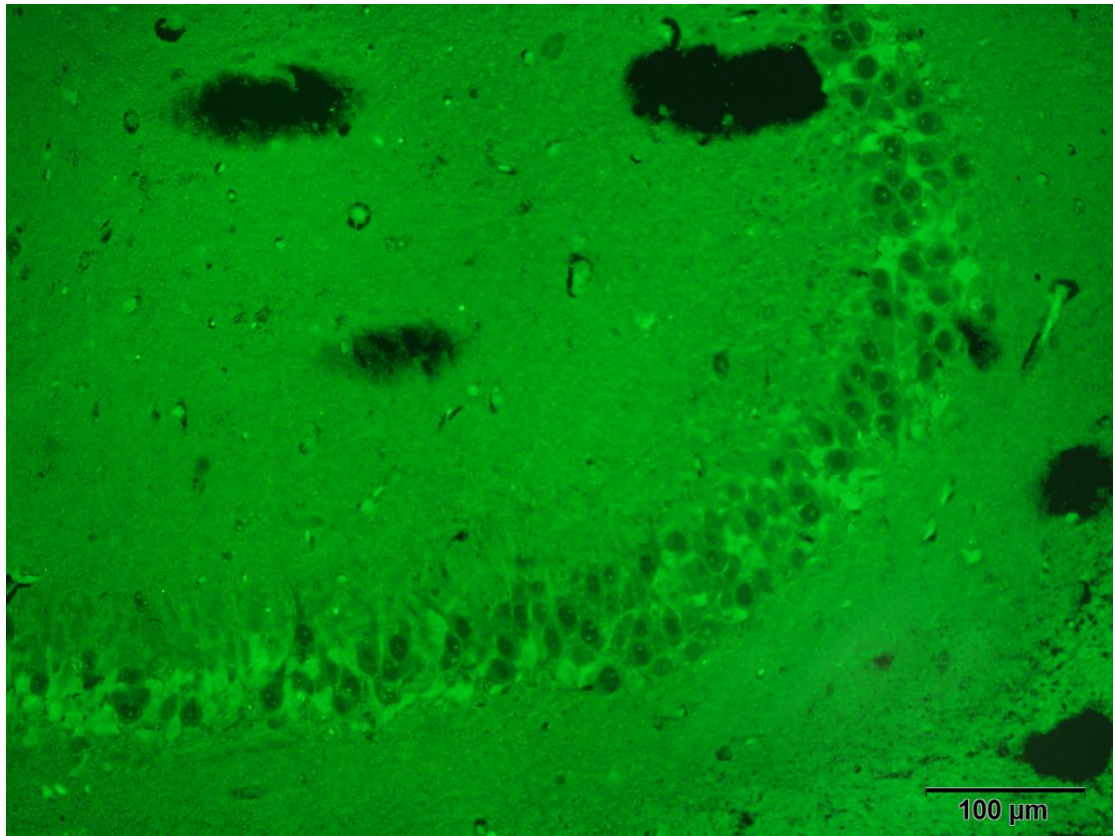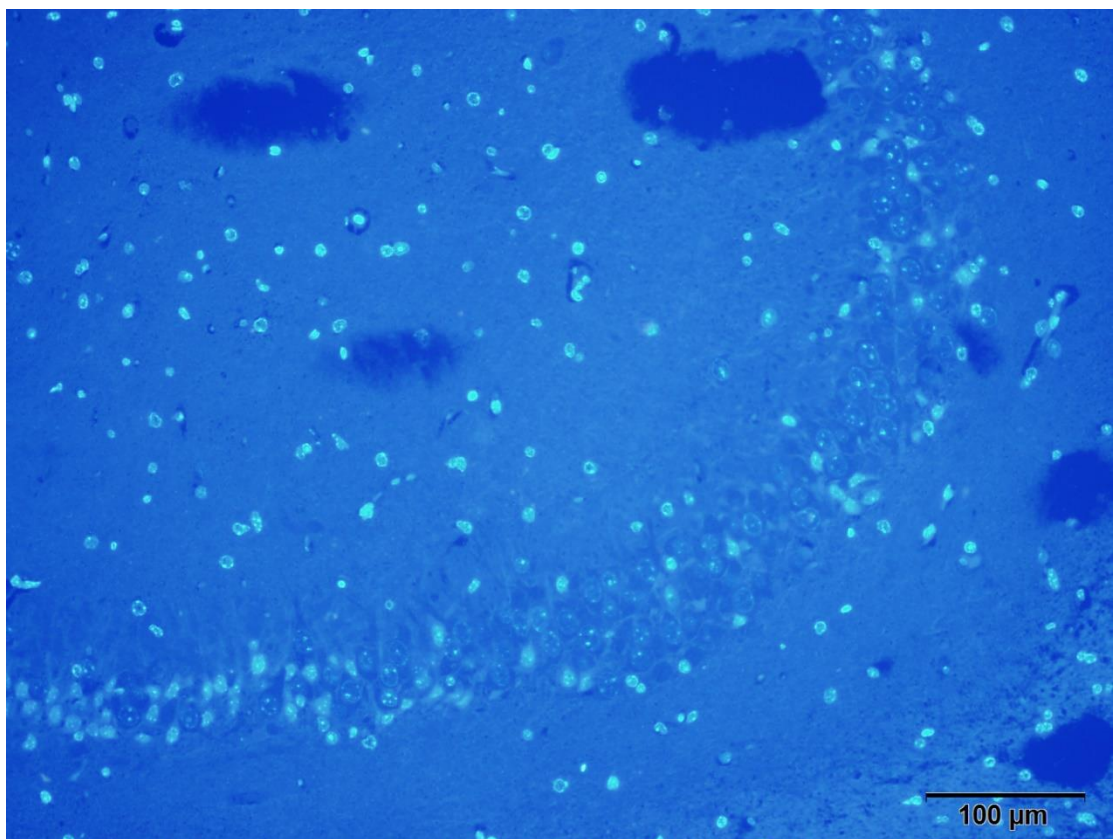

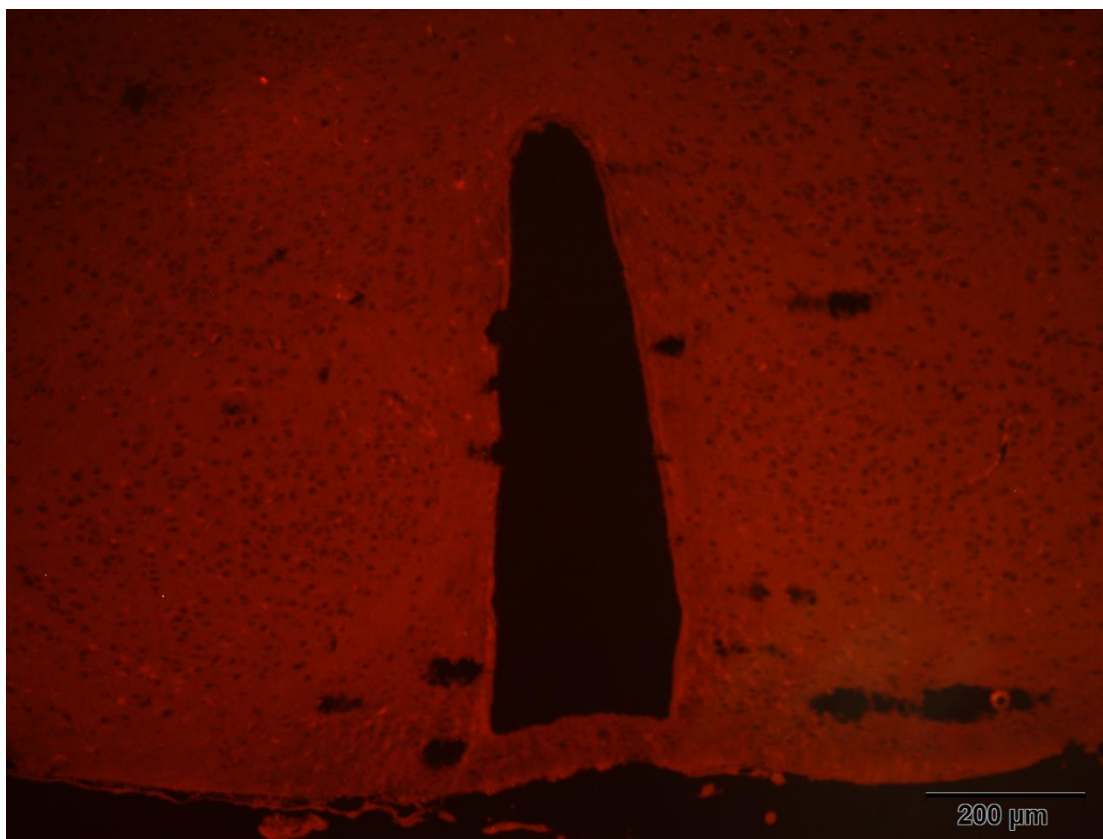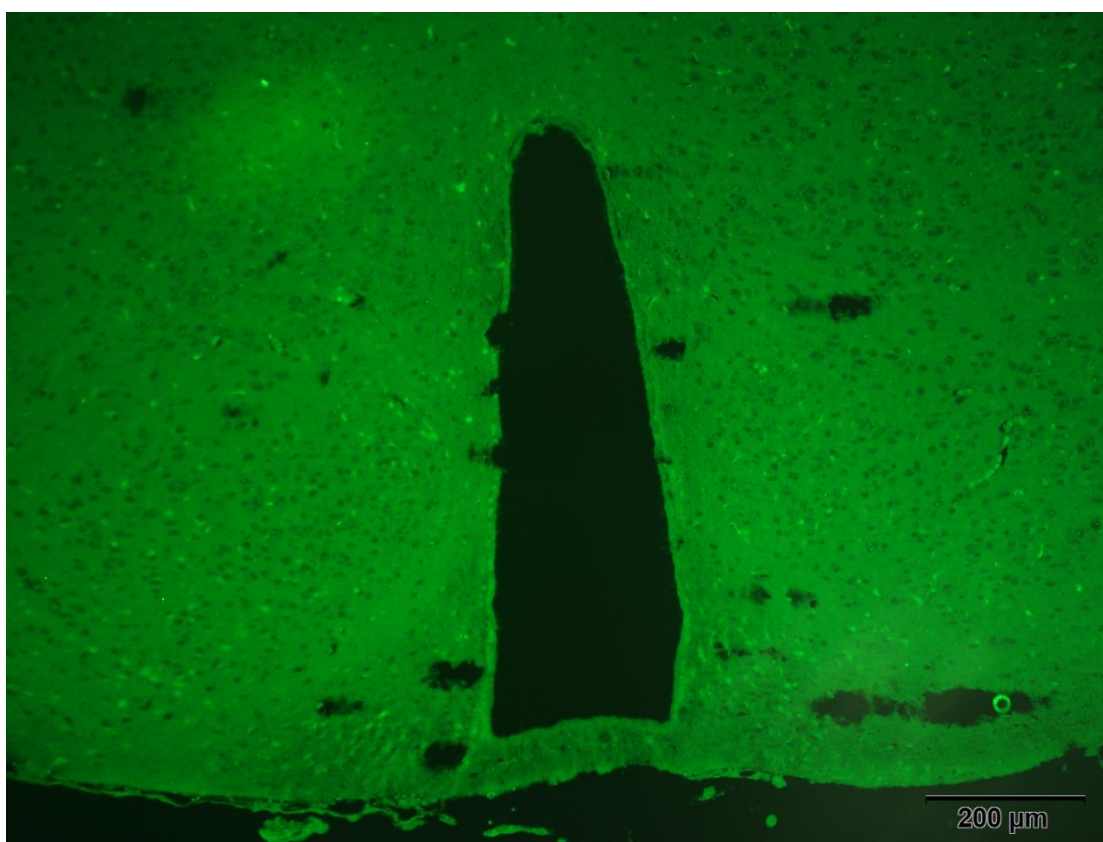

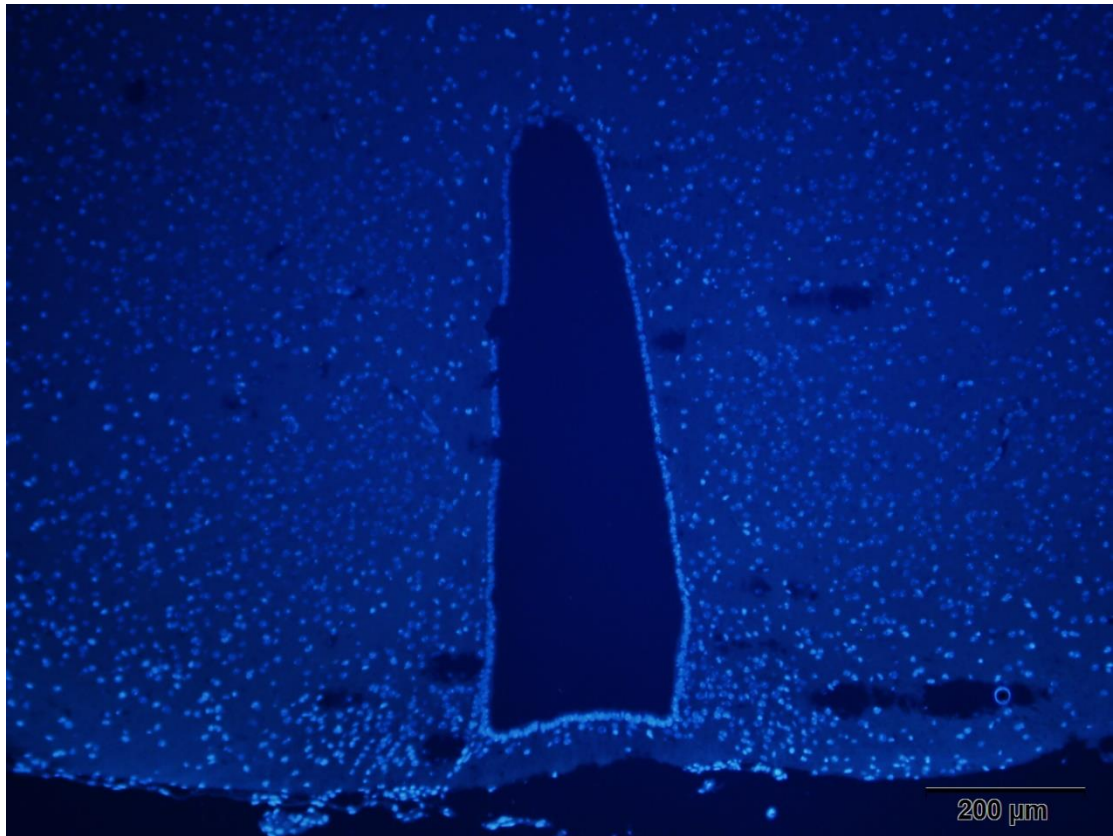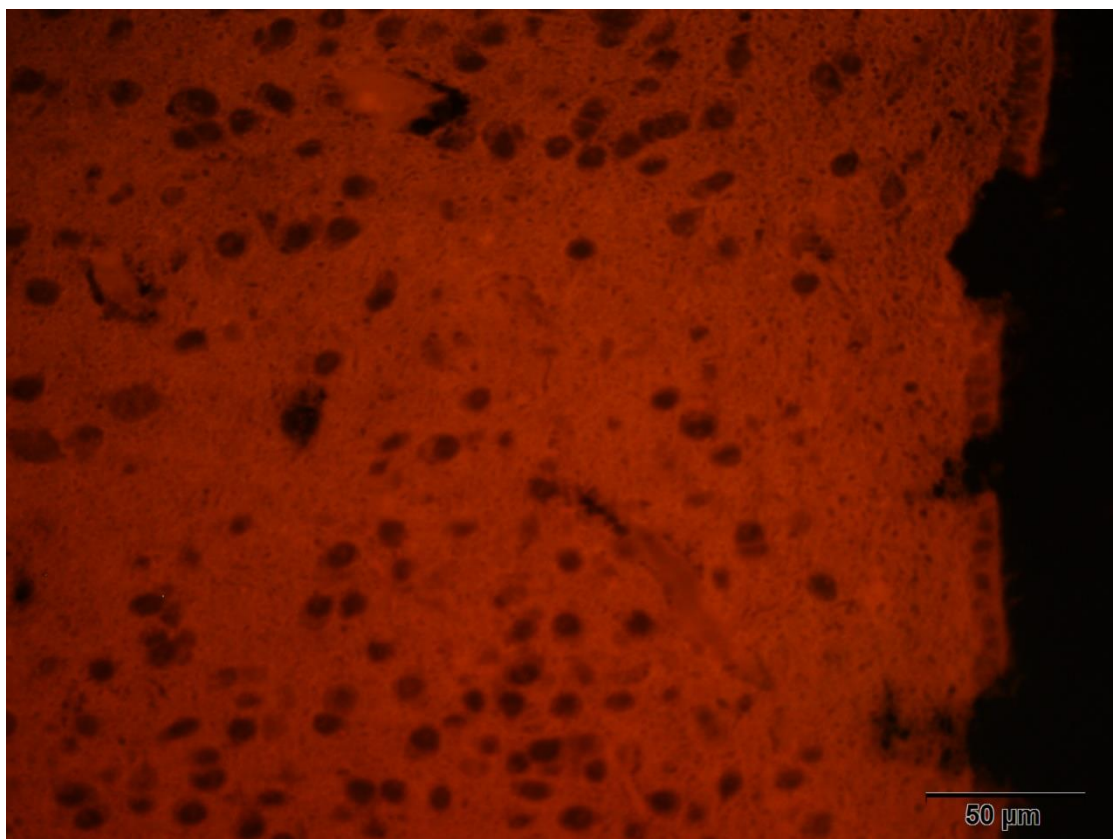

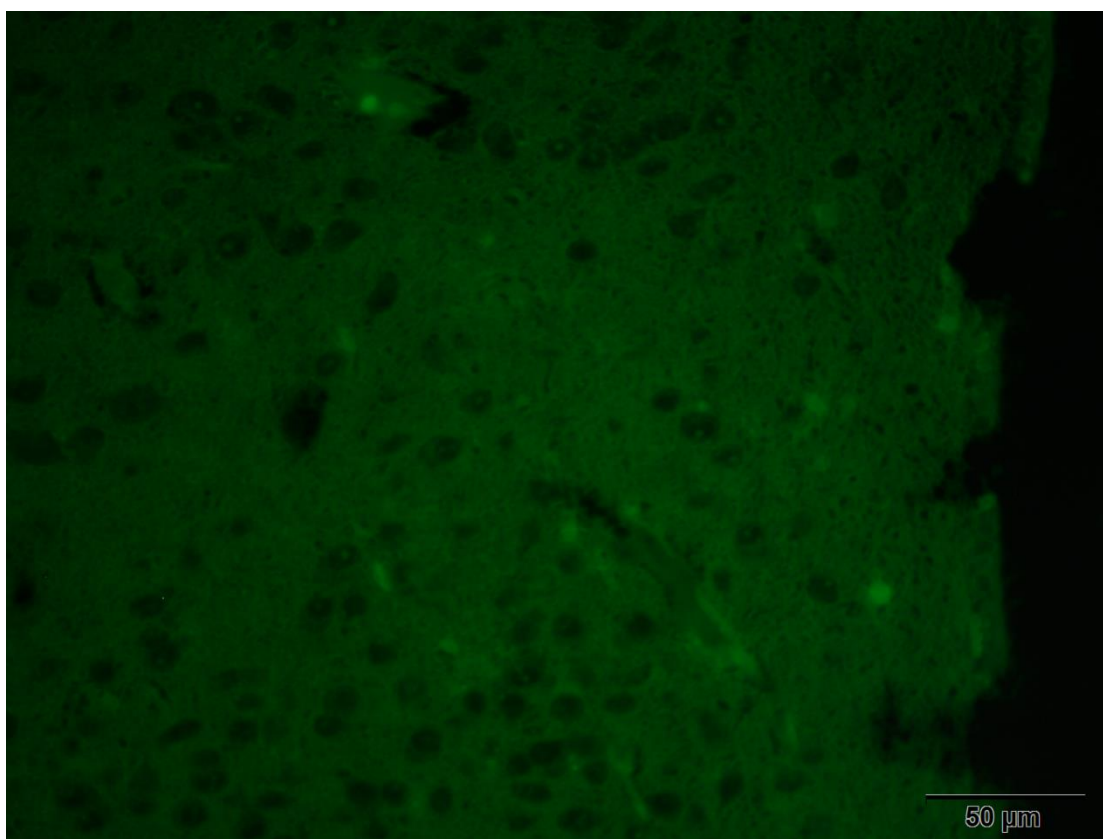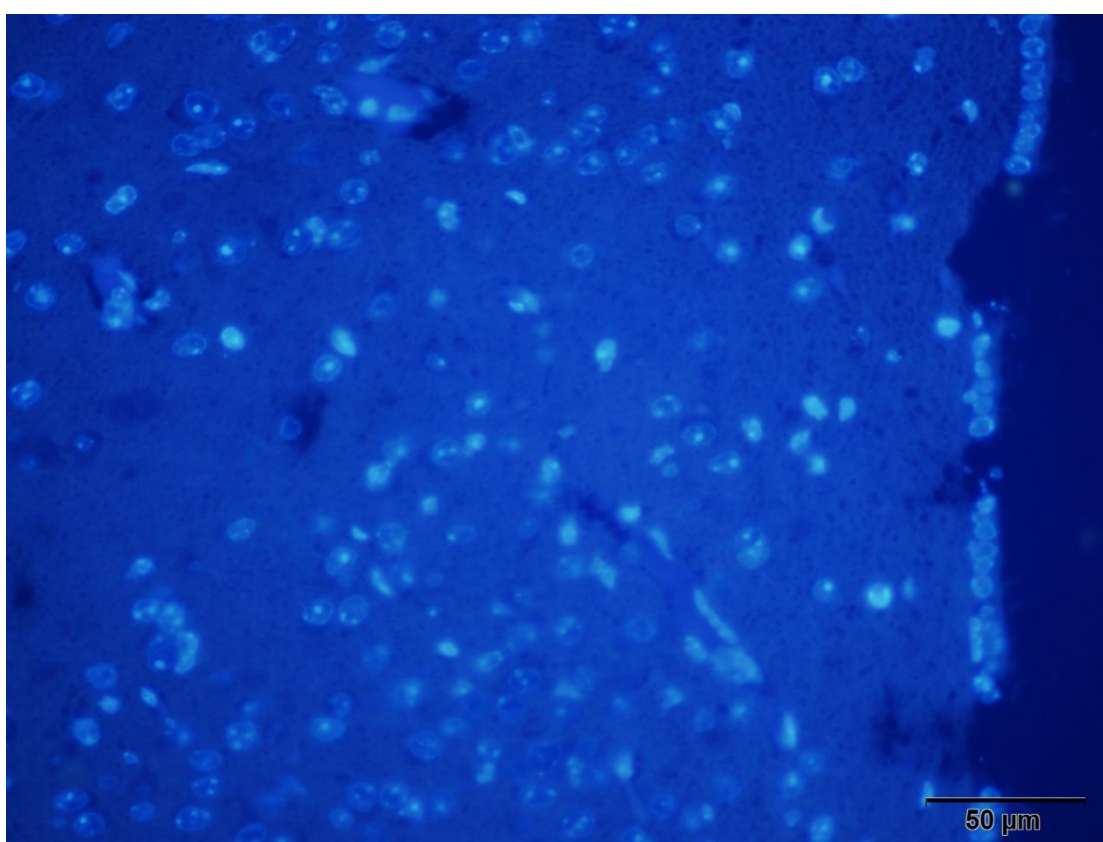

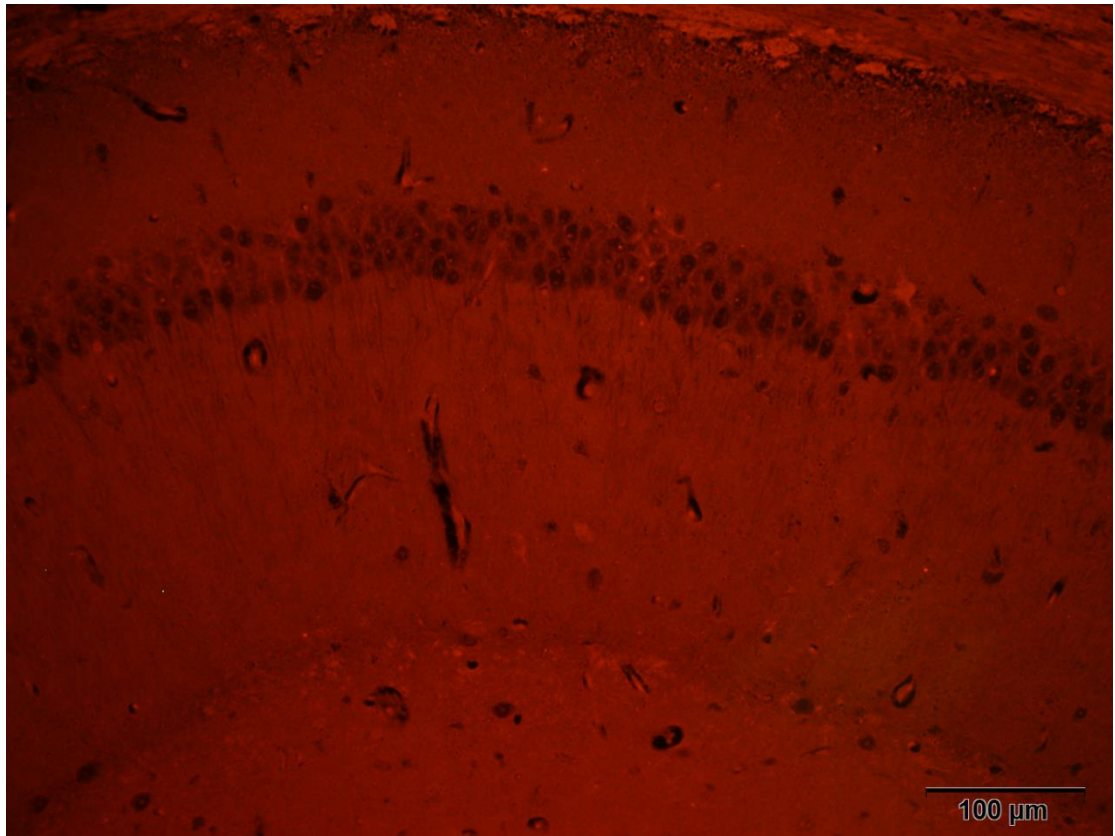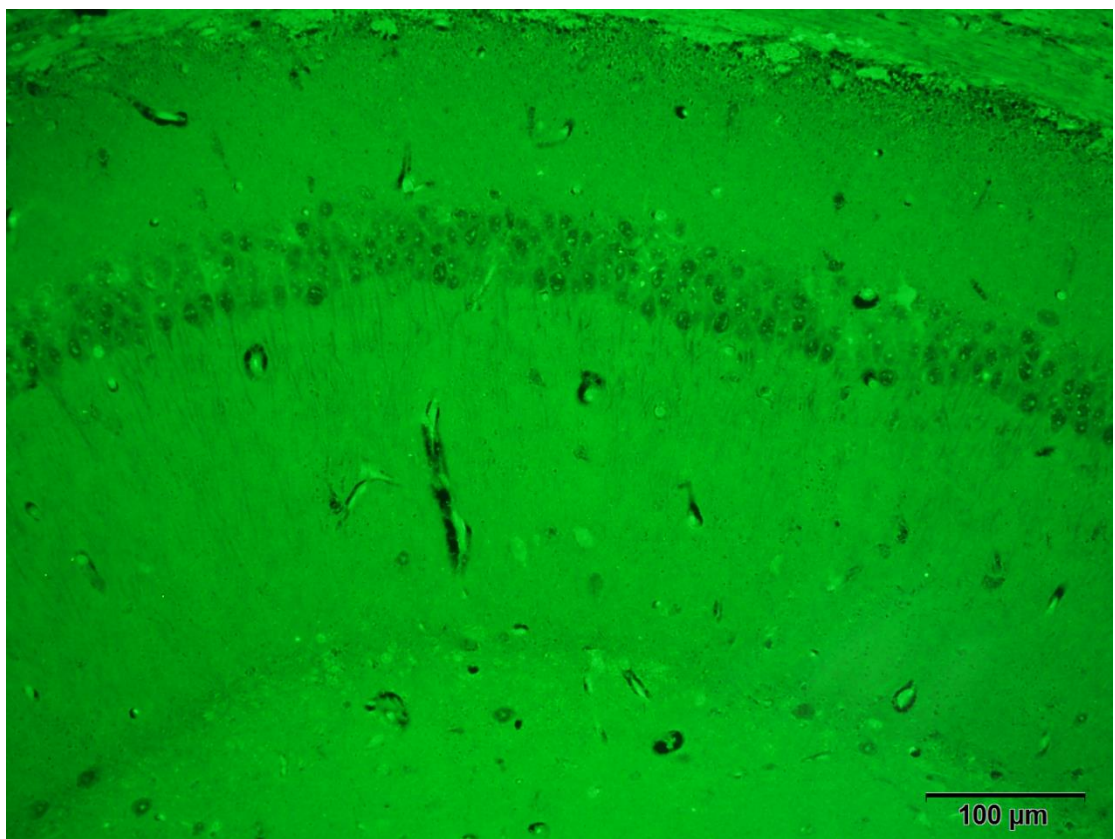

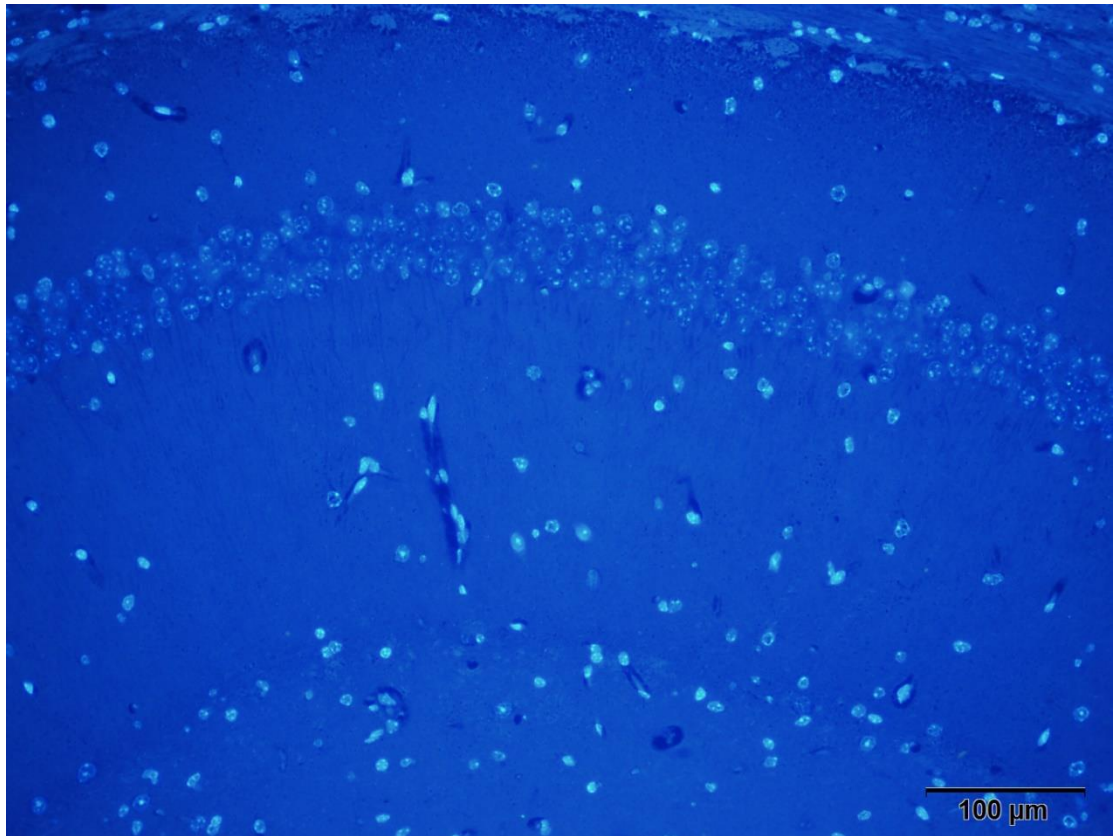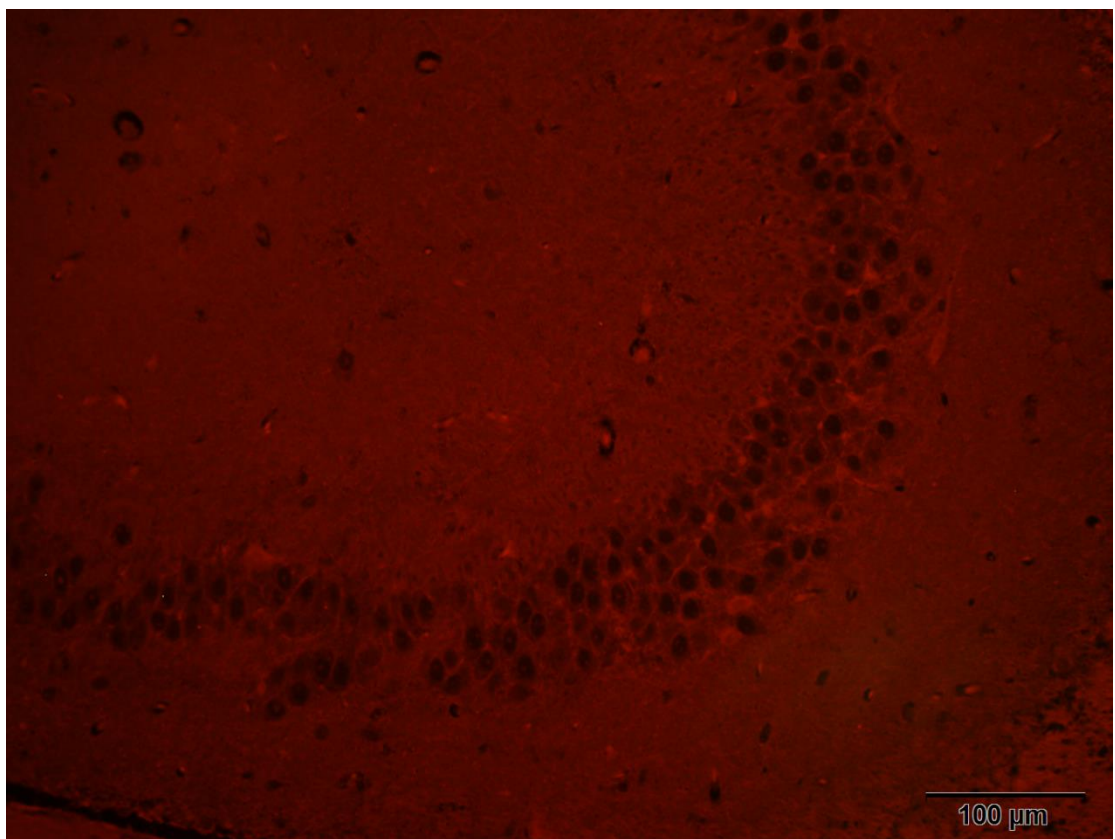

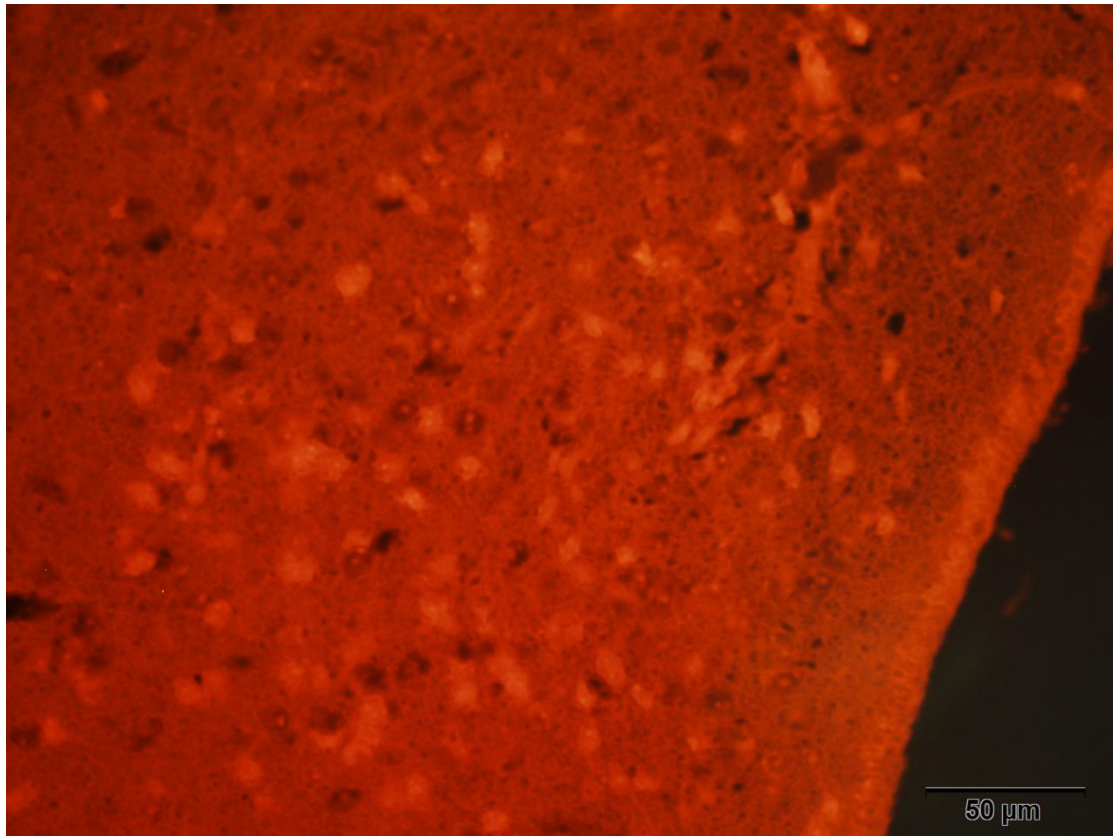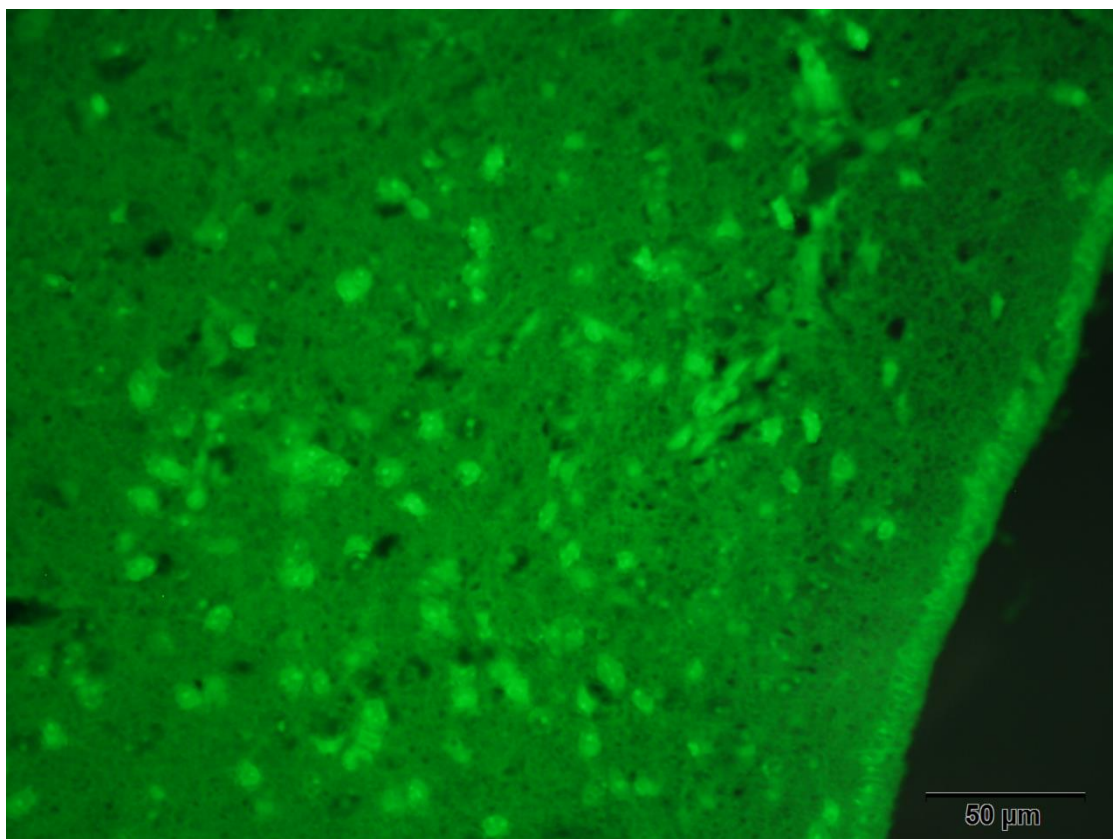

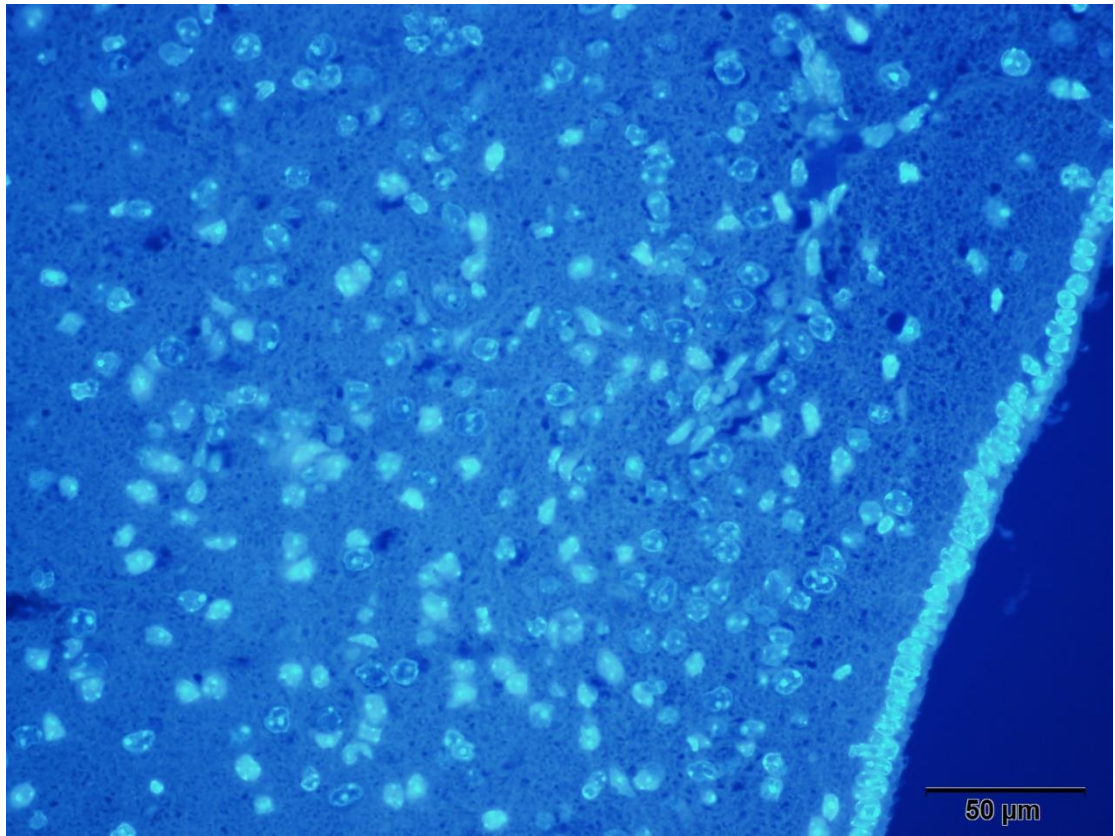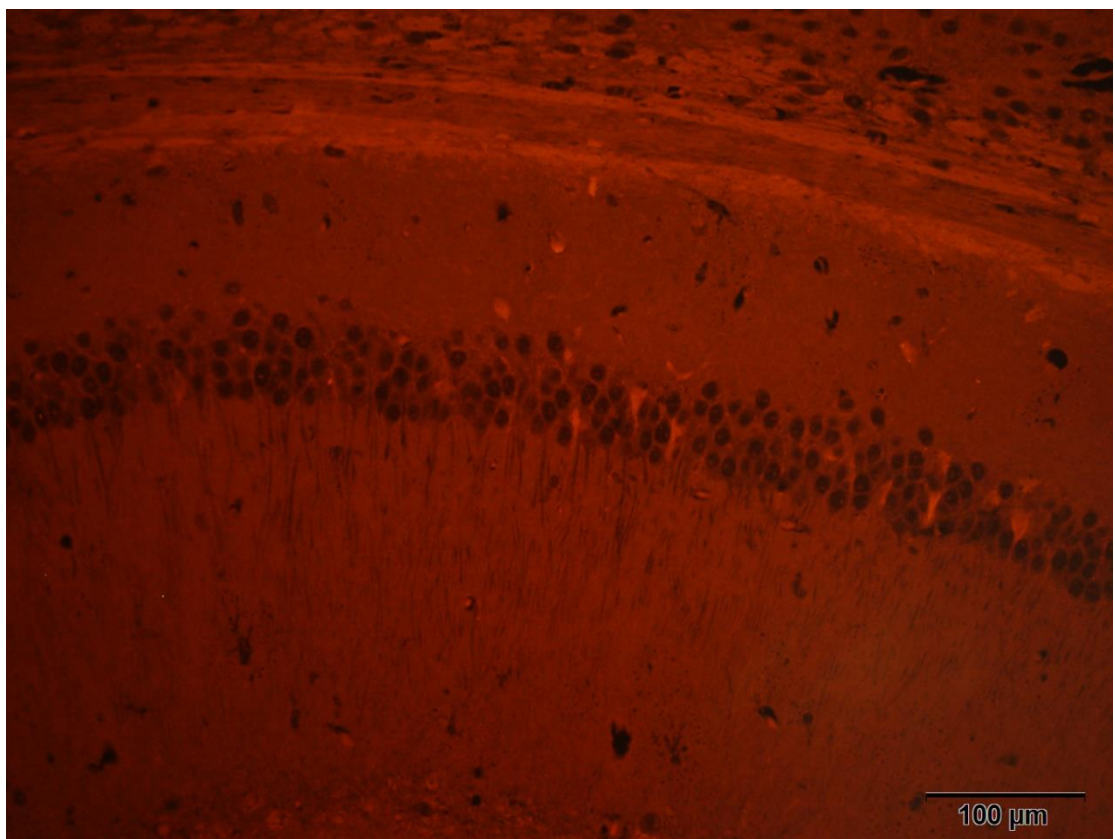

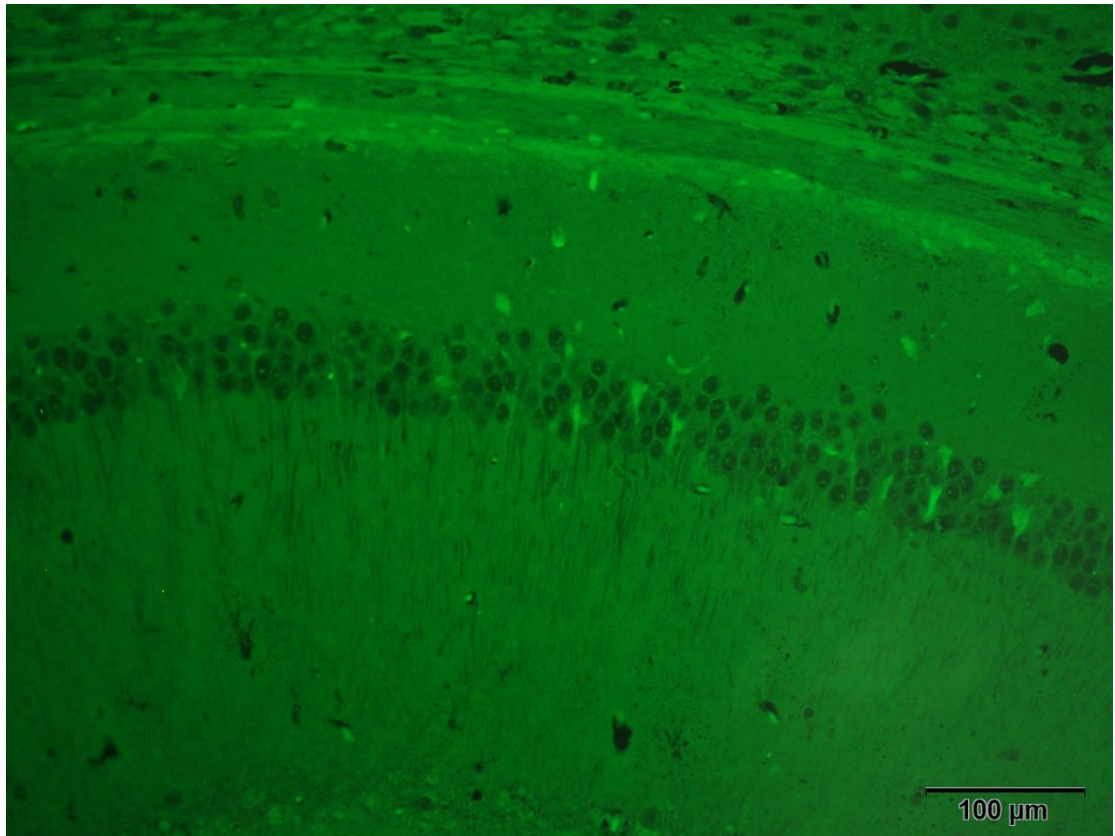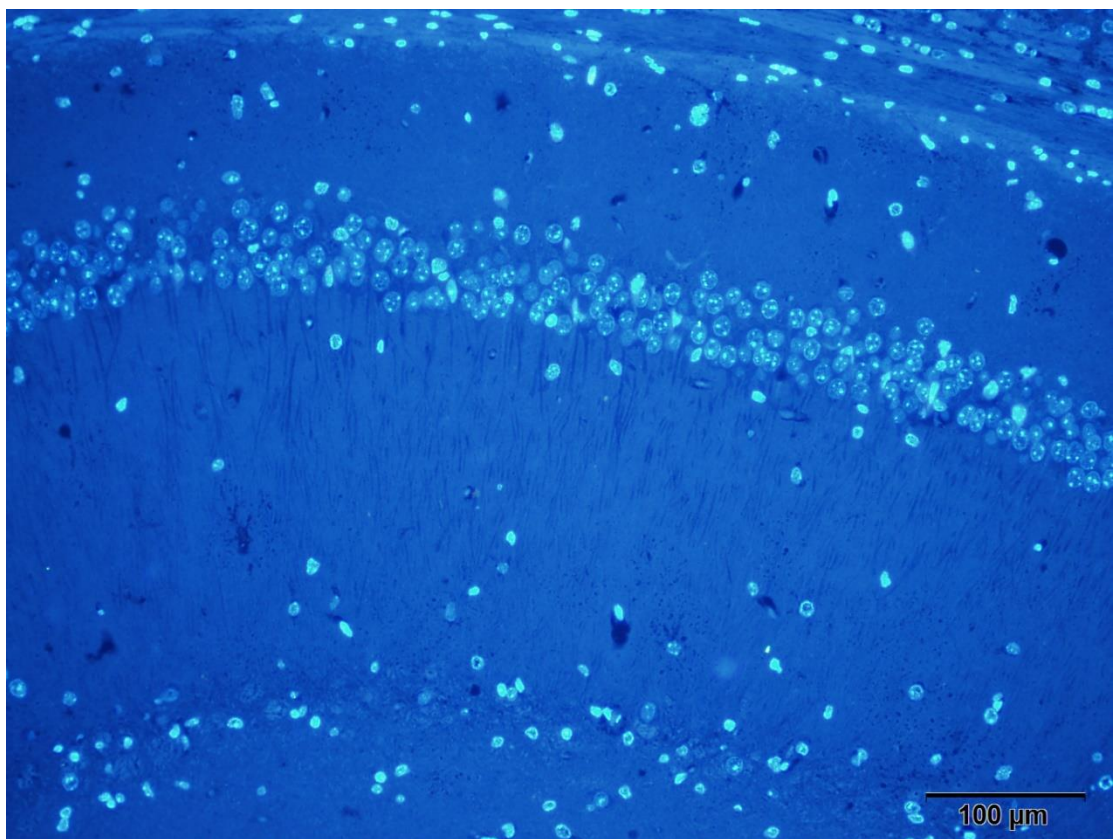

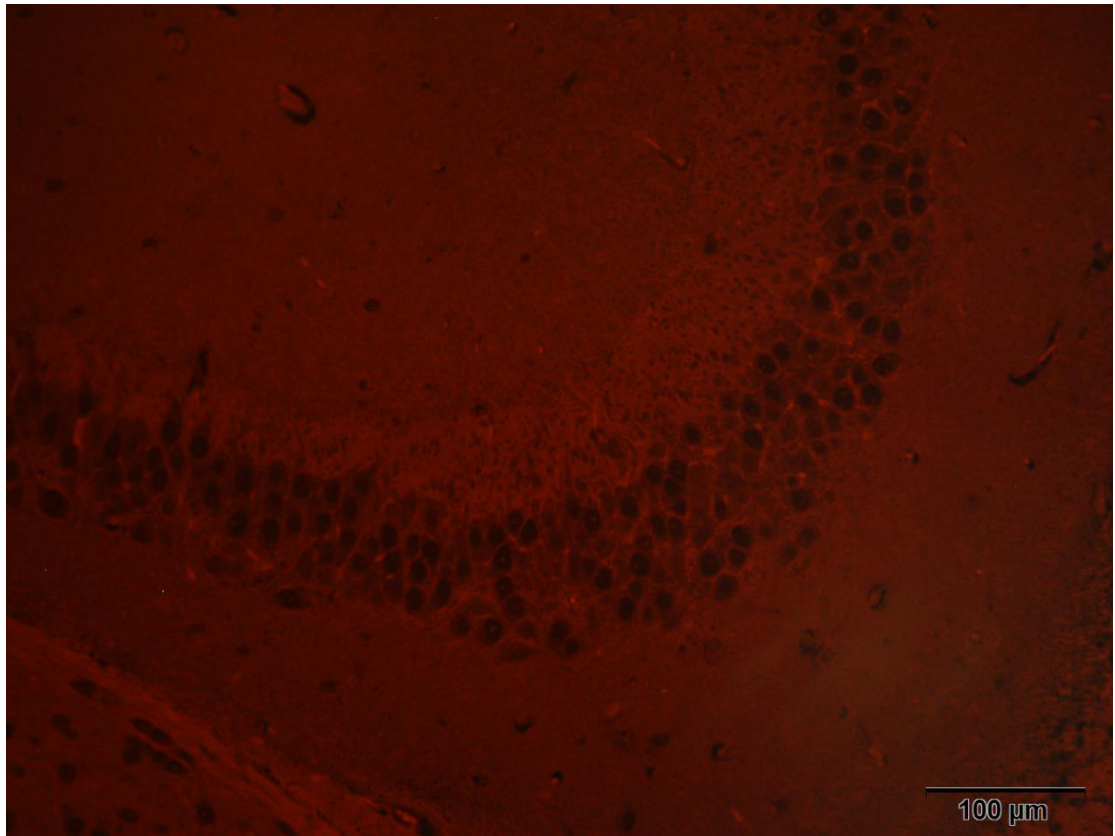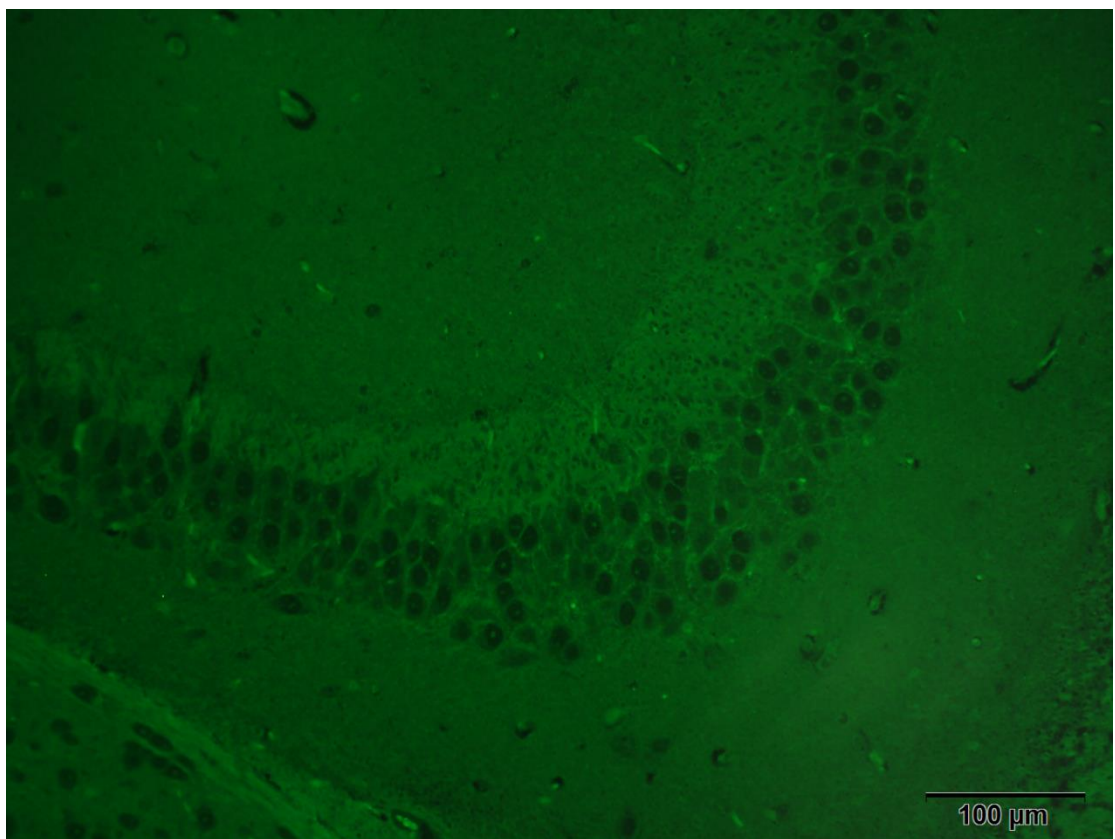

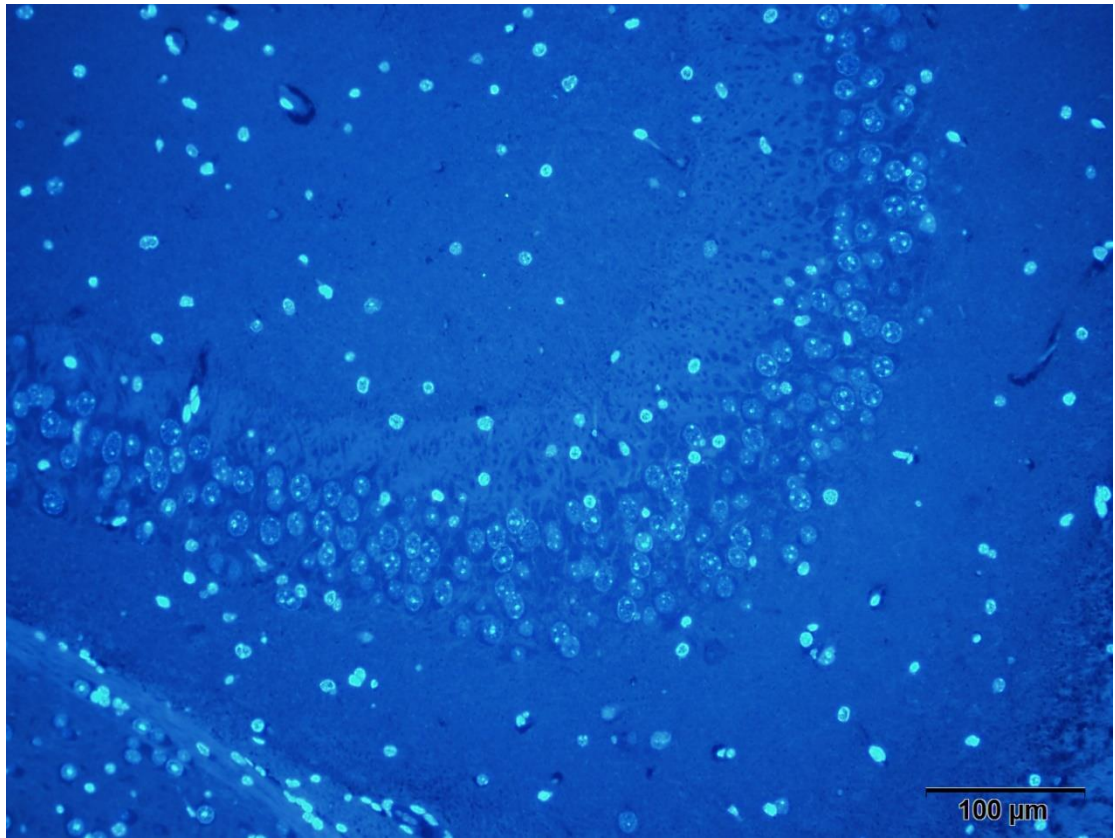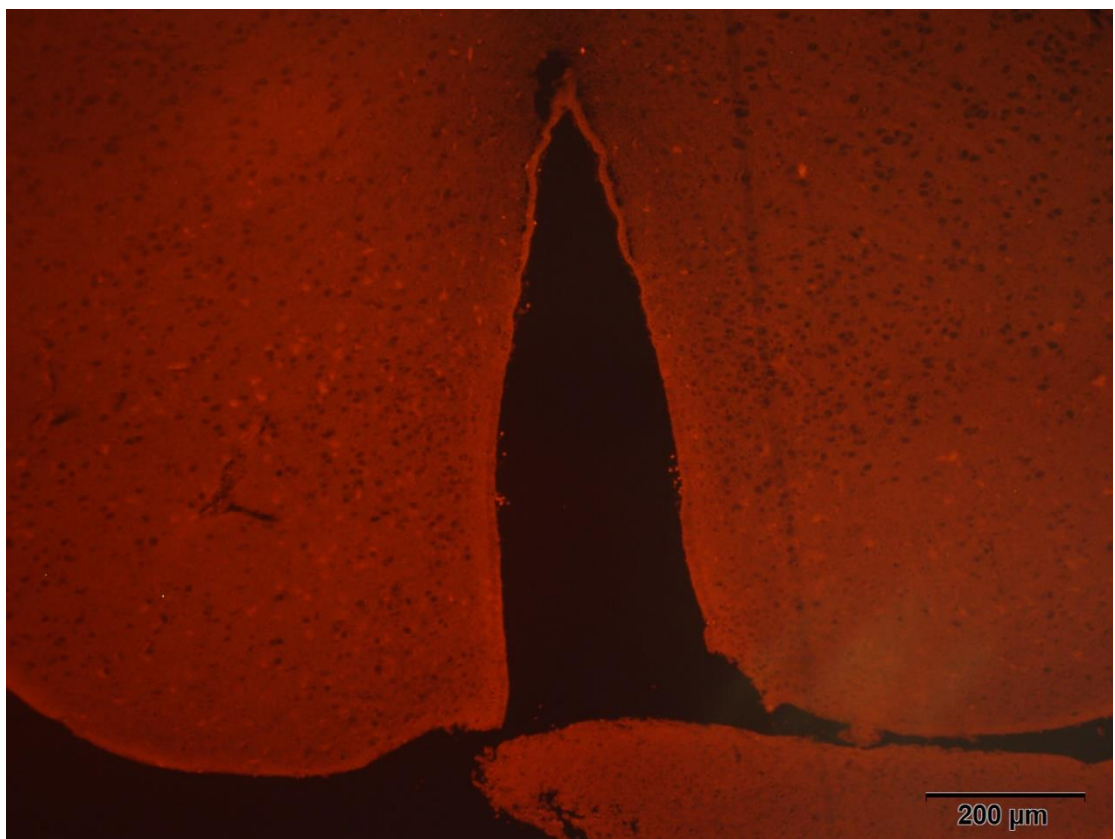

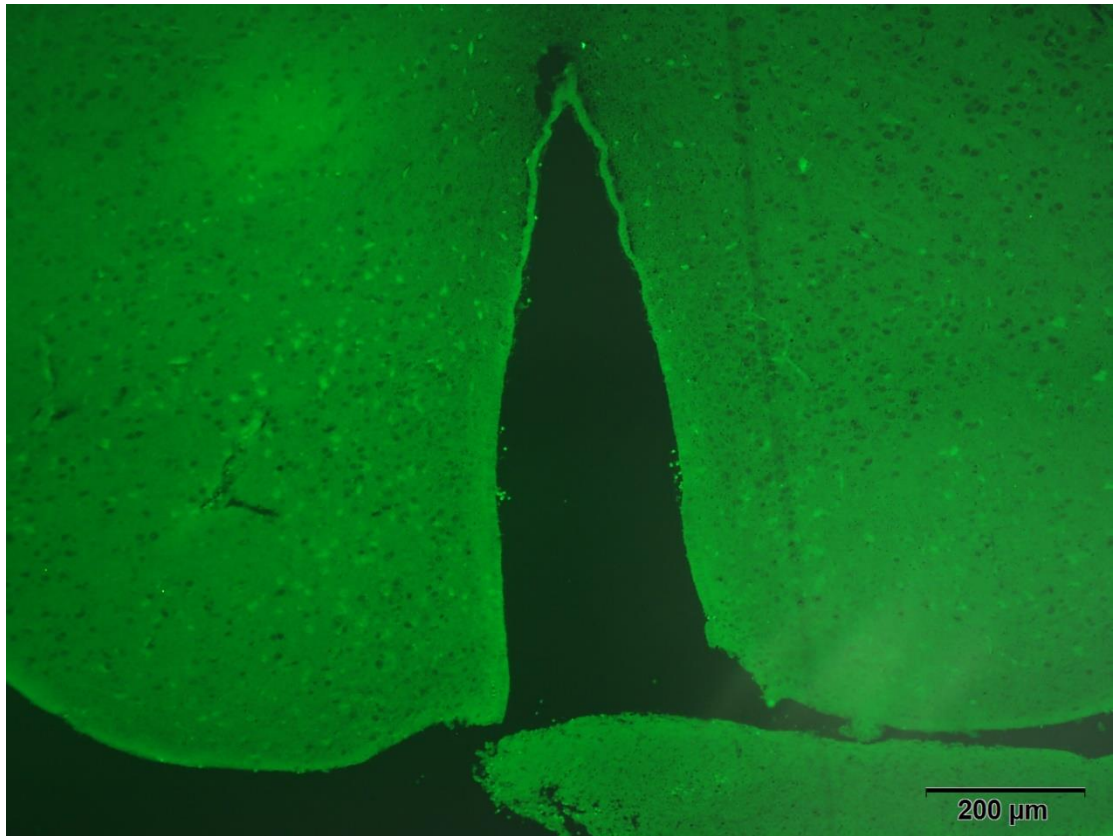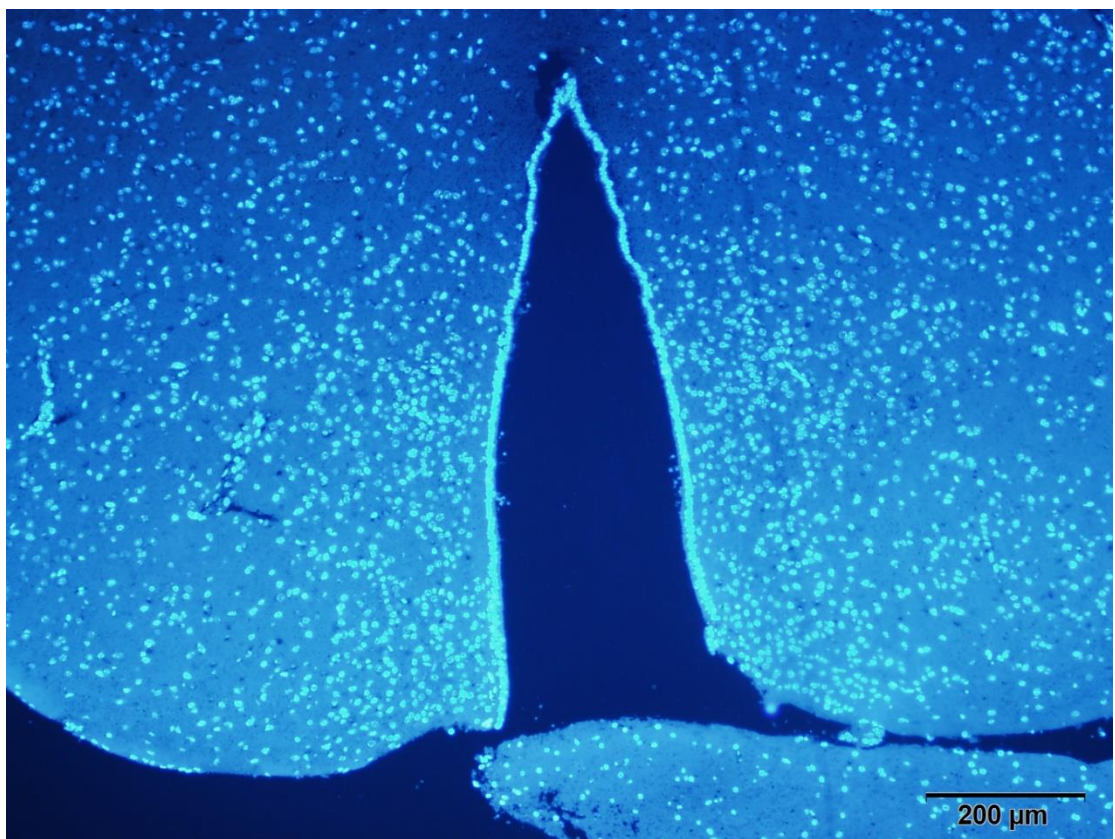

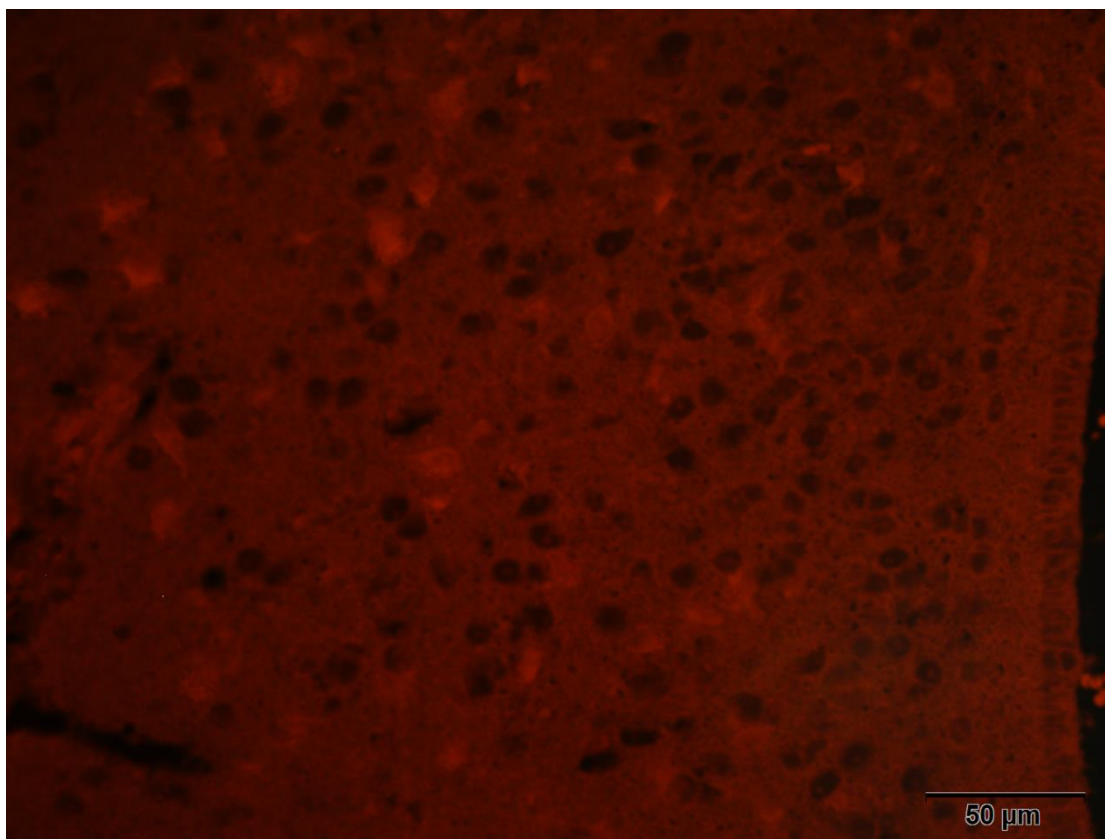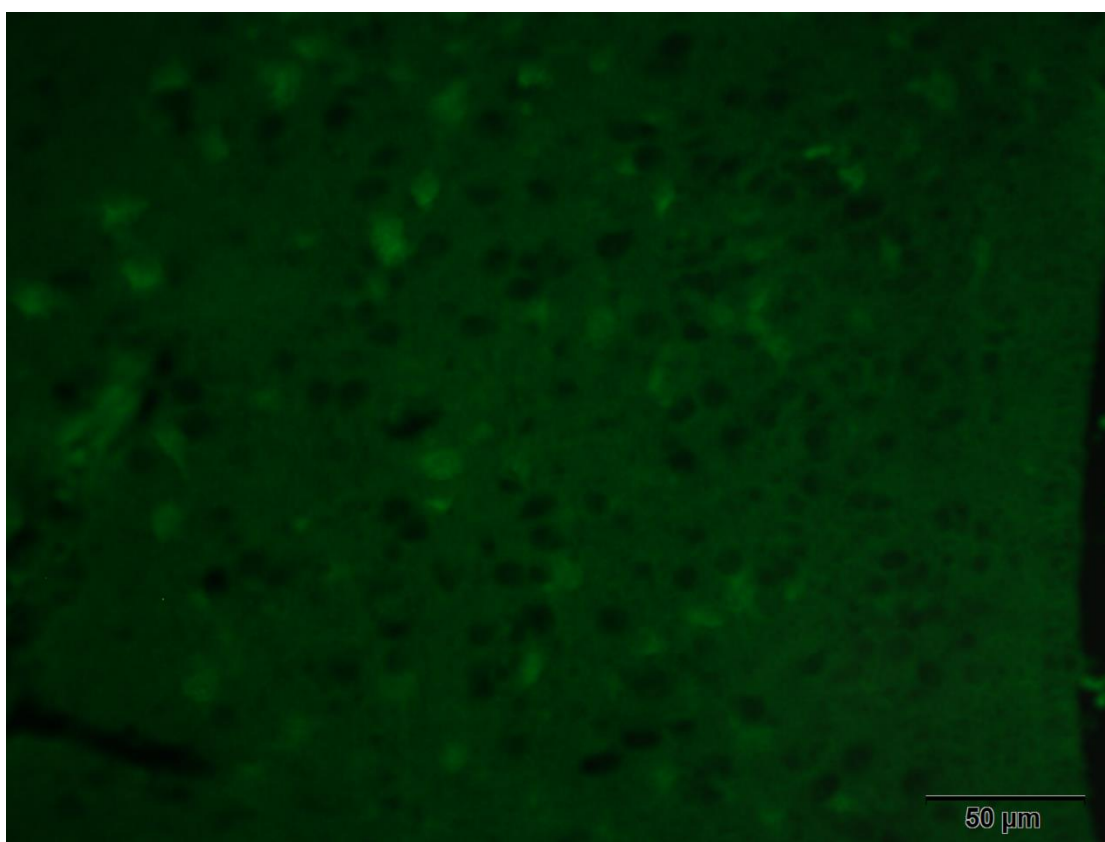

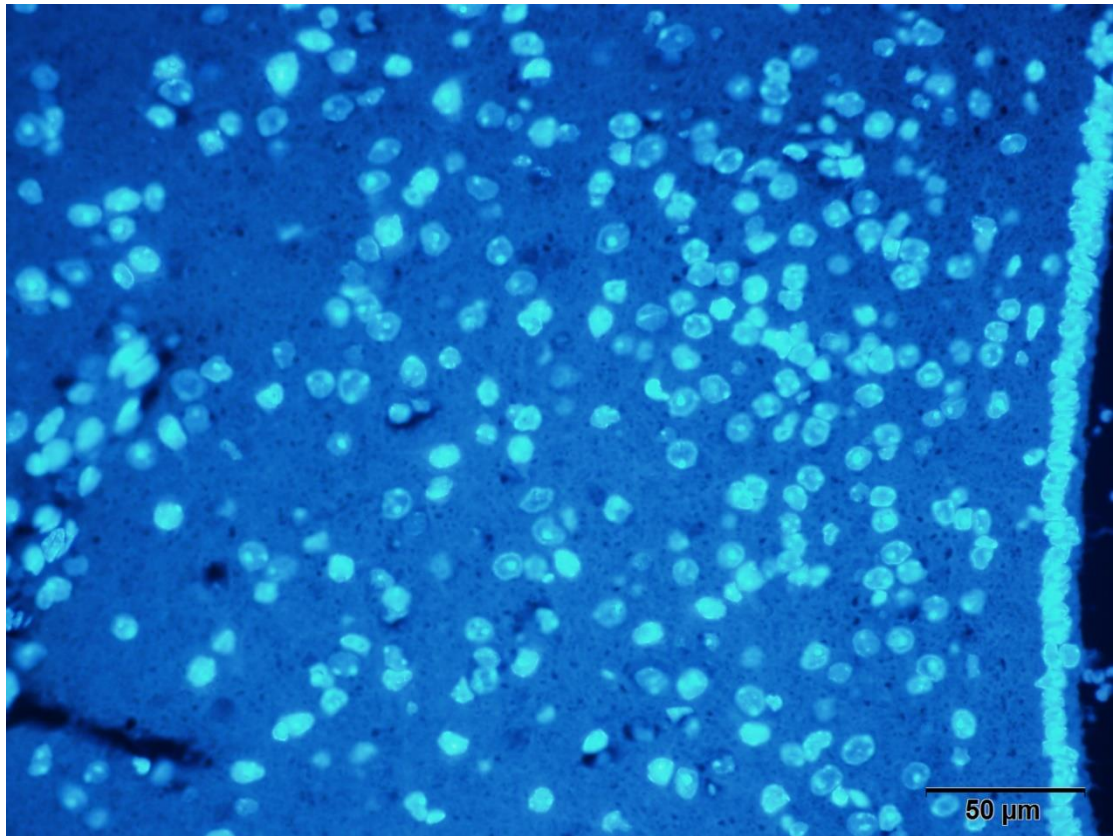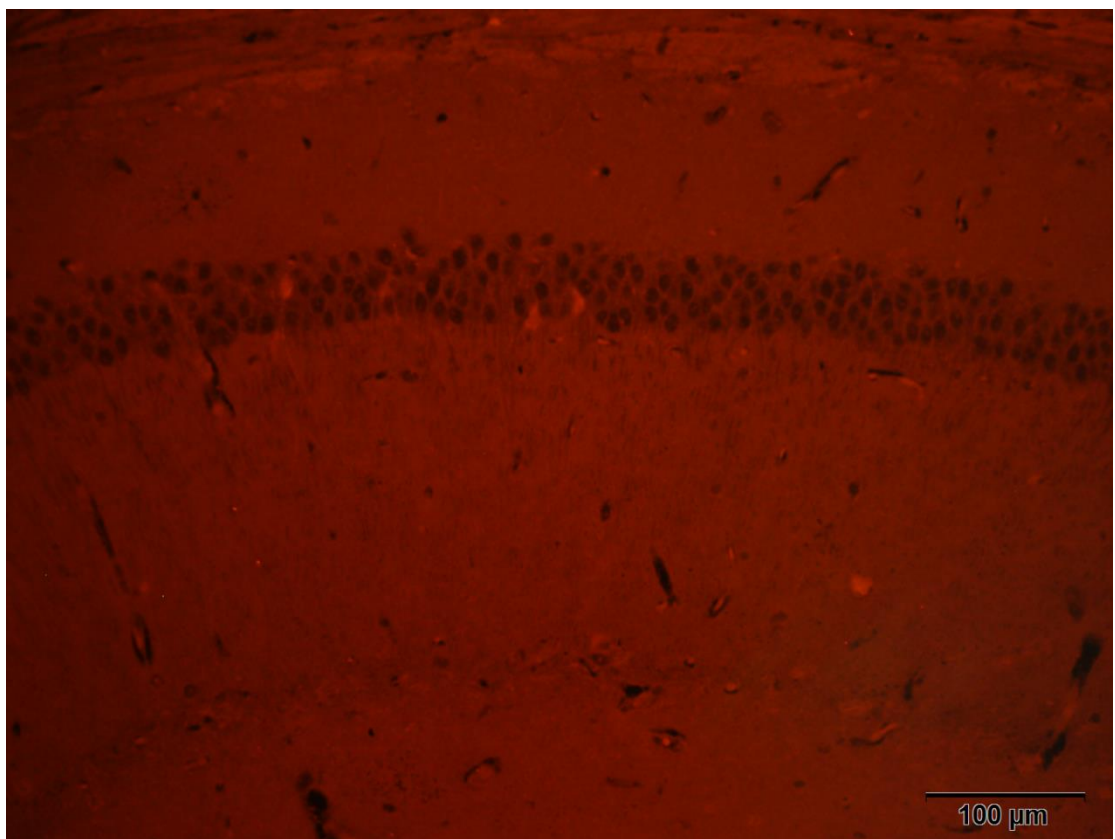

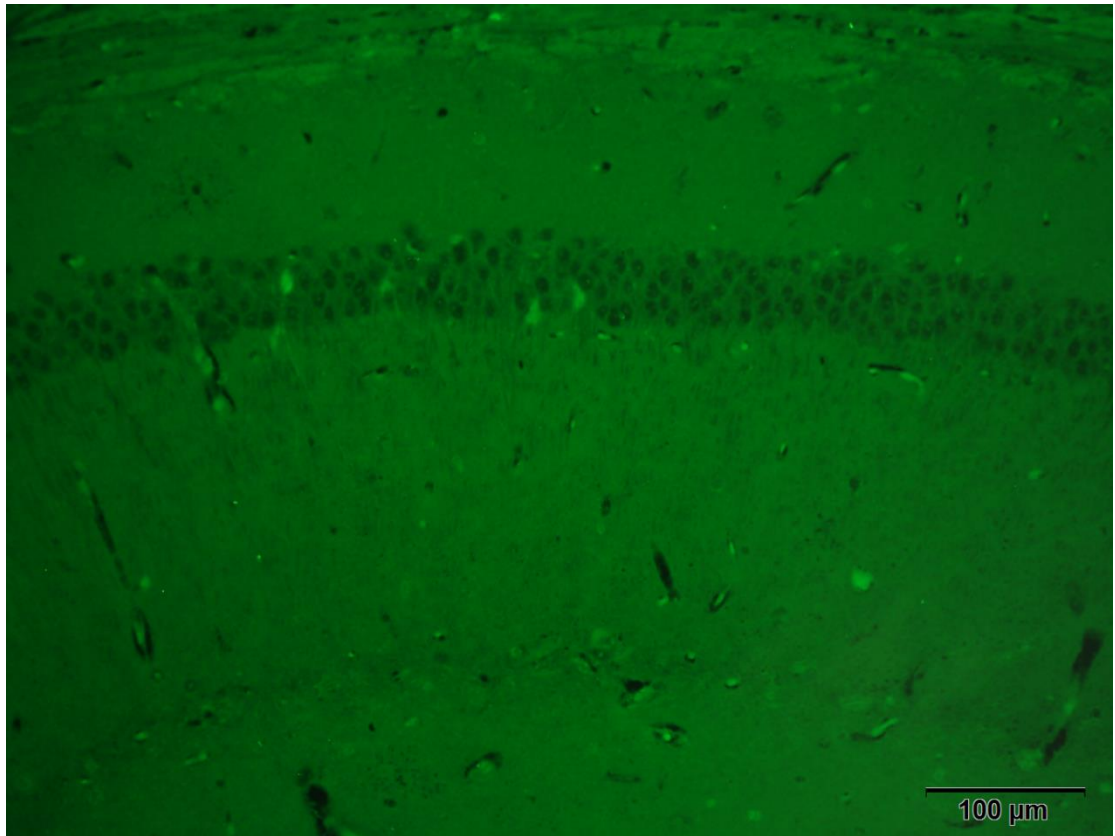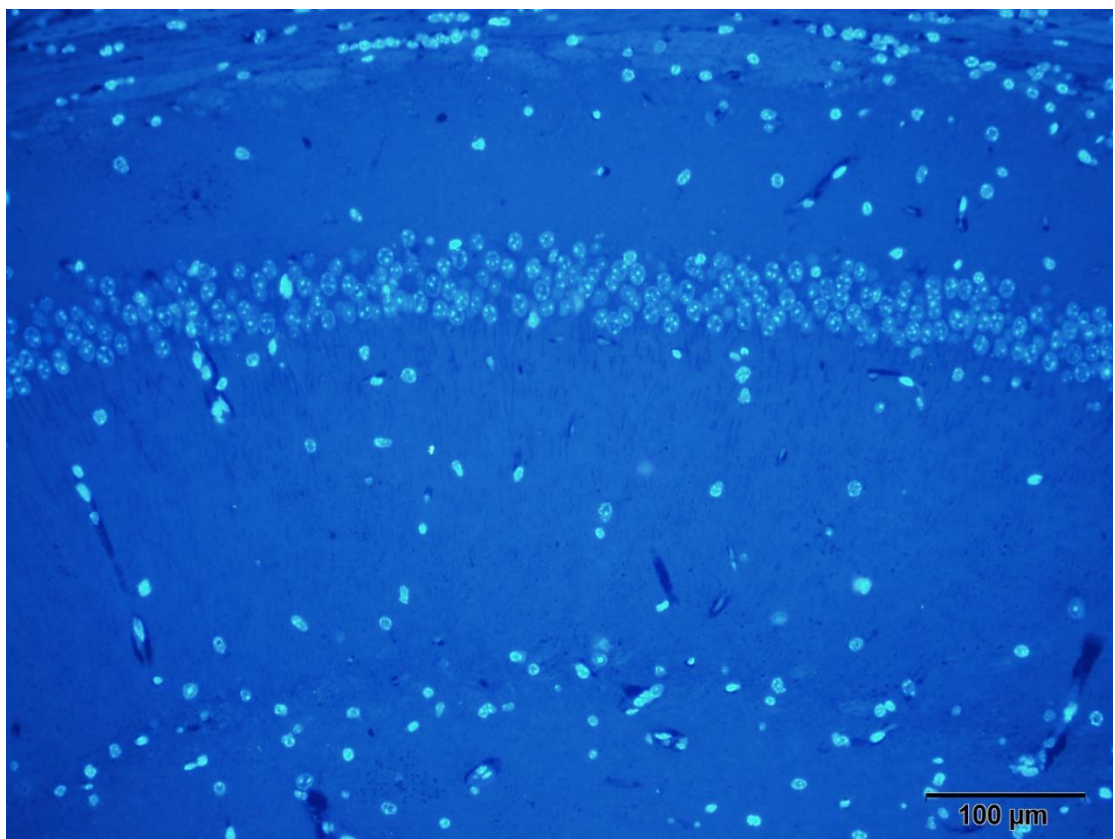

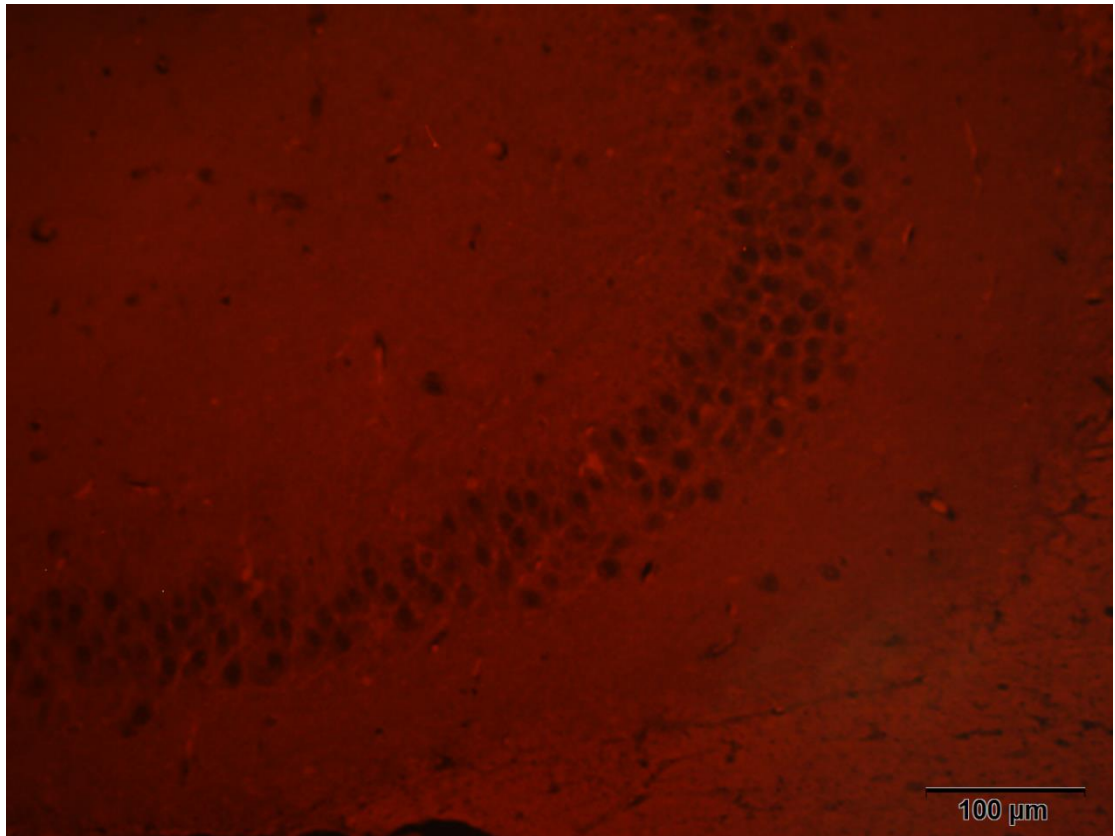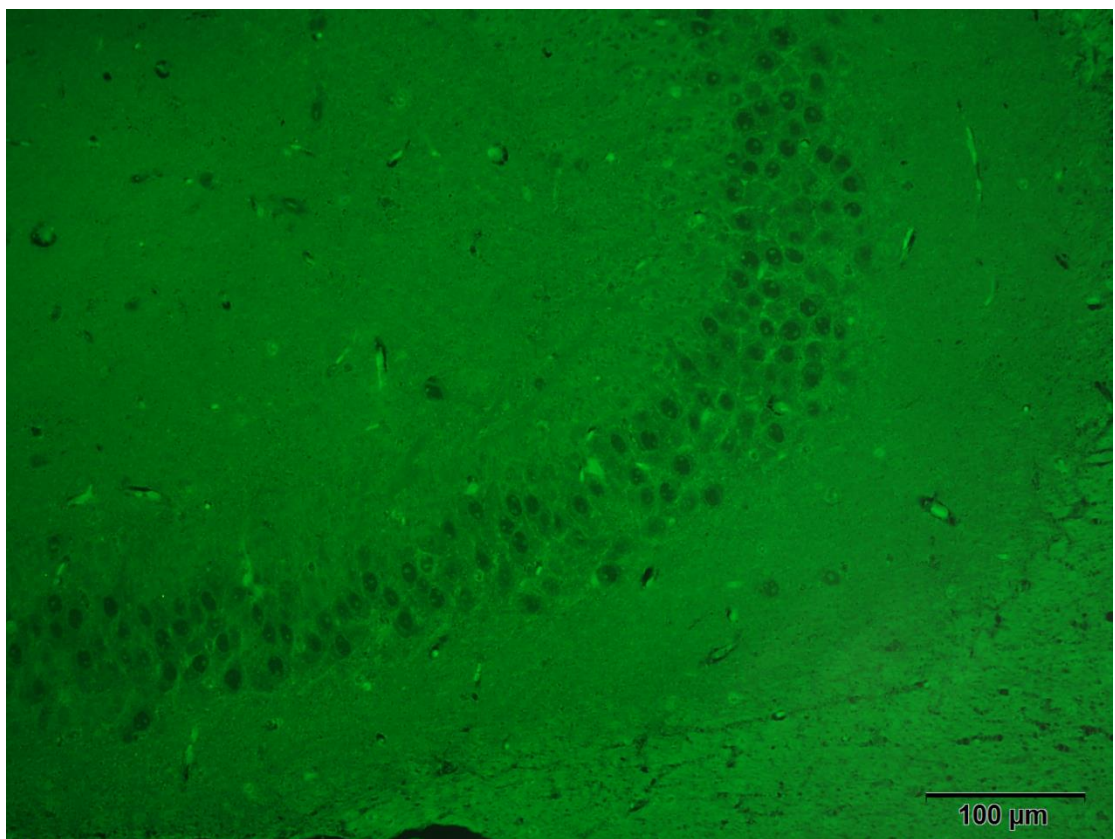

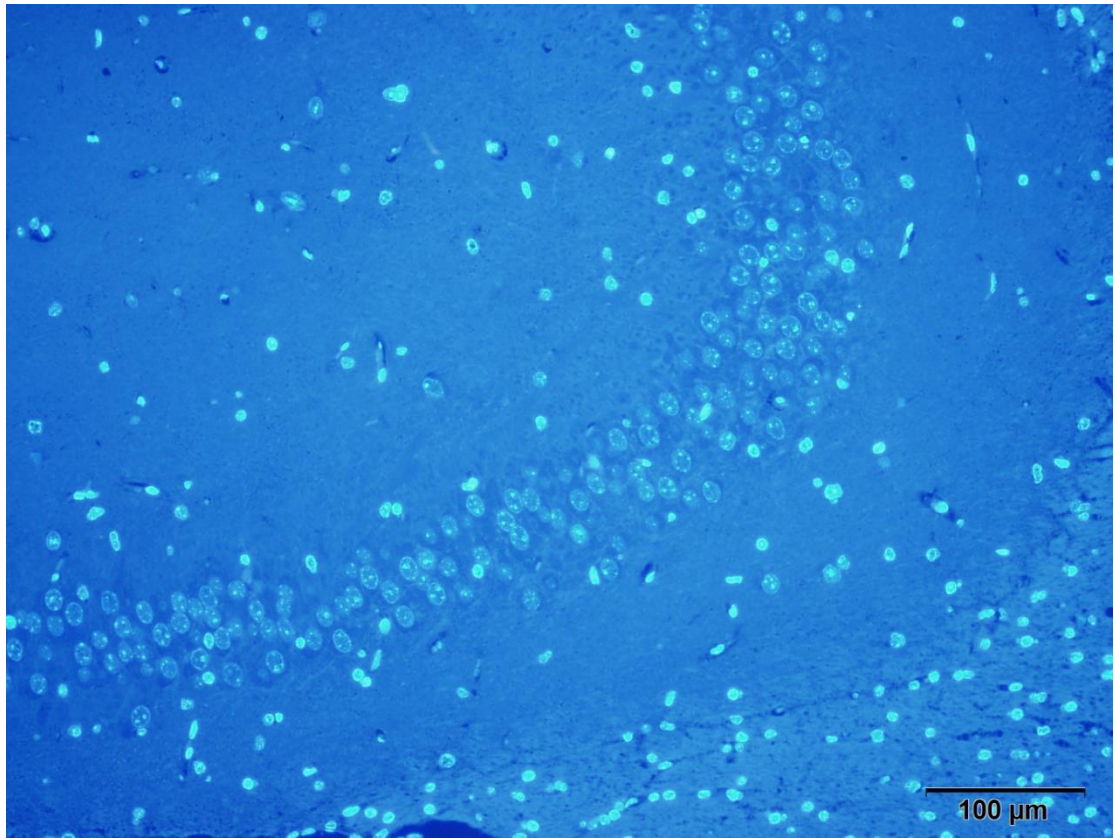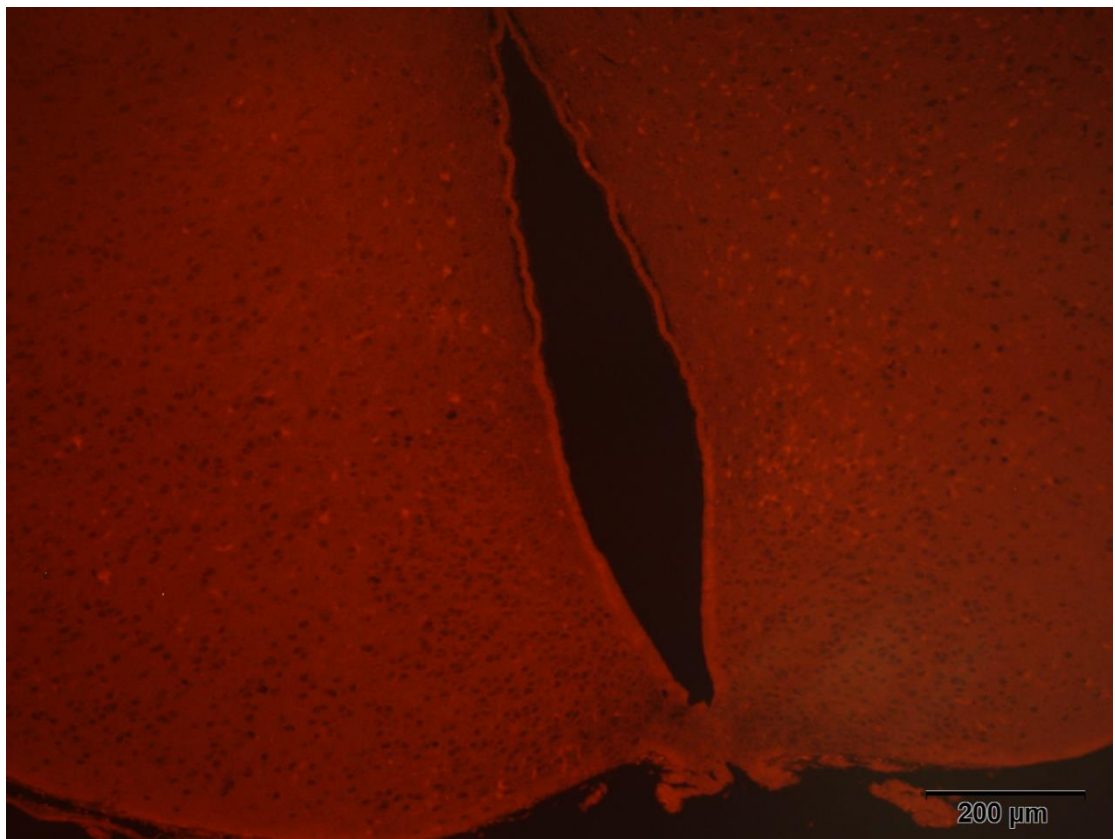

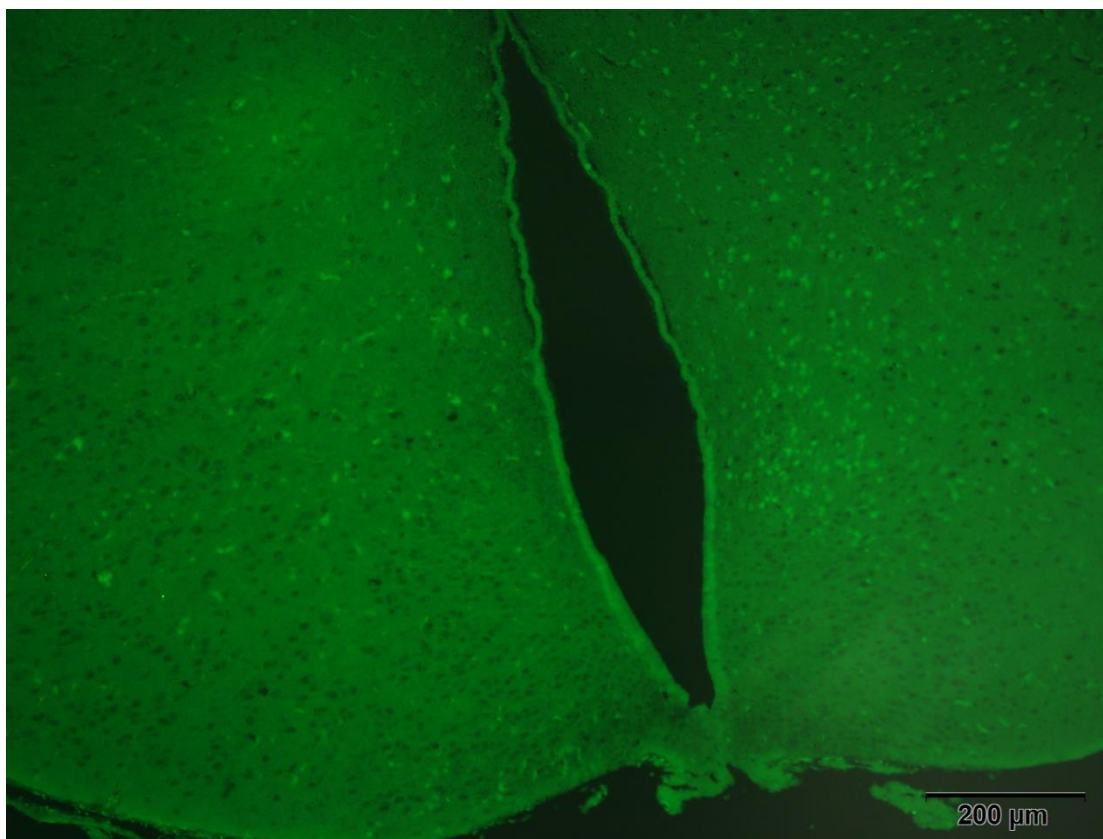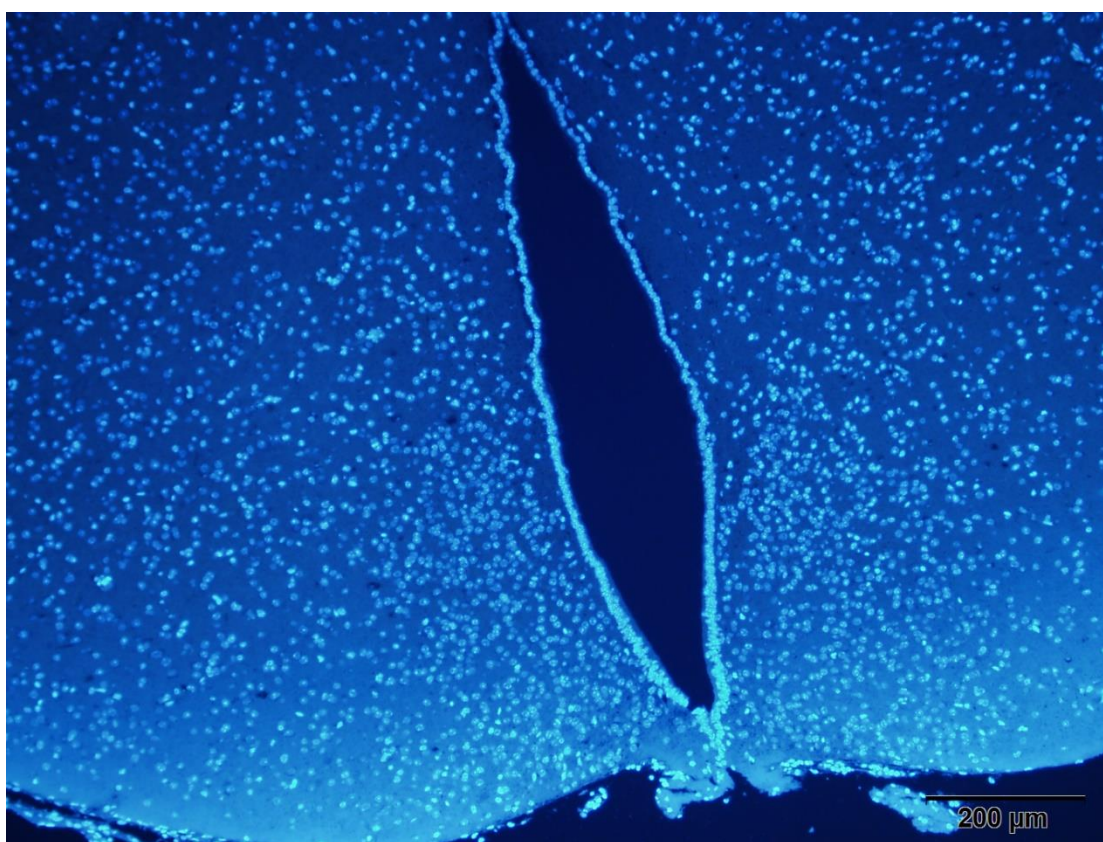

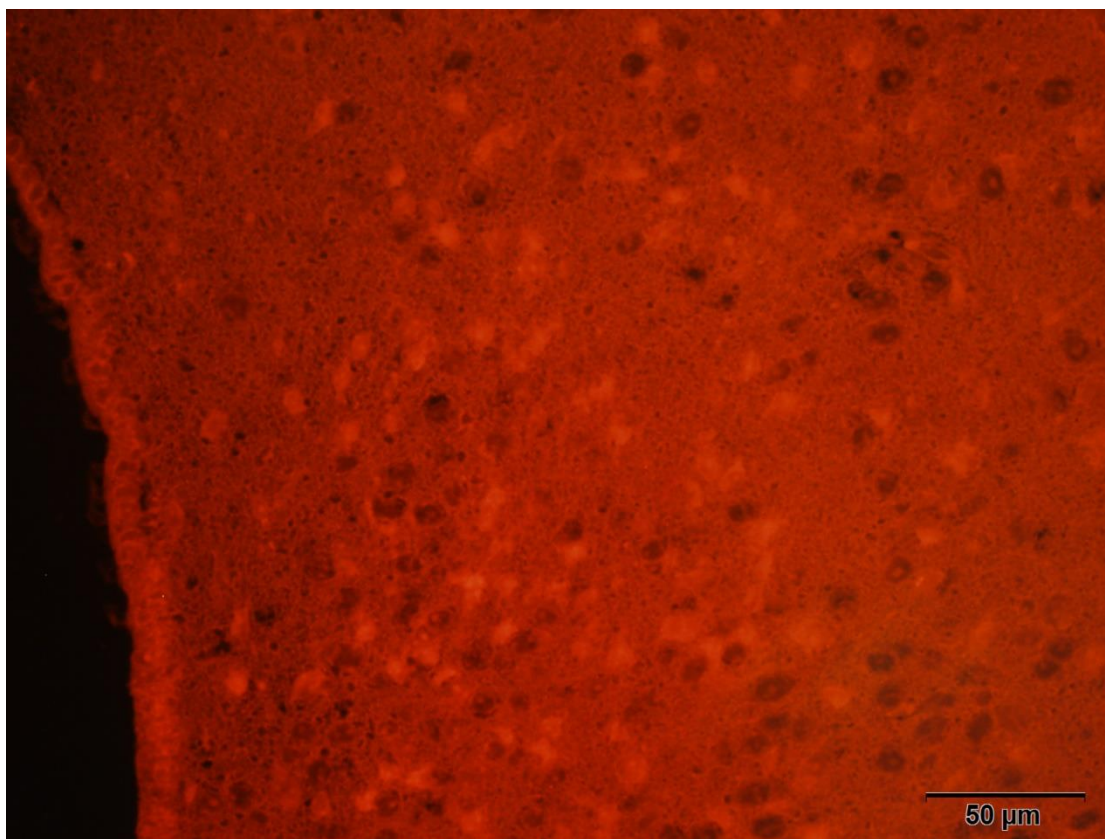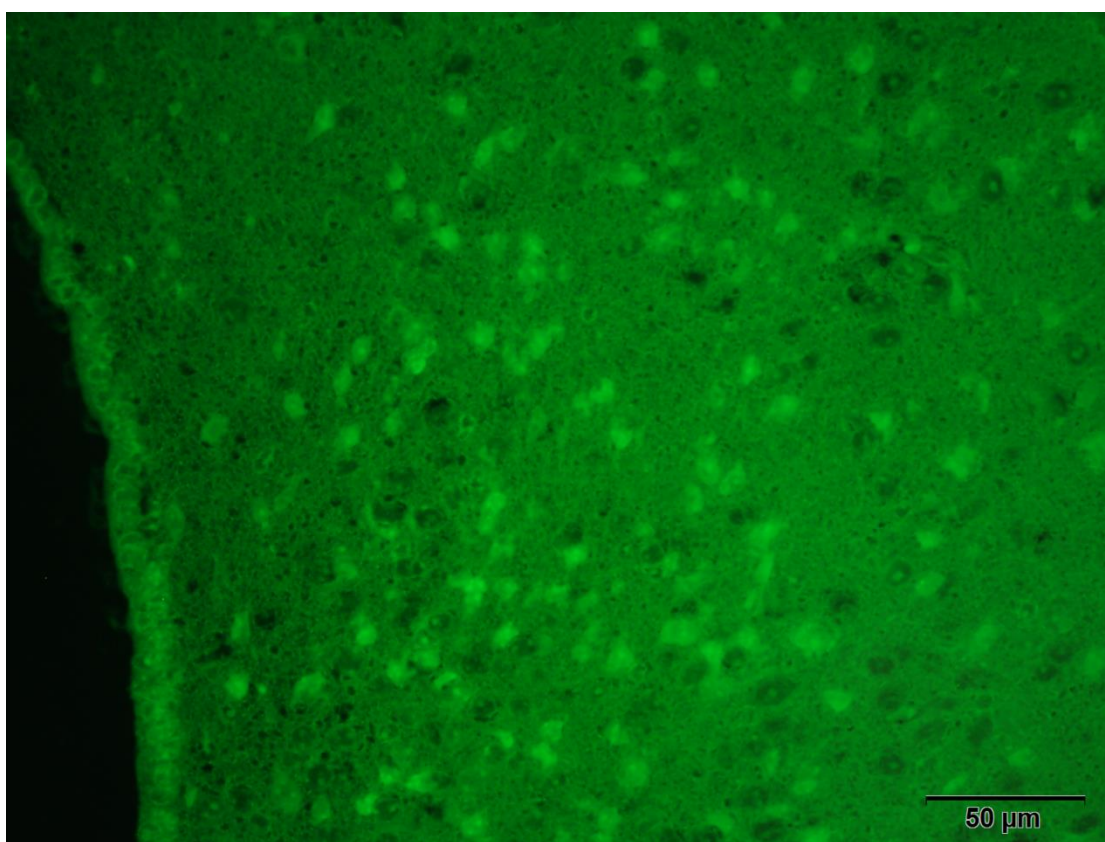

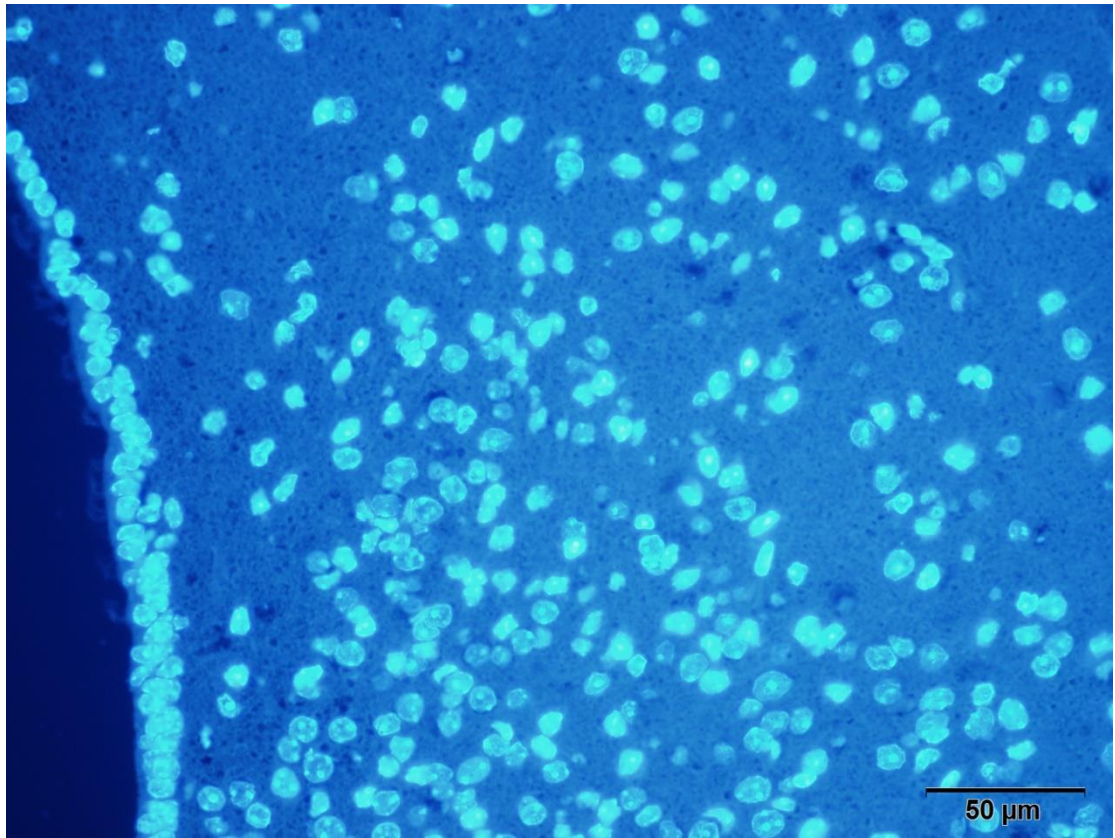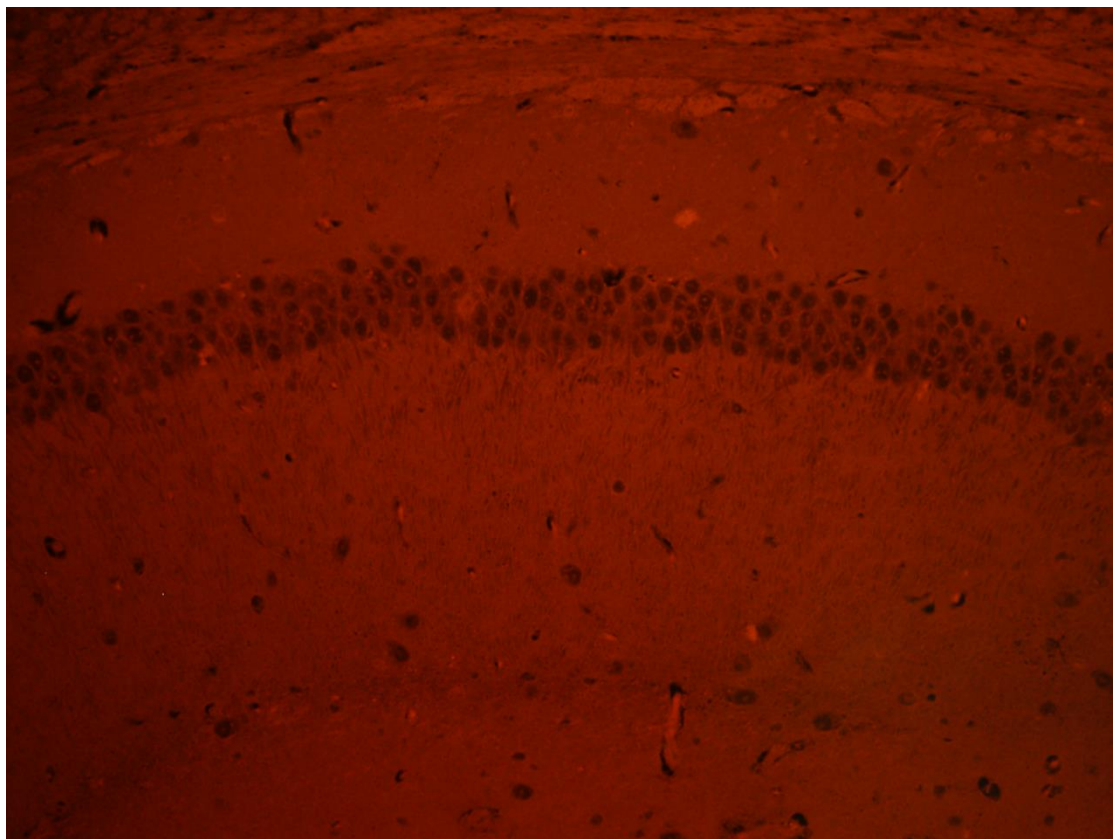

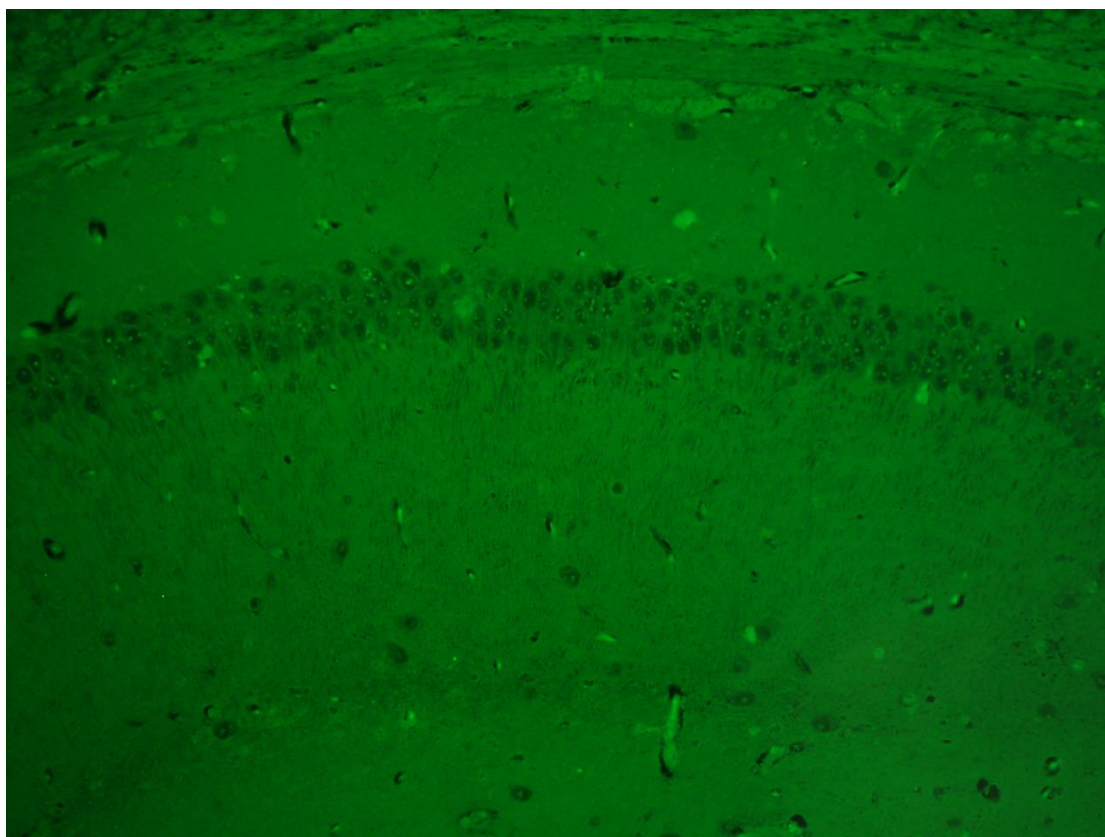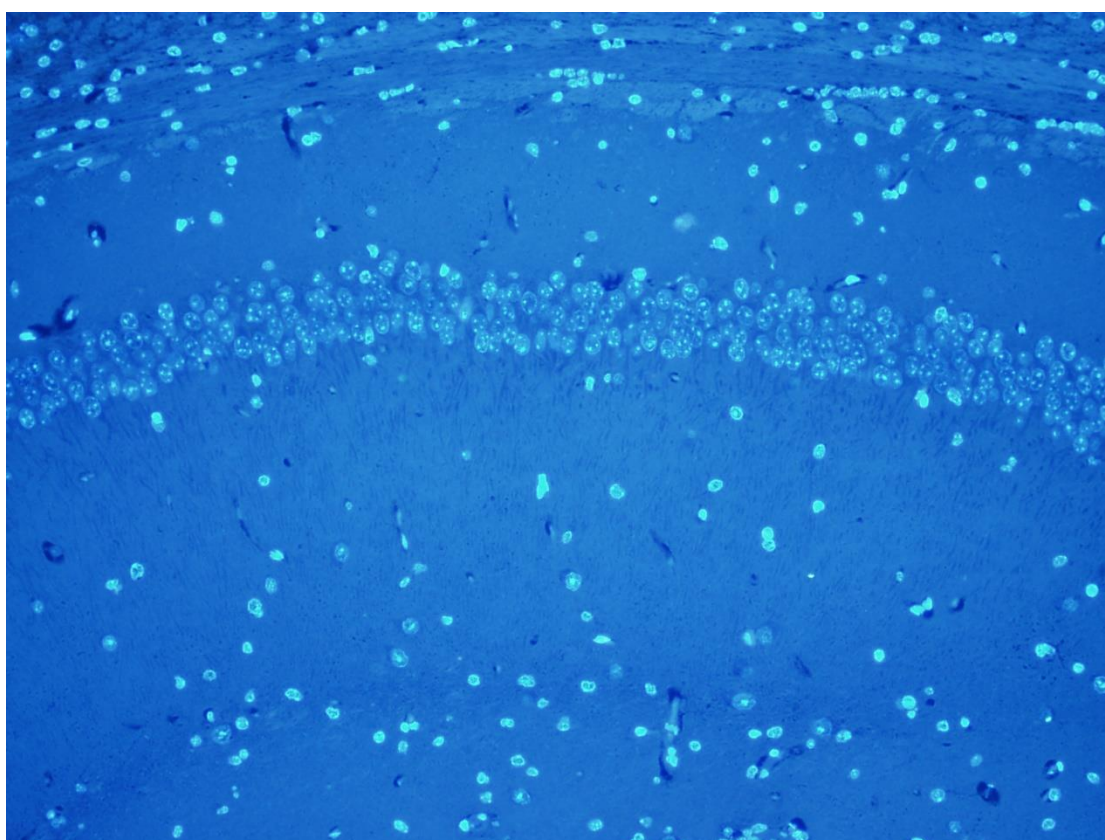

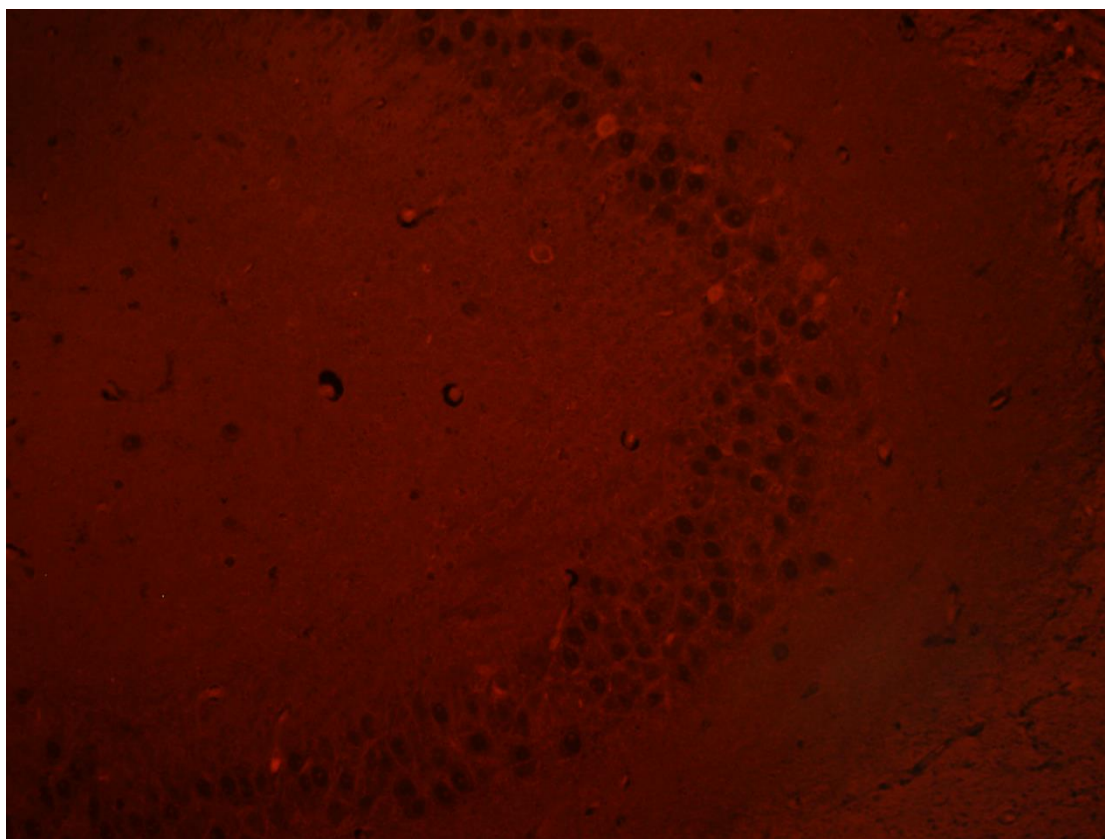

Supplement: Supplementary file 2 [file DataSheet4.PDF]

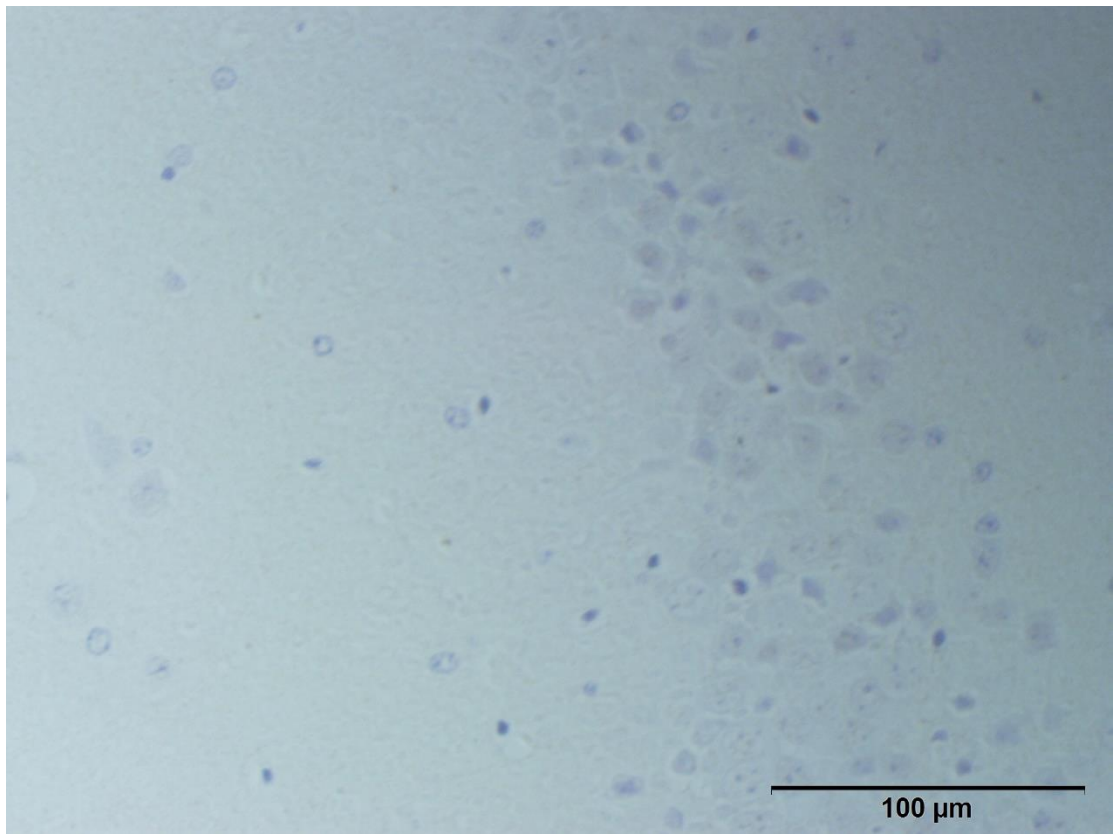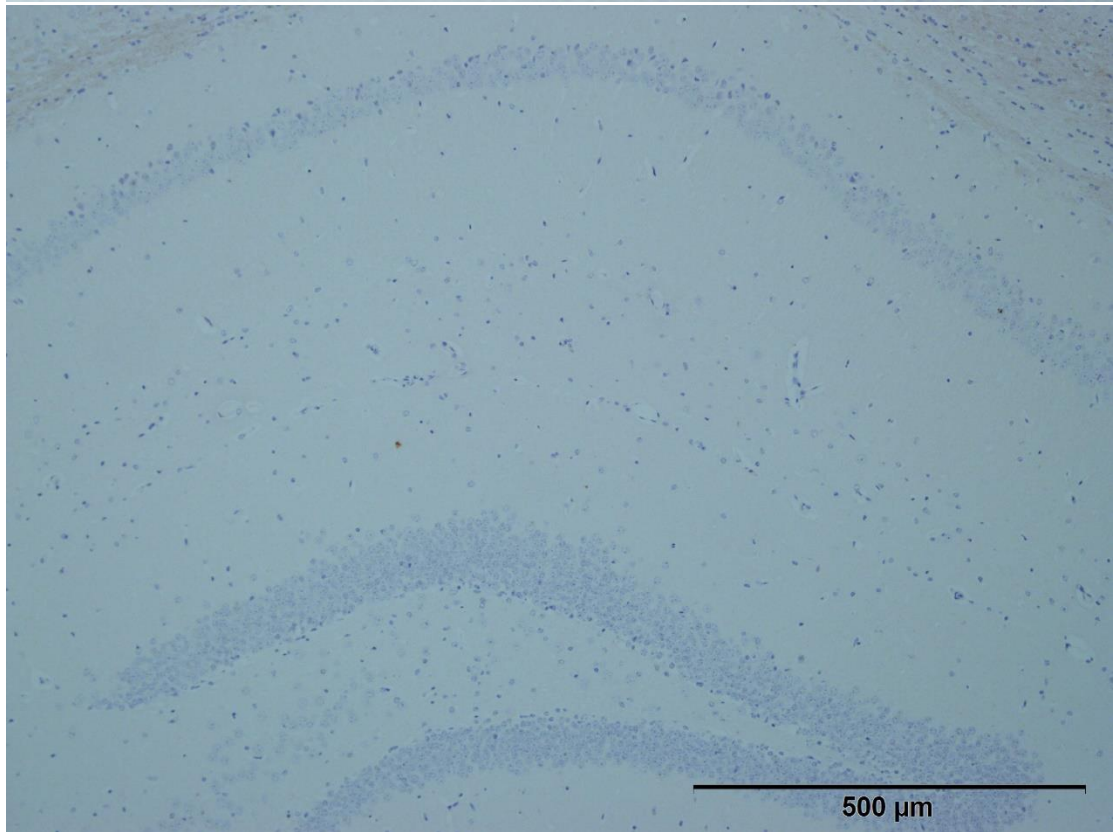

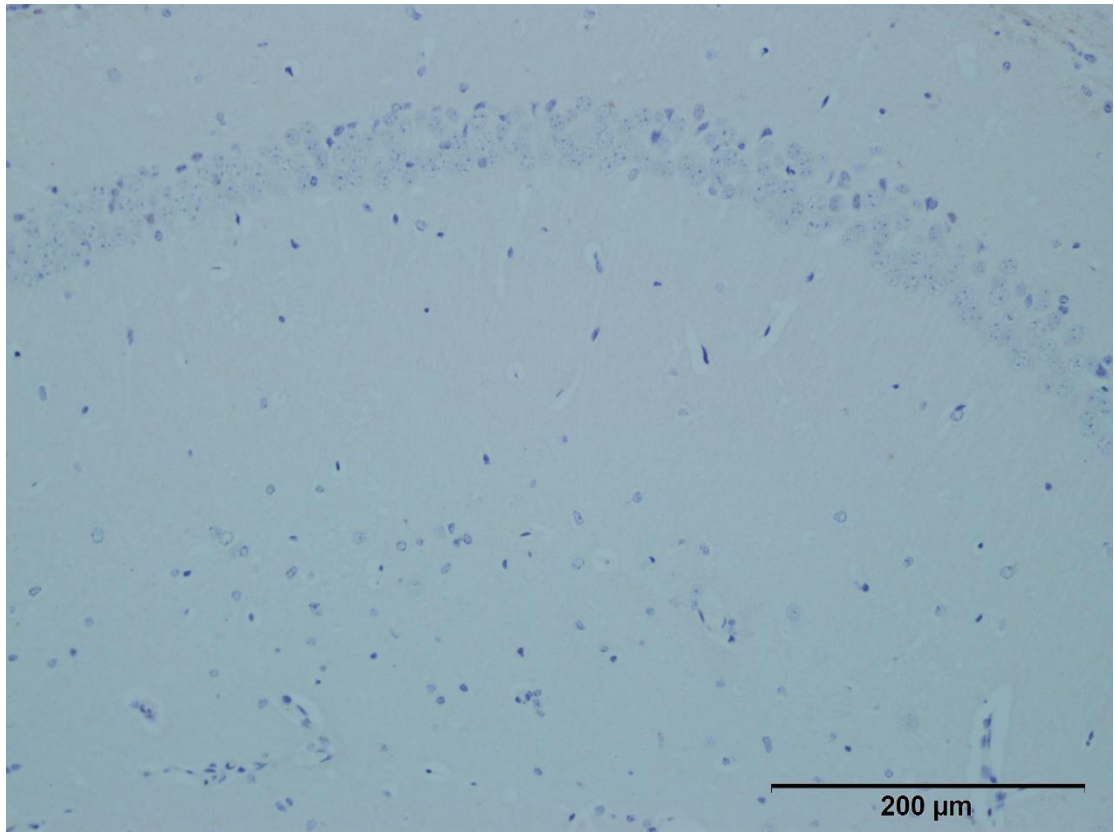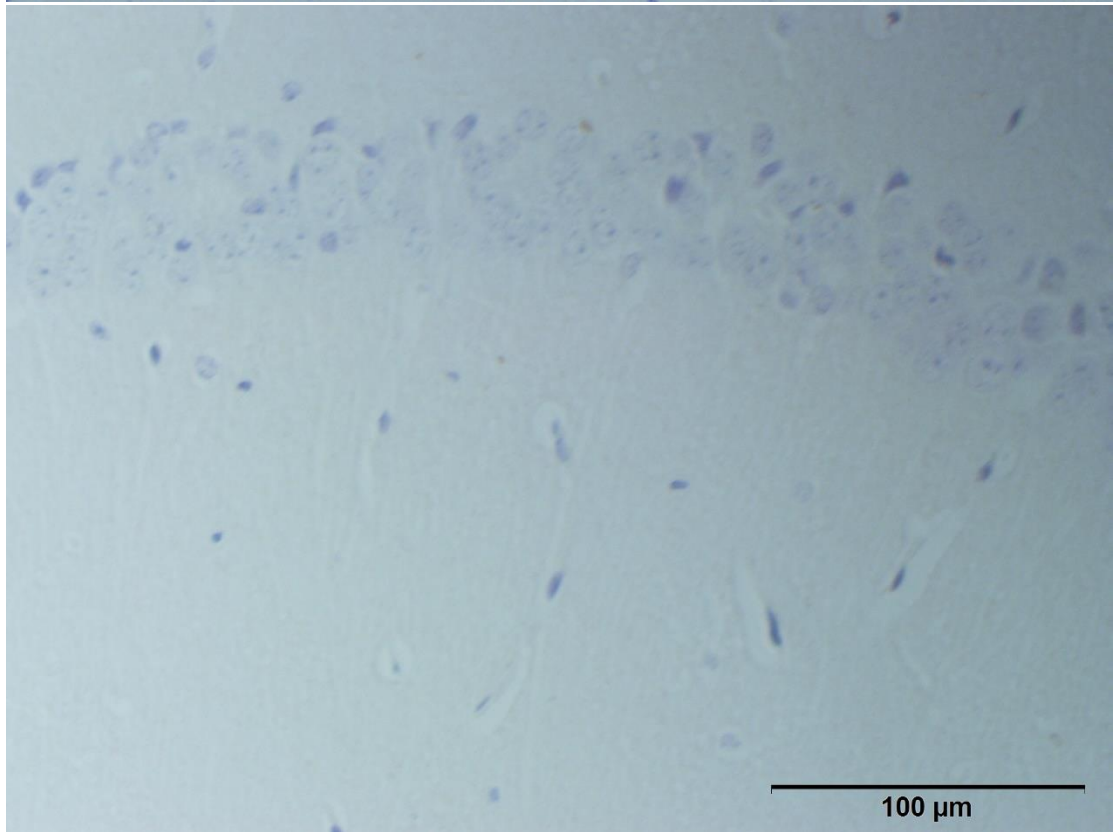

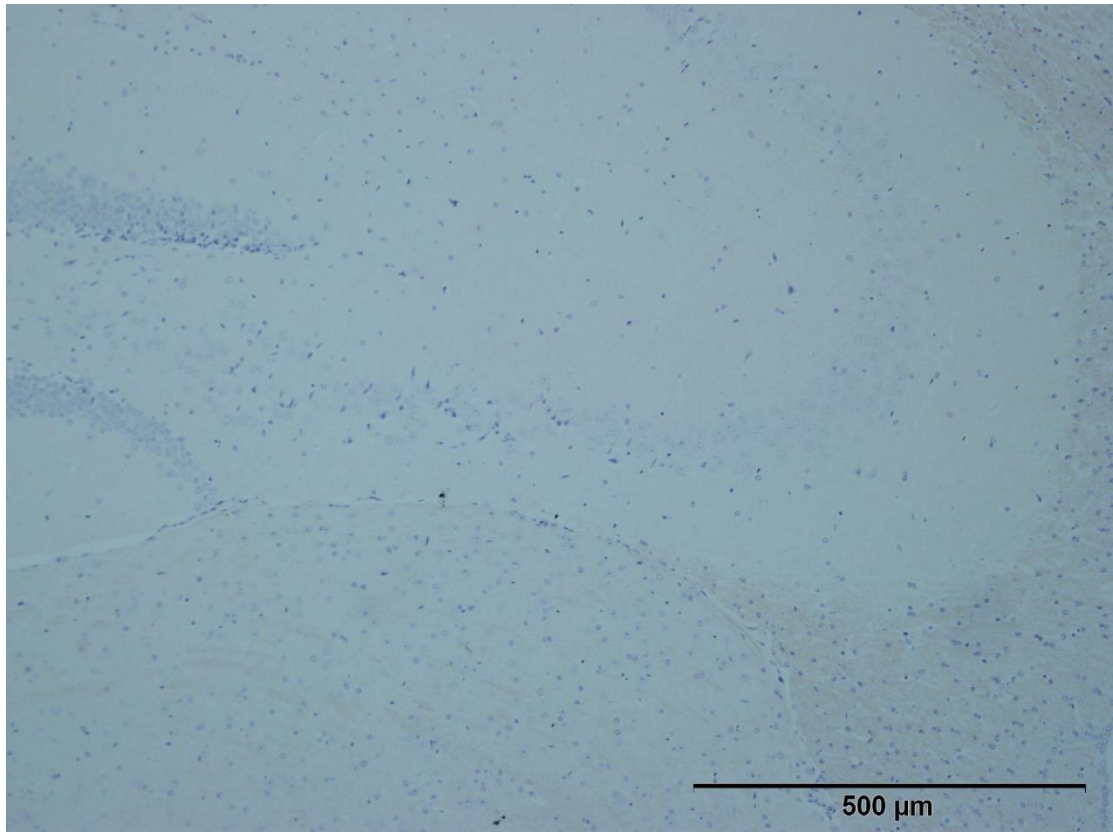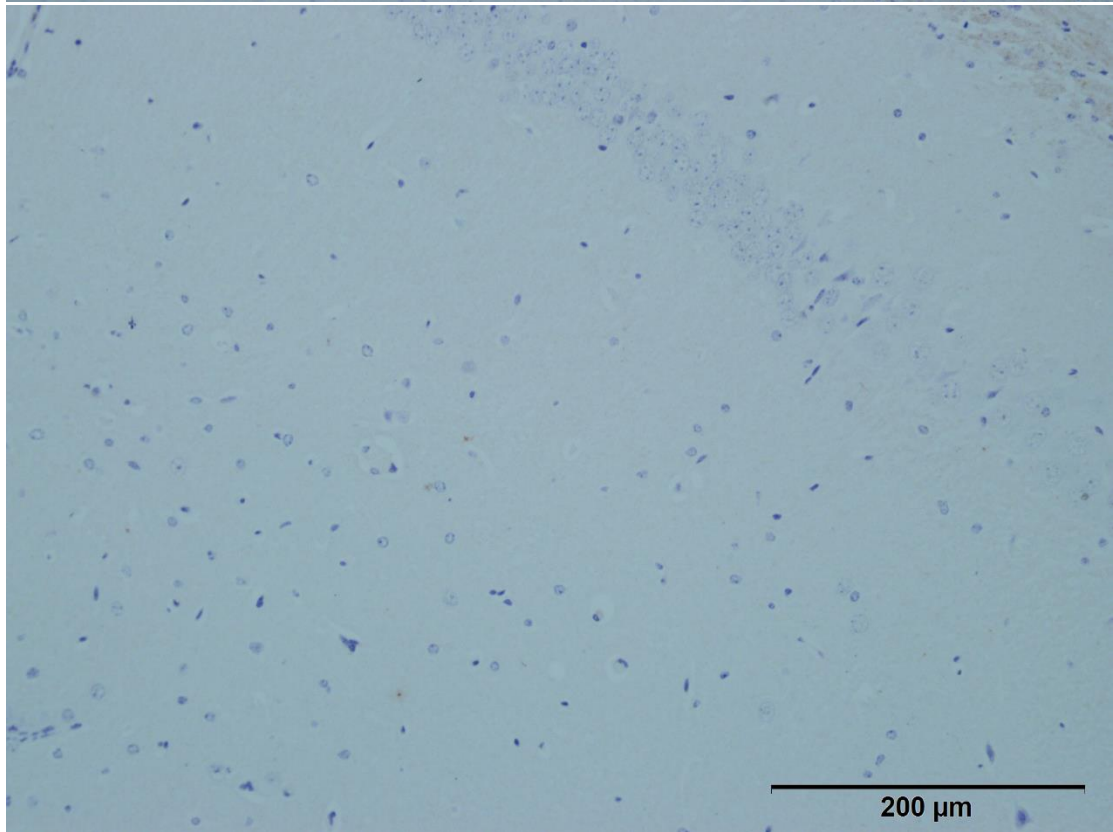

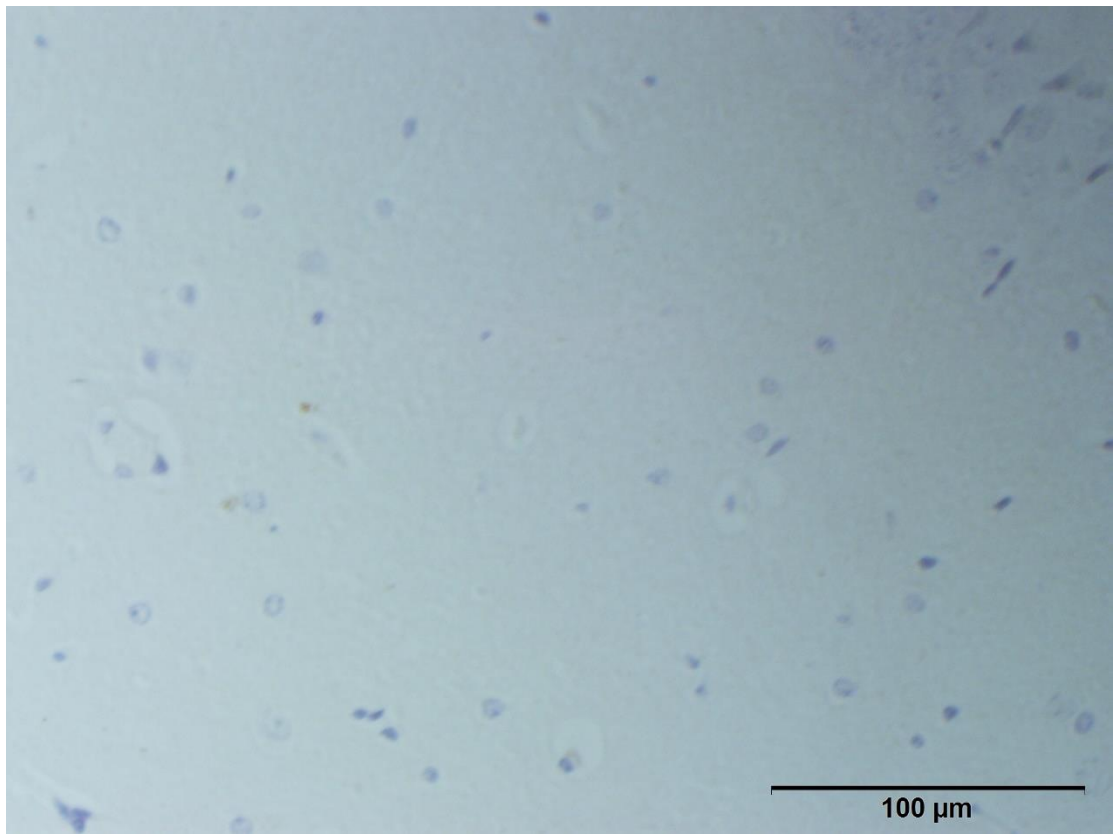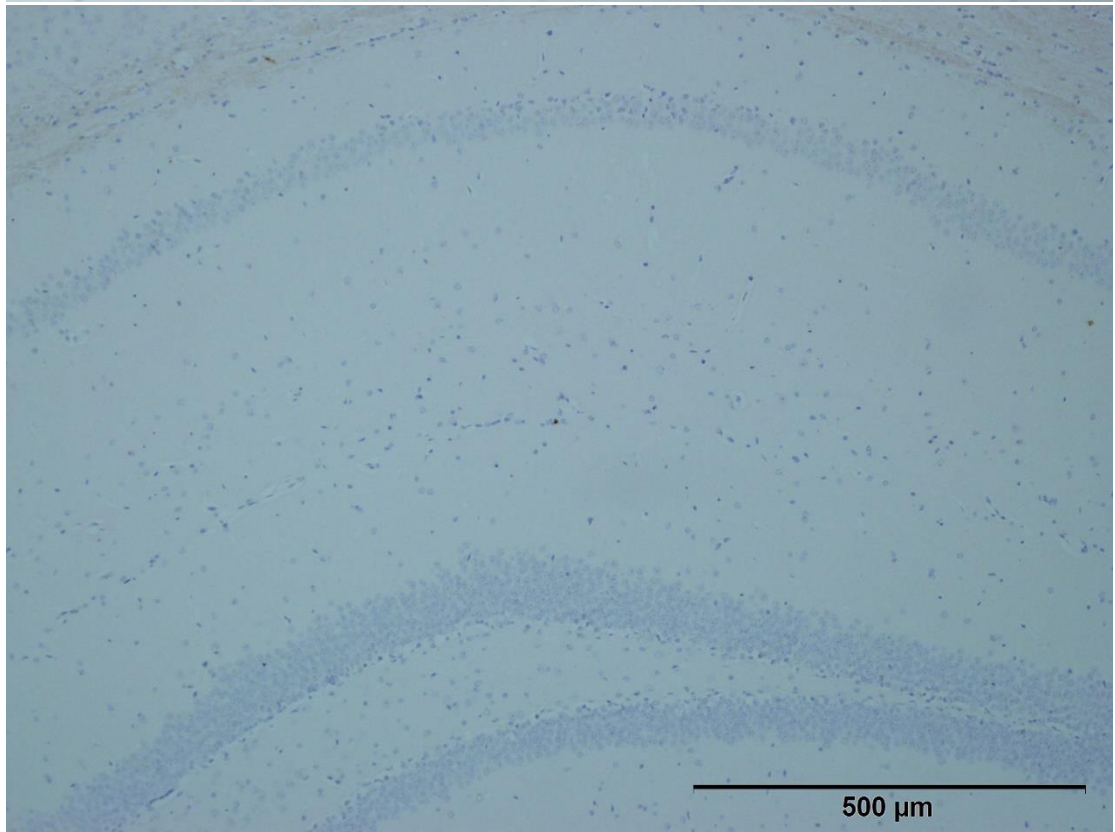

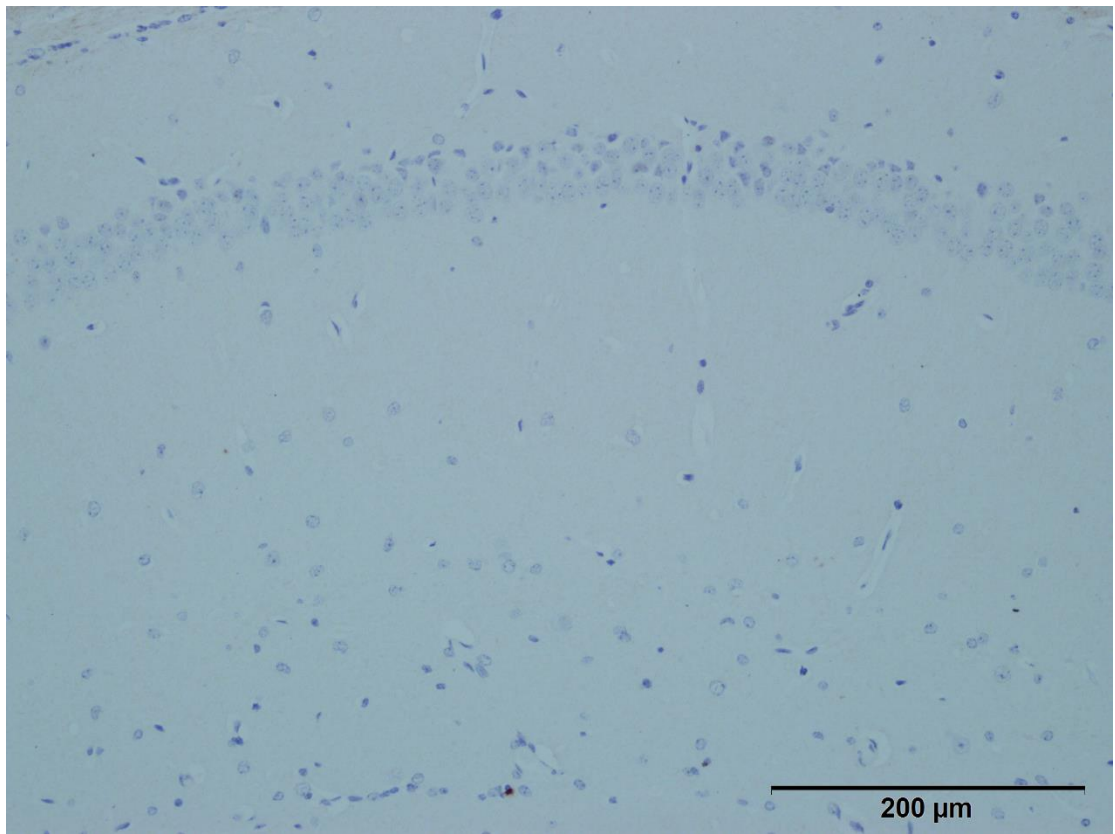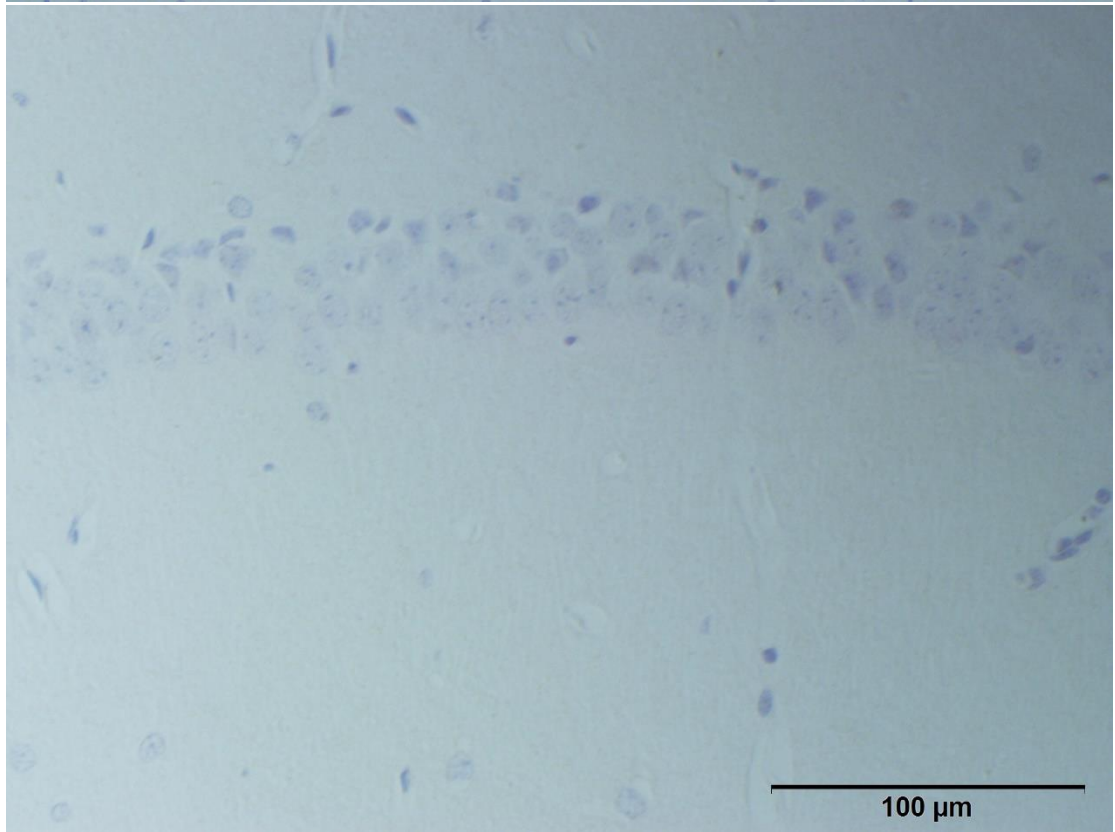

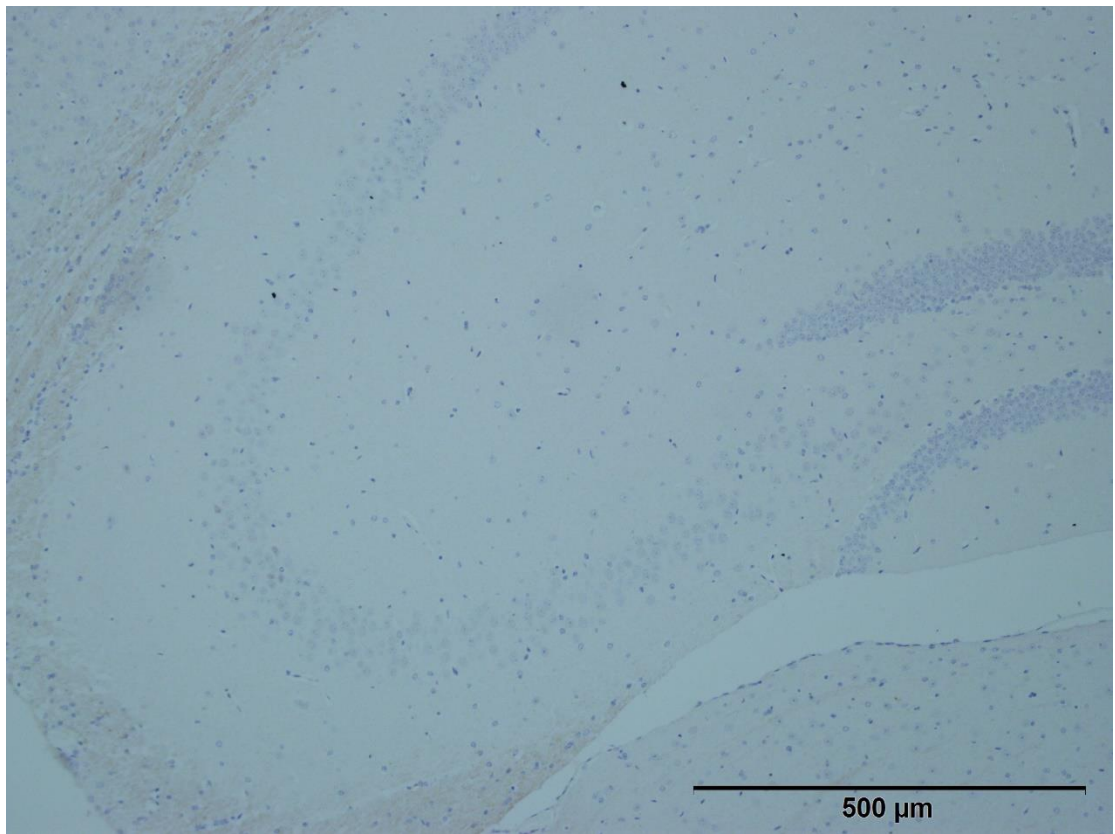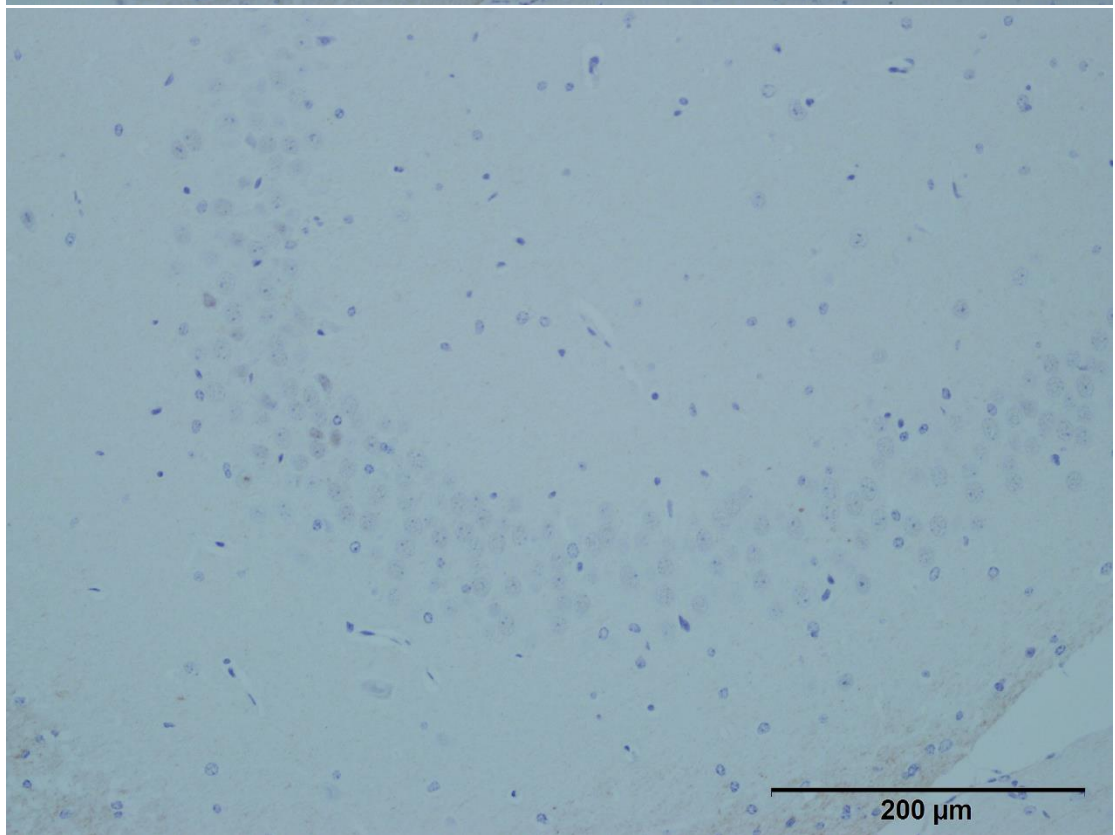

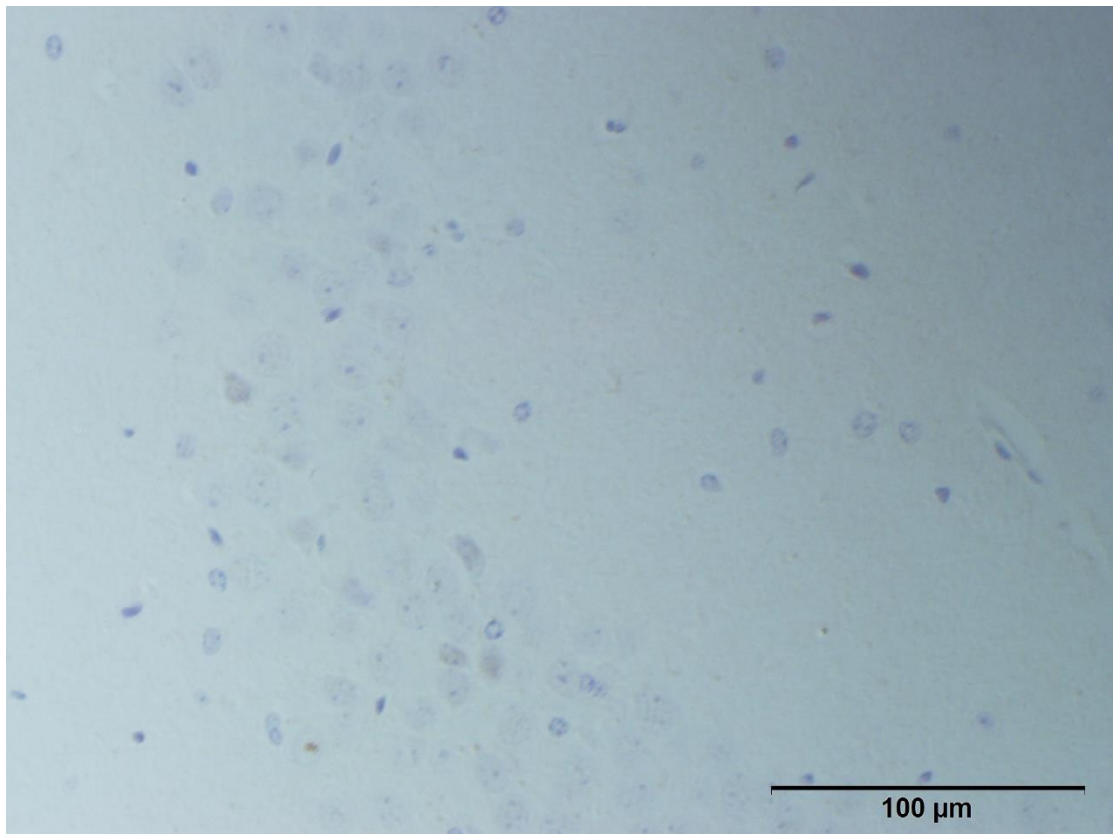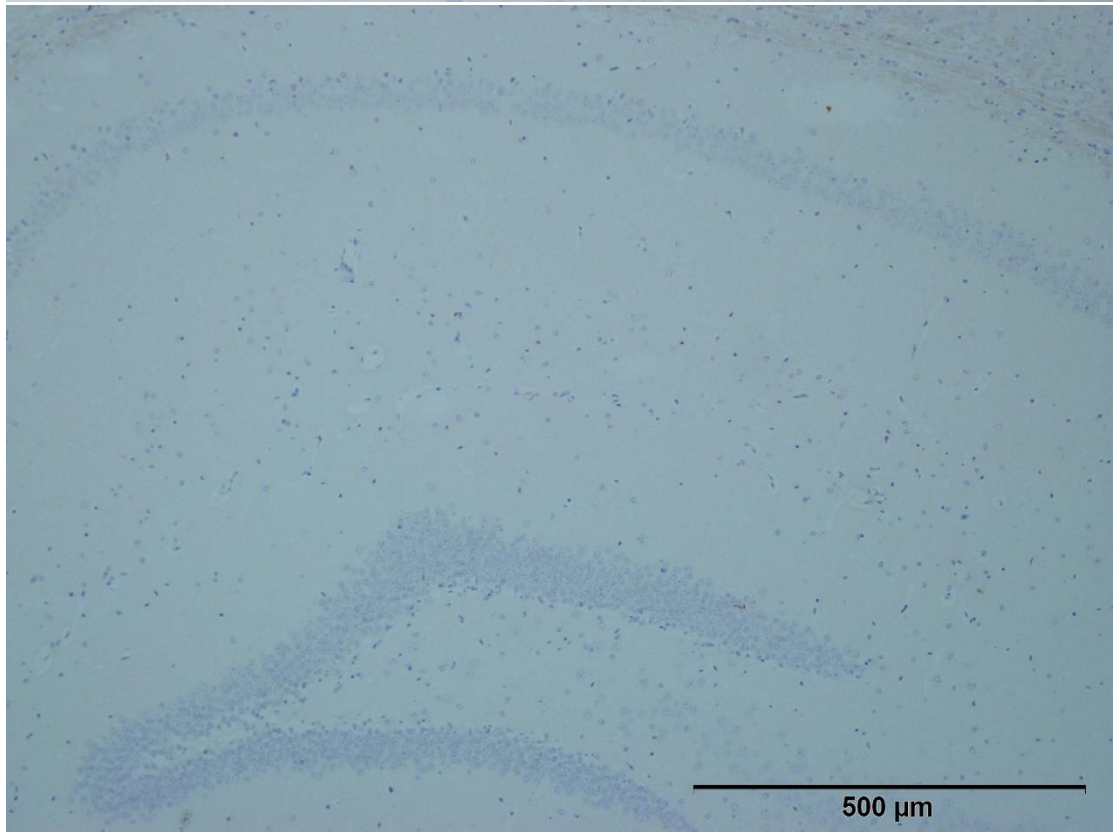

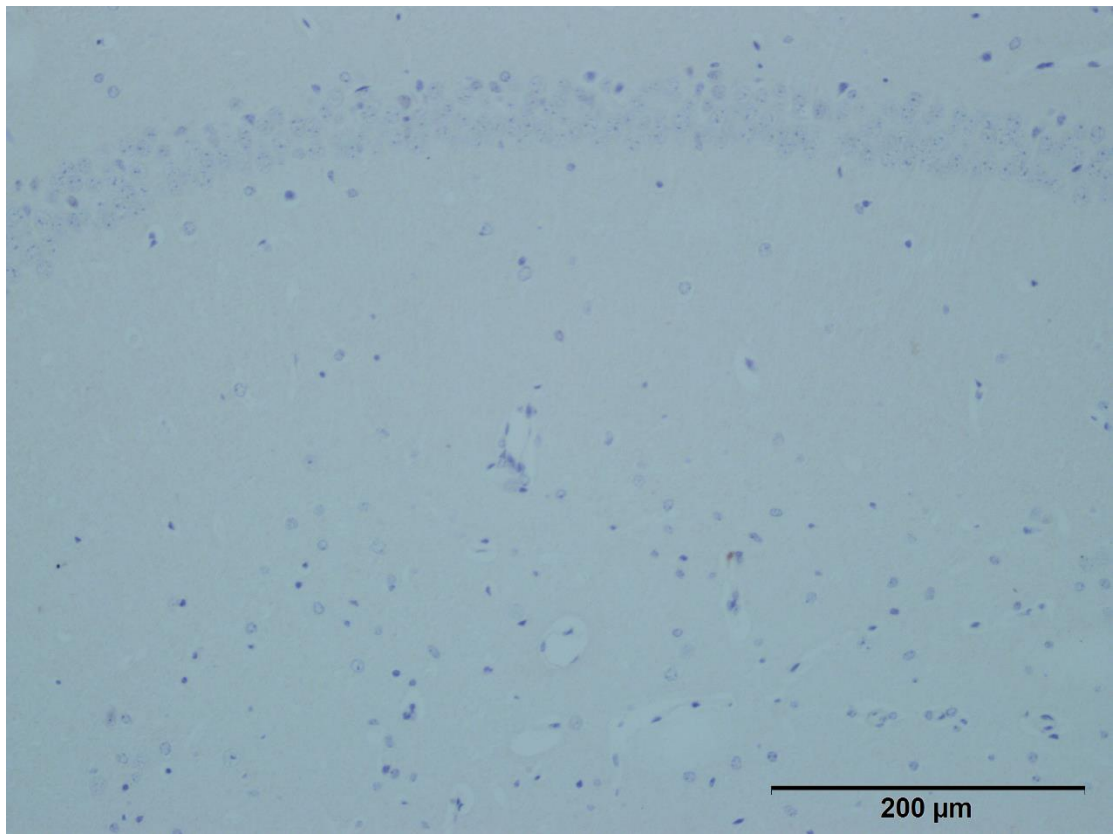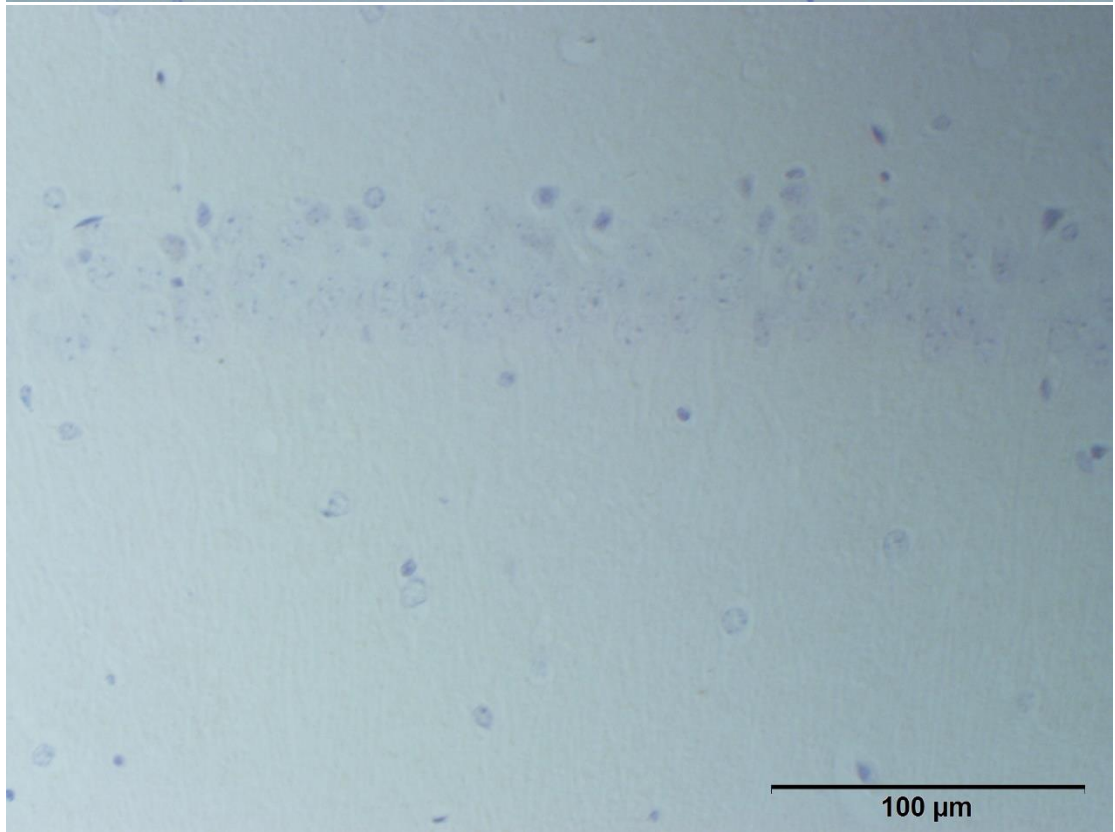

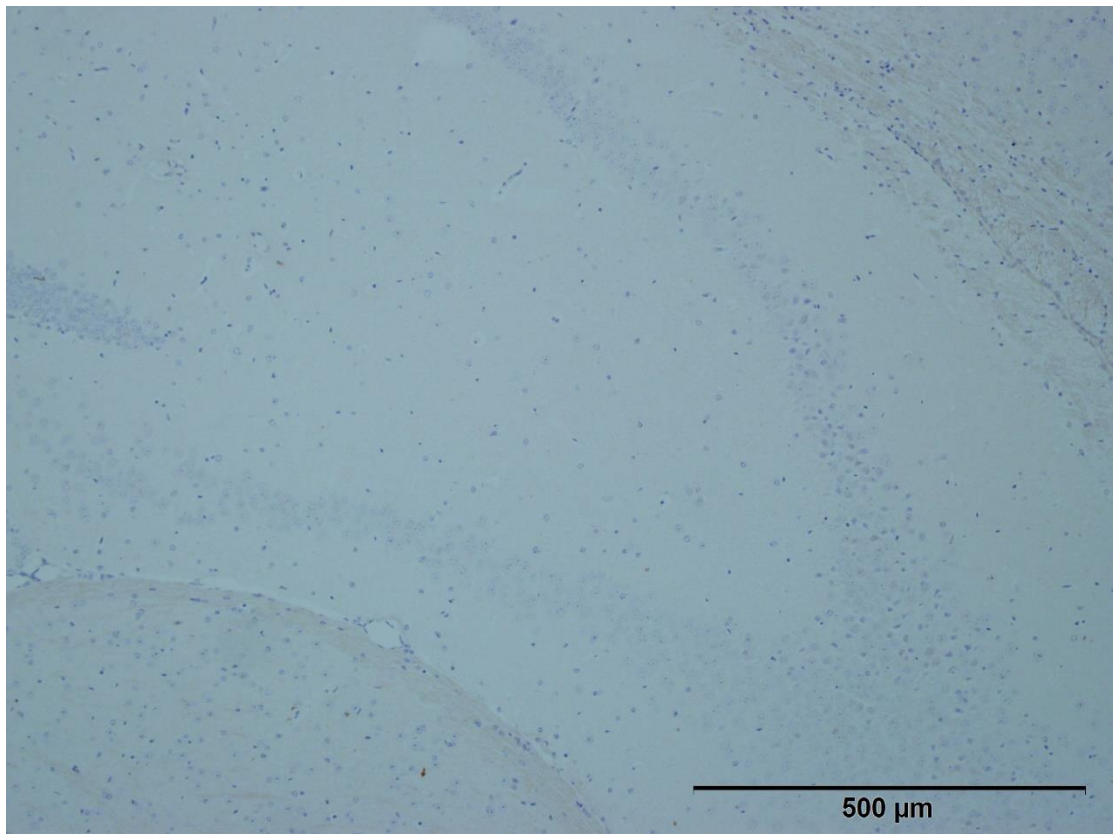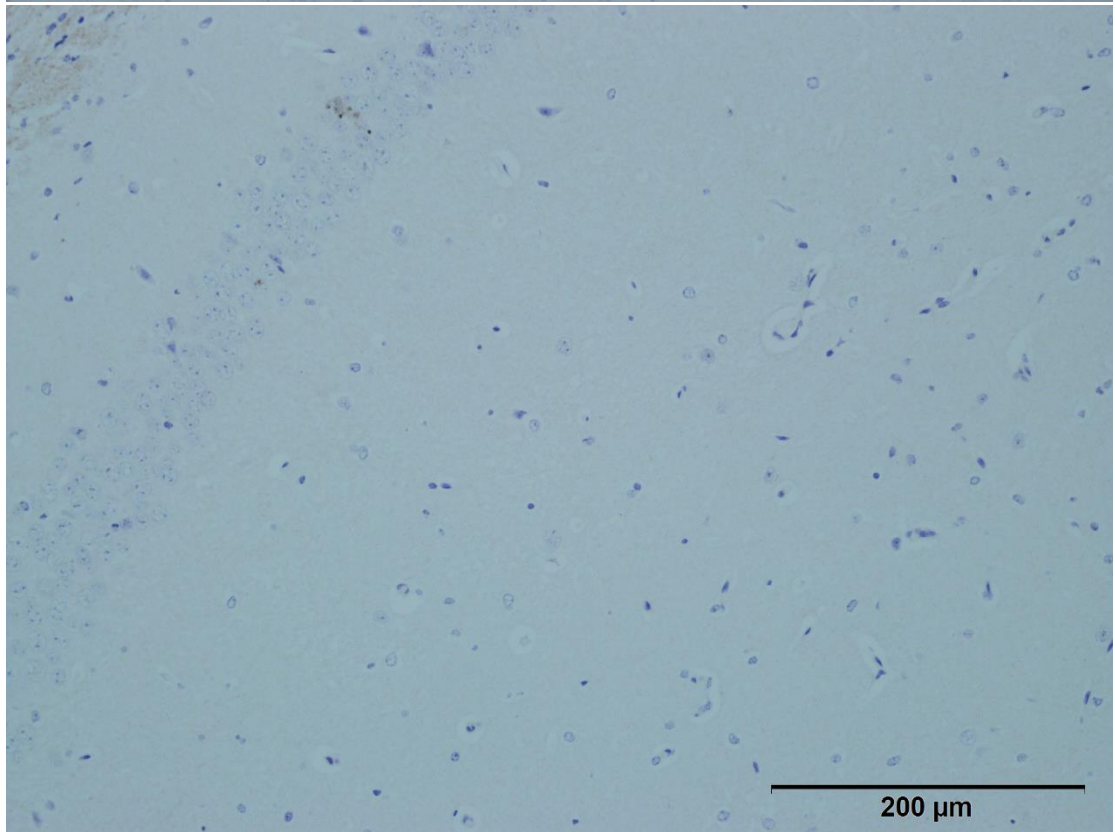

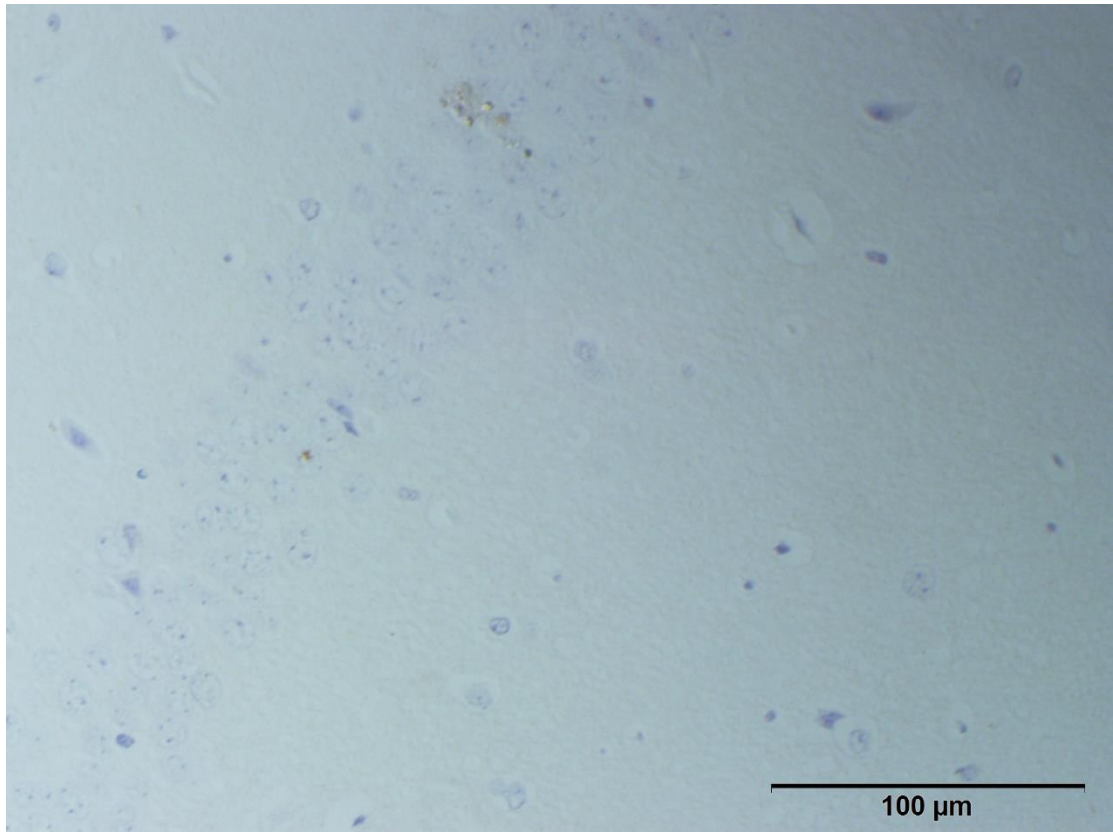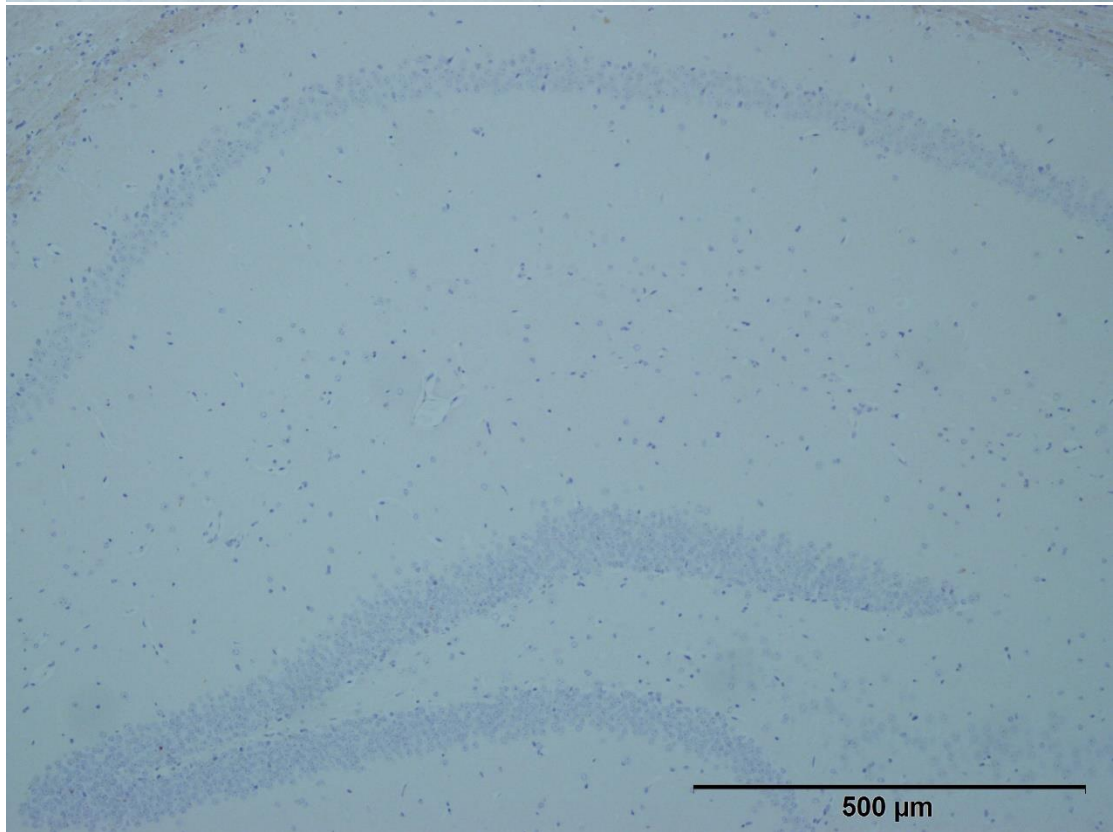

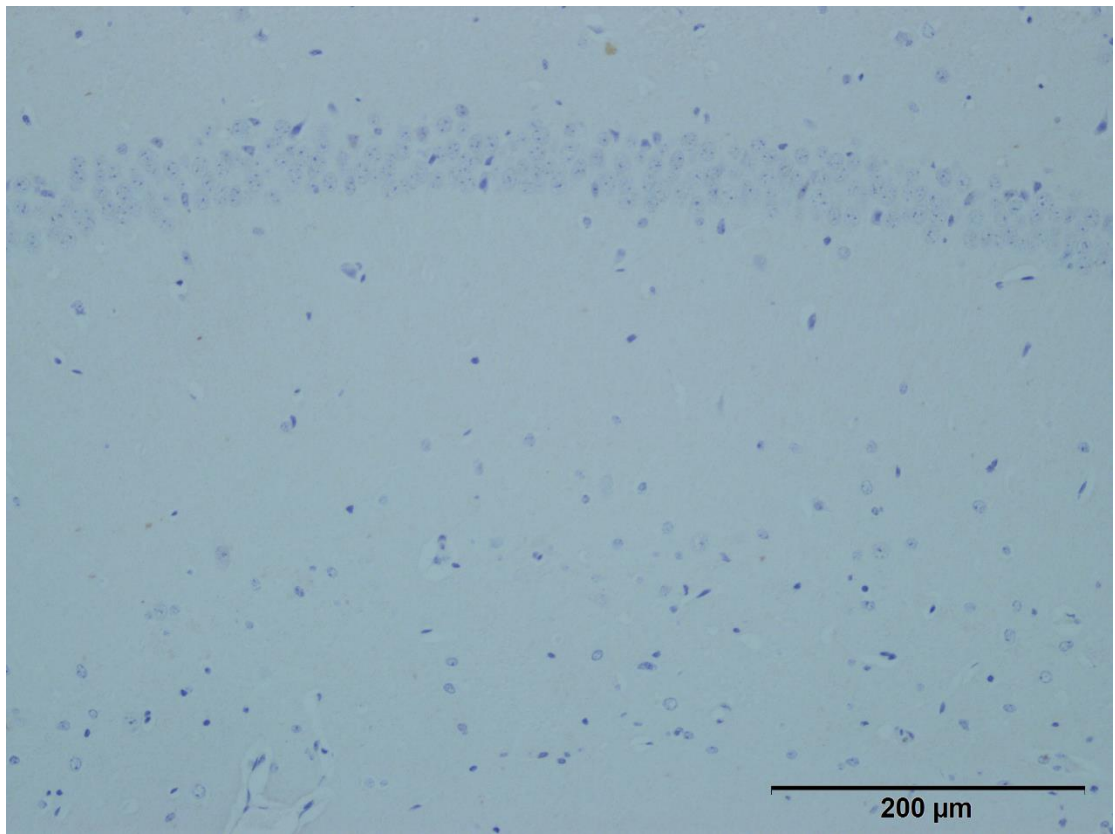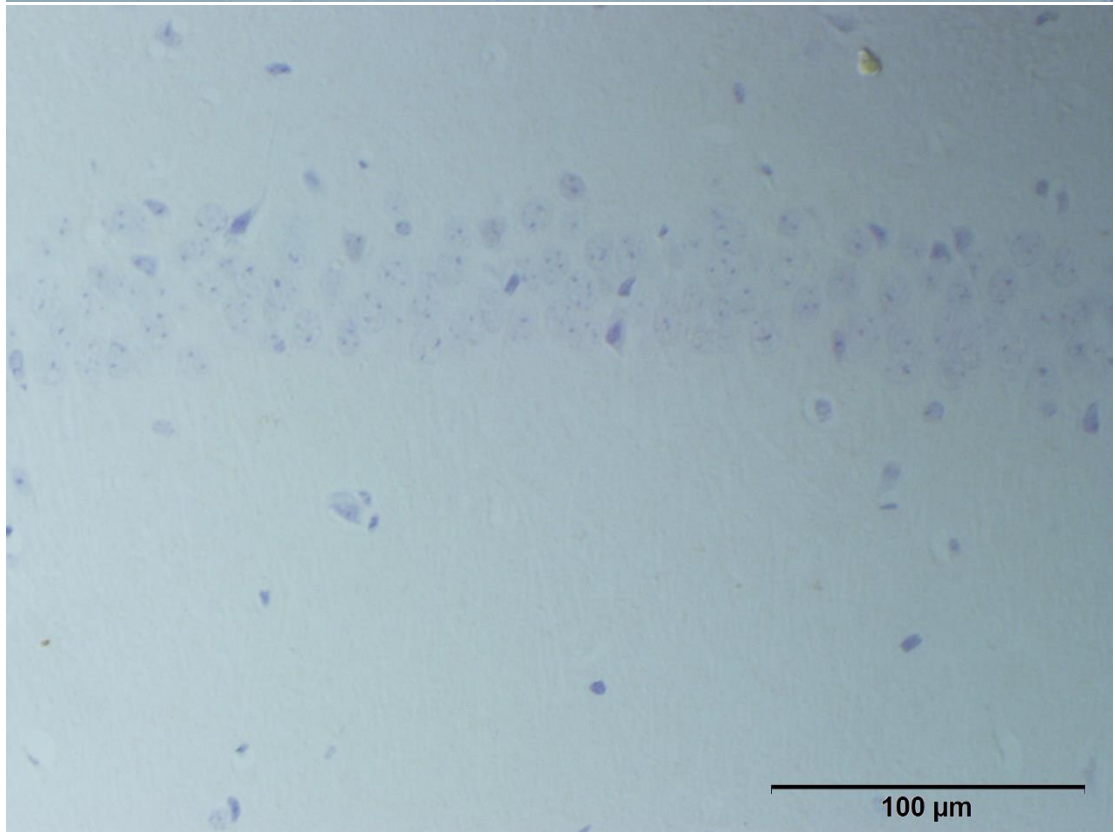

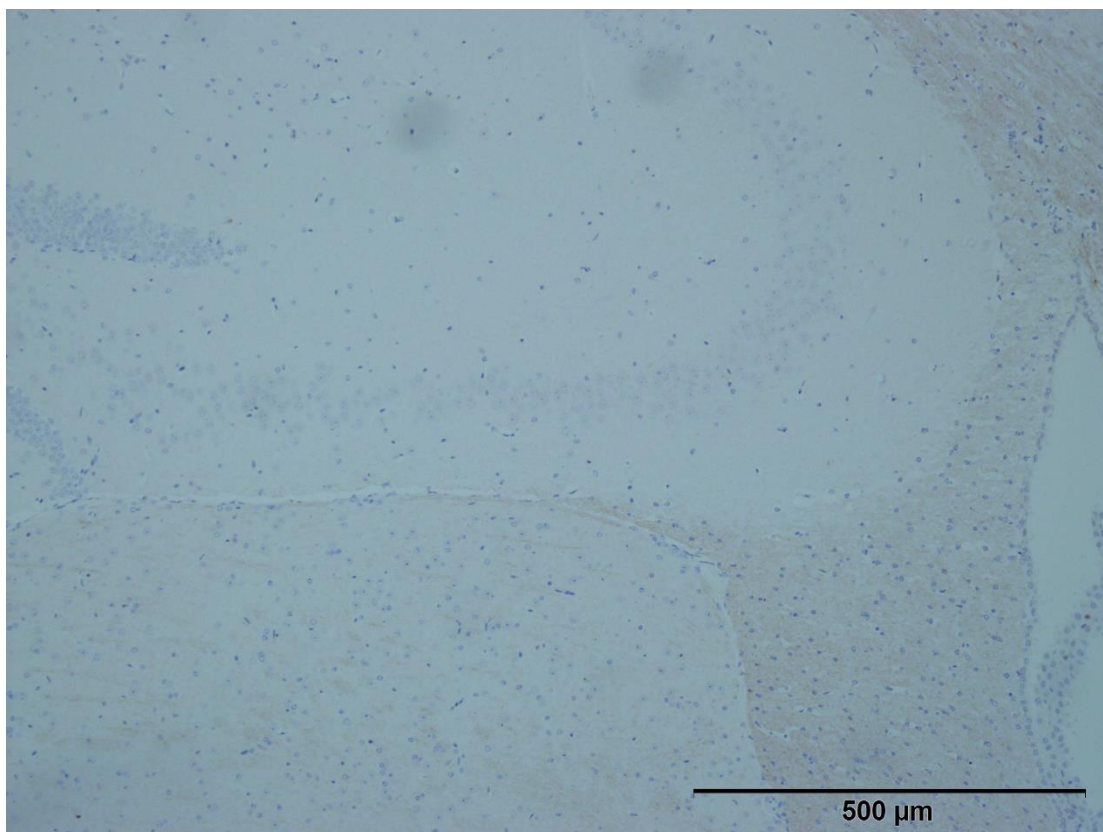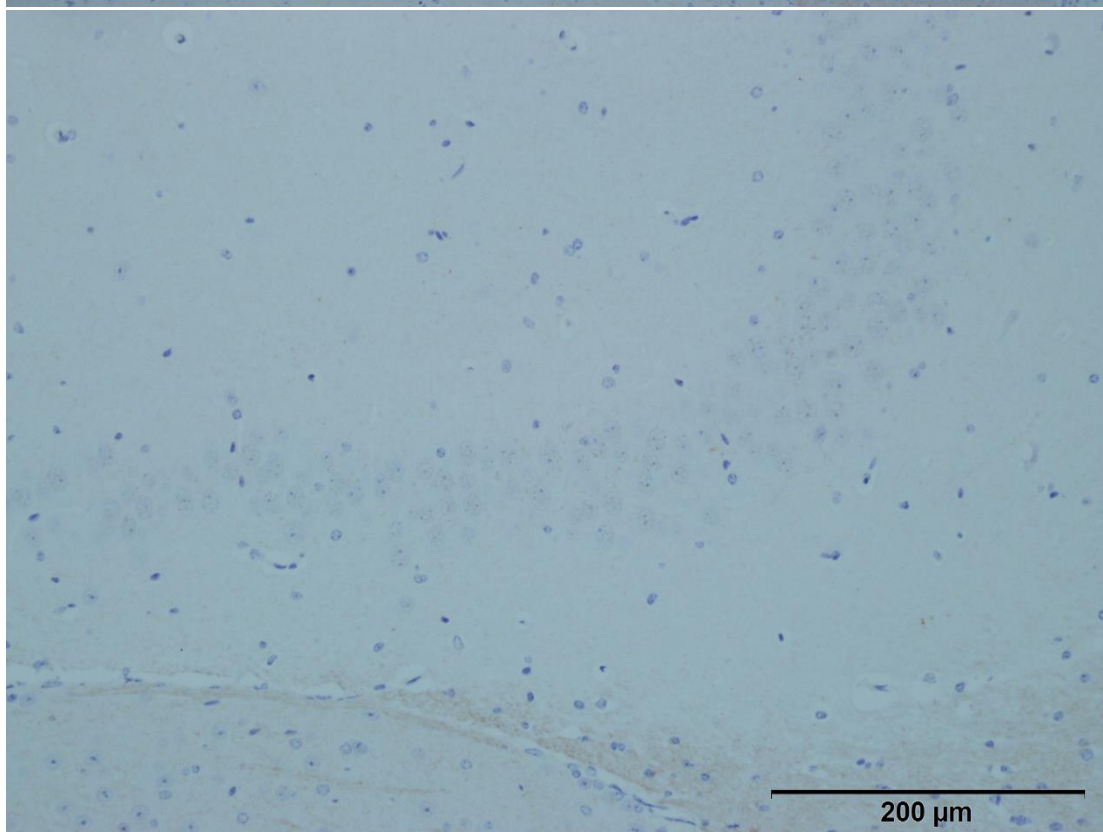

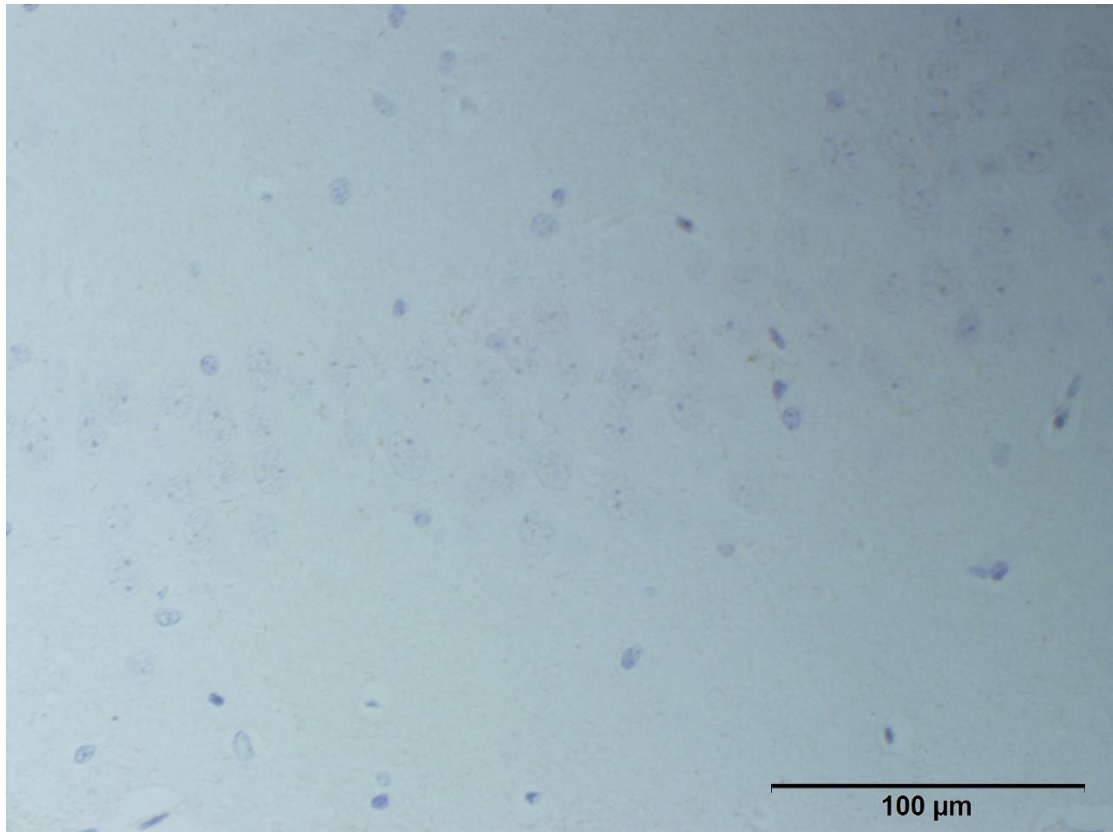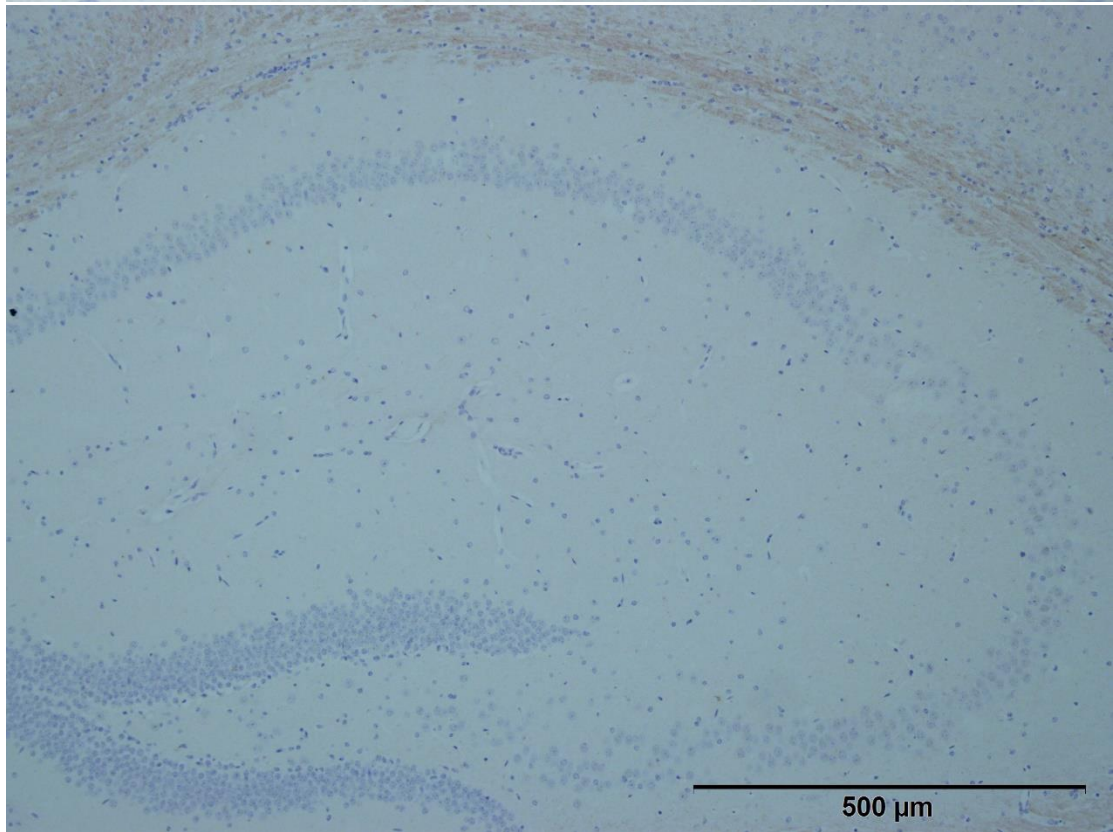

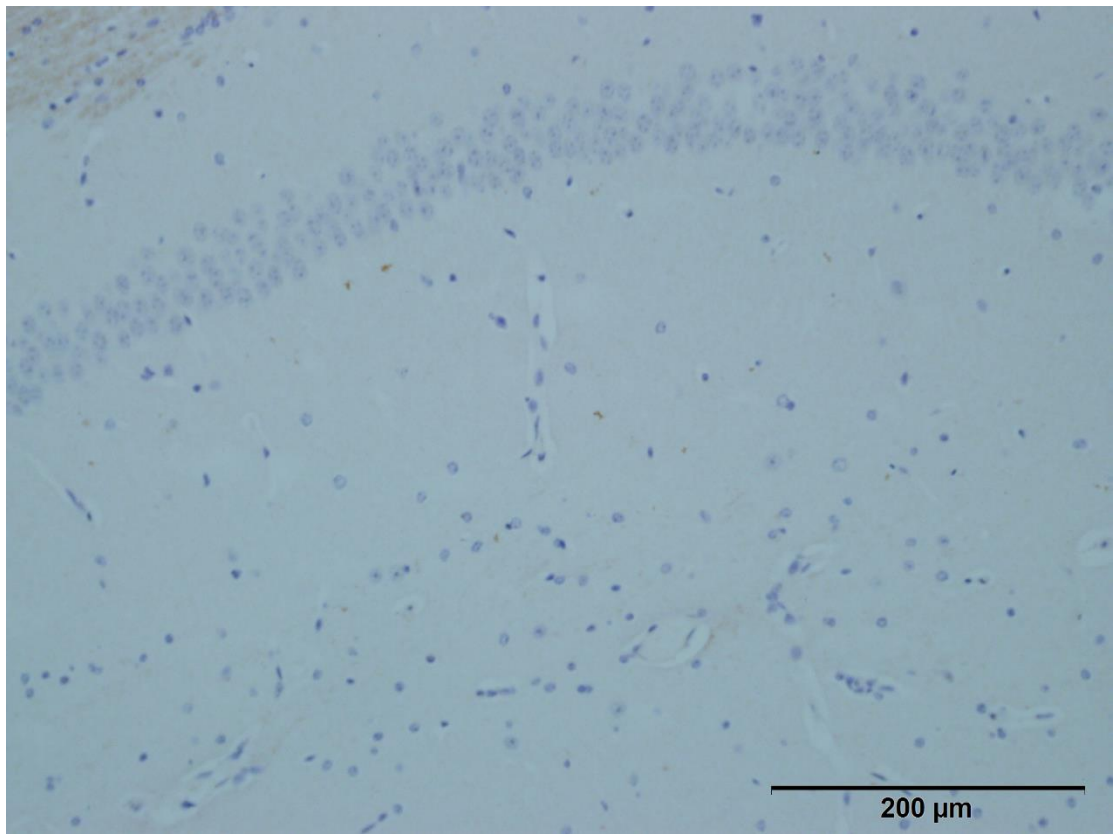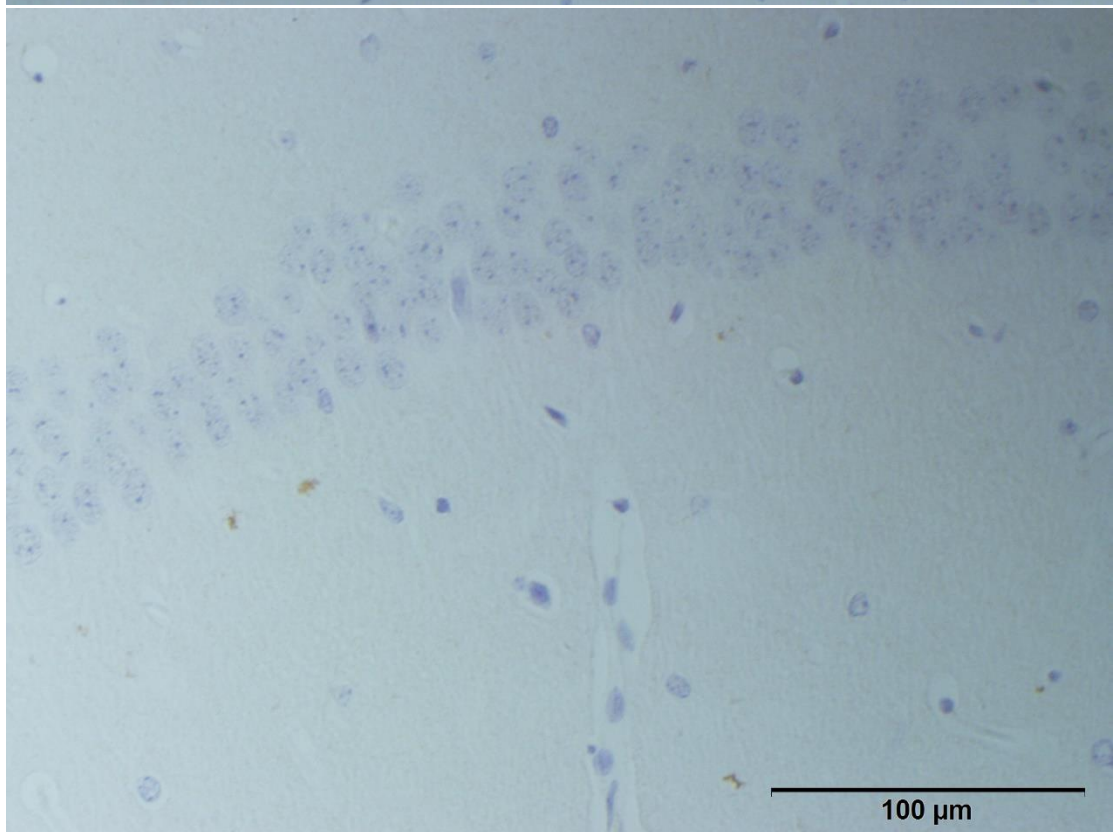

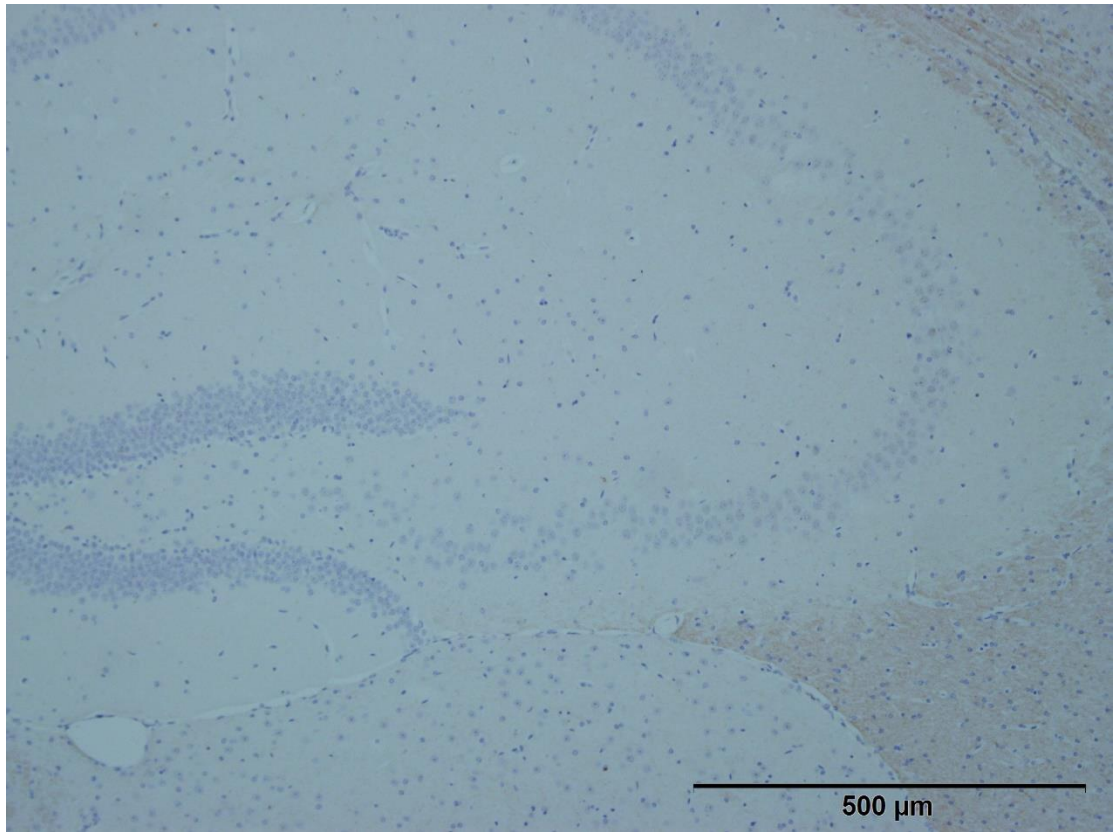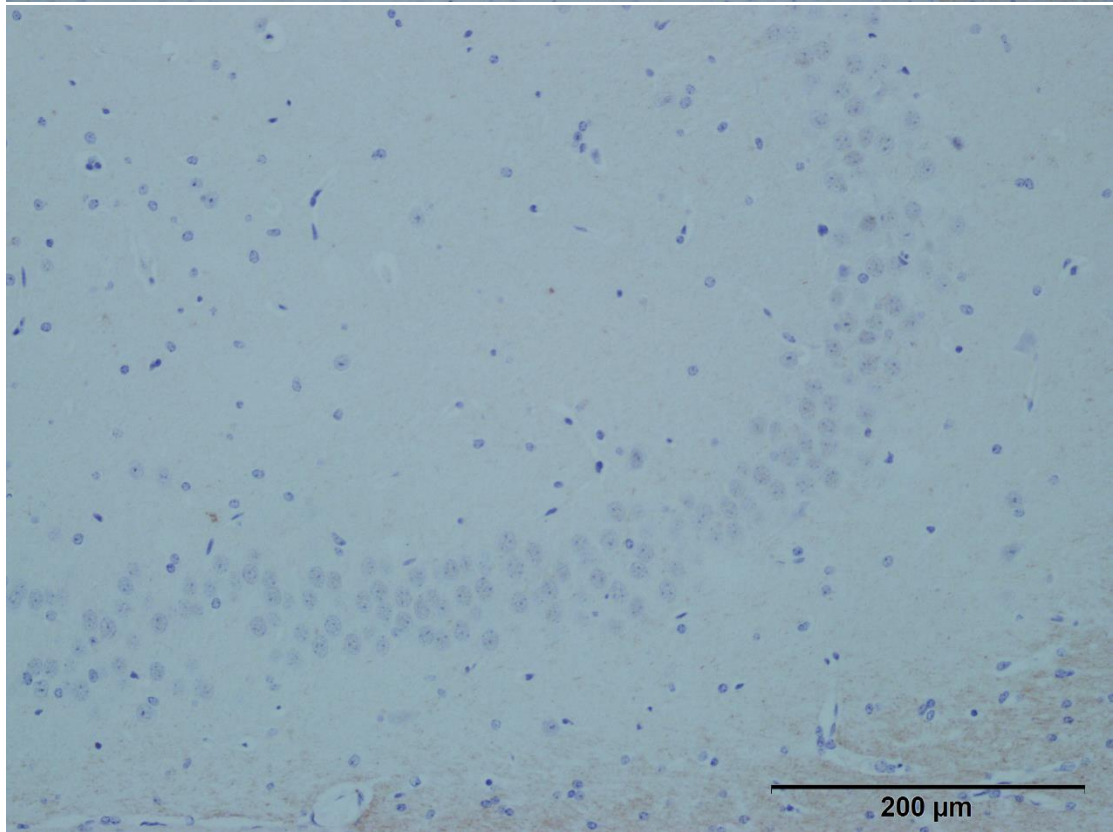

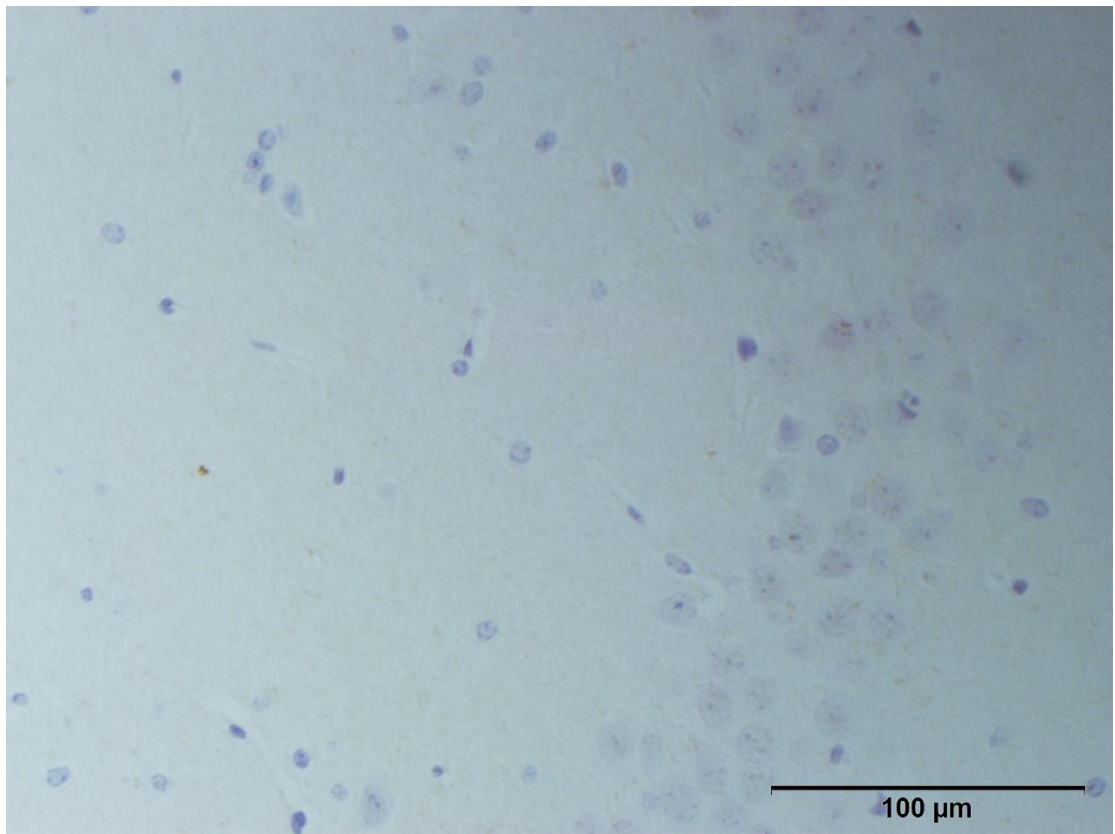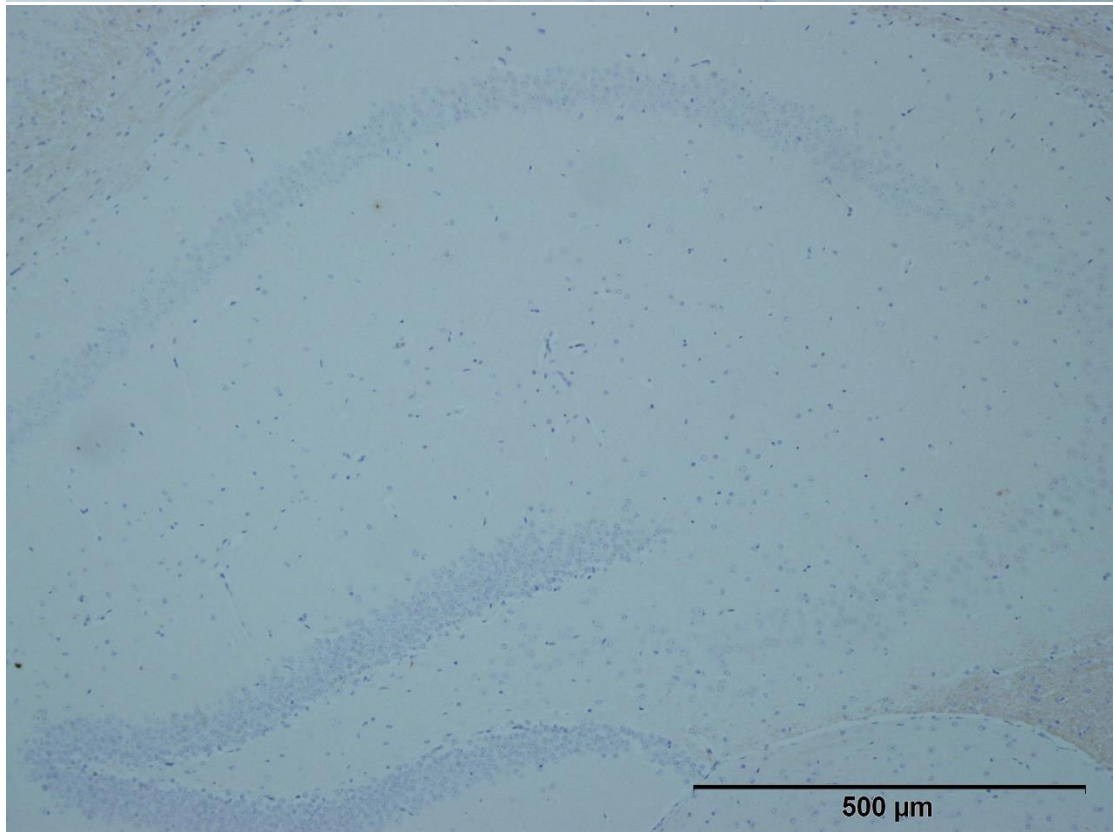

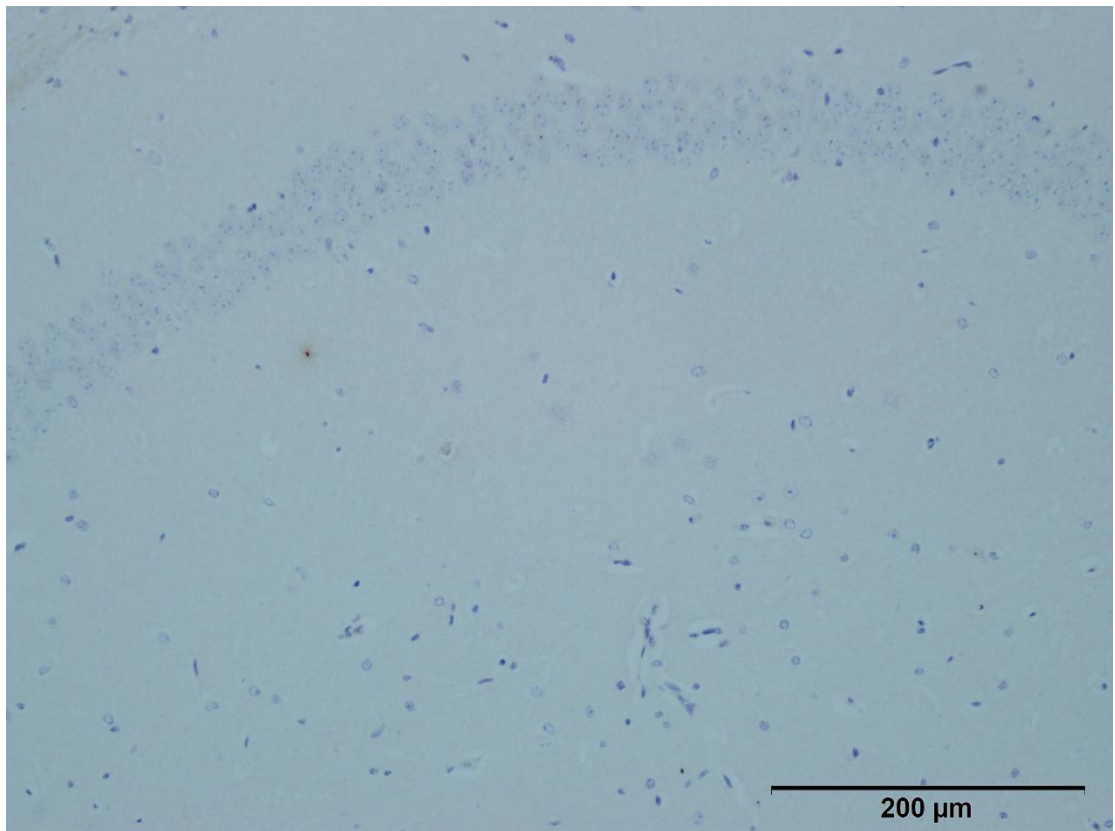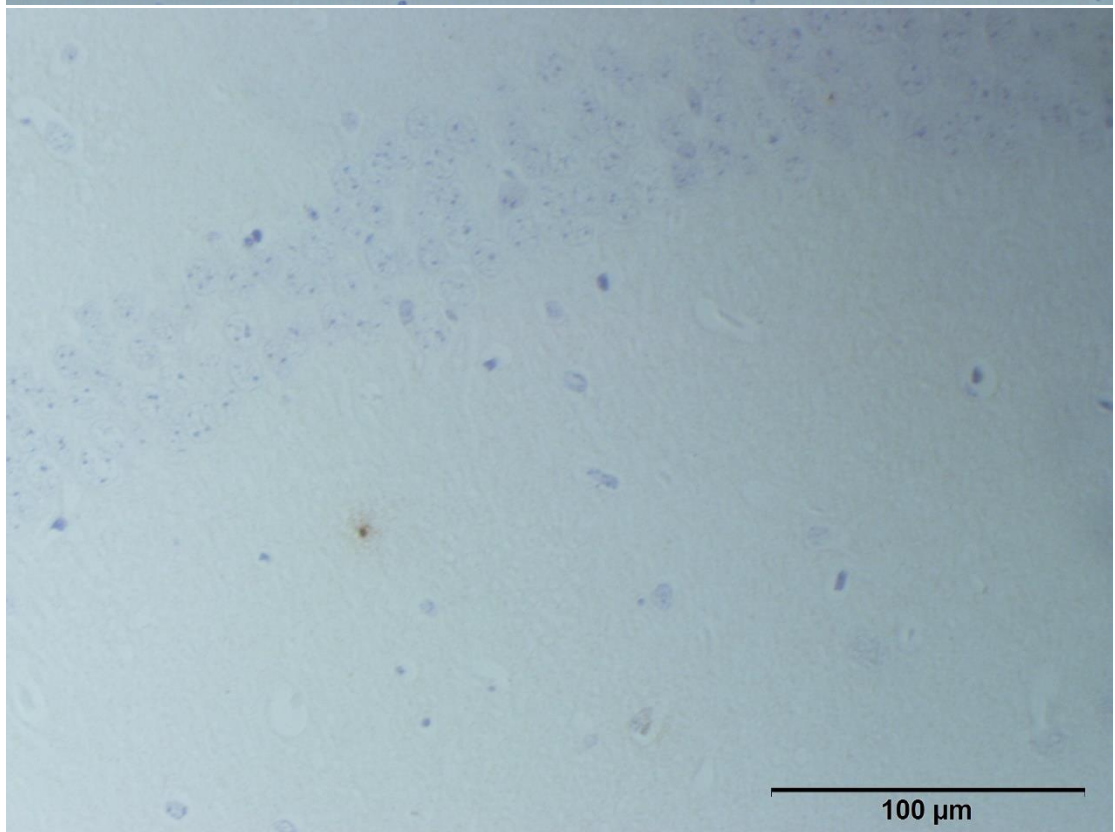

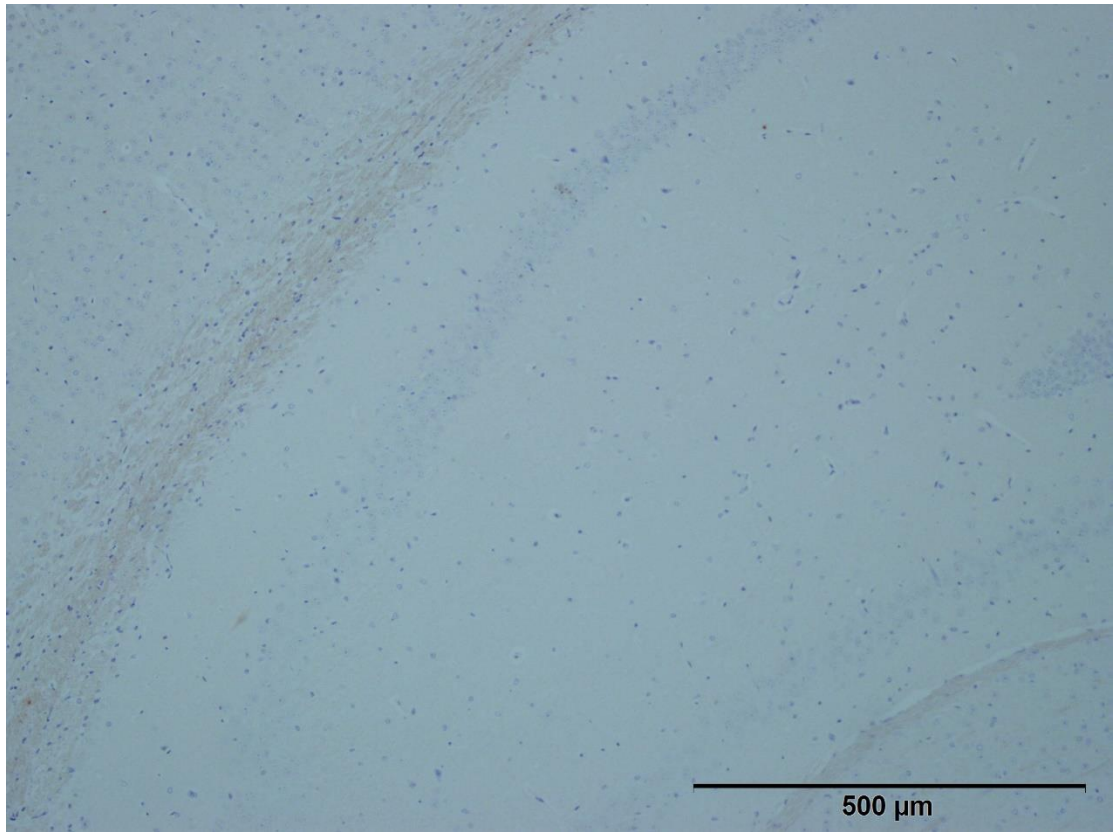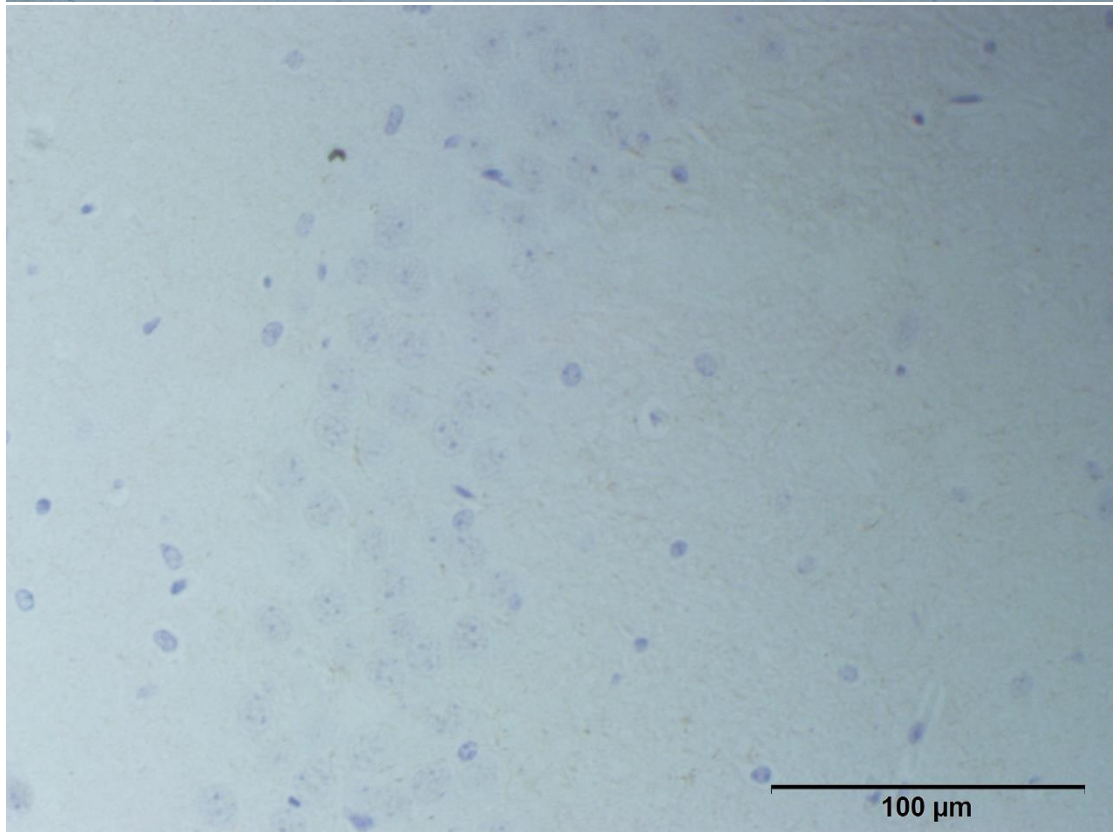

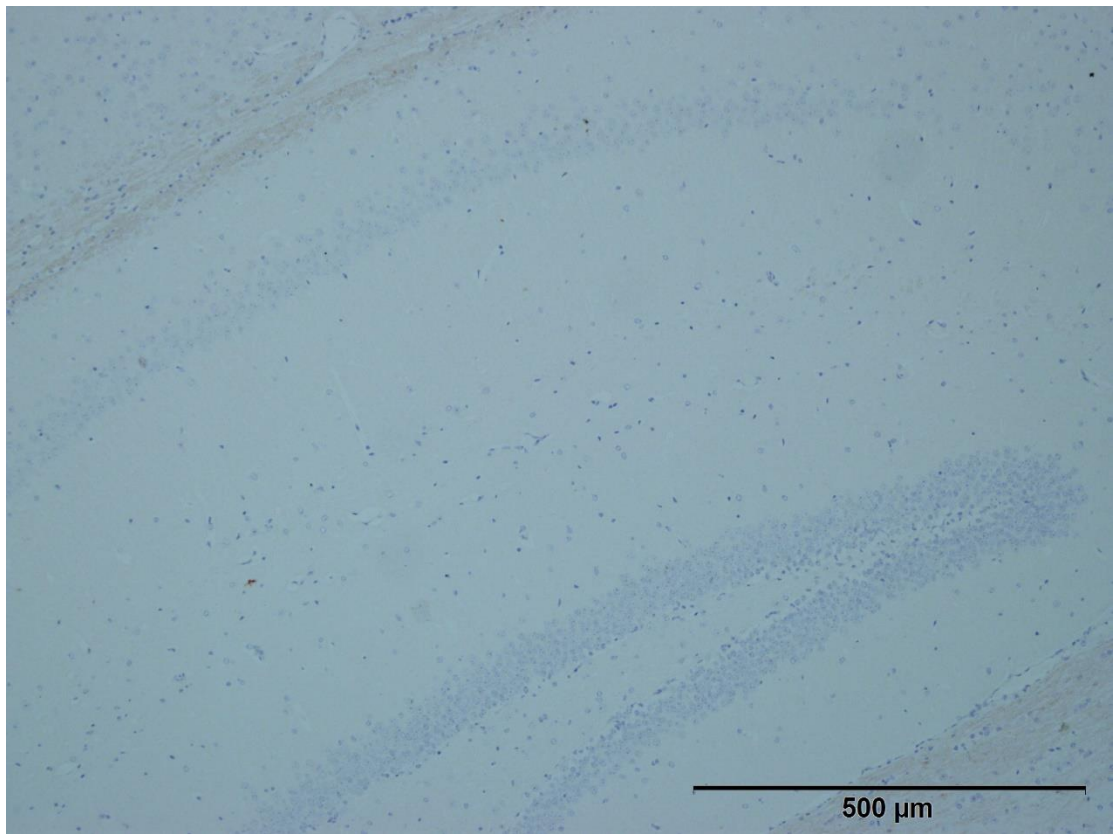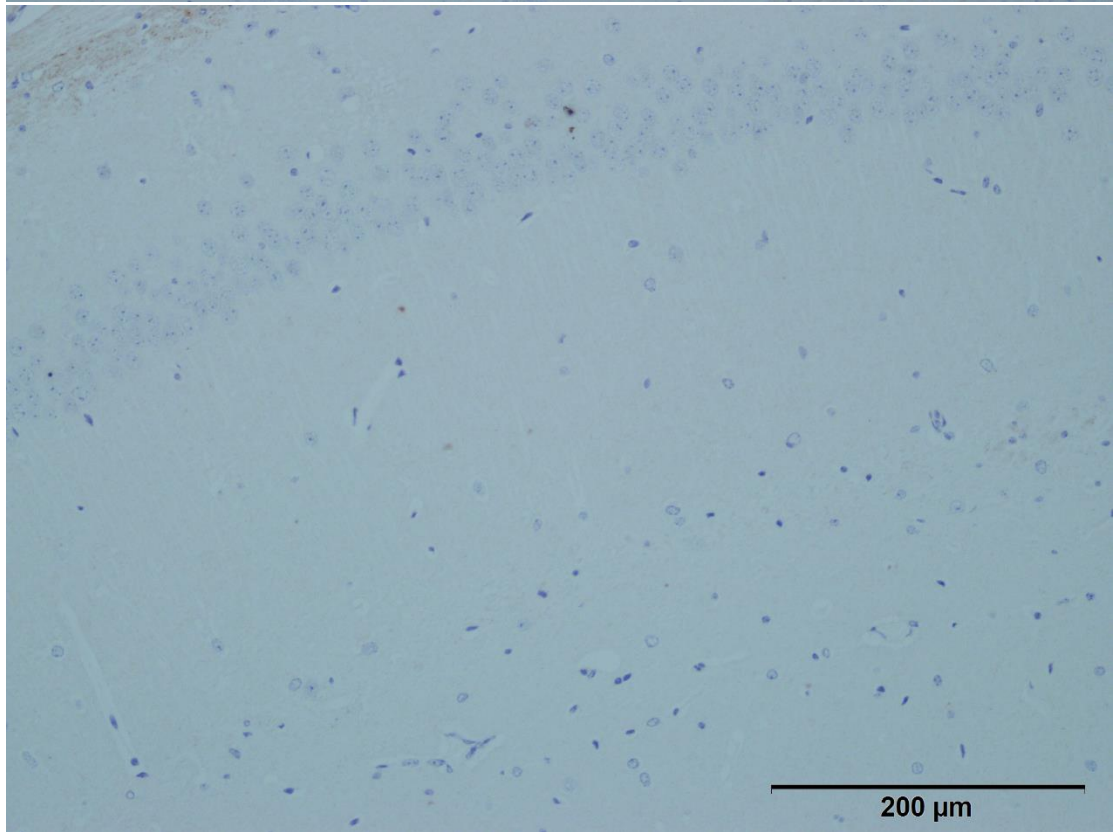

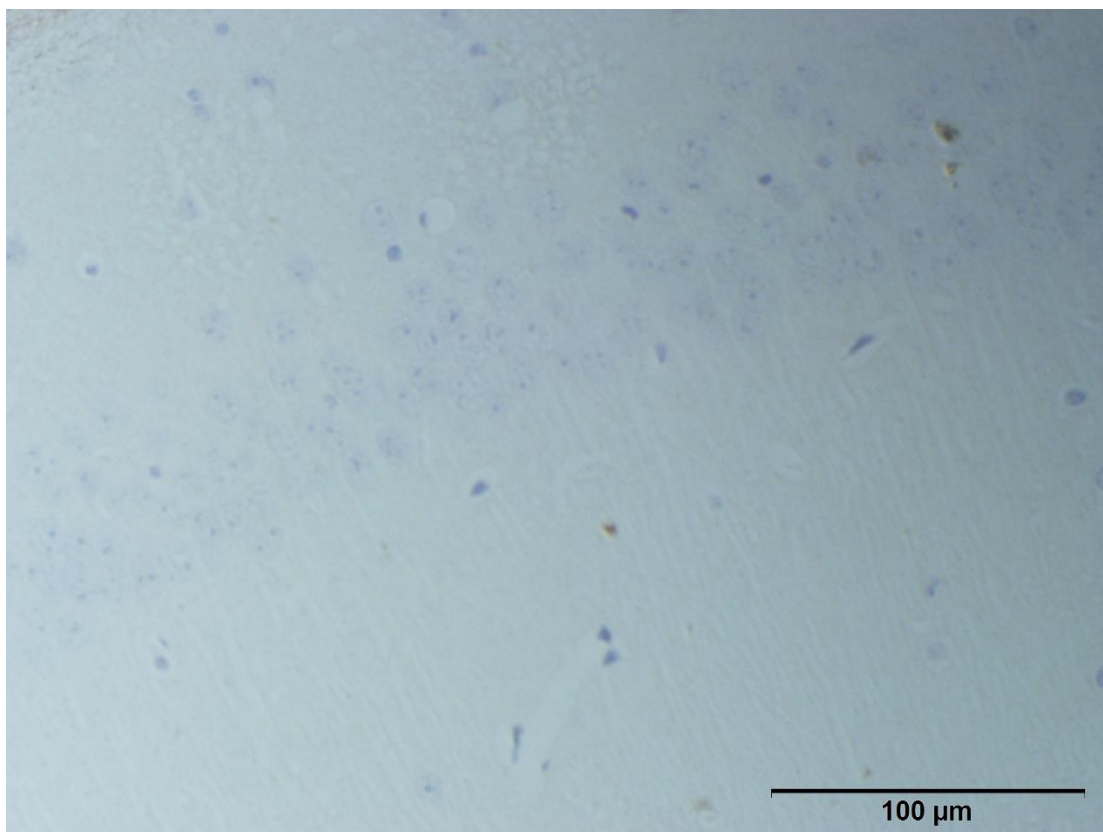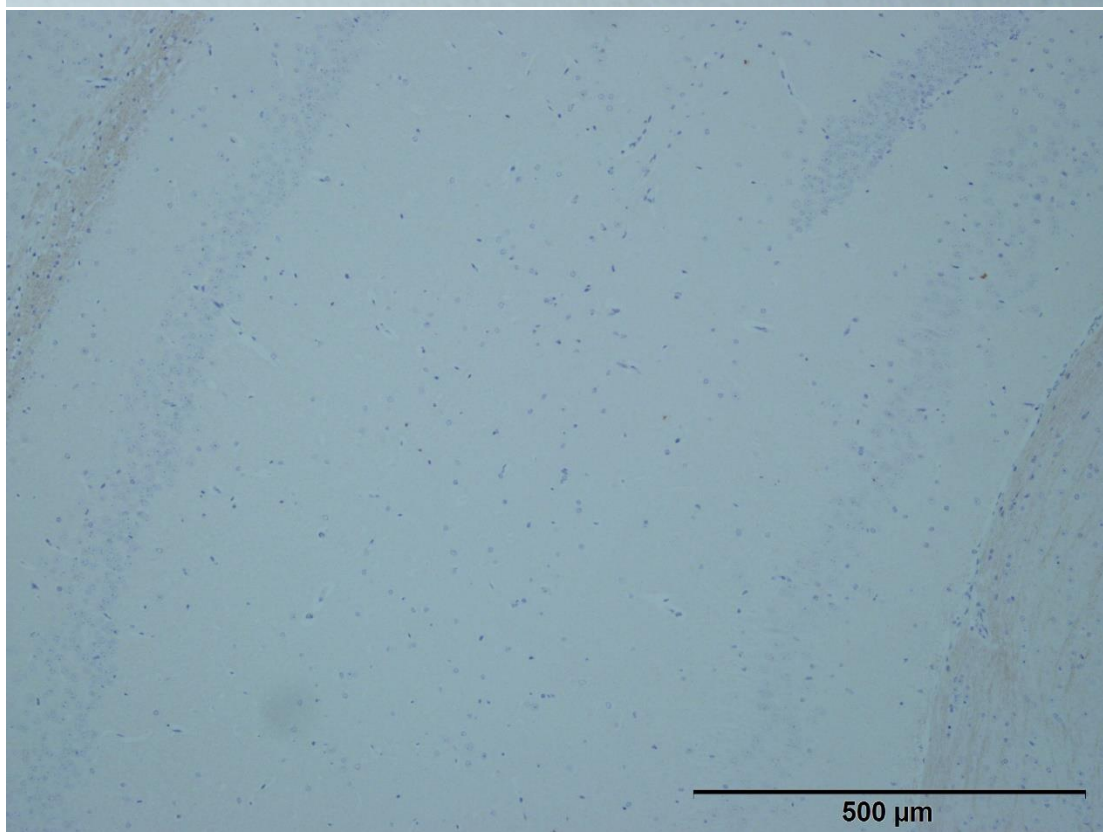

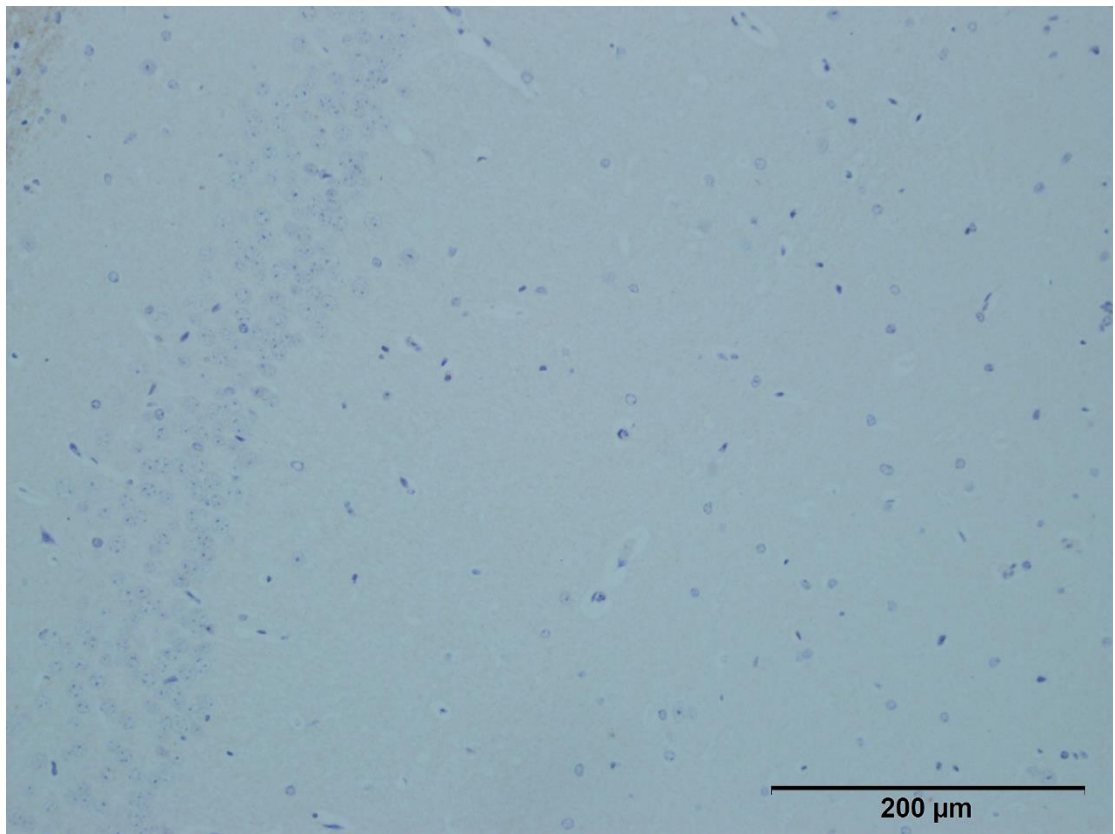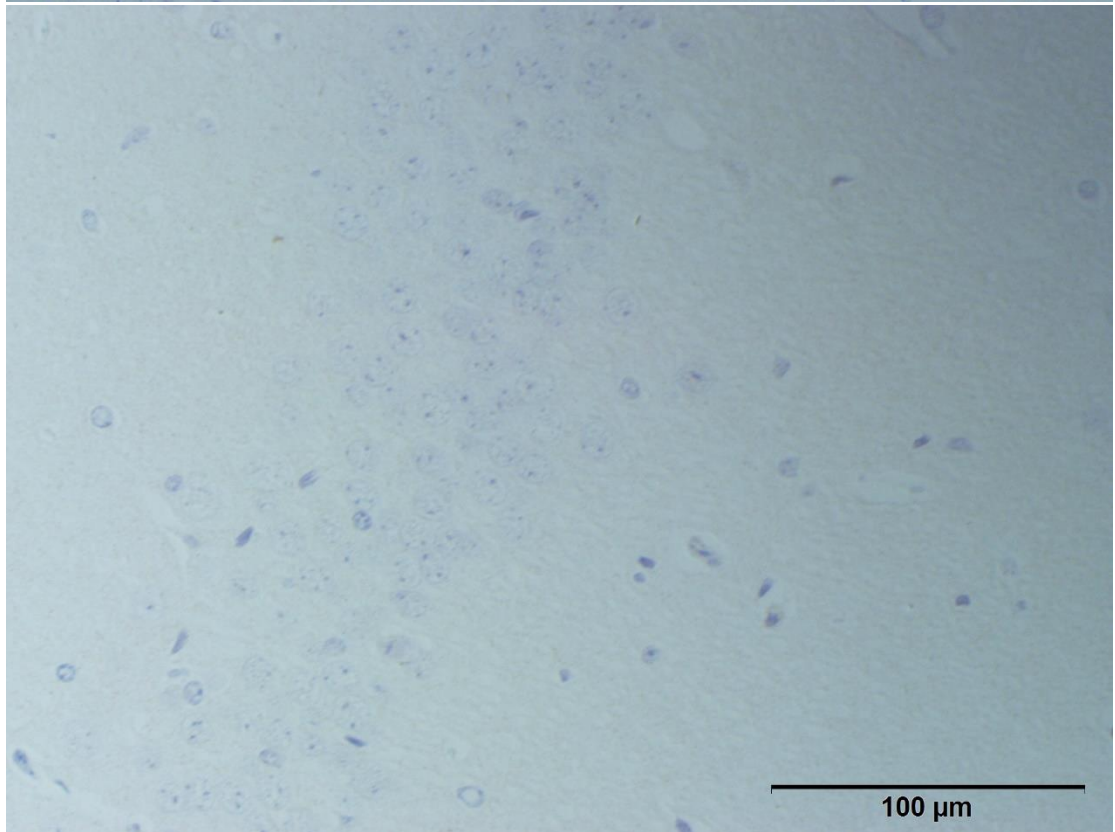

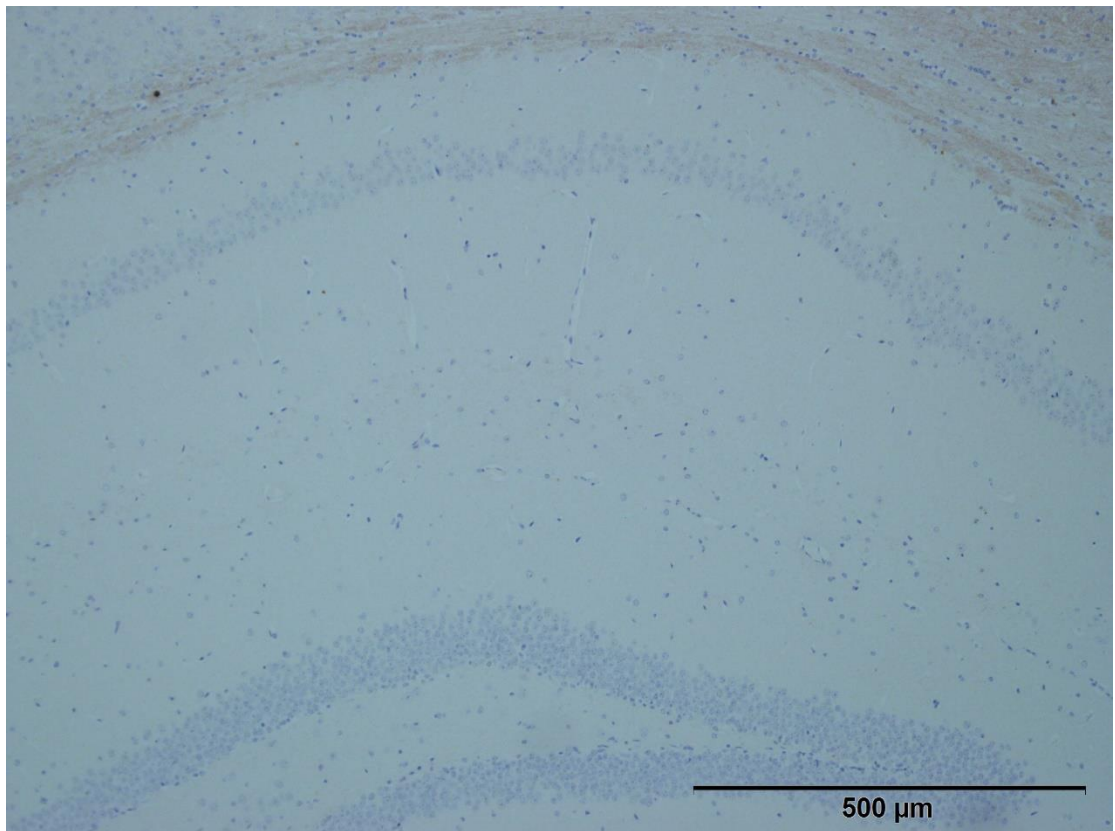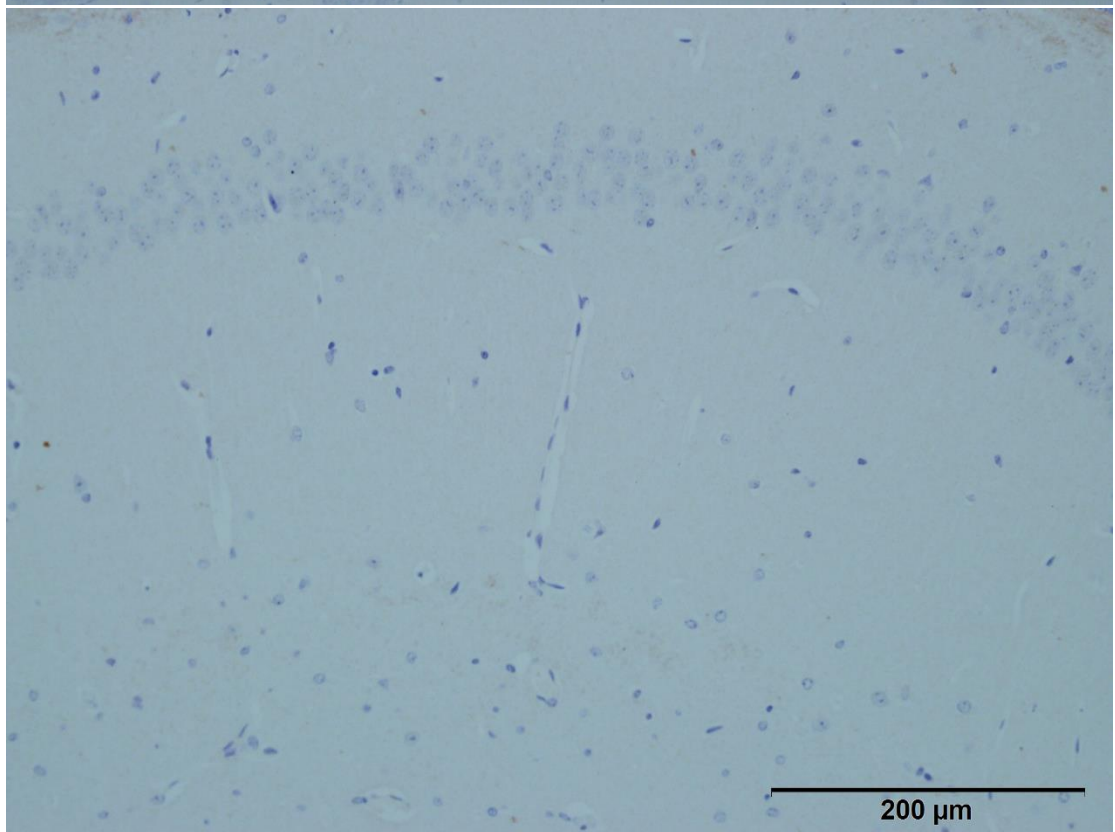

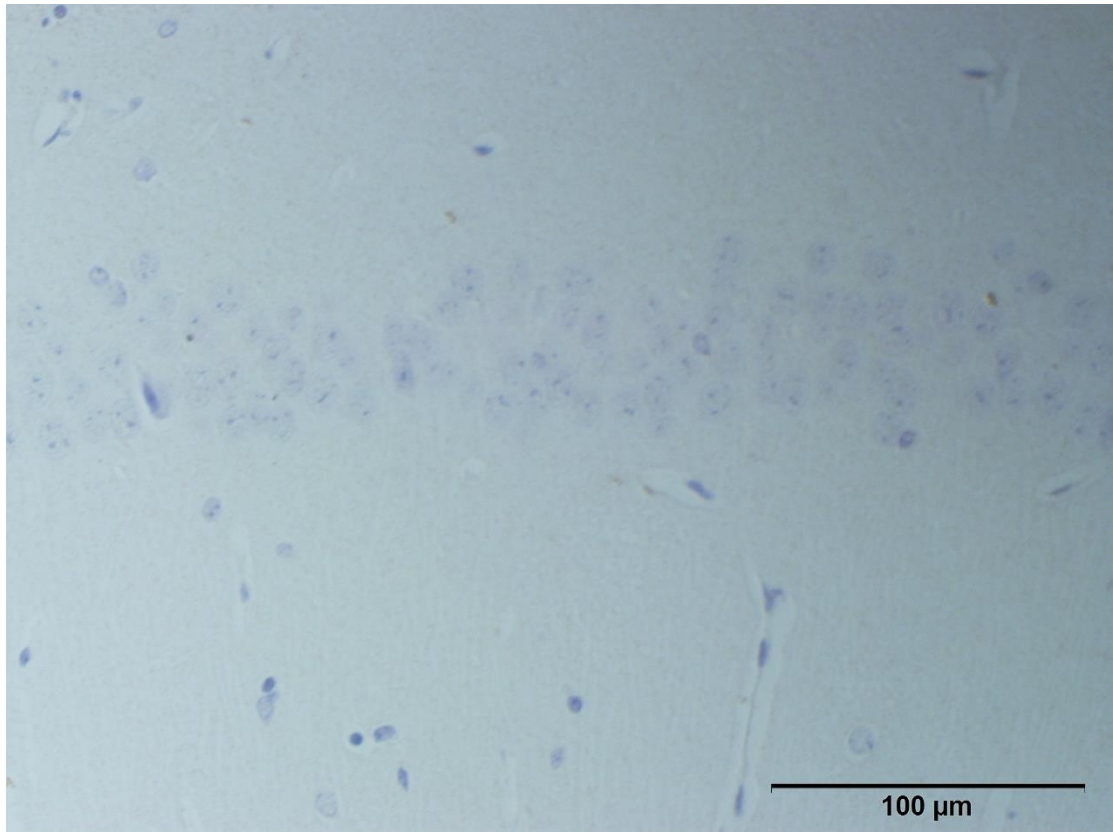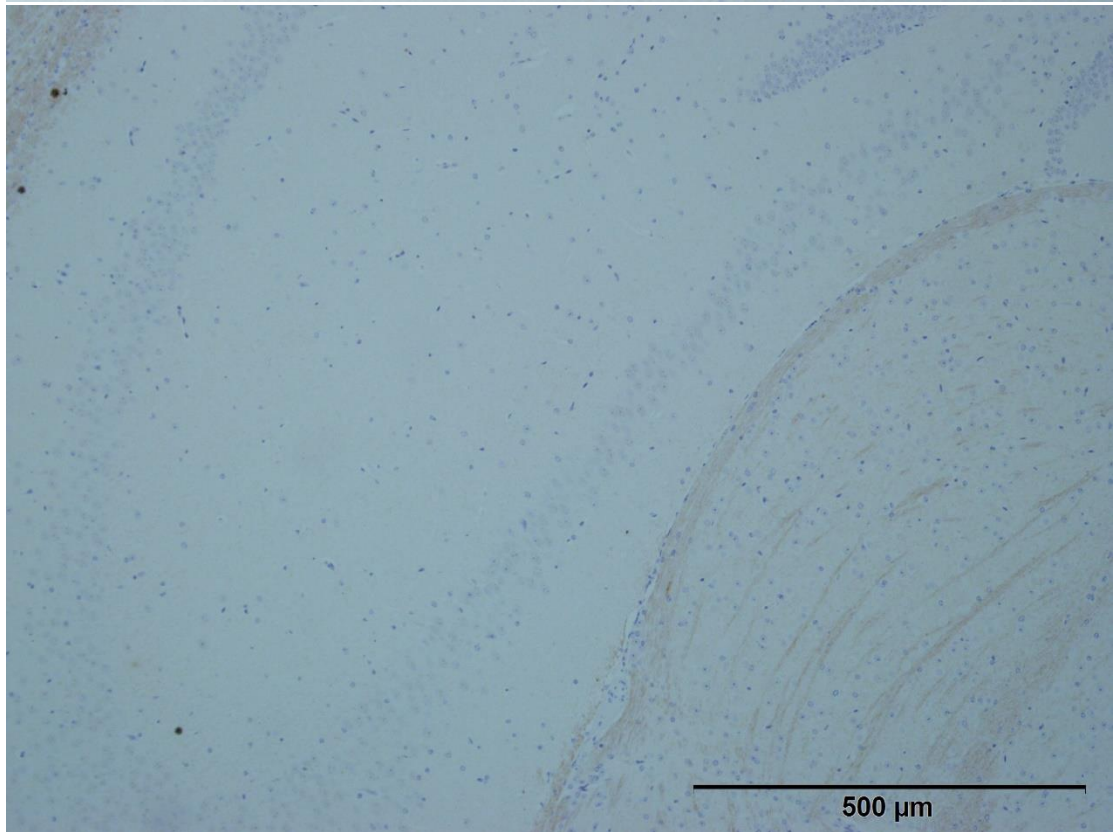

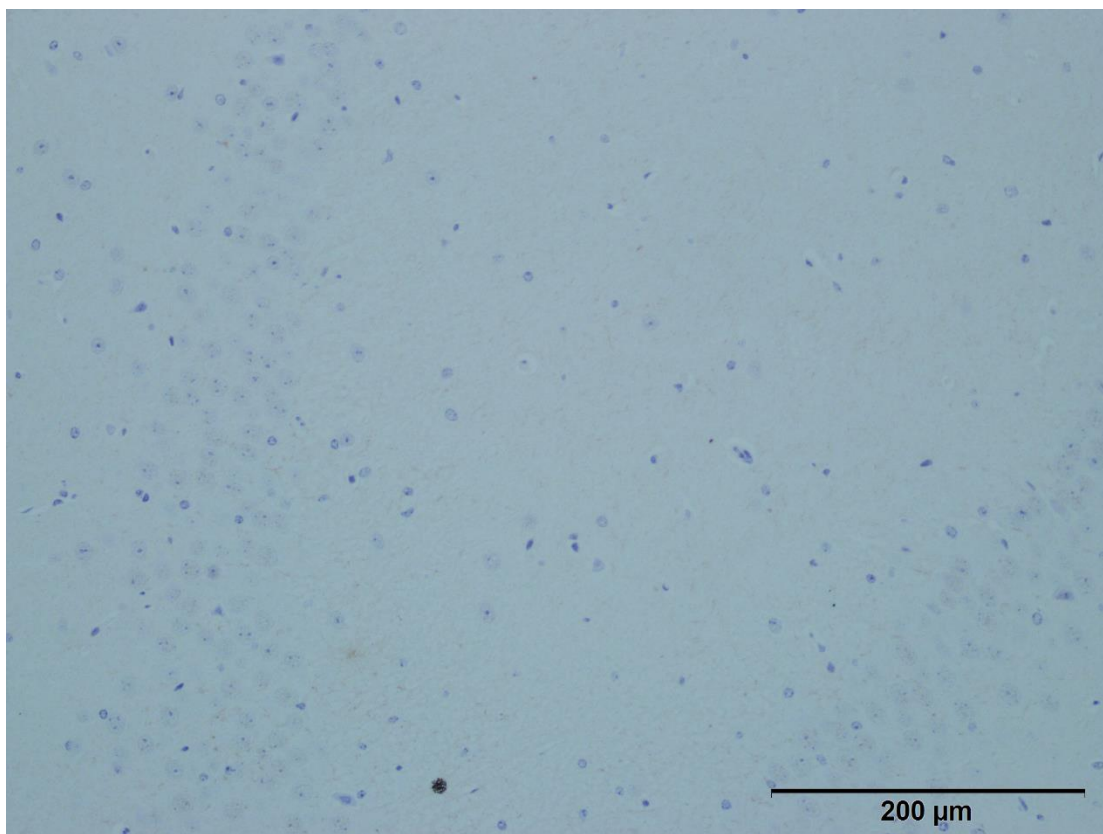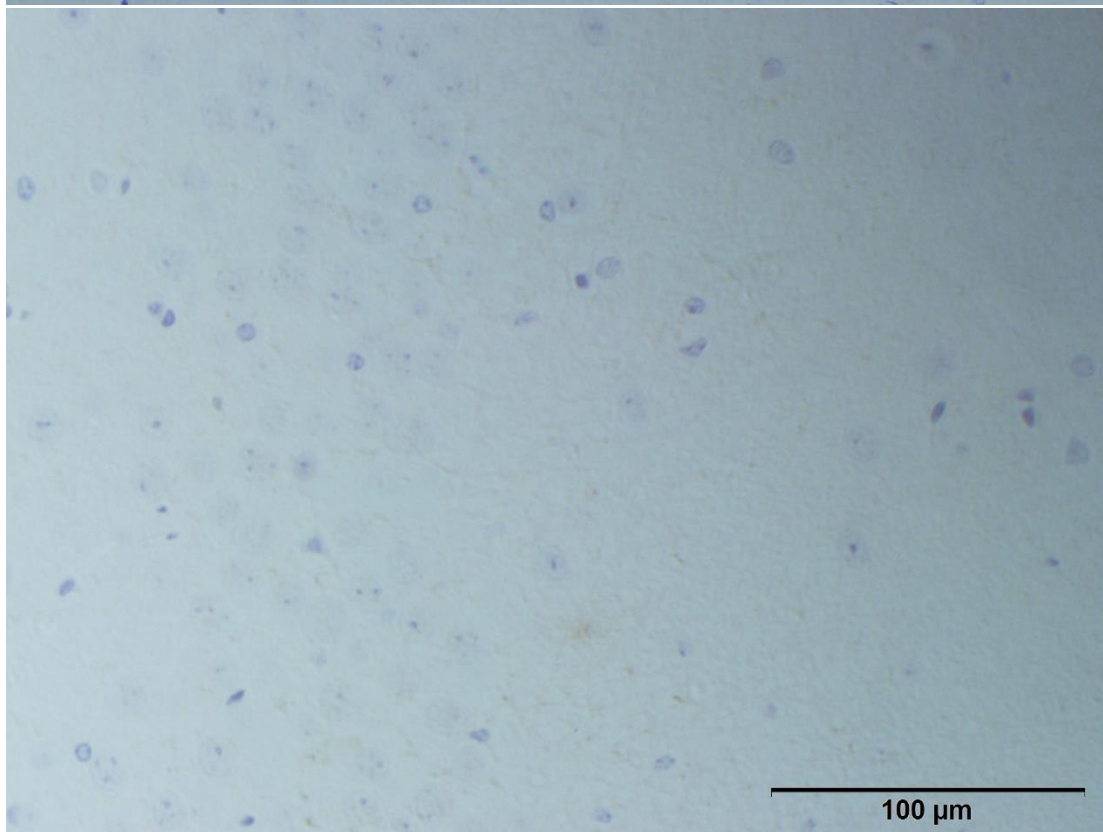

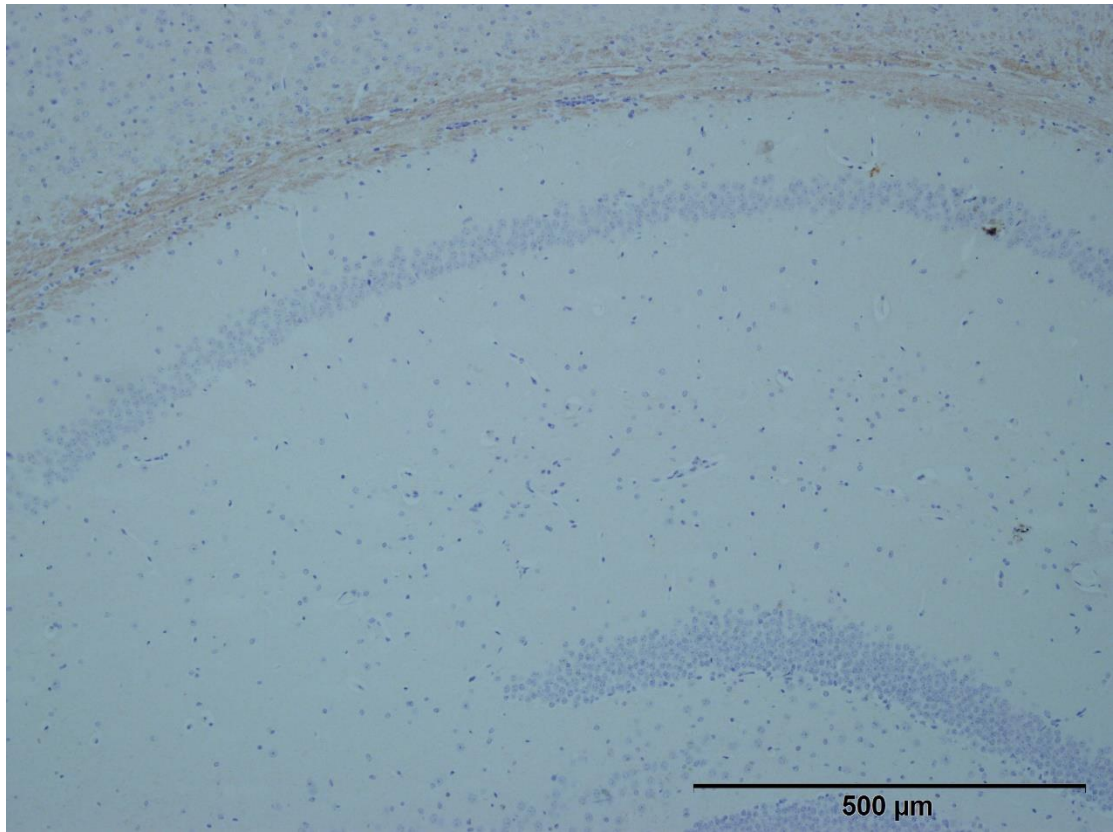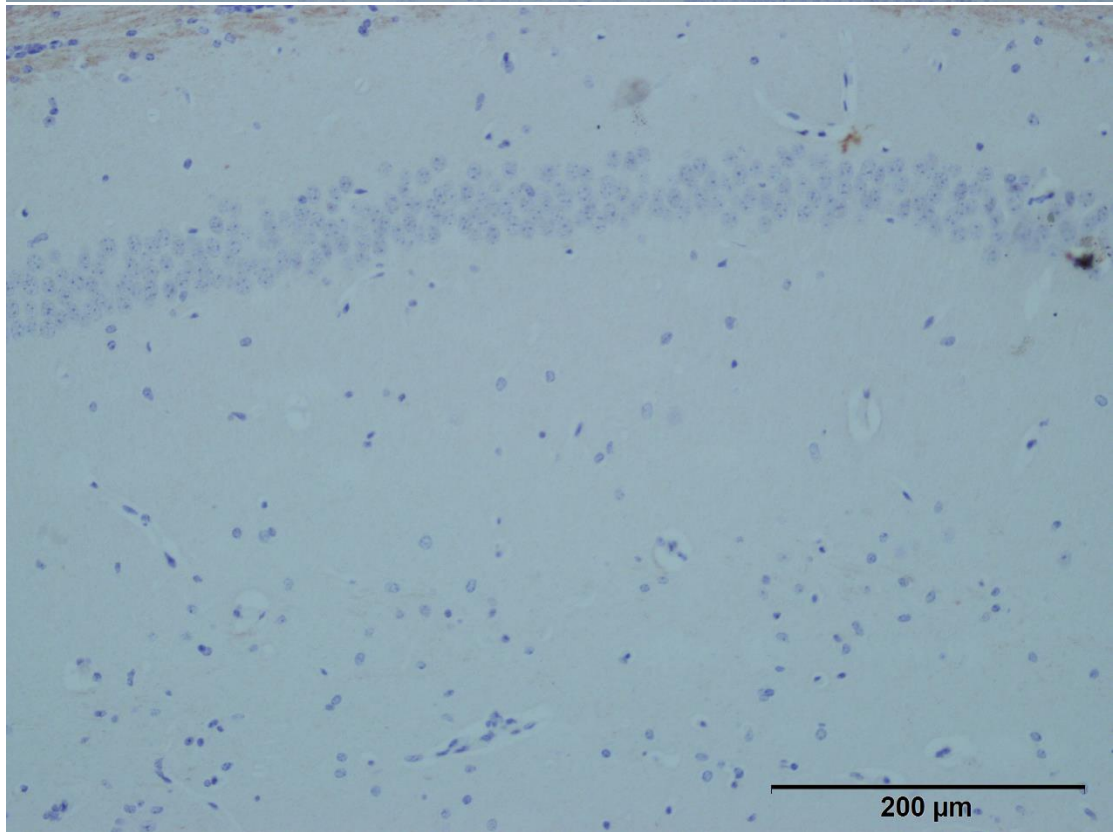

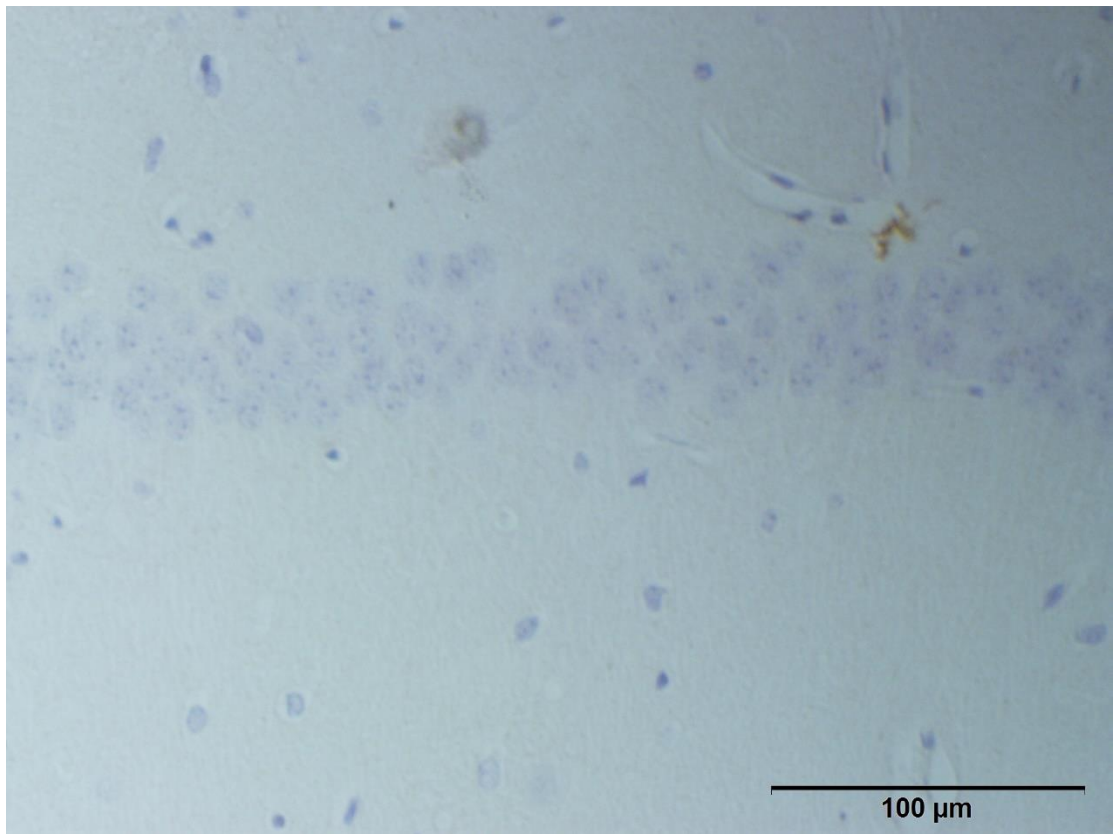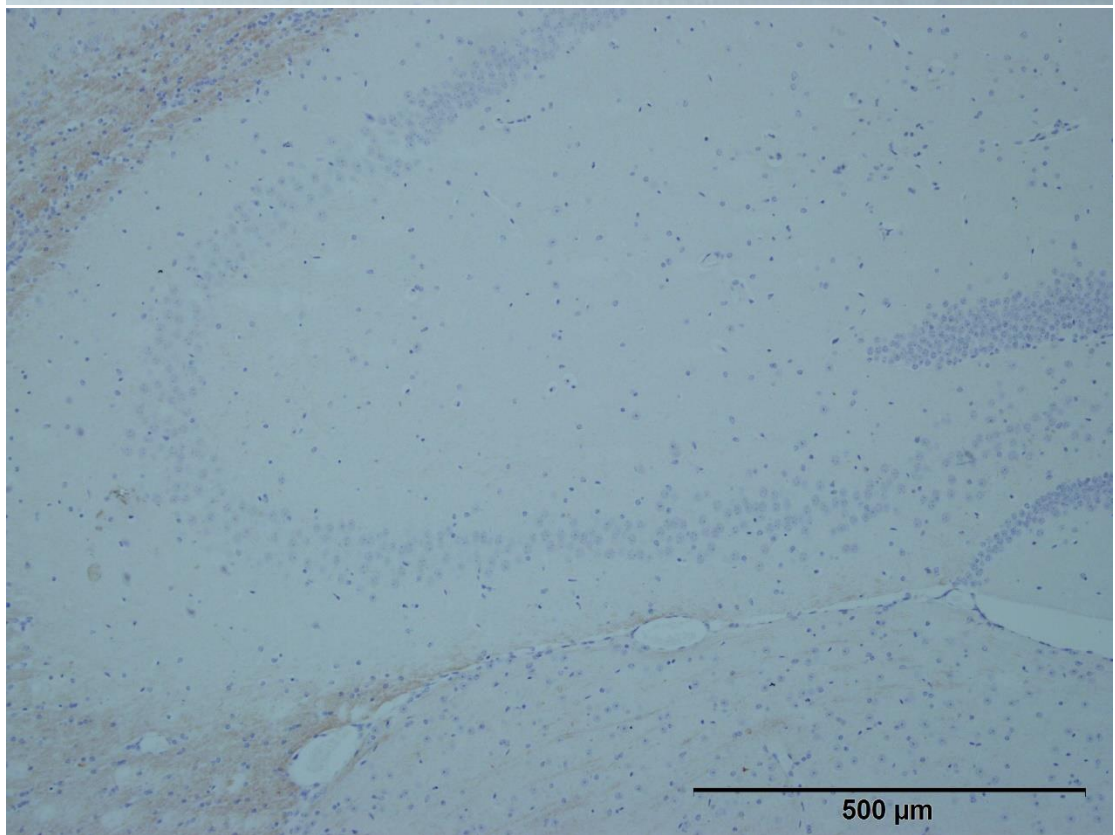

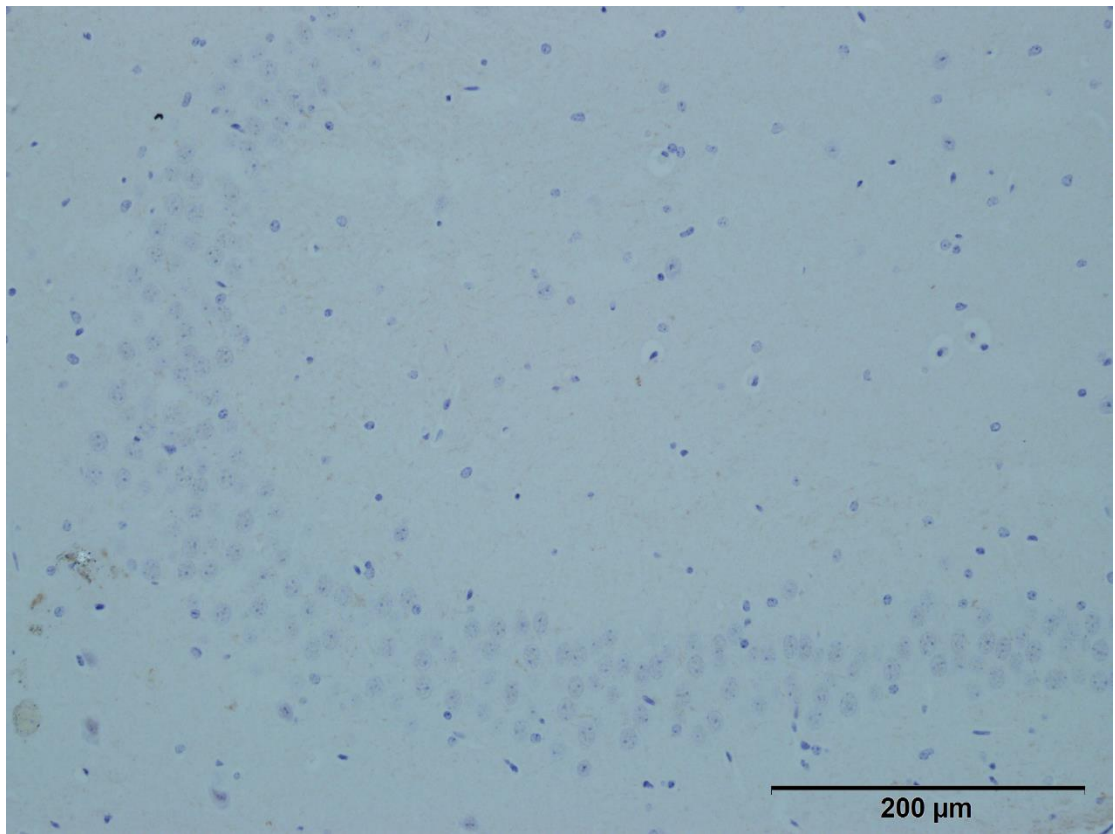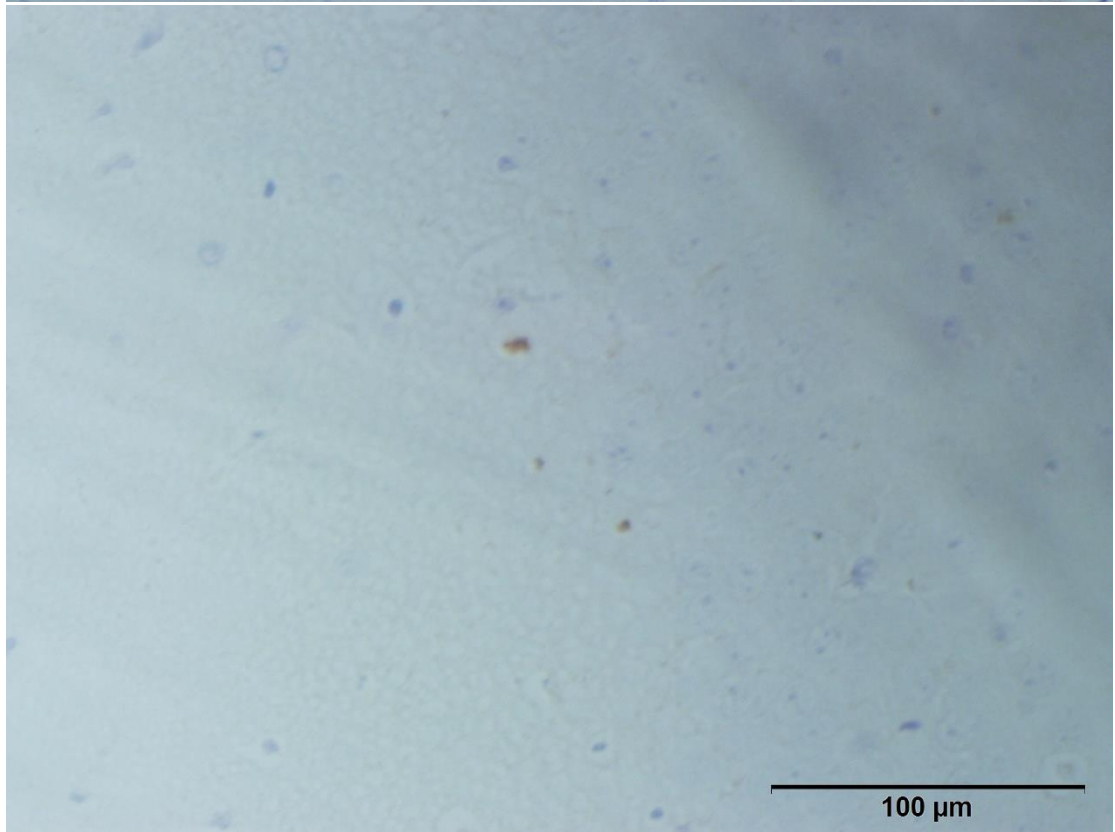

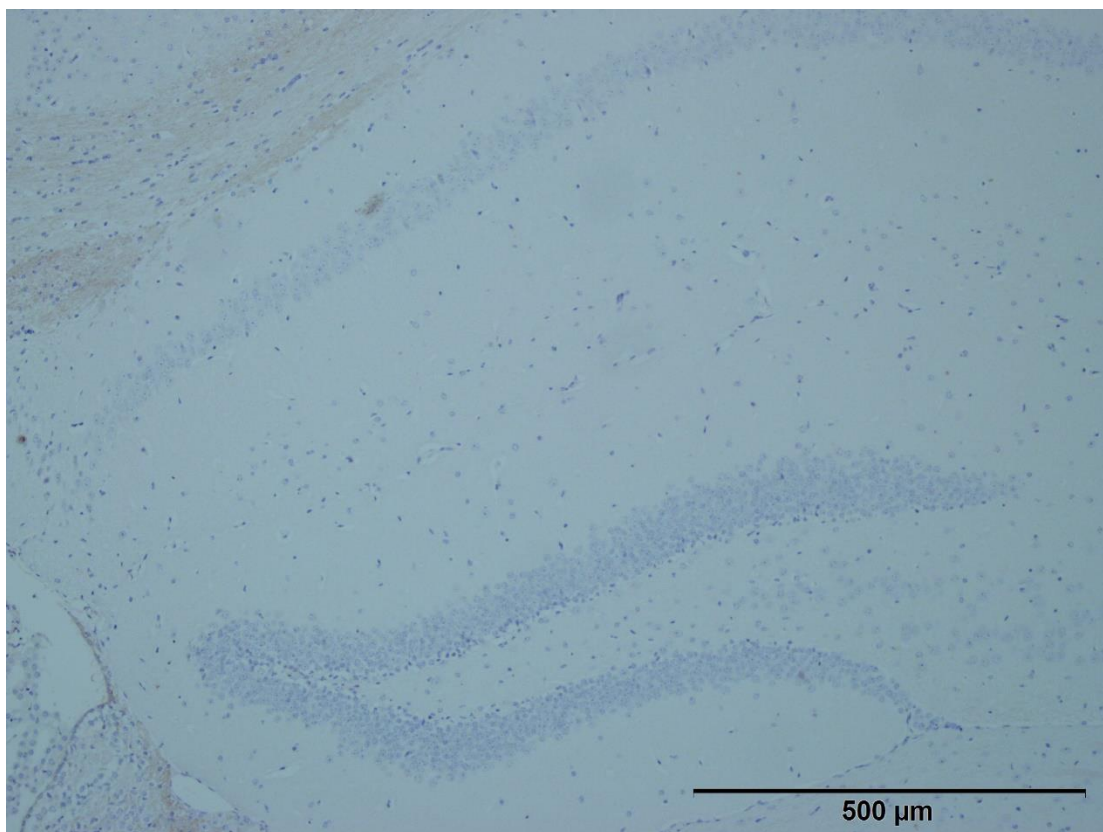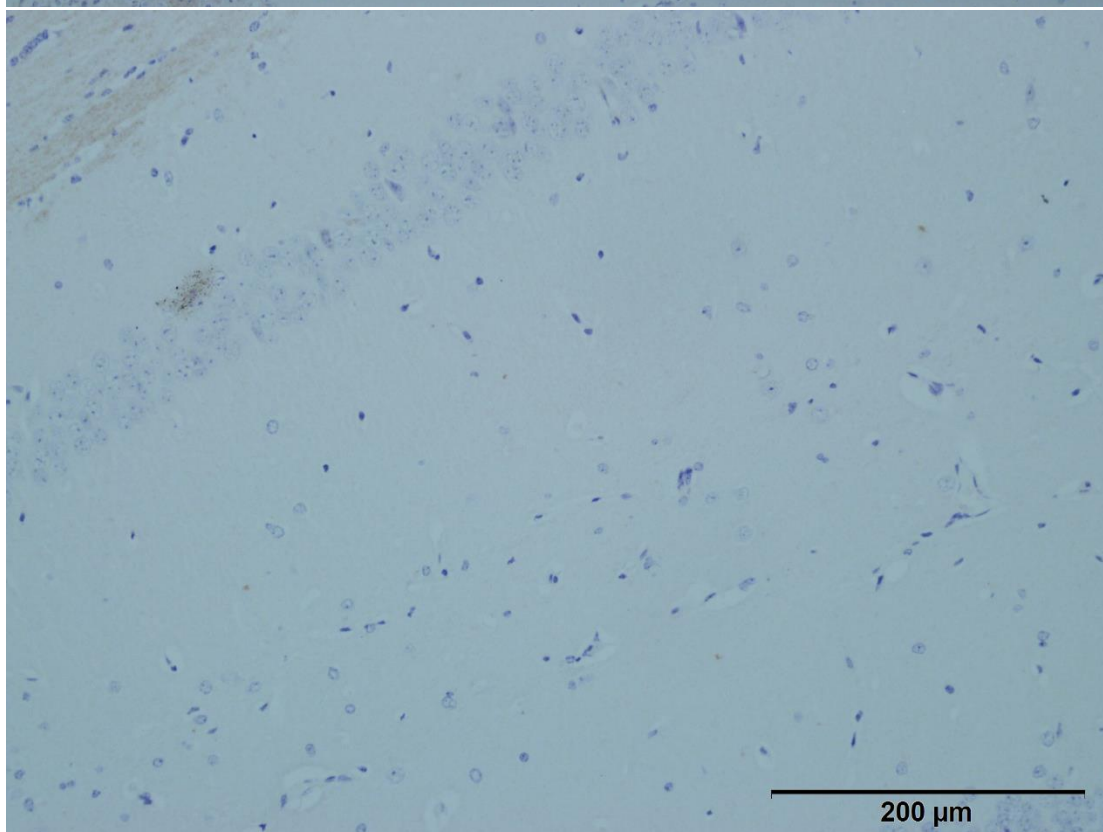

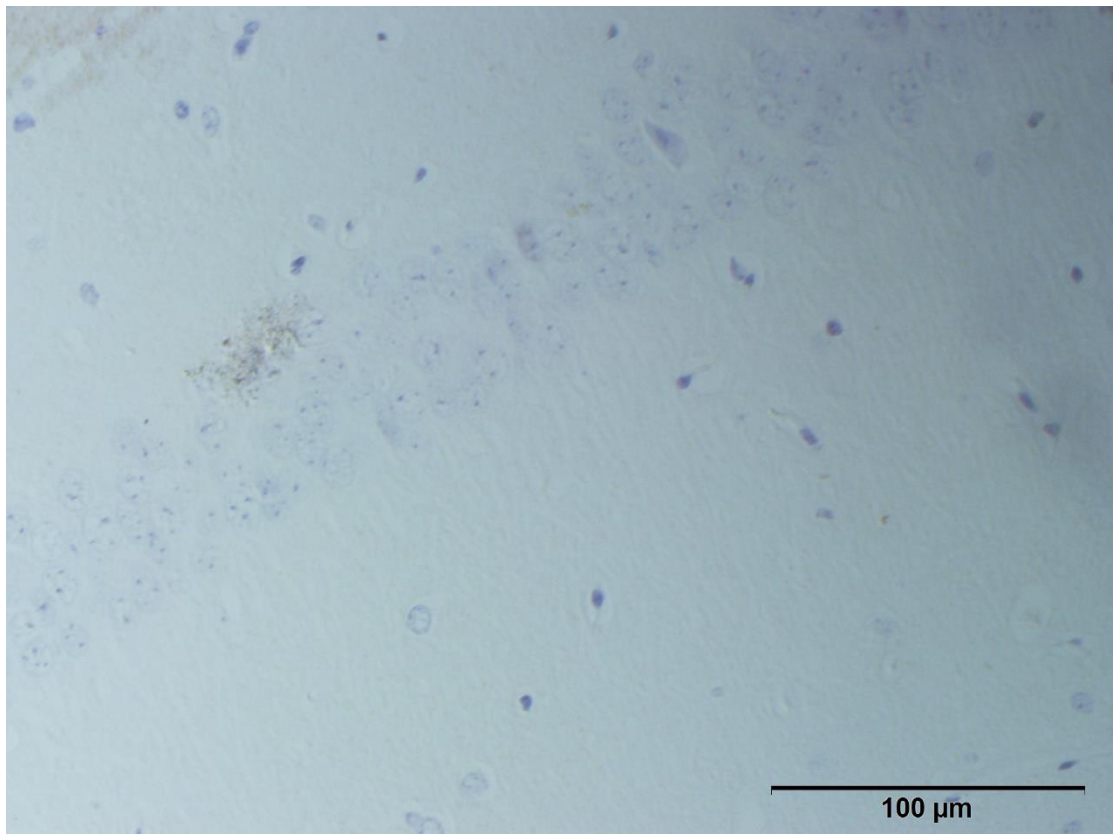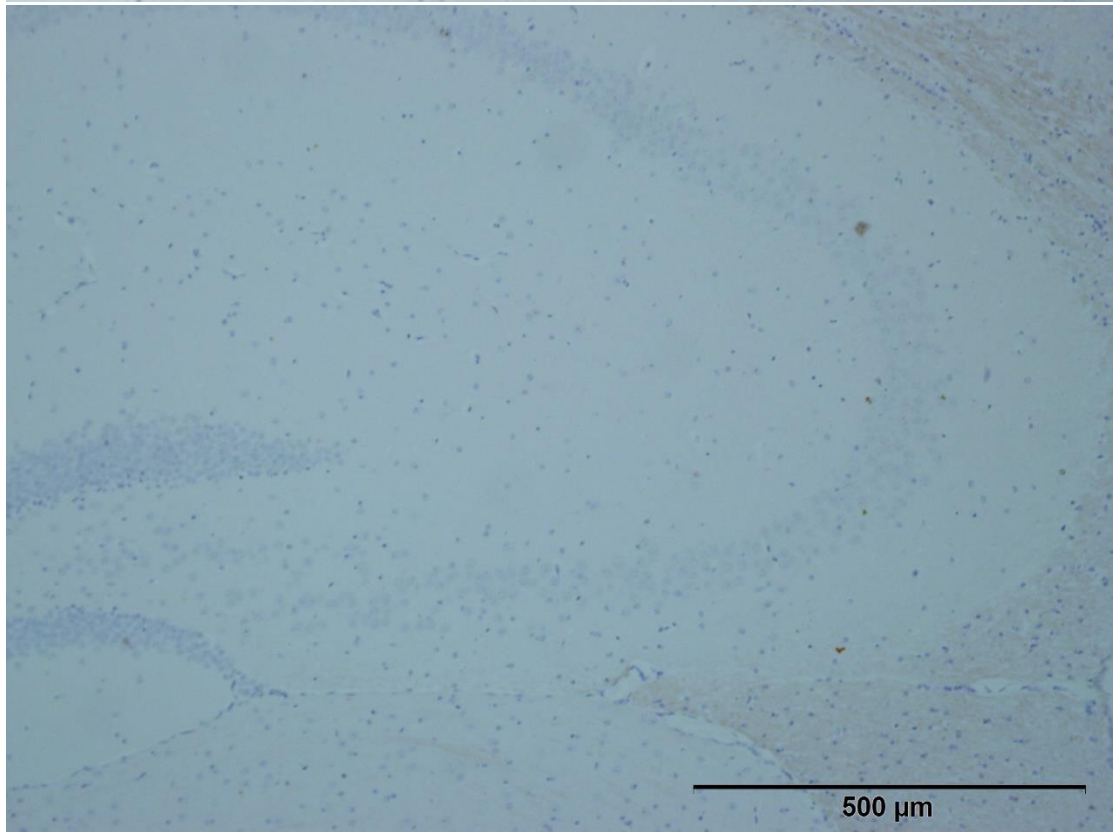

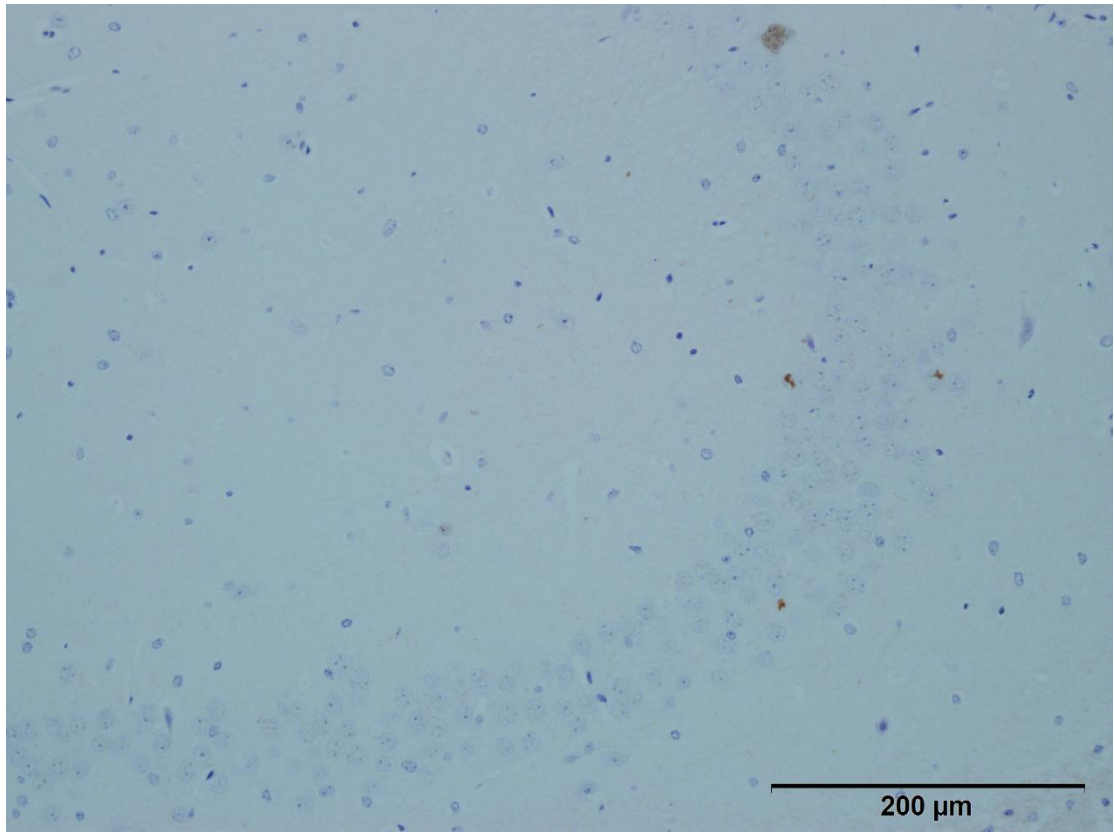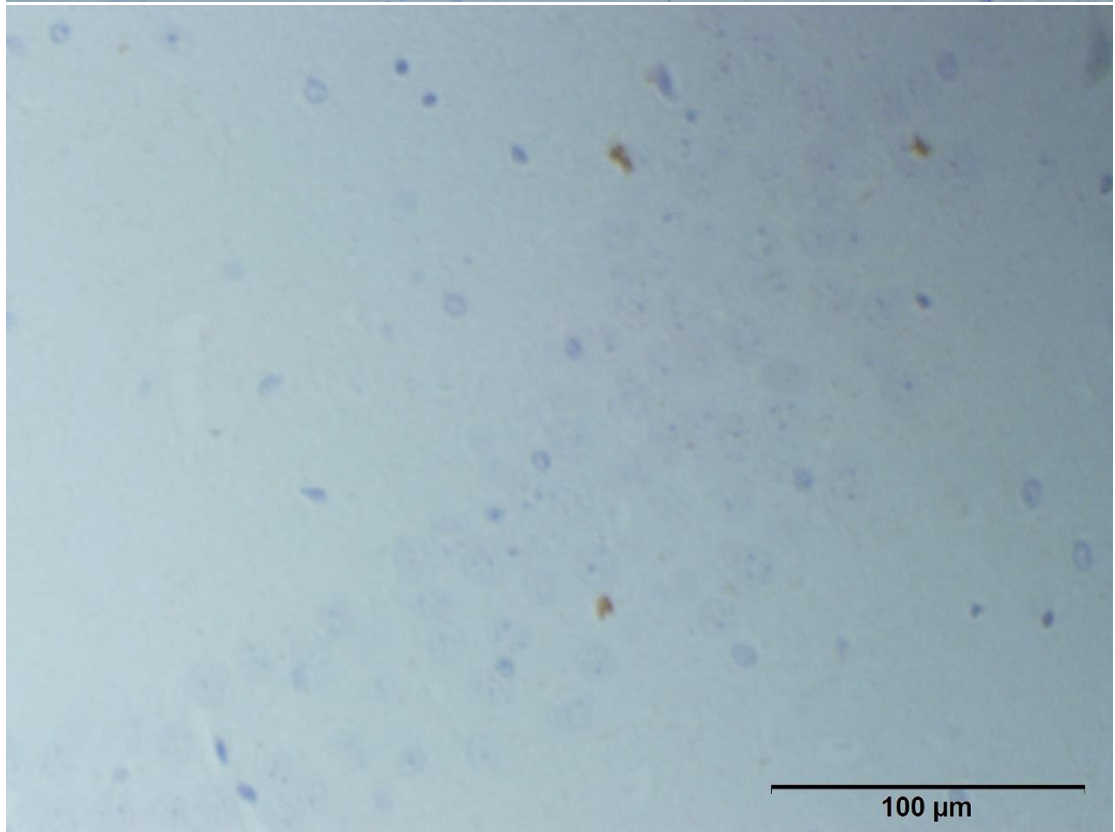

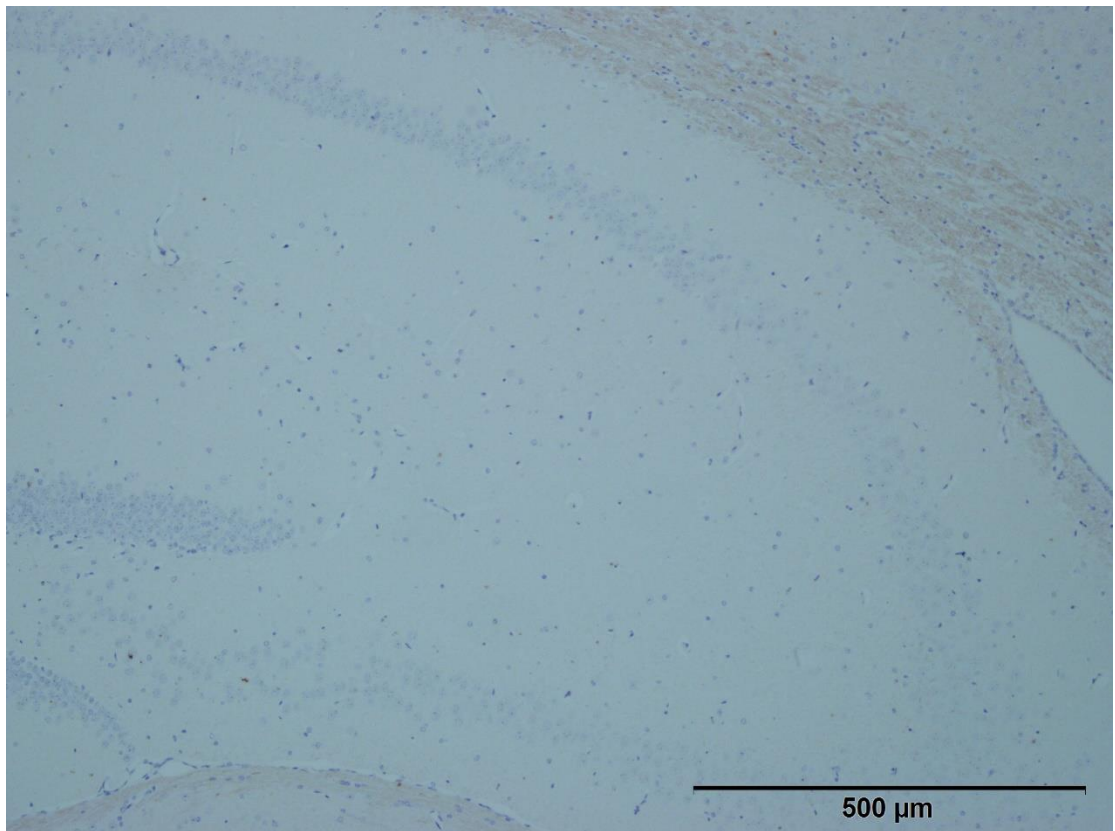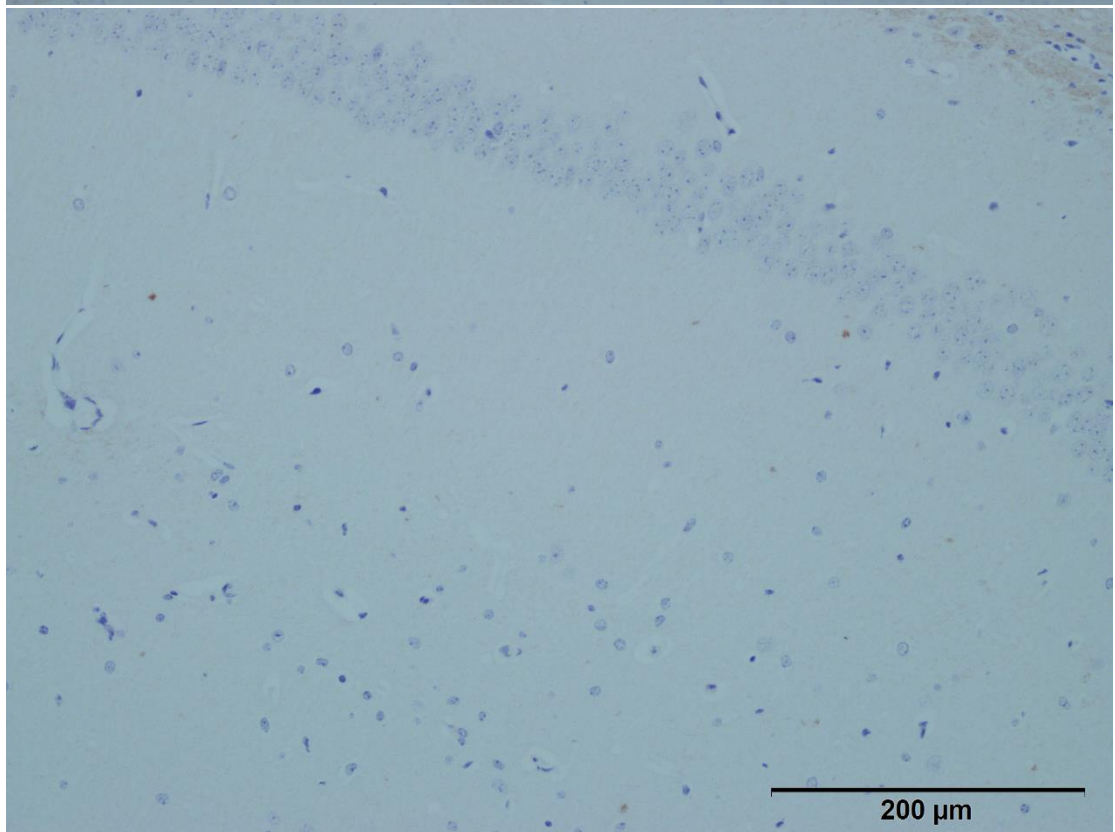

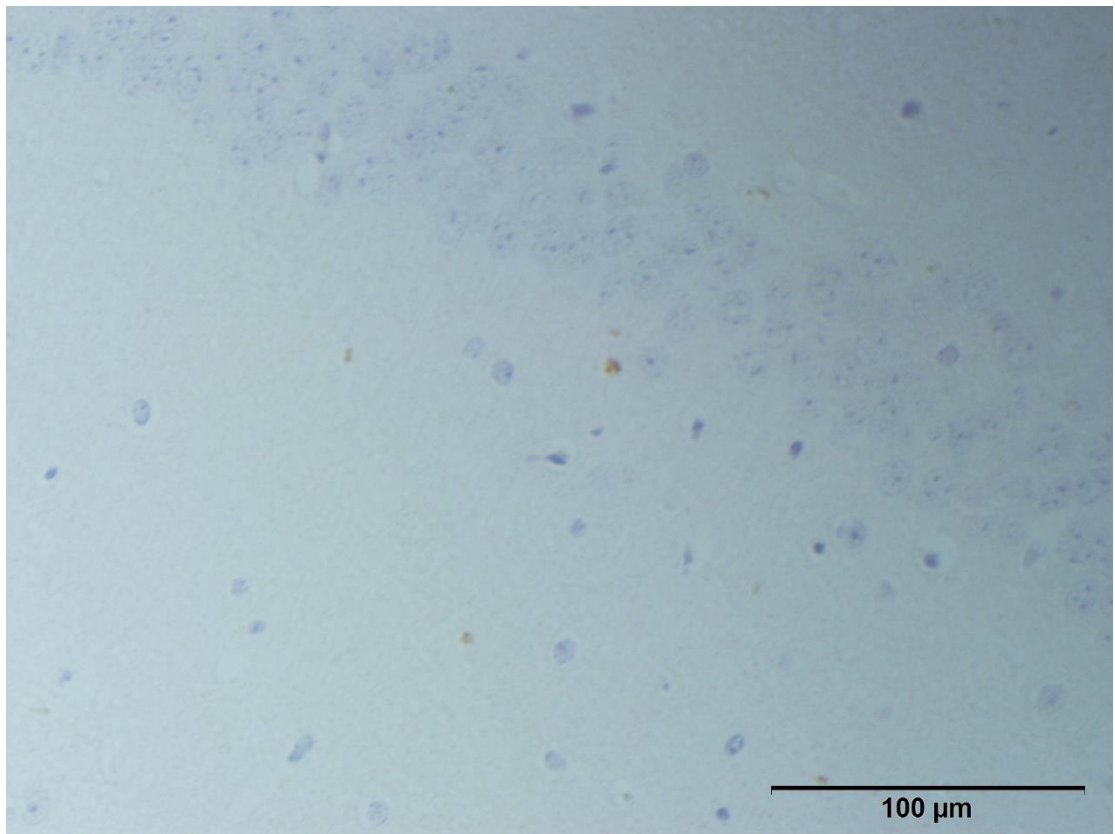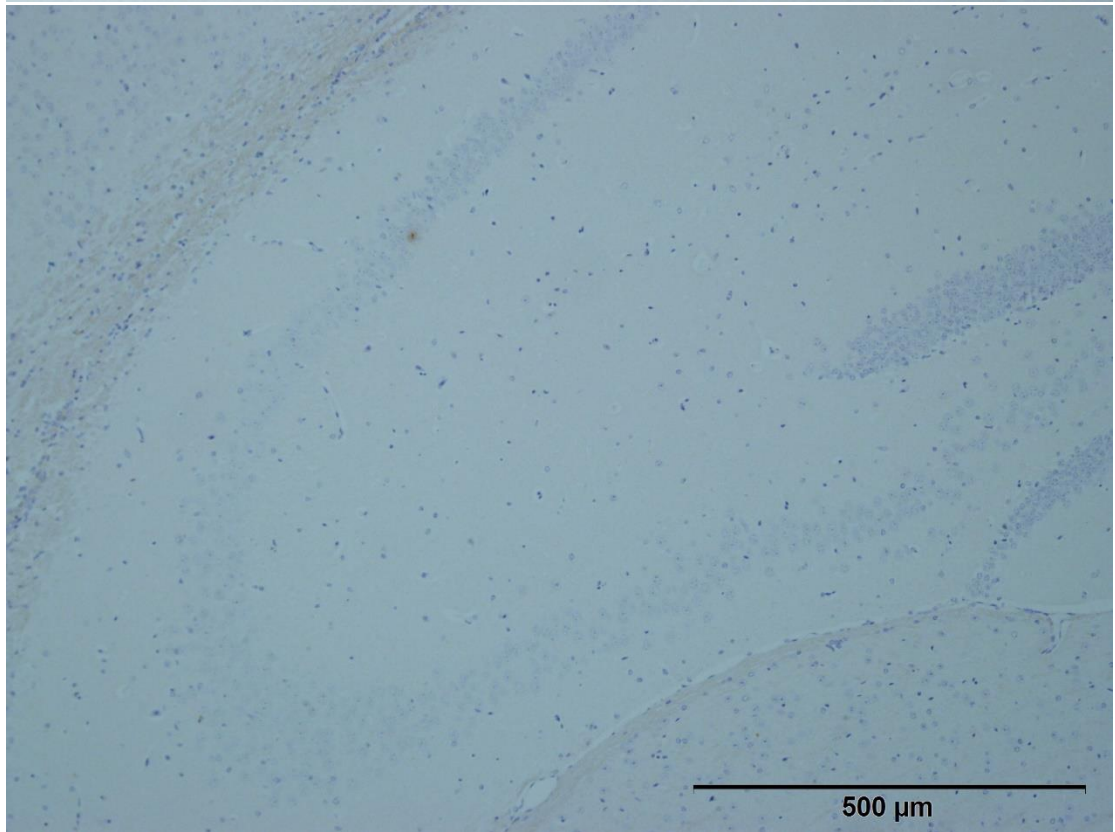

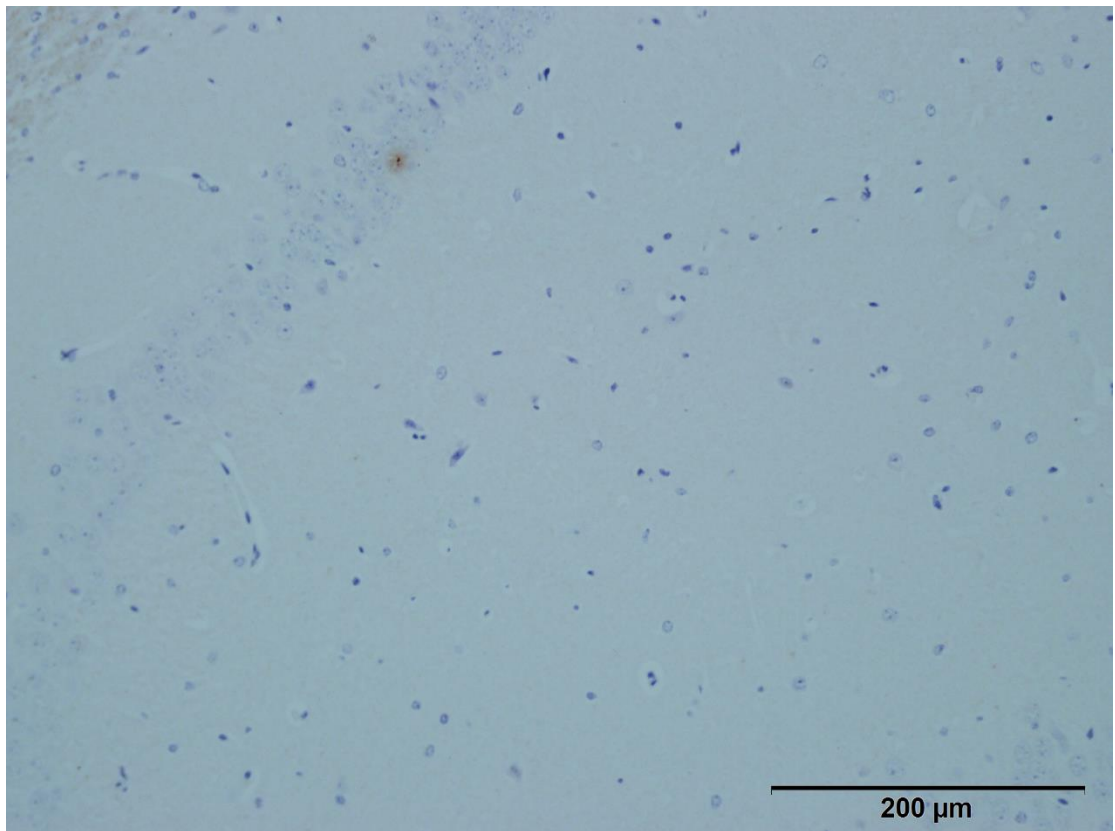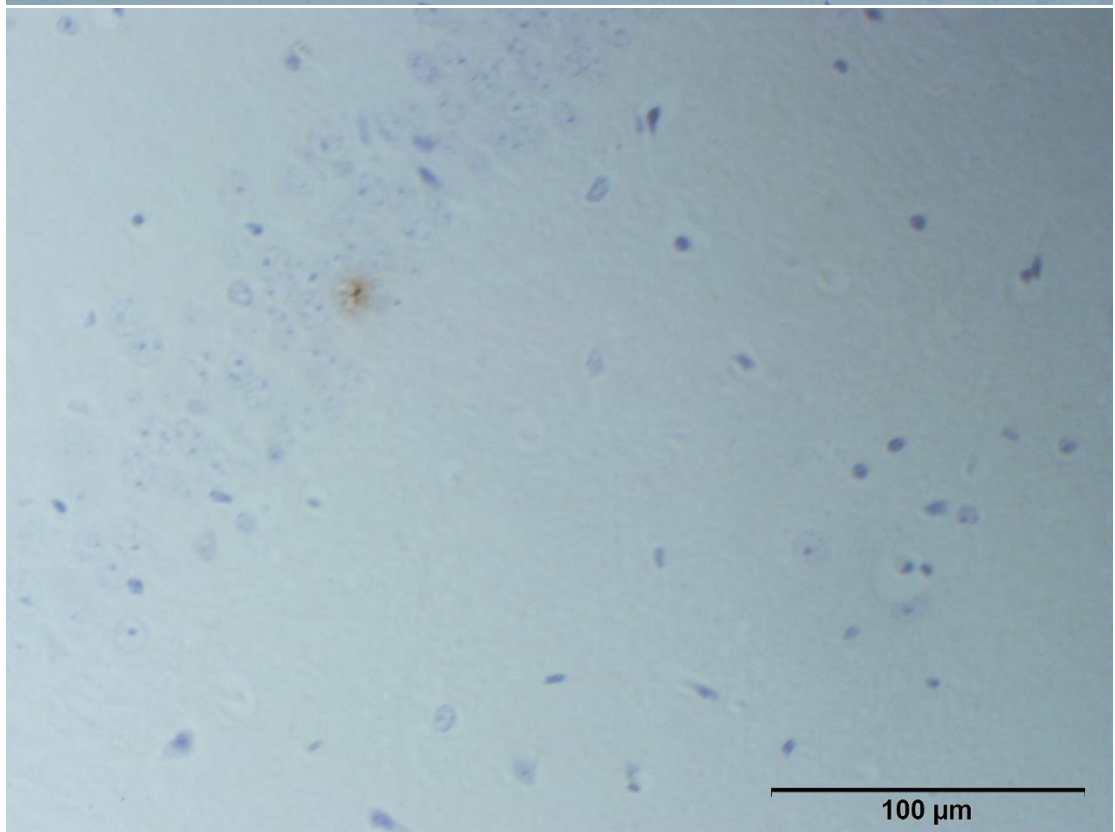

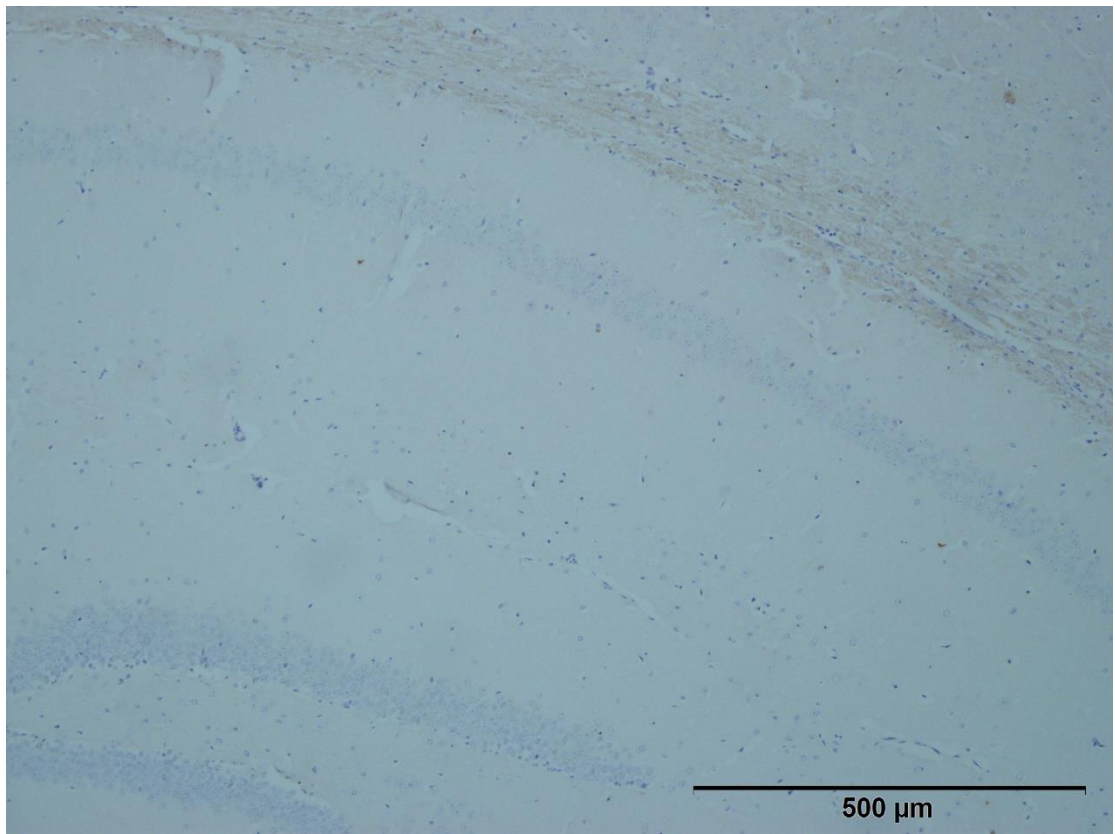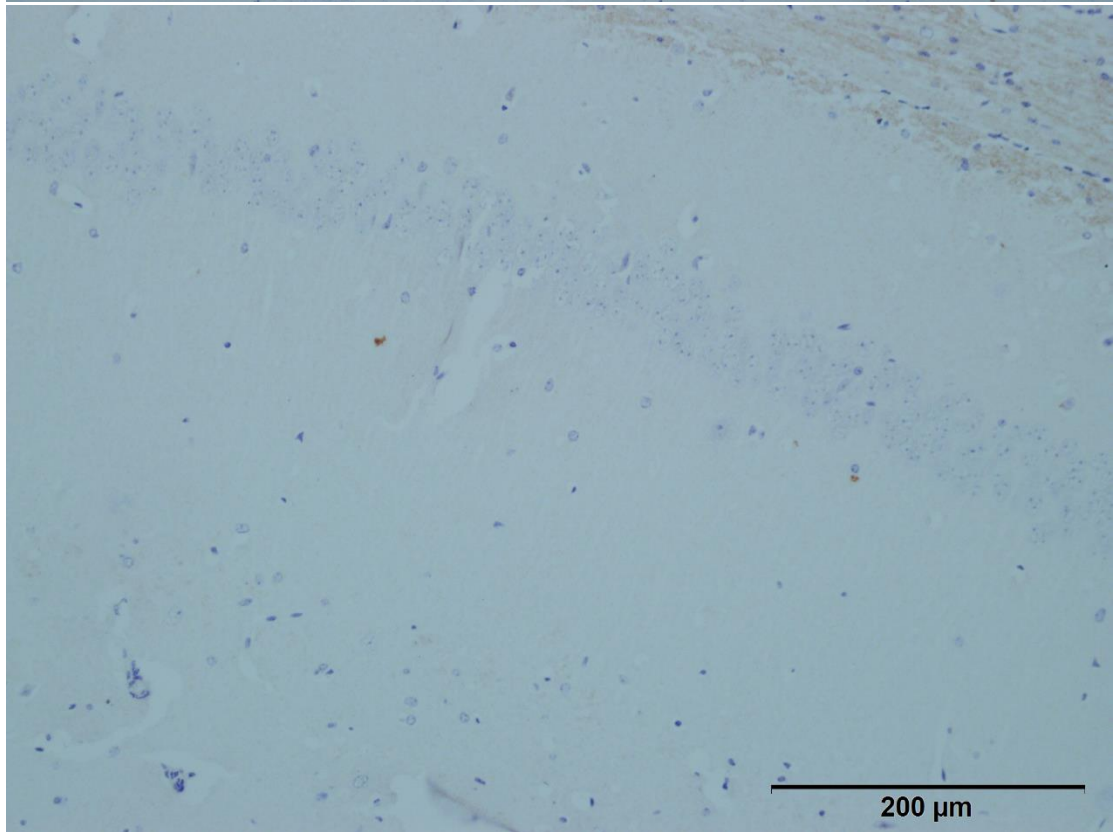

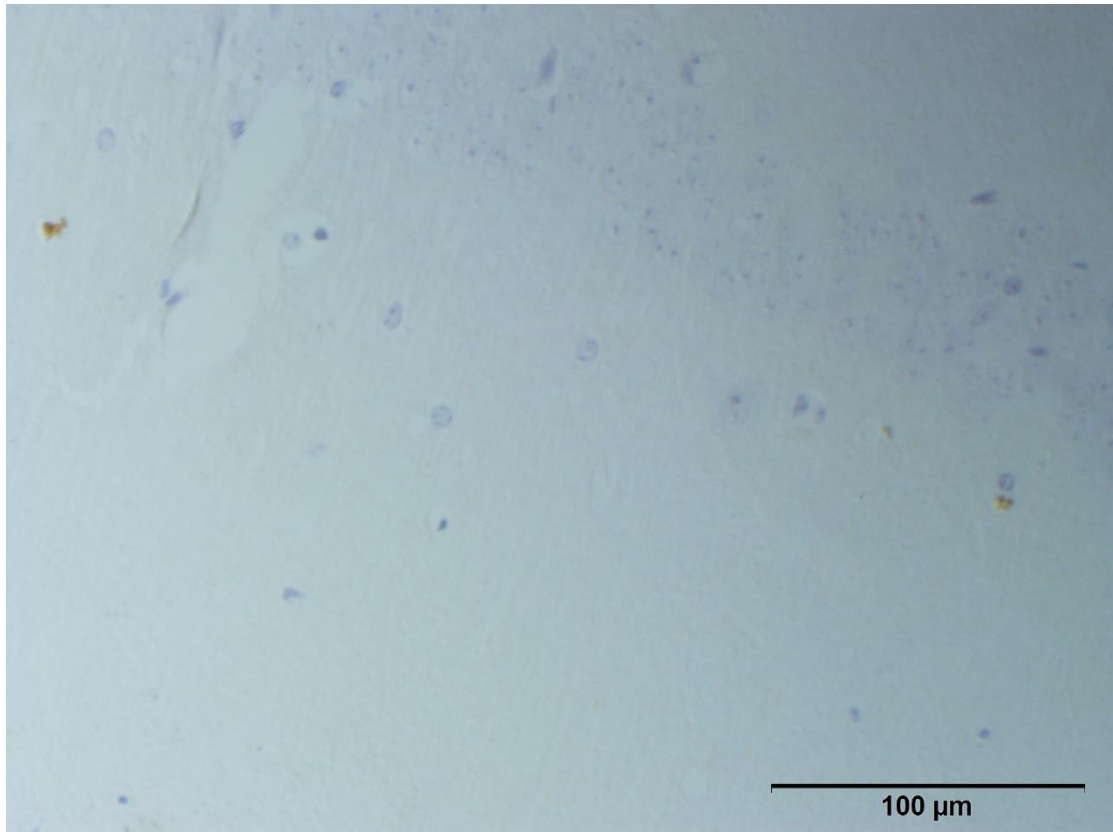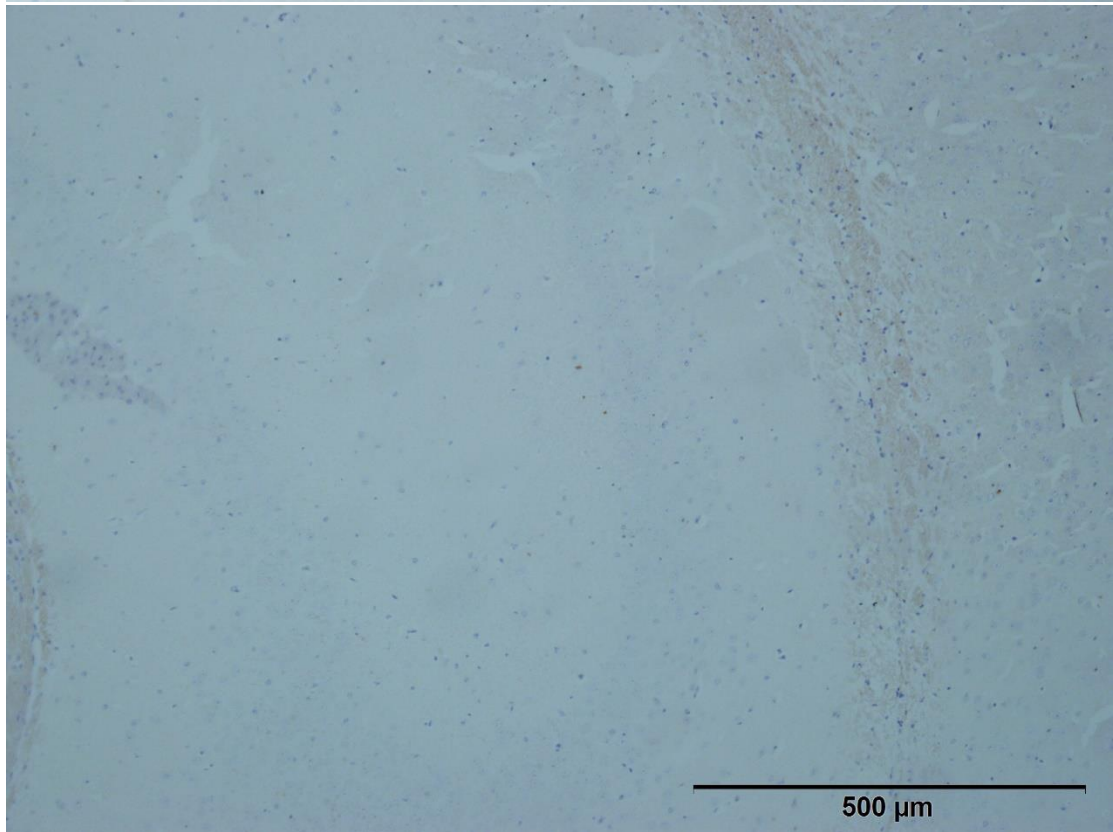

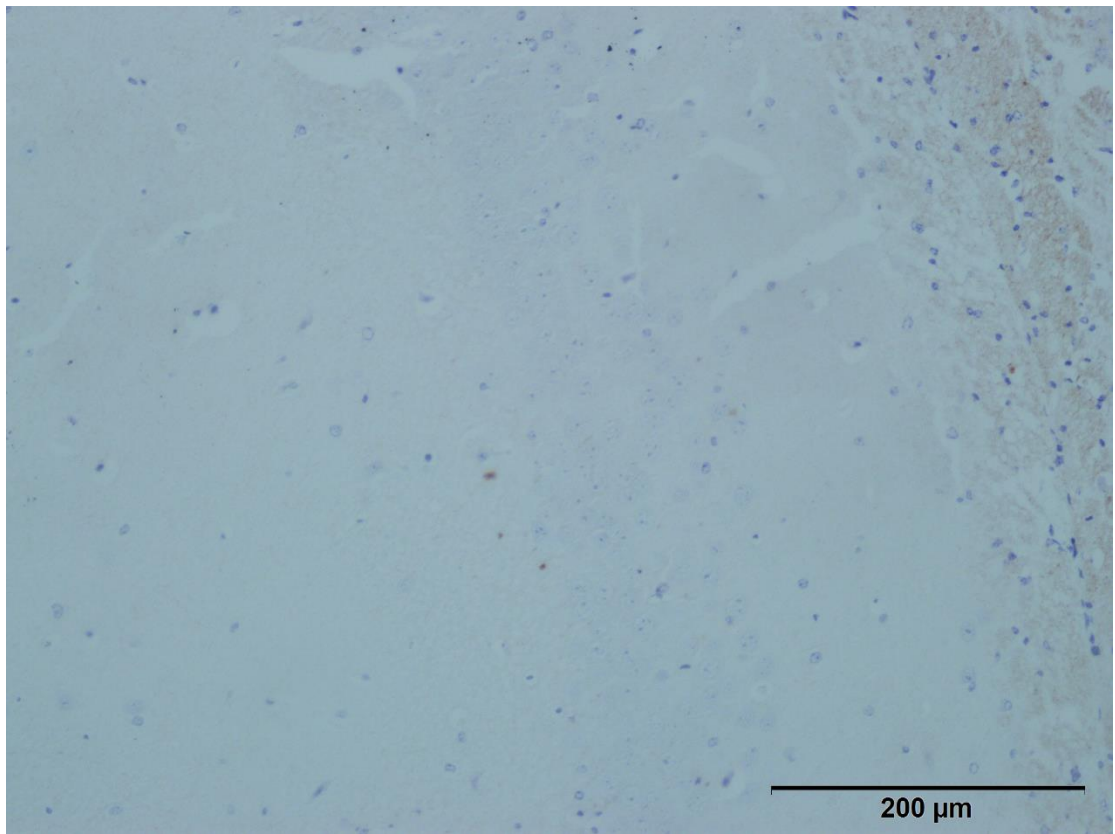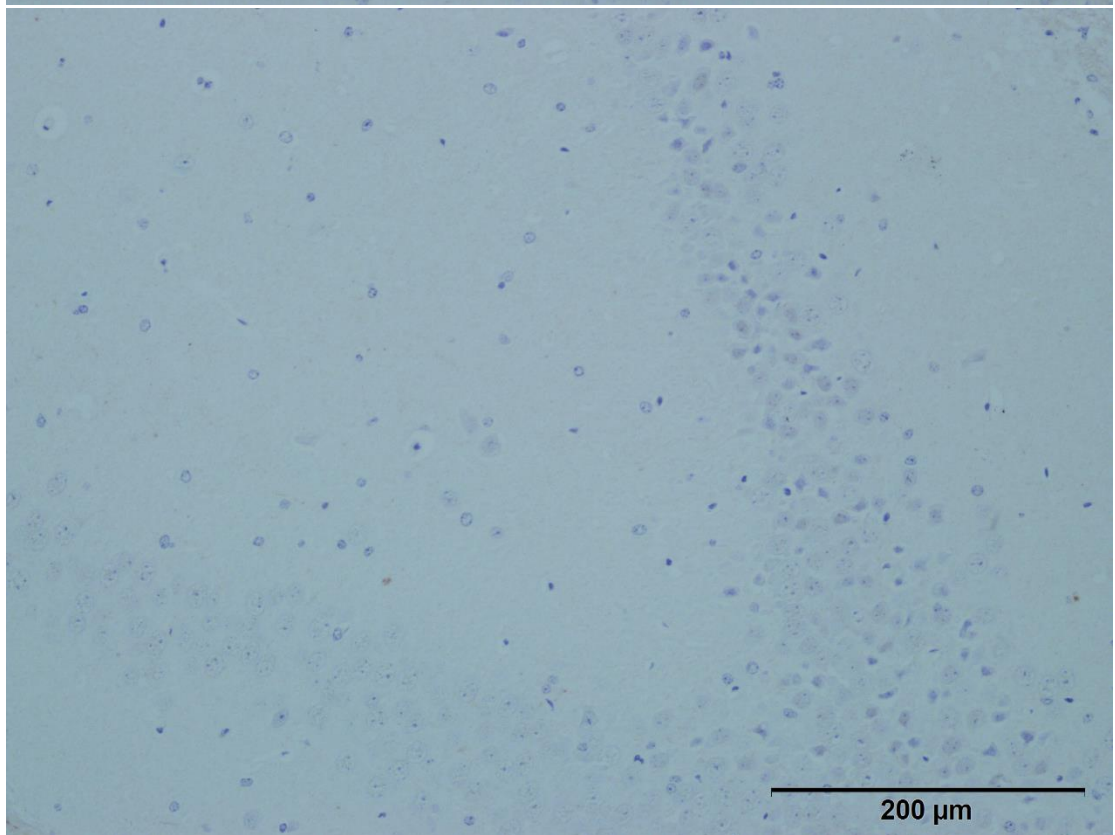

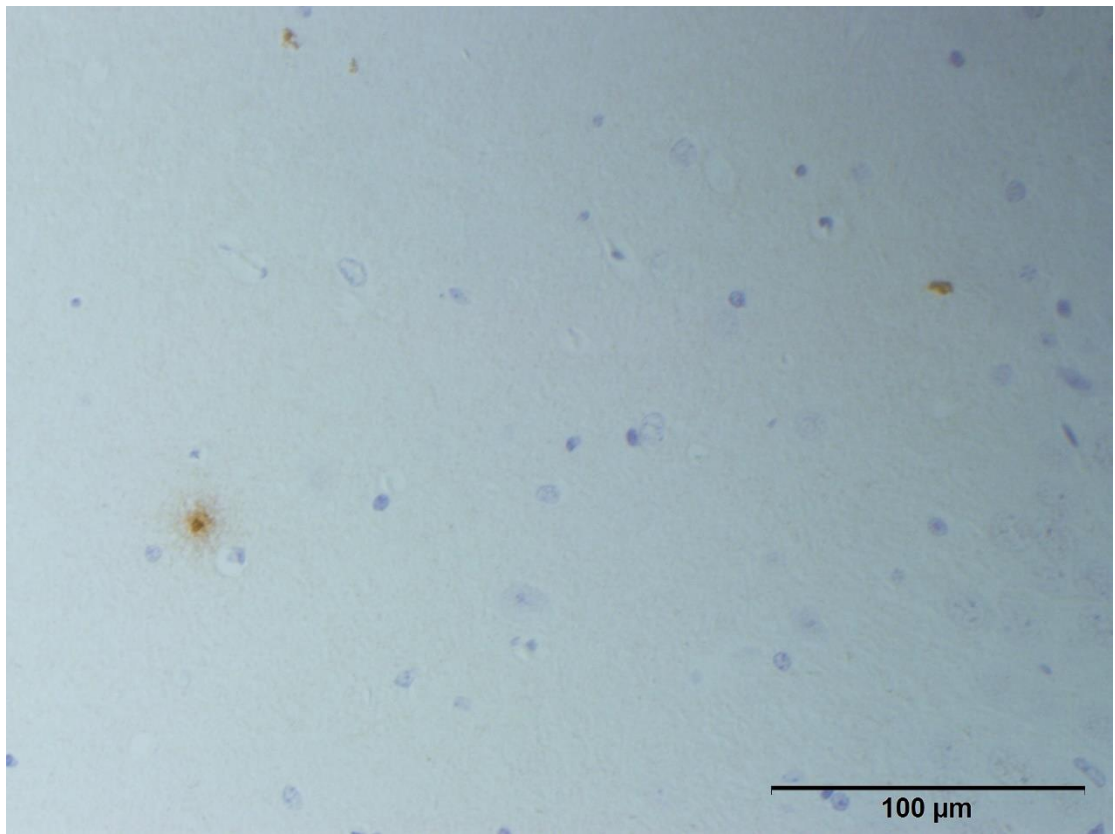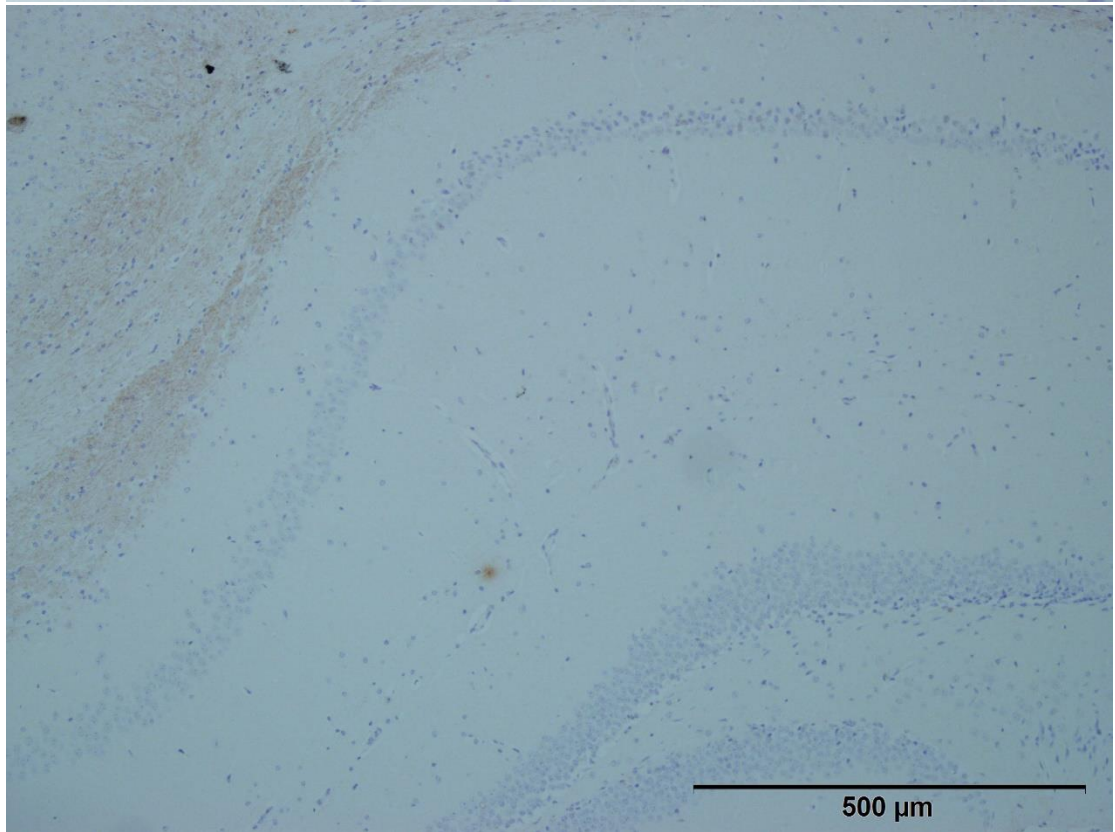

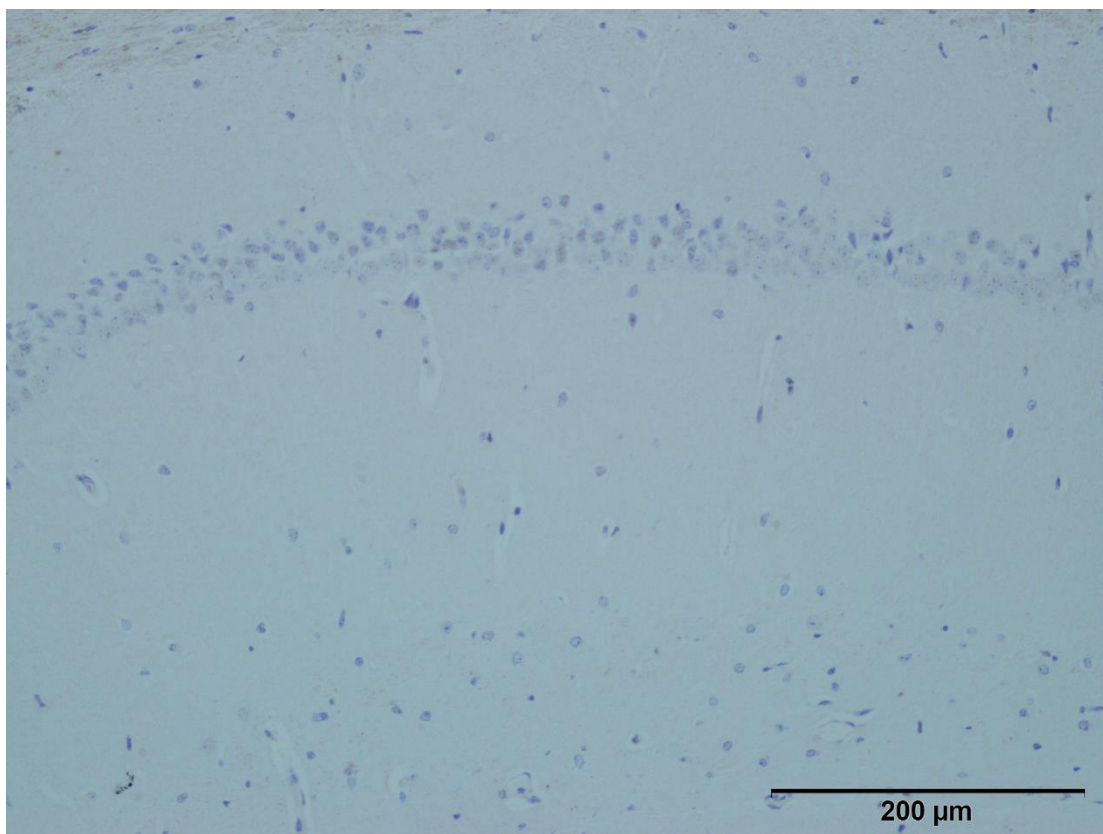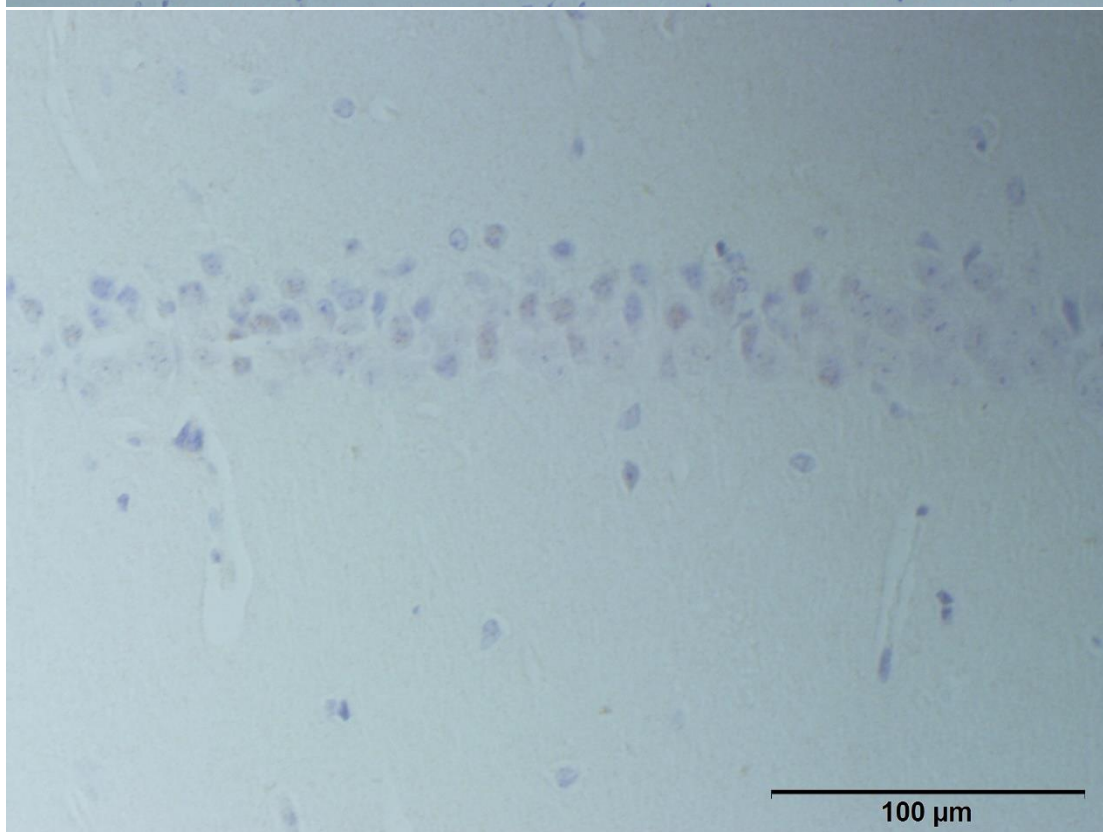

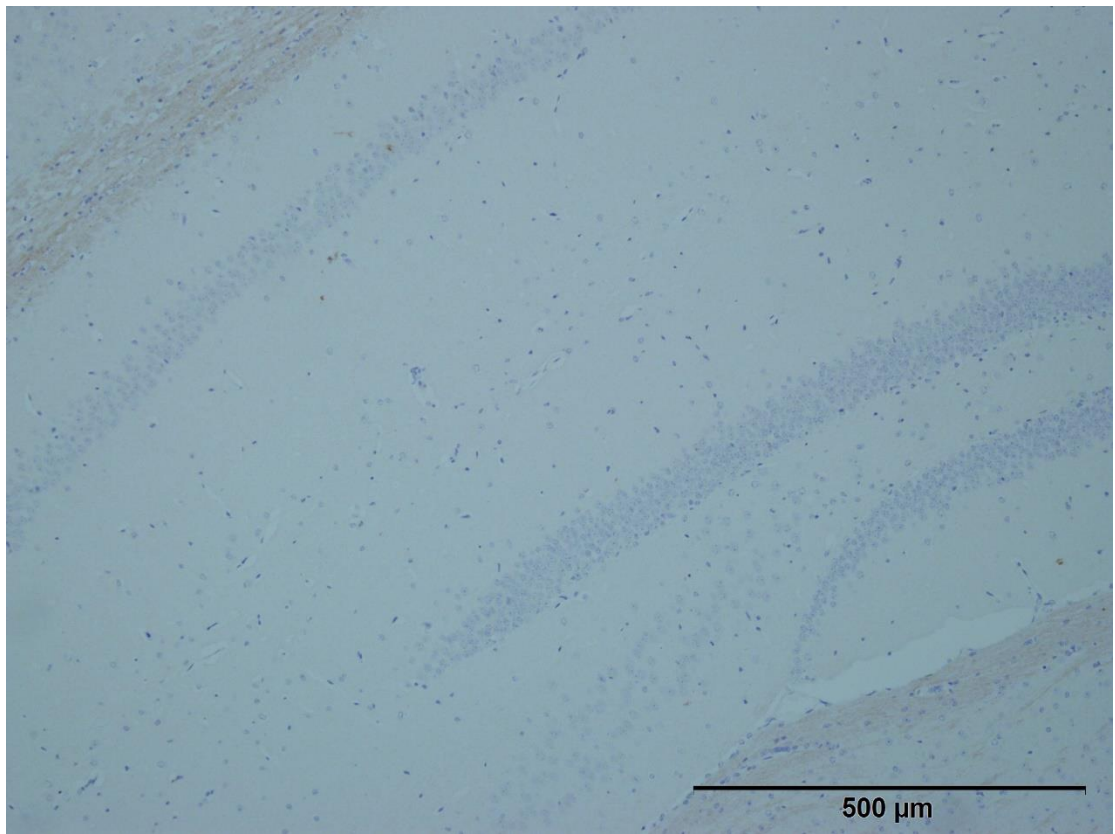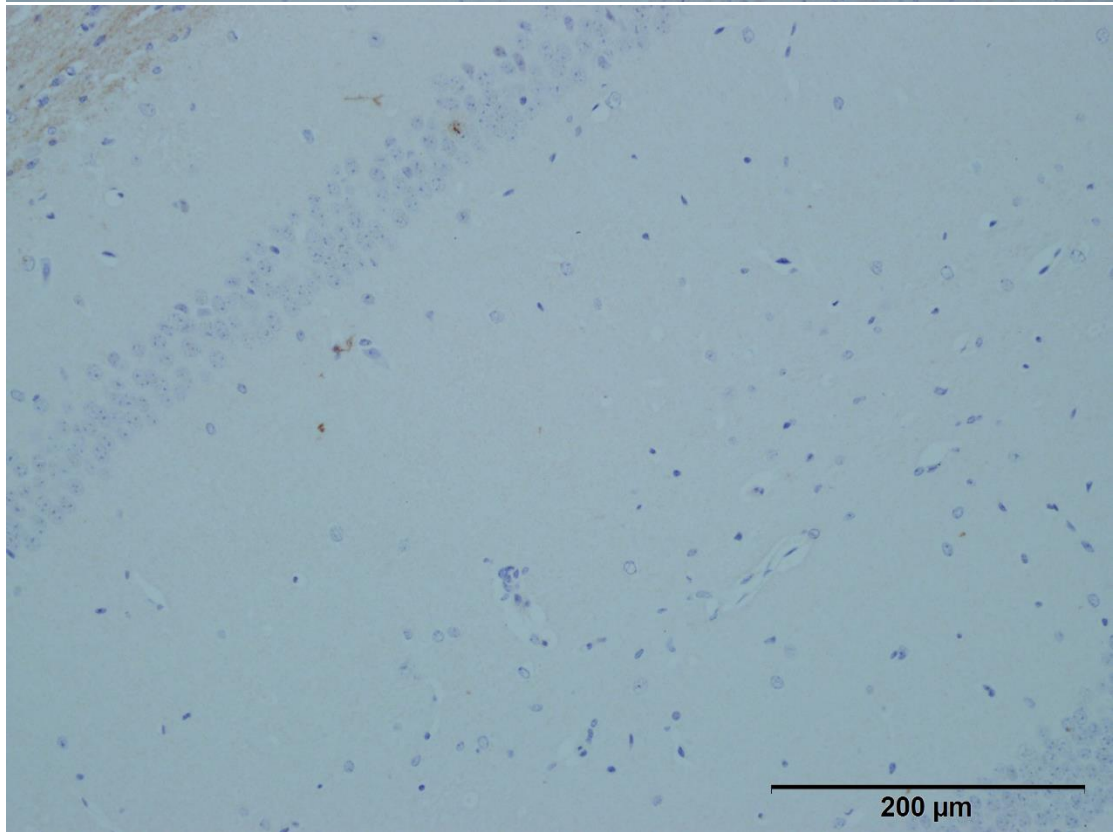

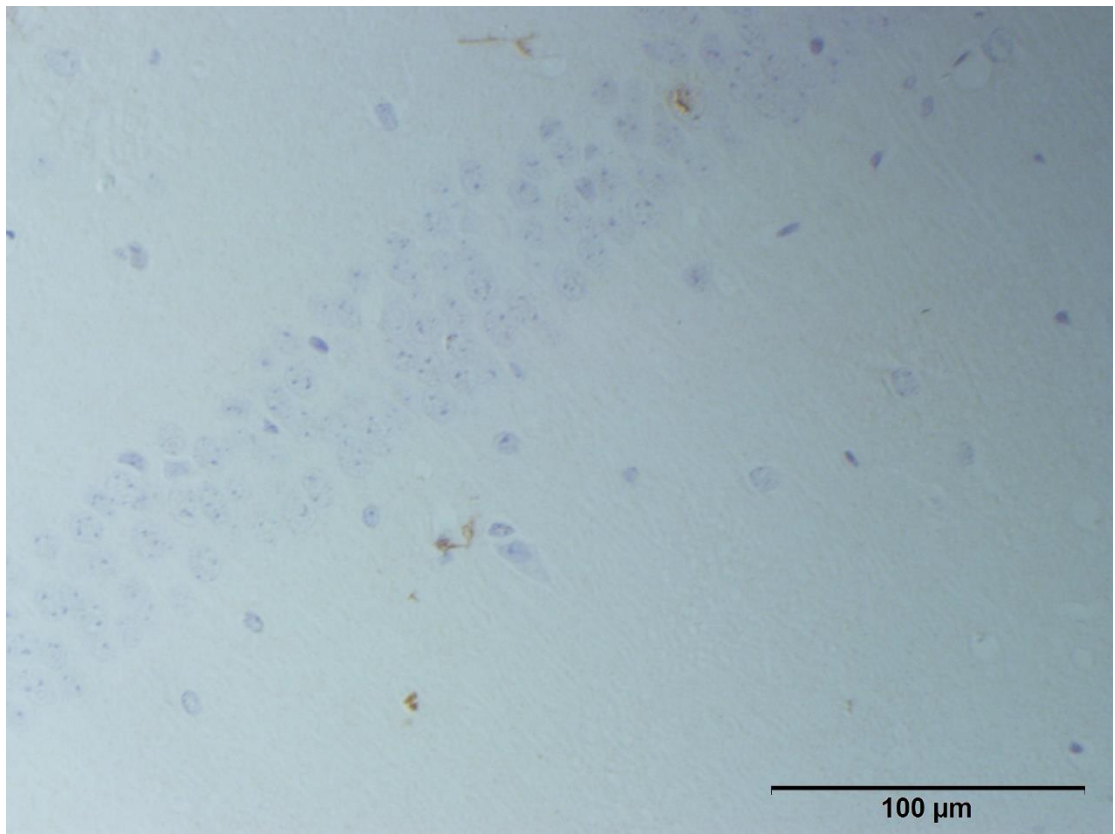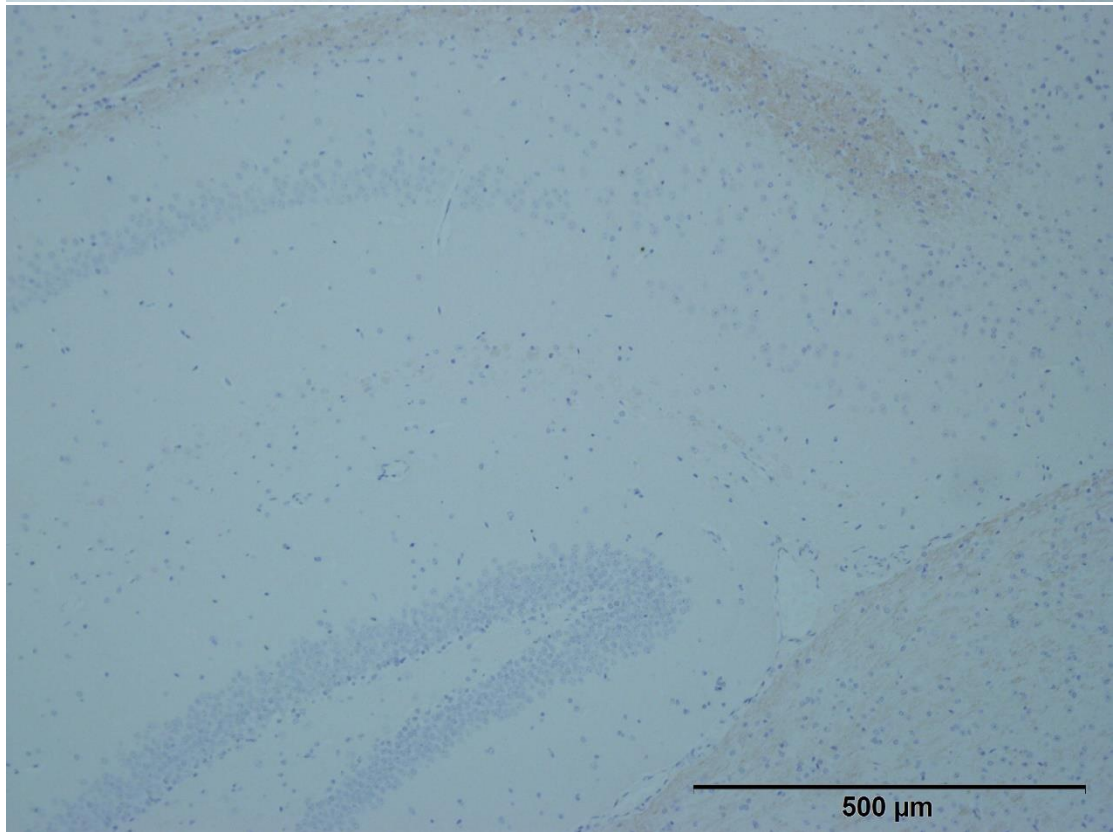

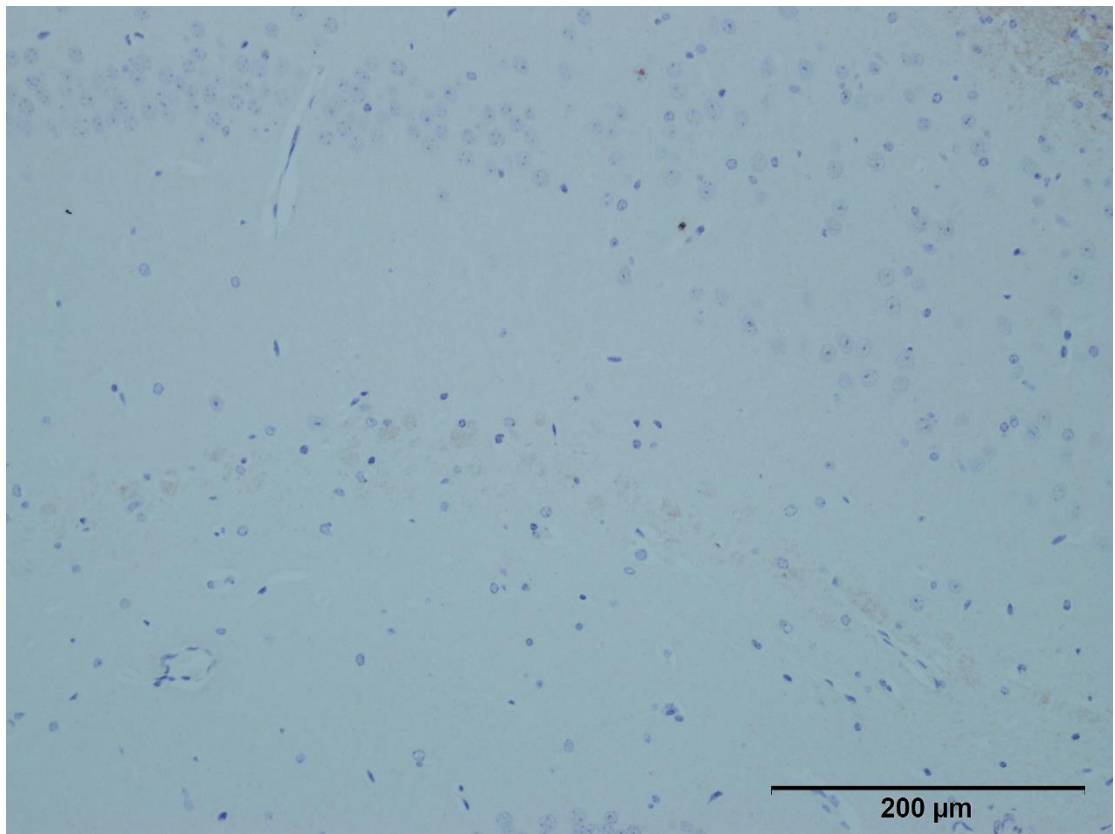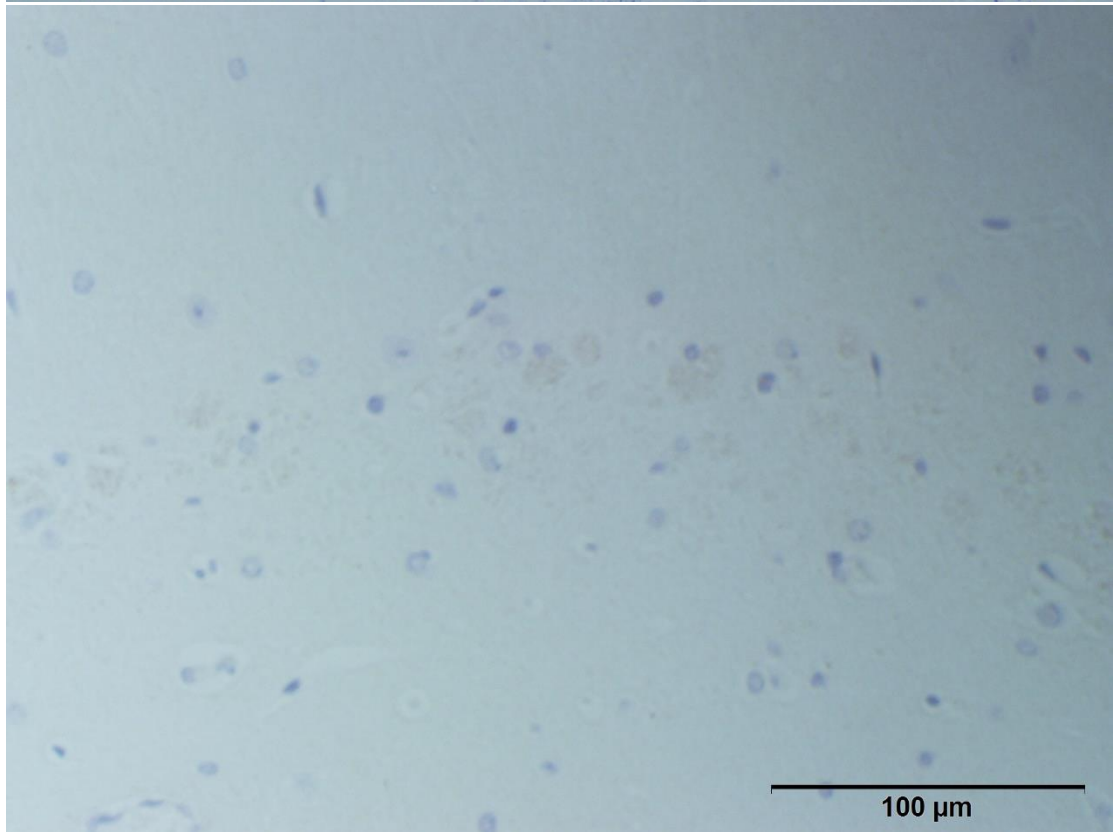

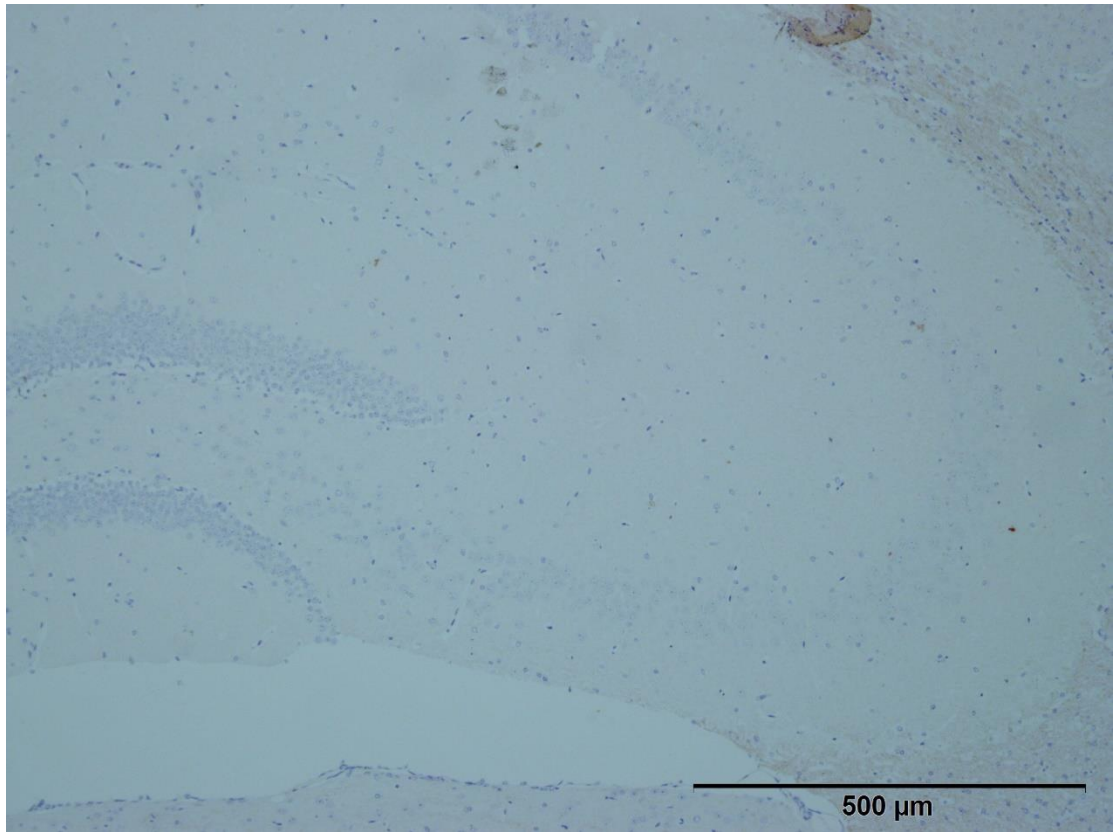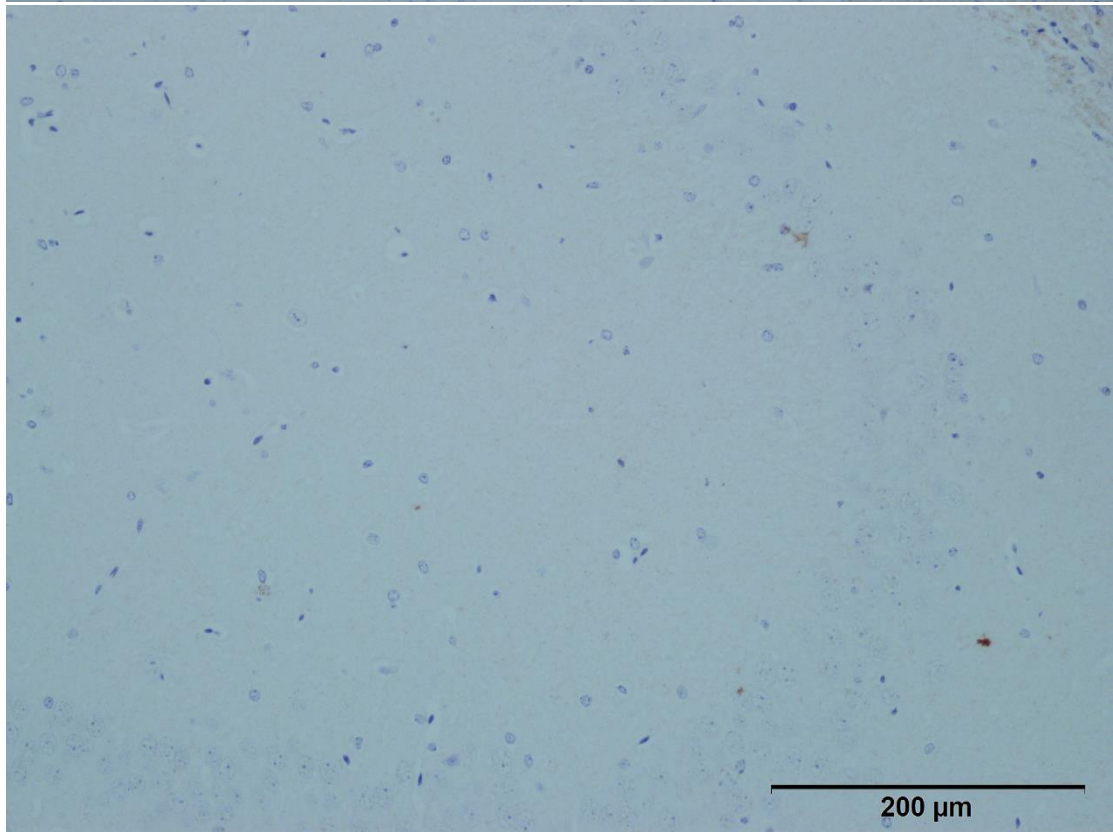

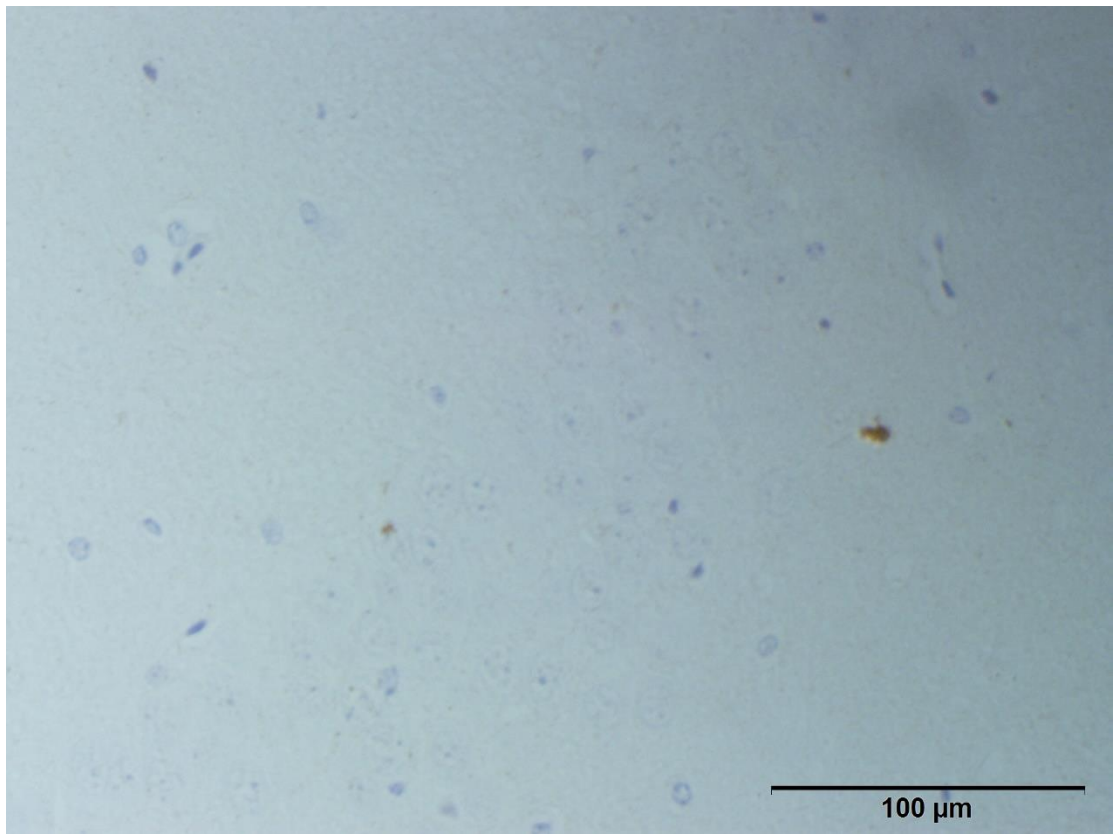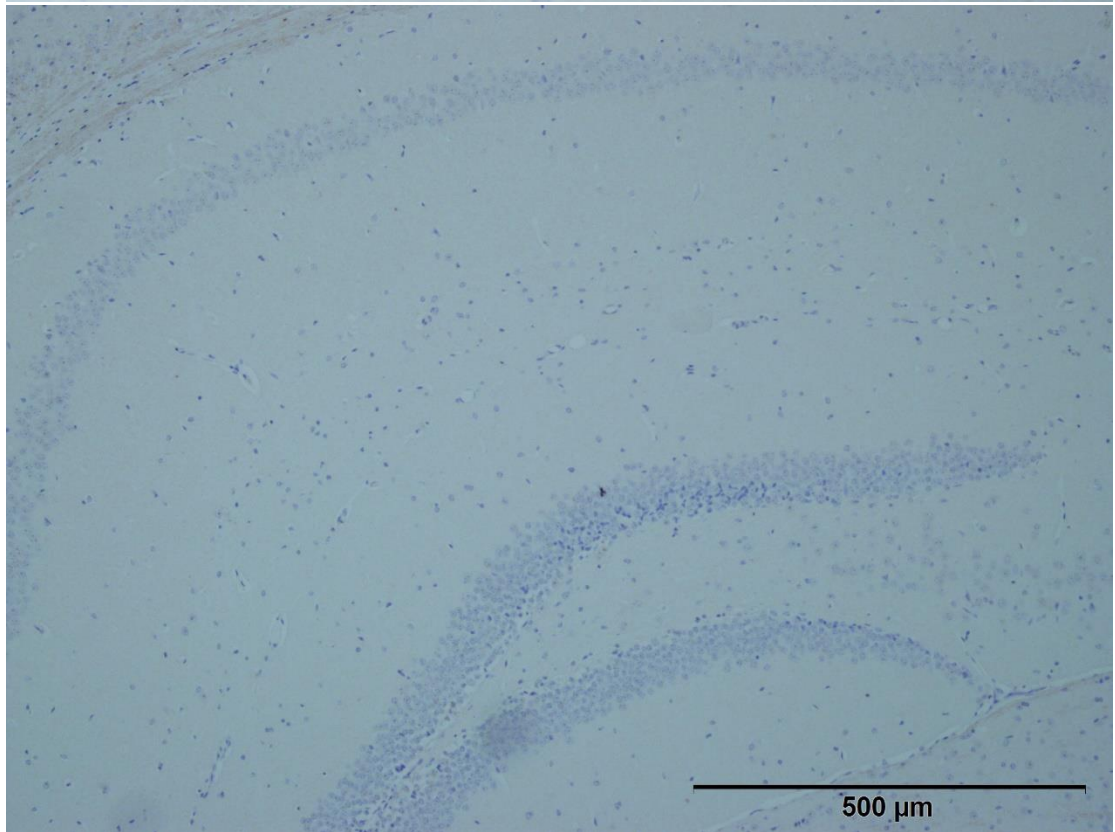

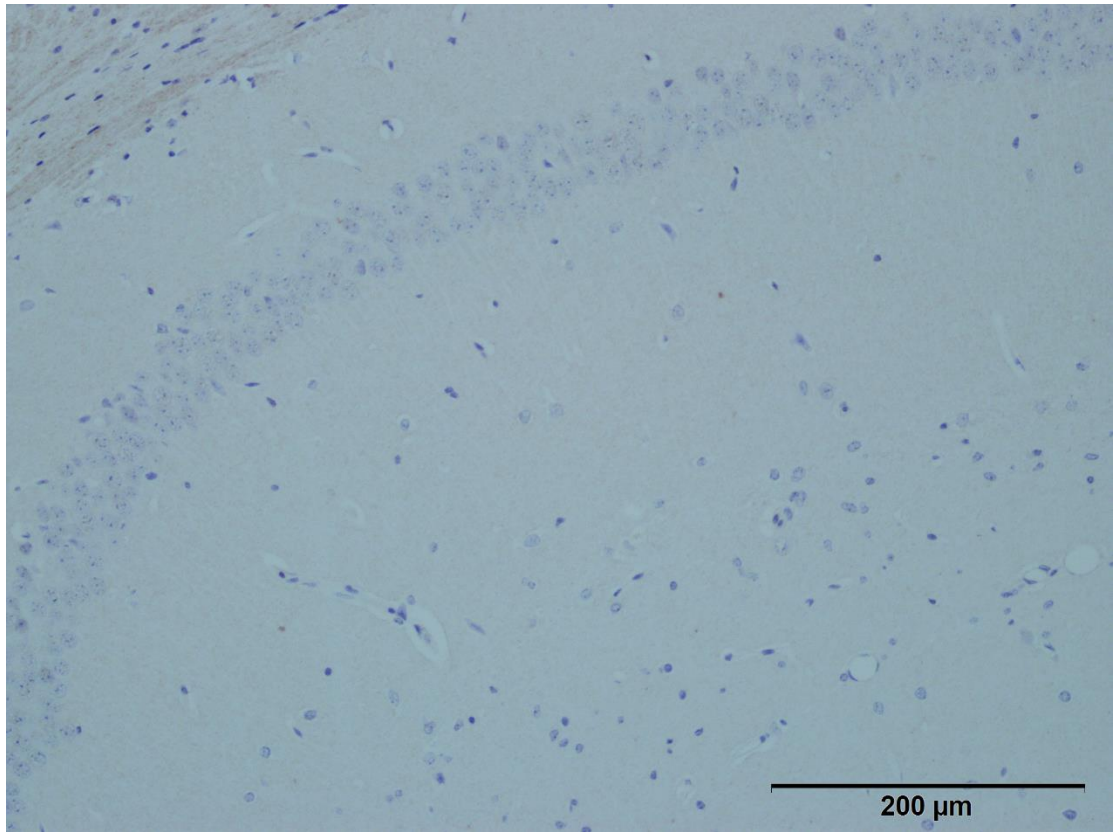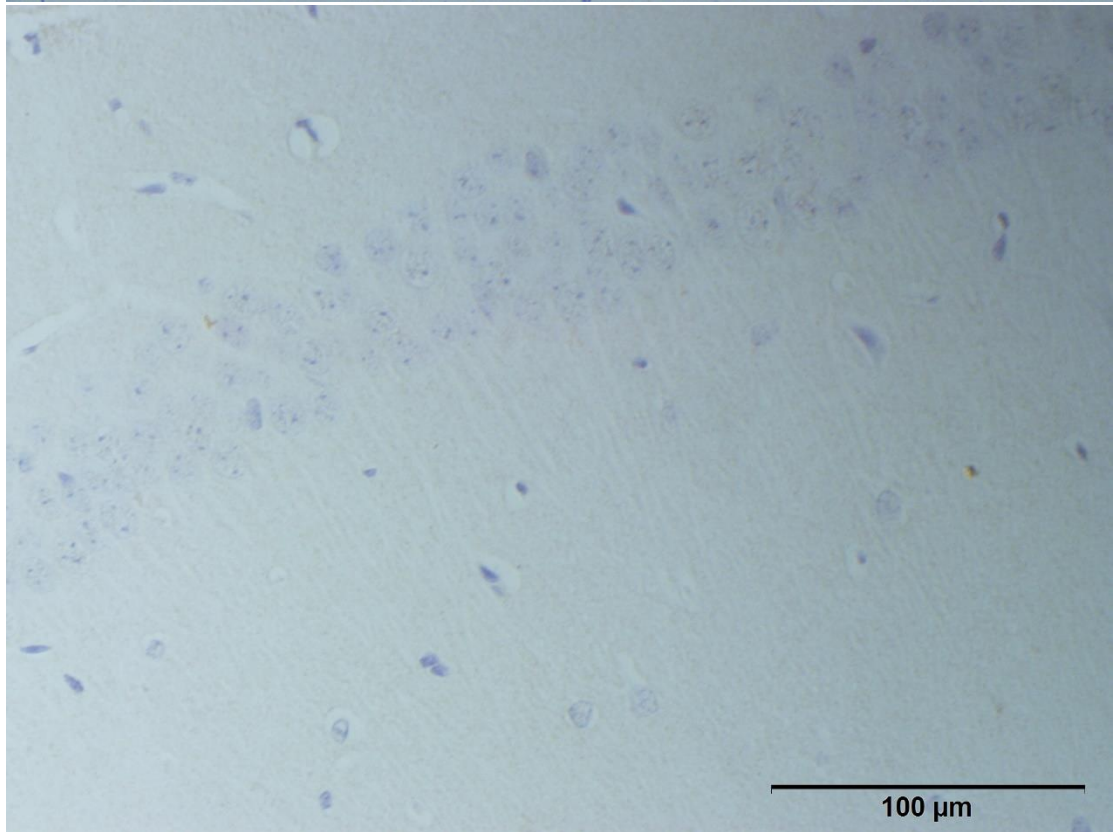

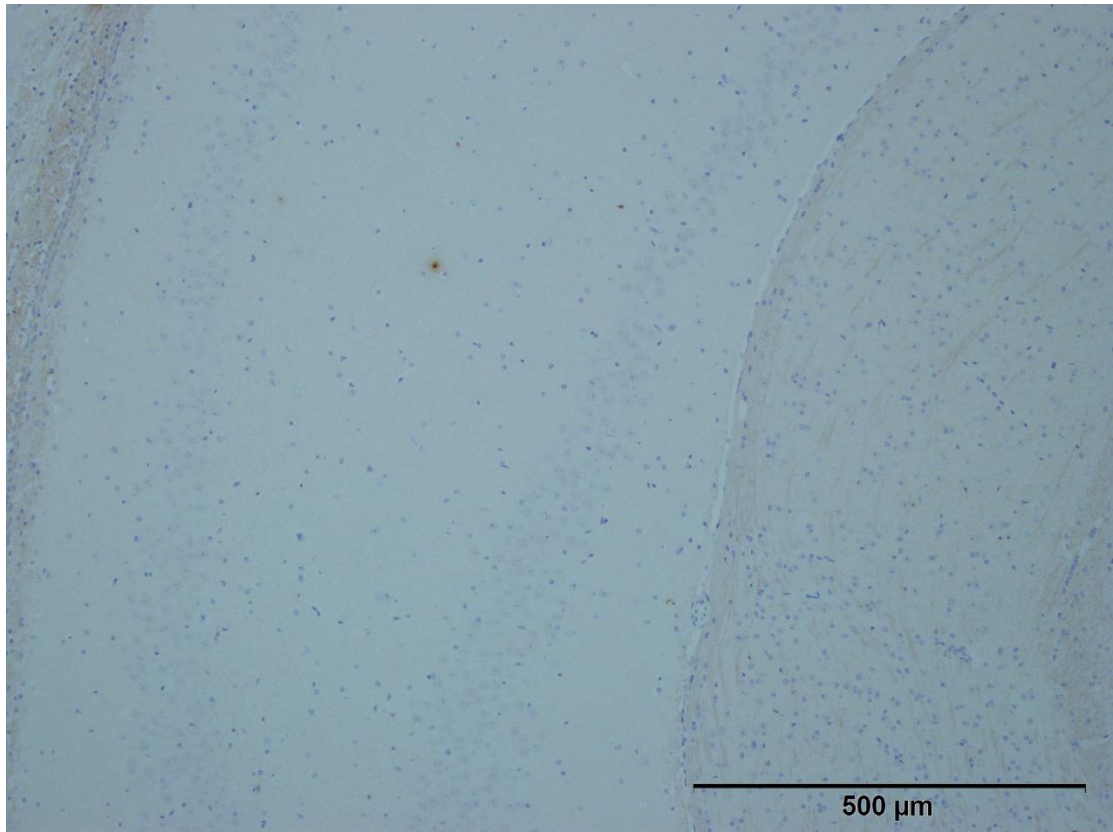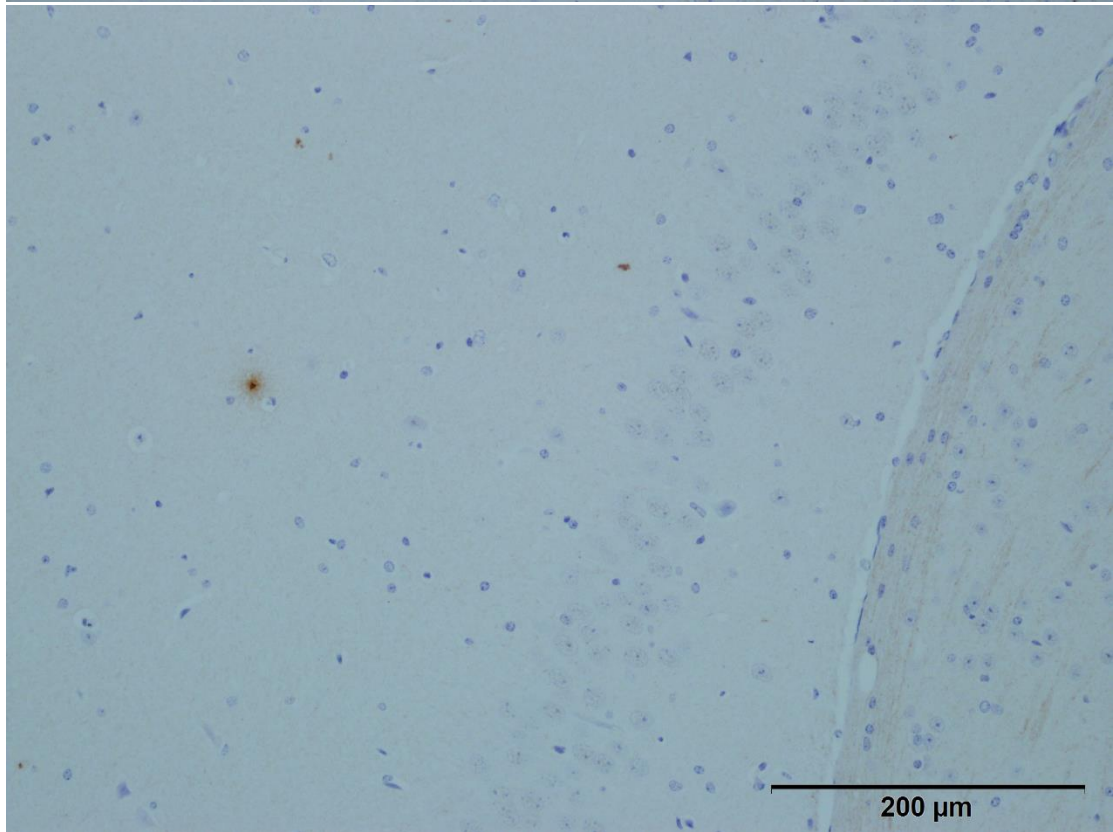

Supplement: Supplementary file 3 [file DataSheet6.PDF]

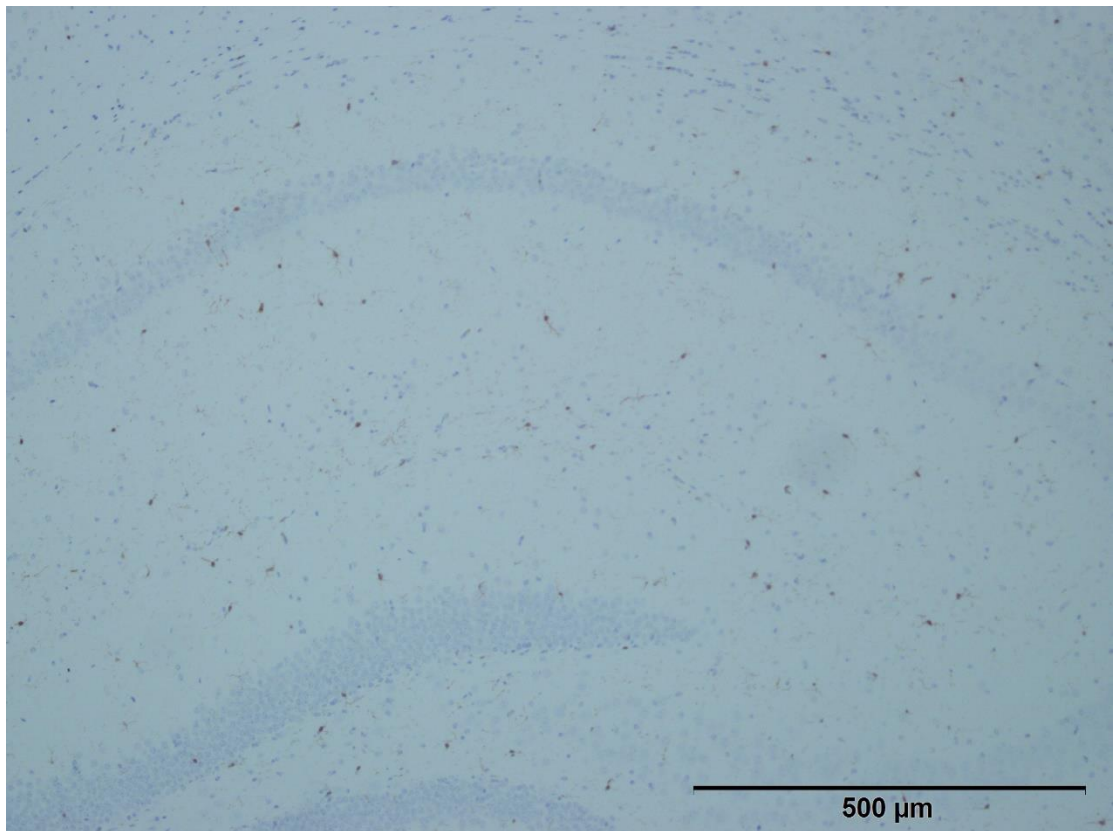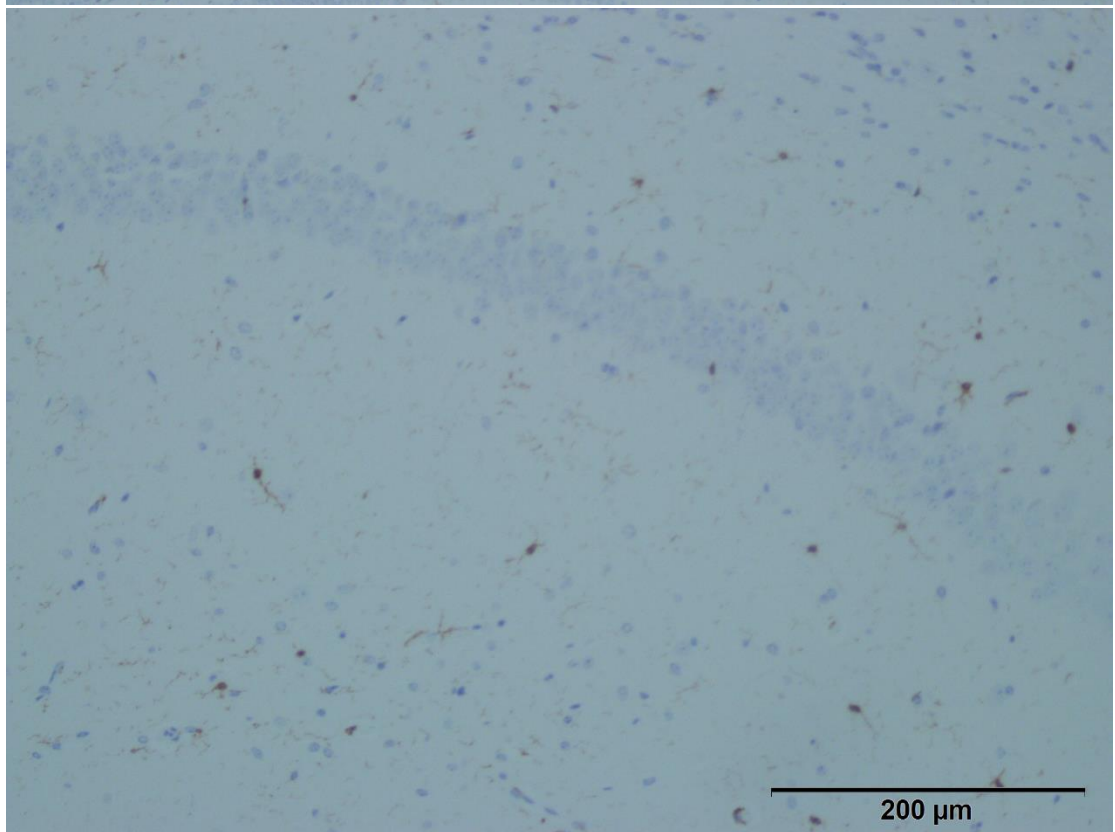

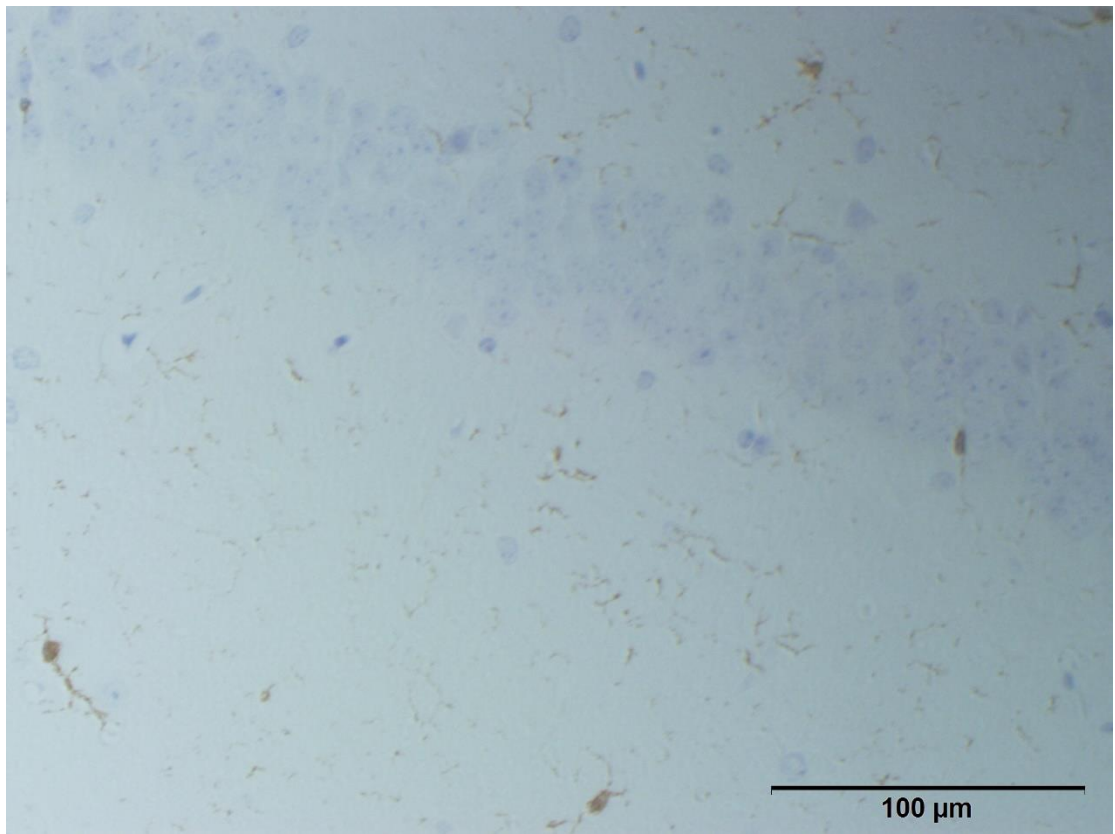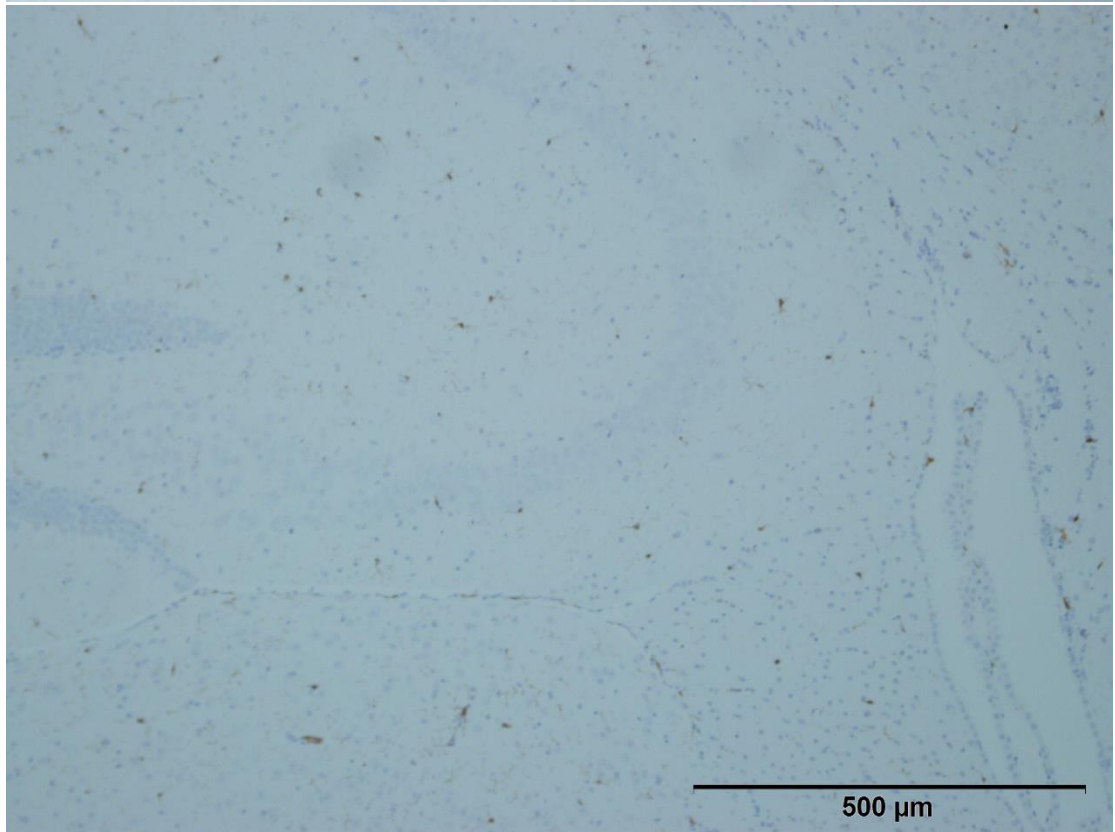

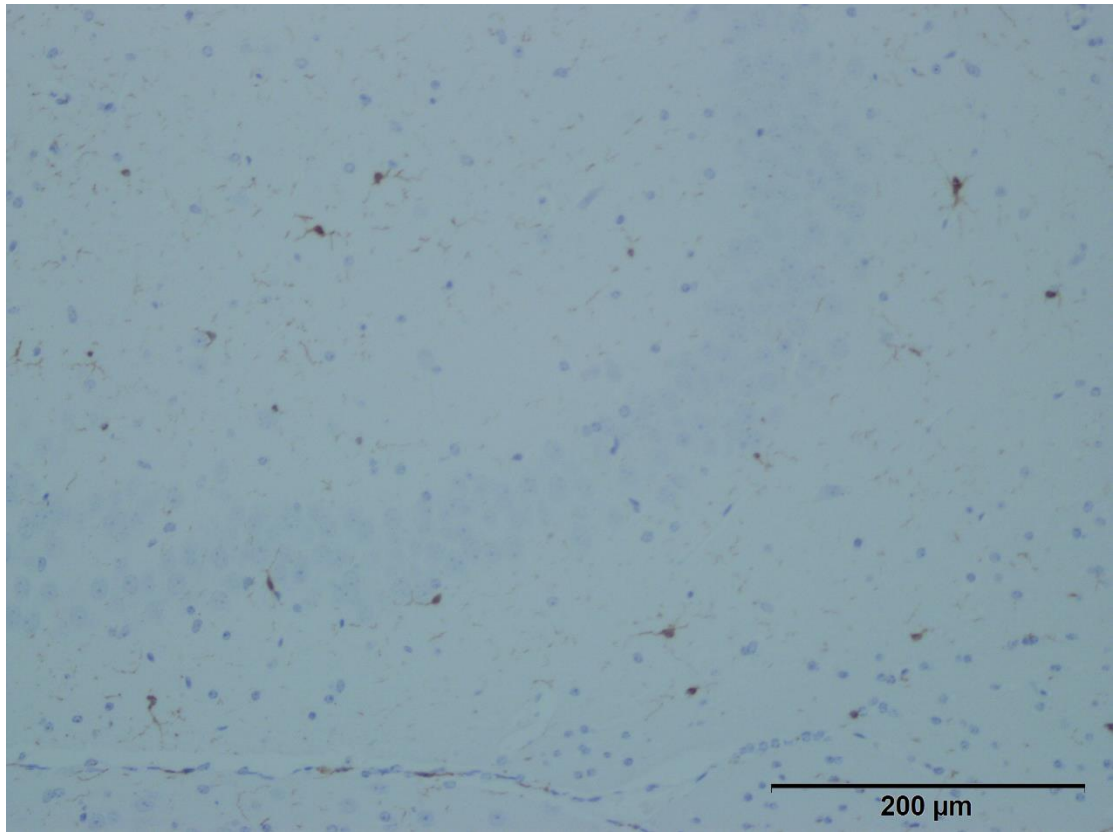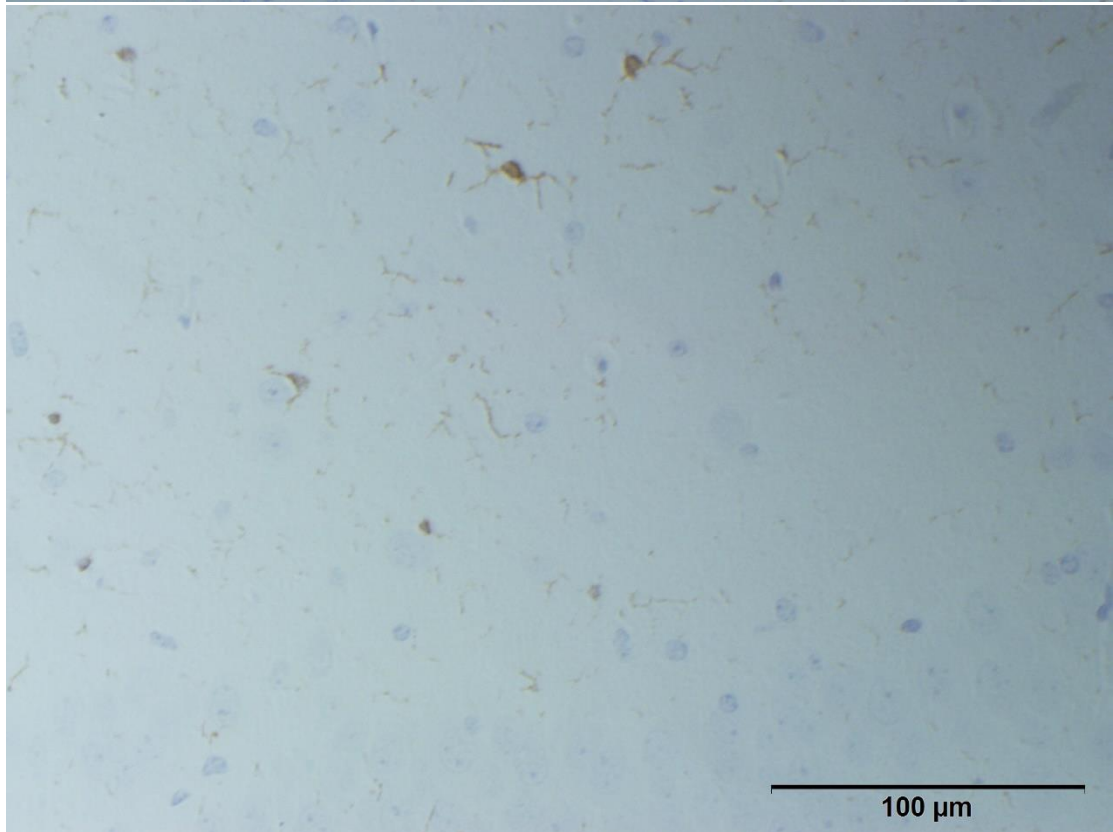

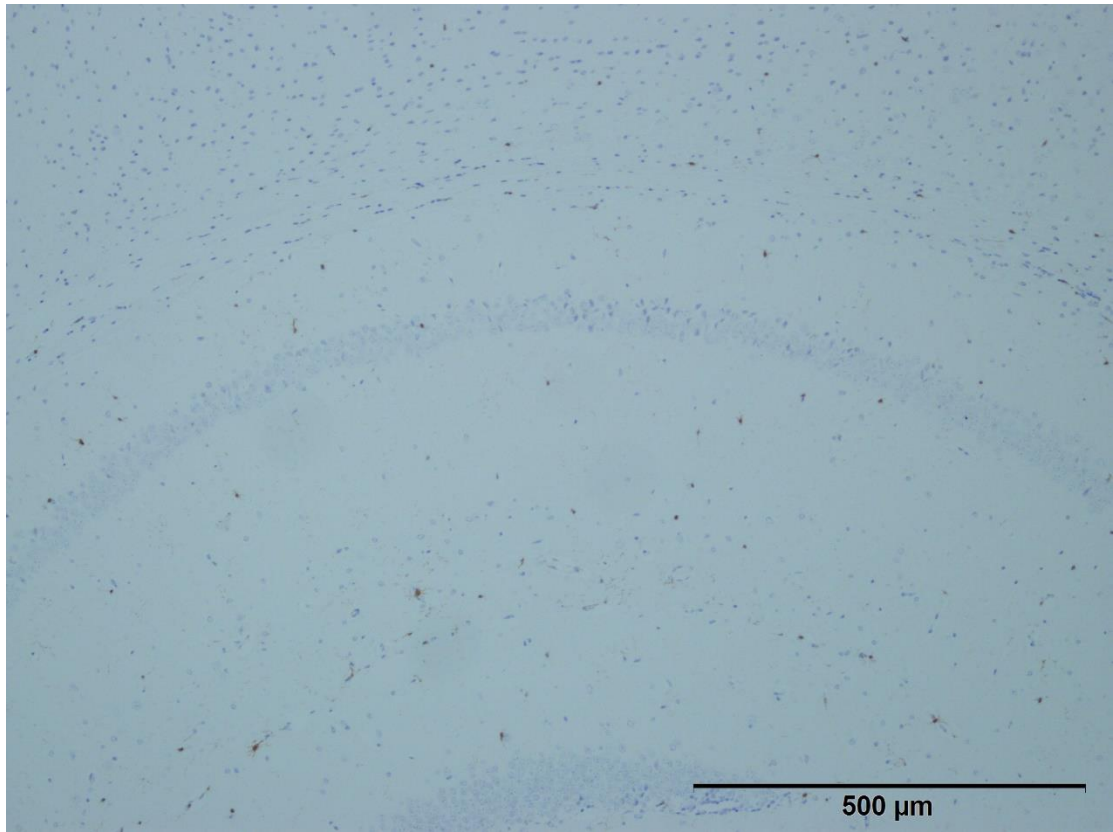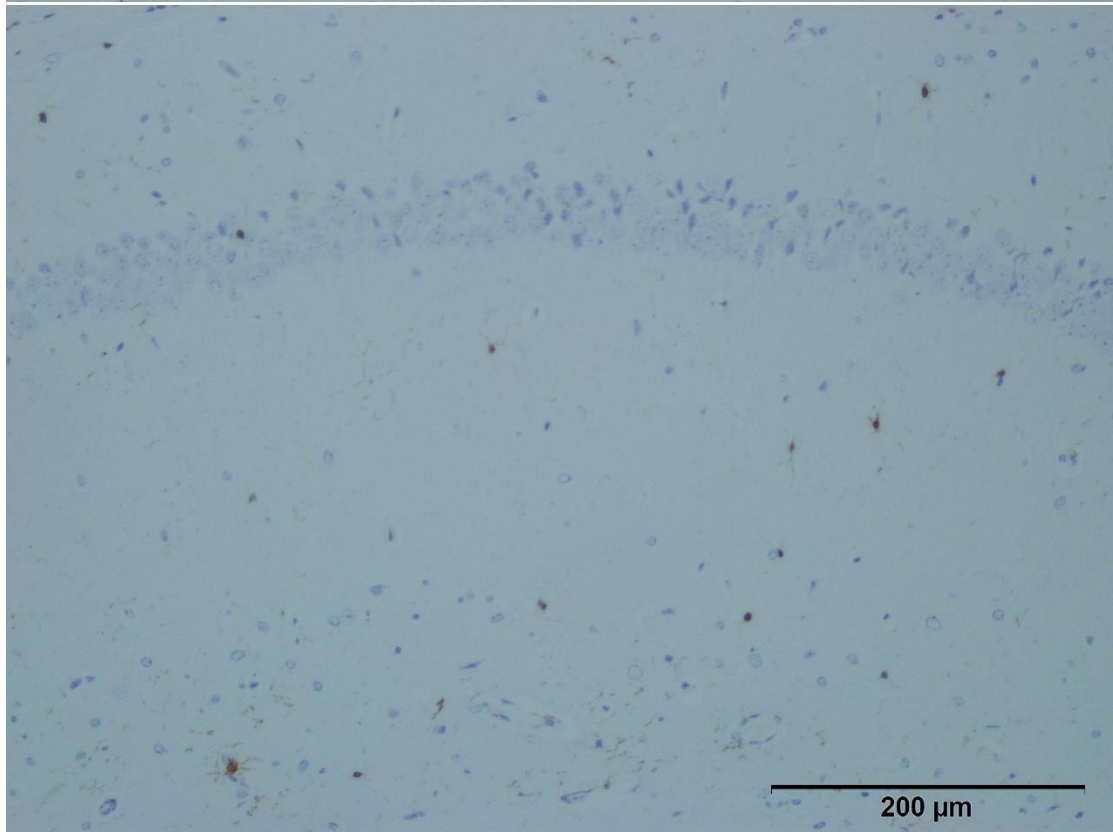

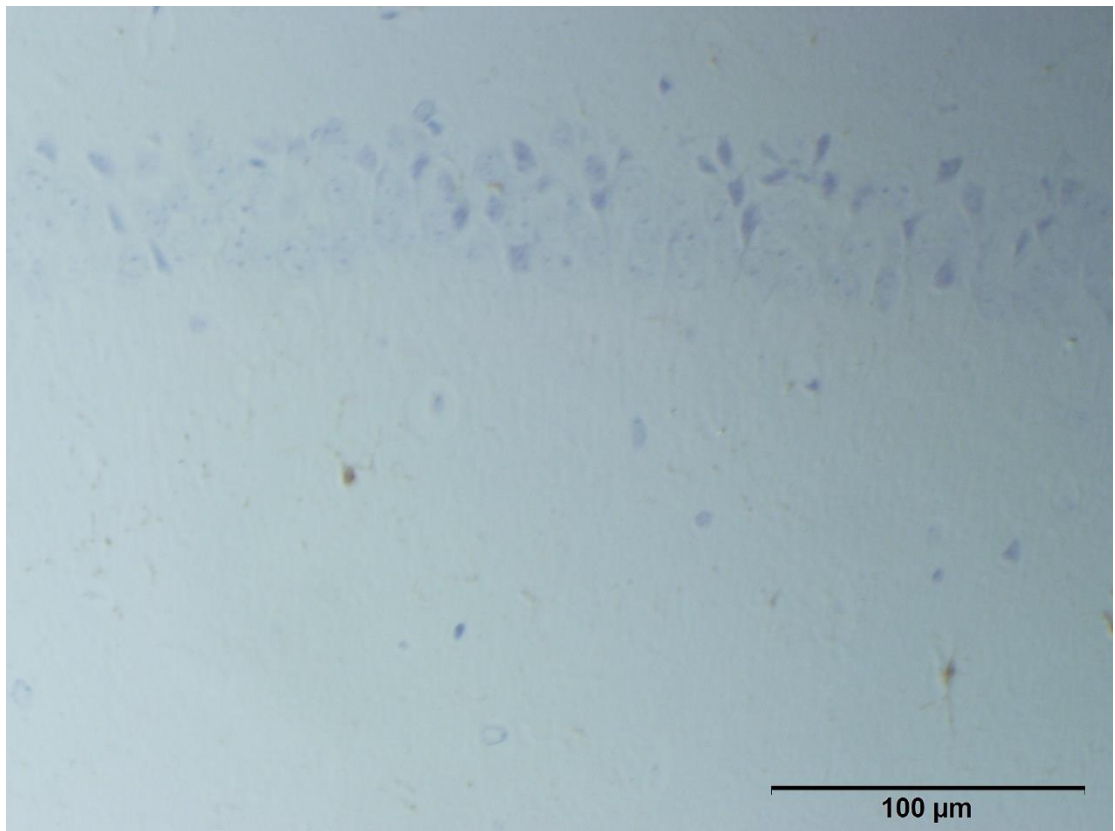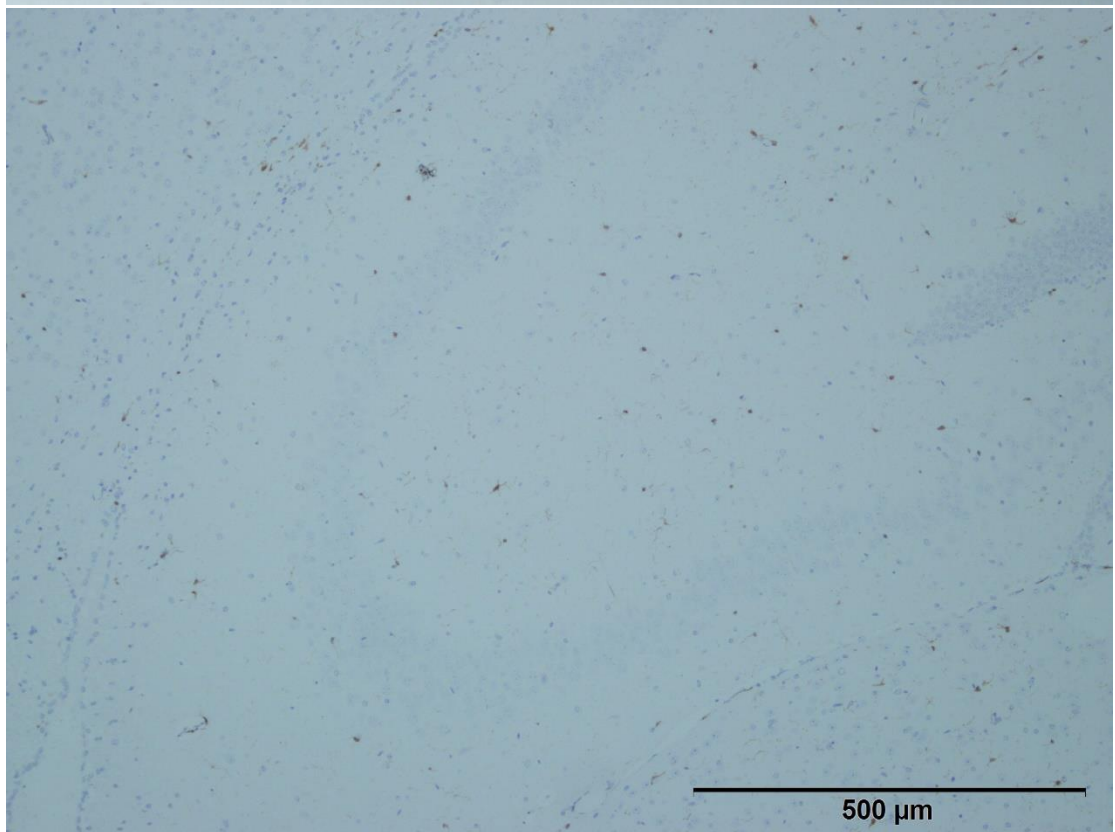

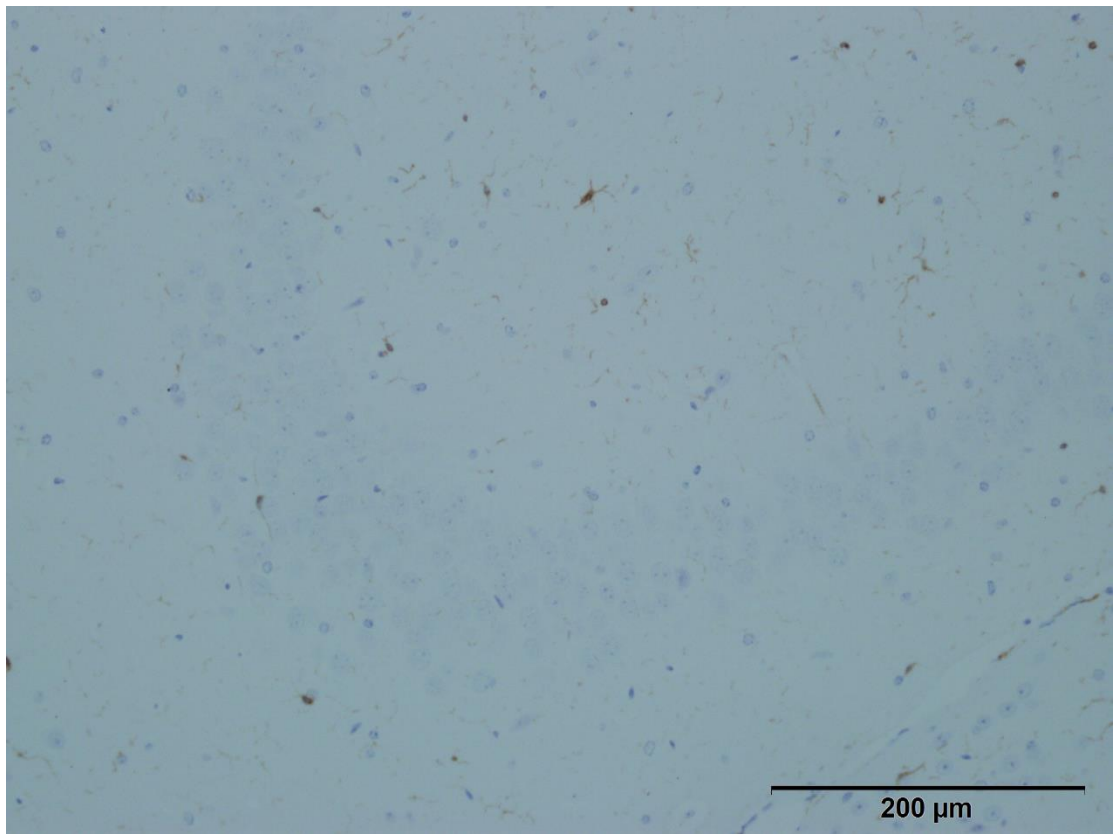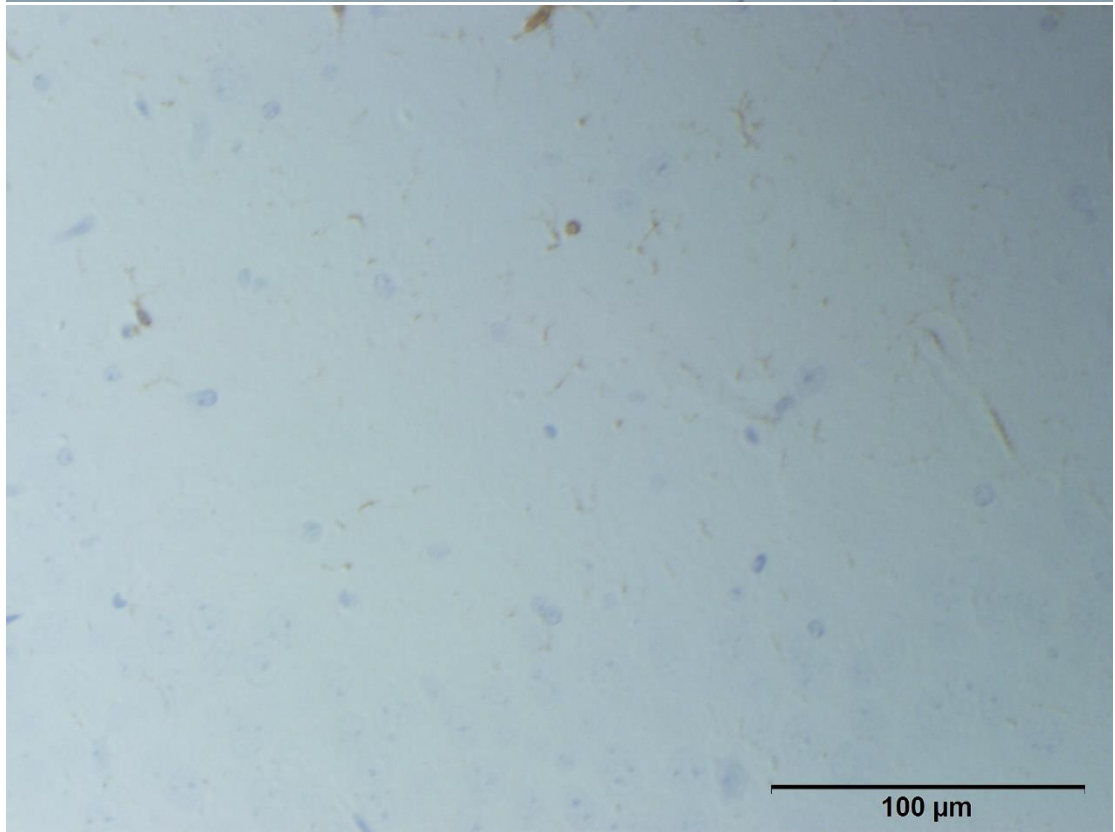

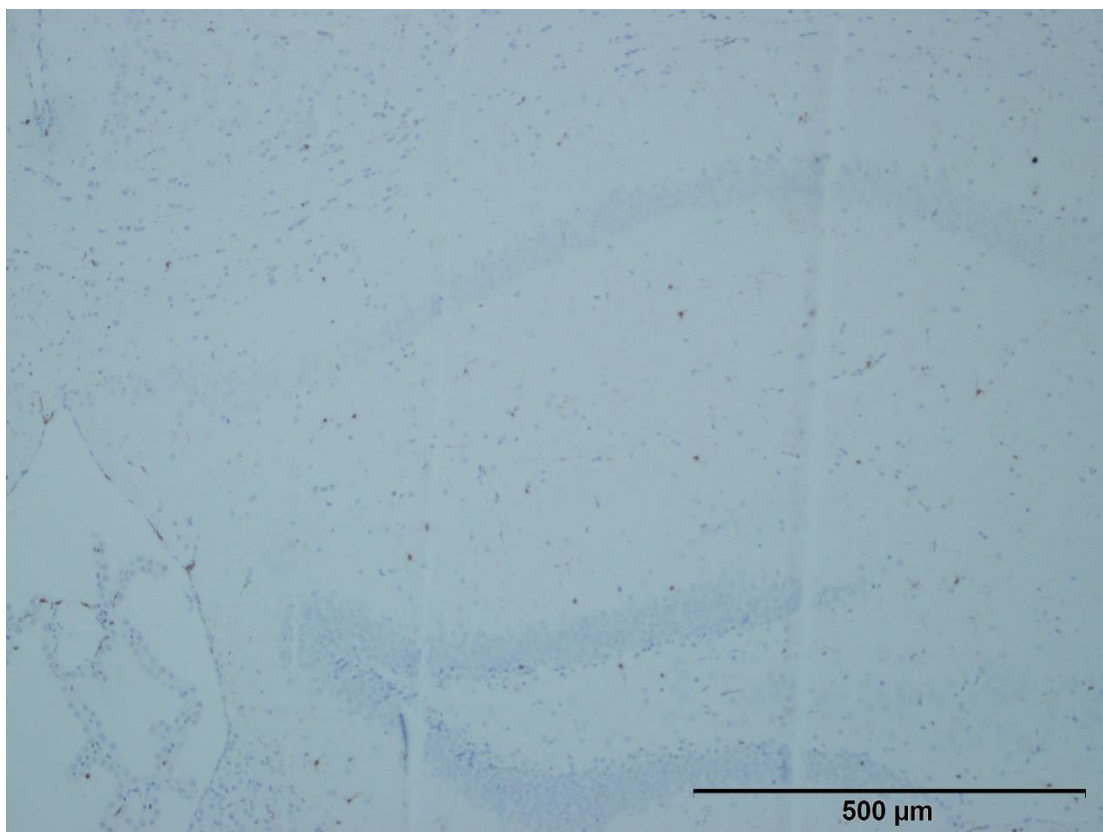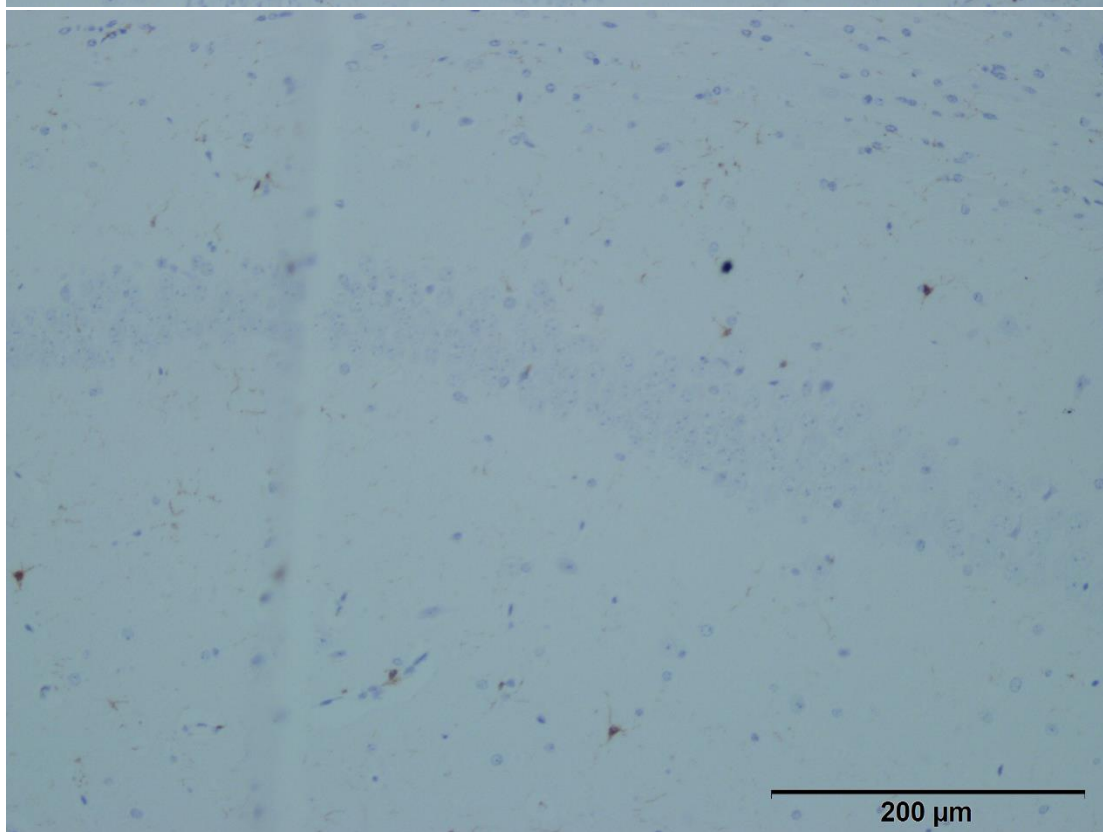

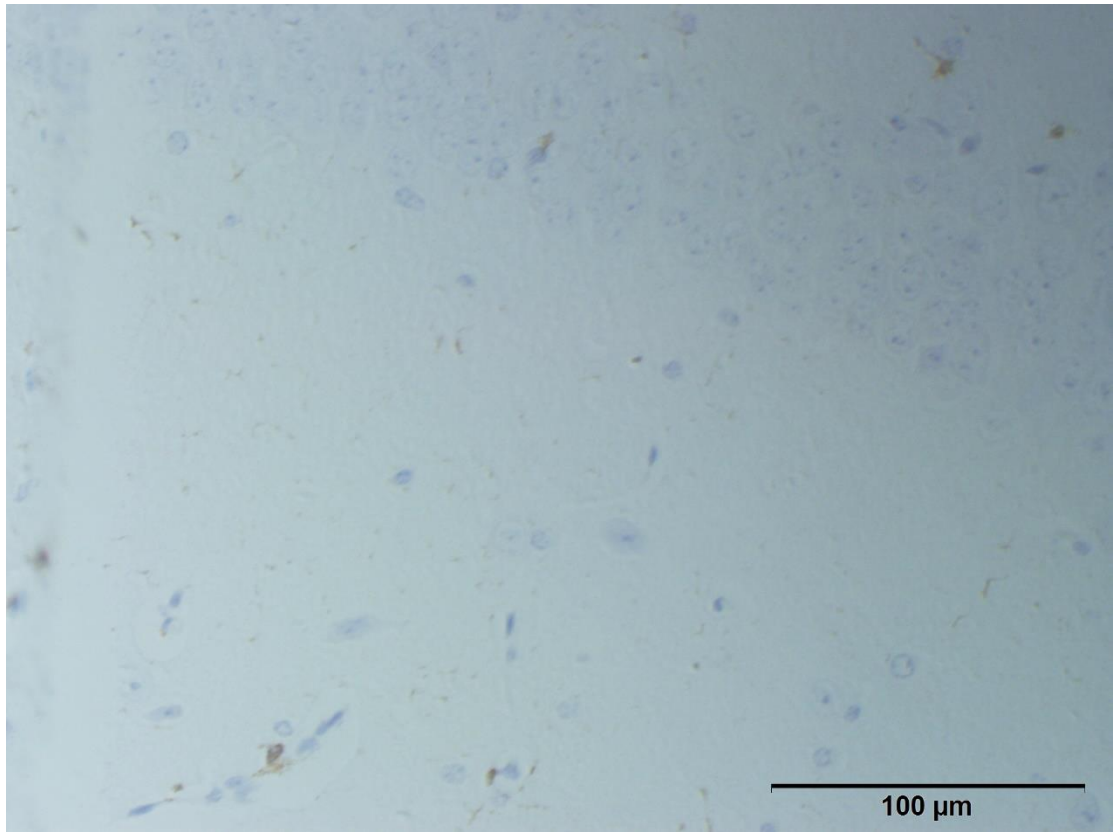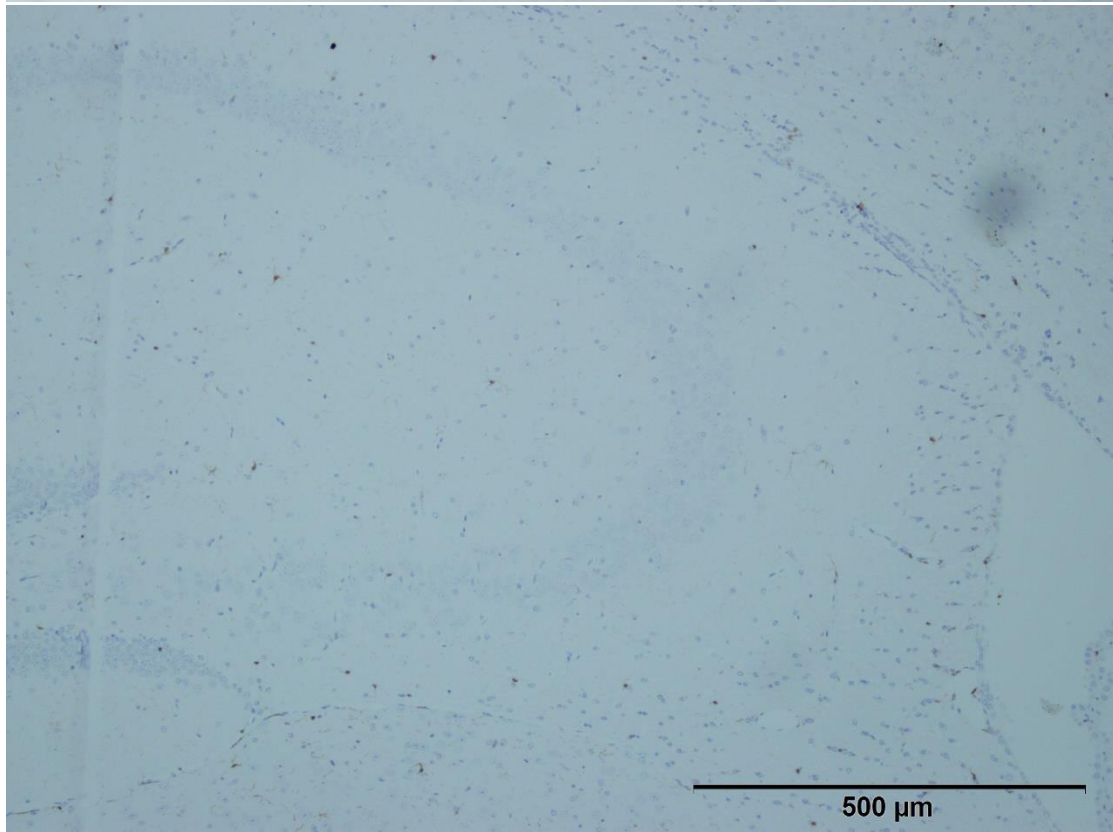

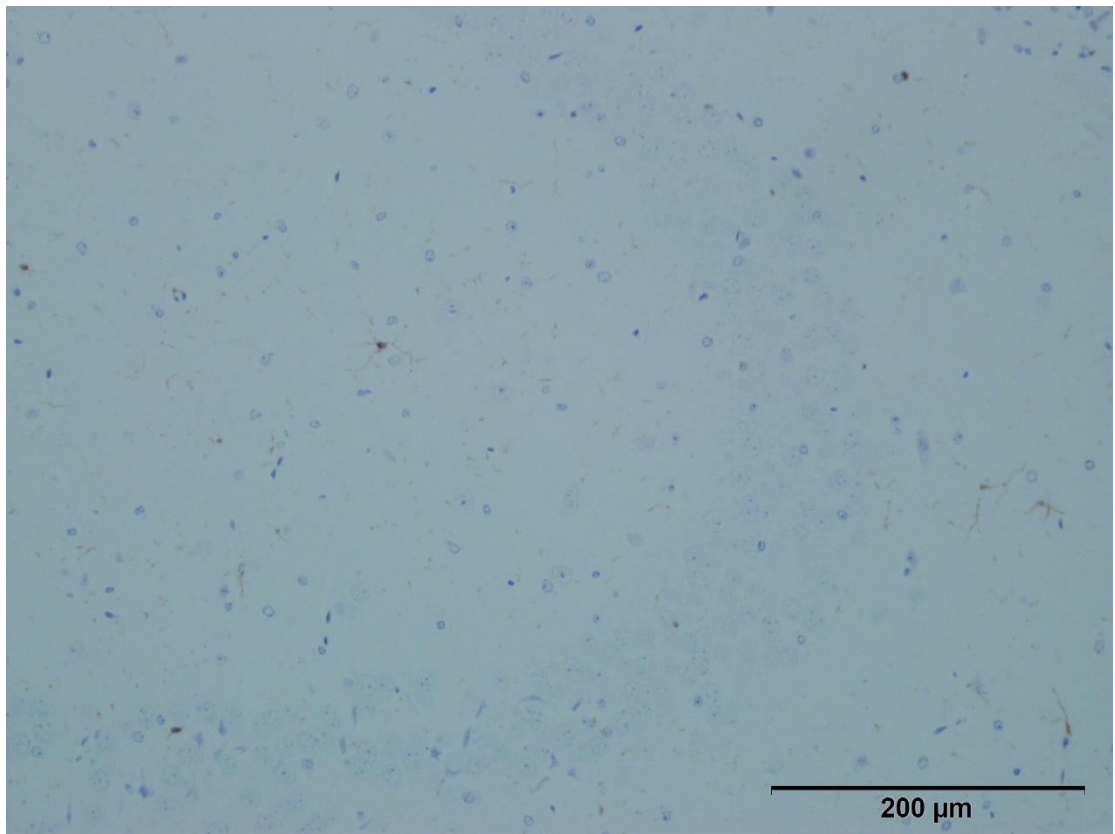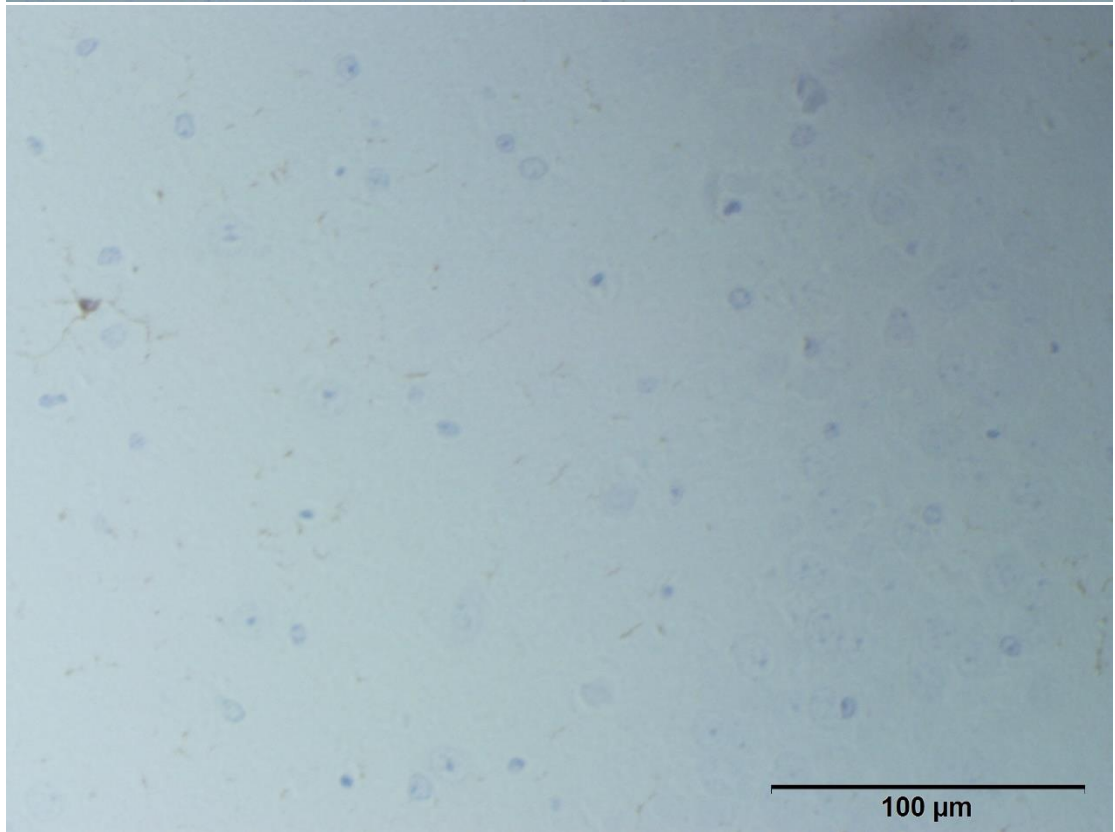

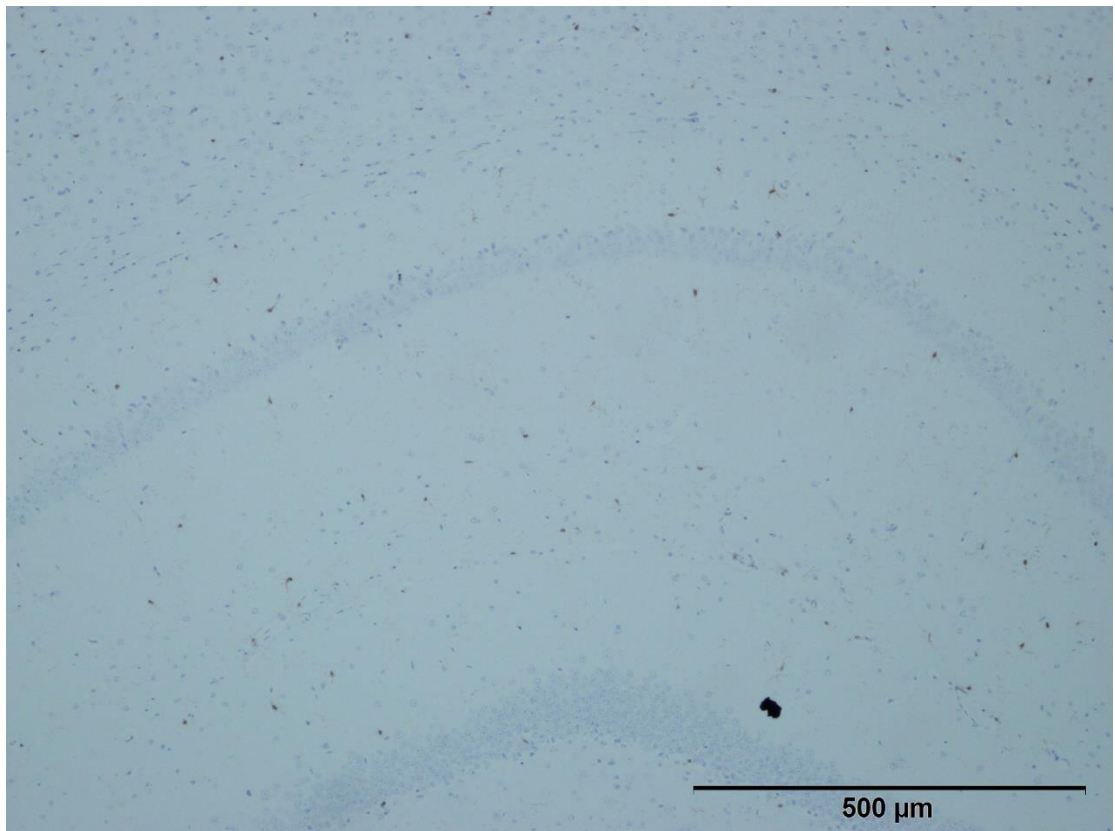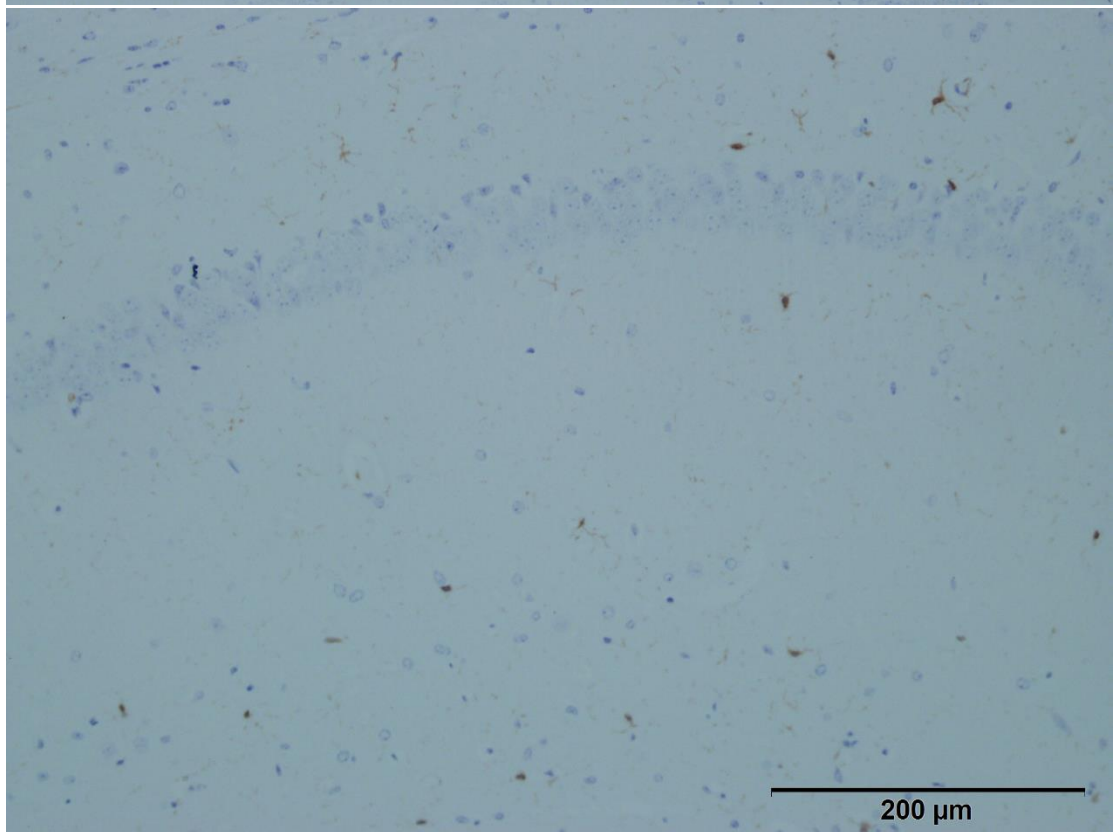

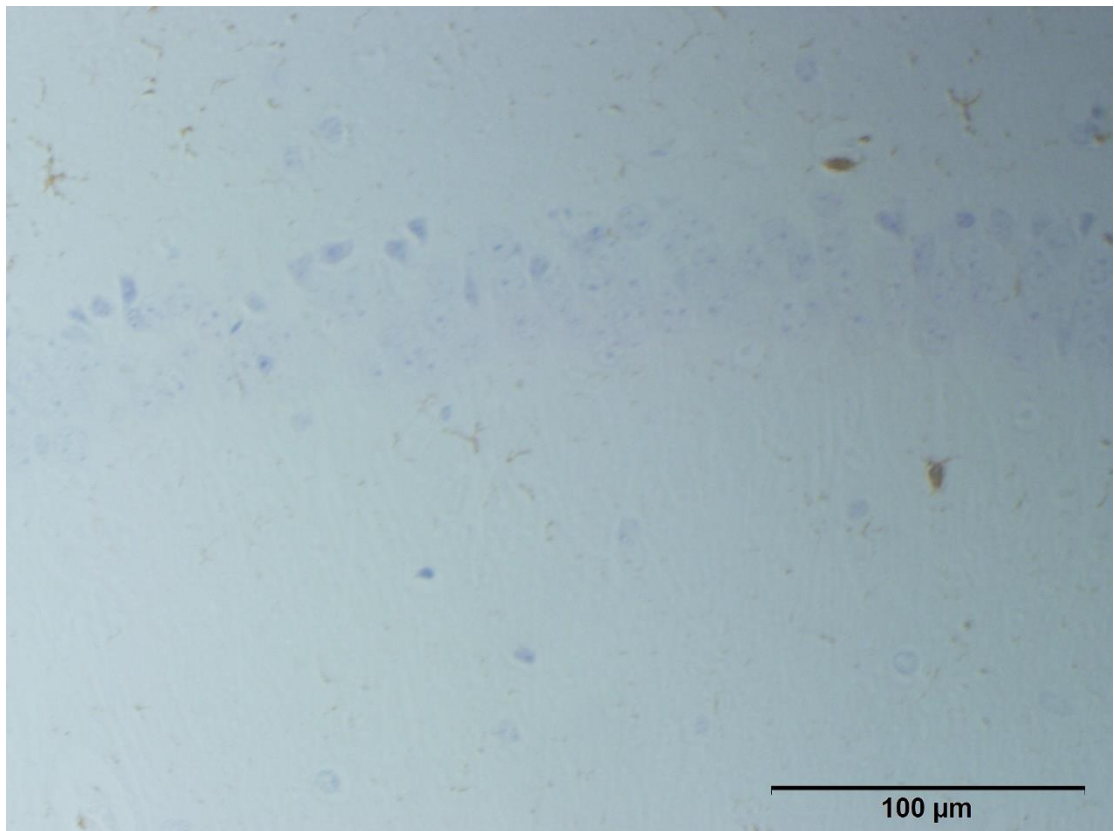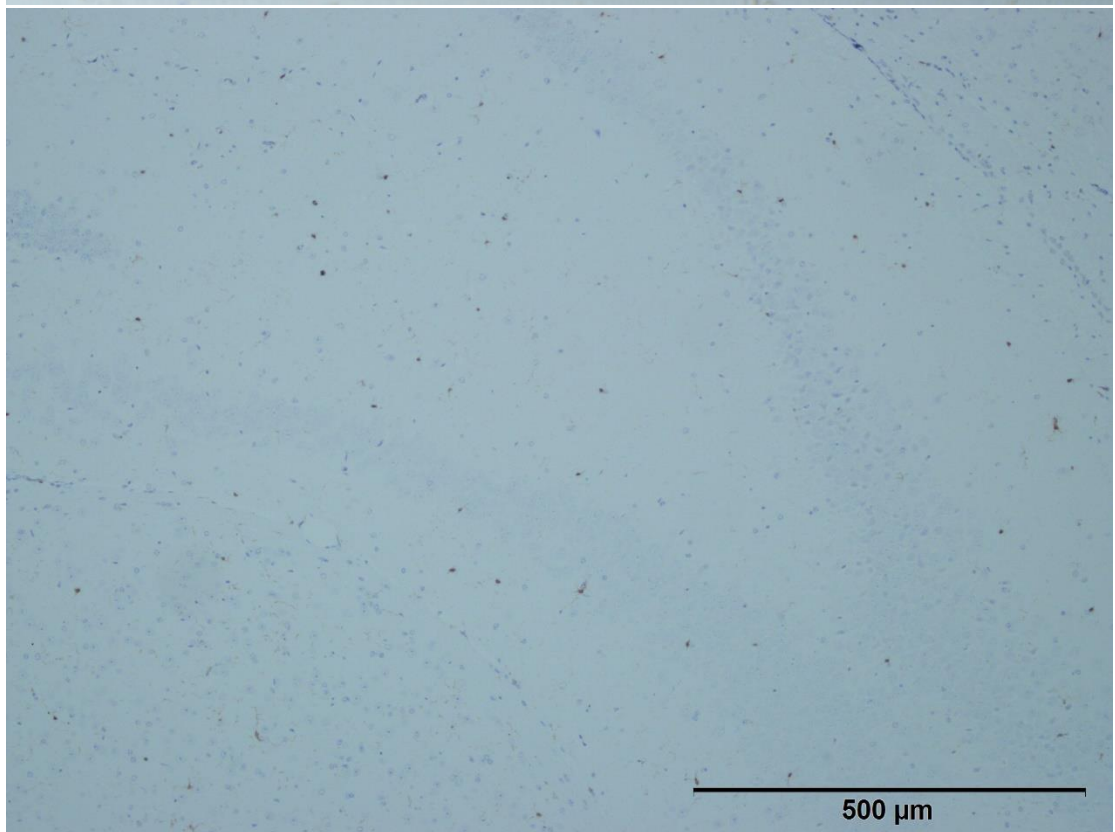

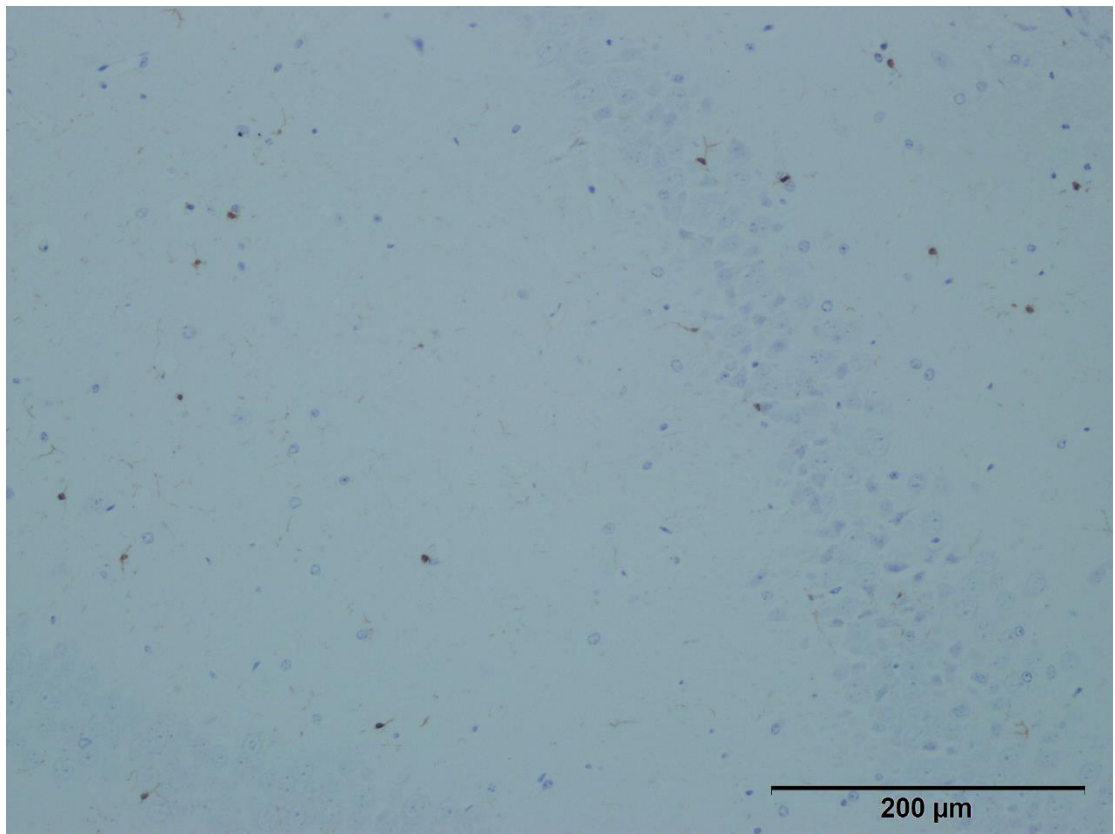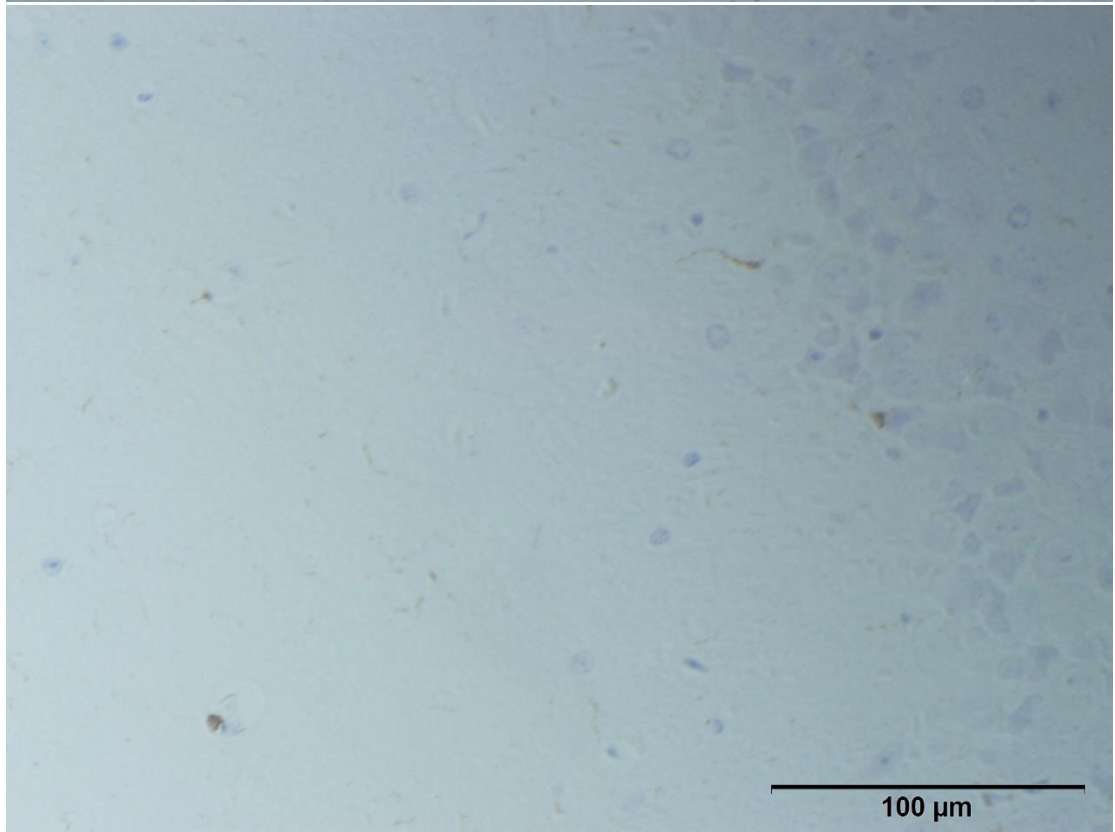

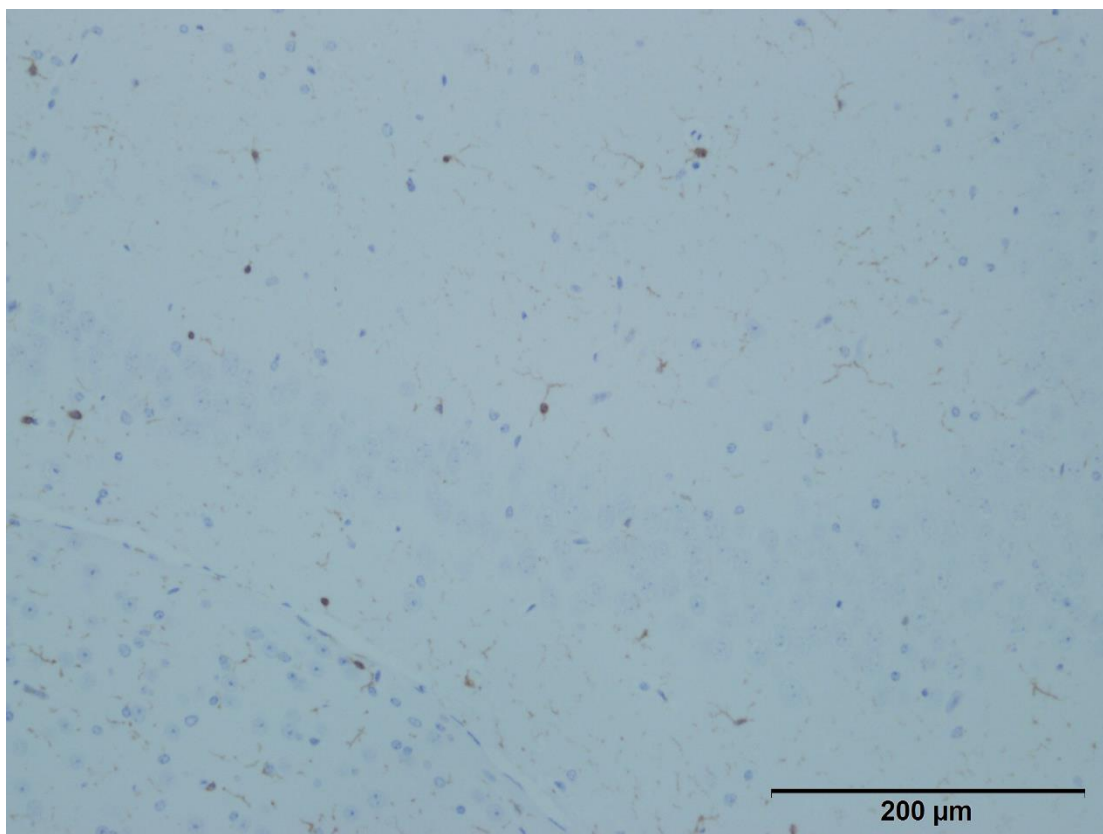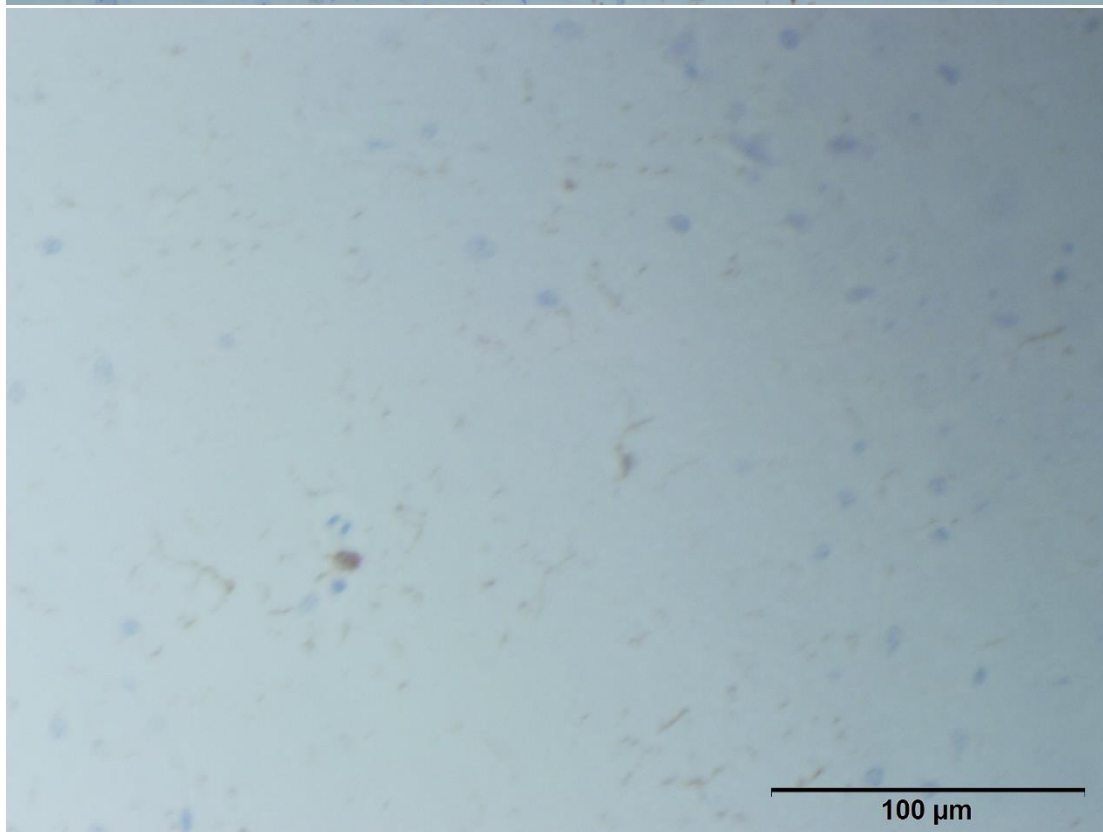

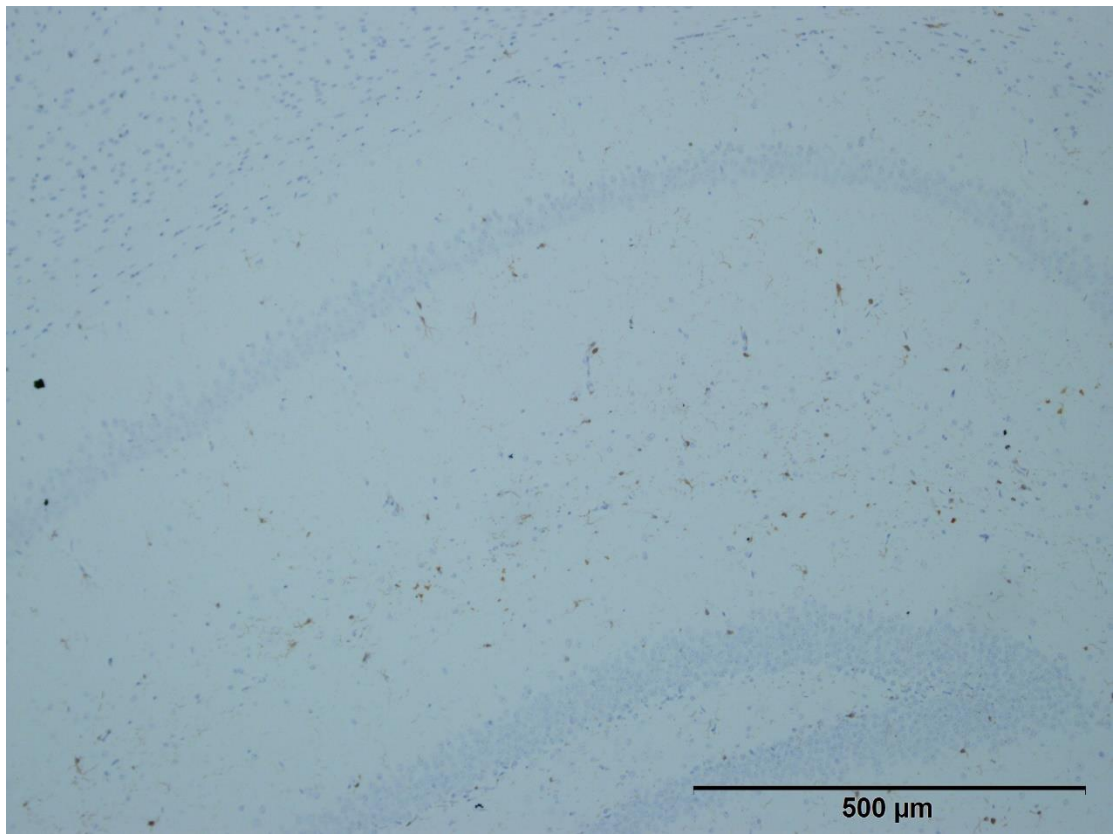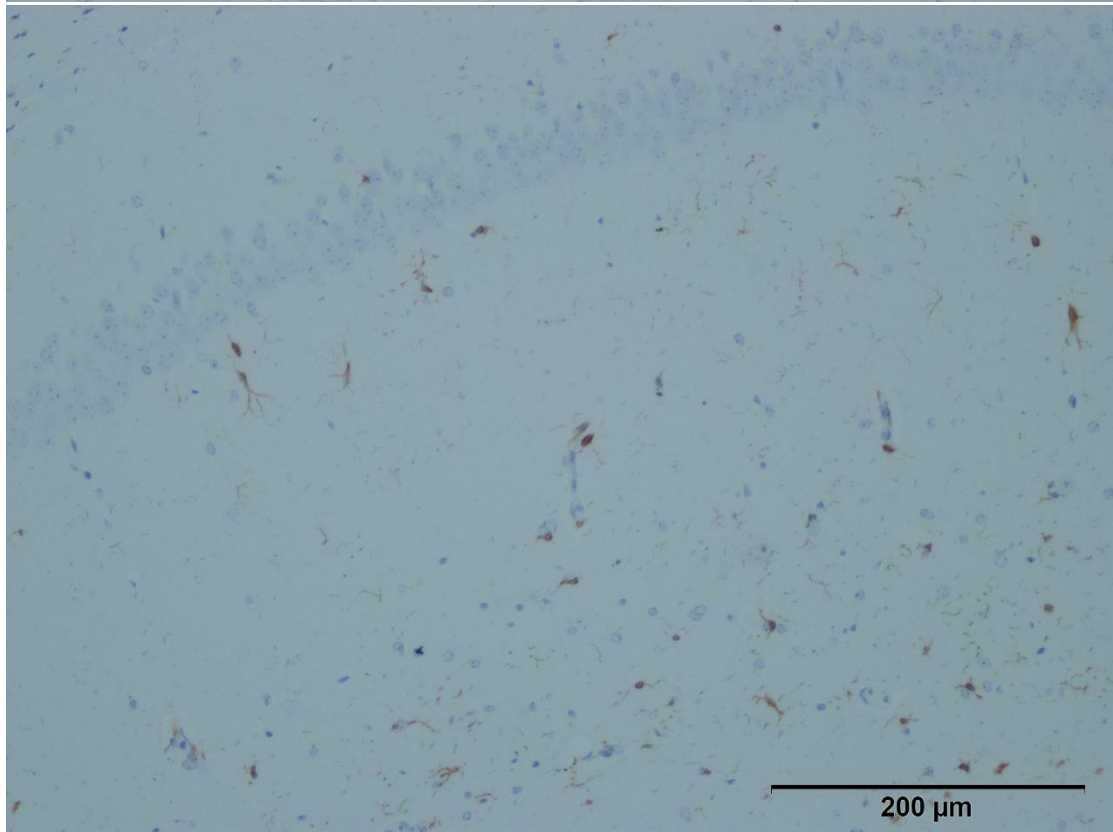

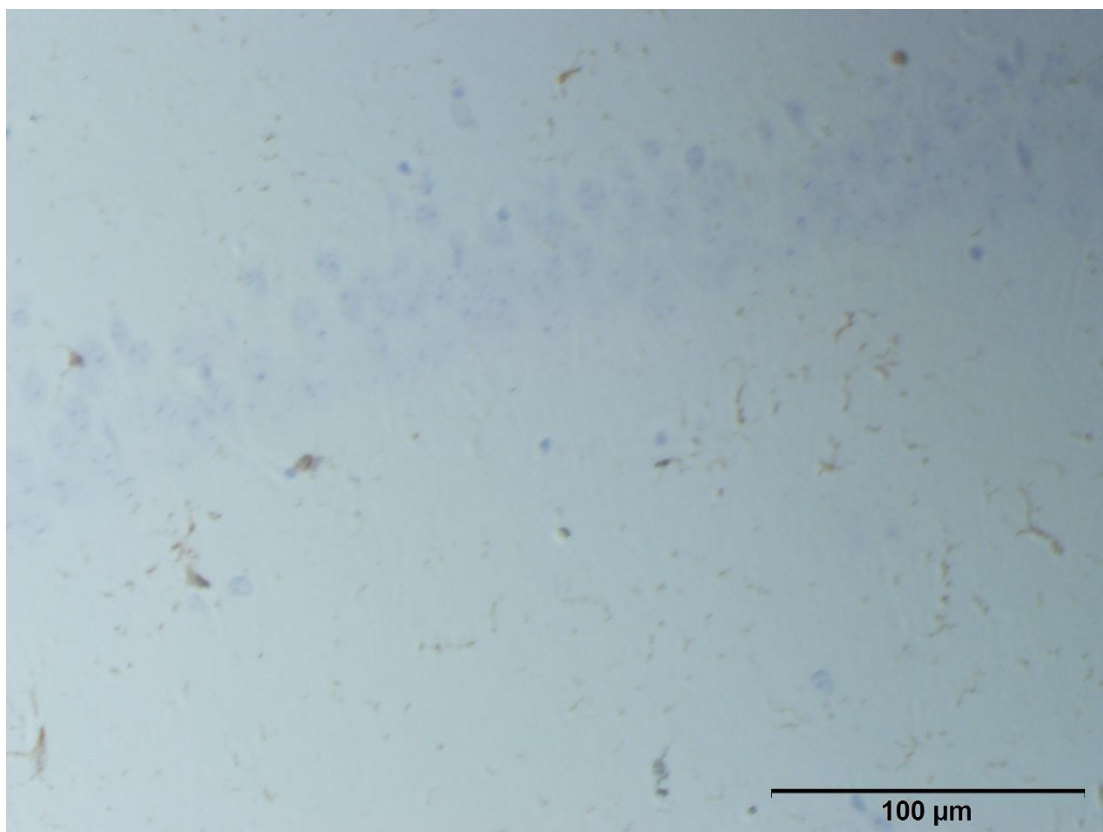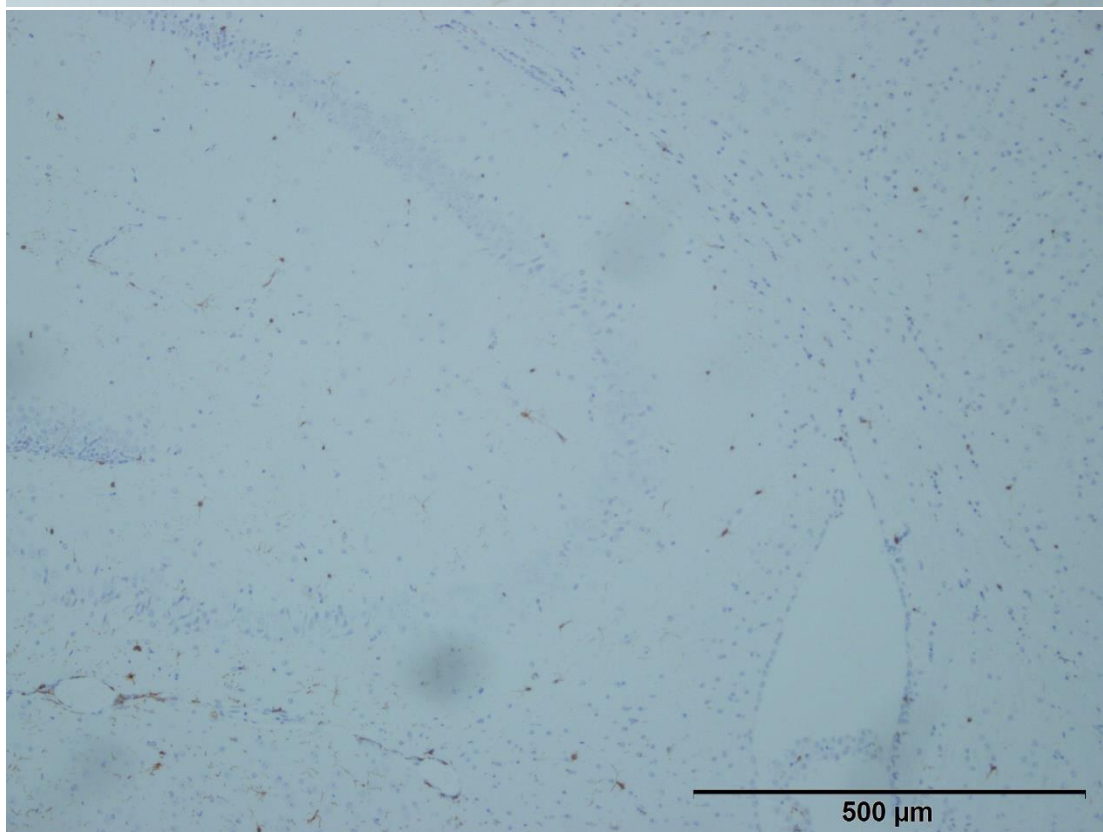

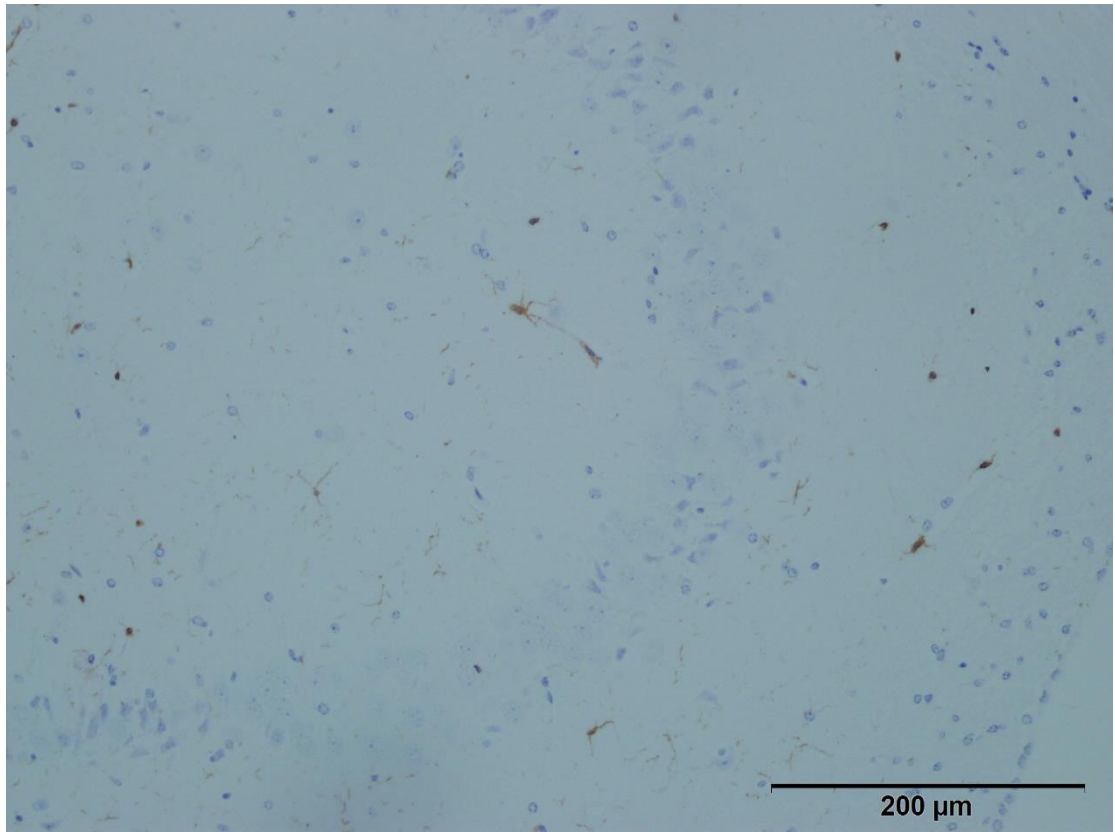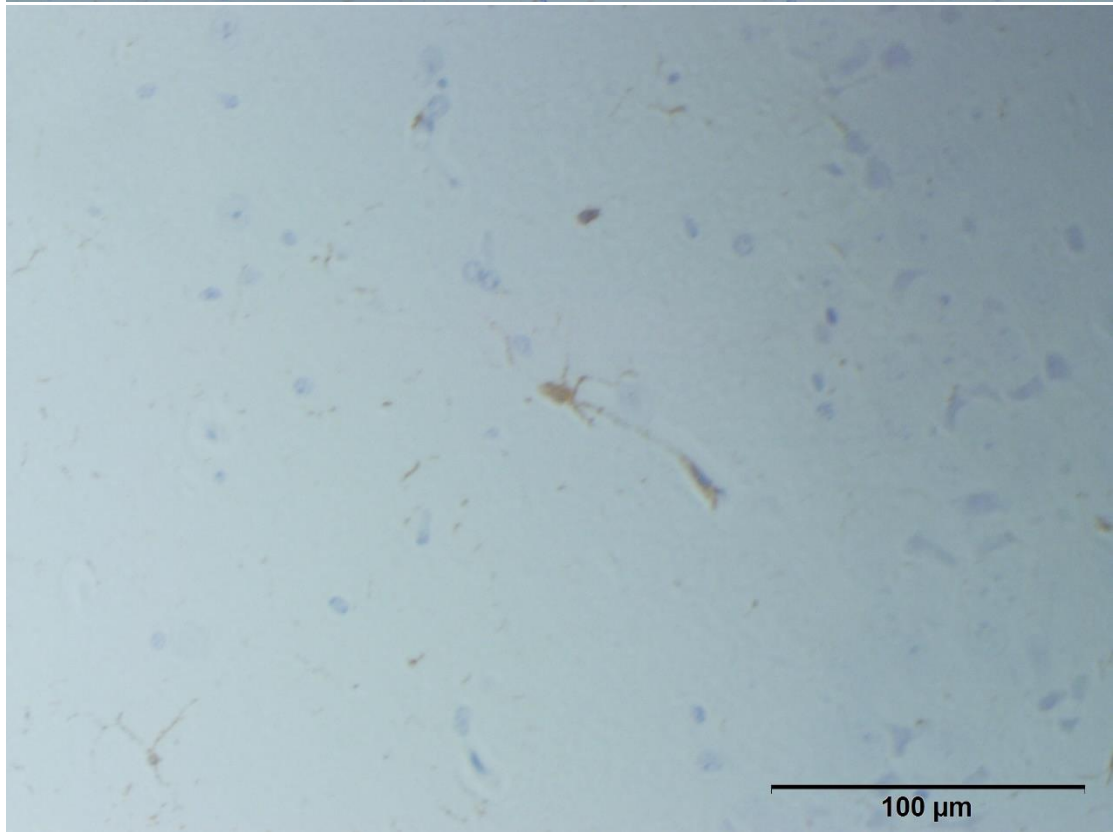

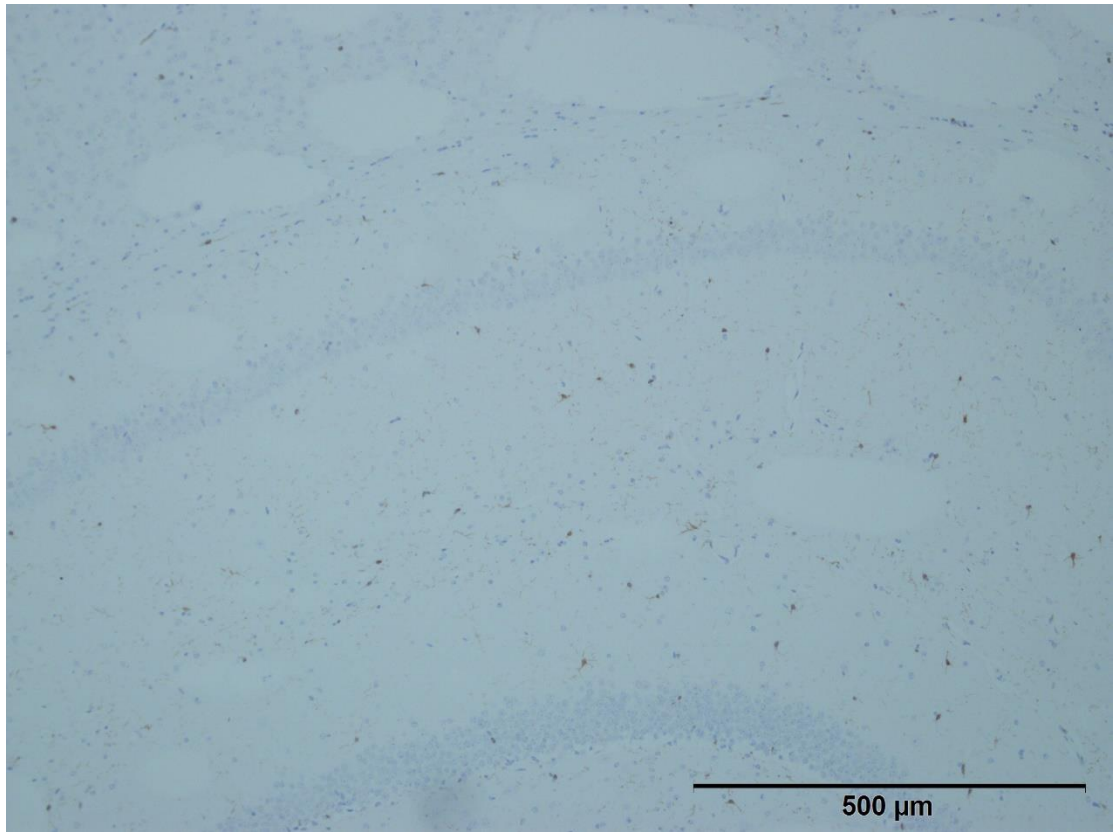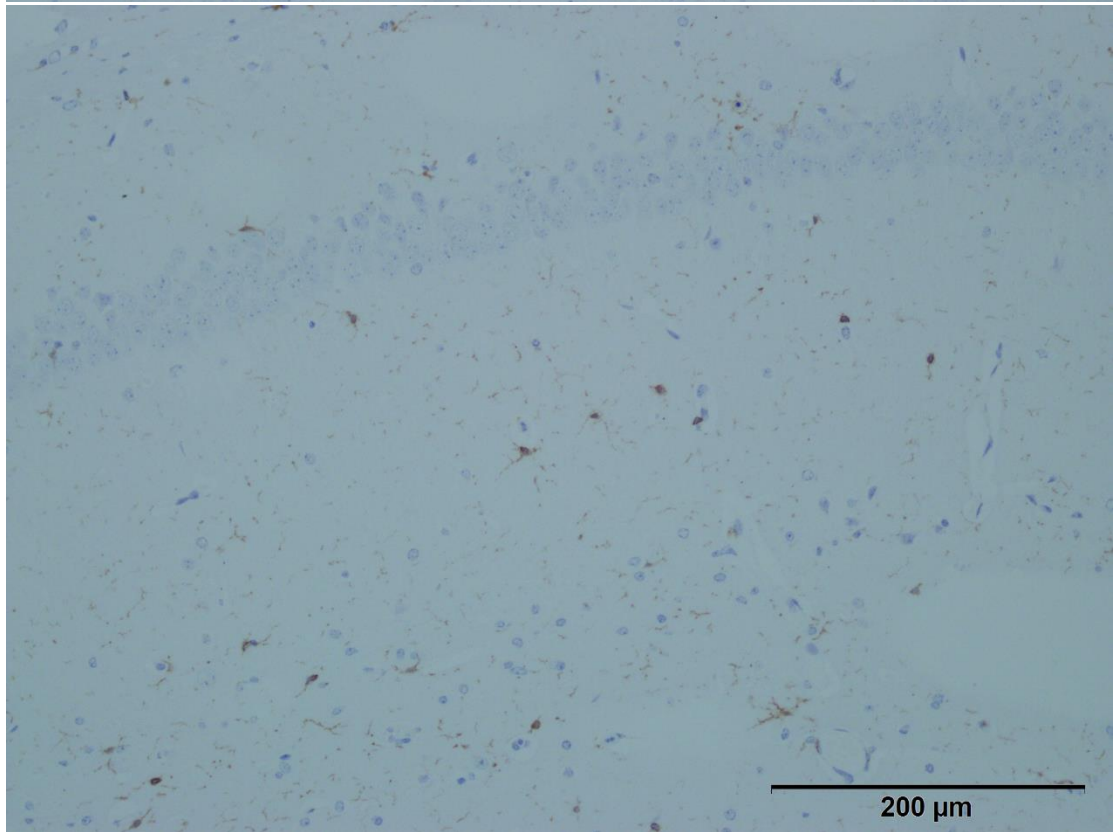

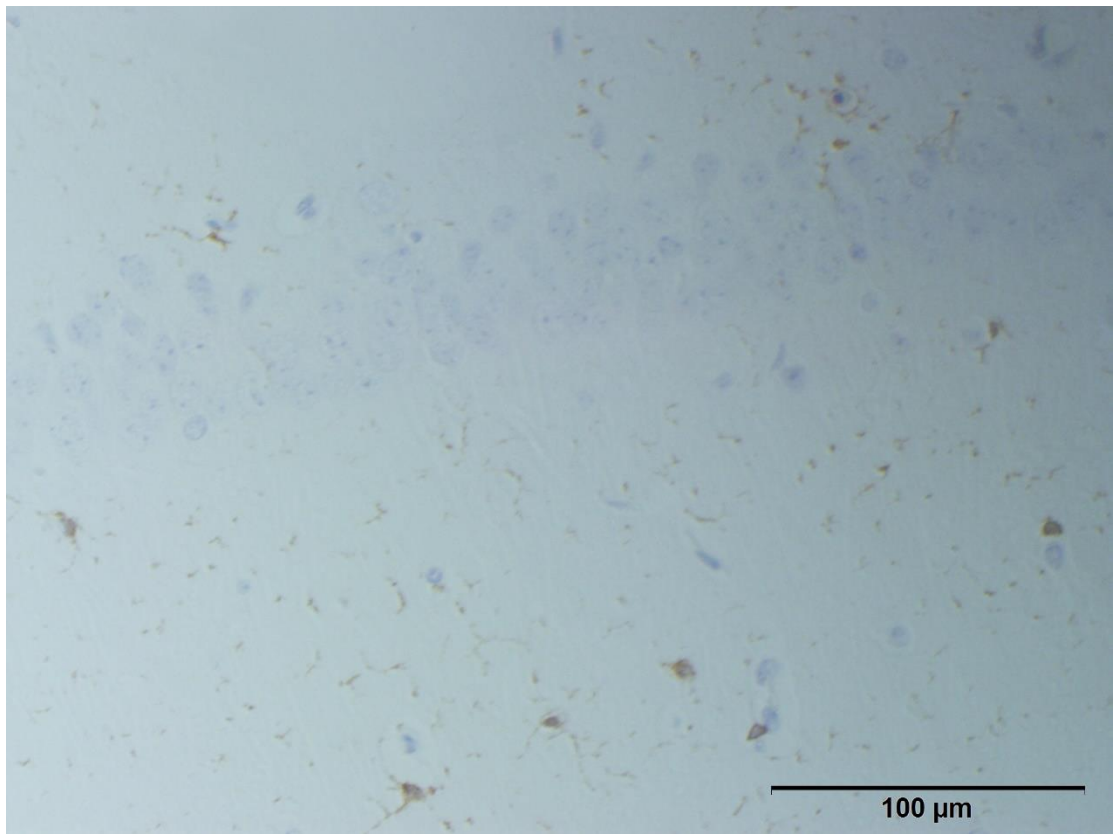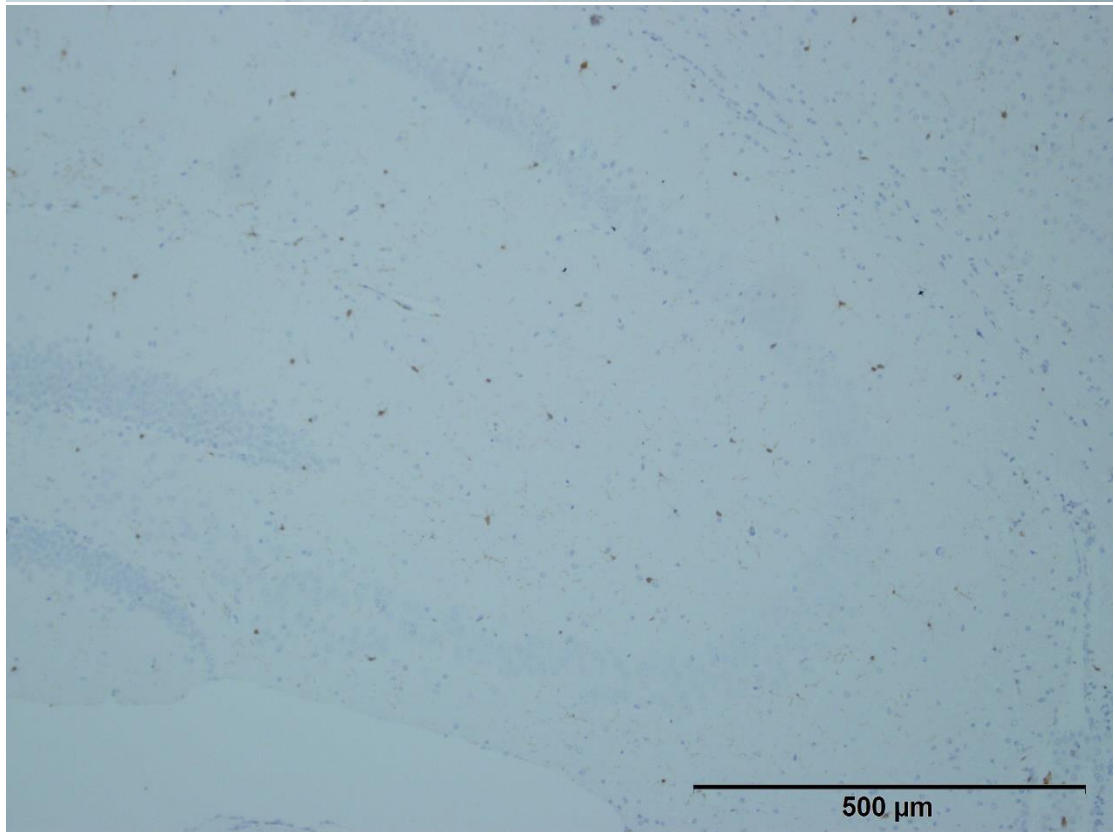

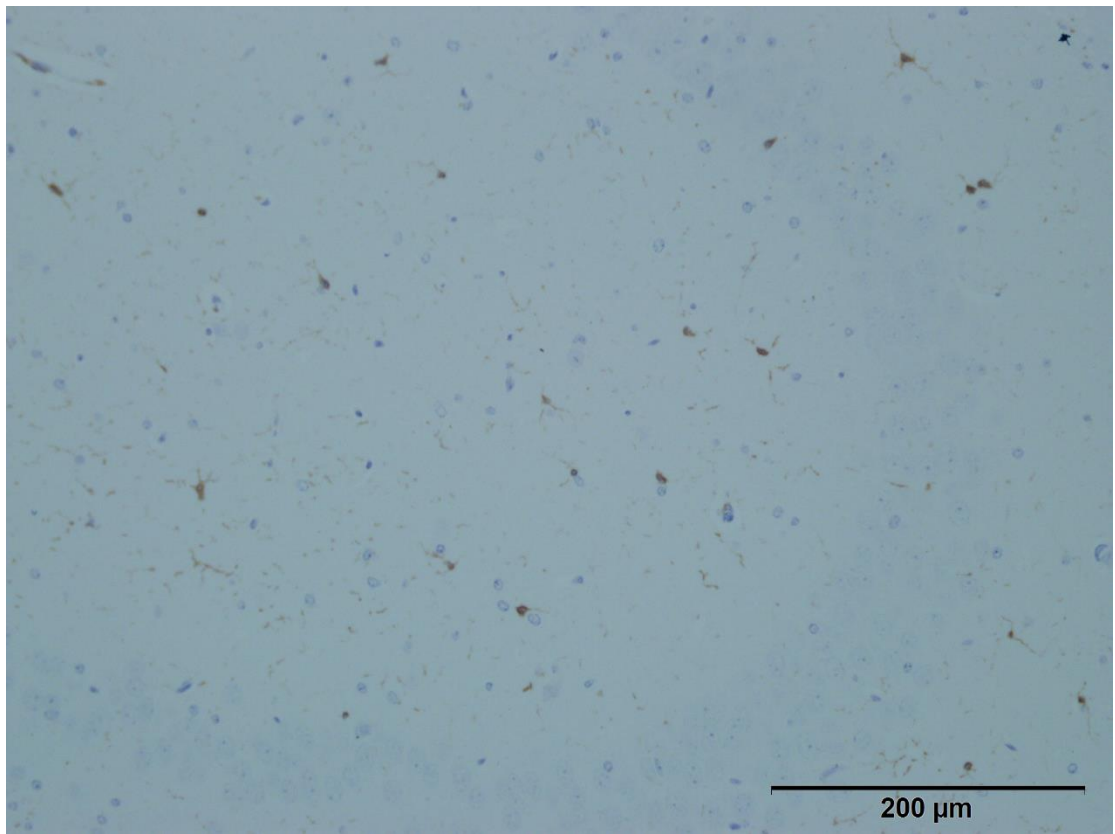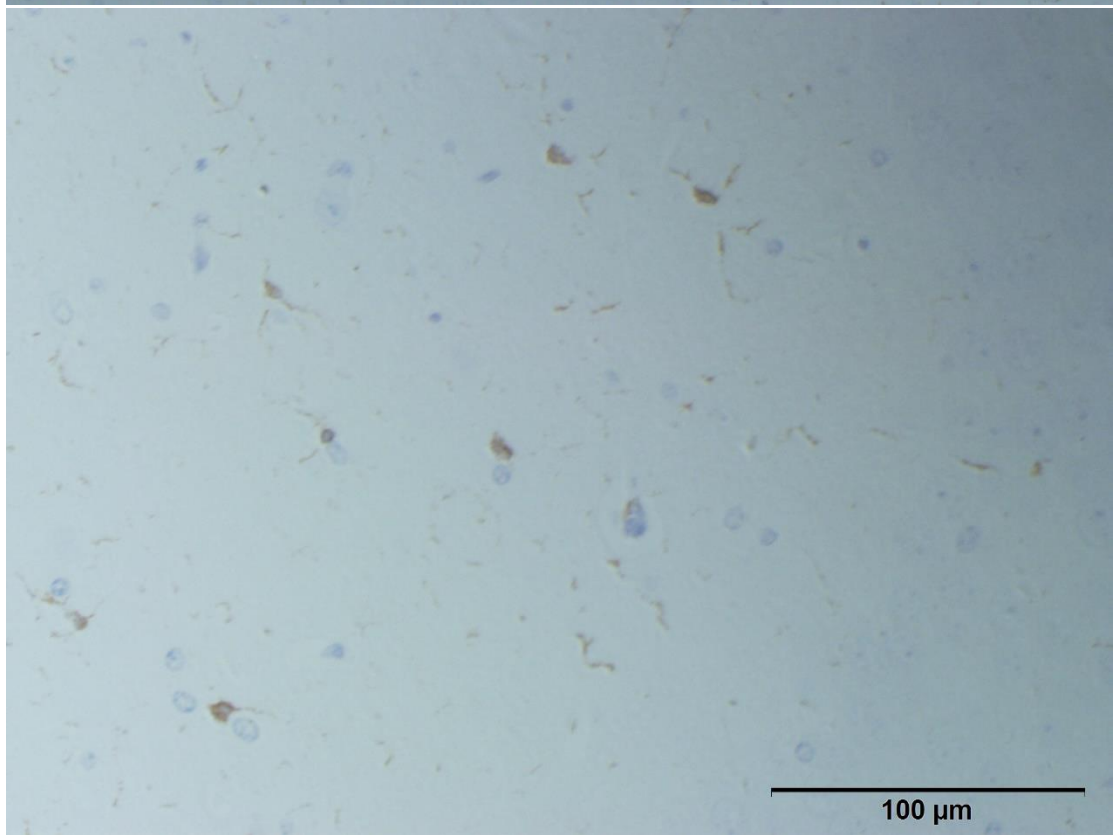

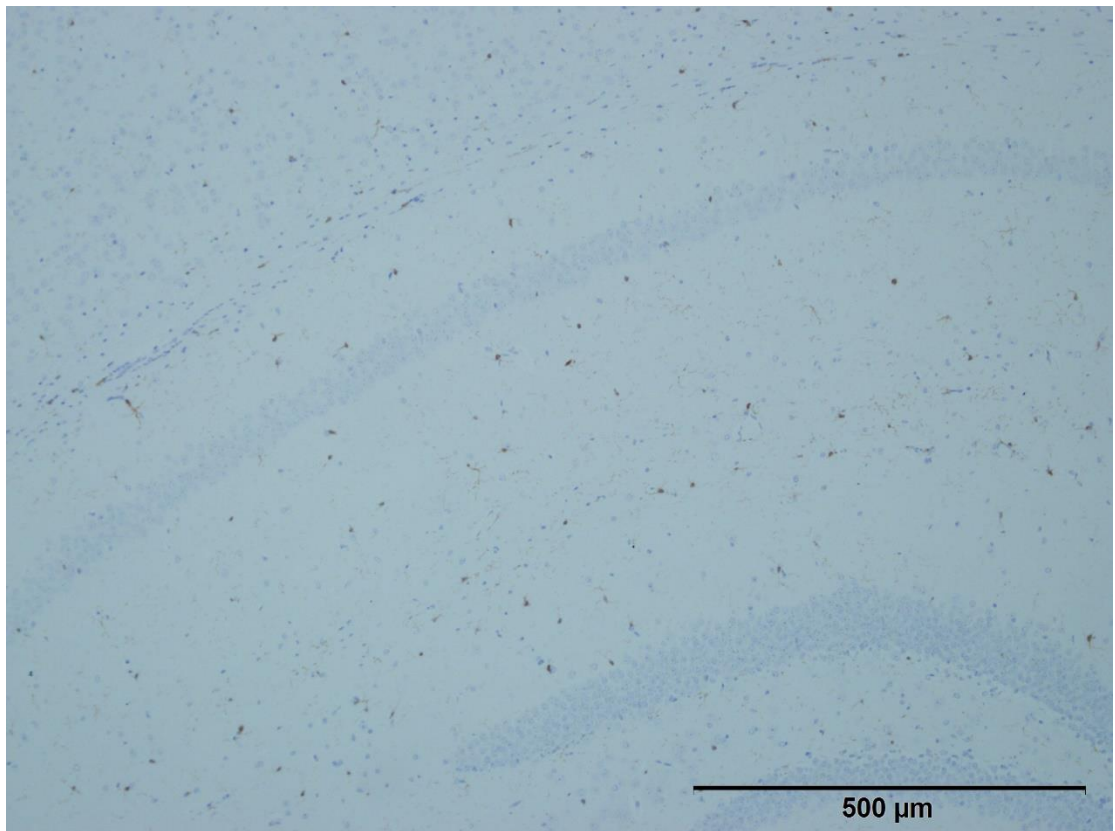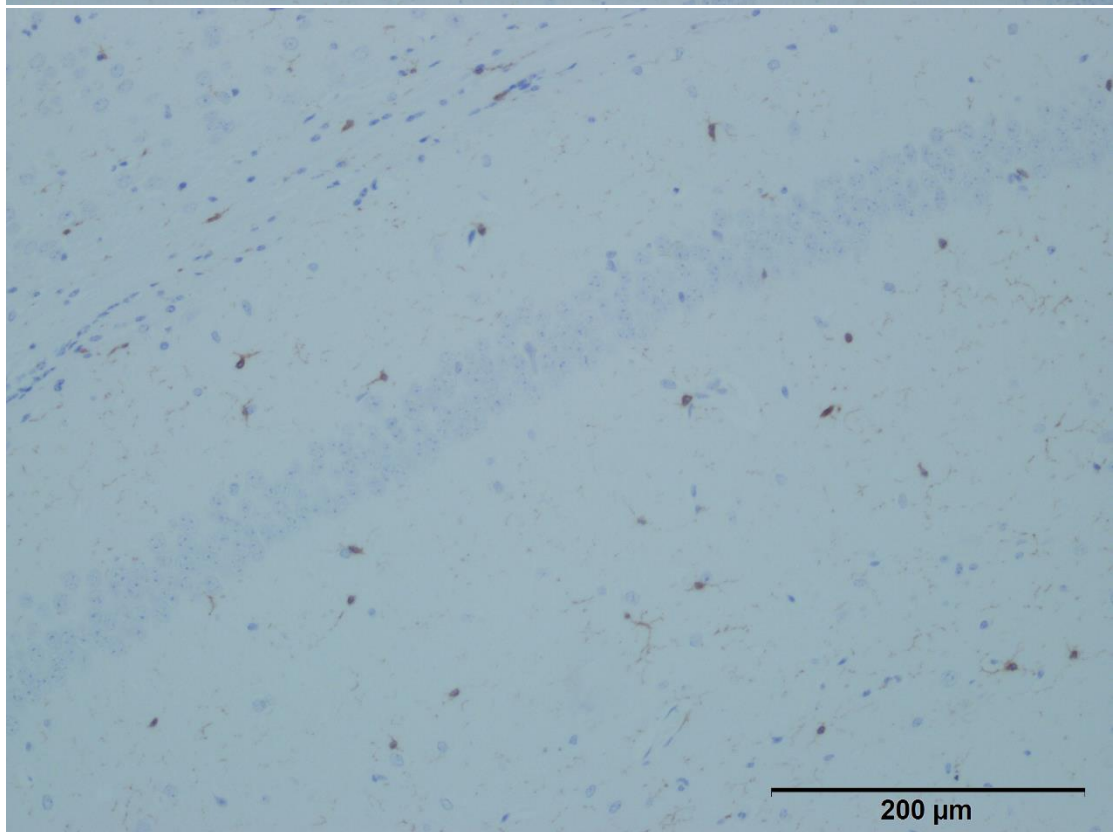

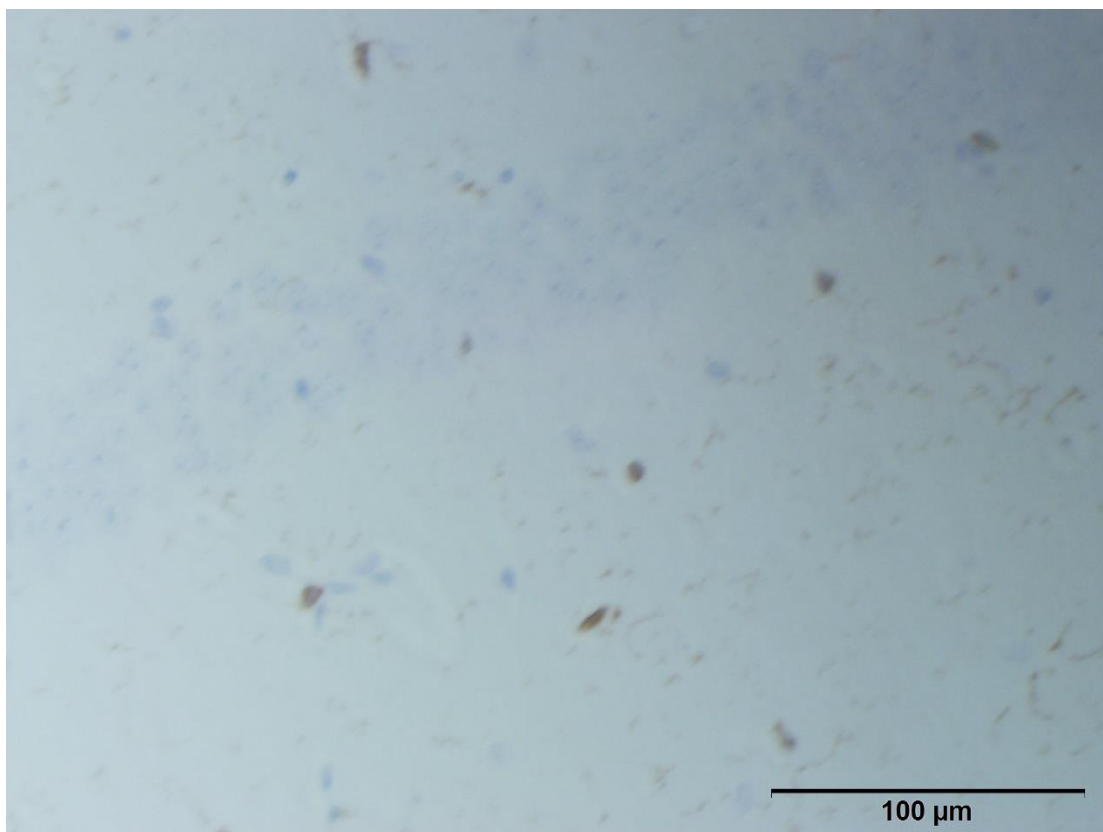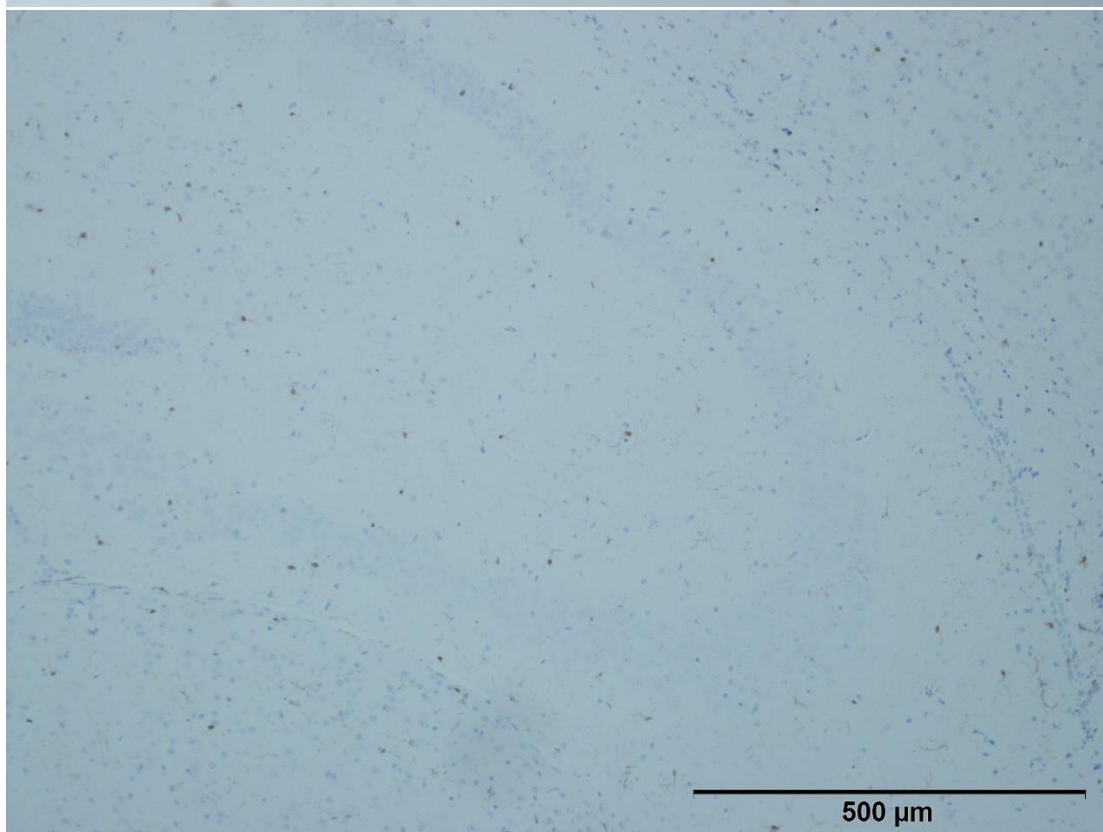

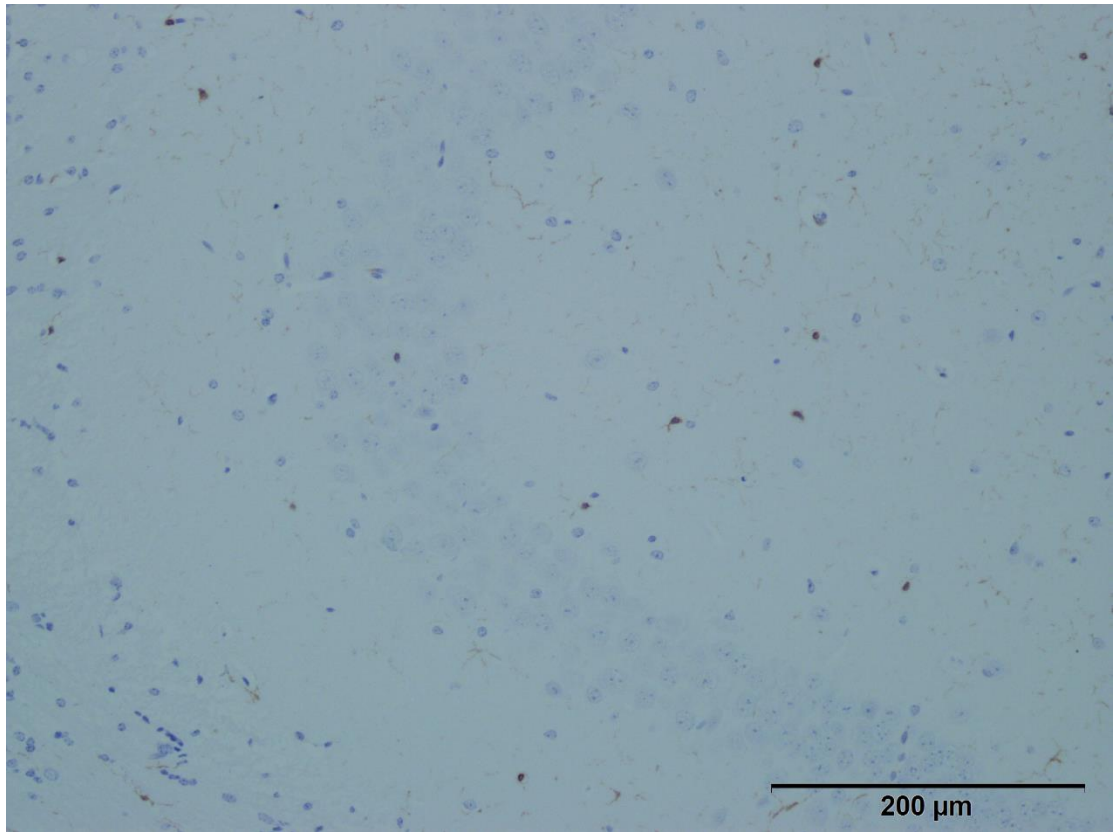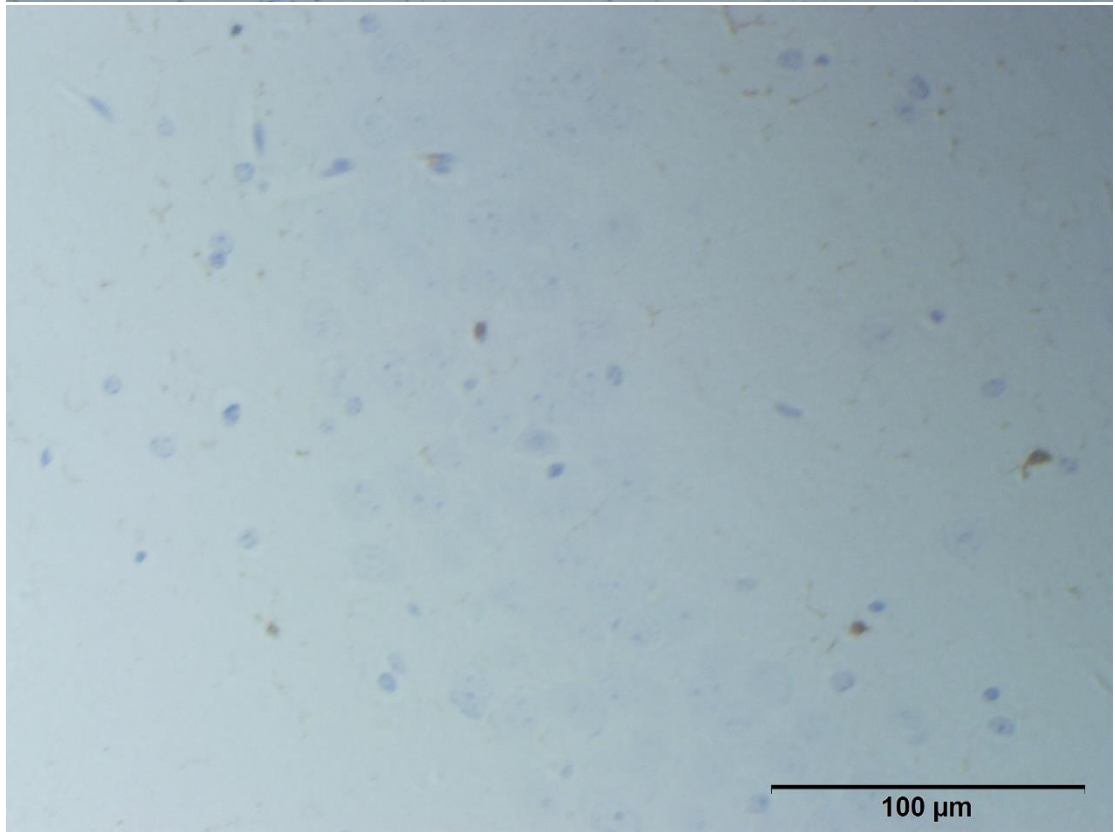

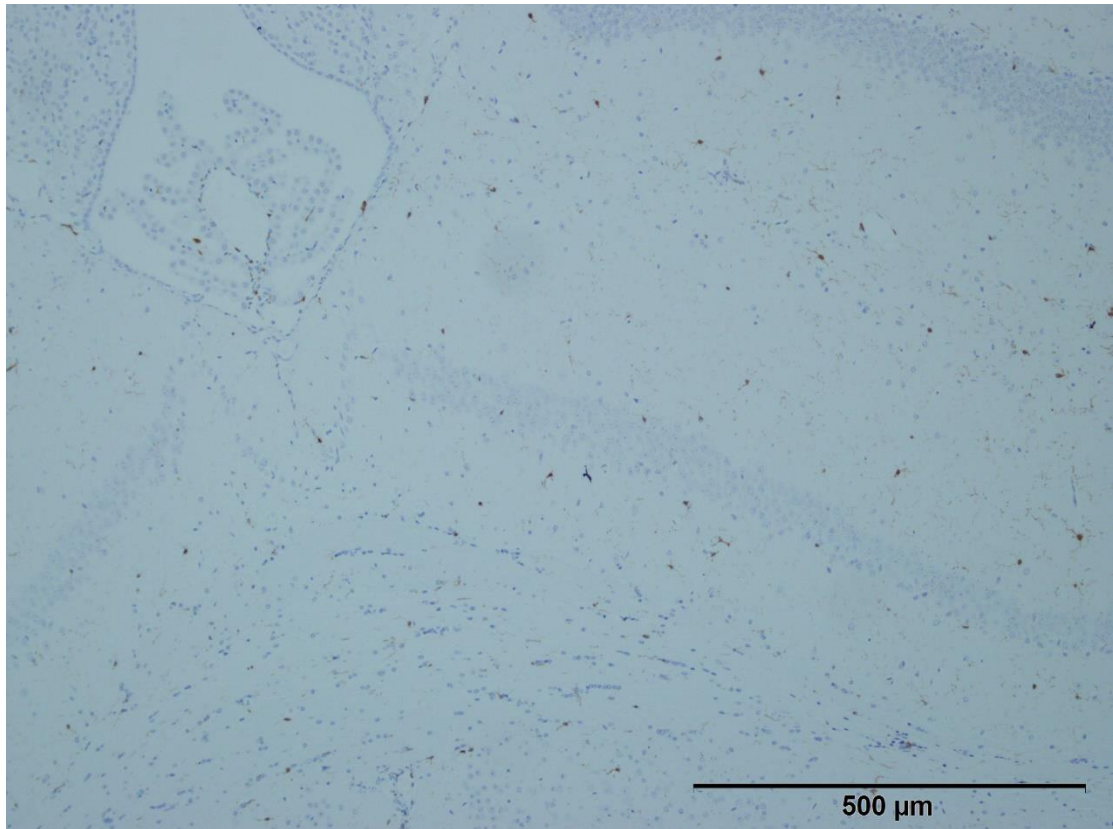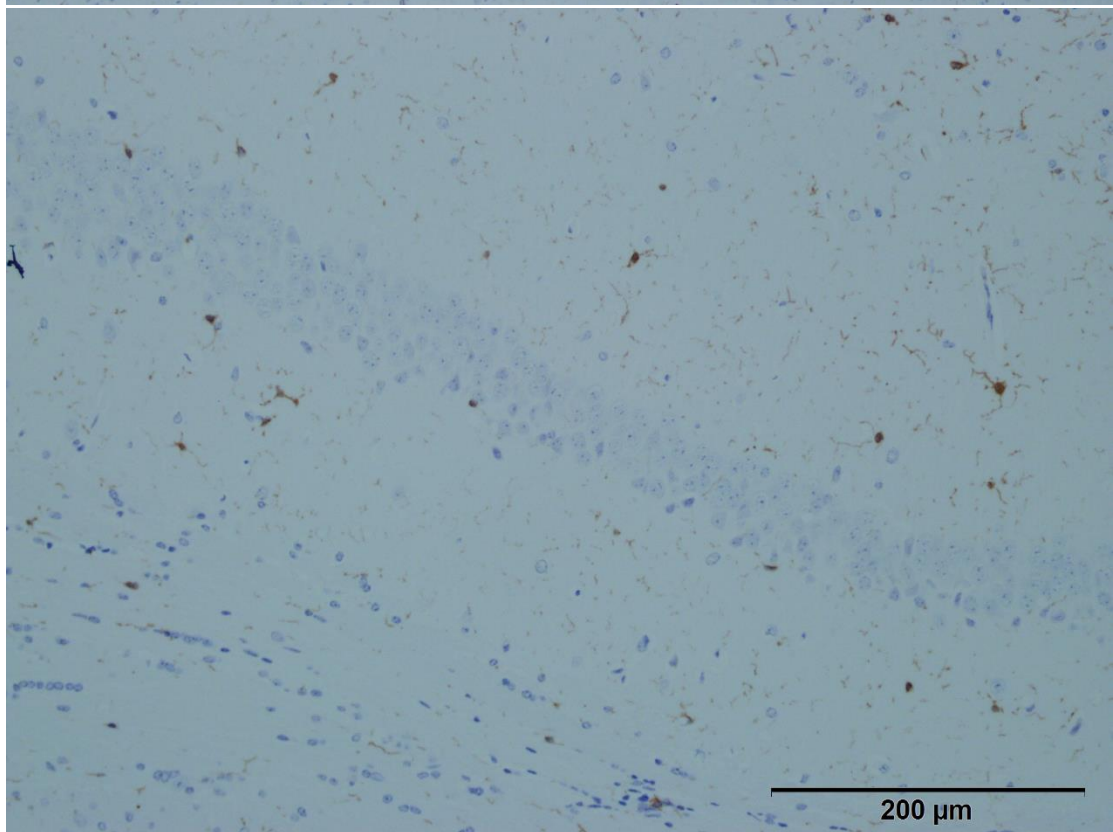

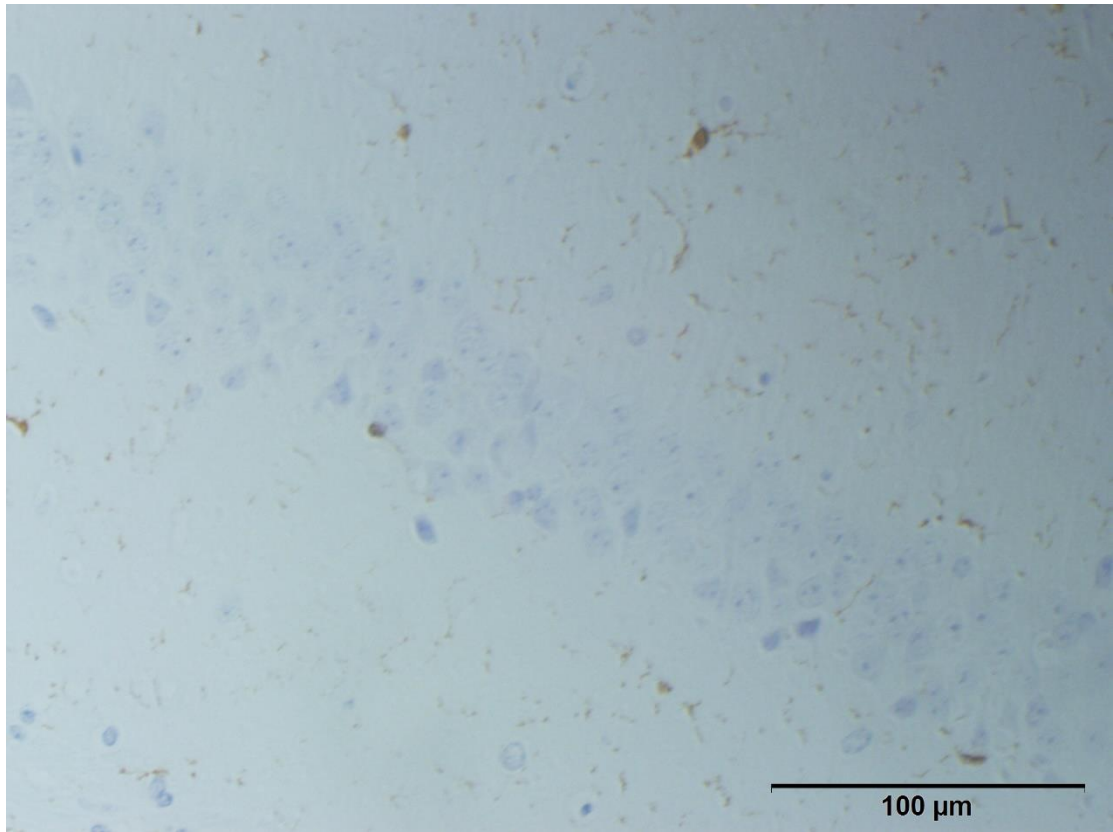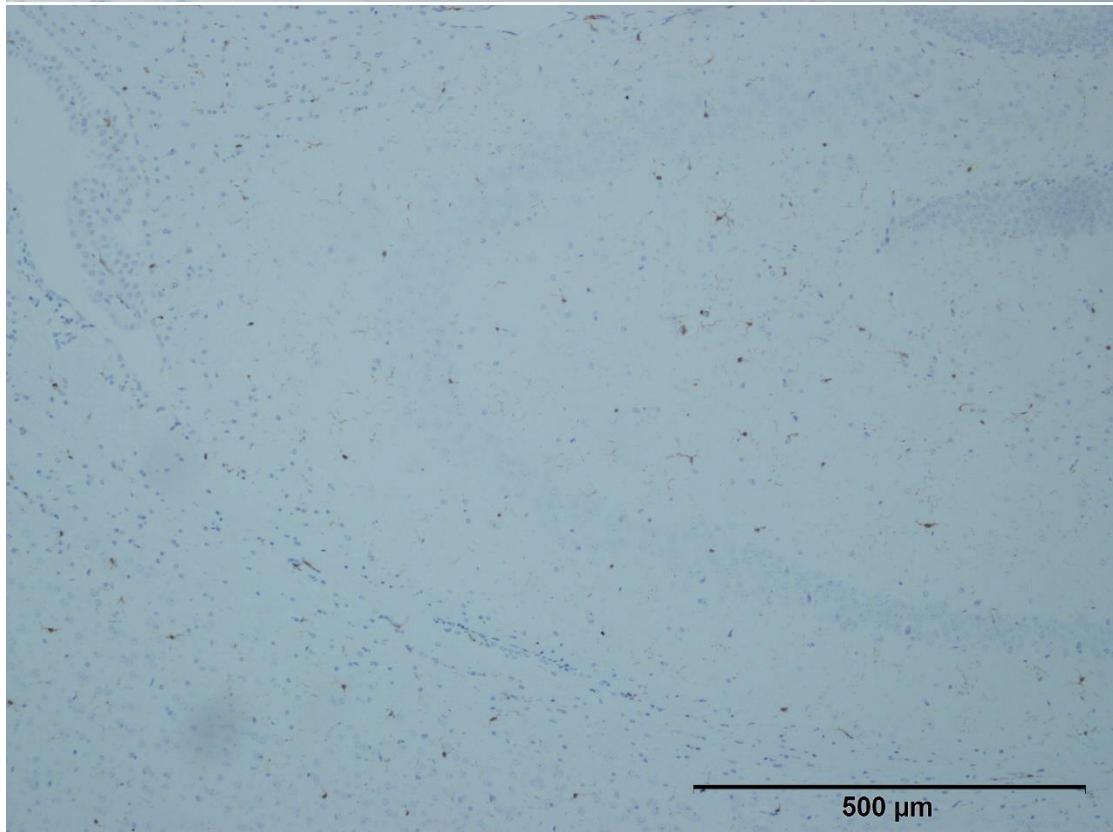

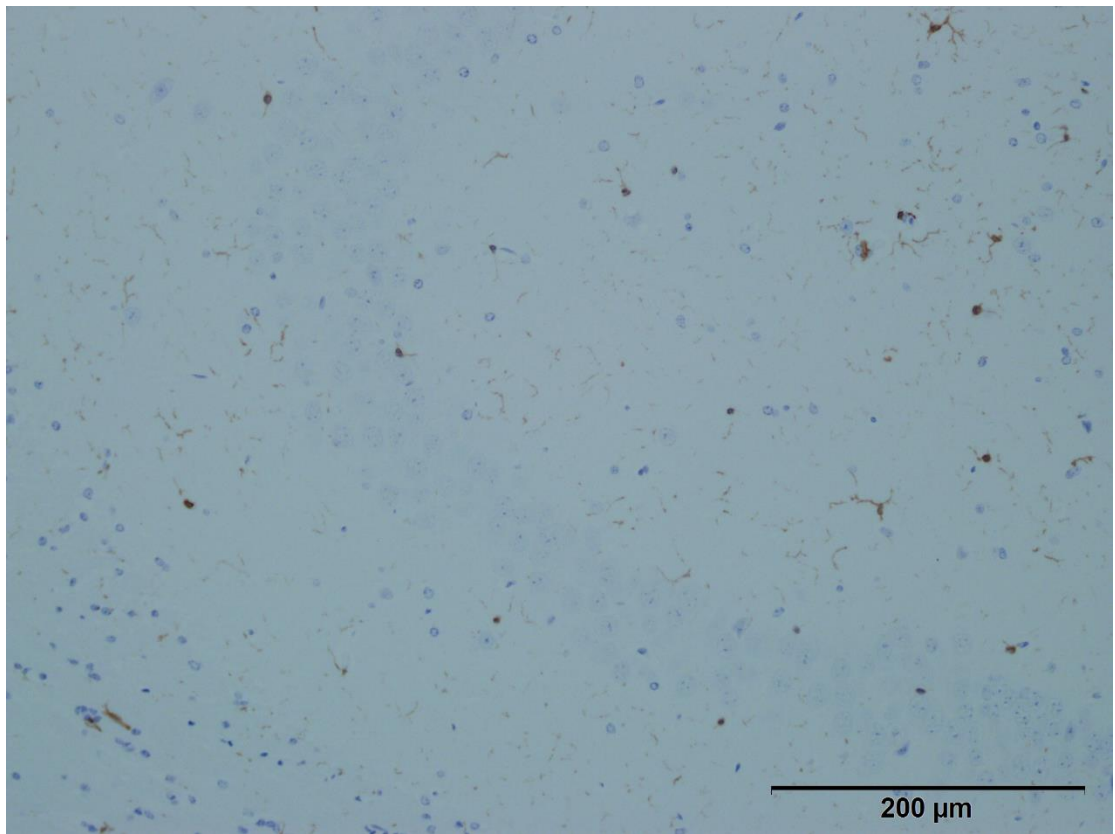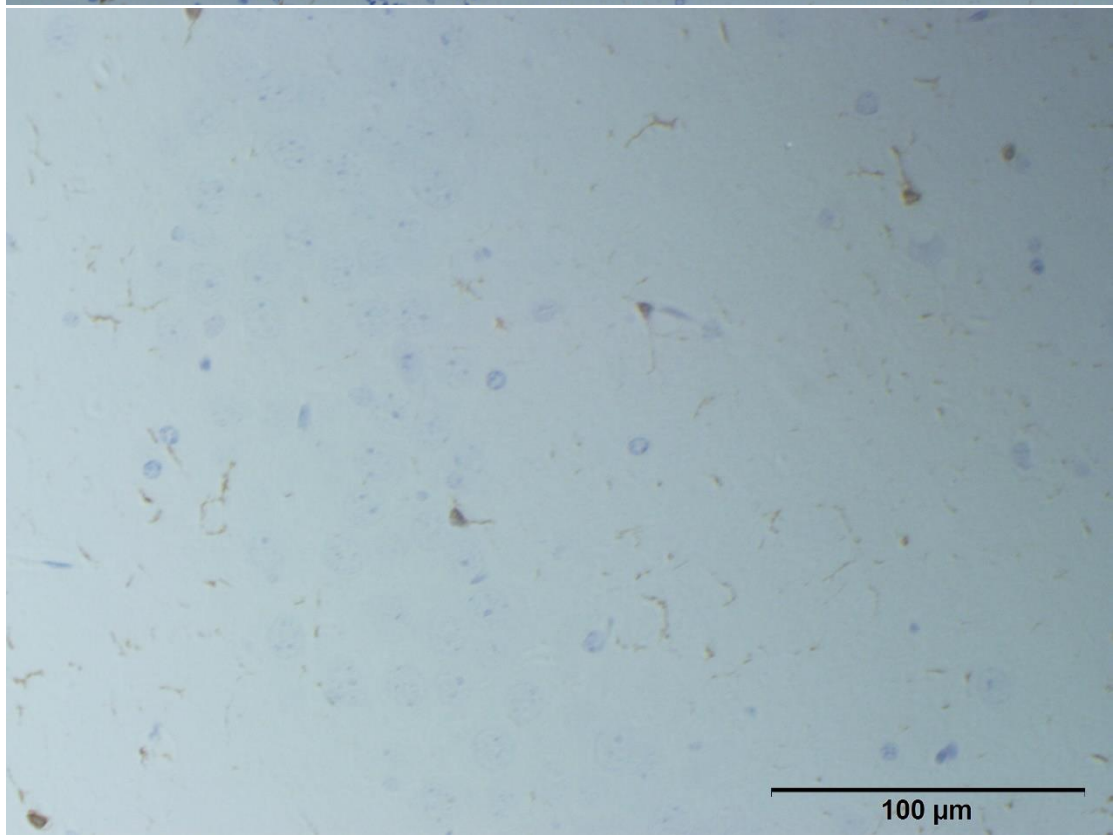

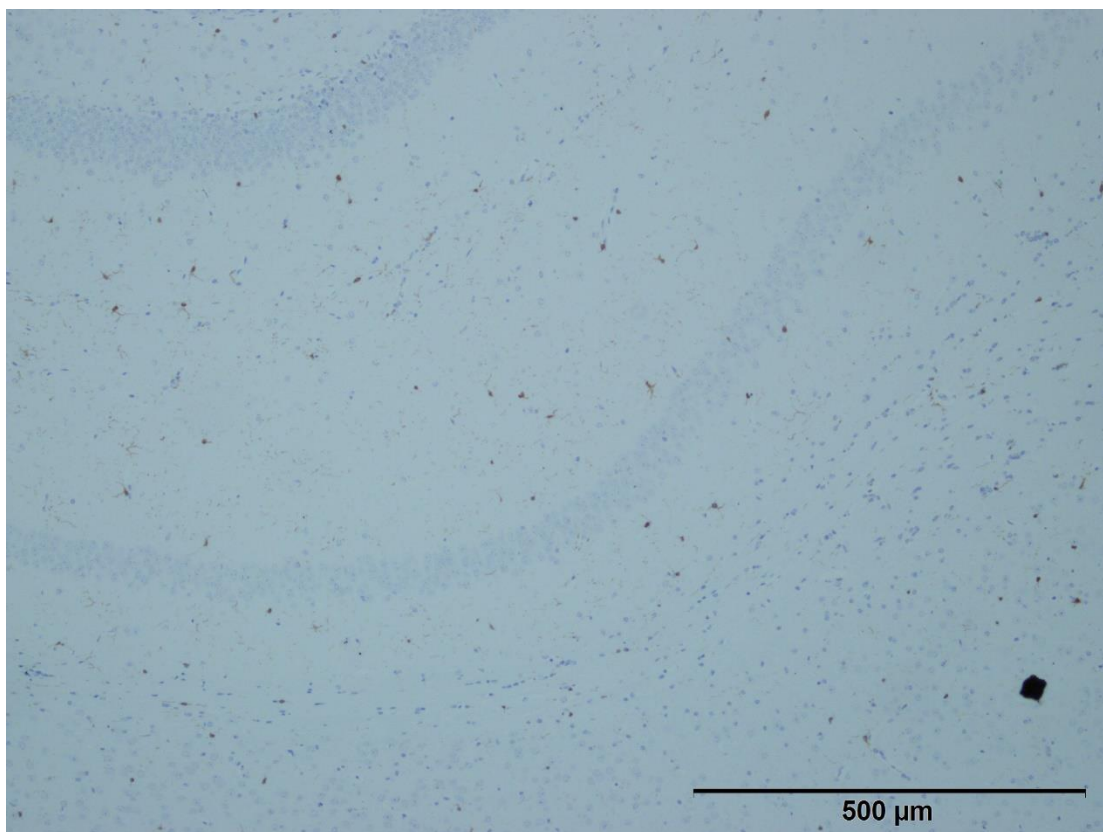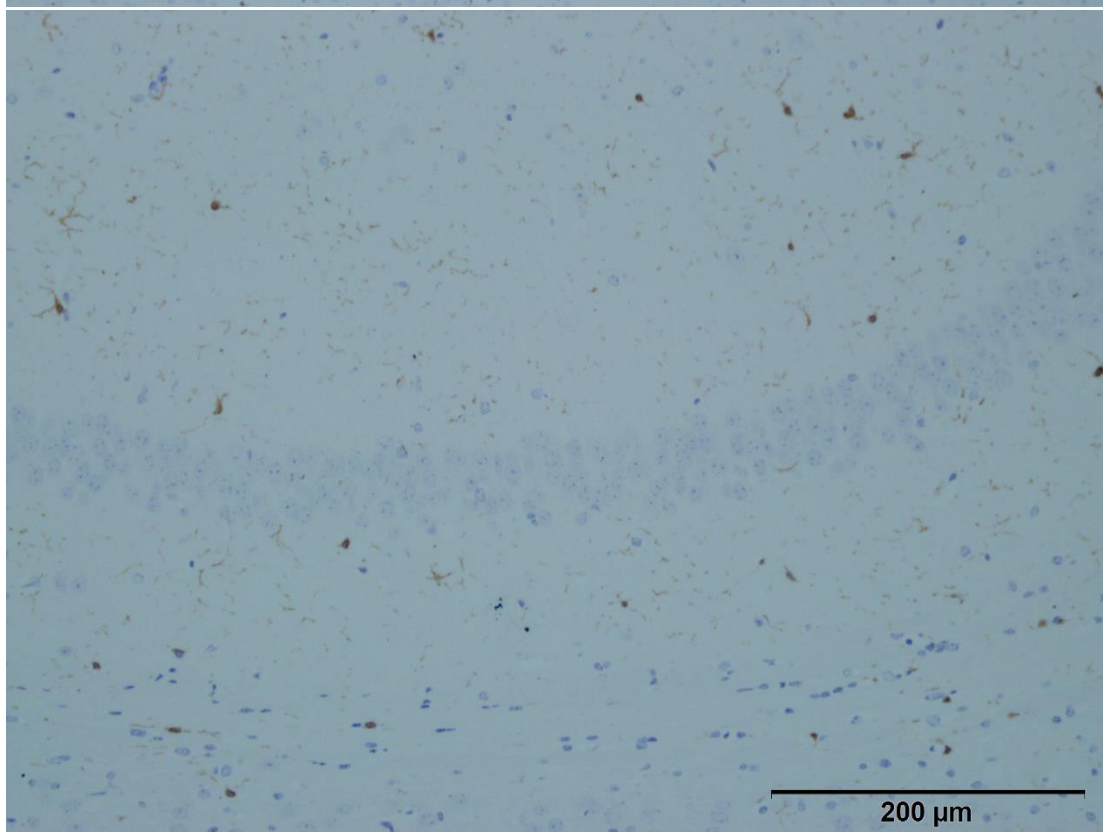

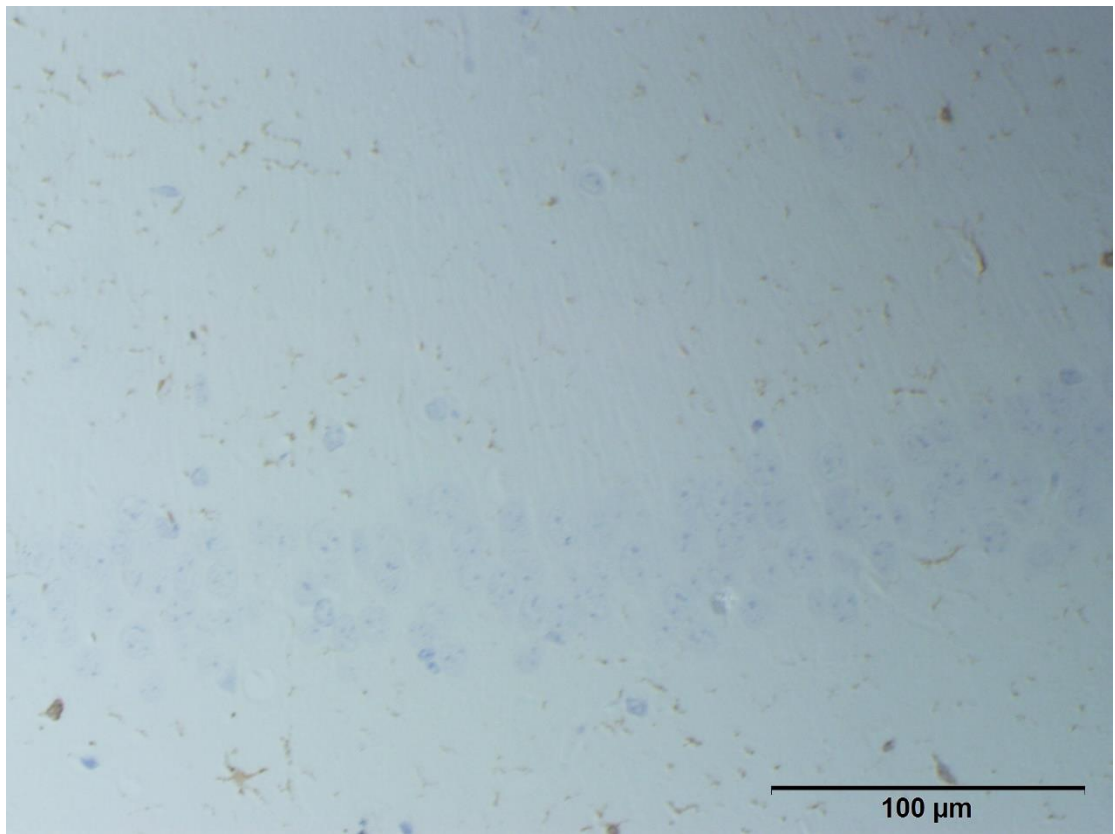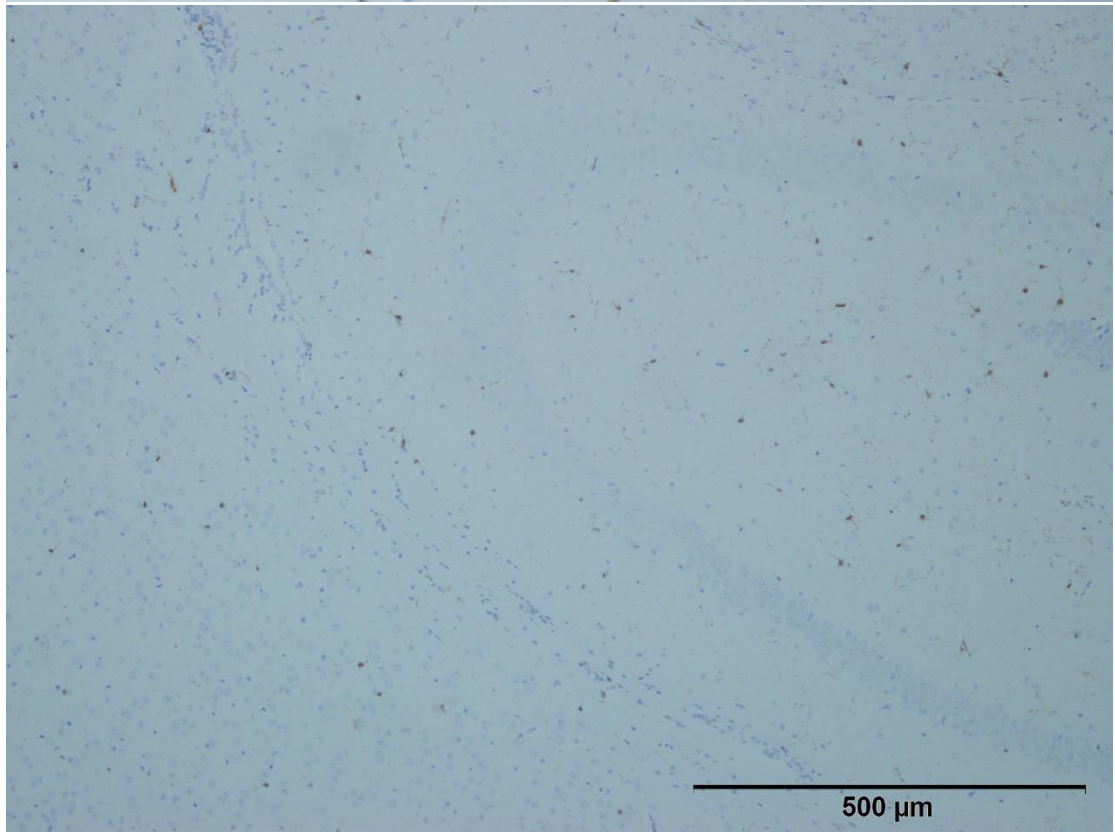

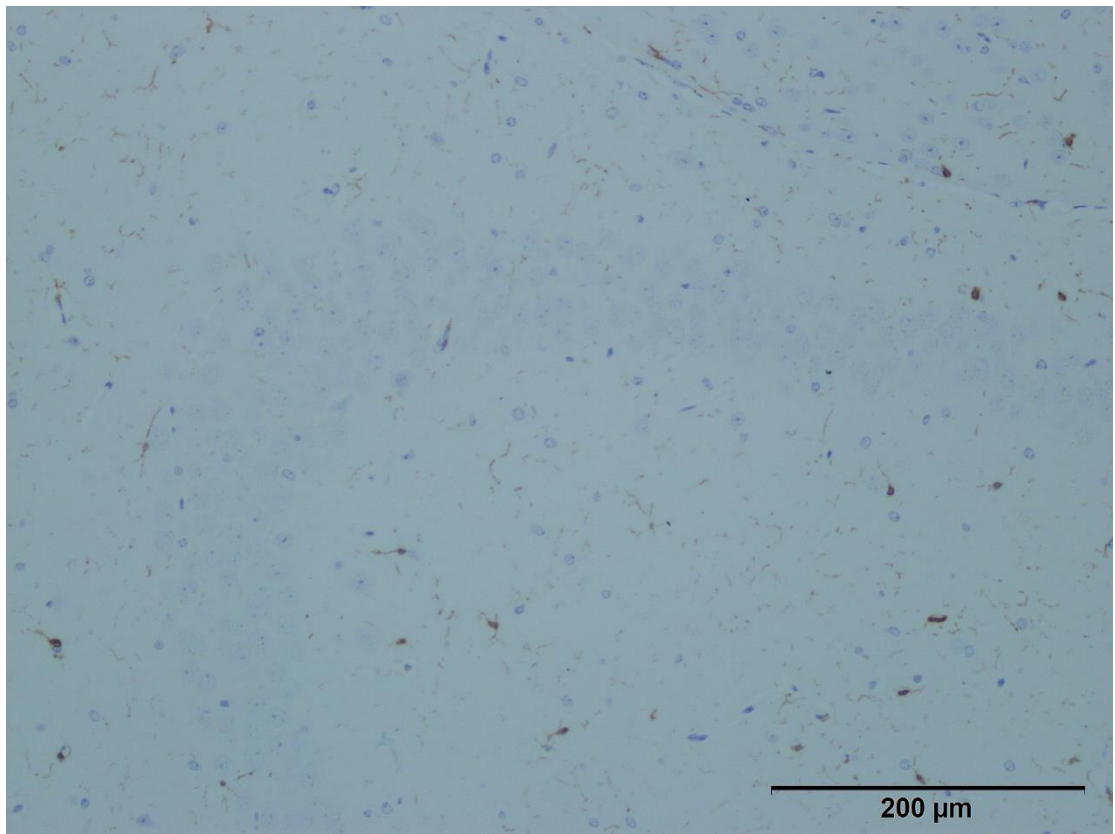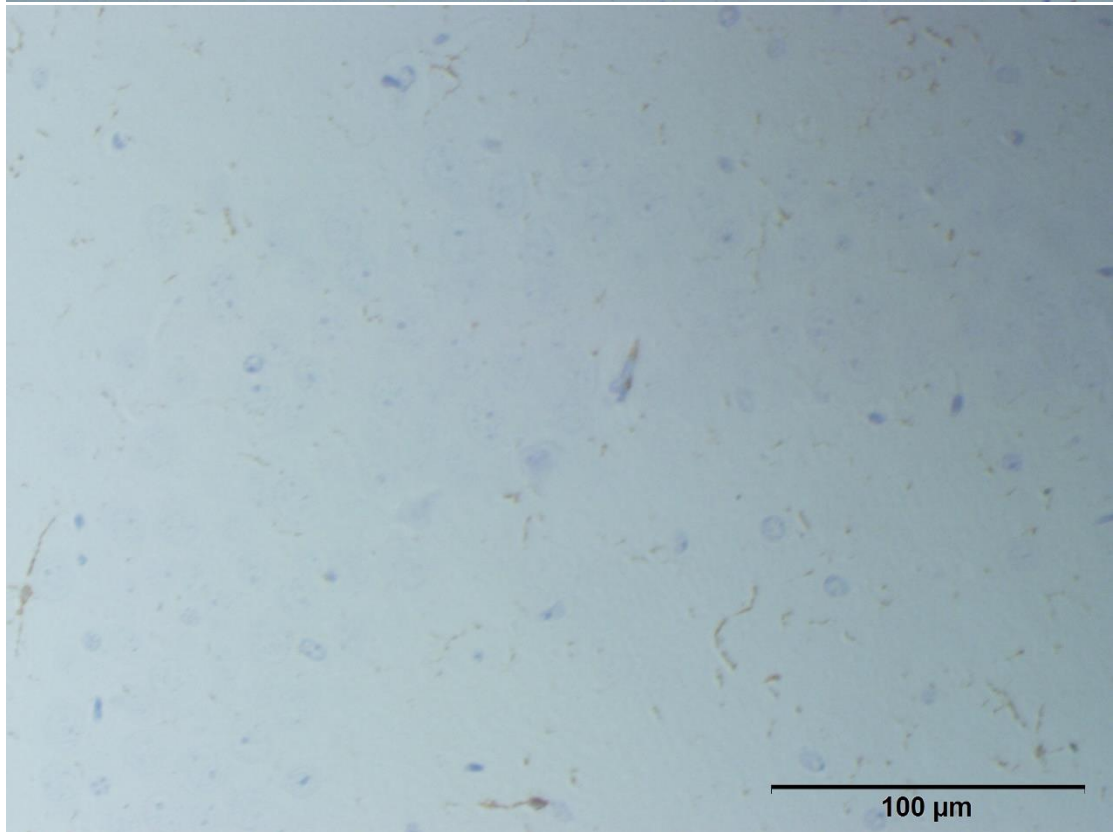

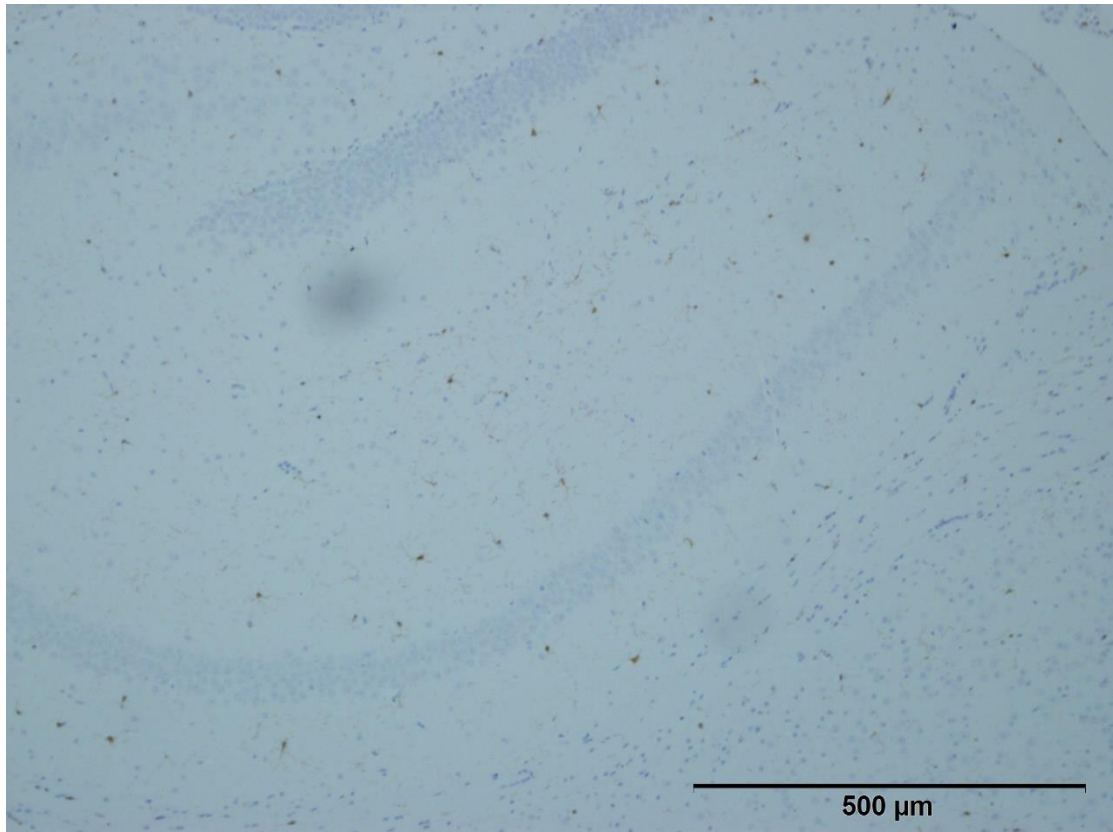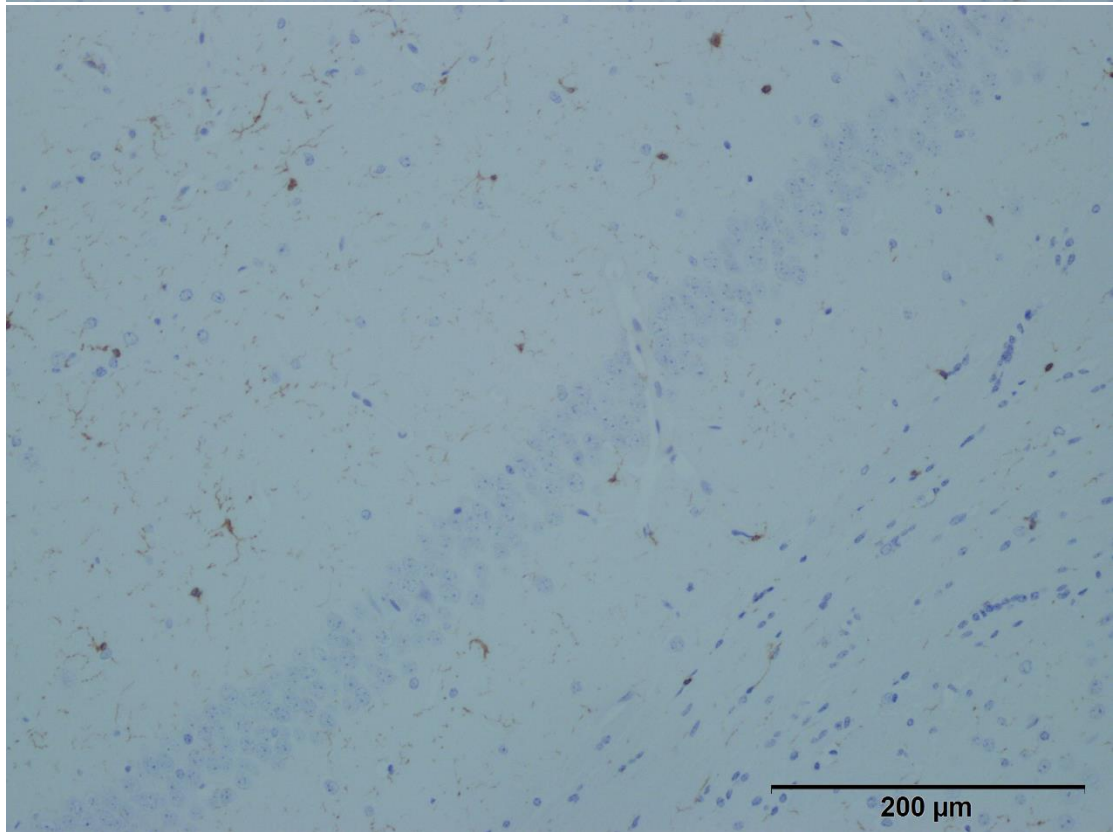

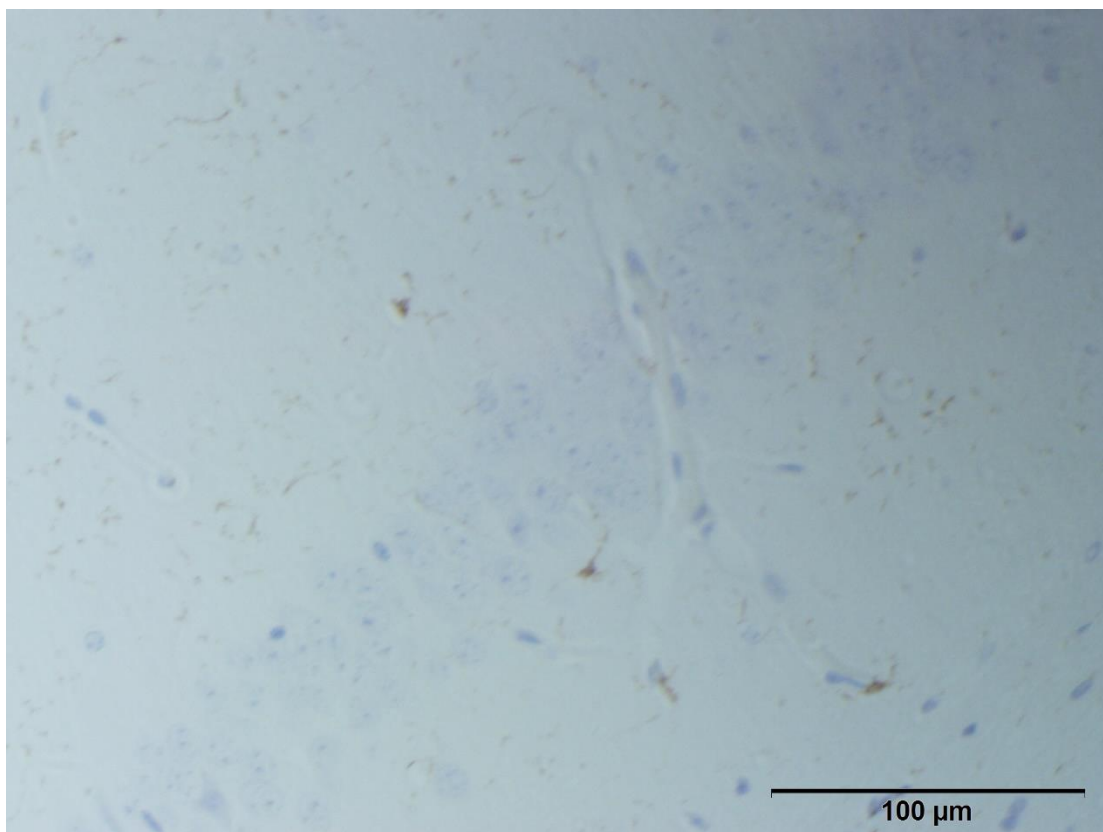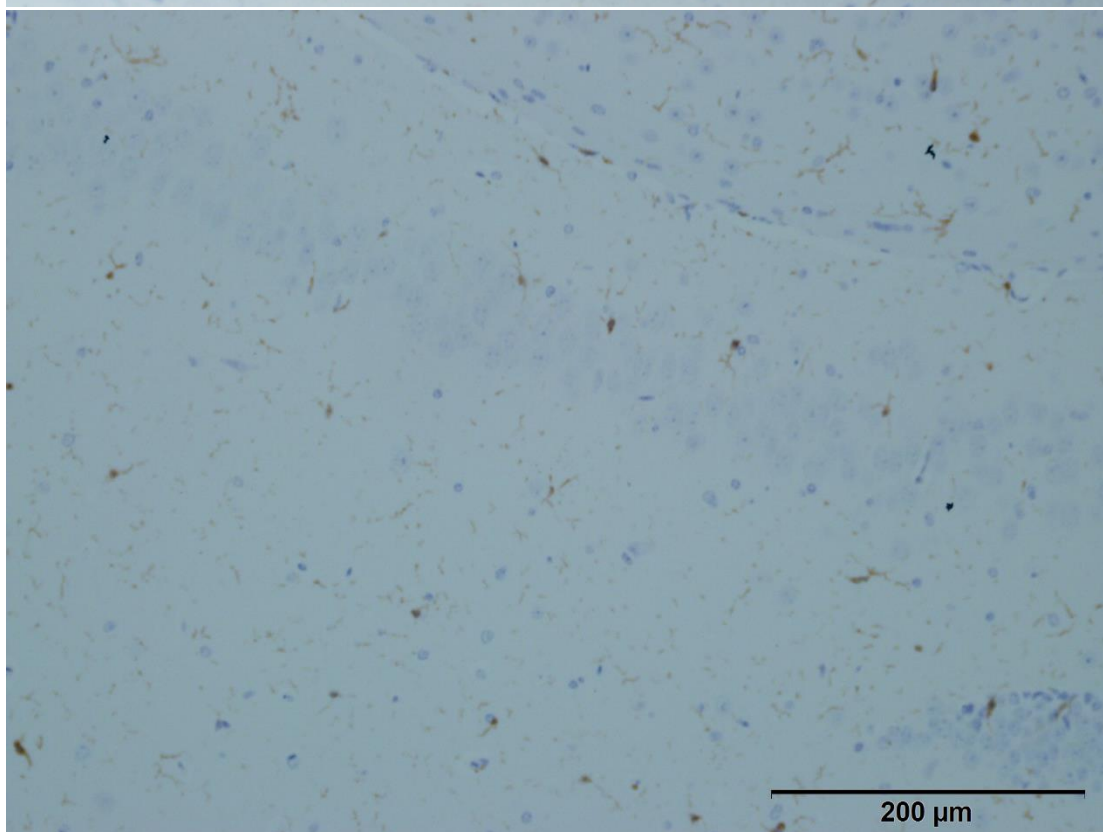

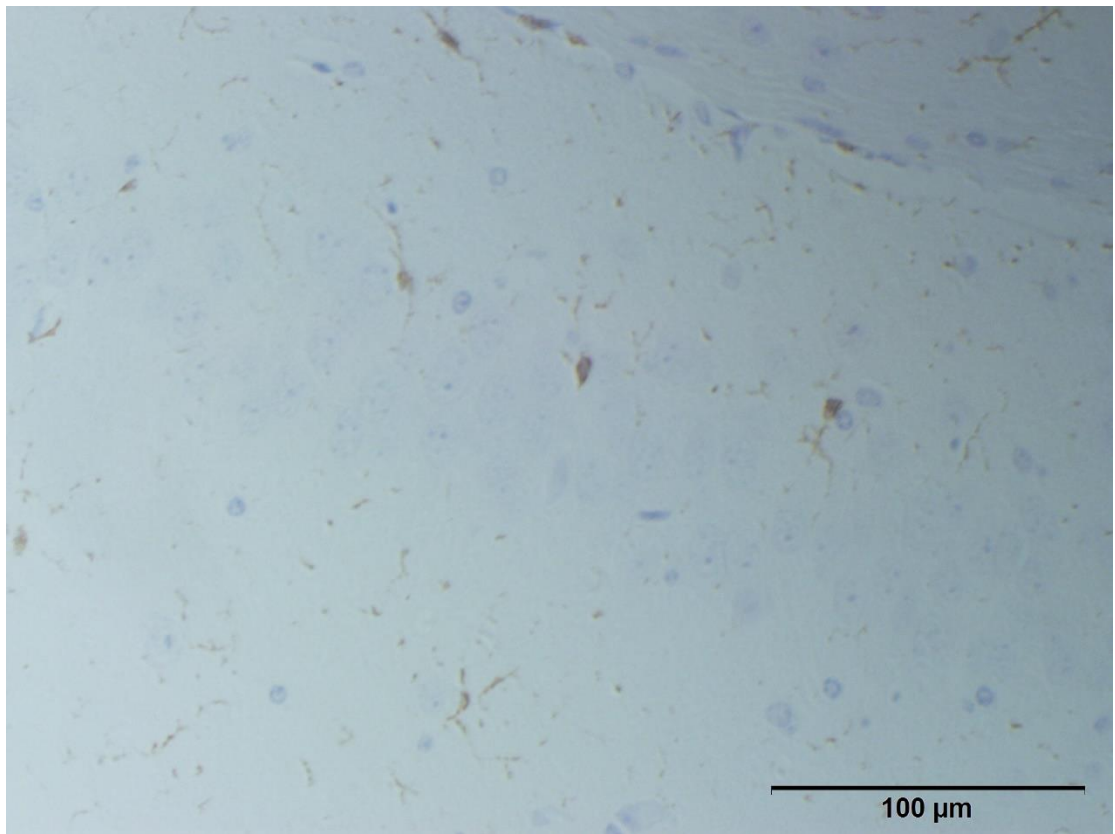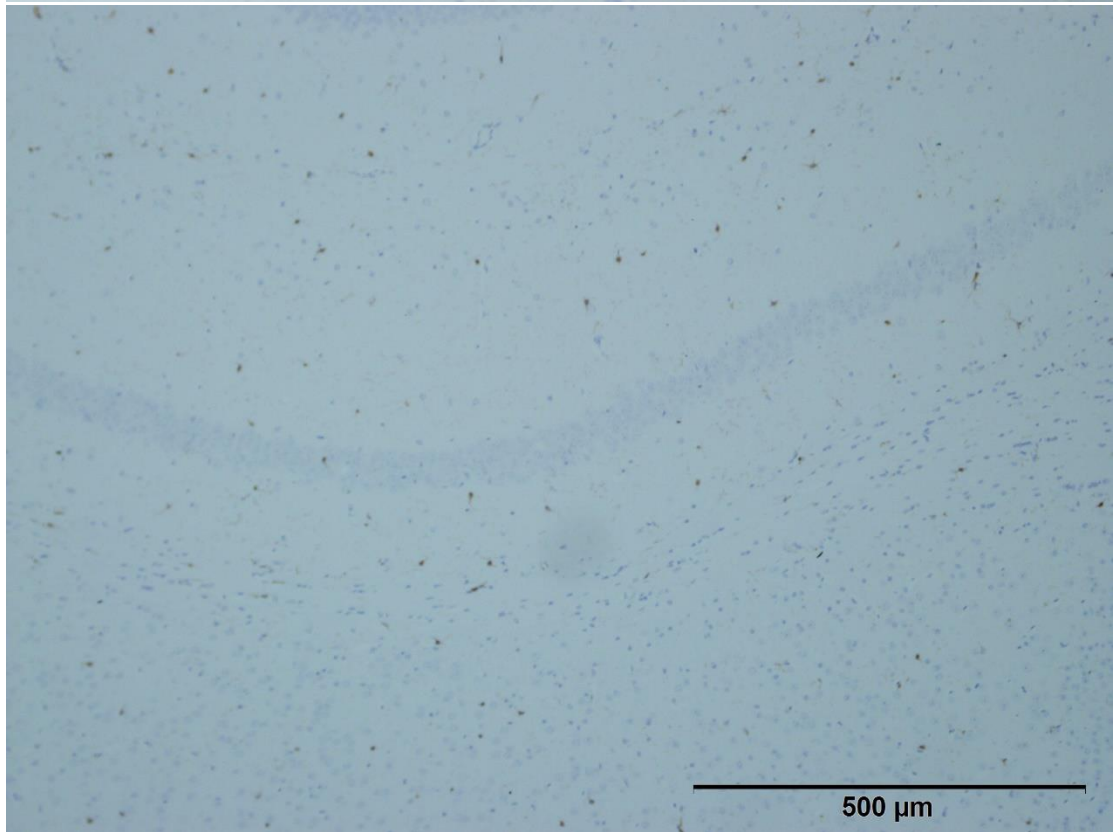

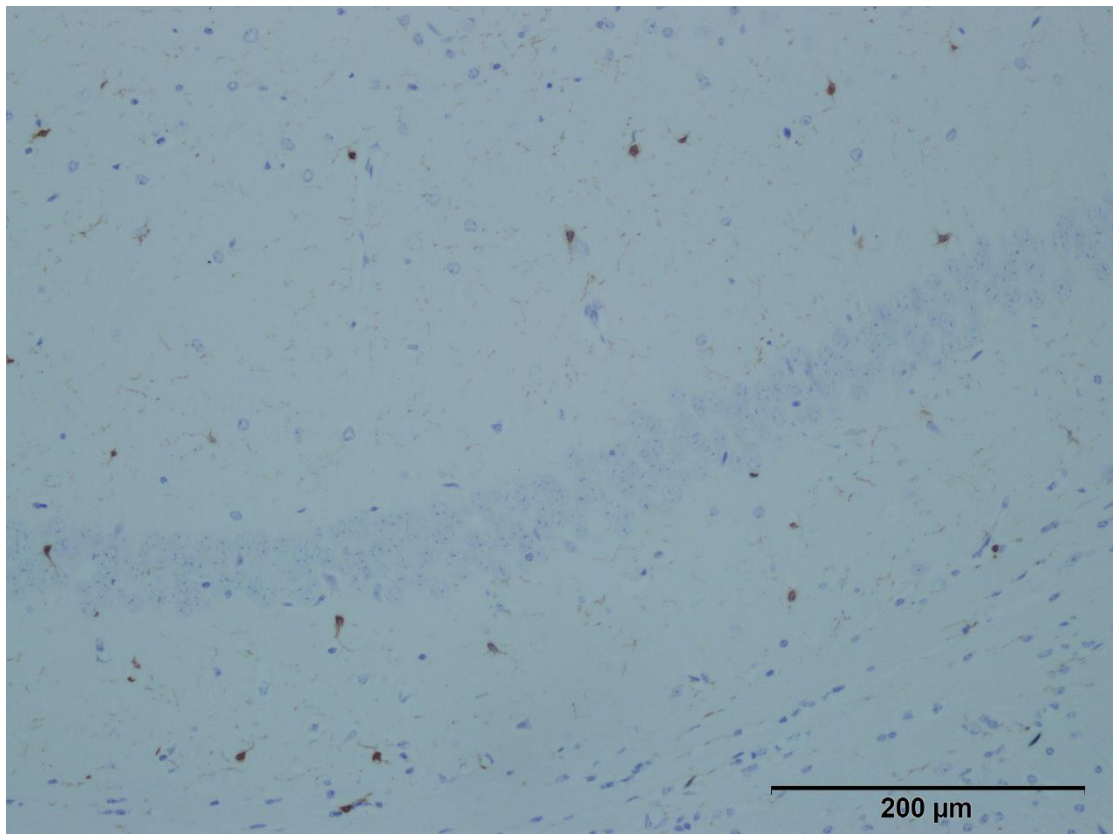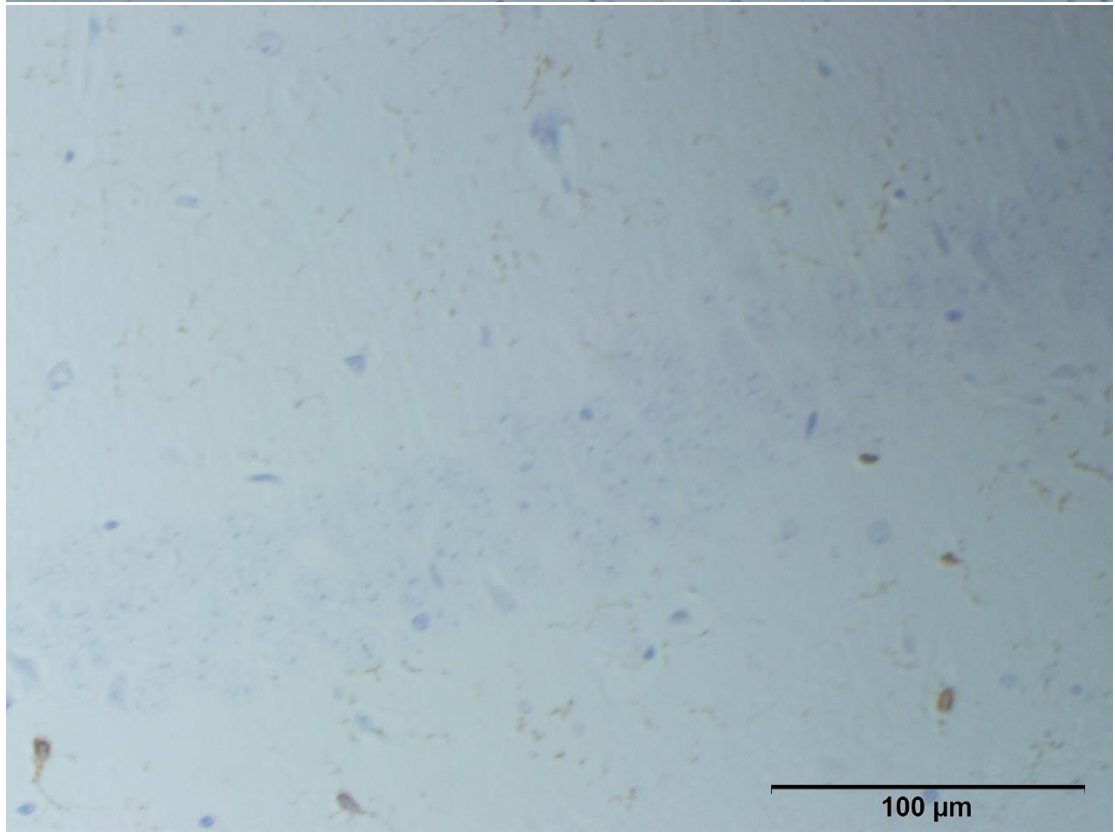

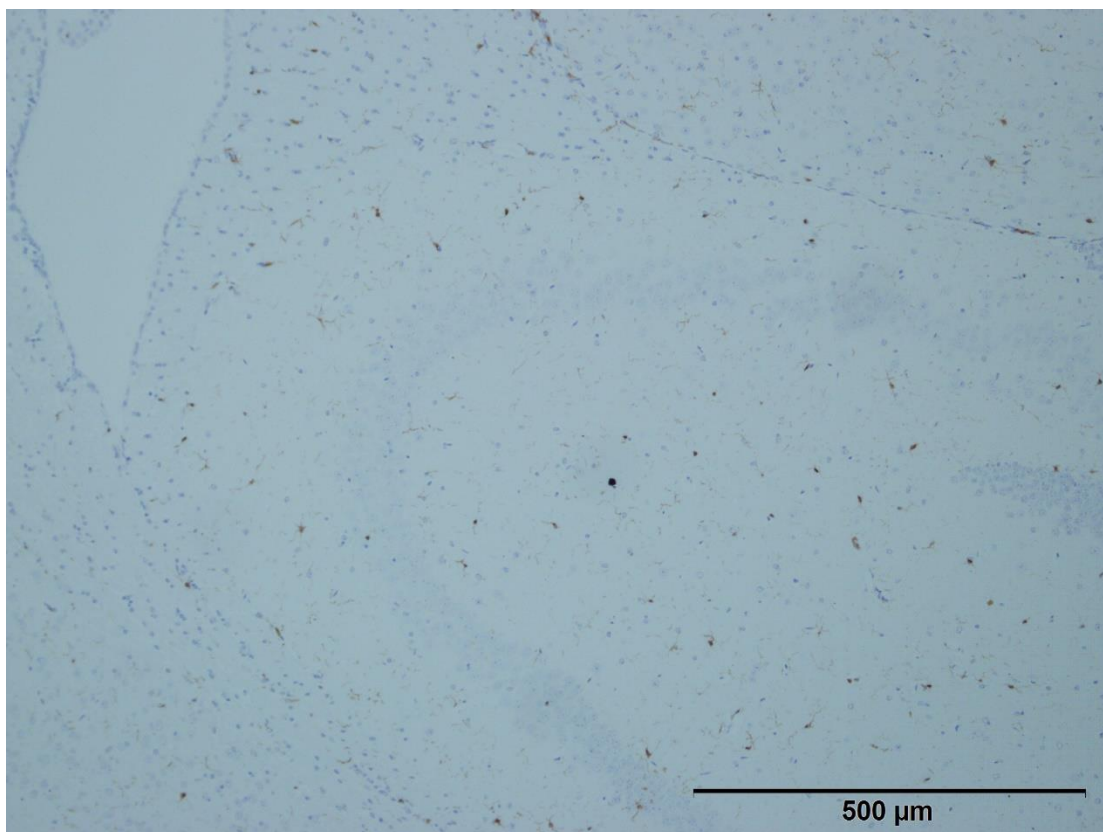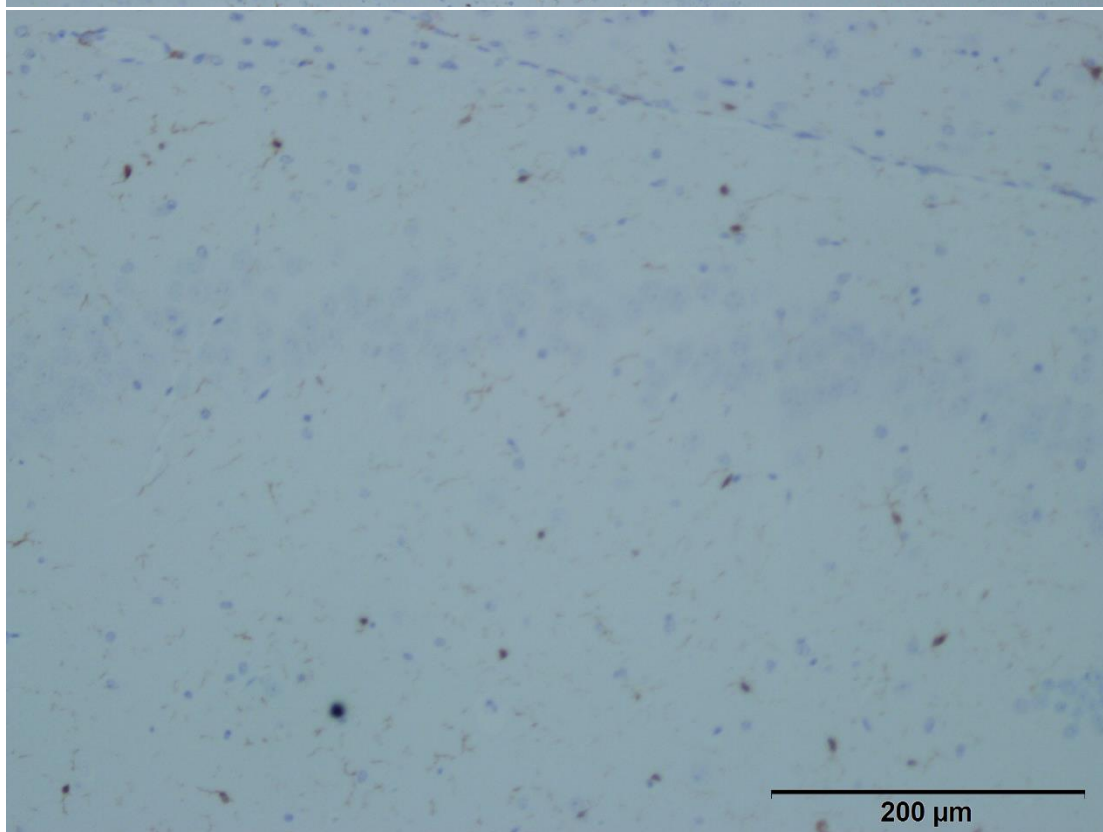

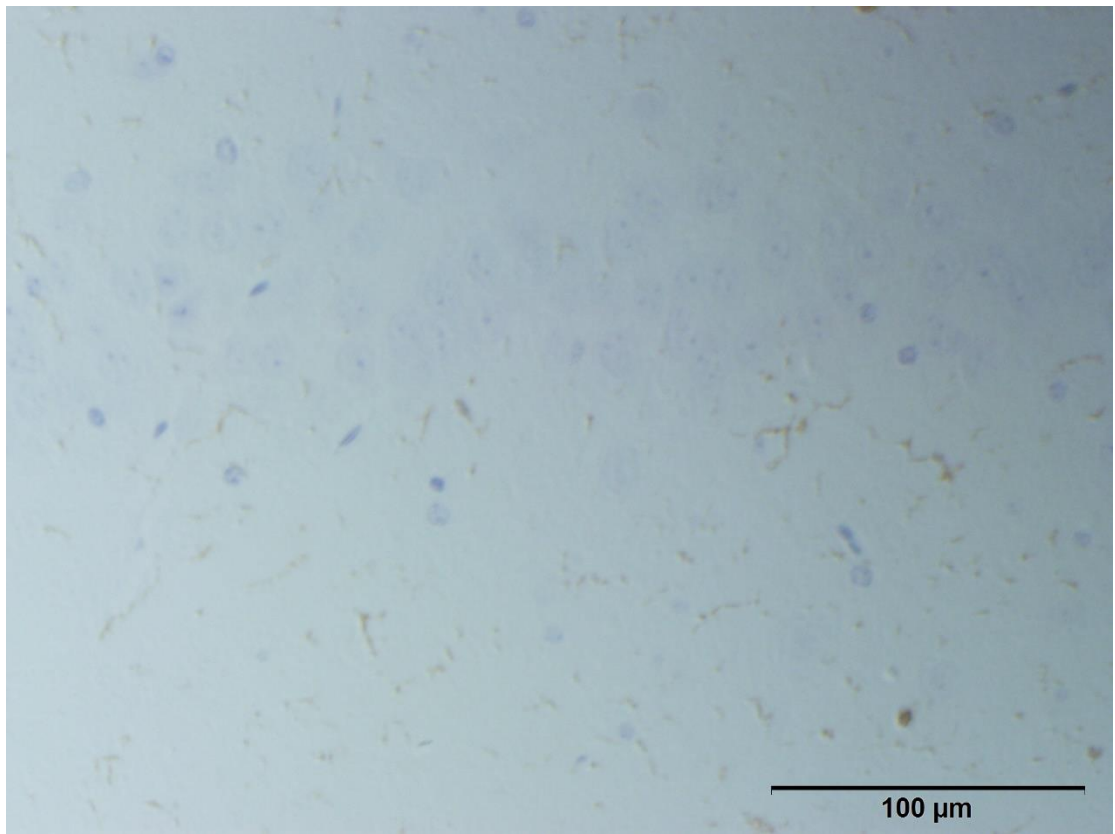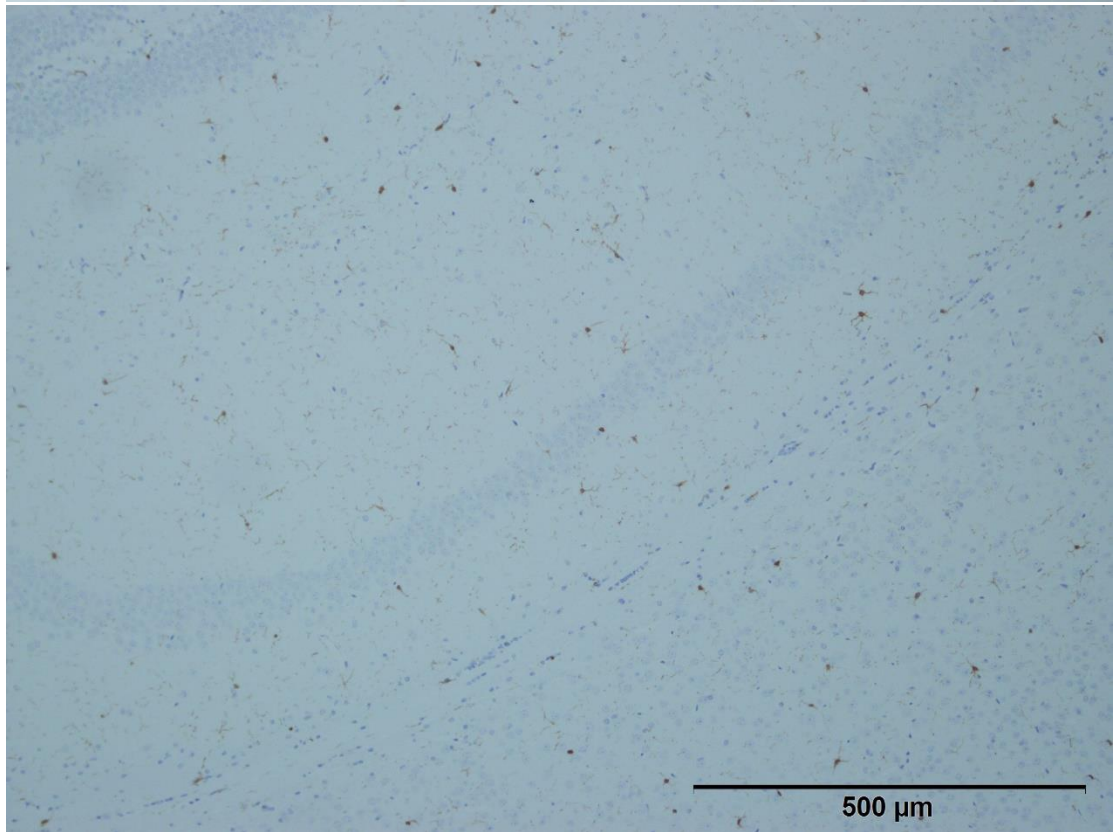

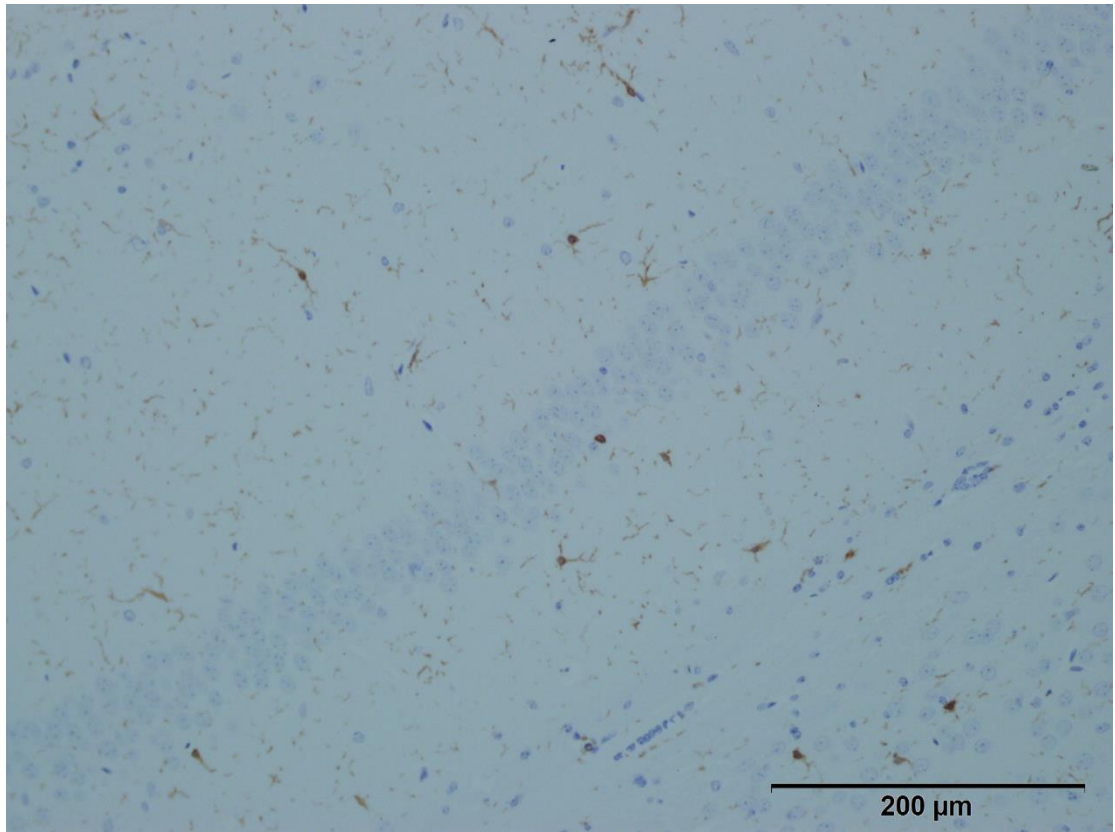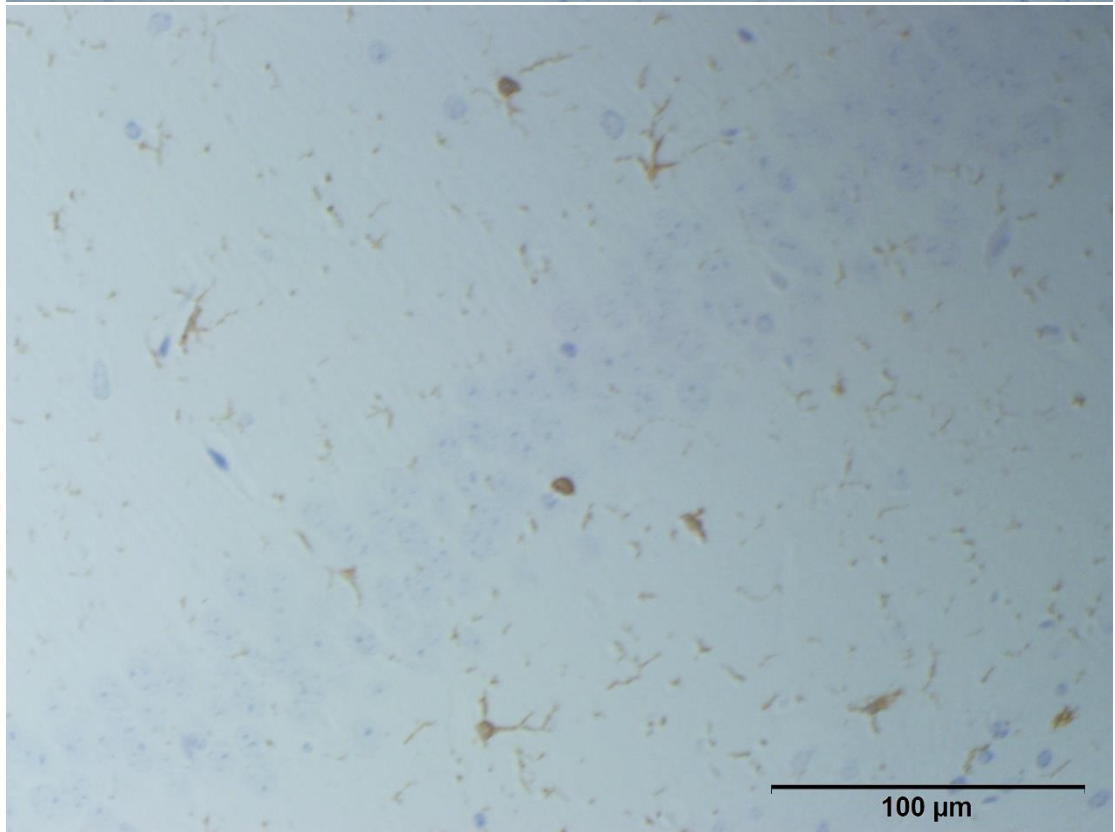

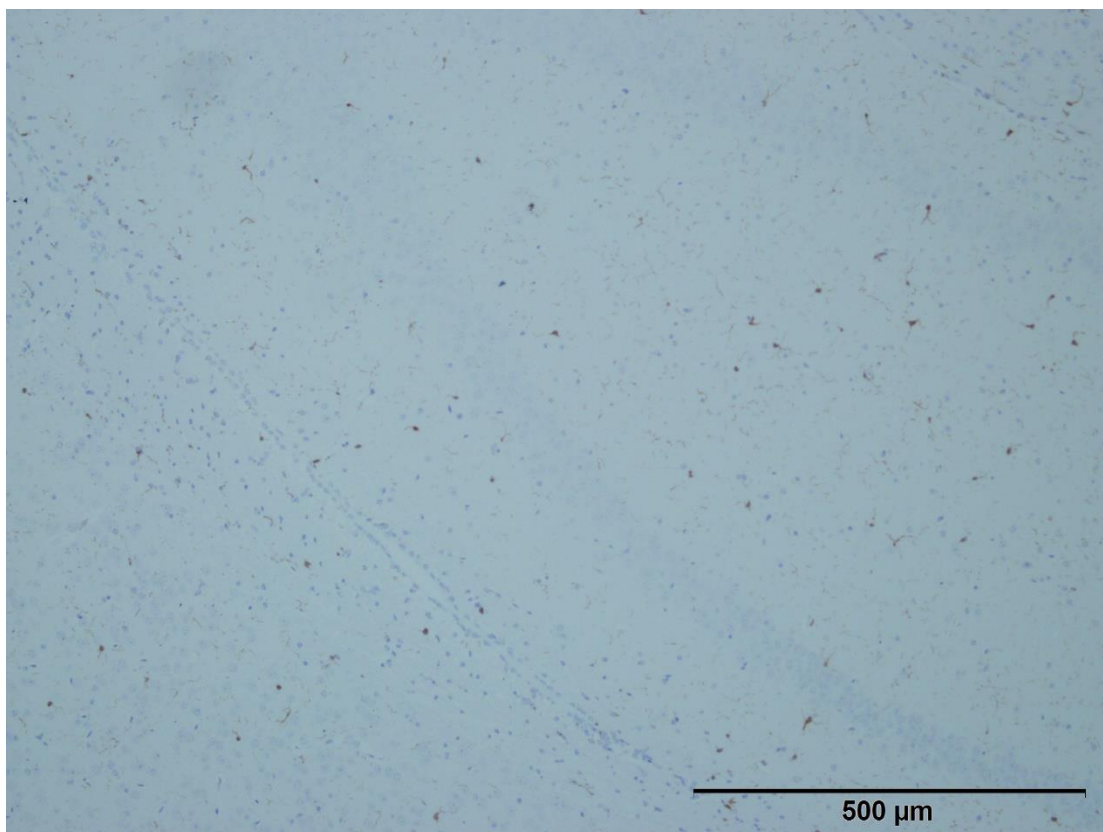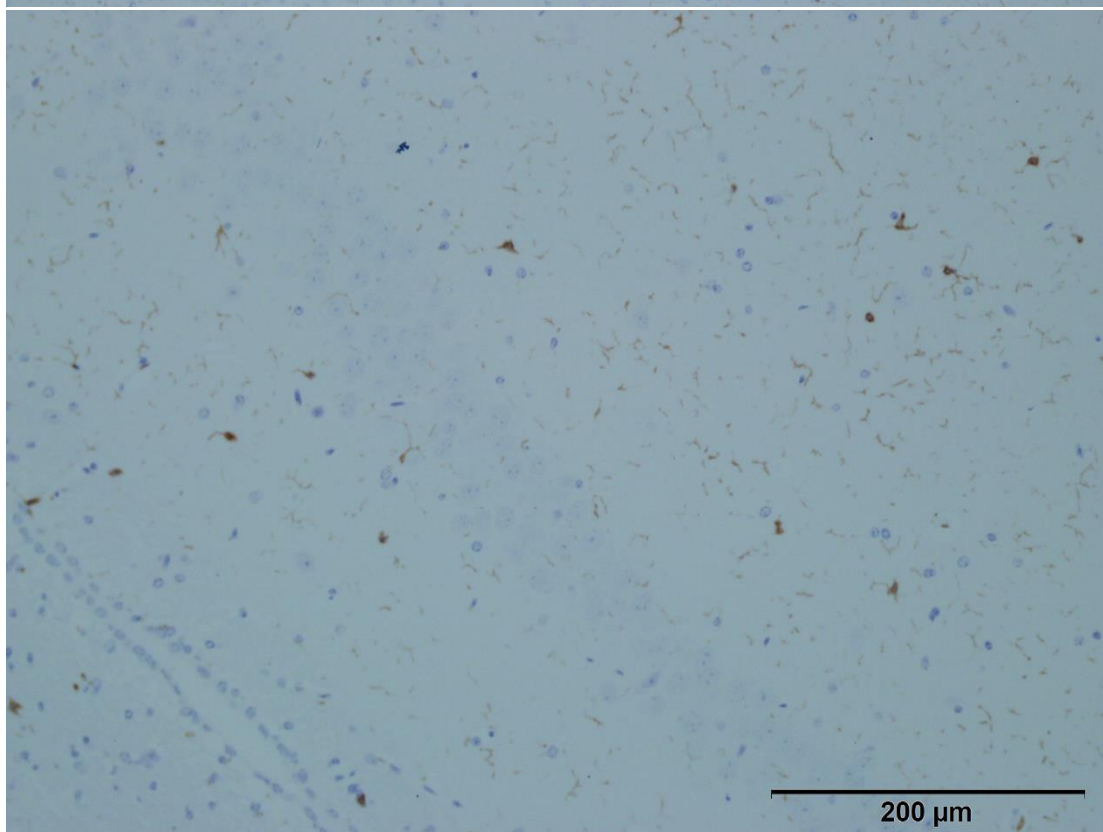

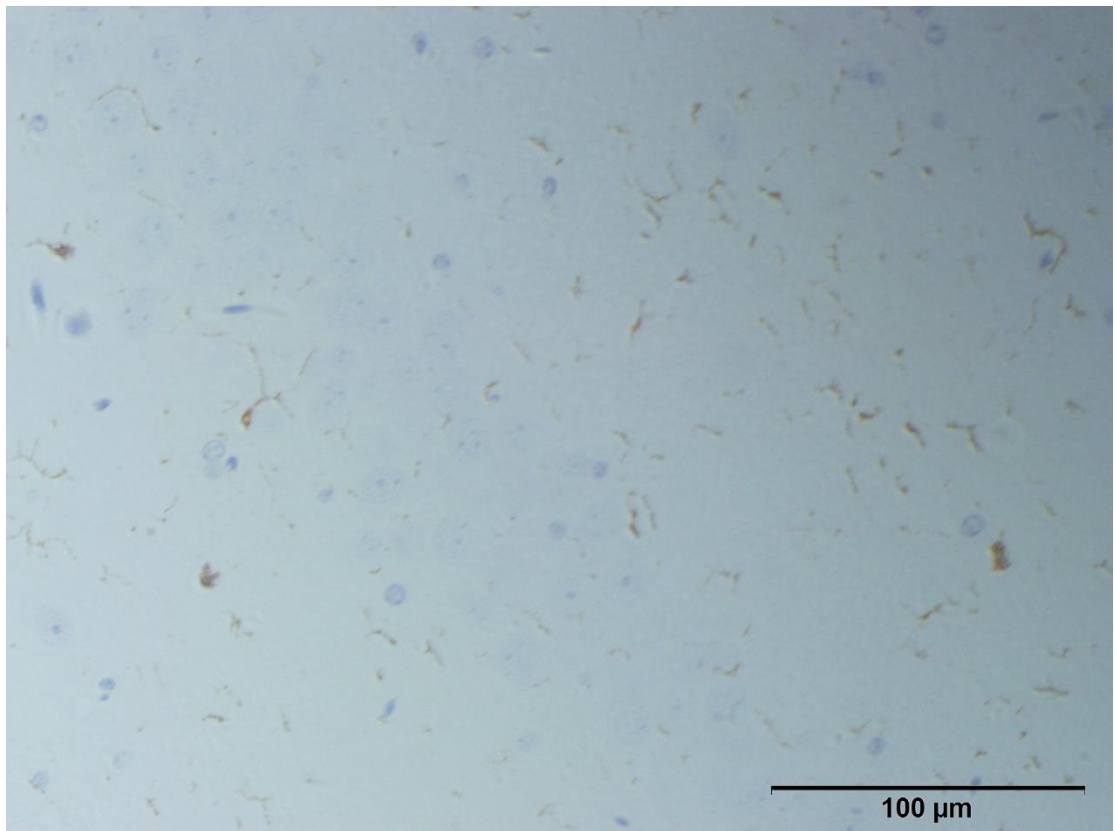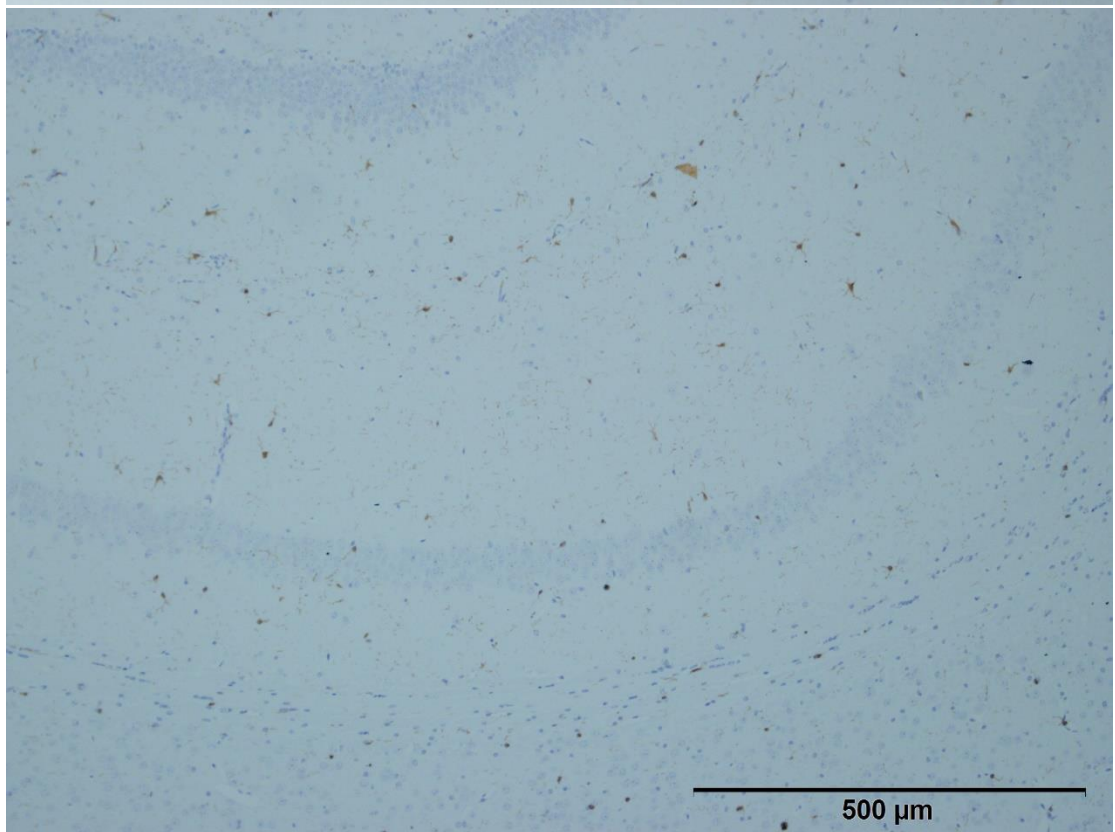

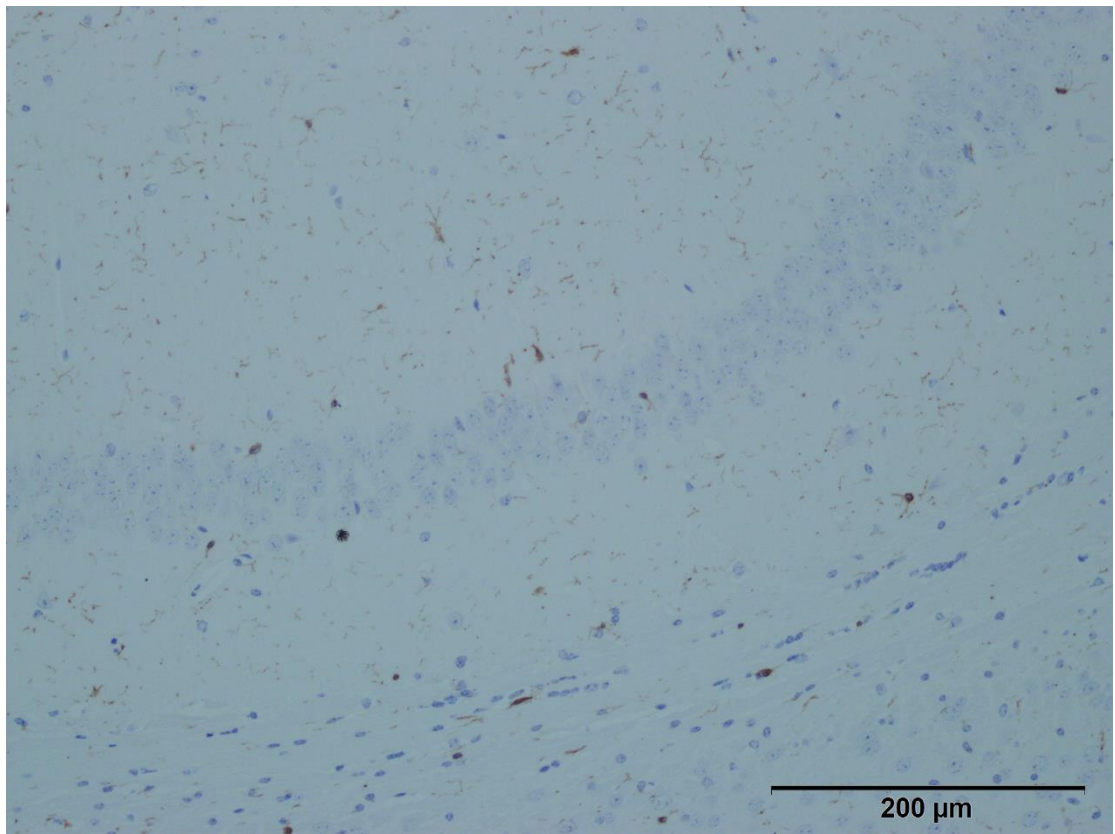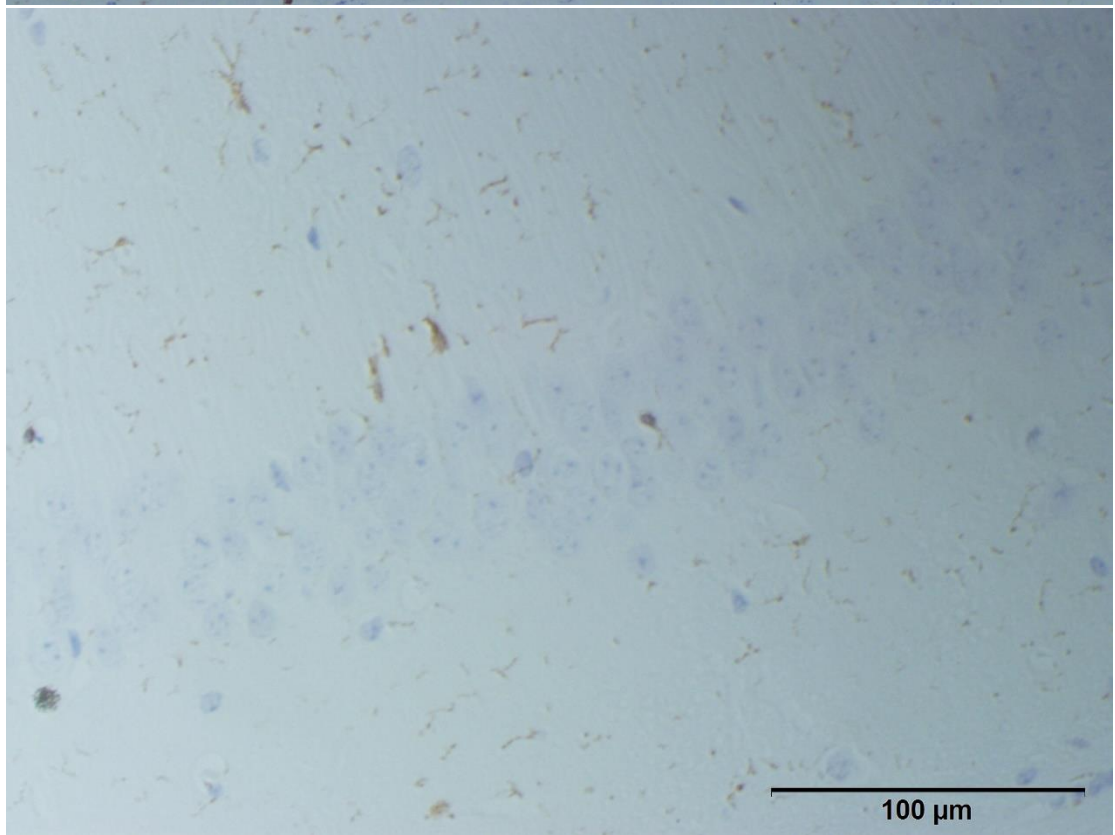

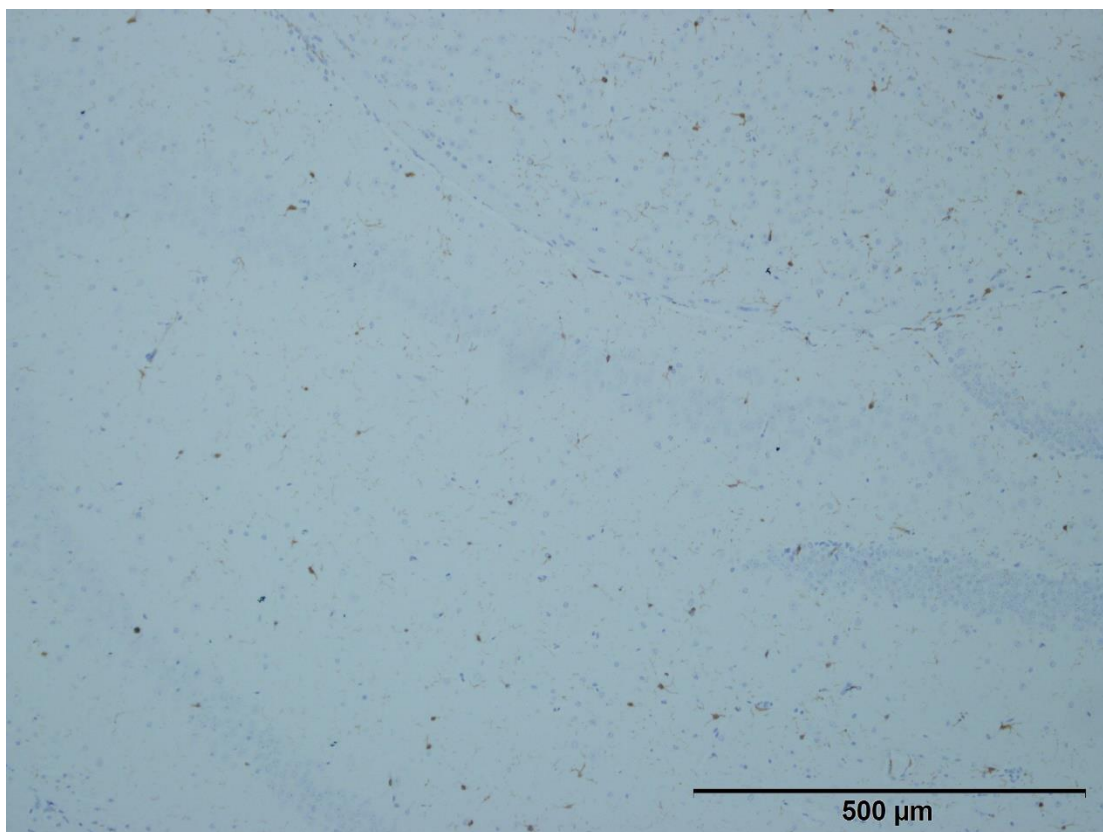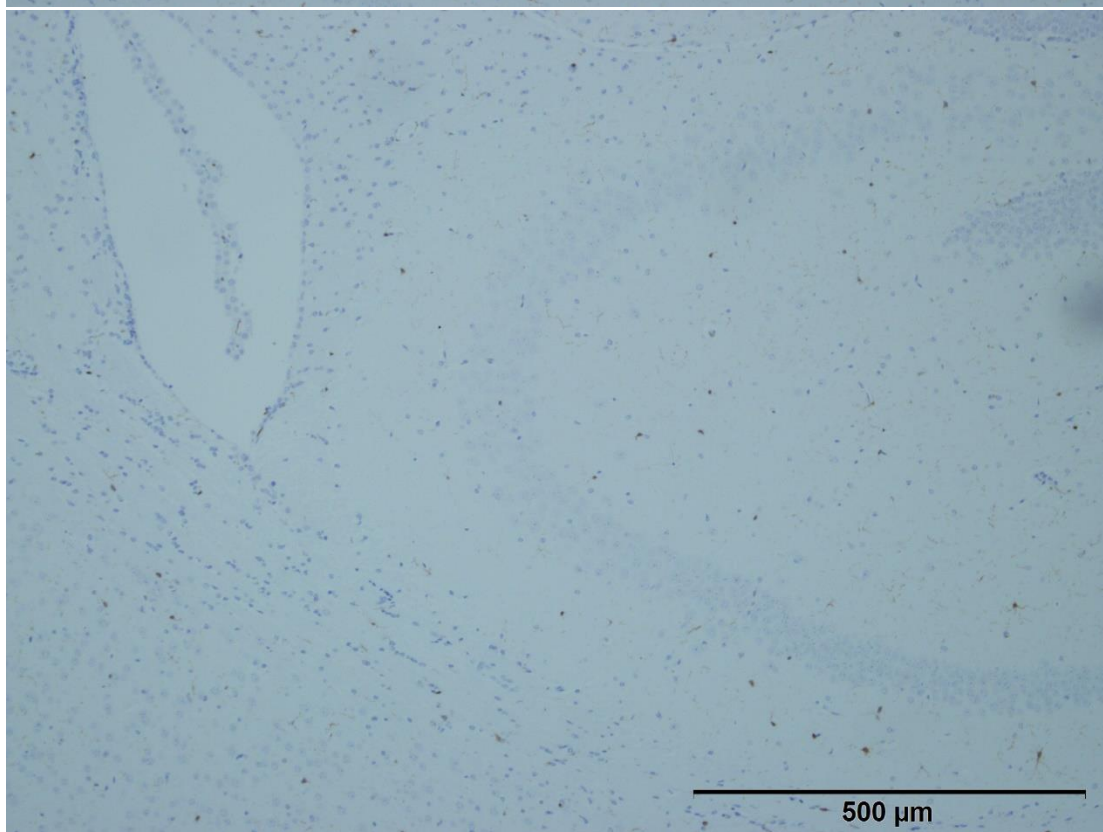

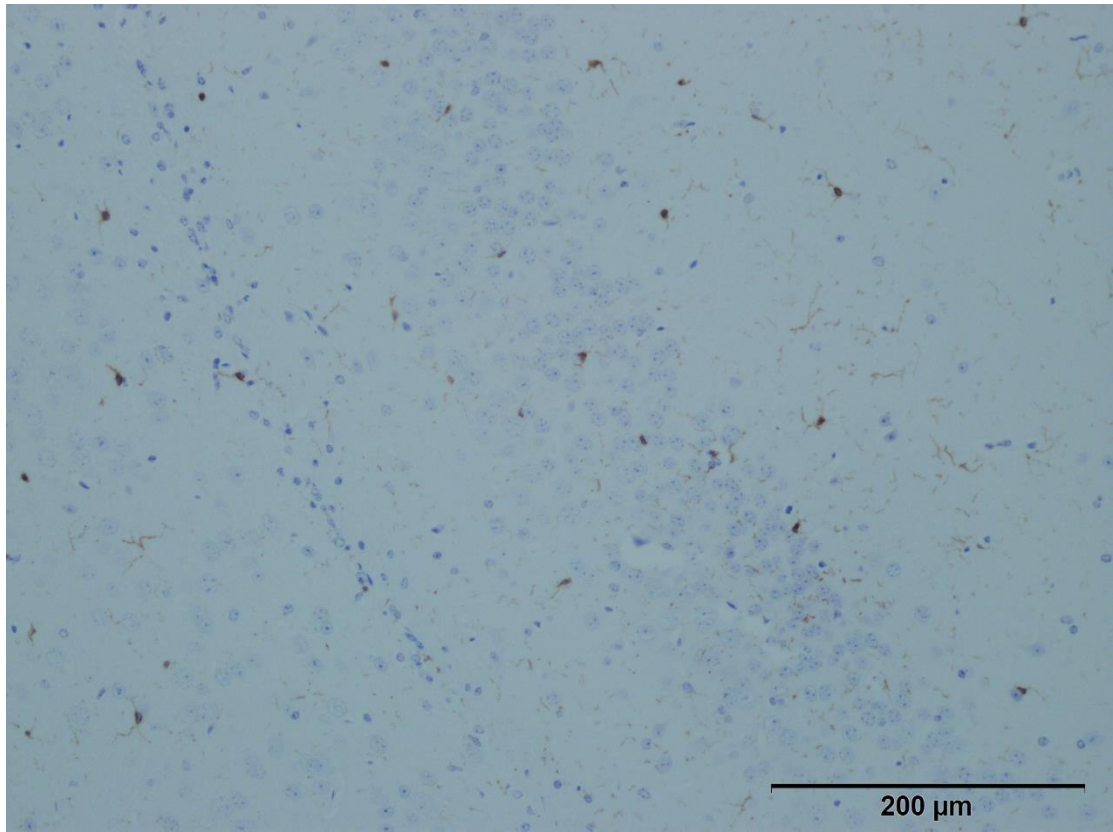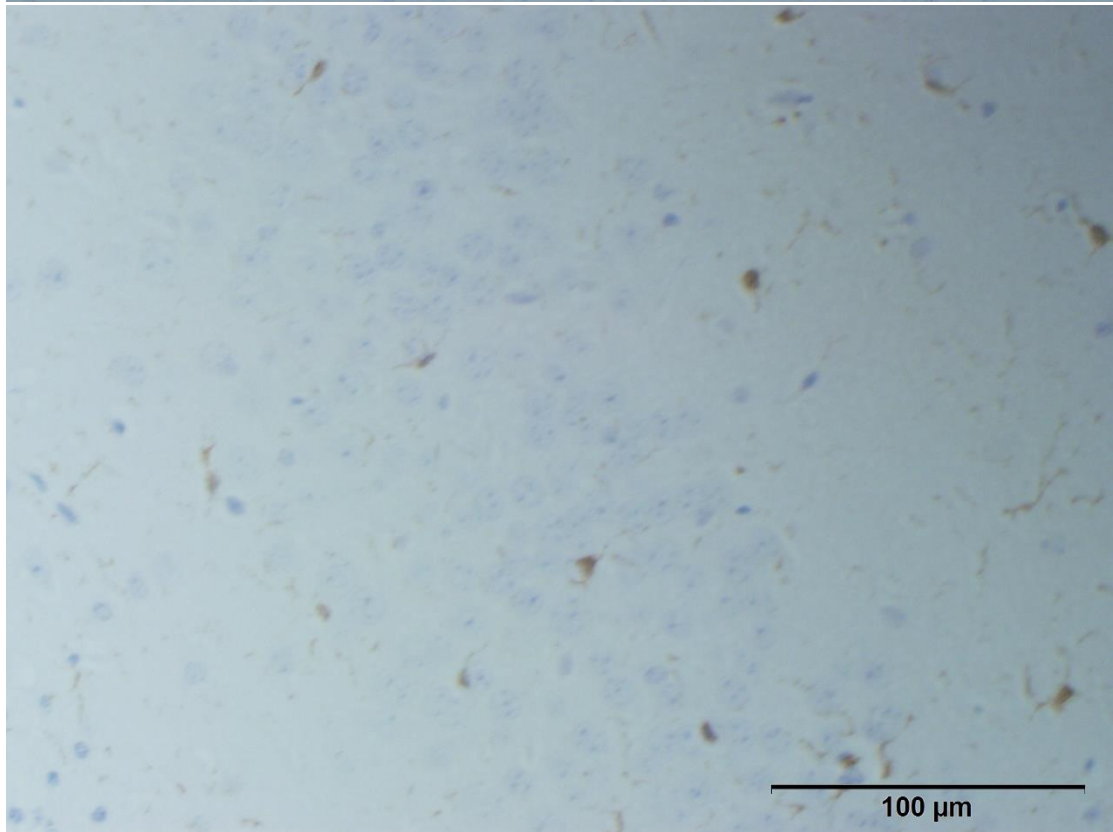

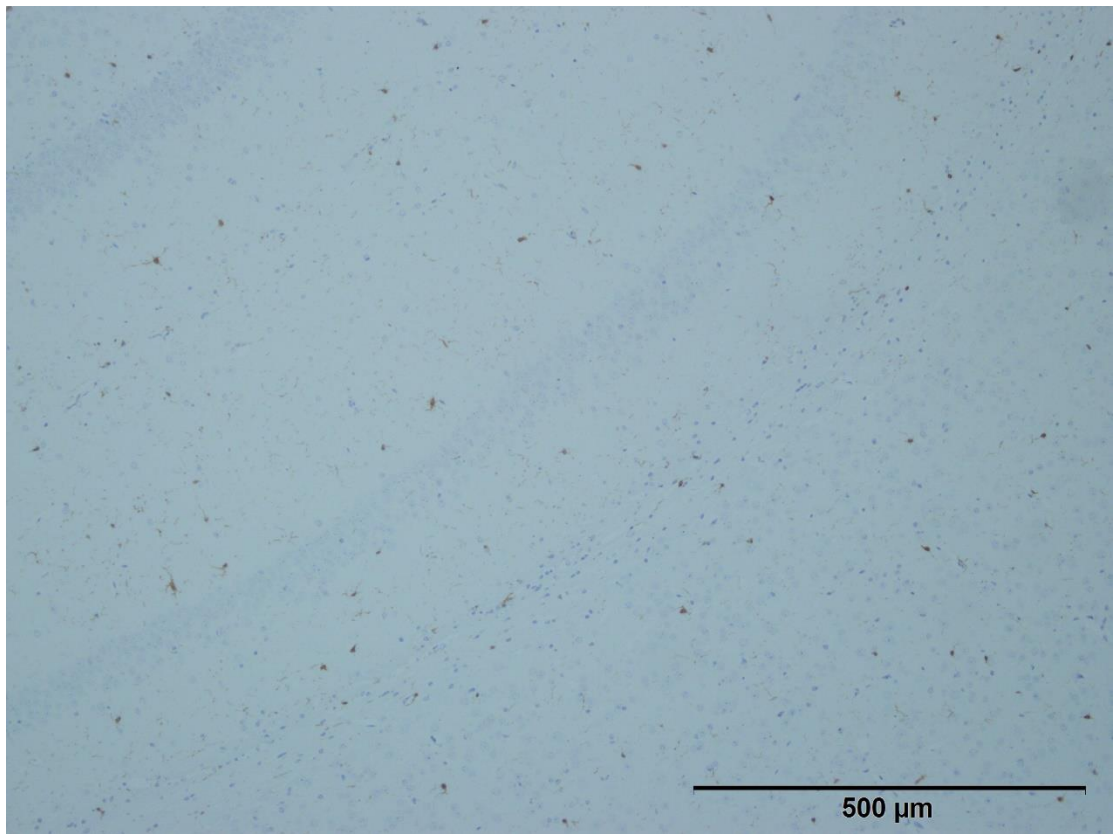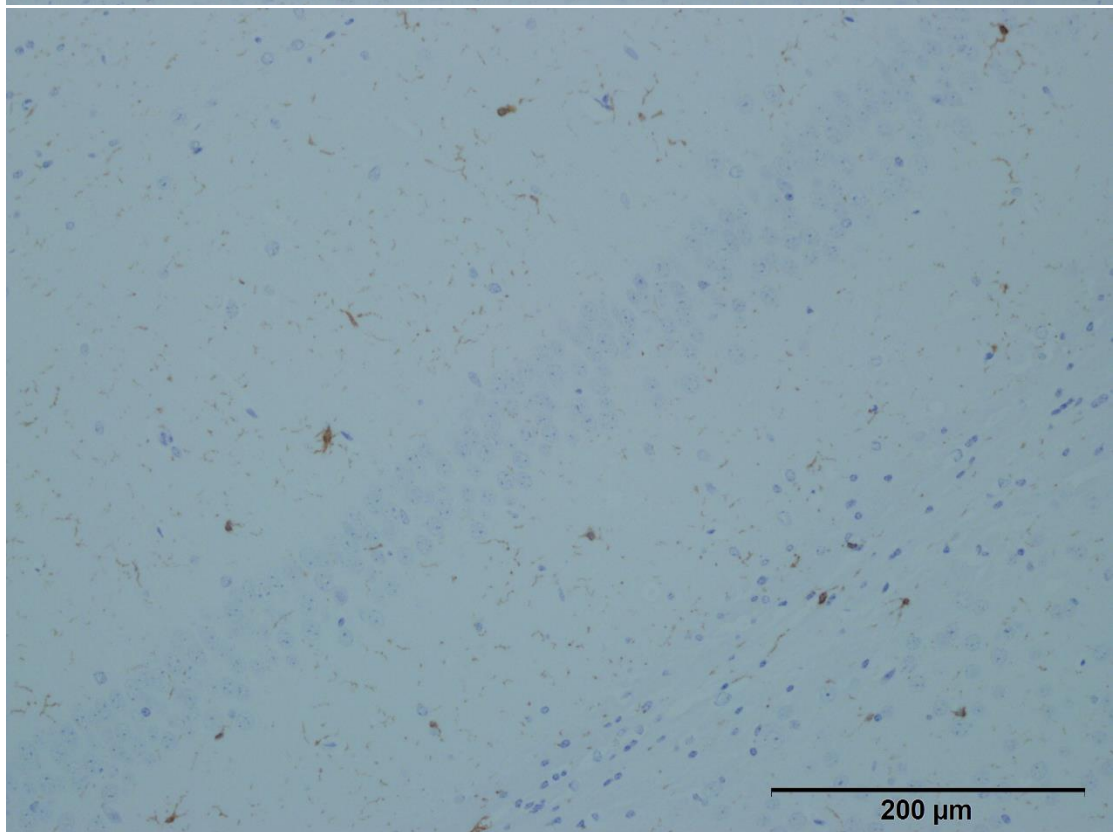

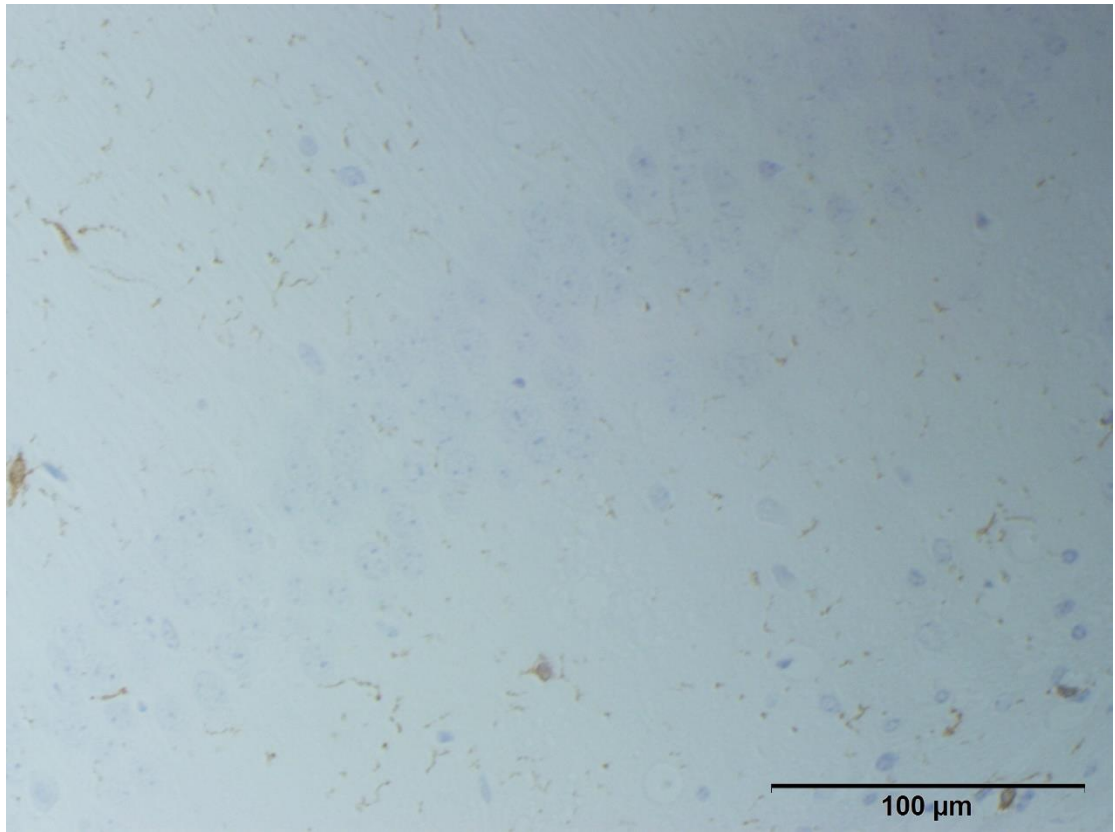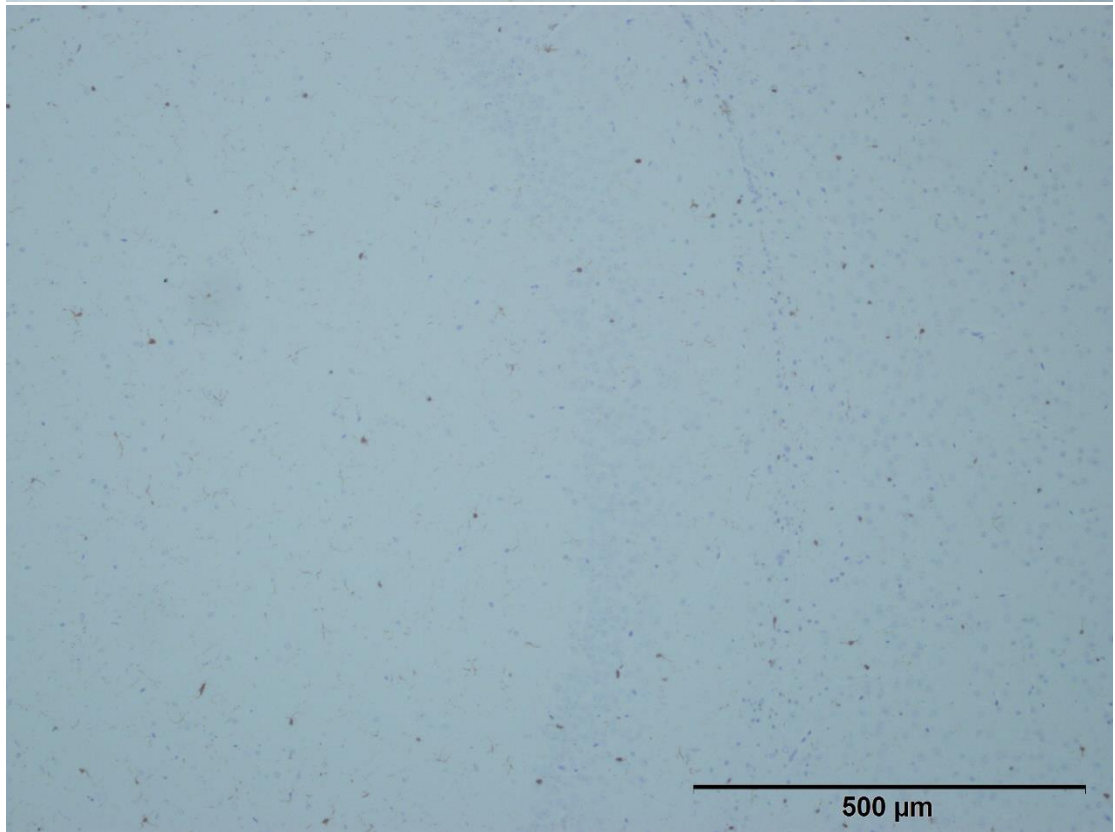

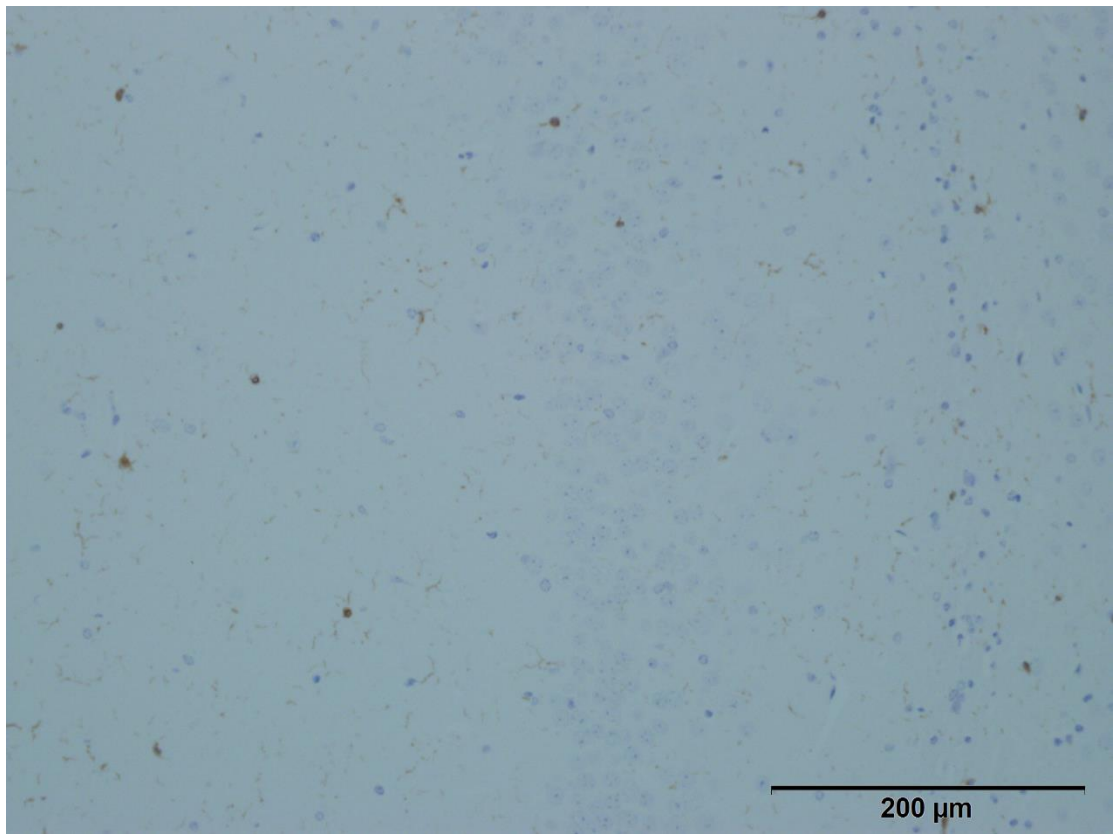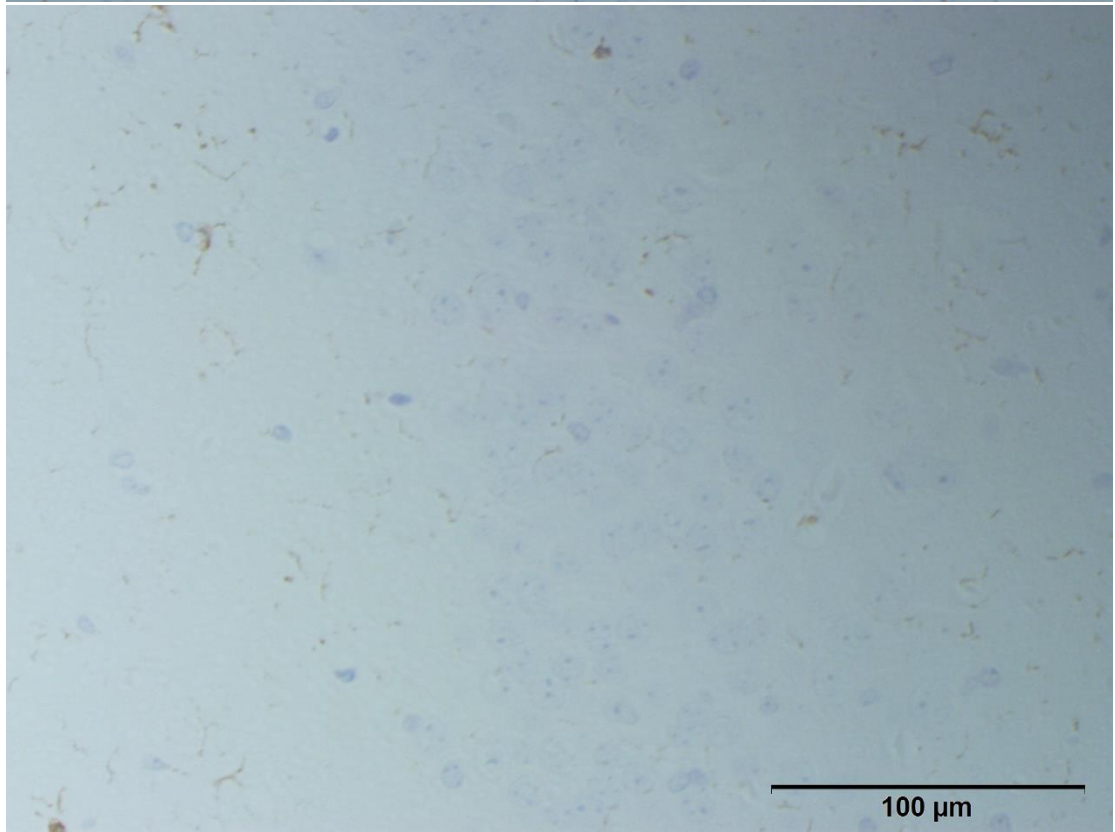

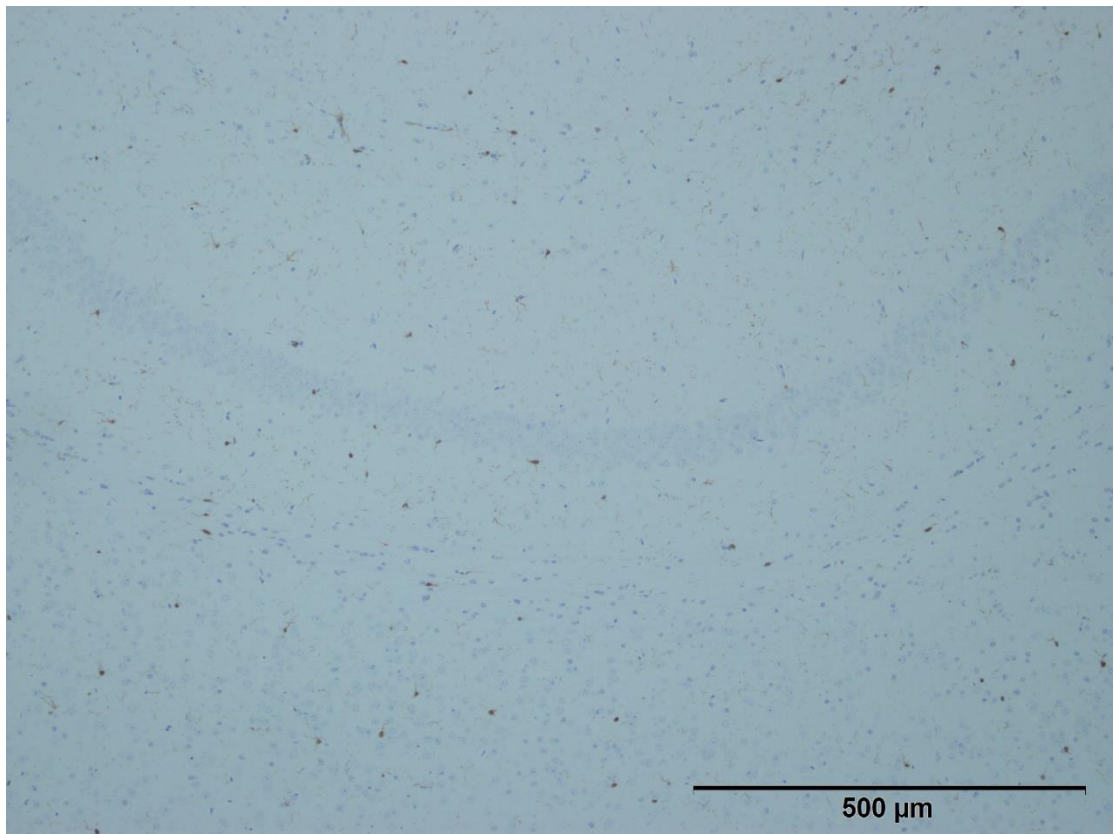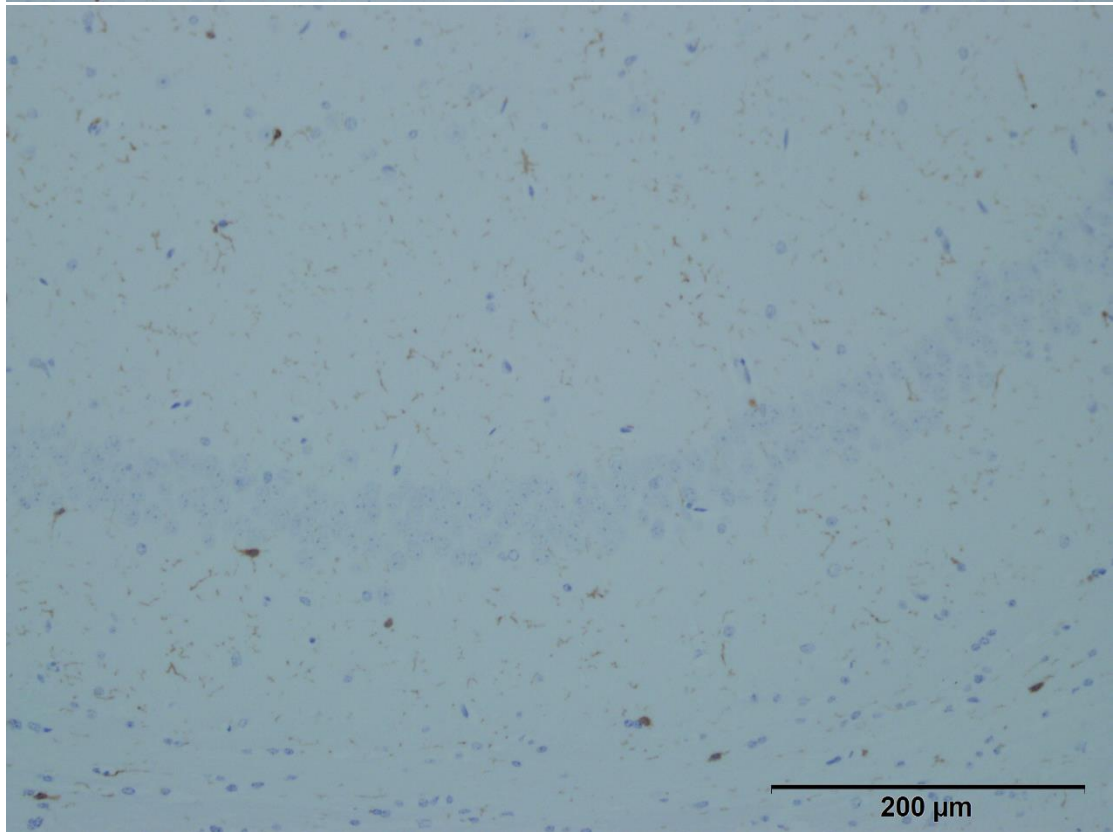

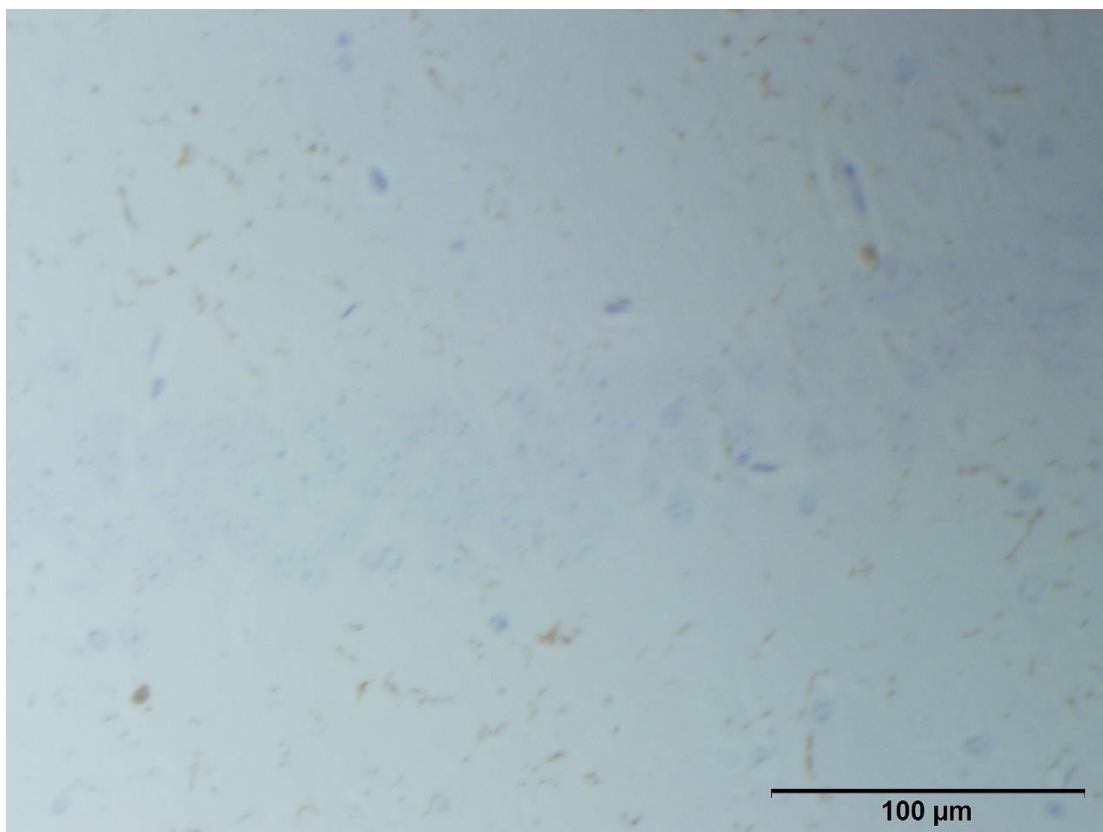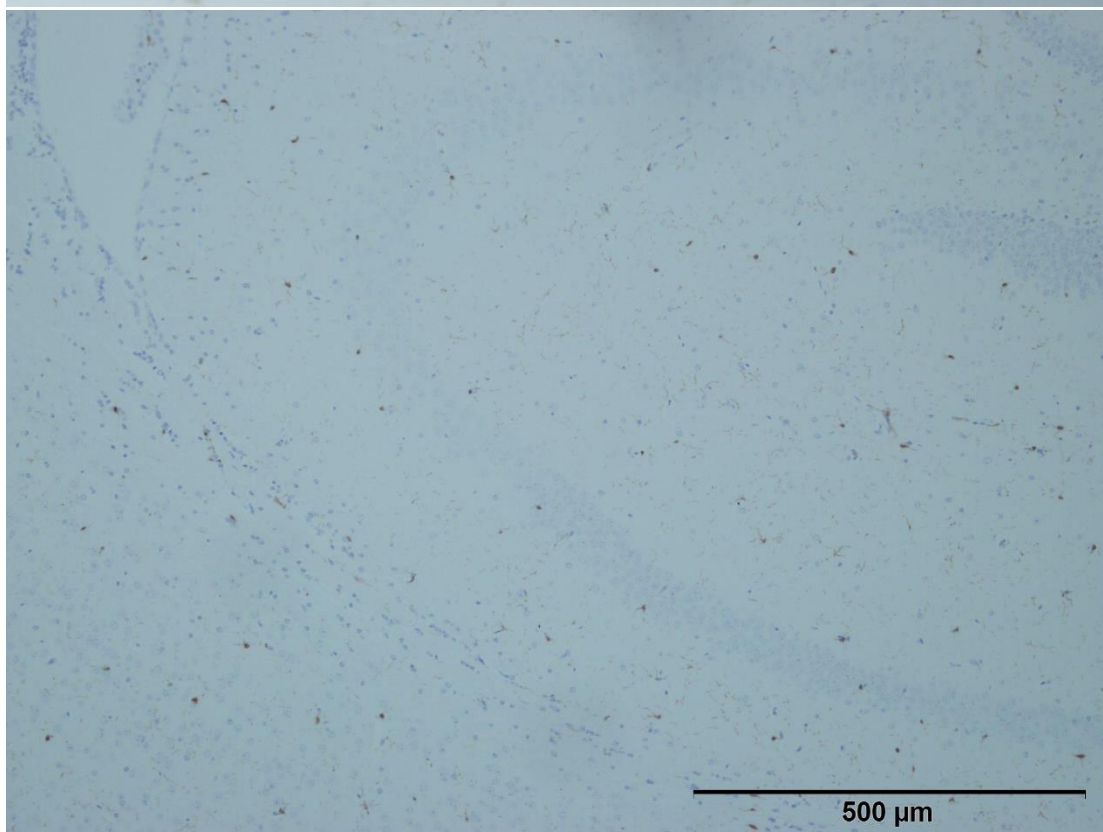

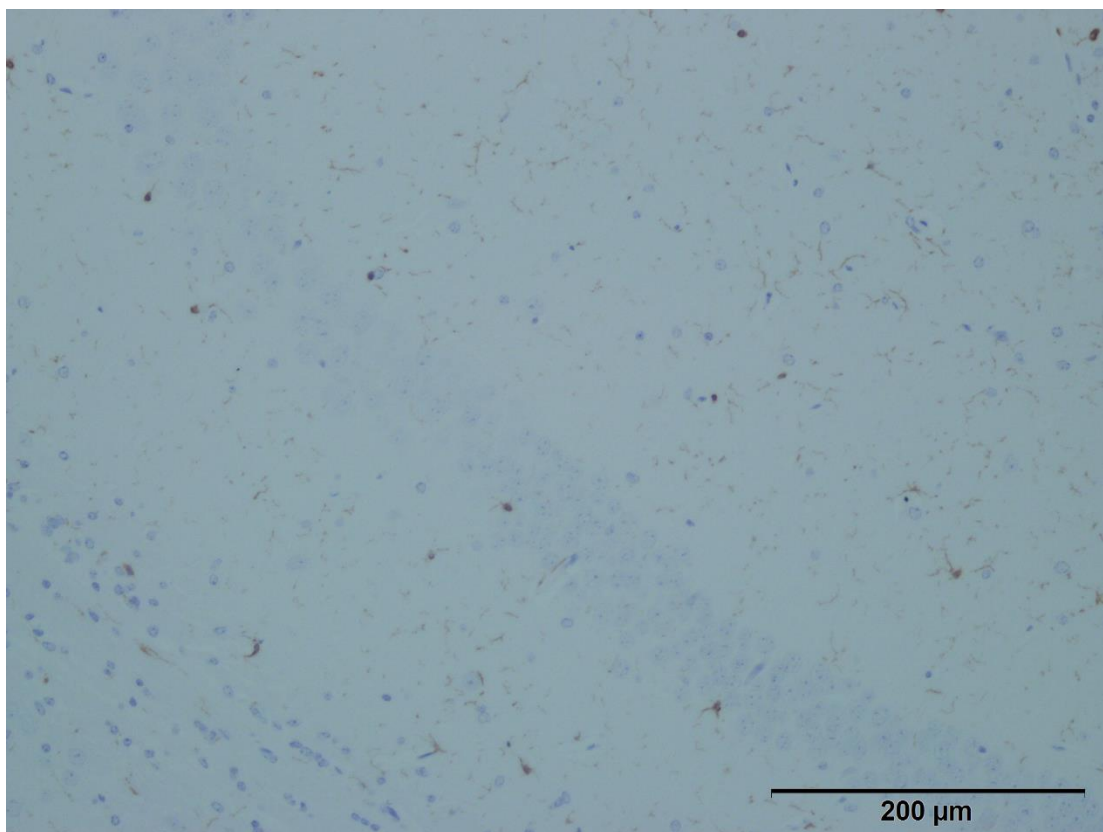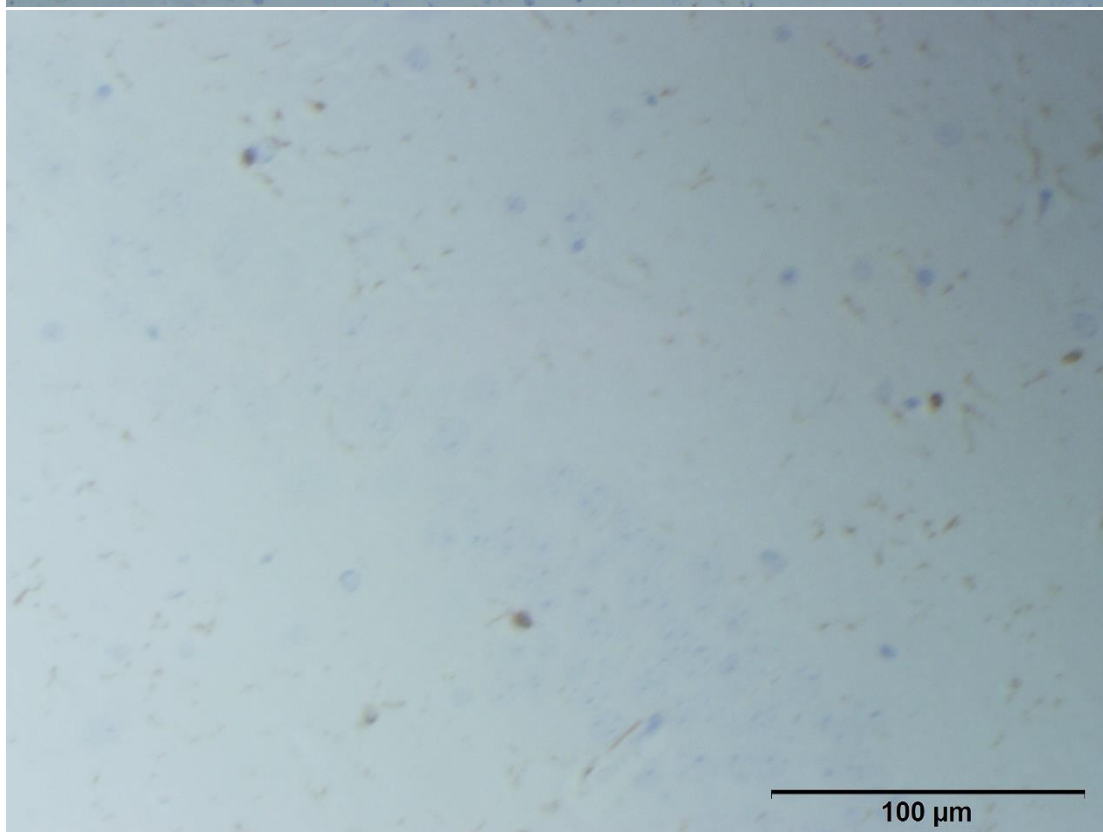

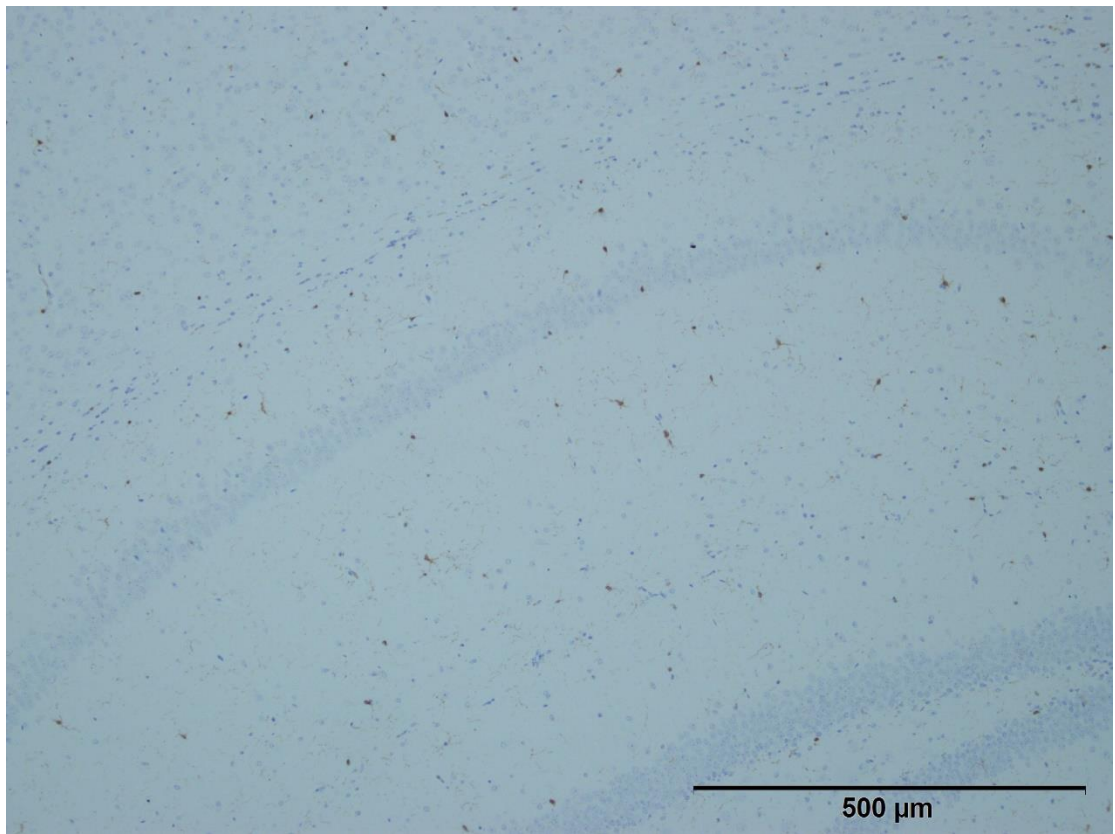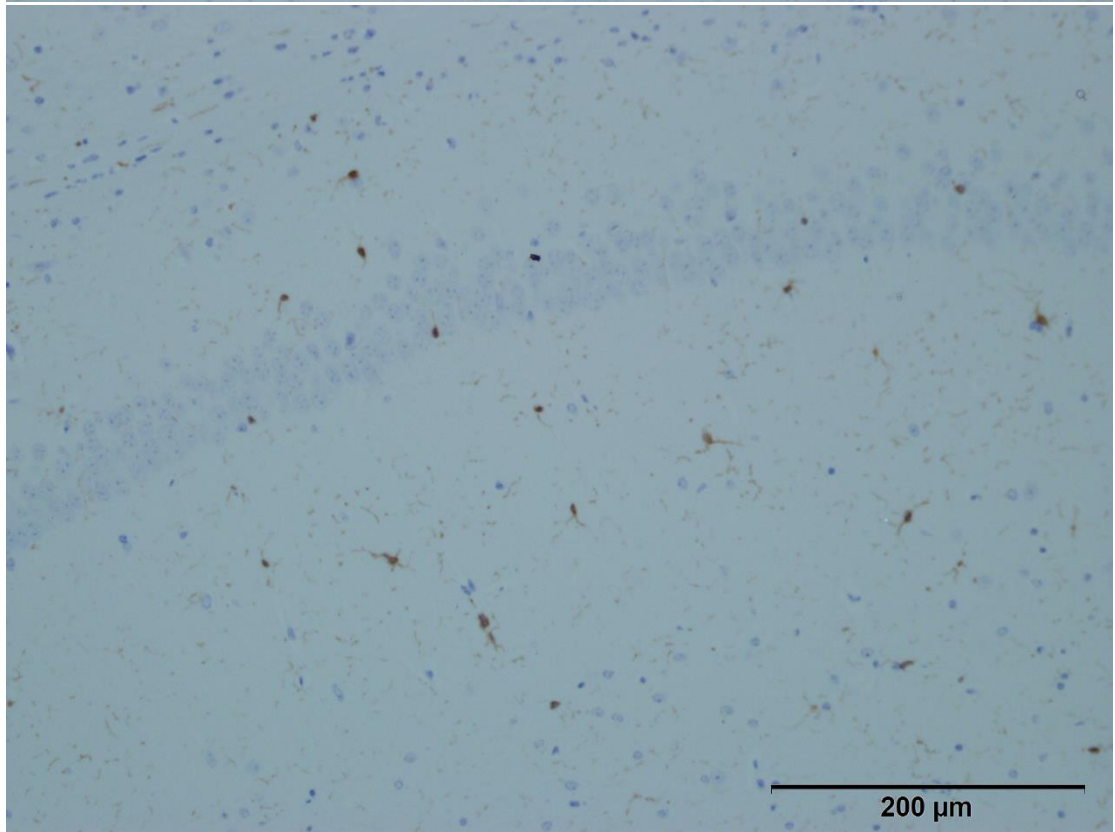

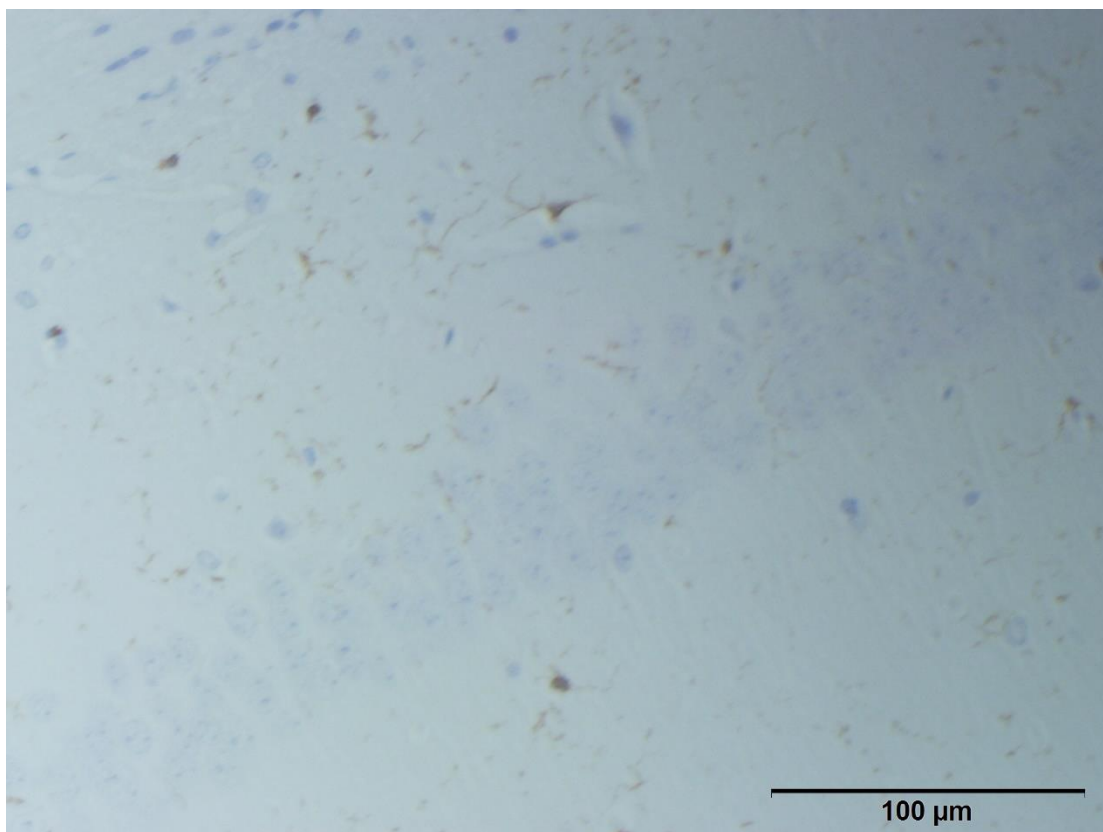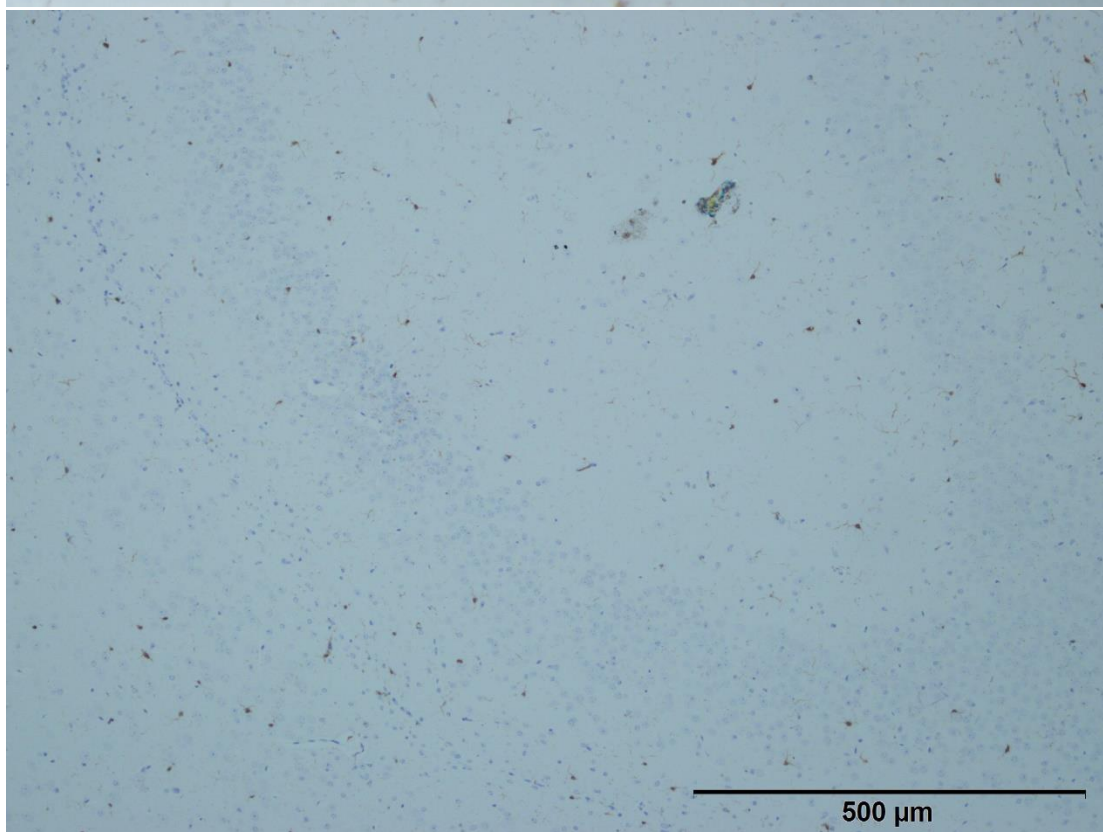

Supplement: Supplementary file 5 [file DataSheet3.PDF]
