# Supplementary material for: Antidepressant Mechanism of Traditional Chinese Medicine Formula Xiaoyaosan in CUMS-Induced Depressed Mouse Model via RIPK1-RIPK3-MLKL Mediated Necroptosis Based on Network Pharmacology Analysis
Source: Front Pharmacol. 2021 Nov 19;12:773562. doi: 10.3389/fphar.2021.773562 (PMC8641697; doi:10.3389/fphar.2021.773562)

WESTERN BLOT Exhibition

**ACTB** 42 kDa Anti-beta Actin antibody (ab8227)













**RIPK1** 76 kDa Anti-RIP抗体[7H10] (ab72139)

**



**

**





**

**RIPK3** 57 kDa Anti-RIP3抗体(ab56164)













**P-mlkl** 54 kDa Anti-MLKL (phospho S345) antibody [EPR9515(2)] (ab196436)














**MLKL** 54 kDa Catalog Number: orb32399




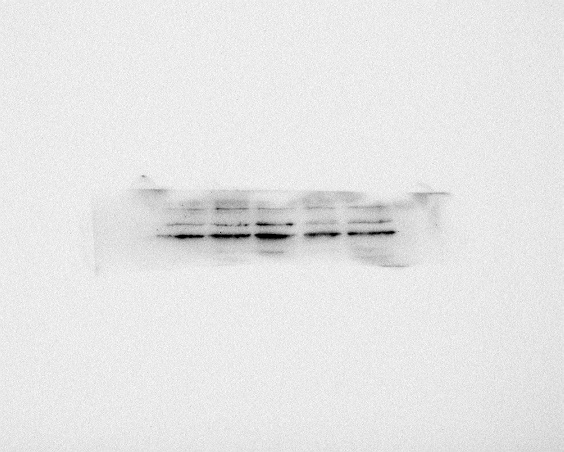








IL-1B 31 kDa Anti-IL-1 beta抗体(ab9722)















IBA1 17 kDa Anti-Iba1抗体[EPR16589] (ab178847)














LCN2 23 kDa Anti-Lipocalin-2 / NGAL抗体[EPR21092] (ab216462)




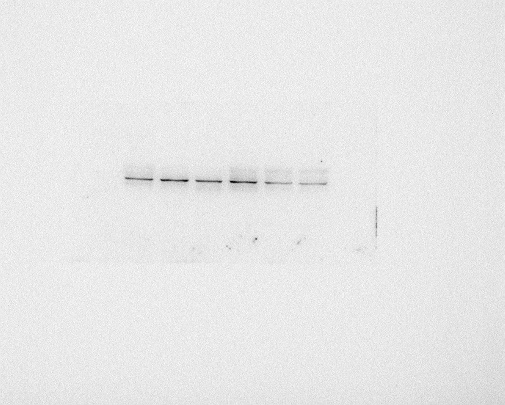







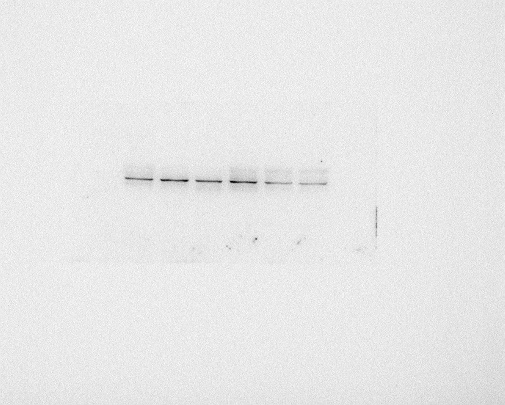

Supplement: Supplementary file 7 [file DataSheet7.ZIP › Data Sheet 1.DOCX]
